# Supplementary material for: Examining and classifying reasons for missing viral loads among adults living with HIV: An extended outcome investigation and ascertainment approach in Western Kenya
Source: PLOS Glob Public Health. 2025 May 12;5(5):e0004038. doi: 10.1371/journal.pgph.0004038 (PMC12068717; doi:10.1371/journal.pgph.0004038)
Supplement: S1 Protocol — (DOCX) [file pgph.0004038.s001.docx]

# TITLE OF THE PROJECT

An Adaptive Strategy for Preventing and Treating Lapses of Retention in HIV Care for Adolescents: ADAPT for Adolescents (A4A)

INVESTIGATORS AND INSTITUTIONAL AFFILIATIONS

| Site Principal Investigator | Elizabeth Bukusi, MBChB, M. Med, MPH, PhD, PGD *Senior Principal Clinical Research Scientist and Deputy Director (Research and Training),*  *Co-Director Research Care Training Program, Center for Microbiology Research, KEMRI* |
| --- | --- |
| Multiple Principal Investigators: | Elvin Geng, MD, MPH  *Professor, Internal Medicine – Infectious Disease*  *Washington University (WU), St. Louis, Missouri*  Lisa Abuogi, MD, MS  *Associate Professor, Divisions of Infectious Diseases*  *Department of Pediatrics, University of Colorado, Denver* |
| Co-Investigators: |  |
|  | Zachary Kwena, PhD  *Research Scientist*  *Center for Microbiology Research (CMR), KEMRI* |
|  | Lina Montoya, PhD  *Post Doctoral Researcher, Department of Biostatistics*  *University of North Carolina at Chapel Hill* |
|  | Starley Shade, MPH, PhD  *Associate Professor, Department of Epidemiology and Biostatistics*  *University of California, San Francisco* |
|  | Eliud Akama, MPH  *Research Scientist*  *Center for Microbiology Research, KEMRI* |

**TABLE OF CONTENTS**

[1.0](#_heading=h.gjdgxs) TITLE OF THE PROJECT 1

[2.0](#_heading=h.1fob9te) ABSTRACT 3

[3.0](#_heading=h.3znysh7) LAY SUMMARY 4

[4.0](#_heading=h.2et92p0) BACKGROUND 6

[5.0](#_heading=h.3dy6vkm) JUSTIFICATION 7

[6.0](#_heading=h.4d34og8) HYPOTHESES 9

[7.0](#_heading=h.2s8eyo1) STUDY OBJECTIVES 9

[7.1](#_heading=h.17dp8vu) GENERAL OBJECTIVE 9

[7.2](#_heading=h.26in1rg) SPECIFIC OBJECTIVES 9

[8.0](#_heading=h.2jxsxqh) DESIGN AND METHODOLOGY 10

[8.1](#_heading=h.z337ya) STUDY DESIGN 10

[8.2](#_heading=h.3j2qqm3) STUDY SITE 11

[8.3](#_heading=h.4i7ojhp) STUDY POPULATIONS 12

[8.4](#_heading=h.2bn6wsx) SAMPLE SIZE 13

[8.6](#_heading=h.qsh70q) STUDY MEASURES 27

[9.0](#_heading=h.3as4poj) ETHICAL CONSIDERATIONS 32

[9.1](#_heading=h.1pxezwc) ETHICAL APPROVAL 32

[9.2](#_heading=h.49x2ik5) HUMAN SUBJECT INVOLVEMENT AND CHARACTERISTICS 32

[9.3](#_heading=h.2p2csry) PROTECTING PRIVACY AND CONFIDENTIALITY 34

[9.4](#_heading=h.147n2zr) REFERRAL FOR ACUTE HEALTH CARE 34

[9.5](#_heading=h.3o7alnk) POTENTIAL RISKS TO STUDY SUBJECTS 35

[9.6](#_heading=h.23ckvvd) ADEQUATE PROTECTION AGAINST RISKS 36

[9.7](#_heading=h.ihv636) DATA SAFETY AND MONITORING PLAN 37

[9.8](#_heading=h.32hioqz) POTENTIAL BENEFITS OF PROPOSED RESEARCH 38

[10.0](#_heading=h.1hmsyys) DATA MANAGEMENT 40

[10.1](#_heading=h.41mghml) DATA COLLECTION AND STORAGE 40

[10.2](#_heading=h.2grqrue) STATISTICAL ANALYSIS 41

[11.0](#_heading=h.2u6wntf) TIME FRAME 43

[12.0](#_heading=h.19c6y18) EXPECTED APPLICATION OF THE RESULTS 44

[13.0](#_heading=h.28h4qwu) REFERENCES 44

[14.0](#_heading=h.20xfydz) BUDGET 49

[14.1](#_heading=h.4kx3h1s) BUDGET JUSTIFICATION 50

[15.0](#_heading=h.302dr9l) INVESTIGATOR ROLES 55

[16.0 APPENDICES 57](#_heading=h.1f7o1he)

# ABSTRACT

Adolescents and young adults (AYA) aged 14-24 years with HIV in Africa experience substantially higher rates of viral failure and HIV-related mortality as compared to adults. Thus, effective public health strategies tailored for and tested in this age group are urgently needed. The physical, psychological and social transitions faced by AYA create unique susceptibilities to prevalent and formidable structural (e.g., transport costs), psychosocial (e.g., the desire to fit in with peers, stigma) and clinic-based (e.g., unfriendly providers, long waiting times) barriers to engagement in public health HIV treatment settings. The variability in the intensity and the nature of barriers, however, poses a critical challenge: if barriers are highly variable, then no individual interventions are needed by all, while they also fail to help all in need. Adaptive strategies represent a novel approach to such problems with no “silver-bullet” solutions, and which we hypothesize have particular relevance for engagement of AYA in HIV treatment. Adaptive strategies typically begin with a less intensive intervention, and then escalate to a more intensive intervention only in those not doing well. We will use developmentally appropriate interventions that will be tailored by AYA pre-implementation through formative work. We will randomize 880 AYA with HIV in Kenya to either (1) youth-centered education & counseling (standard of care) vs. (2) addition of a SMS and peer electronic navigator (e-Nav) who provides support, information and counseling via phone. Those with a lapse in engagement will be re-randomized a second time to one of three higher-intensity re-engagement interventions: (1) standard of care outreach and intensified counseling (SOC-OIC), (2) conditional cash transfers (CCT) and (3) in-person peer navigation (IP-Nav). This study will quantify the relative effectiveness (and cost effectiveness) of several strategies composed of promising individual interventions. We will assess provider and patient experiences and satisfaction with the interventions using mixed methods. This innovative study will offer relevant evidence for public health programming to end the Acquired Immunodeficiency Syndrome (AIDS) epidemic for AYA with HIV.

# LAY SUMMARY

Adolescents and young adults (AYA) aged 14-24 years with HIV in Africa experience higher rates of treatment failure and HIV-related mortality as compared to adults. The physical, psychological and social changes they experience create barriers to engagement in HIV care and treatment. Some AYA do very well in HIV treatment without much additional support, while others struggle. This study will try to prevent HIV treatment challenges using an mHealth peer support approach and then increase to more intensive support through in-person peer support or cash transfers in those AYA who struggle.

Specifically, we propose an adaptive strategy that typically begins with less intensive retention interventions and escalates to more intensive intervention only among those not doing well. We will use developmentally appropriate interventions that will be tailored by AYA. We will randomize 880 AYA with HIV in Kenya to either (1) youth-centered education & counseling (standard of care) vs. (2) addition of a SMS and peer electronic navigator (e-Nav) who provides support, information and counseling via phone. Those who miss their clinic appointments by more than 14 days or have a high viral load will be re-randomized a second time to one of three higher-intensity re-engagement interventions: (1) standard of care outreach and intensified counseling (SOC-OIC), (2) conditional cash transfers (CCT), and (3) in-person peer navigation (IP-Nav). The primary outcome will be viral load suppression.

ACRONYM

| A4A  AE | ADAPT for Adolescents  Adverse Events |
| --- | --- |
| ART | Antiretroviral Therapy |
| ARV  AYA | Antiretroviral  Adolescents and Young Adults |
| CDC  CCT | Centers for Disease Control  Conditional Cash Transfers |
| CI | Confidence Interval |
| CMR  DCE  DREAMS | Center for Microbiology Research  Discrete Choice Experiment  Determined, Resilient, Empowered, AIDS-free, Mentored and Safe Lives |
| EMR  EMRS  E-Nav  ERR | Electronic Medical Record  Electronic Medical Record System  Electronic Navigator/Navigation  Enhanced Retrospective Review |
| FACES | Family AIDS Care and Education Services |
| HIV | Human Immunodeficiency Virus |
| ID  IP-Nav | Identification number  In Person Navigator/Navigation |
| IRB | Institutional Review Board |
| KEMRI  KenyaEMR | Kenya Medical Research Institute  Kenya Electronic Medical Record |
| MOH | Ministry of Health |
| NIH  ODK  OpenMRS | National Institutes of Health  Open Data Kit  Open Medical Records System |
| PEPFAR | President’s Emergency Plan for AIDS Relief |
| PI | Principal Investigator |
| SMS  SOC-OIC | Short Messaging Service  Standard of Care Outreach and Intensified Counseling |
| UCSF  UCD | University of California, San Francisco  University of Colorado Denver |
| UNAIDS | Joint United Nations Program on AIDS |
| WHO | World Health Organization |

WU Washington University, St. Louis, Missouri

# BACKGROUND

While the global response to HIV has reached close to 20 million people with antiretroviral therapy (ART) and saved upwards of 60 million life-years, progress has been uneven: adolescents and young adults (AYA) aged 14-24 years are being left behind. Compared to adults, AYA with HIV face more numerous, diverse and intense barriers to adherence and retention. While promising, evidence-supported interventions to engage AYA with HIV exist (e.g., SMS (text) messages, peer navigation, and conditional cash transfers), each intervention addresses only some of the developmentally-specific psychosocial (e.g., transitions, caregiver change), structural (e.g., school schedules, access to resources), and clinic-based (e.g., lack of youth friendly services) barriers to care. As a result, loss to follow-up and viral failure remain 25%-50% higher among AYA than among adults, and AYA are the only age demographic in which HIV mortality is rising globally.

The diversity of barriers to engagement in AYA make “silver bullet” solutions doubtful and demand innovative approaches. Instead of testing one particular intervention, therefore, we propose evaluating a set of *sequential* *adaptive strategies* for engagement that directly target AYA challenges, in which use of interventions over time depend on patient response, therefore offering an approach that acknowledges and responds to the individual [[1-3](#_heading=h.nmf14n)]. A typical adaptive strategy for AYA engagement would start by offering all patients a lower intensity (and cost) intervention (e.g., routine counseling, SMS messages) and then augment to a more intensive approach (e.g. case management, conditional cash transfer) only in those who fail to remain engaged. Maintaining initial interventions in AYA doing well optimizes efficiency, while intensifying only among those who lapse optimizes effectiveness. Sequential multiple assignment randomized trials (SMART) represent an emerging, rigorous and gold-standard design for assessing adaptive strategies [[2](#_heading=h.37m2jsg), [4](#_heading=h.46r0co2)].

**Our overarching *scientific premise* postulates that traditional trial designs which randomize patients to a fixed, static intervention are imperfectly equipped to address problems such as AYA engagement in HIV treatment where individuals experience varying outcomes and varying barriers to success.** AYA face unique challenges to engagement that vary in both intensity (some do well with minimal additional services while others will require strong efforts to engage) and nature (some face structural while others social barriers). In addition, demographic or clinical characteristics provide little information on whether an individual AYA will respond to a particular intervention for engagement, even when that intervention is supported by evidence. This means that no one intervention will work for all (or even many). Yet to date, the vast majority of trials for engagement in HIV treatment randomize all patients in an arm to a single, fixed intervention [[5-11](#_heading=h.2lwamvv)]. In contrast, research to identify adaptive strategies, each composed of a sequence of evidence-supported interventions, can revolutionize public health strategies for engagement in HIV care for AYA.

**Adolescents and young adults (AYA) are left behind in the global HIV response; the current proposal focuses on this vulnerable population.** AYA with HIV experience worse outcomes than either children or adults with HIV across the HIV care cascade [[12](#_heading=h.3ygebqi), [13](#_heading=h.2dlolyb)]. Recent population-based surveys in southern Africa suggest that about three-quarters aged 15-24 years are virologically suppressed, compared to 90% of adults [[14](#_heading=h.sqyw64)] and, unlike other demographic groups, mortality among AYA with HIV continues to increase [[15](#_heading=h.3cqmetx)]. Poor retention (loss to follow-up is 2-3 fold higher in AYA than adults) and adherence among AYA are major drivers of these disparities [[13](#_heading=h.2dlolyb), [16-18](#_heading=h.1rvwp1q)] and result from a wide range of structural (e.g., lack of money for transportation), clinic-based (e.g., lack of youth-friendly services), and psychosocial (e.g., depression, desire for peer acceptance, stigma) barriers encountered disproportionately by AYA [[19](#_heading=h.1664s55)]. Thus, there is urgent need for development and testing of AYA-tailored strategies, such as those proposed in this study, to improve care engagement of AYA.

**Innovative “youth-centered” models of care are necessary but insufficient: this proposal tests individual-level interventions that can be delivered in the context of facility-level approaches to further optimize engagement and viral outcomes among AYA.** Although clinic-level interventions such as designated “youth” days, youth-specific facilities, and youth-friendly care have shown some success, loss to follow-up remains high even with these interventions [[20-22](#_heading=h.3q5sasy)]. Cluver and colleagues identified several clinic-level factors associated with improved retention for AYA in South Africa in a dose-response manner, including devoting sufficient time to adolescents and adolescent-friendly attitudes among health care workers [[23](#_heading=h.34g0dwd)]. However, even when all factors were present, retention only reached 70% after 1-2 years. Our study will be carried out in a network of clinics in Kisumu County, Kenya, where facilities have youth-centered clinics, but where retention is still not optimal – around 50% of AYA treated in these clinics have lapses in retention or viral failure each year [[24](#_heading=h.1jlao46)]. We will test interventions that build on this foundation of youth-friendly care.

# JUSTIFICATION

**Adaptive interventions tested in a SMART are poised for rapid “translation” into real world practice.** A key limitation of traditional studies comparing two interventions is that in practice, several interventions each with some evidence of efficacy are often available. The question in practice is not just whether “A” is better than “B,” but, how “A” and “B” should be used together. For example, starting with both and then withdrawing one in patients who are succeeding may be better than starting with one and adding the other among failures. Practitioners often choose from evidence-based interventions but do not have evidence about how to use them together to achieve optimal outcomes. A SMART evaluates approaches which are closer to practice because they provide explicit guidance on how best to combine interventions based on patient response.

**Our Stage 1 electronic-navigation intervention marries two promising innovations – electronic communication platforms (SMS messages, phone calls) with peer support to efficiently extend the reach of social influences.** Mobile phone coverage in Africa rose from 0% to 60% between 2001 and 2012, and has reached nearly 80% in Kenya and 90% in Kisumu County [[25](#_heading=h.43ky6rz)]. Text messaging improves adherence to ambulatory care and medications [[6](#_heading=h.111kx3o), [9](#_heading=h.4k668n3)] and has positive effects on clinical follow-up after male circumcision [[26](#_heading=h.2iq8gzs)] and completion of early infant diagnosis for HIV [[25](#_heading=h.43ky6rz)]. Several studies reveal that SMS may be strengthened with interactive platforms and phone calls to promote engagement [[27](#_heading=h.xvir7l), [28](#_heading=h.3hv69ve)]. At the same time, a broad literature on “norms” and developmental theory emphasizes the importance of peers on AYA behaviors, particularly as regards to health [[29-31](#_heading=h.1x0gk37)]. Our E-Nav intervention combines these two approaches to achieve the best of both worlds: the power of peers with the reach of mHealth.

**Incentives for AYA in Africa have demonstrated behavioral effects in HIV prevention, but their role in enhancing engagement in HIV treatment has not been fully investigated [**[**32-35**](#_heading=h.1baon6m)**].**  Economists theorize that individuals often place disproportionate weight on present costs and benefits relative to those in the future [[36](#_heading=h.39kk8xu)], resulting in a tendency to procrastinate activities with immediate costs and delayed benefits [[36](#_heading=h.39kk8xu), **Error! Hyperlink reference not valid.**]. Incentives such as CCT aim to “nudge” individuals toward adopting a healthy behavior by increasing its immediate benefits [[38](#_heading=h.48pi1tg), [39](#_heading=h.2nusc19)]. Alternatively, CCT may also allow households “social protection” to maintain health care when income is uncertain. In either case, empiric studies have shown incentives increase vaccination, reduce the use of addictive substances [[40-43](#_heading=h.1302m92)], keep poor children in school [[44](#_heading=h.319y80a), [45](#_heading=h.1gf8i83)], increase HIV testing [[43](#_heading=h.haapch)], and reduce risky sexual behavior and possibly HIV infections among young women [[7](#_heading=h.3l18frh), [46](#_heading=h.40ew0vw), [47](#_heading=h.2fk6b3p)]. Based on this evidence, the Determined, Resilient, Empowered, AIDS-free, Mentored and Safe Lives (DREAMS) initiative within the U.S. President’s Emergency Plan for AIDS Relief (PEPFAR) uses CCT as a part of its core package of HIV prevention interventions for adolescent girls and young women [[48](#_heading=h.upglbi)]. Incentives, however, have to date, not been used to address engagement in HIV treatment over time among AYA with HIV.

**This study team has demonstrated capabilities to *safely* implemented conditional cash transfers, SMS, and peer navigation in 18-24-year old’s in Kenya.** Our research team has executed studies (including randomized trials) using SMS for follow-up after male circumcision in Africa, prevention of mother-to-child transmission, sociometric mapping, and support of adult HIV adherence and retention [[25](#_heading=h.43ky6rz), [26](#_heading=h.2iq8gzs), [49-52](#_heading=h.3ep43zb)]. Even though we targeted adults in a SMART for retention in Kisumu County, we were able to deliver CCT, SMS, and peer navigation with the same level of fidelity to patients aged 18-24 years as older adults. For example, among 18-24-year old’s in the CCT arms, 95% received their CCT. Even though in theory, cash transfer could lead to robbery or bullying, we have not detected adverse effects in 160 AYA randomized to CCT. Furthermore, we have conducted interviews on patient experiences with CCT among 15 young adults (18-24 years) and found no reports of coercion or negative experiences. Likewise in the current SMART in the field, our ability to implement a personalized automated SMS system was not different between those 18-24 years and older [[26](#_heading=h.2iq8gzs), [53](#_heading=h.184mhaj)].

**We have previously carried out design workshops to tailor engagement interventions to patient preferences for adults and pregnant women in Kenya and plan a similar process in this study.** For interventions (e.g., SMS, navigation, CCT) to be most effective, the frequency, content, and nature should conform to patient needs, desires and circumstances. In previous work with mHealth intervention for engagement, we used mixed methods to adapt SMS content to subgroups by age, sex, pregnancy status and language [[54](#_heading=h.3s49zyc)]. Patients, for example, did not want to use “HIV” or “clinic” in communications and proposed instead a set of “code” words for messages to preserve confidentiality in case others gained access to phones. We will repeat these design approaches in this study to enhance and tailor the interventions.

# HYPOTHESES

We hypothesize that electronic peer navigation (e-Nav) will result in the lowest risk of initial care engagement lapse during the first year after study enrollment. We hypothesize that by addressing multiple barriers to care, in-person navigation (IP-Nav) and conditional cash transfers (CCT) will result in more time spent in HIV care with suppressed viral load among AYA experiencing an initial lapse as compared to the standard of care. Finally, we hypothesize that sequential adaptive strategies that combine low intensity interventions for all patients to prevent initial lapses, followed by higher intensity interventions for patients experiencing an initial lapse, will be effective and cost effective for achieving retention and viral suppression over two years.

# STUDY OBJECTIVES

## GENERAL OBJECTIVE

This study will determine the comparative effectiveness of six such adaptive strategies for engaging AYA in HIV treatment against standard of care to prevent initial engagement lapses (defined as missed clinic visits and virologic failure) and treat those that occur. We will also explore secondary analysis to target specific adaptive strategies to particular subgroups, based on age, sex, and CD4 levels – thus potentially further extending tailoring of strategies to individuals.

## SPECIFIC OBJECTIVES

**Aim 1: Assess the effectiveness of standard of care routine education and counseling vs. electronic navigation for preventing lapses in engagement among 880 AYA living with HIV in Kenya (Stage 1).** We plan simple randomization of both newly initiating and treatment-experienced AYA to these two interventions. Routine counseling follows “youth centered” guidance from the Kenyan Ministry of Health. The E-Nav will pair AYA with sex and age-appropriate treatment-experienced peers who will provide patient-tailored psychosocial support primarily by phone calls and SMS. AYA without mobile phone communication access will be placed in the SOC arm. The primary outcome will be a lapse in engagement, defined as either missing an appointment (either clinical or pharmacy) by 14 days *or* unsuppressed HIV RNA following current MOH guidelines during routine monitoring, *or* death.

**Aim 2: Assess the effectiveness of standard of care outreach and intensified counseling, a conditional cash transfer or an in-person peer navigator to re-engage AYA after a lapse in engagement to achieve viral suppression (Stage 2).** AYA who experience a lapse of engagement (missed visit, high VL, or clinically documented non-adherence) in the first year on treatment will be randomized to standard of care (tracing limited to first 72 hours after missed visit), a CCT for making visits and viral suppression, or an IP-Nav who can form a more intensive, longitudinal relationship to solve complex barriers. The primary outcome will be viral suppression six months after re-randomization.

**Aim 3: Assess the effectiveness and cost effectiveness of six strategies for engagement of AYA based on combinations of two Stage 1 interventions (SOC-REC, e-navigation) with three Stage 2 interventions (SOC-OIC, conditional cash transfer, in-person peer navigation) on sustained viral suppression and engagement in care.** The best Stage 1 intervention paired with the best Stage 2 intervention may not yield the best overall strategy because the nature and number of failures can differ and interventions may influence effects of subsequent interventions. The primary outcome will be an indicator of sustained viral suppression and engagement in care measured at the end of Year 2 of the study. We power our study to detect four pair-wise comparisons between navigator-based adaptive strategies against SOC-REC followed by SOC-OIC for failures.

In addition, we examine the hypothesis that navigation in both forms can create skills that will have durable effects and enhance long term retention even after the intervention comes to an end. We propose to follow patients assigned to E-Nav and IP-NAV for an additional two years. The primary outcome will be sustained viral suppression over time (VL < 200 copies/ml) and sustained retention in care (no missed visits > 14 days) stratified by type of navigation received. No additional intervention will be provided after Year 2 end of study.

**Aim 4: Assess the satisfaction and provider-patient experiences of the AYA randomized to the three active strategies for engagement in HIV care (e-navigation, conditional cash transfer, and in-person peer navigation).** Understanding how the approaches were received, perceived helpfulness and influence on engagement in care, challenges encountered, and suggestions for improvement will inform findings. We will assess implementation and provider-patient experiences through qualitative interviews with a subset of 60 AYA (20 e-NAV, 20 CCT, and 20 IP-NAV) and all navigators.

In addition, we propose to interview a sample of AYA (N=12-20) and peer navigators (N=6-8 from each study) to assess patient and peer provider perceptions of skill formation and creation of resilience as well as challenges and unintended consequences. Similarly, we will carry out focus groups with study site staff and research staff (N=2) to assess for both desirable and undesirable unexpected outcomes from lay health worker interventions.

**Aim 5: Determine impact of interventions on care transitions among adolescents.** AYA peer navigation interventions (both electronic and in-person versions) are intended to assess transition readiness and address barriers to transition to adult care clinics . We will identify a sub-sample of AYA participants who transition from youth clinic to adult clinics (N~100) and assess the impact of the interventions on successful transition defined as retained in care and virally suppressed at the end of Year 3. No additional data or participant contact is needed.

# DESIGN AND METHODOLOGY

## STUDY DESIGN

*Overview of Research Design:*

*Formative work:* We will first conduct formative work for the purposes of intervention tailoring, including Focus Groups, a Design Workshop, and a Discrete Choice Experiment (DCE).

Then, in Stage 1 of this SMART study we will randomize approximately 880 AYA to either (1) standard of care routine education and counseling (SOC-REC) or (2) E-Nav (Figure 1). AYA without phone access will be placed in the SOC-REC arm. Previous data suggests up to 40% of AYA in SOC either miss at least one appointment >14 days or demonstrate virologic failure as per MOH guidelines will meet criteria for a lapse in engagement. We estimate this may be reduced to 25% in E-Nav. Therefore, in Stage 2, approximately 300 patients will miss at least one appointment or have an unsuppressed plasma HIV RNA, or have clinically documented non adherence to treatment and these participants will be randomized a second time to (1) standard of care outreach and intensified counseling (SOC-OIC) (per Kenyan guidelines), (2) a conditional cash transfer (CCT) for re-establishment of care and re-suppression or (3) an age and sex matched IP-Nav 1:1:1. Those who are retained and suppressed one year after enrollment in SOC-REC or E-Nav will continue with routine clinical care and in study follow up measures for two years following study enrolment. After the intervention has been implemented, we will assess user and provider experiences through qualitative interviews and focus groups.

Our primary analyses will identify the best intervention at each stage, as well as carry out four pairwise comparisons between the navigator-based adaptive strategies (e.g., start with E-Nav and escalate to CCT only among those with a lapse in engagement) against the standard current practice, which is start with SOC-REC and escalate to SOC-OIC among those with a lapse in engagement.

| **Figure 1: Study design of the A4A study.** In Stage 1, AYA are randomized to standard of care education and counseling (SOC-REC) vs. the addition of electronic navigation (E-Nav) to prevent engagement failure defined as: (1) missed appointment >14 days, or (2) documentation of unsuppressed viral load after >3 months of treatment, or (3) documentation of non-adherence to treatment or (4) death. Disengaged patients who remain alive are re-randomized to one of three Stage 2 re-engagement interventions: (1) standard of care outreach and intensified counseling (SOC-OIC), (2) conditional cash transfer (CCT) for making visits and suppressed viral load or (3) an in-person peer navigator (IP-Nav). Those in stage 1 who are do not miss a visit by 14 days and are virally suppressed after one year in the study will transition to routine care and participate in study follow for two years following study enrolment. | |
| --- | --- |
|  |  |

## STUDY SITE

The study will be conducted at Kisumu County Ministry of Health (MOH) facilities. Kisumu County in western Kenya county provides HIV care to over 47,000 patients including 3,100 adolescent at 62 government health facilities. This region has the highest HIV prevalence in Kenya (16%). The clinics for conducting this study will be peri-urban and determined based on patient volume, availability of infrastructure, study logistics, and after discussions with health facility management. We plan to enroll 3 health facilities initially and up to 6 maximum until targets are achieved.

*Recruitment of participating heath facilities:* Health facilities for inclusion into the study will be selected by the Scientific Steering Committee (Geng, Abuogi, Odeny) in consultation with representatives from the targeted health facilities. The study team has already obtained a letter of support from the County Health Management Team (Appendix C) which will be sent to the responsible authorities at all potential health facilities. The investigators will recruit potential study sites by sending letters and/or requesting in-person or electronic meetings (telephone/zoom/Google etc.) with the responsible authorities at the selected health facilities. The letter or the call will inform the responsible authorities of the trial’s purpose and will include a request for formal communication agreeing to be part of the study. This process will ensure that the responsible authorities at the County level are aware and supportive of this study, in accordance with Ministry of Health in all our research and programmatic endeavors.

## STUDY POPULATIONS

The target population for this study includes adolescents, ages 14-24, living with HIV in Kisumu County.

*Individual participant inclusion criteria:*

*Formative work: Focus Group Discussions (FGD), Design Workshop, and Discrete Choice Experiment (DCE).*

*Focus Group Discussions:* To gather views and opinions on planned interventions, HIV-seropositive, 14-24 years of age*,* initiating ART or on ART for any length of time at the participating health facilities, living >6 months in Kisumu County in previous year for familiarity of local population, and awareness of HIV status or caregiver agrees to assisted disclosure will be included in the FGD discussions.

*Design Workshop:* A subset of the adolescents and young adults who participated in the FGDs and agreed to be contacted for the workshop will be included. In addition, all peer navigators will be included in the design workshop.

*Discrete Choice Experiment (DCE):* In our DCE, HIV-seropositive adolescents aged 14-24, initiating ART or on ART for any length of time at the participating health facilities, living >6 months in Kisumu County in the previous year will be asked to participate in the DCE.

*Aim 1:* Being HIV-seropositive, initiating ART or on ART for any length of time, 14-24 years of age, living >6 months in Kisumu County in previous year (in order to exclude temporary residents likely to migrate), awareness of HIV status or caregiver agrees to assisted disclosure, ability to read or be read SMS messages, and willingness to be contacted by clinic upon missed appointment. For those who share phones, disclosure of HIV status to that person will be required. Consent for home visits is routinely sought for clinical purposes and <1% of patients refuse. In addition, we will recruit participants who are lost to follow-up who meet the above criteria.

*Aim 2:* The patient population will include AYA from Aim 1 who are at least 14 days late for a visit *or* who have unsuppressed HIV RNA as per current MOH viral load threshold or clinically documented non-adherence to treatment prior to the first year of study enrollment or within the first year of through three months following the first year of study enrollment, who are alive, still residing in the area, and not transferred to another clinic at time of retention/suppression lapse.

*Individual participant exclusion criteria Aims 1 and 2:* Plans to move out of Kisumu County, shares a phone but has not disclosed to the person he/she shares a phone with, acutely ill and requiring hospitalization, absence of consent for those >18-24 and absence of caregiver consent or assent if the AYA is between ages 14 to <18, or participation in other studies with potential to influence retention behaviors. ADAPT-1 participants that are still age eligible for this study (estimated 20 people) will be excluded. Hospitalized patients who later recover will be eligible for enrolment at the first post-hospitalization clinic visit during which eligibility criteria are met.

*Aim 3:* To assess the effectiveness and cost effectiveness of six strategies for engagement of AYA, we will examine and compare combinations of two Stage 1 interventions (SOC-REC, e-navigation) with three Stage 2 interventions (SOC-OIC, conditional cash transfer, in-person peer navigation) on sustained viral suppression and engagement in care measured at the end of Year 2. Thus, the study population will be the same as that of Aim 1. We will follow AYA participants assigned to the e-Nav (N~300) and IP-Nav (anticipated N~90) for up to 2 additional years after the end of the intervention. E-Nav participants will be compared to Stage 1 SOC participants at the end of Year 3 while IP-Nav participants will be compared to all SOC participants in Stage 2 at Year 4.

*Aim 4:* To assess patient and provider experiences, a subset of 60 AYA receiving e-NAV, IP-NAV, or CCT arms of the study as well as peer navigators will participate in in-depth interviews. Intervention satisfaction and experience surveys will also be conducted among all AYA enrolled in the study at multiple timepoints. We propose to interview a sample of AYA (N=12-20) and peer navigators (N=6-8 from each study) to assess patient and peer provider perceptions of skill formation and creation of resilience as well as challenges and unintended consequences. Interview participants will be purposively selected to give diversity in sex, time on ART, and intervention arm. Peer navigators in the A4A study are AYA 18-30 years of age, living with HIV, with demonstrated treatment engagement and viral suppression. Peer navigator interview participants will be purposively recruited to reflect diversity in gender, age, and facility. To obtain perspectives from facility staff and research teams who oversee Peer navigator, focus groups with study site staff and research staff (N=2; 6-8 participants each) to assess for both desirable and undesirable unexpected outcomes from peer support interventions.

*Aim 5:* To determine the impact of interventions on care transitions among adolescents with HIV we will identify and follow a sub-sample of both A4A study participants who transition to adult clinic. We estimate at least 100 participants will transition during the study (through Year 2). AYA usually transition between 18-24 years of age.

## SAMPLE SIZE

*Formative work:* For intervention tailoring and refinement, three FGDs, one design workshop, and a DCE. The design workshop will include eight AYA participants per active intervention strategy: e-NAV, CCT, and IP-NAV. Each FGD will seek gender and age balance. We will purposefully sample among AYA to obtain diversity in age, gender, and care engagement status. We anticipate an adequate level of data for theoretical saturation (depth and diversity of findings) for our research purpose. The DCE will aim to recruit approximately 200 AYAs currently receiving HIV care and treatment at the study facilities. This estimate is based on a questionnaire comprising five incentive attributes, two hypothetical scenarios and 10 questions, using the formula N ≥ (500 x c)/(a x t) - where N is the number of participants, t is the number of questions, a is the number of alternative scenarios and c is the largest number of attribute levels for any one attribute, and when considering two-way interactions, ‘c’ is equal to the largest product of levels for any two attributes [[55](#_heading=h.279ka65)].

*Aim 1, stage 1:* Based on field data, we anticipate that 72% of 880 participants enrolled in the study will have a phone, and thus will be initially randomized to either the e-Nav arm or SOC (approximately N = 634). We anticipate approximately 5% of patients will be censored due to withdrawal or transfer in the first year. Of those randomized, we anticipate a combined one-year cumulative incidence of either a 1) 14 day lapse in care 2) incident viremia or 3) non-adherence to treatment 4) death to be 40% in the SOC-REC arm. Using an alpha of 0.05 for a two-sided hypothesis test, we will be powered at 98% to detect an anticipated absolute difference of 15% (60% consistently successful in the SOC-REC vs. 75% in the e-Nav arm). We anticipate that the number of an adequate level of data for theoretical saturation (depth and diversity of findings) for our research purpose.

*Aim 2, stage 2:* Anticipating a total of 836 patients who remain uncensored due to withdrawal or transfer at the end of Stage 1 of the study (after ~ 5% attrition), if 40% of patients in Stage 1 who are randomized to SOC and 25% who are randomized to E-Nav reach the failure endpoint, then approximately 300 patients will fail Stage 1 interventions and be re-randomized 1:1:1 to three Stage 2 interventions. Anticipating an additional 3% censored due to study transfer or withdrawal following Stage 2 re-randomization (yielding approximately 97 patients with outcomes observed per arm), a viral load suppression probability of 0.40 in the SOC-OIC, 0.60 for CCT and 0.80 for IP-Nav, then using two-sided hypothesis tests with error rate of 0.05, we have ≥80% power to detect a difference between either CCT and SOC-OIC or between IP-Nav and CCT.

*Aim 3:* The total sample size of 880 was chosen to ensure our ability to generate estimates of patient outcomes for six embedded response-based strategies with sufficient precision to meaningfully inform programmatic decisions. Our sample size considerations are thus based on estimates of the degree of precision we anticipate in estimates of our primary outcome were a given strategy to be applied to the whole population. There are several special considerations: 1) a given subject can contribute to estimation for more than one strategy; 2) the number of subjects available for estimation of the expected outcome under a given strategy depends on the probability of initial retention lapse or viral suppression under the first component of the strategy; and 3) estimates must account for the sequentially randomized design by upweighting non-censored failures (or using other methods). Our precision estimates were generated by applying the *ltmle* R package to multiple iterations of a simulated dataset (Lendle et al, 2017). Simulation assumptions were based on estimates of the proportion of sustained viral suppression and engagement in care at 2 years under each prevention and treatment combination using preliminary data from study sites. In particular, we expect that 28% of patients enrolled will not have a phone at enrollment; those participants will deterministically be given SOC as their initial treatment. In addition, participants who were allocated to standard of care (followed with standard of care outreach upon initial lapse if any) will have a probability of sustained viral suppression and engagement in care of 45% at two years follow up, and 8% of subjects will be censored annually due to migration, transfer out, or study withdrawal. Based on these simulations, we anticipate that our estimate of the proportion virally suppressed and engaged in care 1 year after rerandomization among failures and 2 years after study enrolment for those who do not fail will fall within 2.5 percentage points of the truth with 95% confidence (i.e., the absolute difference between our estimate and the truth was less than 5% in 95% of iterations of the simulated experiment). This level of precision provides a strong basis for decision making.

In addition, we will also test the null hypotheses of no difference in proportion of sustained viral suppression and engagement in care between subjects followed with standard of care (including SOC-OIC upon initial lapse if any) and subjects followed with navigator-based strategies. For these four 2-sided hypothesis tests, we anticipate approximately ≥80% power to detect the absolute difference in probability of sustained viral suppression and engagement in care at 2 years in each of these navigator-based arms compared to SOC throughout. Use of longitudinal Targeted Maximum Likelihood Estimation (TMLE) to adjust for covariates predictive of the outcome are expected to improve precision and power further.

We will follow AYA participants assigned to the e-Nav (N~300) and IP-Nav (anticipated N~90) for up to an additional 2 years after the end of the intervention. E-Nav participants will be compared to Stage 1 SOC participants at the end of Year 3 while IP-Nav participants will be compared to all SOC participants in Stage 2 at Year 4.

*Aim 4*: To assess patient and provider experiences, in-depth interviews will be carried out with a subset of 60 AYA randomized to receive one of the three active intervention strategies first three years of the study: 20 e-NAV, 20 CCT, and 20 IP-NAV. Purposeful sampling will be used as described in the formative work. We anticipate an adequate level of data for theoretical saturation (depth and diversity of findings) for our research purpose. In addition, approximately 10 in-depth interviews will be carried out among navigators. Intervention satisfaction and experience surveys will also be conducted among all AYA enrolled in the study at re-randomization and annually, power calculations for aims 1 and 2 will apply. Additionally, we propose to interview a sample of AYA (N=12-20) and peer navigators (N=6-8) to assess patient and peer provider perceptions of skill formation and creation of resilience as well as challenges and unintended consequences.

*Aim 5:* To determine the impact of interventions on care transitions among adolescents with HIV we estimate at least 100 participants will transition during the study (through Year 2).

- 1. **STUDY PROCEDURES**
     1. **ENROLLMENT**

Eighteen years of age is the age of majority in Kenya. Throughout this section if potential participants are ages 14 to 17 years old, we will have a legal guardian/caregiver involved in screening and consenting process, with AYA assent. AYA 18 to 24 years of age will be screened and consented as adults. In light of the emerging Corona Virus Disease 2019 (COVID-2019) we will minimize risk and spread by utilising electronic media for virtual sessions that allows for physical distancing where appropriate, screening staff and participants for COVID-19 symptoms or contact before any study related interactions, providing staff and study participants with necessary protective equipment, and providing handwashing and disinfection supplies.

*Formative:*

*Screening/recruitment:* Study staff will have access to a list of eligible AYA scheduled for the clinic on a given day. They will approach and screen potential eligible AYA (and their accompanying caregiver if appropriate) for FGD and/or HCD participation while they are waiting to be seen in the clinic. They may also reach out to potential study AYA/caregivers through community outreach using phone communication, during adolescent support groups sessions, and other planned adolescent activities. We will also use an alternative strategy where we will ask care providers at health facilities to inform eligible AYAs about the study and ask them if they would be willing to have someone from the research team call and give them more details about the study. Those interested will leave their phone numbers with providers to share with the research team. The research team will call and provide additional information about the study and ask AYA about their willingness to participate in the initial stage of study screening. For AYA less than 18 years of age, the caregiver will be contacted. Screening will consider the Ministry of Health (MOH) infection prevention measures for COVID-19 that include use of mobile phones to ensure physical distancing or maintaining distances of 1 meter, use of masks, and handwashing. Screening will involve asking AYA/caregiver discrete disclosure screening questions (Appendix B1) to prevent accidental HIV disclosure questions, asking about mobile communication device access and length of time living the area. If they are eligible, study staff will schedule a time for a FGD and/or HCD session that day or at later date.

*FGD:* The FGDs will either be conducted during an in -person session or through a virtual platform (e.g phone, WhatsApp, Skype, Zoom, or Google meet) based on COVID-19 prevention guidance. For virtual sessions, we will start by confirming participant identities using series of unique identifiers. For in-person FGD sessions, we will consider the COVID-19 infection prevention recommendations. We will identify large well aerated conference rooms that can accommodate 8 people while maintaining the Ministry of Health and WHO recommended 1 metre distance for participants. In addition, all FGD participants and facilitators will undergo temperature screening using non-contact digital thermometers, screening for symptoms of coughing and for history of travel to or from COVID-19 affected counties, or any known COVID-19 contacts. We will provide masks and handsanitzing facilities for all participants and facilitators. Study staff will request the caregiver to participate in the introductory parts of the session for consenting purposes if they are between 14 to less than 18 years of age. The FGD sessions will focus on gathering views about the planned intervention to inform intervention tailoring. If sessions are conducted virtually due to COVID-19, consent/assent will be obtained verbally. In such cases, study staff will read the consent form word-for-word and check for understanding prior to obtaining verbal consent. In person sessions will obtain written consent.

*Discrete Choice Experiments:* The DCEs will be conducted either during one on one in-person sessions during clinic visits or through a virtual platform e.g phone, whatsapp, skype, zoom or google meet. Research assistants will read out survey questions and show the participant a card which demonstrates to participants the DCE options for both scenarios in each of the 10 questions. RA’s will record the participants selection (answer) on a tablet which displays the same series of questions as presented on the DCE cards. Physical interactions with study participants will consider all the COVID-19 infection prevention practicises as prescribed by the Ministry of Health. Verbal consent/assent will be obtained for in-person and if carried out virtually and the study staff will read the consent form word-for-word and check for understanding.

*Design workshops:* The design workshop will focus on intervention tailoring and refinement based on FGD findings. Approximately four peer navigators and 8 AYA who either participated in the FGD or are recruited for the formative work as described above will be invited to participate in a five-day design workshop to tailor the planned interventions. For those recruited during FGDs for the HCD, screening will involve asking them during FGD consent if they are willing to be contacted to participate in the design workshop. Those that indicated yes, including applicable caregivers, on the consent form will be contacted by phone and invited to participate. Considering the COVID-19 infection prevention recommendations, we will identify large well aerated conference rooms that can accommodate 10 people while maintaining the WHO recommended 1 metre distance for participants. In addition, all HCD participants and facilitators will undergo temperature screening using non-contact digital thermometers, screening for symptoms of coughing and for history of travel to or from COVID-19 affected counties every day before the HCD session. We will provide masks and handsanitizing facilities for all participants and facilitators. In cases where we are not able to do a physical HCD meeting, we will utilize online electronic platforms (e.g phone, WhatsApp, skype. Zoom, or Google meet). We will provide virtual meeting infrastructure to participants that include laptops, tablets, phone and internet using the following models: a) participants with access to smartphones will receive bundles for internet, b) participants with no access to smart phone will be taken to a room set up with a computer or tablet connect to the internet and supported on using the selected virtual meeting platform. Verbal consent/assent will be obtained for in-person and if carried out virtually.

*Informed consent:* Interested and eligible AYA/caregivers, will either be invited that day or be invited to return at more convenient time for an upcoming scheduled focus group session either in-person or virtual. The session will be held in privacy to protect confidentiality. For virtual sessions, we will ask the participants to find a secure and private location with good network connectivity as well as blocked time. The informed consent/assent process will be conducted by trained research staff in the preferred local language (Kiswahili, Dholuo, English). The research assistant will carefully explain the nature and purpose of the study to the AYA/caregivers. Potential participants/caregivers will be given clear explanations that this is something separate from regular care and that they have the option of refusing to participate in any part of the research without any effect on their or their caregivers’ routine care received at the facility. They will be informed that they will participate in one up to 90-minute session. They will receive compensation for the time and/or travel, about Ksh 500. Focus group discussion*:* If the FGD session is in-person consent/assent will be written/signed and if virtual consent/assent will be obtained verbally. If virtual, the study staff will read the consent/assent form word-for-word, check for understanding, and if there is agreement to participate, the form will be ticked and the participant, and applicable caregiver, name printed on the consent form.

Design workshop and DCE: No individual health or other confidential information will be documented and discussions will focus on the intervention. As such, we do not consider the design workshop to pose more than minimal risk. A research assistant will administer a verbal consent/assent either in-person or virtual. This will be conducted prior to the design workshop to ensure voluntary participation and protection of privacy.

For the cessation of the intervention extension follow-up, these participants will be offered the opportunity to provide their consent through virtual means. This will be via phone call by a qualified research assistant Or a peer navigator. The consent forms will be translated into Dholuo and Swahili

*Aim 1: Screening/recruitment:* In the clinic: Screening and recruitment procedures will be as for the formative work described above. Study staff will have a screening list of potentially eligible AYA generated daily from the electronic medical record system (EMRS) and approach the AYA /care givers during their scheduled visit. Screening will involve asking AYA/caregivers eligibility criteria including: discrete disclosure screening questions (Appendix B1) to prevent accidental HIV disclosure questions, asking about mobile communication device access, intentions to reside in the area, and involvement in the prior ADAPT study (fewer than 20 from ADAPT are anticipated to be eligible for this study) (Appendix B2). Recruitment at the clinic will occur during waiting times to minimize disruption to patient flow. In the home or community: Screening and recruitment for AYA enrolled in care but lost to follow up (LTFU), a list of eligible AYA who have not attended a clinic visit for 90 days or more since their last scheduled visit will be abstracted monthly from the electronic medical records. Pharmacy and medical record charts will be reviewed to confirm non-attendance. Those confirmed to be LTFU will be traced. Following routine and well-established program practices for tracing LTFU AYA, the study team will make phone attempts and/or in-person visits to make contacts with the AYA/applicable caregiver to determine the AYA’s whereabouts, current care status, and to introduce the study. Both LTFU patients and for AYA 14-17 years of age who have attended the clinic but without a caregiver, study recruitment may happen in the home or community location if it is more convenient for the AYA/caregiver and there is sufficient privacy for consent/assent and confidential data collection.

*Informed consent:* Consent will occur as described above in the formative work, including in-person or through the phone for COVID-19 precautions. Consenting will occur in privacy room where a trained research assistant will conduct the informed consent process in the preferred local language. For phone sessions, we will ask the participants to find a secure and private location with good network connectivity as well as blocked time. The research assistant will carefully explain the purpose, nature, and methods of the study, interventions, randomization process, procedures to protect the confidentiality of the information, risks involved, and their rights to withdraw from the study at any time. Potential participants/caregivers will be given clear explanations that this is something separate from regular care and that they have the option of refusing to participate in any part of the research without any effect on their or (or applicable accompanying caregivers) routine care received at the facility. Adolescents ages 14-17 will be given the opportunity to provide assent for participation in the study. In the event that a caregiver is not present during recruitment of an adolescent 14-17 years of age, the research assistant will contact the caregiver over the phone for consent. Emancipated and mature minors will be allowed to consent on their own without parental/guardian permission. However, if they indicate the need to seek permission from their parents/guardians the study will allow them. According to the GOK/MOH SRH guidelines (<https://www.ncbi.nlm.nih.gov/books/NBK25556/>) a mature minor is 15 years of age or older; living separate and apart from their parents or guardian, whether with or without the consent of a parent or guardian and regardless of the duration of the separate residence, and managing their own financial affairs, regardless of the source of income. Emancipated minors are children freed from parental custody and control as result of a) marriage, b) court order, and c) military service.

They will be provided information at an age-appropriate level and given the opportunity to assent or decline separate from their caregiver. In the event that the caregiver and the AYA do not agree on study participation, study staff will attempt to understand and address concerns and answer any questions. If either caregiver or AYA still do not wish to participate in the study, the AYA will not be enrolled. They will be asked to sign an informed consent or assent form, or if illiterate, to provide a thumbprint in the presence of a non-study staff witness (the standard practice used in our other Kenyan studies, as approved by the KEMRI IRB). They will be informed that they will be followed up for 24 months.

A consent checklist will be added to the end of the consenting process to ensure that participants and caregivers understand the procedures to which they are consenting. The voluntary nature of the study will be emphasized to study participants. Specifically, we will assess the extent to which each participant is freely volunteering for participation in the study without coercion or pressure from clinic staff, spouses/partners, family members or others. Each consenting individual will be given a copy of the standard Bill of Rights for Patients from UCSF and from KEMRI and a copy of the consent and assent form.

Participants will receive reimbursement for their time and travel if they chose to enroll and complete the enrollment process, the amount will be based on the average costs of transport within the area of our operation other research incentives in the area, approximately Ksh 500.

Those who do not wish to participate, will continue to receive standard HIV services within the facility.

*Aim 4:*

*Screening/recruitment:* For the individual interviews, study staff will have a list of main study participants by intervention arm who are scheduled to return to the clinic. The first set of interviews in year one will focus on the aim 1 active intervention, e-NAV, arm participants for screening and recruitment. Then in years two and three, subsequent sets of interviews will focus on aim 2 active interventions, CCT and IP-NAV, arm participants for screening and recruitment. AYA/caregiver randomized to the intervention arm of focus will be approached while waiting to be seen in the clinic for a routine visit. Similarly, for interviews with participants who have experienced peer navigation to further explore mechanisms of action of peer navigation will be identified through study records. Recruitment will occur during clinic visits, by phone, or a s above, screening may also be conducted through community outreach (Appendix B33-B35). If AYA are less than 18 years of age, parents/caregivers will be asked to accompany them for screening or we may conduct outreach for screening at home if it is more convenient for the parent/caregiver.

Peer navigators who have delivered peer navigation to participants will be identified through study records. They will be recruited by an external qualitative researcher who is not a regular part of the study team via phone, email, or in person (Appendix B33-B35). As part of recruitment and informed consent (see below) they will be informed that participation is entirely voluntary and will not impact their employment status or performance reviews. The MOH COVID-19 infection prevention recommendations that advocate for minimizing physical contact will be considered for the individual interviews where appropriate. Virtual interviewing options that include phones, WhatsApp, skype. Zoom, or Google meet will be considered where possible, along with verbal consent.

Research and clinic staff involved in implementing and supervising peer navigation will be identified through clinic and study records. Potential participants will be recruited by an external qualitative researcher who is not a regular part of the study team via phone, email, or in person (Appendix B33-B35). As part of recruitment and informed consent (see below) they will be informed that participation is entirely voluntary and will not impact their employment status or performance reviews.

*Informed consent:*  Consenting will be conducted as described above in the formative work. Additionally, potential participants/caregivers will be informed that they will participate in one 45 to 60 minutes interview. They will receive compensation for the time and travel, about Ksh 500.

Peer navigators, research and clinic staff will be informed as part of consenting process, that participation is entirely voluntary. Participation (or non-participation) and views expressed will not be shared with supervisors or the study PIs and will not impact employment status or performance reviews. Data will be deidentified by the external qualitative research assistant prior to sharing.

*Aim 5:*

*Screening/recruitment:* New recruitment will not be required. Participants from Aim 1 who undergo transition will be identified through routine study data.

*Informed consent:* Informed consent obtained for Aim 1 discusses use of data collected for the study. No additional data will be collected and no new risks or benefits will arise as part of data analysis for this aim. Therefore, no consenting/re-consenting will be required for this aim.

- - 1. **FORMATIVE**

*Intervention tailoring:* Formative work will be carried out to tailor the e-NAV, CCT, and IP-NAV interventions to the population and local context as described previously. For example, whether the CCT should be offered to a caregiver or a patient will likely depend on the age and preferences in the communities. Intervention tailoring to local preferences will be conducted through three FGDs comprised of approximately eight AYA participants, one per intervention. The FGD will be carried out by trained qualitative staff in the local language. This will be followed by a three-day design workshop comprised of 4 peer navigators and 8 AYA who participated in the FGDs and a DCE targeting approximately 200 AYA. The design workshop will translate preferences into intervention activities (e.g. preference on counseling topics, communication frequency code words). The workshop will include design thinking strategy and iterative prototyping. Both processes will translate conceptual preferences into intervention design, for example, selection of SMS wording, language and tone preferences, and prioritization of anticipated social support needs (e.g. school, life transitions, partnerships, clinic access). A facilitator will probe, assess and evaluate behaviors of targeted groups of patients and peer navigators and assist with translating that knowledge into meaningful information and system designs to inform and improve the proposed interventions. The DCE will help determine AYA preferences for CCT delivery by examining preferences from 200 AYAs. This survey will present several hypothetical scenarios for delivering incentives to AYA, by varying the service attribute levels (eg, two scenarios with different incentive formats e.g., shopping vouchers versus cash). This experiment could demonstrate adolescents’ relative preferences and the amount of another attribute they would be willing to trade.

- - 1. **RANDOMIZATION**

*Aim 1, stage 1:* At stage 1, AYA who do not have access to phones will be allocated to receive standard of care (SOC) and those with consistent phone access will be randomly assigned with equal probability to one of the two Stage 1 arms. Consistent phone access will be defined as the ability to receive and send mobile messages and voice communication regularly. Study staff will carry a phone that has a pre-specified random intervention, which can be revealed when called by the field staff. As an alternative in case the phone system fails, A4A will received paper opaque, intervention assignments in sealed envelopes sorted in random order with back up on battery operated laptop maintained by the study project director. Randomization will be conducted after providing informed consent.

*Aim 2, stage 2:* All AYA who miss an appointment by 14 days in the first year of follow-up, has an unsuppressed HIV RNA based on the current MOH guidelines or clinically documented non-adherence prior to the first year of study enrollment or within and up through three months following the first year of study enrollment will be randomly assigned with equal probability to each of three re-engagement Stage 2 arms (SOC, CCT, IP-Nav). The re-randomization will be done with contact between day 15 and day 28 of missed visit or knowledge of high viral load. If not found, re-randomization will be done by the study staff on day 29 of missed appointment with no contact. All AYA eligible for second line randomization will be identified in real time by study staff based on facility EMRS records. Research staff will use a phone that has a pre-specified random intervention, which can be revealed when called up by the field staff. As a back up, paper opaque, sealed envelopes with each patient’s identifier on it will be used if the electronic system fails. Of note, if the patient is not found two weeks after failing Stage 1 interventions and is assigned to an IP-Nav, the “extra seeking” by the navigator is considered part of the effect of an IP-Nav.

- - 1. **PATIENT TRACING**

All AYA who miss an appointment by 14 days in the first year of follow-up or have a high viral load, based on MOH guidelines, or clinically documented non-adherence prior to first year of study enrollment or within and up through the 3 months following the first year of study enrolment will be traced. We will first try to contact the patient via phone call, and then failing that, via in-person tracing. When patients who missed a visit by 14 days are contacted, they will be assigned their second stage re-engagement intervention, which will be explained in detail. Also, AYA due for their routine viral load who have not returned or are missing their routine viral load, and those who have missed clinic appointments around their year 1 and 2 study enrollment anniversaries may also be traced. Patients who due to viral load will scheduled to return for viral load or if needed and preferred by the patient (and applicable caregiver if between 14 and 17 years old), their viral load will be conducted in the home by a trained and qualified staff (e.g. nurse, clinician, or phlebotomist). Our research group has carried out extensive tracing and is well-experienced in maintaining confidentiality to avoid disclosure of HIV status. If a patient is not contacted in person, a message with a name and number to call will be left, with no health-related information. Passive record review will continue for extended follow up of participants up to an additional 2 years after intervention delivery is complete at the end of year 2.

- - 1. **INTERVENTIONS**

**Aim 1: Assess the effectiveness of standard of care routine education and counseling vs. electronic navigation for preventing lapses in engagement among 880 AYA living with HIV in Kenya (Stage 1).**

Rationalization: Preventing missed visits, poor adherence and viremia is crucial to prevent immunological deterioration and drug resistance [[56](#_heading=h.meukdy), [57](#_heading=h.36ei31r)]. We will directly compare the effectiveness of SOC-REC vs. E-Nav.

AYA initiating ART or on ART for any length of time meeting other inclusion criteria will be randomized to either SOC-REC or e-NAV described below.

**Standard of Care Routine Education and Counseling (SOC-REC):**

*Rationale:* Education and counseling about the importance of visit and medication adherence is routinely provided in all Kenyan MOH facilities. SOC-REC is guided by Kenyan national guidelines on ART support and adherence counseling with specific guidance for AYA topics (e.g., reproductive health) and communication approaches (e.g., non-judgmental communication) (MOH Guidelines for Use of Antiretroviral Drugs in Kenya; Guidelines for Provision of Adolescent and Youth Friendly Services; Adolescent Package of Care in Kenya; https://faces.ucsf.edu/resources).

*Content:* SOC-REC is carried out according to Kenyan National Guidelines and includes standardized developmentally-appropriate, group-based education and counseling on HIV basics, treatment, and adherence. Additionally, all sites have been trained on the Kenya National Adolescent Package of Care which promotes adolescent friendly services [[58](#_heading=h.1ljsd9k)] but day-to-day implementation is variable. On “youth” clinic days, group education is done including HIV basics (e.g., principles of transmission), positive living, operational issues (e.g., visit adherence), psychosocial assessment and support, and ART treatment adherence as well as adolescent health topics such as sexual and reproductive health (SRH). One-on-one counseling may be available to some patients who need additional support. This intervention is offered routinely to all patients. We will standardize its application but not alter its content.

**E-navigation:**

*Rationale:* The E-Nav can address several medical, psychosocial, and structural barriers faced by AYA including lack of knowledge and need for peer support and norming, through promotion of self-efficacy and resiliency, and overcome clinic barriers by having navigators act as clinic liaison and advocate. It proactively supports patients by creating a longitudinal relationship through phone calls, SMS messages, and face-to-face interactions when needed at the facility. The E-Nav leverages (1) the fact that cell phone use among AYA in Kenya exceeds 90% and (2) a robust body of psychological literature demonstrating that social norms drive health-related behaviors and that adolescence is a period in which peer influences are particularly powerful.

**Content:**

*E-navigator background and training:* The navigators will possess secondary school education, fluency in local languages, and will be living with HIV with active engagement in HIV care. E-navigators will receive training to position them to counsel and assist AYA using a 10-day curriculum on adolescent counseling. The study team will adapt and advance curricular materials used previously with more training modules and greater one-to-one instruction. Training will include 1) adolescent friendly care (adapted Kenya national curriculum), 2) psychosocial counseling (stigma, disclosure, depression) and motivational interviewing (FACES Adolescent Peer Leader Training), and 3) study procedures prior to intervention implementation. We will match e-navigators to patients by gender and approximate age to the extent possible. Peer navigators themselves will be coached by an experienced adult professional peer counselor with formal training in counseling and minimum two years’ experience, who will facilitate case reviews, observe navigator-participant interactions, and provide guidance on counseling and case management strategies.

*Activities:* E-navigators will carry out several types of actions. They will start by developing rapport, and then educating, counseling, and problem solving as needed. They will do so through making phone calls or sending messages using participant’s preferred mobile platform to create a personal relationship to inform, influence and motivate patients. This will be supplemented with and sending twice a month automated, theory-based SMS. The E-Nav allows patients to access peer support at the time and place of their choosing through SMS or phone calls, thereby enhancing the potential “dose” especially for AYA who may have limited control over their opportunities to access care. E-navigator messaging content (to be delivered either via SMS or in conversations) will be developed using a theory-based participatory design process [[59](#_heading=h.45jfvxd)] drawing from FGD findings with AYA. Participants will undergo an initial face-to-face introduction and meeting (with counseling, if needed) at the facility at the time of enrollment at which time the e-navigator will systematically assess and document current barriers and strengths.

*Intervention schedule*: Over the first two months, the e-navigator will make at a minimum a phone twice a month, then call monthly, to check in and address issues raised and build on strengths identified during initial assessment. Communication can be more frequent if desired by patient. Face-to-face interactions are allowed on the premises of the facility but not in the community. Developmental tailoring may include focus on peer pressure/influence for younger adolescents vs. focus on intimate and personal relationships and future planning for older participants. Caseloads will target approximately 20-30 active participants. The study will reimburse participants for costs of electronic communications. Electronic contact may use any social media platform preferred by participant (e.g. WhatsApp, Snapchat, or Facebook Messenger). In addition, we will deliver SMS messages twice a month focused on health promotion, psychosocial support, and education, derived through formative design work. The SMS system uses a robotic text messaging software that allows customization of messages based on gender, individual preferences, developmental phase, language and timing [[25](#_heading=h.43ky6rz), [26](#_heading=h.2iq8gzs)]. We will use, as we have previously, user specified “code” words for HIV related activities and messages from the clinic, so that even if the messages are viewed by others, they do not reveal HIV status. SMS and e-navigator contact will be continued until either a patient misses a visit by > 14 days, has viremia > 1000 copies/ml or death for maximum of one year. After withdrawal of the intervention we will continue follow up in order to ascertain sustained impact.

*Goal*: The mechanism by which E-Nav addresses AYA barriers is primarily by reducing demotivation due to misinformation, depression and stigma (which are common among AYA). This will be accomplished through providing (1) knowledge and positive social norms to counteract negative self-perceptions and stigma, (2) a therapeutic alliance to counter isolation and loneliness and (3) “expert patient” to address practical problem solving such as how to re-register for care after missing a visit.

**Aim 2:** **Assess the effectiveness of SOC-OIC, conditional cash transfer or an in-person peer navigator to re-engage AYA after a lapse in engagement to achieve viral suppression (Stage 2).**

**Rationale:** Missed visits and poor adherence in resource-limited settings for AYA are often unavoidable. Navigation is justified as peer effects and social norms are the topic of a large area of behavioral economic and psychological research, which demonstrates that people tend to look to others like themselves when faced with uncertainty [[60](#_heading=h.2koq656), [61](#_heading=h.zu0gcz)]. Peer influences are particularly powerful among AYA during a period of formation of social engagement and emerging norms. Research suggests that peers can foster positive interactions to normalize living with HIV, model “positive living,” and provide a youth-friendly atmosphere at facilities [[31](#_heading=h.2w5ecyt), [62-65](#_heading=h.3jtnz0s)].

AYA enrolled in A4A in Aim 1 will who are at least 14 days late for a visit *or* who have unsuppressed HIV RNA as per current MOH Viral Load threshold or clinically documented non adherence prior to the first year of study enrollment or within and up through three months following the first year of study enrolment and meet other inclusion criteria for stage 2 will be randomized to either SOC-IOC, CCT, or IP-NAV.

**Standard of care outreach and intensified counseling (SOC-IOC):**

*Rationale:* When patients miss visits or have elevated viral load, standard practice per Kenyan MOH guidelines instruct clinic providers or lay health workers to actively contact patients and engage them to intensified adherence counseling.

*Content:* Intensified counseling can take the form of one-to-one counseling or a group-based education which seeks mainly to re-emphasize importance of adherence to treatment. SOC includes a limited assessment of individual circumstances and problem solving, as well as encouragement to return to the clinic. Outreach is a standardized practice within MOH facilities and it is usually attempted during the first three days after a missed visit by lay health workers. A well-established protocol for contacting patients is routine practice. Outreach workers enter the community discretely and with the principles of HIV privacy in mind. The outreach worker does not identify him or herself as being from the clinic, does not wear identifying information, and travels using typical means including public transit, motorbike or on foot. Through training and close supervision this study will ensure for standardized application of procedures.

**Conditional cash transfer (CCT):**

*Rationale:* CCT’s have empiric evidence, including from randomized trials, that show that CCTs can influence behavior in AYA. CCT’s improve adherence to glucose monitoring in AYA with diabetes in North America [[66](#_heading=h.1d96cc0)], and reduce risky sexual behavior and HIV acquisition among adolescent girls in southern Africa [[46](#_heading=h.40ew0vw), [67](#_heading=h.3x8tuzt)]. We extrapolate this evidence to engagement in HIV care.

*Activities:* A research assistant will dispense the CCT in a private office. The AYA may choose to receive the cash through their phone using m-pesa, the most widely used electronic cash transfer system in Kenya which also provides an “electronic audit trail’ for all transactions. M-pesa has been used to delivery incentives in health research in Kenya [[68](#_heading=h.2ce457m)]. In addition, elements of the action (e.g., at what age to disburse to a caregiver instead of patient? and how much?) which we will assess during FGDs and design workshop (described above), as well as with consultations with stakeholders such as the youth community advisory board (CAB) and public health authorities in Kisumu County.

*Intervention Schedule****:*** Study staff will disburse cash or mobile money to patients upon presentation on the date of scheduled visits as well as a separate disbursement for a suppressed viral load. CCT will be continued for two years following study enrolment. The amount will be guided by formative and consultation findings, however program data suggest that round trip transport to clinic costs a median of 0.71 USD (IQR: 0.35-1.18) on public transport [[25](#_heading=h.43ky6rz), [69](#_heading=h.rjefff)]. Including additional opportunity costs, we anticipate a CCT of approximately $5 total per visit and will offer a $5 addition for suppressed viral load. Our prior work suggests this size (i.e., “dose”) of incentive for missed visits is not enough to create perverse behaviors (such as purposefully missing visits) but large enough to encourage engagement.

*Target****:*** CCT’s are anticipated to act on the capability to pay for transportation (which is a challenge for many AYA who have less control over their finances, and in whom new relationships, schools, or caregivers may complicate access). CCT may also act on motivation to seek care by counteracting prioritization of more immediate needs (e.g., food, school fees, social acceptance among peers) over long term health. These mechanisms reflect literature from development economics suggesting that CCTs offer “social protection” with co-responsibilities that give patients and families the capability to prioritize things with long term benefits (e.g., healthcare and education) [[43](#_heading=h.haapch), [70-75](#_heading=h.3bj1y38)] even when facing frequent economic and livelihood uncertainty [[76-78](#_heading=h.243i4a2)]. Incentives are also thought to have particular relevance for motivation of AYA [[67](#_heading=h.3x8tuzt), [79](#_heading=h.1idq7dh)], who are still developing planning behaviors and habits.

**In-person peer navigation (IP-Nav):**

*Rationale:* Navigation is justified as peer effects and social norms are the topic of a large area of behavioral economic and psychological research, which demonstrates that people tend to look to others like themselves when faced with uncertainty [[60](#_heading=h.2koq656), [61](#_heading=h.zu0gcz)]. Peer influences are particularly powerful among AYA during a period of formation of social engagement and emerging norms. The IP-Nav has the ability to address adherence and retention barriers and promote self-efficacy and strengths in a more intensive manner than E-Nav.

*In-person peer navigator background and training:* The IP-Nav will be modeled on, but extend, existing peer support models for PLHIV in Kenya and tailored to the AYA population through a FGD and a design workshop as described above. Like E-Nav, the IP-Nav will be AYA with a secondary school education, fluency in local languages, living with HIV and matched to patients on sex and approximate age to the extent possible. The IP-Nav will undergo a similar 10-day training with additional training on home visits and basic couples and family counseling.

*Activities:* The IP-Nav will act through educating patients, psychosocial support and case management. The IP-Nav will assess retention and adherence barriers in a systematic manner and work with the participant to develop social support and clear, feasible plans to address barriers. Utilizing tools developed and specifically modified for AYA, the IP-Nav will review common issues including stigma and beliefs (thus addressing motivation) disclosure, transport, (thus addressing capabilities), as well as medication side effects, mental health and substance use, and clinic/health care worker concerns. Actions will be tailored on life stage (e.g. school-going, married, etc.), language and approach preferences of AYA. The IP-Nav may also refer the patient to additional resources, group support groups, individual counseling, or facilitate conflict resolution as needed. For participants >18 years, IP-Nav will focus on the participant and include parent/guardian, spouse/partner, and other family as needed. For children <18 years, the navigator will meet initially with participant and parent/guardian together to explain planned support and then meet with participant individually for follow-up visits. For married participants, peer navigators will be trained to carry out couple’s counseling as well as individualized sessions.

*Intervention schedule*: The IP-Nav will conduct an initial introductory visit by contacting participant at home and will conduct at a minimum monthly follow-up visits at location of preference, with more encounters as the patients desire to a ceiling of weekly. The IP-Nav will be able to use mobile phones to communicate with patients as well. They will have a case load targeting around 10-15 active participants. IP-Nav will continue until the patient is re-established into care or resuppressed for no more than two years following study enrolment.

*Target*: IP-Nav will seek to influence motivation, capability and opportunities of AYA to engage in care based on a longitudinal relationship with the client. IP-Nav has several mechanistic advantages over E-Nav. First, face-to-face contact will help grow a therapeutic relationship – which is a key for more patient-centered counseling. Also, by visiting the patient in the community or at home, the IP-Nav can develop a greater appreciation for the context of the patient’s life. Furthermore, the IP-Nav can liaise with either the caregivers, partners, or the clinic to address more complex problems, and address low motivation, while enhancing capability and opportunities (Table 1).

**Aim 3:** **Assess the effectiveness and cost effectiveness of six strategies for engagement of AYA based on combinations of two Stage 1 interventions (SOC-REC, e-navigation) with three Stage 2 interventions (SOC-OIC, conditional cash transfer, in-person peer navigation) on sustained viral suppression and engagement in care.**

*Rationale:* Prevention alone is unlikely to work for all AYA, while exclusive focus on re-engagement risks incurring preventable clinical consequences (such as drug resistance) that has particular consequences for young patients who will need effective regimens for decades.

*Content:* We therefore evaluate six joint adaptive sequential prevention and re-engagement strategies for AYA. In each of six adaptive strategies tested, the initial lower intensity prevention intervention (either SOC-REC or E-Nav) is maintained among individuals who respond well but intensified (to one of three re-engagement interventions) for individuals with early engagement failure. Such an adaptive strategy is a powerful tool to optimize efficiency and effectiveness because early non-response to any given intervention is a strong basis on which to separate AYA for whom the intervention works over the longer term from those in whom it will not work. Costs of unnecessary escalation are minimized, while additional resources are targeted to the patients and at the times most needed. By sequentially randomizing prevention and re-engagement interventions in the same patients, and following these patients prospectively for two years, we will generate rigorous, AYA-specific evidence for the comparative effectiveness and cost-effectiveness of six adaptive strategies. Even though AYA who will respond better to a particular intervention are not easily identified *a priori*, the sequentially randomized design will provide a rich data source for exploratory analyses investigating the potential for further individualization based on patient characteristics. Passive follow up beyond the 2 year study endpoint will allow evaluation of the long term impacts of the interventions after discontinuation.

*Activities:* To describe cost-effectiveness through standard micro-costing techniques to assess costs associated with each intervention strategy from a societal perspective [[80](#_heading=h.42ddq1a), [81](#_heading=h.2hio093)], we plan two-week site visits at two points in time (during early and stable implementation) to assess changes in costs associated with intervention scale-up. We will employ rigorous activity-based costing methods during site visits (key informant interviews with program managers and staff, observation of service delivery for a sample of patient visits, micro-costing, and time-and-motion (T&M) studies) to measure implementation costs of each strategy.

**Aim 4: Assess the satisfaction and provider-patient experiences with the three active strategies for engagement in HIV care (e-navigation, conditional cash transfer, and in-person peer navigation).**

*Rationale*: Understanding how the intervention approaches were received, their perceived helpfulness and influence on engagement in care, challenges encountered, and suggestions for improvement among users will provide critical feedback and insight on implementation outcomes.

*Content*: We will assess implementation outcomes and provider-patient experiences with the three active interventions: e-Nav, CCT, and IP-Nav through in-depth qualitative interviews with all navigators and a subset AYA receiving one of the three interventions. We will purposefully sample to obtain diversity in age, gender, and care engagement status.

*Activities:* We will conduct in-depth interviews with 60 AYA participants enrolled in the main trial and approximately 10 navigators (Appendix B27-B29). The 60 AYA will include interviews with 20 AYA receiving E-Nav, 20 receiving CCT, and 20 receiving IP-Nav. The interviews will be conducted after a minimum of 6 months experience with the intervention with the e-Nav interviews taking place in year 1 and the CCT and IP-Nav interviews taking place in years 2 and 3. Follow up interviews with 12-20 participants who received peer navigation will occur prior to Year 2 study end to assess perceptions of skill formation and creation of resilience as well as challenges and unintended consequences of the peer navigation intervention (Appendix B30-B32). Trained interviewers will conduct the interviews in a quiet and private location and use semi-structured interview guides focused on perception of the intervention, its effects, satisfaction, and unintended effects (Appendix B27-B32). In years 1-3, we will also interview peer navigators (approximately 8-10) to understand their perceptions of how the e-navigation and in-person navigation interventions were received and explore how they perceived it may have influenced participants as well as potential shortcomings. In-depth interviews among navigators will also use semi-structured interview guides (Appendix B27-B32). Interviews with AYA and navigators will each take about 45-60 minutes. The interviews will be audio recorded and recordings marked only with date and a participant number. They will receive compensation for the time and travel, about Ksh 500.

Focus groups will be held with clinic staff at study sites and separately with the research team involved in supervising and implementing the peer navigators. An external qualitative research assistant will conduct the focus groups which will be audio-recorded and last approximately 90 minutes (Appendix B30-B32).

**Aim 5:** **Determine impact of interventions on care transitions among adolescents and postpartum women with HIV.**

The subset of participants who experience transition during study follow up will be identified using clinic registers and study data. We will carry out a secondary analysis of already collected study data without additional data collection.

- - 1. **WITHDRAWALS**

Participants will be free to withdraw from the study at any time by presenting to the clinic and indicating their desire to withdraw, or by sending an SMS with the word ‘STOP’. Reasons for study withdrawal will be ascertained either at the study clinic or by telephone. Where possible, information on study endpoints will be evaluated at the time of study withdrawal. No further study related messages will be sent, or phone calls made, to those who withdraw.

## STUDY MEASURES

Study measures will include formative FGDs, clinical care from routine records supplemented by a battery of standardized and adapted surveys, costing metrics, process data, and in-depth interviews on user experience. The measures are listed below in Table 1 and further detailed after the table by formative work and aim.

| **Table 1: A4A Study Measures and Timepoints** | | | | | | | | |
| --- | --- | --- | --- | --- | --- | --- | --- | --- |
| **Variable(s)** | **Data Source** | **Scre-ening** | **Enroll** | **Lapse^1^** | **Y1^2^** | | **Y2^2^** | |
| Disclosure | Disclosure screening script | X |  |  |  | |  | |
| Eligibility | Eligibility screening form | X |  |  |  | |  | |
| Locator | Locator form |  | X | X | X | | X | |
| Demographic | EMR/routine clinical form |  | X |  |  | |  | |
| Clinical | EMR/routine clinical form | *At routine follow-up* | | | | | | |
| Viral load | EMR/routine lab request form/Extended outcome ascertainment | *At routine follow-up* | | | | | | |
| Vital Status | EMR/routine discontinuation form  LTFU Form/Outcome Ascertainment Form  Cause of death form (verbal autopsy) |  |  | X | X | X | | |
| Transfer Status | OpenMRS EMR/routine discontinuation form  LTFU Form/Outcome Ascertainment Form |  |  | X | X | X | | |
| Socio Demo | Socio Demographic form |  | X |  |  | |  | |
| Food In-Security | Individually Focused Food Insecurity Access Scale  (IFIAS) |  | X | X | X | | X | |
| General Health, Social Support | General Health Assessment for Children (GHAC) Quality of Life Questionnaire – Adolescent |  |  |  |  | |  | |
|  | Changes in last 12 months |  | X | X | X | | X | |
|  | General Health |  | X | X | X | | X | |
|  | Infectious status |  | X |  |  | |  | |
|  | Barriers and preferences |  | X |  |  | |  | |
|  | Psychological well-being |  | X | X | X | | X | |
| Adherence | Comprehensive ART Adherence Measurement for Paediatrics (CAMP) |  | X | X | X | | X | |
| Hospitalization status | Enhanced Retrospective Review – Hospital use (ERR-hosp.) |  | X | X | X | | X | |
| Pregnancy status | Enhanced Retrospective Review – Pregnancy (ERR-Preg.) |  |  | X | X | | X | |
| Depression | Patient Health Questionnaire-9 (PHQ-9) [[8](#_heading=h.wnyagw)2, [8](#_heading=h.3gnlt4p)3] |  | X | X | X | | X | |
| Anxiety | General Anxiety Disorder (GAD)-7 (Spitzer) |  | X | X | X | | X | |
| Alcohol and drug use | Alcohol Use Disorders Identification Test (AUDIT 10+) [[84](#_heading=h.1vsw3ci), [85](#_heading=h.4fsjm0b)] and drug use adapted from KIAS |  | X | X | X | | X | |
| Trauma | Adverse Childhood Experiences (ACES-IQ) |  | X |  |  | |  | |
| e-NAV form | Form to document e-NAV delivery |  | *Completed during each e-NAV session in follow up* | | | | | |
| IP-NAV form | Form to document IP-NAV delivery |  | *Completed during each IP-NAV session in follow up* | | | | | |
| Care barriers | Semi-structured questionnaire - Tracing form [[49](#_heading=h.3ep43zb)] |  |  | X | *At routine follow up if VL due and LTFU* | | | |
| Withdrawal | Study Withdrawal form |  | *If needed, during follow up* | | | | | |
| Intervention experience | Intervention satisfaction and experiences questionnaire (includes other study involvement) |  |  | X | X | | X | |
| Intervention satisfaction | In-depth Interview Guides |  |  |  | X | | X | |
| Intervention tailoring | Focus Group Discussion Guides^3^ | *Prior to study implementation* | | | | | | |
| Process data and fidelity of intervention implementation | Fraction of intended CCT actually disbursed, number and nature of communications between patients and navigators | *Throughout follow-up* | | | | | | |
| Direct and indirect costs | Participant survey, micro-costing worksheets, administrative records and time-and-motion studies | *Measured at sample of times* | | | | | | |
| First appointment missed by > 14 days in year 1 and at routine follow up if VL due and LTFU^2^  Carried out either in clinic, or via community-based tracking for lost to follow-up; ^3^ 60 AYA and 10 peer navigators (the same individual may act as E-Nav or IP-Nav during study period) in years 1-3. | | | | | | | |  |

*Formative work:*

Formative measures to tailor the interventions to the local context will include FGDs and a DCE. FGD Semi- guides (Appendix B24-B26) will be solicit conceptual preferences on intervention design for example, selection of SMS wording, language and tone preferences, prioritization of anticipated social support needs (e.g. school, life transitions, partnerships, clinic access), counseling topics. The FGDs findings will then be used in a design workshop which will translate preferences into intervention activities. The DCE will specifically inform CCT intervention preferences.

**Aim 1: Assess the effectiveness of standard of care routine education and counseling vs. electronic navigation for preventing lapses in engagement among 880 AYA living with HIV in Kenya (Stage 1).**

Patient measurements (Table 1) will allow examination of response heterogeneity to the primary outcome (through subgroup analyses) as well as enhance statistical efficiency (through secondary analyses to adjust analyses for baseline covariates). We will collect socio-demographic (e.g., age, sex) and clinical (e.g., date of enrollment, WHO stage, ART initiation date, pharmacy refill, visit dates, viral load, weight and height for BMI) captured routinely on MOH-issued clinical care forms (eg. MOH 257 Greencard). Data will be in medical charts including clinical electronic medical record system (eg. Open Medical Records System [OpenMRS] http://openmrs.org or Kenya Electronic Medical Record [KenyaEMR]) already functioning at sites.

In addition to routine collected clinical data we will use a battery of standardized measures commonly used in sub-Saharan Africa. Research Assistants will administer the surveys capturing additional socio-demographic information (eg. family life living situation, school, income, drinking water access, sexual history and risk) (Appendix B8); general health (Life events; social support; physical, emotional, and social well being, health care utilization; symptoms) (Appendix B9); food insecurity (Appendix B11); behavior factors (eg. treatment adherence, alcohol and drug use) (Appendices 12, 19); mental health (depression, anxiety, trauma) (Appendices B14-17), and provider satisfaction (B13). Relevant clinical factors not fully captured routinely will be collected. An enhanced retrospective review for recent pregnancy information and a LTFU form for tracing and reasons for missed clinic visits will be used (Appendices B10, B19).

For patients who miss their visits, we will seek to trace and collect HIV viral load in the field which our research group has experience with [[86](#_heading=h.2uxtw84)]. In addition, implementation fidelity will be assessed using patient surveys on number, nature, setting, and outcome of e-navigation contacts at re-randomization, year 1 and year 2 data collection. Automated SMS system documentation of messages sent will also be captured. See Table 1 for a list of study measures by study timepoint.

**Aim 2:** **Assess the effectiveness of SOC-OIC, conditional cash transfer or an in-person peer navigator to re-engage AYA after a lapse in engagement to achieve viral suppression (Stage 2).**

At the start of Stage 2 (when patients are re-randomized to three re-engagement interventions), reasons for non-return and virologic failure will be solicited after tracing. We will use the same surveys indicated in aim 1 capturing socio-demographic information, general health, food insecurity, behavior factors, mental health, provider satisfaction, and additional clinical information. For the outcome, we will examine viral suppression six months after re-randomization (which can happen anytime from six months after enrollment for those who fail first stage intervention immediately to 1.5 years after enrollment if they fail on the last day of year one). Operationally we will find a window of 90 days six-months after re-randomization and assess results of viral load and appointment nearest to that date. We will consider a patient as re-engaged only if both that viral load measurement is suppressed and that visit was made. Present programmatic procedures require a drug pick up or clinic visit every 90 days (including in community-based models of care) so all patients should have an appointment in the 90-day window we examine. We estimate that 50% of patients will be eligible for routine viral load monitoring in that window, and the research team may need to work closely with the clinical team to obtain remaining assays or supplement additional viral load measurements. In addition, implementation outcomes such as fidelity, user experiences, and other study involvement will be obtained through patient questionnaire on number, nature, setting, experience and outcome of each active intervention e-navigation at re-randomization, year 1 and year 2 timepoints (Appendix B21). We will use structured measurements at the time of each visit for those in the CCT arm to verify previous receipt and check for any ensuing harms and document PAC dose delivery.

**Care Status**: At years 1 and 2, and at time of first lapse in retention if any, care status will be ascertained and documented. The LTFU form (Appendix 19.1) is used during tracing and the Outcome Ascertainment form is used at year 1 and year 2 (Appendix 19.2). If the patient is not reached, a report by family member, neighbor or other close informant will be obtained. If transferred or died the information will then be documented in routine Discontinuation form (Appendix B6). If a death, a verbal autopsy will be conducted to obtain details surrounding the adolescent’s death (Appendix B7).

**Viral Load Status**: At 6 months post rerandomization, years 1 and 2, viral load status will be reviewed for completion. For participants with missing viral loads, an Extended Outcome form (Appendix 28) will be used to investigate, locate and document, or classify through an adjudicated decision-making process for viral loads not located. The investigation will involve a desk review of medical records at the 6 months post rerandomization and year 1 timepoints and both a desk review and participant tracing at the year 2 timepoint. The review will involve determining patient status and if alive, HIV care and study status (eg. active in the care/study, transferred, withdrew from study, out of care) and a deeper dive to examine if a viral load was ordered, not ordered during the window period, reasons, and health and clinic visit status to ultimately make an adjudicated decision of if a true missing and unknown.

**Aim 3:** **Assess the effectiveness and cost effectiveness of six strategies for engagement of AYA based on combinations of two Stage 1 interventions (SOC-REC, e-navigation) with three Stage 2 interventions (SOC-OIC, conditional cash transfer, in-person peer navigation) on sustained viral suppression and engagement in care.**

Cost effectiveness analysis requires assessment of expenditure records, discussion with program managers and staff, care delivery logs, and observation of service delivery with T&M methods to assess transportation costs and expenditures for additional health services received (Appendices B22-23). We will capture direct costs incurred prior to implementation (e.g., selection of navigators); direct costs of implementation (e.g., navigator time); and indirect costs of administration. We will place costs into standard expenditure categories—personnel, recurring goods and services (e.g. supplies), and fixed costs (e.g. equipment). We will assess “economic” costs (the true value of resources consumed) through identifying the value of subsidized or donated resources from purchasers and donors, and as needed, three price quotes from appropriate market sources. Personnel costs will be estimated as the sum of the product of resources (e.g. staff minutes) and times unit costs (e.g. compensation levels). We will use standard micro-costing techniques [[87](#_heading=h.1a346fx)] to measure the cost of each component of interventions, such as counselor time and SMS system set-up and maintenance.

**Aim 4: Assess the satisfaction and provider-patient experiences with the three active strategies for engagement in HIV care (e-navigation, conditional cash transfer, and in-person peer navigation).**

In addition to primary study outcomes, we also measure implementation outcomes through in-depth interviews to understand AYA preferences, mediators and how the interventions succeed or fail. Semi-structured interview guides developed based on the COM-B framework, a model focused on designing interventions aimed behavior change, will include (1) perceptions of the intervention, (2) perceived effects of intervention, (3) satisfaction and (4) unintended effects (positive or negative) (Appendix B27-B32). The interviews will be carried out in years 1-3 of the study with AYA receiving e-Nav, CCT, or IP-Nav and all navigators (Appendix B27-B32). Followed by interviews of a sample of participants who received peer navigation and focus groups with clinic and research staff who oversee peer navigators (Appendix B30-B32).

**Aim 5: Determine impact of interventions on care transitions among adolescents.**

Successful transition will be defined as retained in care and virally suppressed 6 months after transition. Transition will be assessed using clinic registers and file location to determine where the participant is receiving HIV care and if transition has occurred, when it happened. We will assess the primary outcome via study data to identify any missed visits > 14 days and all viral loads after transitioning up to one-year post-transition. Participants who discontinue care will be characterized as transfer out if documented transfer in chart, lost to follow up if no visit within 6 months, dead if document or unknown status.

# ETHICAL CONSIDERATIONS

## ETHICAL APPROVAL

Ethical approval for this study will be obtained from all relevant institutional review boards before initiating any of the study data collection activities. In the US, Washington University in St. Louis and University of Colorado Denver (UCD) Institutional Review Boards will review this protocol. In Kenya, the protocol will be reviewed by Kenya Medical Research Institute’s (KEMRI) Scientific & Ethics Review Unit (SERU) (FWA# 00002066). We will ensure that all procedures conform to US, Kenyan, and international ethical standards regarding research involving human subjects.

## HUMAN SUBJECT INVOLVEMENT AND CHARACTERISTICS

In this clinical trial we will follow approximately 1114 adolescents and young adults (AYA) living with HIV ages 14-24 years, over a 24-month period at up to six high-volume MOH HIV care and treatment facilities in Kisumu County, Kenya. At each facility, eligible and consenting AYA will be randomized to either receive standard of care (SOC) based existing Kenya national HIV guidelines or e-navigation intervention which consists of weekly automated health promotion SMS messages and personal contact over the participant’s electronic platform of choice (e.g. phone, WhatsApp®, Snapchat®). In order to not exclude AYA who may not have regular phone access due to lack of a phone, inability to access a phone during school (e.g. boarding school) etc. we will allow AYA without phone access to enroll to the study but will allocate them to SOC-REC in stage one as e-Navigation can not be delivered. If an enrolled AYA (including those without phone access) misses a clinic visit by 14 or more days or has viral failure defined as viral load as per current MOH guidelines or has clinically documented non-adherence, he/she will be traced and re-randomized 1:1:1 to a higher intensity intervention to receive either SOC, conditional cash transfer, or in-person peer navigator which will consist of in-person sessions with the AYA at their location of preference. At enrollment, re-randomization, and 12-months and 24 months following enrollment, AYA will participant in brief study surveys on social support, socio-economics, alcohol use, mental health and well-being. Routine clinic data will also be collected from their medical records including viral load.

At the outset of the study, before the interventions are actually implemented, we propose formative work to understand patient preferences in order to shape the interventions to meet the desires of the target AYA population. The human subject component will involve, during year 1, three FGDs with approximately eight participants each for a total of 24 people. Each of the three FGDs will focus on one of our active interventions: e-navigation, conditional cash transfer, and in-person navigation. Focus group participants will be purposively sampled from the participating clinics striving for age and gender balance. Each FGD will be aimed at gathering AYA insights, opinions, and preferences for a particular intervention. Standard facilitation approaches as well as virtual media tools will be used, including art-based techniques, and attention will be paid to soliciting input from younger individuals. Findings from the FGDs will then be translated into content that will be customized for AYA in the Kisumu region during a design workshop which will be attended by approximately eight peer navigators (i.e., providers) as well as approximately 12 AYA. We use established interactive and interactive design approaches to design the content, nature, and user experience of the three active interventions (CCT, in-person navigation and electronic navigation) to optimize uptake and effects.

We also plan to conduct 60 individual interviews to assess participant experiences with each of the intervention assignments. A subset of AYA participants randomized to e-navigation, in-person navigation, or conditional cash transfer will take part in the interviews; 20 interviews will be conducted per intervention arm in years 1-3. Interview participants will be purposively sampled for age, gender, and level of engagement in care. These interviews will assess patient perceptions of the effects of the intervention on their capabilities, motivation and opportunities to engage in care as well as the acceptability and appropriateness of their interactions. Unexpected effects will be solicited. Lastly, also in years 1-3, we will interview all peer navigators (approximately 8-10) to understand their perceptions of how the e-navigation and in-person navigation interventions was received and explore how they perceived it may have influenced participants as well as potential shortcomings.

Inclusion criteria for both the qualitative work as well as trial itself are: HIV-infection, on or initiating ART, 14-24 years of age, living > 6 months in Kisumu County in previous year, capable of informed consent (> 18 years) or with a legal caregiver available for consent (14-<18 years), ability to read or be read short message service (SMS) messages, willingness to be contacted by clinic upon missed appointment, and for AYA who report phone sharing must have disclosed to the person sharing the phone. Additionally, we will include AYA who are aware of their HIV status or whose caregivers agree to assisted disclosure. Consent for home visits is routinely sought for clinical purposes and <1% of patients refuse. Exclusion criteria are: AYA who participated in ADAPT-R, and those planning to move out of Kisumu County, those acutely ill and requiring hospitalization, and those who report sharing phones but have not disclosed to the person sharing the phone.

## PROTECTING PRIVACY AND CONFIDENTIALITY

*Data collection and storage:* For virtual formative data collection, we will use secure platforms that ensure privacy. Health information will be protected by coding all information required for the study and keeping the coded data separate from clinic records. Electronic study databases will not contain participant names. The list of coded and uncoded information (linkage sheet) will also be kept separately. This linkage sheet will be maintained for a maximum of five years, after which all links between coded and uncoded data will be broken. All databases will be password protected and accessible only to authorized study staff. Similarly, all computers containing study data will be password protected and only accessible to authorized staff. Clinical data extracted from the electronic medical record system will be coded with a participant study ID and that data will be automatically uploaded to a secure server in Kisumu. The codes that link the name of the participant and the study ID will be kept confidential by in a secured database accessible only by the study investigators. The computer containing the automated SMS software and databases will be locked in a secure room with access restricted only to authorized personnel. Because phone numbers to which messages are sent are essentially personal identifiers, the computer on which these phone numbers will be stored will be accessible only to specifically designated study staff. Every effort will be made to safeguard subjects from future contact. For behavioral or other data collected as part of the study, participants will be assigned a unique identification number and information will entered into the electronic web-based data application (eg. Open Data Kit [ODK]) on hand held devices with secure sign in screens. This data will be housed on the secure server in Kisumu. The hand held devices will be stored in a secure place only accessible by study staff. All paper files will be stored in locked file cabinets and electronic files will be stored in password-protected files.

*Staff training:* Staff members who will carry out study procedures will undergo training on research ethics and Good Clinical Practice (GCP) through the online Collaborative Institutional Training Initiative (CITI) prior to initiation of the trial. Certificates of successful completion of GCP training will be kept in the trial master file on site.

## REFERRAL FOR ACUTE HEALTH CARE

When meeting with AYA, and their caregivers if applicable, or when looking for patients as a part of outreach it is possible that study staff will encounter someone who is acutely ill and in need of immediate medical attention. In this case the staff will notify the clinic and the clinic will initiate a response to bring the patients to care. The clinics in which the study is planned have inpatient wards and access to an ambulance for transport of patients who are acutely ill and unable to reach clinic. The study stuff will have access to the clinical staff at all times via phone and we'll be able to make referrals at patient request.

## POTENTIAL RISKS TO STUDY SUBJECTS

Potential risks to participants in this study have been carefully considered and minimized as outlined below.

*Social and privacy risks:* These may occur if information about participants or their family members, specifically HIV status, or other personal details were to be disclosed outside of the research setting. In particular, AYA might face serious social risks (disruption of family, discrimination, and/or physical harm) if their HIV status were to be disclosed without their consent or assent. These risks could occur if clinic or research staff do not maintain confidentiality or secure access and records per clinic and study procedures. It could also occur during outreach (tracing), which is also part of routine care, to patients who experience an initial lapse of retention. Additionally, this could also occur during virtual meetings and interviews during the formative part of this study. There is also risk of privacy loss during SMS messaging and navigation communication, especially if adolescent is using a shared phone and messages are viewed by others.

*HIV self-disclosure and stigma:* There is the potential that younger adolescents may not fully understand their HIV status or how they acquired HIV; caregivers are commonly fearful of potential negative consequences of HIV disclosure to their child. They fear that the child may not react well, may tell others about their HIV status, and may experience HIV-related stigma and discrimination, or ask questions about how they got HIV. Additionally, AYA may experience stress or discomfort during administration of surveys or as part of discussion of difficult topics with peer navigators.

*Viral load testing*: Viral load testing is not expected to pose significant additional risks to study participants outside of the risks they are exposed to regularly as part of routine patient monitoring as part of care in Kenya.

*Cash transfers:* We will offer nominal cash transfers for patients randomized to this study arm. We have designed the cash transfer based on our prior experience delivering over 1,000 cash vouchers to approximately 300 patients who are aged 18-24 years in this setting in Kenya (NCT02338739) without problems. We do not believe the amounts given are coercive based on consultation with in-country partners and prior ethical review. Spouses, family members, or household members may also benefit from the cash transfers and therefore may put pressure on participants to continue in HIV care in order to continue to receive money or may appropriate the cash for themselves especially among younger adolescents and young women in marriages. We will actively solicit these adverse events during encounters with patients through a standardized survey instrument used in the CCT arm as well as any reported adverse events detected by study staff who will be regularly interacting with participants. In our current ADAPT-R study, which includes qualitative interviews about patient experiences with the CCT, participants have not reported any coercive consequences due to funds during qualitative discussions with conditional cash transfer study arm participants or through routine adverse event reporting. Further, research in similar settings reveals that behavioral incentives including cash, nutritional support, water and hygiene supplies etc., are often shared within households promoting both the intended behavior and supporting family health overall. Furthermore, our Youth Community Advisory Board will be requested to report any unintended consequences or concerns that are noted in the community. There is also a potential risk of theft if someone sees a participant receive cash at the facility, however cash will be dispensed in private study office and participants will have the option of receiving funds via m-pesa (electronically) if that modality is preferred.

We will also be assessing the cost effectiveness of this study; this involves micro-costing expenses and personnel time related to intervention set up and delivery. Due to the nature of the proposed costing activities (e.g., review of non-patient administrative data (e.g., procurement data, documentation of health care worker activities), we do not believe there are associated potential risks involved.

***Risks related to employment:*** Current clinic and MOH staff and research staff will be recruited to participate in a focus group discussion. Staff may feel that participation is mandatory or would effect employment status or performance evaluation. They may also not feel free to share their honest opinions for the same reason.

Our study has carefully considered these potential risks and have taken necessary precautions to minimize them as outlined below. We plan to conduct training on maintaining patient privacy and confidentiality for research team and relevant clinic staff. Although patients do consent to outreach as part of routine care when they enroll in care, we will consent/assent again for this at study enrollment and we will ensure that research staff conducting outreach are highly skilled at conducting discrete and confidential patient outreach. Our data collection and storage procedures are designed to ensure confidentiality of all patient data and to protect privacy through SMS. We will ensure that e-navigation communication and navigator correspondence do not contain any information which might identify a patient as HIV infected. Building on our current study’s work, we will ensure patient messaging uses code words to help prevent accidental disclosure of HIV status if others were to see messages. To further prevent any inadvertent disclosure, we will assess children and adolescents’ HIV disclosure status, phone sharing, and understanding of HIV status prior to any description of the study or consent/assent procedures using a standardized operating procedure. Our study team has extensive experience providing peer support and counseling on difficult topics associated with living with HIV in this setting. Our AYA peer navigators will receive training on counseling and providing social support as well as management of mental health crisis (e.g. referral to health care provider) and have supervision and mentorship from experienced adult peer navigators. We will institute rigorous monitoring to assess for any harms with conditional cash transfer receipt and allow for electronic receipt of funds for participants in that arm. For individuals and families experiencing problems that cannot be managed within the clinic, we will establish referral mechanisms.

## ADEQUATE PROTECTION AGAINST RISKS

Our study has carefully considered these potential risks and have taken necessary precautions to minimize them. We will conduct training on maintaining patient privacy and confidentiality for research team and relevant clinic staff. Although patients do consent to outreach as part of routine care when they enroll in care, we will consent/assent again for this at study enrollment and we will ensure that research staff conducting outreach are highly skilled at conducting discrete and confidential patient outreach. Our data collection and storage procedures are designed to ensure confidentiality of all patient data and to protect privacy through SMS. We will ensure that e-navigation communication and navigator correspondence do not contain any information which might identify a patient as HIV infected. Building on our current study’s work, we will ensure patient messaging uses code words to help prevent accidental disclosure of HIV status if others were to see messages. To further prevent any inadvertent disclosure, we will assess children and adolescents’ HIV disclosure status, phone sharing, and understanding of HIV status prior to any description of the study or consent/assent procedures using a standardized operating procedure. Our study team has extensive experience providing peer support and counseling on difficult topics associated with living with HIV in this setting. Our AYA peer navigators will receive training on counseling and providing social support as well as management of mental health crisis (e.g. referral to health care provider) and have supervision and mentorship from experienced adult peer navigators. We will institute rigorous monitoring to assess for any harms with conditional cash transfer receipt and allow for electronic receipt of funds for participants in that arm. For individuals and families experiencing problems that cannot be managed within the clinic, we will establish referral mechanisms.

All participants will provide either assent (14- <18 year olds) or consent > 18 year olds. As part of the assent/consent process, we will meet privately with each potential participant to ensure potential participants are aware it is there decision to participate or not participate in research and they should not feel pressure from clinic staff, spouse, family or others to participate. They will be informed that they will continue to receive regular HIV care regardless of study participation. Clinic staff will not directly benefit by having patients participate in the study and therefore are unlikely to unduly pressure research participation. Ethical research practice during recruitment, enrollment, and study activities will be reviewed as part of clinic education conducted prior to study start.

***Protection against risks related to employment:*** Participation will be voluntary. Recruitment and FGD will be conducted by an external qualitative research assistant not involved in the regular Tatua study activities. PI directly supervising research staff will not be aware of who participates. Data will be de-identified.

## DATA SAFETY AND MONITORING PLAN

This is a low risk study, with behavioral social interventions to enhance retention in HIV care using an adaptive approach, thus a formal Data Safety Monitoring Board is not required, and no formal stopping rules are planned. However, the Multiple Principal Investigators (MPIs) will be responsible for executing the Data Safety and Monitoring (DSM) plan, conforming to rigorous standard monitoring procedures for patient safety, and complying with reporting requirements. If any increased risks are identified, the MPIs will review and determine the adequacy of human subject protections, making recommendations for enhancing these protections if deemed necessary. The MPIs have primary responsibility for the overall conduct of the study, including the safety of human subjects. The MPIs will ensure appropriate (1) conduct of the informed consent process (e.g. that informed consent is obtained before proceeding with study procedures); (2) enrollment of study subjects; (3) collection and analysis of data; (4) implementation of study procedures to ensure consistent monitoring of subjects for possible adverse events; (5) immediate receipt and review of standardized and detailed adverse reports and reporting of to the Institutional Review Boards; and (6) maintenance of the privacy and confidentiality of study subjects.

The MPIs maintain ultimate responsibility for the project and for the safety of study participants. Details of potential risks and measures in place to protect participants can be found under Human Subject Protections documents. The MPIs will be in contact with the research team on a regular basis to review the progress of the study and address any human subject issues that occur. Adverse events and unanticipated problems will be reported by study staff to the MPIs and then to respective IRBs per the individual institutional guidelines. These discussions may involve adverse event prevention measures, recruiting of appropriate study subjects, research staff training on protection of human subjects, as well as occurrence of adverse events, unexpected incidents, or protocol problems. For more details on the MPIs’ roles and responsibilities please see the MPI plan. This is a low risk study, with behavioral social interventions to enhance retention in HIV care using an adaptive approach, thus a formal Data Safety Monitoring Board is not required, and no formal stopping rules are planned.

For data safety, clinical data extracted from the electronic medical record system will be coded with a participant study ID to maximize confidentiality. Survey data, collected at the time of enrollment, time of first treatment lapse, at 12- and 24-month follow up, using standardized computer questionnaires, will be entered into password protected and encrypted mobile phone devices and transmitted to a secure Aggregate server and synchronized to a secure local database each night in Kisumu. If data systems are not working, paper questionnaires will be used and the data entered into the devices once available. Paper surveys will be stored locked cabinet and locked room only accessible by research staff. The codes that link the name of the participant and the study ID will be kept confidential in a secured database accessible only by the study investigators and limited research staff. For the qualitative data, study IDs will be used rather than names on qualitative written records and audio recordings. The audio-recordings will be destroyed immediately after the transcript is produced. All paper files will be stored in locked cabinets. Clinical and qualitative databases will be maintained in a secure Windows-based operating system behind a secure firewall with password protection and the latest encryption technology and backed up daily (24G NT Backup). Laptops and handheld devices will be encrypted, password protected, backed up daily, and checked in and out of a locked room each day.

All serious adverse events associated with the procedures of this study will be reported within the specified time-frame to the appropriate Institutional Review Boards. Field staff will be trained to complete descriptions of adverse events that will then be communicated to the onsite Study Coordinator immediately, and then sent electronically to both the U.S. multiple-PI’s - and the Kenyan co- Investigators within 24 hours. Adverse event reporting will include social and privacy adverse events that may result as part of study interventions. Additionally, we will engage a Youth Community Advisory Board (Youth CAB) for the study made up of AYA living with HIV in the study community but not participating themselves, such as members of youth HIV advocacy groups. The Youth CAB will meet twice annually and as part of their terms of reference, will discuss and report unintended harms or consequences of study interventions to the research team.

## POTENTIAL BENEFITS OF PROPOSED RESEARCH

This may be a direct and significant benefit to the study participants as AYA who are at much higher risk of adherence and retention gaps and subsequently increased risk of morbidity and mortality. In general interventions offered such as SMS messages, peer support, and cash transfers increase connection to HIV care and therefore improve the overall health outcomes of the patients. Participating in this trial, therefore, may avail AYA of activities and supports which are helpful to their health. In addition to these direct benefits to study participants, the data collected from this study will produce generalizable knowledge that is likely to benefit this patient population in the long-term. Improving engagement and retention in care for AYA, as well as increasing viral suppression, are key global targets for HIV care.

- 1. **IMPORTANCE OF KNOWLEDGE TO BE GAINED**

Importance of the minimal risk in this study is outweighed by the importance of knowledge to be gained. The global response to HIV is based on engaging persons with HIV in care and keeping them in care once they have established care. Public health must prioritize science to engage AYA because this group is particularly vulnerable and epidemiological studies show they are experiencing worse clinical outcomes, including mortality, when compared to either adults or younger children. Therefore, our research objective to assemble promising interventions into strategies that can have the most effect at the population level is a critical mission of public health. If we can use these interventions optimally and leverage from the experience gained in our existing study to better engage AYA we may be able to substantially improve health outcomes for AYA and prevent mortality.

- 1. **INCLUSION OF WOMEN AND MINORITIES**

This study does not use gender as an enrollment criterion, nor does it use minority status as an enrollment criterion. The study will be conducted exclusively within western Kenya and all participants will be Kenyans or full-time residents of Kenya, but there is no further distinction made in terms of ethnicity. The clinics from which prospective study participants will be enrolled in Kenya serve an ethnically diverse Kenyan population.

- 1. **INCLUSION OF PREGNANT WOMEN**

Young pregnant women are a critical population affected by HIV and important to involve in a study of retention both in order to maximize their own health and also for prevention of mother to child transmission. No biological interventions are proposed and therefore there is no inherent “risk” to the fetus in participation.

- 1. **INCLUSION OF CHILDREN**

This research is focused on adolescents (ages 14-19) and young adults (ages 20-24) (AYA) living with HIV. There are an estimated 1.8 million adolescents living with HIV globally, 80% of whom live in sub-Saharan Africa. Kenya, Tanzania, and Uganda are among the six countries that are home to half of the world’s adolescents living with HIV. Until quite recently, adolescents have been the only age group with increasing HIV mortality, at a time of massive scale-up of treatment programs, when mortality is declining in all other age groups. The last national survey of viral suppression in Kenya (KAIS) found that adolescents and young adults on treatment were 15% more likely to have virologic failure as compared to adults (70% vs. 85%). Retention in care is vital to achieving longstanding viral suppression, improved clinical outcomes, reduce mortality, and to preventing onward transmission of HIV. This adaptive study will contribute valuable science and approaches to reduce the burden of these outcomes. The Multiple Principal Investigators (MPIs) have significant experience working clinically with children and adolescents and conducting studies within the pediatric population in Kenya. Dr. Lisa Abuogi is a clinically trained pediatrician who specializes in child and teen health. She has over 10 years of experience working in HIV care with pediatrics and adolescents within the FACES program in Kenya, is a collaborator in several international pediatric HIV research networks and has served on the Kenya National Working Group for Pediatric and Adolescent HIV. Dr. Elvin Geng specializes in engagement in HIV care in Africa and has served on the Scientific Advisory Board for the Pediatric Adolescent Treatment Africa – a non-governmental organization that leads international efforts to advance HIV care and treatment for adolescents. In addition, the study staff and research assistants including the adolescent peer navigators working for the study work will receive specific training on recruitment, enrollment, and engagement with AYA patients. We note the age of majority in Kenya is 18 years and thus some participants will be below this age and require consent of a legal guardian as well as assent. We recognize that extra steps are needed to ensure human subjects’ protections when conducting research with children and have explained these in detail in the Human Subjects Protection section.

# DATA MANAGEMENT

## DATA COLLECTION AND STORAGE

Study data will be maintained on a secure study server at the ADAPT-UHAI office in Kisumu. We will use existing data collection currently in place at study clinics including MOH routinely captured clinic and demographic information. Additional psychosocial, general health, and socioeconomic survey data will be collected using an electronic web-based portal (eg. ODK) with data securely stored on the study server. Qualitative data will also be obtained. Each process is described below.

*Routine clinical data:* Data extracted from the clinical electronic medical record system will be stored securely on encrypted study server in Kisumu. Key data to identify participants who need to be traced based on missing a visit by 14 days will be flagged and securely pushed to encrypted mobile handheld devices in Kenya each day to facilitate patient tracing. The Kenyan study server will be kept secure and may only be accessed through passwords that are available only to study investigators and study data managers.

*Study-specific survey instruments:* These measurements will be made using instruments administered to AYA through handheld mobile devices using a web-based secure data portal (eg. ODK or RedCAP) which will house the data on the secure study server in Kisumu. The server is maintained with secure connections and accessible only through passwords to investigators and data management staff. The handheld devices will be password protected and stored securely where they are only accessible to study staff. These measures will be collected at enrollment, time of re-randomization, end of year 1, and at end of year 2 by contacting patients when they come to the clinic for regularly scheduled visits or by contacting the patient at home.

*Qualitative:* Data will come from focus group discussions and individual interviews. Focus group discussions and individual interviews will be audio-recorded. Data will also include field notes documented by a focus group note-taker or the interviewer to capture the tone of the discussion, any non-verbal communications, and if a focus group the composition of the group. Audio-recordings will be transcribed and transcripts stored securely.

*Data Back Up:* Electronic data including all study databases and supporting electronic documentation will be backed up daily on a secure cloud-based server. On a monthly basis, a complete backup tape will be transported off-site to the KEMRI offices for rotating secure storage.

## STATISTICAL ANALYSIS

**Analysis for Formative work:**

Audio-recorded FGDs will be transcribed, coded and processed to derive themes. Dr. Kwena will debrief regularly with the team as these data are being collected and review the transcripts [[88](#_heading=h.3u2rp3q)]. Transcripts of recordings will be coded by qualitative assistants using content analysis, facilitating open coding (inductive approach), a deductive approach that uses the categories using the COM-B framework to advance conceptualization [[89](#_heading=h.2981zbj)] and then develop major themes to understand how interventions worked (or did not work).

**Analysis for Aim 1:**

*Primary analysis:* Among the Aim 1 population, our primary outcome for Aim 1 is defined as care engagement failure, i.e., experiencing any of the following events at follow-up: lapse in retention (defined as 14 days late for a scheduled visit) within the first year of study enrolment *or* viral failure (defined as a viral load >1000 copies/ml or current MOH viral load threshold) prior to the first year of study enrollment or within and up through three months following the first year of study enrollment *or* death. Patients will be censored if they withdraw or if they transfer to another clinic. We will use targeted maximum likelihood for estimation (TMLE); in particular, adjustment for covariates can be done to increase the precision of effect estimates and control for potentially informative censoring due to migration, transfer, and withdrawal.

*Secondary analyses:* Additional analysis will use a Kaplan Meier and log rank test to compare time to first lapse between arms, examine alternative outcome definitions such as retention alone (mean visit adherence, visit constancy [[90](#_heading=h.odc9jc)]), medication possession ratio [[91](#_heading=h.38czs75)], and HIV RNA levels. Prespecified subgroup analyses (age 14-19 vs 20-24, sex, site, CD4 level) will be carried out to assess whether differences in the effect of adaptive strategies across these groups.

**Analysis for Aim 2:**

*Primary analysis:* Among the Aim 2 population, the primary outcome is viral suppression six months after re-randomization to one of three Stage 2 interventions. As in Aim 1, we will use TMLE to compare the proportion suppressed in the SOC-OIC, CCT and IP-Nav arms.

*Secondary analysis:* An alternative secondary outcome is a composite of time to return for the subset of patients failing Stage 1 treatments through missed visits, and time to viral resuppression for those who failed through an elevated viral load. As with Aim 1, we will seek differential response by subgroups defined by age, sex, and CD4 level. In exploratory analysis we will treat the two components of the composite endpoint (failure to make visits, and high viral loads) as separate endpoints. *Qualitative analyses:* Analysis of qualitative data will be conducted as per Aim 1 methods above.

**Analysis for Aim 3:**

*Primary analysis of combined adaptive strategies:* The primary outcome for Aim 3, the primary outcome for the trial, is the indicator of sustained viral suppression and engagement in care measured at the end of Year 2 of the study, under each of six strategies. The primary analysis will be comparison of the probability of this outcome between each of four navigator-based strategies vs. reference strategy of SOC-REC and SOC-OIC using TMLE. The current schedule of HIV RNA monitoring is set by Kenyan national guidelines (at six months after treatment initiation and thereafter every 12 months), provided commodities for viral load monitoring are in stock. Elevated HIV RNA levels trigger a repeat test three months later. We will also classify missing viral loads through a process of adjudication as described above. Official transfers out will be censored at the time of transfer out. Deaths will be classified as viral load failures. In order to estimate expected outcome under a given strategy, all subjects assigned to the prevention component of that strategy will initially be eligible; subjects will be censored if and when they deviate from the strategy. Censoring is guaranteed to be random conditional on engagement failure, ensuring that it can be fully adjusted for using inverse weights [[92](#_heading=h.1nia2ey)].

*Secondary analyses*: Secondary outcomes, including mean visit adherence, visit constancy, and on-time pharmacy pick-ups will be compared between each of the embedded strategies. Machine learning-based optimal dynamic treatment rules will be used to identify and evaluate the presence of effect heterogeneity (Montoya, et al 2021; Montoya et al 2021). Sample size: Standard statistical software packages cannot calculate sample size for SMARTs, we therefore carried out calculations using longitudinal maximum likelihood estimation (using the ltmle R package) to multiple iterations of a simulated dataset [[96](#_heading=h.3ls5o66)]. Simulations were based on assumed probability of initial engagement lapse under SOC using preliminary data from study sites, an assumed effect sizes of 12-20% between each of the four navigator-based adaptive strategies in the trial, respectively, and the reference strategy of SOC-REC followed by SOC-OIC if failure occurs. Assuming approximately 810 individuals with a measured outcome at the end of two years (an attrition of 70 from those initially enrolled), under these assumptions we are powered at approximately 80% or more to reject each of the four null hypotheses that each of the navigator-based strategies are the same as the reference strategy. Overall, even for comparisons which are not directly hypothesis tested (as between two non-reference strategies, the width of the confidence intervals (CI) are around point estimates are around 5-10%, thereby providing data on which to appraise individual interventions.

*Cost effectiveness primary analysis:* We will compute unit cost for intervention activity and use information on activities for each participant to compute intervention cost per participant for each intervention strategy. We will quantify *net* *costs* for each intervention strategy – i.e. intervention costs adjusted for added or averted patient and health care costs. Longer-term health care costs will be projected using clinical simulation modeling, based on observed changes in health status, combined with estimates from published studies. Population-level health will be quantified using directly measured health-related study outcomes (retention and viral suppression) and also using *disability-adjusted life years (DALYs)* which includes years of life gained and the collective disability effects of living with HIV. We use Markov modeling to estimate DALYs gained for the short term (during the trial) and the long-term (5, 10, and 20 years) using data from the trial, our own ongoing studies and published estimates of future retention in care, viral suppression, as well as morbidity and mortality attributed to un- or partially- treated HIV disease. We will calculate efficiency and incremental cost-effectiveness for each intervention strategy as net cost per additional person retained, viral suppression achieved and per DALY gained. We will compute *incremental cost-effectiveness ratios (ICERs)* across intervention strategies to establish the relative cost-effectiveness of each intervention strategy. We plan robust sensitivity analyses.

**Analysis for Aim 4**

The post-intervention interviews will be audio-record, the interviewer will write field notes, which capture the composition as well as the tone of the discussion. The interviewer will then listen to the audio-recording and write an analytic memo. The interviewer will write field notes, which capture the composition as well as the tone of the discussion. The interviewer will then listen to the audio-recording and write an analytic memo. Dr. Kwena will debrief regularly with the team as these data are being collected and review the field notes and memos [[88](#_heading=h.3u2rp3q)]. Transcripts of recordings will be coded by qualitative assistants using content analysis, facilitating open coding (inductive approach), a deductive approach that uses the categories using the COM-B framework to advance conceptualization [[89](#_heading=h.2981zbj)] and then develop major themes to understand how interventions worked (or did not work).

For the evaluation of perspectives on peer-delivered interventions, semi-structured interview guides will focus on (1) perceptions of the intervention, (2) perceived benefits and components of the interventions that were most helpful, and (3) unintended consequences and challenges of the interventions. For LHW interviews, we will ask similar themes and additionally explore impact on the LHW themselves (positive and negative). Focus groups will be held separately for facility staff and research teams and will explore perceptions of LHW delivered study interventions, impact, successes, challenges, and suggestions for further adaptation to improve outcomes for participants and support of LHW. Interviews and FGDs will be led by trained Kenyan qualitative research assistants in the preferred language of the participants. Audio-recordings will be transcribed, coded, and processed to derive themes. Transcripts of recordings will be coded by qualitative assistants using content analysis, facilitating open coding (inductive approach), a deductive approach and then develop major themes to understand how interventions worked (or did not work) to support continued treatment success and engagement and impact on LHW.

**Analysis for Aim 5**

Our primary outcome will be sustained engagement in care and sustained viral suppression 6 months post-transition. For those participants who have reached one-year post-transition, we will measure continued care engagement at 1 year as a secondary outcome. We will report the primary outcome overall and by type of intervention every received in participants from both studies. Further, we will determine sustained engagement and retention by demographic and clinical characteristics.

# TIME FRAME

The study, including preparation, enrollment, follow up, and analysis, is projected to last 5 years, with Year 1 beginning January 2020. We anticipate active enrollment for up to two years but possibly less (Table 2). Enrolled participants will each be followed for a maximum of 4 years

| **Timeline** | Year 1 | | | | Year 2 | | | | Year 3 | | | | Year 4 | | | | Year 5 | | | |
| --- | --- | --- | --- | --- | --- | --- | --- | --- | --- | --- | --- | --- | --- | --- | --- | --- | --- | --- | --- | --- |
| **Aim 1:**  **Assess the effectiveness of prevention intervention among 880 adolescent patients starting treatment in Kenya (Stage 1)** | | | | | | | | | | | | | | | | | | | | |
| Obtain IRB approval, hire, and train staff |  |  |  |  |  |  |  |  |  |  |  |  |  |  |  |  |  |  |  |  |
| Refine interventions to developmental phases and preferences of patient population (focus group discussions, design workshop) |  |  |  |  |  |  |  |  |  |  |  |  |  |  |  |  |  |  |  |  |
| Operationalize study procedures |  |  |  |  |  |  |  |  |  |  |  |  |  |  |  |  |  |  |  |  |
| Recruit/enroll 880 adolescents and randomize to Stage 1 interventions |  |  |  |  |  |  |  |  |  |  |  |  |  |  |  |  |  |  |  |  |
| Interim review and analyses of Stage 1 intervention experience |  |  |  |  |  |  |  |  |  |  |  |  |  |  |  |  |  |  |  |  |
| Monitoring and analysis of data quality and intervention fidelity |  |  |  |  |  |  |  |  |  |  |  |  |  |  |  |  |  |  |  |  |
| Follow adolescent participants for two years |  |  |  |  |  |  |  |  |  |  |  |  |  |  |  |  |  |  |  |  |
| **Aim 2:**  **Assess the effect of re-engagement intervention among adolescents who have missed a visit by 14 days (Stage 2)** | | | | | | | | | | | | | | | | | | | | |
| Re-randomization for treatment lapse to Stage 2 interventions |  |  |  |  |  |  |  |  |  |  |  |  |  |  |  |  |  |  |  |  |
| Interim review and analyses of re-engagement intervention experiences |  |  |  |  |  |  |  |  |  |  |  |  |  |  |  |  |  |  |  |  |
| Monitoring and analyses of data quality and intervention fidelity |  |  |  |  |  |  |  |  |  |  |  |  |  |  |  |  |  |  |  |  |
| Follow re-randomized adolescents for total of two years |  |  |  |  |  |  |  |  |  |  |  |  |  |  |  |  |  |  |  |  |
| **Aim 3: Assess the effectiveness and cost effectiveness of the best sequenced strategy for retention** | | | | | | | | | | | | | | | | | | | | |
| Conduct cost effectiveness assessment and analysis* |  |  |  |  |  |  |  |  |  |  |  |  |  |  |  |  |  |  |  |  |
| Clean and analyze quantitative data |  |  |  |  |  |  |  |  |  |  |  |  |  |  |  |  |  |  |  |  |
| Disseminate study findings to inform best practices |  |  |  |  |  |  |  |  |  |  |  |  |  |  |  |  |  |  |  |  |

# EXPECTED APPLICATION OF THE RESULTS

This study will allow us to move towards individualized public health by providing evidence-based retention interventions that offer the right intervention to the right patient at the right time, thereby *simultaneously optimizing efficiency and effectiveness*. The long term impact will be to maximize the public health benefits of care and treatment for HIV, reduce morbidity and mortality among AYA, and restore the economic and social productivity of communities affected by HIV/AIDS.

We will share the findings from this study with the health facilities involved, community leaders, County Ministers of Health, and NASCOP. Moreover, we will disseminate our findings at local, national, regional, and international meetings and conferences.

#

# REFERENCES

**References**

1. Lei, H., et al., *A "SMART" design for building individualized treatment sequences.* Annu Rev Clin Psychol, 2012. **8**: p. 21-48.

2. Murphy, S.A., *An experimental design for the development of adaptive treatment strategies.* Stat Med, 2005. **24**(10): p. 1455-81.

3. Murphy, S.A. and L.M. Collins, *Customizing treatment to the patient: Adaptive treatment strategies.* Drug Alcohol Depend, 2007. **88**(Suppl 2): p. S1.

4. Murphy, S.A., et al., *Developing adaptive treatment strategies in substance abuse research.* Drug Alcohol Depend, 2007. **88 Suppl 2**: p. S24-30.

5. Chung, M.H., et al., *A randomized controlled trial comparing the effects of counseling and alarm device on HAART adherence and virologic outcomes.* PLoS Med, 2011. **8**(3): p. e1000422.

6. Pop-Eleches, C., et al., *Mobile phone technologies improve adherence to antiretroviral treatment in a resource-limited setting: a randomized controlled trial of text message reminders.* AIDS, 2011. **25**(6): p. 825-34.

7. Baird, S., et al., *The short‐term impacts of a schooling conditional cash transfer program on the sexual behavior of young women.* Health economics, 2010. **19**(S1): p. 55-68.

8. Aad, G., et al., *Two-particle Bose-Einstein correlations in pp collisions at [Formula: see text] 0.9 and 7 TeV measured with the ATLAS detector.* Eur Phys J C Part Fields, 2015. **75**(10): p. 466.

9. Lester, R.T., et al., *Effects of a mobile phone short message service on antiretroviral treatment adherence in Kenya (WelTel Kenya1): a randomised trial.* Lancet, 2010. **376**(9755): p. 1838-45.

10. Kunutsor, S., et al., *Improving clinic attendance and adherence to antiretroviral therapy through a treatment supporter intervention in Uganda: a randomized controlled trial.* AIDS Behav, 2011. **15**(8): p. 1795-1802.

11. Nachega, J.B., et al., *Randomized controlled trial of trained patient-nominated treatment supporters providing partial directly observed antiretroviral therapy.* Aids, 2010. **24**(9): p. 1273-80.

12. Slogrove, A.L., et al., *Living and dying to be counted: What we know about the epidemiology of the global adolescent HIV epidemic.* J Int AIDS Soc, 2017. **20**(Suppl 3): p. 21520.

13. Enane, L.A., R.C. Vreeman, and C. Foster, *Retention and adherence: global challenges for the long-term care of adolescents and young adults living with HIV.* Curr Opin HIV AIDS, 2018. **13**(3): p. 212-219.

14. Petersen, M., et al., *Association of Implementation of a Universal Testing and Treatment Intervention With HIV Diagnosis, Receipt of Antiretroviral Therapy, and Viral Suppression in East Africa.* JAMA, 2017. **317**(21): p. 2196-2206.

15. Ryscavage, P.A., et al., *Clinical outcomes of adolescents and young adults in adult HIV care.* JAIDS Journal of Acquired Immune Deficiency Syndromes, 2011. **58**(2): p. 193-197.

16. Wong, V.J., et al., *Adolescents, young people, and the 90-90-90 goals: a call to improve HIV testing and linkage to treatment.* AIDS, 2017. **31 Suppl 3**: p. S191-S194.

17. Enane, L.A., et al., *Traversing the cascade: urgent research priorities for implementing the 'treat all' strategy for children and adolescents living with HIV in sub-Saharan Africa.* J Virus Erad, 2018. **4**(Suppl 2): p. 40-46.

18. McHugh, G., et al., *Clinical outcomes in children and adolescents initiating antiretroviral therapy in decentralized healthcare settings in Zimbabwe.* J Int AIDS Soc, 2017. **20**(1): p. 21843.

19. Ammon, N., S. Mason, and J. Corkery, *Factors impacting antiretroviral therapy adherence among human immunodeficiency virus–positive adolescents in Sub-Saharan Africa: a systematic review.* Public health, 2018. **157**: p. 20-31.

20. Ojwang, V.O., et al., *Loss to follow-up among youth accessing outpatient HIV care and treatment services in Kisumu, Kenya.* AIDS Care, 2016. **28**(4): p. 500-7.

21. Reif, L.K., et al., *Impact of a youth-friendly HIV clinic: 10 years of adolescent outcomes in Port-au-Prince, Haiti.* J Int AIDS Soc, 2016. **19**(1): p. 20859.

22. Teasdale, C.A., et al., *Impact of Youth and Adolescent Friendly Services on Retention of 10-24-Year-Olds in HIV Care and Treatment Programs in Nyanza, Kenya.* J Acquir Immune Defic Syndr, 2016. **71**(2): p. e56-9.

23. Cluver, L., et al., *STACKing the odds for adolescent survival: health service factors associated with full retention in care and adherence amongst adolescents living with HIV in South Africa.* J Int AIDS Soc, 2018. **21**(9): p. e25176.

24. (FACES), F.A.C.a.E.S., *FACES Internal Program Data*. 2018: Kisumu, Kenya.

25. Odeny, T.A., et al. *Texting improves testing: a randomised controlled trial of text messaging to increase postpartum attendance and rates of early infant diagnosis of HIV*. in *7th International AIDS Society Conference on HIV Pathogenesis, Treatment and Prevention*. 2013. Kuala Lumpur

26. Odeny, T.A., et al., *Text messaging to improve attendance at post-operative clinic visits after adult male circumcision for HIV prevention: a randomized controlled trial.* PLoS One, 2012. **7**(9): p. e43832.

27. Rana, Y., et al., *Short message service (SMS)-based intervention to improve treatment adherence among HIV-positive youth in Uganda: focus group findings.* PloS one, 2015. **10**(4): p. e0125187.

28. Kassaye, S.G., et al., *Cluster-Randomized Controlled Study of SMS Text Messages for Prevention of Mother-to-Child Transmission of HIV in Rural Kenya.* AIDS Res Treat, 2016. **2016**: p. 1289328.

29. Brechwald, W.A. and M.J. Prinstein, *Beyond Homophily: A Decade of Advances in Understanding Peer Influence Processes.* J Res Adolesc, 2011. **21**(1): p. 166-179.

30. Wilson, K.S., et al., *"At our age, we would like to do things the way we want: " a qualitative study of adolescent HIV testing services in Kenya.* AIDS, 2017. **31 Suppl 3**: p. S213-S220.

31. Organisation, W.H., *Providing peer support for adolescents and young people living with HIV*, P. Child Survival Working Group: EDC, UNICEF, WHO, Editor.

32. Foundation, E.G.P.A., *Incentives Handbook for Adolescents and Health Care Providers (Kenya)*. 2017.

33. Kranzer, K., et al., *Economic incentives for HIV testing by adolescents in Zimbabwe: a randomised controlled trial.* Lancet HIV, 2018. **5**(2): p. e79-e86.

34. Yotebieng, M., et al., *Continuous quality improvement interventions to improve long-term outcomes of antiretroviral therapy in women who initiated therapy during pregnancy or breastfeeding in the Democratic Republic of Congo: design of an open-label, parallel, group randomized trial.* BMC Health Serv Res, 2017. **17**(1): p. 306.

35. Pettifor, A., et al., *The effect of a conditional cash transfer on HIV incidence in young women in rural South Africa (HPTN 068): a phase 3, randomised controlled trial.* Lancet Glob Health, 2016. **4**(12): p. e978-e988.

36. Loewenstein, G., T. Brennan, and K.G. Volpp, *Asymmetric paternalism to improve health behaviors.* JAMA, 2007. **298**(20): p. 2415-7.

37. O’Donoghue, T. and M. Rabin, *Doing it now or later.* American Economic Review, 1999. **89**(1): p. 103-124.

38. Thaler, R.H. and C.R. Sunstein, *Libertarian paternalism.* The American Economic Review, 2003. **93**(2): p. 175-179.

39. Thaler, R.H. and C.R. Sunstein, *Nudge: Improving decisions about health, wealth, and happiness*. 2008: Yale University Press.

40. Volpp, K.G., et al., *P4P4P: an agenda for research on pay-for-performance for patients.* Health Affairs, 2009. **28**(1): p. 206-14.

41. Lussier, J.P., et al., *A meta-analysis of voucher-based reinforcement therapy for substance use disorders.* Addiction, 2006. **101**(2): p. 192-203.

42. Banerjee, A.V., et al., *Improving immunisation coverage in rural India: clustered randomised controlled evaluation of immunisation campaigns with and without incentives.* BMJ, 2010. **340**: p. c2220.

43. Thornton, R., *The Demand for, and Impact of, Learning HIV Status.* American Economic Review, 2008. **98**(5): p. 1829-1863.

44. Rivera, J.A., et al., *Impact of the Mexican program for education, health, and nutrition (Progresa) on rates of growth and anemia in infants and young children: a randomized effectiveness study.* JAMA, 2004. **291**(21): p. 2563-70.

45. Schultz, T.P., *School Subsidies for the Poor: Evaluating the Mexican Progresa Poverty Program.* Journal of Development Economics, 2004. **74**(1): p. 199-250.

46. Baird, S.J., et al., *Effect of a cash transfer programme for schooling on prevalence of HIV and herpes simplex type 2 in Malawi: a cluster randomised trial.* The Lancet, 2012.

47. Cluver, L., et al., *Child-focused state cash transfers and adolescent risk of HIV infection in South Africa: a propensity-score-matched case-control study.* The Lancet Global Health, 2013. **1**(6): p. e362-e370.

48. (ICAP), I.C.f.A.C.a.T.P.s., *Comprehensive Peer Educator Training Curriculum:Trainer Manual*.

49. Geng, E.H., Odeny, T.A., Lyamuya, R., Nakiwogga-Muwanga, A., Bwana, M. *A Sampling-based Approach to Assessing Patient-reported Structural, Clinic-based and Psychosocial Barriers to Retention in Care among HIV-infected Patients on Antiretroviral Therapy in East Africa* in *20th Conference on Retroviruses and Opportunistic Infections* 2013. Atlanta.

50. Geng, E.H., et al., *Retention in Care and Patient-Reported Reasons for Undocumented Transfer or Stopping Care Among HIV-Infected Patients on Antiretroviral Therapy in Eastern Africa: Application of a Sampling-Based Approach.* Clin Infect Dis, 2016. **62**(7): p. 935-944.

51. Odeny, T.A., et al., *Maximizing adherence and retention for women living with HIV and their infants in Kenya (MOTIVATE! study): study protocol for a randomized controlled trial.* Trials, 2018. **19**(1): p. 77.

52. Dillabaugh, L.L., et al., *Towards Elimination of Mother-to-Child Transmission of HIV: The Impact of a Rapid Results Initiative in Nyanza Province, Kenya.* AIDS Research and Treatment. **2012**: p. 602120.

53. Odeny, T.A., et al., *Texting improves testing: a randomized trial of two-way SMS to increase postpartum prevention of mother-to-child transmission retention and infant HIV testing.* Aids, 2014. **28**(15): p. 2307-12.

54. Musoke, P., et al., *A text messaging intervention to support Option B+ in Kenya: A qualitative study.* Journal of the Association of Nurses in AIDS Care, 2018. **29**(2): p. 287-299.

55. Orme, B., *Getting Started with Conjoint Analysis: Strategies for Product*

*Design and Pricing Research*. Second Edition ed. 2010, Madison, Wisconsin, USA: Research Publishers LLC.

56. Bello, G. *HIV drug resistance and tracing outcomes among antiretroviral therapy defaulters in Malawi*. in *27th International Workshop on HIV Drug Resistance and Treatment Strategies*. 2018. Johanesburg, South Africa.

57. Deeks, S.G., et al., *Virologic and immunologic consequences of discontinuing combination antiretroviral-drug therapy in HIV-infected patients with detectable viremia.* New England Journal of Medicine, 2001. **344**(7): p. 472-480.

58. *National Guidelines for the provision of adolescent and youth friendly services in Kenya*. 2017, Ministry of Health, Nairobi, Kenya Ministry of Health.

59. Janz, N.K. and M.H. Becker, *The health belief model: A decade later.* Health Education & Behavior, 1984. **11**(1): p. 1-47.

60. Lapinski, M.K. and R.N. Rimal, *An explication of social norms.* Communication theory, 2005. **15**(2): p. 127-147.

61. Martin, S., *98% of HBR Readers Love This Article.* Harvard Business Review, 2012. **90**(10): p. 23-25.

62. Emmons, K.M., et al., *Peer-delivered smoking counseling for childhood cancer survivors increases rate of cessation: the partnership for health study.* Journal of Clinical Oncology, 2005. **23**(27): p. 6516-6523.

63. Funck-Brentano, I., et al., *Evaluation of a peer support group therapy for HIV-infected adolescents.* AIDS, 2005. **19**(14): p. 1501-8.

64. Gittings L, M.D., Soeters H, Ronan A, Hatane L, *“If you trust us, help us to do it by ourselves”: Promising practices in peer support for adolescents and young people living with HIV.* 2017, PATA Pediatric-Adolescent Treatment Africa.

65. Rotheram-Borus, M.-J., et al., *Project Masihambisane: a cluster randomised controlled trial with peer mentors to improve outcomes for pregnant mothers living with HIV.* Trials, 2011. **12**(2): p. 1-10.

66. Wong, C.A., et al., *Effect of financial incentives on glucose monitoring adherence and glycemic control among adolescents and young adults with type 1 diabetes: a randomized clinical trial.* JAMA pediatrics, 2017. **171**(12): p. 1176-1183.

67. Cluver, L.D., et al., *Cash plus care: social protection cumulatively mitigates HIV-risk behaviour among adolescents in South Africa.* Aids, 2014. **28**: p. S389-S397.

68. Wakadha, H., et al., *The feasibility of using mobile-phone based SMS reminders and conditional cash transfers to improve timely immunization in rural Kenya.* Vaccine, 2013. **31**(6): p. 987-993.

69. Odeny, T.A., et al., *Effect of Text Messaging to Deter Early Resumption of Sexual Activity after Male Circumcision for HIV Prevention: A Randomized Controlled Trial.* JAIDS Journal of Acquired Immune Deficiency Syndromes, 2013.

70. Tuller, D.M., et al., *Transportation Costs Impede Sustained Adherence and Access to HAART in a Clinic Population in Southwestern Uganda: A Qualitative Study.* AIDS Behav, 2009.

71. Hardon, A.P., et al., *Hunger, waiting time and transport costs: time to confront challenges to ART adherence in Africa.* AIDS Care, 2007. **19**(5): p. 658-65.

72. Geng, E.H., et al., *Understanding reasons for and outcomes of patients lost to follow-up in antiretroviral therapy programs in Africa through a sampling-based approach.* J Acquir Immune Defic Syndr, 2010. **53**(3): p. 405-11.

73. Maskew, M., et al., *Lost to follow up: contributing factors and challenges in South African patients on antiretroviral therapy.* S Afr Med J, 2007. **97**(9): p. 853-7.

74. McGuire, M., et al., *Vital Status of pre-ART and ART patients defaulting from care in rural Malawi.* Tropical Medicine & International Health, 2010. **15**(S1): p. 55-62.

75. Kiwanuka, S., et al., *Access to and utilisation of health services for the poor in Uganda: a systematic review of available evidence.* Transactions of the Royal Society of Tropical Medicine and Hygiene, 2008. **102**(11): p. 1067-1074.

76. Cluver, L.D., et al., *Social protection: potential for improving HIV outcomes among adolescents.* Journal of the International AIDS Society, 2015. **18**: p. 20260.

77. Barber, S.L. and P.J. Gertler, *The impact of Mexico’s conditional cash transfer programme, Oportunidades, on birthweight.* Tropical Medicine & International Health, 2008. **13**(11): p. 1405-1414.

78. Davis, B., et al., *Evaluating the impact of cash transfer programmes in sub-Saharan Africa: an introduction to the special issue.* Journal of development effectiveness, 2012. **4**(1): p. 1-8.

79. Mulvaney, S. and J.M. Lee, *Motivating Health Behaviors in Adolescents Through Behavioral Economics.* JAMA pediatrics, 2017. **171**(12): p. 1145-1146.

80. Edejer, T.T.-T., *Making choices in health: WHO guide to cost effectiveness analysis*. 2003: World Health Organization.

81. Sanders, G.D., et al., *Recommendations for conduct, methodological practices, and reporting of cost-effectiveness analyses: second panel on cost-effectiveness in health and medicine.* Jama, 2016. **316**(10): p. 1093-1103.

82. Crane, P.K., et al., *Measuring depression levels in HIV-infected patients as part of routine clinical care using the nine-item Patient Health Questionnaire (PHQ-9).* AIDS Care, 2010. **22**(7): p. 874-85.

83. Reece, M., Kroenke, K., Otieno, O., Yebei, V., Ojwang, C., *Validity/reliability of PHQ-9 and PHQ-2 depression scales among adults living with HIV/AIDS in western Kenya.* J Gen Intern Med, 2009. **24**(2): p. 189-197.

84. Beresford, T.P., et al., *Comparison of CAGE questionnaire and computer-assisted laboratory profiles in screening for covert alcoholism.* The Lancet, 1990. **336**(8713): p. 482-485.

85. Ewing, J.A., *Detecting alcoholism.* JAMA: the journal of the American Medical Association, 1984. **252**(14): p. 1905-1907.

86. Jain, V., et al., *Assessment of Population-Based HIV RNA Levels in a Rural East African Setting Using a Fingerprick-Based Blood Collection Method.* Clinical infectious diseases, 2013. **56**(4): p. 598-605.

87. Muennig, P., *Cost-Effectiveness Analysis in Health: A Practical Approach* 2nd ed. 2007, San Francisco: Wiley & Sons.

88. McMahon, S.A. and P.J. Winch, *Systematic debriefing after qualitative encounters: an essential analysis step in applied qualitative research.* BMJ global health, 2018. **3**(5): p. e000837.

89. Elo, S. and H. Kyngäs, *The qualitative content analysis process.* Journal of advanced nursing, 2008. **62**(1): p. 107-115.

90. Mugavero, M.J., et al., *From access to engagement: measuring retention in outpatient HIV clinical care.* AIDS Patient Care STDS, 2010. **24**(10): p. 607-613.

91. Bisson, G.P., et al., *Pharmacy refill adherence compared with CD4 count changes for monitoring HIV-infected adults on antiretroviral therapy.* PLoS Med, 2008. **5**(5): p. e109.

92. Hernán, M.A., et al., *Comparison of dynamic treatment regimes via inverse probability weighting.* Basic & clinical pharmacology & toxicology, 2006. **98**(3): p. 237-242.

93. van der Laan, M.J., Gruber, S., *Targeted Minimum Loss Based Estimation of Causal Effects of Multiple Time Point Interventions.* International Journal of Biostatistics, 2012. **8**(1): p. 1557.

94. van der Laan, M.J. and M.L. Petersen, *Causal effect models for realistic individualized treatment and intention to treat rules.* International Journal of Biostatistics, 2007. **3**(1): p. 3.

95. Robins, J., L. Orellana, and A. Rotnitzky, *Estimation and extrapolation of optimal treatment and testing strategies.* Statistics in medicine, 2008. **27**(23): p. 4678-4721.

96. Petersen, M.L., Schwab, J., Gruber, S., Blaser, N., Schomaker, M., van der Laan, M.J., *Targeted Maximum Likelihood Estimation for Dynamic and Static Longitudinal Marginal Structural Working Models <*[*http://biostats.bepress.com/ucbbiostat/paper312*](http://biostats.bepress.com/ucbbiostat/paper312)*>.* U.C. Berkeley Division of Biostatistics Working Paper Series, 2013. **Working Paper 13**.

#

# BUDGET

|  | **YEAR 1 (USD)** | **YEAR 1KSH**  (rate: 99) |
| --- | --- | --- |
| 1. **PERSONNEL COSTS** |  |  |
| Salaries and gratuity (20%) | $240,192 | 23,779,008 |
| Personnel benefits (Staff Health Insurance, Workers Injury Benefits Insurance, NSSF) | $20,223 | 2,002,077 |
| **PERSONNEL TOTAL** | **$260,415** | **25,781,085** |
| 1. **TRAVEL** |  |  |
| Local Travel | $2,500 | 247,500 |
| International Travel | $4,000 | 396,000 |
| **TRAVEL TOTAL** | **$6,500** | **643,500** |
| 1. **SUPPLIES and OTHER:** |  |  |
| Laptops, server, furniture, office maintenance, office supplies, phones. | $13,746 | 1,360,854 |
| Vouchers | $3,270 | 323,730 |
| Training Costs | $3,500 | 346,500 |
| Focus groups, design workshop, and interviews | $2,188 | 216,612 |
| Patient Outreach Costs | $1,500 | 148,500 |
| Rent and Utilities | $14,000 | 1,386,000 |
| **SUPPLIES and OTHER TOTAL** | **$38,204** | **3,782,196** |
| 1. **OTHER COSTS** |  |  |
| Internet and telephones | $6,000 | 594,000 |
| Printing and reproduction | $1,000 | 99,000 |
| Vehicle and motorcycle insurance, fuel and maintenance | $5,000 | 495,000 |
| **OTHER TOTAL** | **$12,000** | **1,188,000** |
| **Total Direct Costs** | **$317,119** | **31,394,781** |
| **Indirect Costs (8%)** | **$25,108** | **2,485,692** |
| **TOTAL COSTS** | **$342,227** | **33,880,473** |

## BUDGET JUSTIFICATION

**PERSONNEL**

**Site Principal Investigator: Professor Elizabeth Bukusi.** As the Site Principal Investigator of this study, Prof Bukusi’s role will be oversight of research development and implementation in Kenya. She will facilitate collaboration between WU and KEMRI, serve on the Scientific Steering Committee with the MPI’s, oversee all phases of study development and implementation in the field, and assure for feasibility and cultural appropriateness of study procedures and materials. She will be the chief liaison with the local HIV implementing partners, local government, and KEMRI leadership

**Co-Investigator: Eliud Akama.**

He will be responsible for overseeing the day-to-day study activities, managing the study staff, tracking study progress, and will communicate closely with the KEMRI investigators and MPIs. He will be responsible for facilitating study training, managing the local regulatory submissions, and ensuring for protocol compliance

**Co-Investigator: Dr. Zachary Kwena**

He will lead and supervise implementation of qualitative activities including focus group and individual interview guide development, conduct, coding/analysis, integration into intervention refinement and results dissemination. Specifically, In Year 1 he will oversee activities to obtain adolescent input through focus groups to tailor the interventions to the AYA context and oversee the design workshop to translate FGD findings into adapted interventions for AYA. He will also facilitate gathering patient experiences with the interventions through in-depth interviews. He will work closely with Drs. Abuogi, Dr. Odeny, and the qualitative coordinator to carry out these activities.

**Finance Manager: Kevin Okoth.** The Finance Manager at 25% effort is based in Nairobi. He will be instrumental in budget management, fast tracking of project payments to suppliers, and facilitating staff contracts and subcontracts. He will be key in processing invoices and cash transfer requests to the donor. He will also provide overall supervision to the study administrator and support on all financial matters related to the project.

**Study Coordinator; Sarah Iguna.** She will be responsible for overseeing the day-to-day study activities, managing the study staff, and reporting to the PIs on the study progress. She will supervise the Assistant Coordinator and County Level Navigator. She will conduct site level visits to assess, for, mentor, and consistently ensure for protocol and standard operating procedure compliance. She will be responsible for managing study human resource needs, facilitating study training, and managing the local regulatory submissions and reporting. She will be in frequent communication with the local PIs and work closely the US-based investigator team, project coordinator, local data manager, and local administrator to ensure for smooth and compliant study operations.

**Assistant Study Coordinator;** **Harriet Adhiambo.** She will report to the study coordinator and be responsible for supervising study activities at the three clinic sites. She will provide key support to study implementation. She will mentor study staff on recruitment, screening, informed consent, enrollment, data collection and patient follow up. She will generate study reports to facilitate daily tracing and study management, provide written reports on site progress and performance as may be required. She will help train and support Research Assistants on study protocol and standard operating procedure compliance.

**Data Manager/Analyst; (TBN)** The Data Manager/Analyst will manage the local data system, ensure data is transmitted securely and efficiently to the central server ensure for high quality study data and develop and generate preliminary findings for review. He/she will spear head preparation of standard operating procedures for data management, including quality control procedures and verification processes, and prepare data source documentation. He/she will program and test study surveys, train the study team on data entry procedures, troubleshoot data issues that may arise, respond to data queries, and generate routine data reports. He/she will report to the local PIs, supervise the Assistant Data Manager, work closely with the Programmer and ICT Officer, coordinators and the US Analyst.

**Assistant Data Manager; Evelyne Nyandiek.** The Assistant Data Manager will assist the Data Manager in preparation of standard operating procedures for data management, including quality control procedures to review source documentation for completion, respond to data queries and quality control reports, and ensure overall high-quality data at the site.

**Qualitative Researcher: (TBN).** She/he will work closely with the co-investigator, Dr. Kwena, to carry out the qualitative research activities including workplan development, FGD and interview organization and facilitation, and support transcript coding.

**Site Lead Research Assistant (TBN).** He/she will be responsible for overseeing study management at the site, including patient recruitment, enrollment, tracing, data collection and data entry, documentation, and organization per the study protocol and standard operating procedures. He/she will make sure the site is well stocked and managed. He/she will cultivate and maintain a cohesive working relationship between the study and clinic staff. In addition to site oversight, this person will also participate in daily patient recruitment, screening, informed consent, enrollment, data collection and entry, and patient follow up. This person will communicate regularly with the Assistant Study Coordinator and Study Coordinator and other study staff and will respond to data and study queries with information and reports as required.

**Navigator Mentor.** The Navigator Mentor will work at the county level and be responsible supervising, training, mentoring, and supporting the Peer Navigators at each of the sites. He/she build their skills in counseling, psychosocial support, rapport and trust building, and ability to probe, identify, and address engagement in care issues resourcefully and with a high level of sensitivity, understanding, and complete confidentiality. She/he will have frequent communication with the Peer Navigators and observe and critique their work for continuous improvement. He/she will report to the Study Coordinator.

**Research Assistant/Peer Navigator (TBN).** Eight Research Assistants will be cross trained in the Peer Navigation role. This will enable the study to better match patients randomized to e-navigation and in-person Navigation by age and gender and allow a for a manageable workload. In their capacity as a Research Assistant they will conduct patient recruitment, screening, informed consent, randomization/re-randomization, data collection and entry, and patient follow up in compliance with the study protocol. They will maintain study registers, logs, and files and make sure all study documents are kept secure and locked. They also trace patients by phone and or in the field. They will develop and maintain strong positive relationships with community and community leaders to facilitate patient tracing activities. They will maintain a high level of patient confidentiality. In their Peer Navigator role they will be responsible for delivering e-navigation and in-person Navigation intervention with regular contact per the study protocol. They will work closely with study participants to identify and address barriers to engagement in care, provide psychosocial support, and work resourcefully to re-engage them in HIV care.

**Administrator: Irene Orenyo.** This administrator will be responsible for assisting in overall planning for the project in liaison with the Study coordinator, budget preparation, maintaining of proper audit trail for study funds and expenditures, generate monthly invoices, and other financial reports as required in the subcontract. In liaison with the supplies officer she will facilitate departmental requisitions and procurements while adhering to administrative and procurement policies. She will also be responsible for the handling and accounting for the field imprest given to the project.

**Driver:** Responsible for the project vehicle and driving of project staff and participants as required. He will be based in Kisumu. The driver will also double up as a cleaner/messenger at the study office. He will report to the administrator.

**Costing Research Assistant: (TBN).** The Costing Research Assistant will support the gathering, management, and analysis of cost and output data from the 12 participating communities in Kenya. The work encompasses data collection, data management, preliminary analysis, and writing. The data will be collected mainly through two methods - micro-costing and time and motion (T&M). The Costing Research Assistant will work closely with the Dr. Starley Shade and the research team.

**Programmer, (TBN)** The programmer will play an integral role in the development and execution of the electronic data enterprise for the study. He/she will help to develop and implement a data system that combines clinical data from the EMR platform and study survey data on the ODK platform and migrates that data to central local data server. He/she will create and facilitate queries for ongoing to data integrity and consumption. He/she will work to develop user-facing modules for RA use. Under the direction of the investigators, he/she will assist with system design, testing, and implementation. He/she will work closely with study data manager/analyst and IT manager to facilitate the testing, roll-out, and maintenance of the data system. The programmer will be well-versed in Javascript frameworks (Node.JS, angular or react etc), Java, SQL.

**ICT Officer; (TBN)** The IT support will work in tandem with the programmer to network, test, monitor, and maintain the study data system. He/she will provide support to the programmer and end users on system utilization, as well as troubleshoot and resolve data issues that may arise. He/she will also help develop and maintain IT documentation, processes and procedures.

**OTHER EXPENSES**

Travel

1. Local Travel: This cost item will go toward supporting the study coordinator, data manager, and county research assistant and co-investigators to perform routine site supervision and assessments, study protocol monitoring, and data system checks.
2. International Travel: The budget for international travel has been allocated to the PIs and the coordinators to attend annual international investigator meetings e.g. Conference on Retroviruses and Opportunistic Infections (CROI). Such meetings will offer platforms to disseminate project results, build scientific capacity and professional networks, and the opportunity to obtain valuable exposure to the research community.

Participant Costs

1. Participant reimbursement: Participants will receive approximately $5 reimbursement for their time and transport costs at study enrollment. Also, participants who are randomized to the Conditional Cash Transfer arm will receive approximately $5 at each scheduled clinic visit for one year and an additional $5 for suppressed viral load. Participants who take part in the focus groups and individual interviews will also receive approximately $5 for their time and transport costs.

Supplies

1. Office supplies: The supplies needed include pens, paper, markers, rulers, diaries, message pads, staples, envelopes, box files, and spring files, file separators, labels, binders, paper clips, cello tapes, glue sticks, cartridges, staples, pins and badges among others.
2. Furniture, fittings, and office maintenance: Funds have been allocated chairs, tables, desks, cabinets, as well as for small office repairs that may be needed.
3. Central servers, laptops, phones, tablets and software licenses: Costs have been allocated for a central server to house all study data securely, laptops, phones, tablets, and licenses to support the study activities. Laptops, tablets, and smart phones are needed for entering clinical records into secure electronic medical records and platforms for study management. Staff will require phones for contacting study participants, communication with the study team, and for administering and entering survey data into the data platforms on phones. The central server is approximately $2,500, laptops are approximately $1,500 each, tablets approximately $300, and smart phones $200 each. Software and annual licensing for antivirus, data manipulation, qualitative analyses are needed for the laptop security and study data management and analyses.

Other

1. Printing, reproduction, photocopier: To print study training materials, consent forms, tools, and other related study documents.
2. Vehicle and motorcycle insurance, fuel & maintenance: Funds for fueling the study vehicle and four motorcycles. These funds will cover monthly maintenance costs, fuel, annual insurance and purchase of tires.
3. Focus groups: Three groups of 8 participants will participate in three formative AYA focus group discussions (FGD), one per intervention: e-navigation, CCT, and in-person navigation in year 1 only. The FGDs will solicit AYA insight and preferences on the intervention content and delivery for the AYA context. The focus groups will be co-facilitated by the qualitative researcher and adolescent peer leader however an outside note-taker will be brought in for the sessions at a cost of $180 based on $60 per session; transcription costs will be $240, based on $80 per focus group transcription. Participants will be provided with light refreshments at cost of $48, based on $2 per participant. Participants will also be reimbursed for their time and transport which is listed below in participant reimbursement.
4. Design workshop: As part of intervention refinement for the AYA population, a 3-day design workshop will be held with e-navigators and AYA patients to translate focus group findings into a package of interventions tailored to the AYA context in year 1. $300 is budgeted for a facilitator skilled in cognitive interviewing, based on $100 per day for 3 days and $800 for lunch and refreshments at the in-house workshop for 23 participants: 12 AYA patients, 8 e-navigators, 1 facilitator, and 2 staff members for 3 days.
5. Individual interviews: 60 interviews, 20 per three study arms (e-navigation, in-person navigation, and cost transfer), will be conducted to understand and learn from AYA experiences with each intervention. In addition, each of the peer navigators will participate in-depth interviews to understand their perceptions of how the e-navigation and in-person navigation interventions influenced participants. The interviews will be conducted by the qualitative researcher beginning in the later part of year 1 and concluding in year 3. Each interview will be transcribed at a cost of $50 per interview and refreshments will be provided at cost of $2 per interview.
6. Meetings and Trainings: There will be two study team meetings per year for training, refresher training, and study progress review including a one-day annual retreat with the MPIs. These meetings will ensure standard operating procedures are being followed, enrollment targets are being met, and data collection is appropriate and adequate. These meetings will also allow for problem-solving for challenges met on the ground. The youth community advisory board will meet twice annually.
7. Patient Outreach: These funds are allocated for tracing participants who miss their clinic and Navigator intervention sessions. These funds will also cater for light refreshments offered to study participants during study enrollment and follow up survey data collection.
8. Rent and Utilities: These are costs associated with renting out a premise for study office use, its maintenance and its security. Rent and security costs are incurred to keep the office and secure it. Utilities are water, electricity and garbage collection which are used by staff occupying the office.
9. Telephone and Internet Communications: This includes internet services costs, airtime for both staff and sites for communication. Server costs are costs for sending out automated short messages (through Nyaruka) to participants who are in the e-navigation arm intervention. Nyaruka costs are the costs for setting up the TextIT application for short messages and its maintenance.
10. Indirect Costs. Indirect costs are calculated at the research rate of 8%, allowable per NIH Grants Policy Statement for foreign instituion.

# INVESTIGATOR ROLES

Global health research often requires teams that are distributed across countries and institutions to achieve a broad range of academic and implementation expertise. Our team, assembled after a decade of collaboration, brings together complementary expertise to ensure effective team science. We combine a strong track record with management plans that ensure we can harness the strengths of a multi-institutional proposal. **Elvin Geng,** **MD, MPH** is Professor of Medicine at Washington University and studies engagement in HIV care in Africa using observational epidemiology as well as individual and cluster randomized trials. He is principal investigator or co-investigator on several NIH-funded studies, including two using a SMART design (NCT02338739 and NCT03500172). **Lisa** **Abuogi,** **MD, MS** is an Associate Professor of Pediatrics in Infectious Diseases at University of Colorado, Denver. After leading clinical care for Family AIDS Care and Education Services (FACES) for three years in Kisumu, she now leads, as PI, two NIH-funded projects focused on HIV among pregnant women (NCT02491177) and adolescents (NCT03820323) in Kenya. She has served on the Kenya National Working Group for Pediatric and Adolescent HIV. Together, Drs. Geng and Abuogi have critical breadth of expertise to lead this study. **Elizabeth Bukusi, MBChB, MMED, MPH, PhD, PGD, MBE** is a Chief Research Officer and Co-Director of the Research, Care, and Training Program at KEMRI and an internationally known researcher in HIV as well as collaborative leadership and research ethics. She is the Kenya PI of the current our adult SMART study For HIV (ADAPT-R) study and PI of the FACES program. **Zachary Kwena, PhD, MA** is a KEMRI Research Scientist with extensive experience in conducting qualitative research with vulnerable populations with HIV, including young women and street youth, in Kisumu, Kenya. **Eliud Akama, MPH** is Research scientist in KEMRI with broad research experience and has led large HIV research and programmatic projects. **Starley Shade, PhD, MPH**, is an Associate Professor of Epidemiology and Biostatistics at UCSF, and an expert in economic evaluation of HIV-related health systems interventions. **Lina Montoya,** PhD, is a Postdoctoral Researcher at University of North Carolina, Chapel Hill, with expertise in research on causal inference and (optimal) dynamic treatment regimes. The breadth of expertise and experience along with a strong track record of collaboration position this team to successfully execute this study. .Please see their biosketches in Appendix A.

| Name | Title | Role |
| --- | --- | --- |
| Elizabeth Bukusi, MBChB, M.Med, MPH, PhD, PGD  **KEMRI** | Site Principal Investigator | She will lead the study in Kenya, ensuring the study is carried out on time, within budget and to high quality standards. Shewill be responsible for managing study field operations in Kenya, including oversight of regulatory, logistics, implementation, and administration. She will provide critical expertise on ethical considerations, collaborative management, policy and study implementation. |
| Elvin Geng, MD, MPH  **Washington University** | Multiple Principal Investigator | He will co-lead the overall study with his role drawing on his expertise in methods and engagement in HIV care to lead the design of the sequential randomization, the implementation of study interventions (drawing from previous experience with SMART implementation), the data collection plan, database creation and maintenance. |
| Lisa Abuogi, MD, MS  **University of Colorado, Denver** | Multiple Principal Investigator | She will co-lead the overall study with her role drawing on her experience with adolescents and peer-based interventions to refine study interventions, train and oversee research staff, supervise intervention application in the field, and measurement of fidelity and client experience. |
| Zachary Kwena, PhD  **KEMRI** | Co-Investigator | He will oversee qualitative work with adolescents to tailor interventions and obtain participant intervention experiences. |
| Eliud Akama  BScN, MPH | Co-Investigator | He will be responsible for overseeing the day-to-day study activities, managing the study staff, tracking study progress, and will communicate closely with the KEMRI investigators and MPIs. He will be responsible for facilitating study training, managing the local regulatory submissions, and ensuring for protocol compliance |
| Starley Shade, MPH, PhD  **University of California San Francisco** | Co-Investigator | She will guide the cost-effectiveness component of the study. |
| Lina Montoya, PhD  *Post Doctoral Researcher*  *University of North Carolina at Chapel Hill* | Co-Investigator | She will be the trial statistician, carrying out and/or leading analyses including (but not limited to) power analyses, exploratory analyses, primary analyses, and secondary analyses. |

# 16.0 APPENDICES

Appendix A: Biosketches and CITI of each investigator

Dr. Elvin Geng

Dr. Lisa Abuogi

Dr. Elizabeth Bukusi

Dr. Starley Shade

Eliud Akama

Dr. Lina Montoya

LINA MARIA MONTOYA

**Phone**: +1 (410) 585-7470

**Email**: [lmontoya@unc.edu](mailto:lmontoya@unc.edu)

**Website**: lina-montoya.com

**Research interests**: causal inference, data science, (optimal) dynamic treatment regimes, precision medicine/public health, adaptive designs, impact evaluation, United States criminal justice reform, socioeconomic development in Colombia

**EDUCATION & FELLOWSHIPS**

# Post-doctoral Fellowship 2021 - present

**University of North Carolina, Chapel Hill, Biostatistics**

Advisor: Michael Kosorok, PhD

# PhD University of California, Berkeley, Biostatistics 2017 - 2020

Dissertation: “Estimation and Evaluation of the Optimal Dynamic Treatment Rule: Practical Considerations, Performance Illustrations, and Application to Criminal Justice Interventions”

Advisor: Maya Petersen, MD PhD

# MA University of California, Berkeley, Biostatistics 2014 - 2017

# BA Johns Hopkins University, Psychology 2008 - 2012

Graduated with General Honors

Minor in Applied Mathematics and Statistics

**GRANTS & AWARDS**

# National Science Foundation travel grant 2022

**Ruth L. Kirschstein Predoctoral Individual National Research Service Award 2019 - 2021**

National Institute of Allergy and Infectious Diseases, National Institutes of Health (NIH)

Title: “Personalized strategies for HIV treatment maintenance: an application of novel machine learning methods to HIV care in East Africa” (F31AI140962)

Sponsors: Maya Petersen, MD PhD; Elvin Geng, MD; Mark van der Laan, PhD

| **American Society of Clinical Psychopharmacology New Investigator Award** | **2020** |
| --- | --- |
| **UC Berkeley Biostatistics Graduate Division Summer Grant** | **2018** |
| **Reshetko Family Scholarship in honor of Chin Long Chiang** | **2017** |
| **UC Berkeley Outstanding Graduate Student Instructor Award** | **2016** |
| **Johns Hopkins University Julian C. Stanley Award** | **2012** |
| **RESEARCH** |  |
| **University of North Carolina, Chapel Hill**  **Co-Investigator**, Advisors: Elvin Geng, MD and Maya Petersen, MD PhD | **2022 - present** |

Lead analyst for the Sequential Multiple Assignment Randomized Trial (SMART) called An Adaptive Strategy for Preventing and Treating Lapses of Retention in HIV Care for Adolescents (A4A; NCT04432571).

# University of California, Berkeley 2016 - present

**Post-doctoral researcher (formerly Graduate Student Researcher)**, Advisor: Jennifer Skeem, PhD

Biostatistician for projects aimed at reducing recidivism in the United States criminal justice system. Application of point-treatment and longitudinal causal inference estimation techniques to study: (1) individualized interventions for reducing recidivism among adult offenders with mental illness; (2) the effect of specialty versus traditional probation on probationers’ future re-arrest and violence; (3) the effect of reporting to a researcher versus clinician on high-risk patients’ self-perceived violence and self-harm.

# University of California, Berkeley 2017 - 2020

**Graduate Student Researcher**, Advisor: Maya Petersen, MD PhD

Lead analyst for the SMART called Adaptive Strategies for Preventing and Treating Lapses of Retention in HIV Care (ADAPT-R; NCT02338739). Primary roles: (1) leading the execution of primary analyses; (2) developing point-treatment and sequential individualized (optimal dynamic) treatment rules to increase HIV treatment retention in rural Kenya.

# Universidad Nacional de Colombia (Bogotá, Colombia) 2017 - 2020

**Research Volunteer,** Advisor: Piedad Urdinola, PhD

Biostatistician for project leveraging data from the Insituto Nacional de Salud (Colombia) to predict incidence of domestic violence in the country using machine learning.

# Boston Children’s Hospital/Harvard Medical School, Labs of Cognitive Neuroscience 2012 - 2014

**Research Assistant**, Advisor: Charles A. Nelson, PhD

Collected, managed, and analyzed electrophysiology, electroencephalography (EEG), near-infrared spectroscopy, galvanic skin response, electrocardiogram, eye-tracking, and genetic data to study the development and neural bases of emotion processing in infancy and early childhood.

# Johns Hopkins University 2011 - 2012

**Research Assistant**, Advisors: Lisa Feigenson, PhD and Melissa Libertus, PhD

Collected and analyzed data on numerical development; specifically, visual habituation experiments with: (1) 6- month-old infants on numerical discrimination tasks; (2) 4- to 7-year-old children’s on working memory development.

**National Institutes of Health (National Institutes on Drug Abuse) 2006, 2007, 2010 Research Assistant**, Advisor: Marisela Morales, PhD

Implemented basic wet laboratory techniques (e.g., cellular staining, imaging, perfusions) and studied neuronal pathways driving the neurobiology of drug addiction.

**TEACHING**

# Short Course Co-Instructor 2020 – 2022

- *Causal inference for multiple time-point (longitudinal) exposures.* This short course applies the Causal Roadmap to estimate the causal effects with multiple intervention variables, such as the cumulative effect of an exposure over time, controlled direct effects, and effects on survival-type outcomes with right-censoring.
- Co-instructor for the following:
  - Atlantic Causal Inference Conference (May 2022)
  - Society of Epidemiological Research Conference Workshop (December 2020, June 2021, May 2022, June 2022)
  - American Statistical Association Webinar (April 2021)

# University of North Carolina, Chapel Hill

**Postdoctoral Fellow Assistant 2021**

- *Precision Medicine* (Bios 740), Fall 2021
  - Guest lecturer on topics in Precision Medicine, including an introduction to Causal Inference and an introduction to SMART trials
  - Graded assignments, including written assignments on deep learning and SMART design proposals

# University of California, Berkeley 2014 - 2018

**Graduate Student Instructor (GSI)**

- *Introduction to Causal Inference* (PH 252D), Spring 2017 and 2018
  - Taught computer labs related to causal inference estimation in R
  - Held weekly office hours and supplementary sections
  - Led class discussions and graded assignments, labs, and final projects
  - Average GSI Rating: 6.94/7
- *Advanced Topics in Causal Inference* (PH 252E), Fall 2017
  - Designed, wrote, and taught computer labs related to longitudinal causal inference estimation and simulations in R
  - Held weekly office hours and graded assignments, labs, and final projects
  - Average GSI Rating: 6.86/7
- *Statistical Analysis of Continuous Outcome Data* (PH 145), Fall 2015 and 2016
  - Assisted in the design of course structure and materials
  - Taught weekly computer labs related to statistical analysis and computer programming in R
  - Held weekly office hours and graded projects, exams, and weekly assignments
  - Average GSI Rating: 6.66/7
- *Modeling the Dynamics of Infectious Disease Processes* (PH 252B), Spring 2016
  - Assisted with weekly computer labs related to infectious diseases, dynamical systems, and computer programming in Berkeley Madonna and R
  - Held weekly office hours and graded assignments
  - Average GSI Rating: 6.83/7
- *Introduction to Probability and Statistics in Biology and Public Health* (PH 142/141), Fall 2014, Spring 2015, and Summer 2015
  - Taught weekly supplemental sections and Stata computer labs
  - Held weekly office hours and graded exams quizzes, and weekly assignments
  - Average GSI Rating: 6.50/7

# University of California, Berkeley 2015 - 2016

**Student Learning Center Tutor**

Tutored students individually and in small groups at Math/Statistics drop-in tutoring for classes such as *Concepts of Probability* (STAT 134), *Concepts in Computing with Data* (STAT 133), *Introduction to Probability and Statistics* (STAT 20)*,* and *Introduction to Statistics* (STAT 2).

# San Quentin Prison and University of California, Berkeley 2016

**Tutor**

Tutored incarcerated men at San Quentin Prison through Teach in Prison “DeCal” class on phonics, writing, high school math, and GED preparation.

# Johns Hopkins University 2011

**Teaching Assistant for *Psychopharmacology* (AS PSYC 200.376)**

Held office hours and assisted with grading.

**PUBLICATIONS**

**Montoya, L.,** Skeem, J. L., Lowenkamp, C. (in preparation). Comparing Risk Assessment Instrument Decision- Making in Federal Pretrial Recommendations versus the Status Quo.

Skeem, J. L., **Montoya, L.,** Lowenkamp, C. (in preparation). Understanding Racial Disparities in Federal Pretrial Detention Recommendations to Inform Policy Solutions.

**Montoya, L.,** Kosorok, M., Geng, E., Schwab, J., Odeny, T., Petersen, M. (submitted). Efficient and Robust Approaches for Analysis of SMARTs: Illustration using the ADAPT-R Trial.

Geng, E.H., Odeny, T., **Montoya, L.**, Iguna, S., Kulzer, J., Adhiambo, F., Eshun-Wilson, I., Akama, E., Nyandieka, E., Guze, M., Shade, S., Packel, L., Camlin, C., Thirumurthy, H., Bukusi, E., Petersen, M. (submitted). Adaptive Strategies for Engagement in HIV Treatment: A Sequential Multiple Assignment Randomized Trial among Persons Living with HIV in Kenya.

**Montoya, L.,** van der Laan, M., Skeem, J.L., Petersen, M. (2022). Performance and Application of Estimators for the Value of an Optimal Dynamic Treatment Rule. *International Journal of Biostatistics*.

**Montoya, L.**, van der Laan, M., Luedtke, A., Skeem, J.L., Coyle, J., Petersen, M. (2022). The Optimal Dynamic Treatment Rule SuperLearner: Considerations, Performance, and Application. *International Journal of Biostatistics*.

Pokaprakarn, T., Prieto, Juan., Price, J., Kasaro, M., Sindano, N., Shah, H., Peterson, M., Akapelwa, M., Pailya, F., Sebastião, Y., Goodnight, W., Stringer, E., Freeman, B., **Montoya, L.**, Chi, B., Rouse, D., Cole, S., Vwalika, B., Kosorok, M., Stringer, J. (2022). AI Estimation of Gestational Age from Blind Ultrasound Sweeps in Low- Resource Settings. *New England Journal of Medicine Evidence*.

Iguna, S., Getahun, M., Lewis-Julzer, J., Odhiambo, G., Adhiambo, F., **Montoya, L.**, Petersen, M., Bukusi, E., Odeny, T., Geng, E., Camlin, S. (2022). Attitudes Towards and Experiences with Economic Incentives for Engagement in HIV Care and Treatment: Qualitative Insights from a Randomized Trial in Kenya. *PLOS Global Public Health*.

Dickerson, K.L., Quas, J., **Montoya, L.**, Skeem, J.L. (2020). A Randomized Controlled Trial of Positive Emotion Training with Maltreated Youth: Reducing Anger Bias and Aggression. *Clinical Psychological Science*.

Skeem, J.L., **Montoya, L.**, Manchak, S. (2018). Comparing costs of traditional vs specialty probation for people with serious mental illness. *Psychiatric Services*.

Velez, M.L., McConnell, K., Spencer, Nancy., **Montoya, L.**, Tuten, M., Jansson, L.M. (2018). Prenatal buprenorphine exposure and neonatal neurobehavioral functioning. *Early Human Development*.

Skeem, J.L., Manchak, S., **Montoya, L.** (2017). Comparing public safety outcomes for traditional probation vs specialty mental health probation. *JAMA Psychiatry*.

**Montoya, L.**, Westerlund, A., Troller-Renfree, S., Righi, G., Nelson, C. (2015). The effect of heterogeneous race exposure during infancy. *Cognitive Development.*

Vanderwert, R.E., Westerlund, A., **Montoya, L.**, McCormick, S.A., Miguel, H.O., Nelson, C.A. (2014). Looking to the eyes influences the processing of emotion on face-sensitive event-related potentials in 7-month-old infants. *Developmental Neurobiology*.

**INVITED LECTURES & RESEARCH COLLOQUIUM**

- Answering Causal Questions with SMART data: Illustration using the ADAPT-R Trial

o 2022

- Causal Inference Research Group (CIRG). University of North Carolina, Chapel Hill.

o 2021

- Center for Targeted Machine Learning and Causal Inference - Adaptive Designs Working Group. University of California, Berkeley.
- SuperLearner-based Optimal Dynamic Treatment Rule Estimation and Evaluation with Application to Criminal Justice Interventions in the United States

o 2021

- Computational Social Science Forum. University of California, Berkeley.
- University of North Carolina, Chapel Hill Biostatistics Departmental Seminar. University of North Carolina, Chapel Hill.

o 2020

- Advanced Topics in Causal Inference (252E). University of California, Berkeley.
- Biostatistics Seminar Series. Department of Biostatistics & Epidemiology at University of Massachusetts, Amherst.
- Causal Inference Reading Group. Department of Statistics of University of California, Berkeley.

o 2019

- Causal Inference Working Group. Department of Biostatistics at Johns Hopkins School of Public Health.
- Biomedical Big Data Seminar. Division of Biostatistics at University of California, Berkeley.
- Analysis of Parameters Under Optimal Dynamic Treatment Rule-Based Adaptive Designs

o 2019

- Petersen/Balzer Biostatistics Lab Meeting. University of California, Berkeley.
- Reglas (Óptimas) para Asignar Tratamiento con Aplicación al Estudio ADAPT-R en Kenia

o 2019

- Statistics Seminar. Department of Statistics at Universidad Nacional – sede Bogotá.
- SuperLearner-based Optimal Dynamic Treatment Rule Estimation and Evaluation with Application to HIV Care in Zambia

o 2019

- Petersen/Balzer Biostatistics Lab Meeting. University of California, Berkeley.
- Applications of (Optimal) Dynamic Treatment Rule Estimation and Evaluation to HIV Prevention in East Africa

o 2018

- Implementation Science Lab Group. University of California, San Francisco.
- Advanced Topics in Causal Inference (252E). University of California, Berkeley.

**SELECTED CONFERENCE PRESENTATIONS**

**Montoya, L.** (2022). Applying the Causal Roadmap in Precision Medicine: Speed Bumps and Detours. Symposium presentation at the *Society for Epidemiological Research.*

**Montoya, L.** (2021). Estimating Effects from a SMART with Application to the ADAPT-R Trial. Symposium presentation at the *Society for Epidemiological Research*.

**Montoya, L.** (2021). Optimal Dynamic Treatment Rule Estimation and Evaluation with Application to Criminal Justice Interventions in the United States. Workshop presentation at the *International Conference on Machine Learning*.

**Montoya, L.**, Petersen, M., Farrell, S., Skeem, J. (2020, 2021). Estimating Individualized Treatment Rules for Reducing Recidivism Among Criminal Justice-Involved Adults with Mental Illness. Virtual poster presented at *American Society of Clinical Psychopharmacology*.

**Montoya, L.**, Petersen, M., Farrell, S., Skeem, J. (2020). Estimating Individualized Treatment Rules for Reducing Recidivism Among Criminal Justice-Involved Adults with Mental Illness. Virtual poster presented at *American Psychological Association*.

**Montoya, L.,** van der Laan, M., Petersen, M. (accepted 2020, presentation postponed due to COVID-19). Performance and Application of Estimators for the Value of an Optimal Dynamic Treatment Rule. Poster for *Atlantic Causal Inference Conference*, Montreal, Canada.

**Montoya, L.,** Chen, D., Sikombe, K., Eshun-Wilson, I., Holmes, C., Sikazwe, I., Beres, L.K., Mukambda, N., Simbeza, S., Moore, C., Somwe, P., Mody, A., Pry, J., Geng., E., Petersen, M. (accepted 2020, presentation postponed due to COVID-19). Resource-Constrained Optimal Dynamic Treatment Rule Estimation with Applications to HIV Care in Zambia. Poster for *Society for Epidemiologic Research*.

**Montoya, L.**, Roose, J., van der Laan, M. (2019). Comparison of Parameter Estimates from Optimal Dynamic Treatment Rule-Based Adaptive Designs. Poster presented at *Joint Statistical Meetings*, Denver, CO.

**Montoya, L.**, Petersen, M., van der Laan, M. (2019). Performance of “SuperLearner”-based Optimal Dynamic Treatment Rule Estimation. Poster presented at *Atlantic Causal Inference Conference*, Montreal, Canada.

**Montoya, L.**, Skeem, J.L. (2017). Do Patients’ Minimize Self-Perceived Levels of Risk When Talking to Clinicians Rather than Researchers? Paper talk at *American Psychology Law Society*, Seattle, WA.

**Montoya, L.,** Westerlund, A., McCormick, S., Nelson, C. (2014). Discrimination of species-specific emotions across development: an ERP study. Poster presented at the *Cognitive Neuroscience Society Annual Meeting*, Boston, MA.

**Montoya, L.,** Libertus, M., Feigenson, L. (2011). Infants' ability to track numerosity frequencies. Poster presented at the *Johns Hopkins University Summer Internship Poster Session*, Baltimore, MD.

**Montoya, L.,** Ng, T., Morales, M. (2010). Detection of dopaminergic markers in glutamatergic neurons within the A10 region of the midbrain dopamine system. Poster presented at the *National Institutes of Health Summer Internship Poster Session*, Bethesda MD and the *Johns Hopkins Bayview Medical Center Poster Session*, Baltimore, MD.

**Montoya, L.,** Hein, K., Morales, M. (2007). Expression of vesicular glutamate transporter 2 mRNA in the ventral tegmental area neurons of methamphetamine rats. Poster presented at the *National Institutes of Health Summer Internship Poster Session*, Bethesda, MD and the *Johns Hopkins Bayview Medical Center Poster Session*, Baltimore, MD.

**SERVICE & ACTIVITIES**

# Reviewer

- 2022 Atlantic Causal Inference Conference (ACIC) poster reviewer
- National Institutes of Health grant reviewer:
  - Small Business Innovation Research (R43/R44) for National Institute on Drug Abuse. August 2019, June 2020.
- Ad Hoc Journal Reviewer: *Journal of the American Statistical Association, The Annals of Applied Statistics, Statistical Methods in Medical Research, American Journal of Epidemiology, Journal of Causal Inference, Epidemiologic Reviews, Drug and Alcohol Dependence*

# Media

- “Applying the Causal Roadmap to Optimal Dynamic Treatment Rules with Lina Montoya.” Podcast interview on *This Week in Machine Learning (TWIML)*. https://twimlai.com/applying-the-causal-roadmap- to-optimal-dynamic-treatment-rules-with-lina-montoya/

| **Activities** |  |
| --- | --- |
| Co-leader, Precision Health and AI Research (PHAIR) Kosorok Lab | 2021 - present |
| Certified Mental Health First Aider | 2021 |
| Co-founder, Biostatistics Anti-Racism Group | 2020 |
| Colombians at Berkeley Group | 2017 - 2020 |
| Biostatistics Graduate Student Association | 2016 |
| Childhood, Health, and Society Intersession Program, Makerere University (Kampala, Uganda) | 2012 |
| Semester study abroad, Universitat de Barcelona (Barcelona, Spain) | 2011 |
| Music theory and performance, Peabody Conservatory and Howard Community College | 1995 - 2010 |

# Statistical consultant

- Consultant for Masters in Public Health (MPH) seminar at UC Berkeley (PB HLTH 292) offering statistical guidance for students in the MPH program completing their theses
- Statistical consulting class (STAT 272) offering free statistical consulting services for members of the UC Berkeley community
- Associated Students University of California (ASUC) department of Mental Health and Wellness - analysis of Cal Student Assessment on Mental Health survey of 7,000 undergraduate students about their well-being and access to mental health/wellness resources
- Consultant for dissertation analyses of PsyD students at The Wright Institute

**LANGUAGES**

**English**: Fluent

**Spanish**: Fluent

**COMPUTING SKILLS**

**Programming**: R, Python, Stata, Berkeley Madonna, SPSS, MATLAB, Java

**Github:** github.com/lmmontoya

# R packages:

- “SL.ODTR”. Estimates (using SuperLearner) and evaluates (using CV-TMLE, TMLE, IPTW, and G- computation estimator) the optimal dynamic treatment rule.

**OTHER**

Proficient in reading music and performing piano, guitar, and percussion Dual citizenship United States and Colombia

Appendix B-Data Collection Tools

B1 Disclosure screening script, version 2019.10.17

B2 Eligibility screening form, version 2019.10.17

B3 Locator form, version 2019.10.17

B4 Routine medical record ‘Greencard, MOH 257’

B5 Routine viral load request form

B6 Routine discontinuation form

B7 Cause of death form (verbal autopsy), version 2019.09.16

B8 Socio Demographic survey (Socio-demo) for adolescents, version 2019.10.17

B9 General Health Assessment for Children (GHAC) survey for adolescents, 2019.10.17

B10 Enhanced retroactive review of preganancy (ERR-Preg), version 2019.10.17

B11 Individually Focused Food Insecurity Access Scale (IFIAS), version 2019.10.21

B12 Comprehensive ART Adherence Measurement for Paediatrics (CAMP), version 2019.10.17

B13 Hojat Satisfaction Scale, version 2019.10.17

B14 Patient Health Questionnaire-9 (PHQ-9), version 2019.10.17

B15 General Anxiety Disorder (GAD-2) screener

B16 General Anxiety Disorder (GAD-7)

B17 Adverse Childhood Experiences (ACES-IQ)

B18 Alcohol Use Disorders Identification Test (AUDIT) and drug use, version 2019.10.17

B19.1 Lost to Follow Up (LTFU) questionnaire (tracing and barriers), version 2019.10.17

B19.2 Outcome Ascertain form, version 2018.07.26

B20 Study withdrawal form, version 2019.10.17

B21 Intervention experience survey, version 2019.10.17

B22 Costing – Time and Motion tool

B23 Costing – Micro-costing tool

B24 FGD guide – English, version 2020.06.22

B25 FGD guide –Kiswahili, version 2020.06.22

B26 FGD guide – Dholuo, version 2020.06.22

B27 IDI guide – English, version 2019.10.17

B28 IDI guide – Kiswahili, version 2019.10.17

B29 IDI guide – Dholuo, version 2019.10.17

B30 Peer Navigation Interview & FGD guide – English version 2023.07.31

B31

B32

B33 Peer Navigation Interview & FGD Recruitment Script – English version 2023.07.31

B34

B35

B36 DCE Data Collection tool-English, Kiswahili and Dholuo Version 2020.06.22

B37. Extended outcome investigation and classification form Version 2.0 2022.01.18

**Appendix B1_A4A Screening and Disclosure Form**

Date: Time:

Screening ID:

PID:

Age:

Sex:

**1. Disclosure Screening**

If <18 years old, when talking to caregivers use “your adolescent” instead of “you”.

**Recruitment Script (Part 1)**

**My name is …………’**

“I would like to ask you a few questions to see if you might be eligible for a research study. I will take less than five minutes of your time. The Kenya Medical Research Institute (KEMRI) and Washington University are conducting a study examining adolescent engagement in HIV care. The study is led by Drs. Elvin Geng Lisa Abuogi, and Dr. Elizabeth Bukusi.

The questions I will ask are about your clinic visits and health. May I go ahead and ask you the questions? You have the right to choose to answer or not answer these questions.”

**If agrees or disagrees to be screened**

If the patient says “no” - “Thank you for your time today”.

If the patient says “yes” - Proceed with disclosure and eligibility screening.

**Disclosure Screening Questions**

If adolescent is below 18 years old,

Ask caregiver; Has the child been disclosed to? Yes□ No□

(If Yes, proceed to disclosure screening questions. If no, offer to assist with disclosure and postpone screening to the next visit/contact with the participant.)

If adolescent is above 18 years old, proceed with screening to determine eligibility.

| **DO NOT READ OPTIONS, THIS IS AN OPEN-ENDED QUESTION**  1. Why do you come for visits at the clinic?  □ HIV □ Other illness □ Does not know □ Other: | |
| --- | --- |
| **DO NOT READ OPTIONS, THIS IS AN OPEN-ENDED QUESTION**  2. Do you know what your illness is called?  □ HIV □ Other illness □ Does not know □ Other: | |
| **DO NOT READ OPTIONS, THIS IS AN OPEN-ENDED QUESTION**  3. Why do you have to take medication?  □ HIV □ Other illness □ To stay healthy □ Does not know  □ I wonder about this □ Other: | |
| ***If the answers to all questions have been “does not know” or “other”, may need to consider child NOT DISCLOSED to and abandon further questions and go to reason not eligible and tick “Not yet disclosed to”. If you think the child KNOWS their status, then proceed to next questions – which will make it clear that you are talking about HIV.*** | |
| 4. Have you been told that your illness is HIV? | □ Yes □ No |
| 5. Before you knew that you had HIV, did you ask questions about why you had to take medication? | □ Yes □ No □ Do not know |
| 6. Do you still have questions about why you have to take medication? | □ Yes □ No □ Do not know |

If the adolescent has been disclosed to and is aware of his/her HIV status, proceed to the **Recruitment script, part 2** for other screening questions below;

If <18 years old, when talking to caregivers use “your adolescent” instead of “you”.

**Recruitment script, part 2 (other screening)**

“I would like to provide you with a little more information about the study. The purpose of this study is to find out which strategies best help adolescents and young adults stay in care and take their medications well. Retention in HIV care and treatment and viral load suppression are important for healthy outcomes. However, a number of patients are lost to follow-up after enrolment and some have high viral loads. In this study, we are trying to learn how to give adolescents the strategy that works best for them and at the time when it will be most helpful. I have a few more questions about your age, where you live, moving plans, phone access, and other study involvement. May I go ahead and ask you the questions? You have the right to choose to answer or not answer these questions.

**If agrees or disagrees to be screened with other eligibility questions**

If the patient says “no” - “Thank you for your time today”.

If the patient says “yes” - Proceed with other screening questions.

**a. Other eligibility screening questions**

Are you a resident in the area?

Do you have plans of moving out the area?

Do you have access to a phone? If yes, proceed to the phone access screening algorithm **(Appendix 1 below)**

Have you disclosed your HIV status to the person you are sharing phone with?

Determine if;

the caregiver has consented or not

the participant was in former ADAPT-1 study by counter checking the ADAPT-1 sticker on the patient’s file

the participant is enrolled in another study

the participant is acutely ill and requiring hospitalization

If participant is not eligible, explain to the participant why he/she is not eligible. (Proceed to b)

b. Why is the patient not eligible? (Check all that apply)

Not resident in the area

Plans to move out of the area

Shares phone but has not disclosed to the person he/she is sharing with

Absence or lack of consent from caregiver

Not yet disclosed to

Former ADAPT-1 Participant

Enrolled in other study

Acutely ill and requiring hospitalization

If 14-17 years old:

1. Does the parent/caregiver consent for his/her child to enroll in the study (Yes/No/NA)
2. Does the patient assent to enroll in the study (Yes/No/NA)

If 18 years old and above:

a) Does the patient consent to enroll in the study (Yes/No/NA)

Complete the paper-based screening and disclosure form, study screening log and enter the screening details in ODK

**Appendix 1**

Have you disclosed your HIV status to the person you share a phone with?

Are you enrolled in or attend boarding school?

Y

N

Do you have a trusted person (eg. matron/teacher/nurse) who will allow you to use their phone twice monthly for up to 15-20 minutes?

Yy

N

Proceed to enrollment.

Randomize to

e-Nav/SOC

Proceed to enrollment.

Put in SOC

Do you have your own phone or access to a shared a phone?

Own phoneeye

No

phone

Shared phone

Proceed to enrollment.

Randomize to

e-Nav/SOC

Yy

Ny

Proceed to enrollment.

Put in SOC

Proceed to enrollment.

Randomize to

e-Nav/SOC

Proceed to enrollment.

Put in SOC

Note: those randomized to e-NAV/SOC and those put in SOC are both eligible for rerandomization

**Appendix B8**

**A4A – Socio-Demographic Questionnaire**

| **COMPLETE ALL OF THE INFORMATION ON THIS PAGE BEFORE BEGINNING THE INTERVIEW.** | | | |
| --- | --- | --- | --- |
| **SD-A-ID** | Study subject identification number | □□□-□□□ | |
| **SD-A-SITE** | Study site | Site 1  Site 2  Site 3  Site 4  Site 5  Site 6 | 1  2  3  4  5  6 |
| **SD-A-ENGAGE** | Engagement status  (of participant) | Retained in care  Lost to follow up | 1  2 |
| **SD-A-SEX** | Study subject sex | Male  Female | 1  2 |
| **SD-A-LANG** | Language of the interview | English  Kiswahili  Dholuo | 1  2  3 |
| **SD-A-DATE** | Date of assessment | DAY MONTH YEAR | |
| **SD-A-RA** | Research assistant initials |  | |

*INSTRUCTIONS:* *Interviewer instructions are in italic print—these are for your use and should not be read aloud. If necessary, you may read the options of questions to prompt the respondent. Read all of the questions and bolded non-italic print aloud to the respondent. Please read all information as it is written. Do not read aloud the answer options of “Don’t know” or “Refused”. Only circle the number next to the option “Don’t know” if the respondent states he/she doesn’t know. Only circle the number next to the “Refused” option if the respondent states he/she does not want to answer. For questions that require you to write an answer rather than circle a response category on the form, or when respondent answers “Other” and provides his/her own answer, please record respondent’s comments IN CAPITAL LETTERS as directly and carefully as possible.*

*INTRODUCTION:* **Thank you again for agreeing to participate in our study. Some of the questions I will ask are about private things and it is possible that they may make you feel shy or uncomfortable. You can choose not to answer any questions that you do not want to. You may also ask me to explain questions if you do not understand them. You may also stop the interview at any time if you decide you no longer want to participate. Please remember that your responses to our questions are identified only by number and will be kept confidential. Your name does not appear on this questionnaire.**

| **No.** | **QUESTIONS & INSTRUCTIONS** | **RESPONSES** |  | **SKIPS** |
| --- | --- | --- | --- | --- |
| ***DEMOGRAPHICS***  **Let us start with a few background questions about you.** | | | | |
|  | How old are you today?  *Enter response in years. If respondent answers “don’t know”, probe to nearest 5 years. If s/he refuses to answer, enter 99. If the respondent gives the year of birth, calculate the age and enter. If the participant provides only the date of birth, enter the information in the space provided and calculate the age after the interview.* | Years  DIA MÊS ANO  *DATE OF BIRTH*  DAY MONTH YEAR |  |  |
|  | Are you currently attending school? | Yes  No  *Don’t know*  *Refused* | 1  2  *88*  *99* | ***If 1, 88,99*** *🡪* ***Q4*** |
|  | What is the main reason you are not attending school? (Primary,Secondary,College/University) | I have been sick  I don’t like school  I have to look after family members  There’s not enough money  School is too far away  I am working  I have completed my education*.*  *Don’t know*  *Refused* | 1  2  3  4  5  6  *7*  *88*  *99* |  |
|  | What is the highest level of formal schooling you have reached?  *Do not read options aloud. Circle one response.* | None  Primary school (1^st^ – 8^th^ class)  Secondary school (Form 1-4)  Pre University, colleges, technical and vocational training institutions  University  *Don’t know*  *Refused* | 1  2  3  4  5  *88*  *99* |  |
|  | Are you currently in a relationship with someone? This may be a spouse or someone you call your partner. | Yes  No  Don’t know  *Refused* | 1  2  *88*  *99* | **If 2 🡪 Q6** |
|  | (*Ask if in a relationship with someone*),  How many partners/spouses? |  | 1  2  3  4  5  88  99 |  |
|  | What is your relationship with your partner? *(If more than one spouse/partner, probe for relationship with ‘main partner’)* | Married, living together  Married, living apart  Living together, not married  Not living together  Separated/divorced  Widowed  *Don’t know*  *Refused* | 1  2  3  4  *5*  *6*  *88*  *99* |  |
| 1. ~~1a~~ | What is the main activity that occupies your day?  *Do not read options aloud. Circle one response.* | Student  Not employed  Housewife  Farmer  Seller (e.g. market, streets)  Househelp (e.g. cleaner, cook, nanny)  Professional (e.g. teacher, nurse)  Hairdresser/beautician  Guard/security  Construction worker  Driver  Other______________________  *Don´t know*  *Refused* | 1  2  3  4  5  6  7 8  9  10  11  12  *88*  *99* |  |
| **CHILD-CAREGIVER RELATIONSHIP AND HOUSEHOLD STRUCTURE**  **Now I’d like to ask some questions about your household.** | | | | |
|  | What is your birth order in your family? | 1^st^ born  2^nd^ born  3^rd^ born  4^th^ born  5^th^ born  Last born  Other (specify ___)  *Specify the birth order number if not 1-5 or last born*  Don’t know  Refused | 1  2  3  4  5  6  7  88  99 |  |
|  | What best describes your current living situation?  *Allow respondent to describe and RA to code appropriate response. Do not read options aloud.* | Household with immediate family members (may include extended family members)  Household with extended family members (but no immediate family members)  Household with non-family members  Institution/Boarding school, specify:_______________  Homeless / street living  *Don’t know*  *Refused* | 12  3  4  5  *6*  *7*  *88*  *99* |  |
|  | Who takes most responsibility for your well-being and care? | Self  Mother  Father  Aunt/Uncle  Grandparent  Sibling  Partner  Other relative  Guardian, non-relative  *Don’t know*  *Refused* | 1  2  3  4  5  6  7  8  *9*  *88*  *99* | ***If 1, 88,99*** *🡪* ***Q12*** |
|  | Does she/he live in the same household as you? | Yes  No  *Don’t know*  *Refused* | 1  2  *88*  *99* |  |
|  | Including you if you are over 18, how many adults >18 years live in your household right now? | Adults  *Don’t know*  *Refused* | 88  99 |  |
|  | Including you if you are under 18, how many children ≤18 years live in your household right now? | Children  *Don’t know*  *Refused* | *88*  *99* |  |
|  | In an average month, about how much income and other earnings does your household make (combining all sources of income)?  *If the participant needs help, read options aloud. Circle one response. Enter amount in the space provided. If respondent answers “Don’t know”, enter 88. If respondent refuses to answer, enter 99.* | Less than 1000 Kshs  1000 – 3000 Kshs  4000 – 8000 Kshs  8000 to 15000 Kshs  More than 15000 Kshs  *Don’t know*  *Refused* | 1  2  3  4  5  *88*  *99* |  |
|  | In the last 12 months, how often did your household not have enough food?  *Circle one response.* | Never  Sometimes  Always  *Don’t know*  *Refused* | 1  2  3  *88*  *99* |  |
|  | What is the main source of drinking water for your household?  *Read options aloud. Circle one response.* | Public dug well  Private dug well  Public tap / standpipe  Piped water (tap) directly in residence  Other, _____________________  *Don’t know*  *Refused* | 1  2  3  4  5  *88*  *99* |  |
|  | Do you use electricity at home?  *Circle one response.* | Yes  No  *Don’t know*  *Refused* | 1  2  *88*  *99* |  |
|  | Do you have a mobile phone that you consider your own?  *Circle one response.* | Yes  No  *Don’t know*  *Refused* | 1  2  *88*  *99* | *If yes, Q23* |
|  | *If no to mobile phone of own: D*o you share a mobile phone or have access to a shared mobile phone?  *Circle one response* | Yes  No  *Don’t know*  *Refused* | 1  2  *88*  *99* |  |
| ***SEXUAL HISTORY AND RELATIONSHIP***  **You may find the next set of questions personal and sensitive. Your honesty is appreciated and please remember that this information is kept confidential. We would like to act you some questions about your relationships and your experiences with sex.** | | | | |
|  | Do you know what sex is?  *Circle one response* | Yes  No  *Don’t know*  *Refused* | 1  2  *88*  *99* |  |
|  | Have you ever had sex?  *Circle one response* | Yes  No  *Don’t know*  *Refused* | 1  2  *88*  *99* | **If 2 🡪 Q34** |
|  | How old were you when you first had sex? | Years  DIA MÊS ANO  *Don’t know*  *Refused* | *88*  *99* |  |
|  | The first time you had sex, was it because you wanted to or because you were forced to?  *Circle one response* | Wanted to  Forced  *Don’t know*  *Refused* | 1  2  *88*  *99* |  |
|  | In total, how many different people have you had sex with? Please give your best guess. | Number  *Don’t know*  *Refused* | *88*  *99* |  |
|  | Did you use a condom the last time you had sex?  *Circle one response* | Yes  No  Don’t remember  *Don’t know*  *Refused* | 1  2  3  *88*  *99* |  |
|  | Are you or your partner currently doing something or using any method to delay or avoid getting pregnant?  *Circle one response* | Yes  No  Do not have a partner/Not applicable  *Don’t know*  *Refused* | 1  2  3  *88*  *99* | **If 2, 3, 88, 99 🡪 Q31** |
|  | If you are using a method to delay or avoid getting pregnant, which method are you or your partner using?  *Circle all that apply.* | IUD/Coil  Injections  Oral Contraceptive Pills  Condom  Natural methods  Withdrawal  Not having sex  Female sterilization  Male sterilization  Other______________  *Don’t know*  *Refused* | 1  2  3  4  5  6  7  8  9  10  *88*  *99* |  |
|  | Are you currently pregnant?  *Circle one response* | Yes  No  *Don’t know*  *Refused* | 1  2  88  99 |  |
|  | Have you ever been pregnant?  *Circle one response* | Yes  No  *Don’t know*  *Refused*  Number | 1  2  *88*  *99* | **If 2 🡪 Q34**  *For females only* |
|  | If you have been pregnant, how many children have you given birth to? | *Don’t know*  *Refused* | *88*  *99* | *For females only* |
|  | How old are your children now? | *Code don’t know 88 or refused 99 per child as needed*  Age  Age  Age  Age  *Add notes here in case figures don’t add up.* |  |  |
|  | Have you ever had a vaccination to prevent cervical cancer (HPV vaccine)?  *Mark ‘yes’ if had at least one dose (can be two doses or three depending on age given)* | *Yes*  *No*  *Don’t know*  *Refused* | 1  2  88  99 | *Ask only females* |
| ***HEALTH STATUS OF HOUSEHOLD MEMBERS***  ***INSTRUCTIONS:***  **The next few questions are about your health and the health of people in your household.** | | | | |
| **32** | To your knowledge, aside from you, who else in your household has been diagnosed with HIV?  *Circle all responses mentioned.*  *Probe:* **‘Anyone else?**’  *If respondent mentions an answer not provided, specify next to Other* | No one  AYA’s father  AYA’s mother  AYA’s siblings  AYA’s other relative(s)  AYA’s Partner/Spouse  Other_______________________  *Don’t know*  *Refused* | 1  2  3  4  5  6  7  88  99 | ***If = 1 🡪 Q34*** |
| **33.** | To your knowledge, aside from you, who in your household is on HIV treatment (ART)?  *Circle all responses mentioned.*  *Probe:* **‘Anyone else?**’  *If respondent mentions an answer not provided, specify next to Other* | No one  AYA’s father  AYA’s mother  AYA’s siblings  AYA’s other relative(s)  AYA’s Partner/Spouse  Other_______________________  *Don’t know*  *Refused* | 1  2  3  4  5  6  7  88  99 |  |
| ***HEALTHCARE AND CLINIC CHARACTERISTICS***  **We would like to ask you some questions about the healthcare clinic that you attend or have attended in the past.** | | | | |
| **34** | Who typically takes you to the clinic for HIV care?  *Circle all responses mentioned.* | Self  Mother  Father  Aunt/Uncle  Grandparent  Sibling  Other relative  Partner/Spouse  Guardian, non-relative  Does not currently attend clinic  *Don’t know*  *Refused* | 1  2  3  4  5  6  7  8  9  10  *88*  *99* |  |
| **35.** | How long does it take you to travel to your clinic for HIV care, one way? Please include the time you spent waiting for transport as well as the time spent actually travelling to the clinic.  **If adolescent does not currently attend clinic, ask about when the child was attending clinic.*  *Enter the amount of time in the space provided. If the time is less than 1 hour, enter 00 and then the number of minutes. If respondent answers “Don’t know”, enter 88. If respondent refuses to answer, enter 99 99.* | HOURS : MINUTES  *Don’t know*  *Refused* | *88*  *99* |  |
| **36.** | Approximately how much does it cost you to travel to this clinic, one way?  **If adolescent does not currently attend clinic, ask about when the child was attending clinic.*  *Enter amount in the space provided. If respondent answers “Don’t know”, enter 8888. If respondent refuses to answer, enter 9999.* | Kenya shillings  *Don’t know*  *Refused* | *88*  *99* |  |
| **37.** | *For AYA lost to follow up:*  Why did you stop coming to the clinic?  *Circle all responses mentioned.*    ***If child is retained in care, do not ask this question and go to next question.* | **Access to care**  Transportation problems  Funds to get to the clinic  Didn’t have enough time for the clinic  Other access to care reasons, (specify ______________)  **Work, School, and Family**  Work interference  School interference w/refill pick up  School interference w/taking treatment  AYA/Caregiver attended funeral  AYA/Caregiver had other family obligation  Partner didn’t want me to attend clinic  Marriage, birth, caregiver change (specify ______________)  Other family/work reason, specify  **Medical**  Felt too sick to come to clinic  Caregiver felt too sick to come to clinic  Side effects from pills  Pill burden  Wanted a break from pills  Pregnancy/gave birth  Other medical reasons, specify:  ___ Hospitalized🡪 Admit date: ______/________/________  DD /MM /YYYY  Hospital name:  Hospitalization Reason:    **Clinic Quality**  The clinic ran out of medications  Transition from adolescent center to the adult clinic  The staff was not nice  The care was not good  Too many admin requirements  The waiting area was uncomfortable  The clinic was too crowded  I have to wait too long during visits  I didn’t want to go to retention class    **Psycho-social**  Felt well, didn’t see need to visit clinic  Attending clinic risked HIV disclosure to someone in community  Afraid clinic would scold me for missing appoint.  Because I went to someone who tried / is trying to cure me by prayer / religious rituals.  Because I saw / am seeing a traditional healer instead  Felt hopeless/depressed  Alcohol consumption  Forgot appointment  Family member or other important person said child should stop going to clinic  *Don’t know*  *Refused* | 1  2  3  4  5  6  7  8  9  10  11  12  13  14  15  16  17  18  19  20  21  22  23  24  25  26  27  28  29  30  30  31  32  33  34  35  36  37  88  99 | ***Applies to only patients lost to care***  ***Multiple select***  ***If child retained in care 🡪***  ***Stigma section*** |

| STIGMA  **The next set of questions is about your thoughts and feelings about how people may treat you if they know you have HIV. Please remember that there are no right or wrong answers and anything you say will be kept private. If any of these questions make you uncomfortable, just let me know and we can skip the question.** | | | | | |
| --- | --- | --- | --- | --- | --- |
| **No.** | **Questions & Instructions** | **Responses** | | **Skips** | **No.** |
|  | How concerned are you that other people in the neighborhood, such as your neighbors, will find out your HIV status without you telling them?  *Read response options aloud. Circle one response.* | Very concerned  A little concerned  Not at all concerned  *Don’t know*  *Refused* | | 1  2  3  *88*  *99* |  |
| **I am going to read a series of statements. For each one, please tell me whether you agree or disagree.** | | **Agree** | **Disagree** | ***Don’t know*** | ***Refused*** |
|  | I have lost friends due to my HIV status. | 1 | 2 | *88* | *99* |
|  | I have been called names, been bullied, or insulted due to my HIV status. | 1 | 2 | *88* | *99* |
|  | I have experienced discrimination at home due to my HIV status. | 1 | 2 | *88* | *99* |
|  | I have experienced discrimination at school due to my HIV status. | 1 | 2 | *88* | *99* |
|  | I have experienced discrimination in the community due to my HIV status. | 1 | 2 | *88* | *99* |
|  | I have experienced discrimination at the clinic due to my HIV status. | 1 | 2 | *88* | *99* |
|  | HIV stigma has made me feel stressed or anxious. | 1 | 2 | *88* | *99* |
|  | HIV stigma has made me feel depressed or sad. | 1 | 2 | *88* | *99* |
|  | I choose not to play with others, spend time with others, or go places due to my HIV status. | 1 | 2 | *88* | *99* |
|  | I feel like it is important to keep my HIV status a secret. | 1 | 2 | *88* | *99* |
|  | My future or hopes for my future have changed in a negative way due to HIV. | 1 | 2 | *88* | *99* |
|  | I sometimes delay taking my medicines so that others will not see me taking them. | 1 | 2 | *88* | *99* |
|  | Most people in the community think that HIV is a dirty or immoral disease. | 1 | 2 | *88* | *99* |

| H/A Knowledge and beliefs  THESE QUESTIONS REFER TO H/A KNOWLEDGE AND BELIEFS.  **I’d like to know about your beliefs and thoughts about HIV/AIDS, and the services and treatment available for people who are HIV positive. I will read a series of statements and I would like you to tell me whether you agree or disagree with each of them. Please remember that there are no right or wrong answers and anything you say will be kept private and will not affect the care you receive for HIV. If any of these questions make you uncomfortable, just let me know and we can skip the question.** | | | | | |
| --- | --- | --- | --- | --- | --- |
| *Read each statement to the participant, followed by "do you agree or disagree?"* | | **Agree** | **Disagree** | **Don’t know** | **Refused** |
|  | Persons with HIV can live a long and healthy life if they take ART. | 1 | 2 | *88* | *99* |
|  | ART is not effective. | 1 | 2 | *88* | *99* |
|  | Persons who take ART are less likely to pass HIV to their partners during sex. | 1 | 2 | *88* | *99* |
|  | After testing HIV positive, there is no need to immediately get HIV care if you are feeling good. | 1 | 2 | *88* | *99* |
|  | Traditional medicine is not as good as ART in treating HIV/AIDS. | 1 | 2 | *88* | *99* |
|  | ART can help women have babies without HIV. | 1 | 2 | *88* | *99* |
|  | If people follow instructions for taking ART they will be much healthier. | 1 | 2 | *88* | *99* |
|  | AIDS no longer kills everyone because of ART. | 1 | 2 | *88* | *99* |
|  | Traditional healers have cured people with HIV. | 1 | 2 | *88* | *99* |
|  | Only when a person with HIV starts to feel bad should he/she go to the clinic. | 1 | 2 | *88* | *99* |
|  | ART is not worth taking because it has harmful side effects. | 1 | 2 | *88* | *99* |
|  | ART will make you sterile. | 1 | 2 | *88* | *99* |
|  | Persons with HIV can live a long and healthy life if they get treated with traditional medicine. | 1 | 2 | 3 | *99* |
|  | As long as a person with HIV feels healthy, there is no need to go to the clinic. | 1 | 2 | 3 | *99* |
|  | HIV/AIDS is not as severe when people take traditional medicine. | 1 | 2 | 3 | *99* |
|  | ART is only given to persons with HIV who are feeling ill. | 1 | 2 | 3 | *99* |
|  | ART will NOT help me live longer. | 1 | 2 | 3 | *99* |

*Interviewer Comments:*

*Interview was ______ Routine _____ Not routine If not routine, why:*

*Did the participant have any negative reactions in the interview? ______ Yes ______ No*

*If yes, specify:*

*Other observations / comments:*

**Appendix B9**

**A4A**

**General Health Assessment for Children (GHAC)**

**Quality of Life Questionnaire – Adolescent**

1. Since the last visit, have any of the following occurred?

At enrollment: Have any of the following occurred within the last 12 months?

1. You, parent, or partner lost job ◻ Yes ◻ No
2. You, parent, or partner started a new job ◻ Yes ◻ No
3. Family member left home ◻ Yes ◻ No
4. Loss of housing or had to move ◻ Yes ◻ No
5. Loss of support, such as nutritional support ◻ Yes ◻ No

of assistance from social work

1. Transition from adolescent center to adult clinic ◻ Yes ◻ No
2. Loss of health insurance ◻ Yes ◻ No
3. Family member hospitalized ◻ Yes ◻ No
4. Family member very sick ◻ Yes ◻ No
5. Change of caretaker ◻ Yes ◻ No
6. Separation from partner or parents ◻ Yes ◻ No
7. Divorce from partner or of parents ◻ Yes ◻ No
8. Jail sentence for you, caregiver, or partner ◻ Yes ◻ No
9. Marriage for you or caregiver ◻ Yes ◻ No
10. Birth of sibling ◻ Yes ◻ No
11. Birth of a child ◻ Yes ◻ No
12. Mother starting to work ◻ Yes ◻ No
13. Beginning school/college/university ◻ Yes ◻ No
14. Moving to a new to a new school/college/university ◻ Yes ◻ No
15. Completing or leaving school/college/university ◻ Yes ◻ No
16. Change in financial status of parent ◻ Yes ◻ No
17. Loss of close friend (to AYA) ◻ Yes ◻ No
18. Death in family ◻ Yes ◻ No

If death in family, who died ◻ Mother

◻ Father

◻ Brother or sister

(stepbrother or stepsister)

◻ Grandparent

◻ Spouse/partner

◻ Other family member

1. When I have a difficult problem or something that is really bothering me, I have friends or family members I can turn to for help. Would you say this statement is:

◻ Definitely true ◻ Probably true ◻ Probably false ◻ Definitely false

| ***I. General Health Ratings*** | | |
| --- | --- | --- |
| These statements ask about your health and behavior. On a scale from 1 to 10 (1 being the very worst and 10 being the very best): How have you been feeling on the average, during the past three months? | | |
| Feeling | The Very Worst | The Very Best |
| Overall, in general | 1 2 3 4 5 6 7 8 9 10 | |
| Physically? | 1 2 3 4 5 6 7 8 9 10 | |
| Emotionally? | 1 2 3 4 5 6 7 8 9 10 | |
| About their usual daily activities, such as schoolwork, jobs or housework? | 1 2 3 4 5 6 7 8 9 10 | |

| ***II. Infection Status*** |
| --- |
| These questions ask about your knowledge of your health. Fill in the blanks where appropriate. |
| 1. How old were you when you were told of your HIV diagnosis?  ____Years, ____Months □ Can’t remember / Don’t know |
| 2. Who discussed the diagnosis with you?  □ Primary caregiver □ Other family members  □ Health care team: Who___________□ Other: Who:___________ |
| 3. Who participated in the discussion of the diagnosis?  □ Primary caregiver □ Other family members: Who:___________  □ Health care team: Who___________ □ Other: Who:___________ |
| 4. How did you feel about the discussion of your diagnosis?  □ I felt the discussion was helpful  □ I felt I had enough support from the health care team  □ I understood what was explained  □ I did not feel like I understood what was explained  □ I did not feel the health care team was helpful enough  □ I felt the discussion was too difficult  □ Other |
| 5. When you were first told of your diagnosis, were you:   | 1. Sad? | □ Yes □ No | | --- | --- | | 1. Content/glad? | □ Yes □ No | | 1. Frightened? | □ Yes □ No | | 1. Relieved? | □ Yes □ No | | 1. Angry? | □ Yes □ No | | 1. Had no reaction? | □ Yes □ No |   7. How do you feel NOW about life with your medical condition?   | 1. Sad? | □ Yes □ No | | --- | --- | | 1. Content/glad? | □ Yes □ No | | 1. Frightened? | □ Yes □ No | | 1. “In Control”? | □ Yes □ No | | 1. Angry? | □ Yes □ No | | 1. No change? | □ Yes □ No | |
| 8. What has been good about you knowing your diagnosis?   | 1. I am more cooperative about medical care and taking medicine | □ Yes □ No | | --- | --- | | 1. I am more open with family and friends | □ Yes □ No | | 1. I understand why I am sick | □ Yes □ No | | 1. I understand responsibility to avoid infecting others | □ Yes □ No | | 1. Telling me the truth has improved my relationship with my family | □ Yes □ No | | 1. Other | □ Yes □ No  Specify_____________________________________ |   9. Are there any of the following problems because you know your diagnosis?   | 1. I told friends, neighbors or others who shouldn’t know | □ Yes □ No | | --- | --- | | 1. I told family members who shouldn’t know | □ Yes □ No | | 1. I am sad or depressed | □ Yes □ No | | 1. I am afraid of death | □ Yes □ No | | 1. My behavior is worse | □ Yes □ No | | 1. I am less cooperative with medical care or taking medicines | □ Yes □ No | | 1. I am left out or discriminated against | □ Yes □ No | | 1. Other? | □ Yes □ No Specify:_____________ | |

| **III. Barriers and Preferences** |  |
| --- | --- |
| Thinking about your clinic attendance and viral suppression, what do you think are your biggest challenges? Please rank the top 5, with 1 being the biggest challenge from the list. | 1. 2. 3. 4. 5.   Biggest challenge. 5^th^ biggest challenge |
| Thinking about other people your age attending the clinic and viral suppression, what do you think are their biggest challenges? Please rank the top 5, with 1 being the biggest challenge from the list. | 1. 2. 3. 4. 5.   Biggest challenge. 5^th^ biggest challenge |
| 1. **Access to care**    1. Transportation problems    2. Funds to get to the clinic    3. Didn’t have enough time for the clinic    4. Other access to care reasons, (specify ______________) 2. **Work, School, and Family**     1. Work interference    2. School interference w/refill pick up    3. School interference w/taking treatment    4. AYA/Caregiver attended funeral    5. AYA/Caregiver had other family obligation    6. Partner didn’t want me to attend clinic    7. Marriage, birth, caregiver change (specify ______________)    8. Other family/work reason, specify 3. **Medical** 4. Felt too sick to come to clinic    1. Caregiver felt too sick to come to clinic    2. Side effects from pills    3. Pill burden    4. Wanted a break from pills    5. Pregnancy/gave birth    6. Other medical reasons, specify:    7. ___ Hospitalized🡪 Admit date: ______/________/________    8. DD /MM /YYYY    9. Hospital name:    10. Hospitalization Reason: 5. **Clinic Quality**    1. The clinic ran out of medications    2. Transition to the adult clinic    3. The staff was not nice    4. The care was not good    5. Too many admin requirements    6. The waiting area was uncomfortable    7. The clinic was too crowded    8. I have to wait too long during visits    9. I didn’t want to go to retention class 6. **Psycho-social**     1. Felt well, didn’t see need to visit clinic    2. Attending clinic risked HIV disclosure to someone in community    3. Afraid clinic would scold me for missing appoint.    4. Because I went to someone who tried / is trying to cure me by prayer / religious rituals.    5. Because I saw / am seeing a traditional healer instead    6. Felt hopeless/depressed    7. Alcohol consumption    8. Forgot appointment    9. Family member or other important person said child should stop going to clinic 7. *Don’t know* 8. *Refused* | |
|  |  |
| If you are struggling with staying engaged in HIV care (eg. challenges attending clinic visits, taking medication daily, being virally suppressed), which support would likely be most helpful to you? Rank the top 3, with 1 being the top choice. | Mobile peer support  In-person peer support  Small financial assistance  Text reminders  Other (specify) ___________________  No additional support |
| If someone your age is struggling with staying engaged in HIV care, which support would likely be most helpful to them? Rank the top 3, with 1 being the top choice. | Mobile peer support  In-person peer support  Small financial assistance  Text reminders  Other (specify) ___________________  No No additional support |

| ***IV. Psychological Well-Being*** |
| --- |
| These statements are about behavior problems many adolescents and young adults have. As you read each sentence, decide which phrase best describes your behavior over the past 3 months. |
| Thinking about my behavior, during the past 3 months…… |
| 1. I have sudden changes in mood or feelings   □ Often True □ Sometimes True □ Not True |
| 1. I feel that no one loves me   □ Often True □ Sometimes True □ Not True |
| 1. I am rather high strung, tense, and nervous   □ Often True □ Sometimes True □ Not True |
| 1. I cheat or tell lies   □ Often True □ Sometimes True □ Not True |
| 1. I am too fearful or anxious   □ Often True □ Sometimes True □ Not True |
| 1. I argue too much   □ Often True □ Sometimes True □ Not True |
| 1. I have difficulty concentrating, cannot pay attention for long   □ Often True □ Sometimes True □ Not True |
| 1. I am easily confused, seem to be in a fog   □ Often True □ Sometimes True □ Not True |
| 1. I bully or am cruel or mean to others   □ Often True □ Sometimes True □ Not True |
| 1. I am disobedient at home   □ Often True □ Sometimes True □ Not True |
| 1. I am disobedient at school   □ Often True □ Sometimes True □ Not True |
| 1. I do not feel sorry after I misbehave   □ Often True □ Sometimes True □ Not True |
| 1. I have trouble getting along with others   □ Often True □ Sometimes True □ Not True |
| 1. I have trouble getting along with teachers   □ Often True □ Sometimes True □ Not True |
| 1. I am impulsive, or act without thinking   □ Often True □ Sometimes True □ Not True |
| 1. I feel worthless or inferior   □ Often True □ Sometimes True □ Not True |
| 1. I am not liked by others   □ Often True □ Sometimes True □ Not True |
| 1. I have a lot of difficulty getting my mind off certain thoughts   □ Often True □ Sometimes True □ Not True |
| 1. I am restless or overly active, cannot sit still   □ Often True □ Sometimes True □ Not True |
| 1. I am stubborn, sullen or irritable   □ Often True □ Sometimes True □ Not True |
| 1. I have a very strong temper and lose it easily   □ Often True □ Sometimes True □ Not True |
| 1. I am unhappy, sad or depressed   □ Often True □ Sometimes True □ Not True |
| 1. I am withdrawn, do not get involved with others   □ Often True □ Sometimes True □ Not True |
| 1. I feel others are out to get me   □ Often True □ Sometimes True □ Not True |
| 1. I hang around with kids who get into trouble   □ Often True □ Sometimes True □ Not True |
| 1. I am secretive, keep things to myself   □ Often True □ Sometimes True □ Not True |
| 1. I worry too much   □ Often True □ Sometimes True □ Not True |
| 1. I am too dependent on others   □ Often True □ Sometimes True □ Not True |

**V. A4A COVID-19 experiences**

(*Do not read “don’t know” or “prefer not to answer” as a choice for each question. Use if the patient*

*Indicates that they do not know or declines to answer a question*.)

**Section 1. COVID-19 knowledge**

| 1.1 | Have you heard of the coronavirus outbreak/pandemic also called COVID-19? | ⬜ yes  ⬜ no  ⬜ *don’t know*  ⬜ *prefer not to answer* |
| --- | --- | --- |

**Section 2. COVID-19 preparedness**

| 2.1 | Do you have any concerns about being able to stay healthy and safe during the coronavirus outbreak/pandemic? | ⬜ yes, have concerns  ⬜ no, do not have concerns 🡪 2.3  ⬜ *don’t know* 🡪 2.3  ⬜ *prefer not to answer* 🡪 2.3 |
| --- | --- | --- |
| 2.2 | If yes, what is your greatest concern? What other concerns do you have? | ⬜ *prefer not to answer* |
| 2.3 | What measures (if any) have you been practicing to protect yourself from coronavirus? (*Do not read the list; tick all that are stated*) | ⬜ washing hands frequently / more often than usual  ⬜ covering your face with a mask or scarf  ⬜ avoiding touching your face  ⬜ avoiding people who are sick  ⬜ avoiding visitors or social gatherings  ⬜ avoiding public transportation  ⬜ not going to work site / not going to do informal work  ⬜ not going to church  ⬜ other (specify _______________________)  ⬜ none / no changes to routine  ⬜ *don’t know*  ⬜ *prefer not to answer* |

**Section 3. Impact on life**

| 3.1 | Has the coronavirus impacted where you are living now? | ⬜ yes  ⬜ no 🡪 SKIP to 3.3  ⬜ don’t know 🡪 SKIP to 3.3  ⬜ prefer not to answer 🡪 SKIP to 3.3 |
| --- | --- | --- |
| 3.2 | Where are you living now? | ⬜ usual residence  ⬜ rural home  ⬜ relative’s home  ⬜ friend’s home  ⬜ work site  ⬜ school  ⬜ institution / group home  ⬜ street-connected  ⬜ other (specify _______________________)  ⬜ *prefer not to answer* |
| 3.3 | Have you had to travel away from town to a rural area because of the current outbreak? | ⬜ yes (If so, why? ______________________________ )  ⬜ no  ⬜ *don’t know*  ⬜ *prefer not to answer* |
| 3.4 | Has the coronavirus (COVID-19) impacted your classes/studies? | ⬜ yes, studies are virtual now  ⬜ yes, studies are mixed now (virtual and some in-person)  ⬜ yes, but studies are now all in-person (reverted back to in-person)  ⬜ yes, no longer enrolled in school/college/university studies  ⬜ yes, decided to enroll in school/college/university studies  ⬜yes changed/transferred schools  ⬜ no change  ⬜ not applicable  ⬜ *don’t know*  ⬜ *prefer not to answer* |
| 3.5 | Prior to the current outbreak/pandemic, were you working or earning income? | ⬜ yes  ⬜ no 🡪 SKIP to  ⬜ don’t know 🡪 SKIP to  ⬜ prefer not to answer 🡪 SKIP to |
| 3.6 | Have/did you lose a job or lost income during the outbreak? | ⬜ no loss of job or income  ⬜ lost formal job  ⬜ lost other income  ⬜ *don’t know*  ⬜ *prefer not to answer* |
| 3.7 | Have you started a new job or earned new income since the outbreak? | ⬜ no new job or income  ⬜ new formal job  ⬜ new other income  ⬜ *don’t know*  ⬜ *prefer not to answer* |
| 3.8 | How much has/did coronavirus (COVID-19) impact your access to food? | ⬜ Not at all ⬜ A little ⬜ Much ⬜ Very much ⬜ Extremely  ⬜ *don’t know*  ⬜ *prefer not to answer* |
| 3.9 | How much is the coronavirus (COVID-19) impacting your current day-to-day life? | ⬜ Not at all ⬜ A little ⬜ Much ⬜ Very much ⬜ Extremely ⬜ *don’t know*  ⬜ *prefer not to answer* |

**Section 4. Access to antiretroviral therapy**

| 4.1 | Do you currently have ARV medications? | ⬜ yes, currently have ARVs  ⬜ no 🡪 **DIRECT REFERRAL**  ⬜ N/A – not on treatment 🡪 SKIP to next section.  ⬜ *don’t know*  ⬜ *prefer not to answer* |
| --- | --- | --- |
| 4.2 | During the outbreak/pandemic, have you had any concerns about running out of your medications? | ⬜ yes  ⬜ no  ⬜ *don’t know*  ⬜ *prefer not to answer* |
| 4.3 | During the outbreak/pandemic, have you skipped any medication doses because you were concerned about running out of medications? | ⬜ yes  ⬜ no  ⬜ *don’t know*  ⬜ *prefer not to answer* |
| 4.4 | During the outbreak/pandemic, have you had greater difficulties than usual in refilling your medications? | ⬜ yes 🡪 **DIRECT REFERRAL**  ⬜ no 🡪 SKIP to next section.  ⬜ *don’t know*  ⬜ *prefer not to answer* |
| 4.5 | If yes, what kinds of difficulties have you had refilling your medications? (Don’t read the options; tick all that are stated.) | ⬜ clinic or facility not open  ⬜ clinic or facility out of stock  ⬜ transportation too expensive or not available  ⬜ curfew, restrictions on travel, or police crackdowns  ⬜ fear of traveling  ⬜ have been unwell (self)  ⬜ household or family members have been unwell  ⬜ other (specify _______________________)  ⬜ *don’t know*  ⬜ *prefer not to answer* |
| 4.6 | Since the outbreak/pandemic, did the clinic change how often you come to the clinic or how frequently you receive your ART? (Tick all that apply) | ⬜ yes, my TCA was shortened  ⬜ yes, my TCA was extended  ⬜yes, I received a longer time between refills  ⬜yes, I had clinical care over the phone with a provider (telemedicine)  ⬜yes, I had my medicine delivered  ⬜yes, I was told not to come to the clinic  ⬜yes, other (specify ____________)  ⬜ no 🡪 SKIP to 5.1  ⬜ not applicable / has not sought medical care 🡪 SKIP to 5.1  ⬜ *don’t know* – SKIP to 5.1  ⬜ *prefer not to answer* 🡪 SKIP to 5.1 |
| 4.7 | If yes, do/did you prefer the change? | ⬜ yes, very much prefer  ⬜ yes, somewhat prefer  ⬜ no, prefer usual care  ⬜no opinion, fine either way  ⬜ *don’t know*  ⬜ *prefer not to answer* |

**Section 5. Antiretroviral adherence**

| 5.1 | Have you made any changes regarding taking your ARV medications as a result of the current outbreak? | ⬜ yes  ⬜ no 🡪 SKIP to 6.1  ⬜ don’t know 🡪 SKIP to 6.1  ⬜ prefer not to answer 🡪 SKIP to 6.1 |
| --- | --- | --- |
| 5.2 | What changes have you had to make (if any) regarding taking your ARV medications as a result of the current outbreak? |  |

**Section 6. Coronavirus (COVID-19) symptoms, testing, diagnosis and treatment**

| 6.1 | Since the outbreak, have you had any of the following symptoms? (read options; tick all that apply) | ⬜ fever  ⬜ cough  ⬜ shortness of breath or difficulty breathing  ⬜ tiredness / fatigue  ⬜ body aches / myalgias  ⬜ runny nose / congestion  ⬜ sore throat  ⬜ vomiting  ⬜ diarrhea  ⬜ **any other symptoms** not listed (specify _______________________)  ⬜ no symptoms  ⬜ *don’t know*  ⬜ *prefer not to answer* |
| --- | --- | --- |
| 6.2 | Have you had a laboratory test for coronavirus (COVID-19) (*a swab was used to take a sample from your nose*)? | ⬜ yes  ⬜ no – SKIP to 6.4  ⬜ *don’t know* – SKIP to 6.4  ⬜ *prefer not to answer*. – SKIP to 6.4 |
| 6.3 | Have you ever been told by a medical provider that you have had coronavirus (COVID-19)? | ⬜ yes, based on positive test  ⬜ yes, based on symptoms  ⬜ yes, unsure how determined  ⬜ no  ⬜ *don’t know*  ⬜ *prefer not to answer* |
| 6.4 | Were you hospitalized for medical care for COVID-19? | ⬜ yes  ⬜ no  ⬜ *don’t know*  ⬜ *prefer not to answer* |
| 6.5 | Have any household members or other close contacts ever been told by a medical provider that they have had coronavirus (COVID-19)? | ⬜ yes, based on positive test  ⬜ yes, based on symptoms  ⬜ yes, unsure how determined  ⬜ no 🡪 SKIP to 7.1  ⬜ *don’t know* 🡪 SKIP to 7.1  ⬜ *prefer not to answer* 🡪 SKIP to 7.1 |
| 6.6 | Has any household member of close contact with COVID-19 been hospitalized for medical care for COVID-19? | ⬜ yes  ⬜ no  ⬜ *don’t know*  ⬜ *prefer not to answer* |

**Section 7. Mental well-being**

| 7.1 | Since the outbreak, would you say that your overall stress level has improved, gotten worse, or stayed about the same? |  Stayed about the same   Gotten worse   Improved   *don’t know*   *prefer not to answer* |
| --- | --- | --- |
| 7.2 | Since the outbreak, would you say that your overall mental health has improved, gotten worse, or stayed about the same? |  Stayed about the same   Gotten worse   Improved   *don’t know*   *prefer not to answer* |

**Appendix B10**

**ADAPT-R Enhanced Retrospective Review – hospitalizations**

**Demographics**

1. Adapt study identifier
2. Names

Administrative

1. Date of interview

Interviewer initials Healthcare utilization in the past 30 days

| **Illness or injury in the past 30 days (ONE episode per line)** | | | | | | | | | | | | | | | | |
| --- | --- | --- | --- | --- | --- | --- | --- | --- | --- | --- | --- | --- | --- | --- | --- | --- |
|  | During the past 30 days, did you suffer from any illness or injury, for example, a cough, cold, diarrhea, an injury due to an accident or any other illness?  1 Yes, illness  2 Yes, injury  3 Yes, illness and injury  4 No **àGo to the Hospitalization Section** | Starting with the most severe episode, what sort of illness/injury did you suffer?  *Record up to 3 disease/symptom codes per episode (see code list including other-specify)* | | | Where did you go for treatment?  If no treatment was sought, why didn’t you go for treatment?  *Record up to 3 provider codes per episode; if no treatment was sought, use the “none” codes to record the reason(s). Allow for other-specify* | | | **Total amount spent for medical care (in Kenya Shillings)**  If care was received for free, record “0” | | | | | | Total number of hours spent seeking and receiving care | Total number of hours lost from usual school, employment, or household activities by you because of this illness/injury? | Was there any other illness/injury you suffered in the past 30 days?  1. Yes ***à Go to the next line, question 3a, for the next most severe episode, up to 3 episodes per individual***  2. No **à *Go to the Hospitalization Section*** |
|  |  |  |  |  |  |  |  | Travel for medical care (round-trip) | Outpatient consultation fees | Medicines | Laboratory tests | Inpatient hospital fees *(incl. food, etc.)* | Other health expenditures |  |  |  |
| 1 | 2 | 3a | 3b | 3c | 4a | 4b | 4c | 5 | 6 | 7 | 8 | 9 | 10 | 11 | 12 | 13 |
| Episode 1 |  |  |  |  |  |  |  |  |  |  |  |  |  |  |  |  |
| Episode 2 |  |  |  |  |  |  |  |  |  |  |  |  |  |  |  |  |
| Episode 3 |  |  |  |  |  |  |  |  |  |  |  |  |  |  |  |  |

Hospitalizations in the past 12 months

| During the past year, how many times were you hospitalized (stayed in a health facility overnight)?  If “0” *à* ***End the survey*** | 2. Starting from the most recent hospitalization,  - What was the main diagnosis/reason for that hospitalization? *Record the main disease/symptom code for each hospitalization*.  - How many days did you spend in the hospital?  *Record up to 3 hospitalizations, starting from the most recent one.* | | | | | | | |
| --- | --- | --- | --- | --- | --- | --- | --- | --- |
|  | Hospitalization 1 (most recent) | | | Hospitalization 2 | | | Hospitalization 3 | |
|  | a. Diagnosis | | b. Days | c. Diagnosis | d. Days | | e. Diagnosis | f. Days |
|  |  | |  |  |  | |  |  |
|  |  | |  |  |  | |  |  |
|  |  | |  |  |  | |  |  |
| **Disease/symptom codes:**   1. Abdominal pain 2. Accident/Trauma 3. Arthritis 4. Breathing difficulty 5. Burn 6. Cancer 7. Chills (feeling hot and cold) 8. Coughing 9. Coughing blood 10. Dental 11. Diabetes 12. Diarrhea (acute) 13. Diarrhea (chronic, 1 month or more) 14. Fainting 15. Fever (acute) 16. Fever (recurring) 17. Fracture 18. General body pain 19. Genital sores | | 1. HIV/AIDS 2. Headache (severe) 3. Hypertension 4. Malaria 5. Measles 6. Mental disorder/illness 7. Pain on passing urine 8. Pneumonia 9. Pregnancy related 10. Sore throat 11. Skin rash 12. TB 13. Ulcers 14. Vomiting 15. Weakness 16. Weight loss (major) 17. Wound 18. Other (specify) | | | | **Provider codes**   1. Hospital 2. Home (family) 3. Home (traditional healer) 4. Traditional healer (outside home) 5. Private clinic 6. Health center 7. Drug shop 8. Pharmacy 9. Ordinary shop 10. Other (specify the treatment location) 11. None – Too expensive to seek treatment 12. None – Far away from the health care provider 13. None – Didn’t feel sick enough 14. None – Had other responsibilities (e.g., school, employment, housework, etc.) 15. None – Other reason (specify the reason for not seeking treatment) | | |

**Appendix B12**

**Comprehensive ART Adherence Measurement**

**Short Form (CAMP-SF) – ADOLESCENT EVALUATION**

*Now I am going to ask you some questions about adherence.*

| **1. Are you enrolled in a nutrition program?** □ Yes □ No | |
| --- | --- |
| **ADHERENCE QUESTIONNAIRE** | |
| **2. Do you ever have problems keeping time with the medicines?**  □ Yes □ No  **When?** □ Mornings □ Evenings □Weekends  □Weekdays □ Other:____________________ | **3. Do you ever have problems with taking the medicines?**  □ Yes □ No  **What problems do you have? (explain)** |
| **4. Sometimes taking medicine can be difficult. Have you not taken medicines for any of these reasons:**  □ I forgot to take medicine □ I get tired of taking the medicine every day  □ I was at school/work/away from home. □ I refused to take medicine  □ Did not want others to see □ I find medicines too bitter  □ The medicine makes me feel sick □ Other (specify):_________________________  □ I felt ill or was vomiting  □ I can’t take without food  □ None of the above | |
| **5. Sometimes, problems at the clinic make it difficult for you to take these medicines every day. Have any of these things been a problem for you:**  □ The medicine was not available in the pharmacy ***Which medicine?*** □ ARVs □ Septrin □ Other (include abx)  □ I finished or ran out of the medicines  □ The wait time was too long and I left  □ Other (specify) __________________________  □ None of the above | |
| **We are now going to ask you a few questions about missing doses. What we mean by a “dose” is any of the medicine that you are supposed to take in the mornings or evenings. If you took your medicines in the morning but missed even one medicine in the evening, then you would have taken one dose and missed one dose. If you missed any of the medicines in the morning and missed again in the evening, you would miss two doses. If you have questions about doses, please ask as we go along.** | |
| **6. In the past week,**  a. On how many days did you miss at least one dose? □0 □1 □2 □3 □4 □5 □6 □7 **□** Don’t know  b. On how many days did you take a dose more than an hour late? □0 □1 □2 □3 □4 □5 □6 □7 **□** Don’t know  c. How many extra doses or syringes of medicine did you take? _________________________ **□** Don’t know | |
| **7. How many doses of medicine did you miss in the last month?** ___________ **□** Don’t know | |

**Appendix B14**

PATIENT HEALTH QUESTIONNAIRE-9 ( P H Q - 9 )

Circle the appropriate response.

| Questions | Response | | | |
| --- | --- | --- | --- | --- |
| Over the last 2 weeks, how often have you been bothered by any of the following problems? (Use “✔” to indicate your answer) | Not at all | Several days | More than half the days | Nearly every day |
| 1. Little interest or pleasure in doing things | 0 | 1 | 2 | 3 |
| 2. Feeling down, depressed, or hopeless | 0 | 1 | 2 | 3 |
|  | If #1 and #2 are both 0, go to end.  Otherwise proceed to #3 | | | |
| 3. Trouble falling or staying asleep, or sleeping too much | 0 | 1 | 2 | 3 |
| 4. Feeling tired or having little energy | 0 | 1 | 2 | 3 |
| 5. Poor appetite or overeating | 0 | 1 | 2 | 3 |
| 6. Feeling bad about yourself — or that you are a failure or have let yourself or your family down | 0 | 1 | 2 | 3 |
| 7. Trouble concentrating on things, such as reading the newspaper or watching television | 0 | 1 | 2 | 3 |
| 8. Moving or speaking so slowly that other people could have noticed? Or the opposite — being so fidgety or restless that you have been moving around a lot more than usual | 0 | 1 | 2 | 3 |
|  |  |  |  |  |
| 9. Thoughts that you would be better off dead or of hurting yourself in some way | 0 | 1 | 2 | 3 |

Total score:

Score 10 to 19 = refer to clinic for counseling

Score 20 or higher = refer to clinic for psychiatrist referral

**Appendix B17**

Adverse Childhood Experiences International Questionnaire (ACE-IQ)

|  | DEMOGRAPHIC INFORMATION | |
| --- | --- | --- |
| 1 | MARRIAGE | |
| 1.2  [M2] | If you have ever been married, at what age were you first married? | Age [ ][ ] |
|  |  | Never married [Go to M5] |
|  |  | Refused [Go to M5] |
| 1.3  [M3] | At the time of your first marriage did you did you choose your husband/wife? | Yes *(Go to M5)* |
|  |  | No |
|  |  | Don't know / Not sure |
|  |  | Refused |
| 1.4  [M4] | At the time of your first marriage if you did not choose your husband/wife yourself, did you give your consent to the choice? | Yes |
|  |  | No |
|  |  | Refused |
| 1.5  [M5] | If you are a mother or father what was your age when your first child was born? | Age [ ][ ] |
|  |  | Not applicable |
|  |  | Refused |

| 2 | RELATIONSHIP WITH PARENTS/GUARDIANS | | |
| --- | --- | --- | --- |
|  | *ASK ALL*  During your childhood (the first 18 years of your life) . . . | | |
| 2.1  [P1] | Did/do your parents/guardians understand your problems and worries? | Always | |
|  |  | Most of the time | |
|  |  | Sometimes | |
|  |  | Rarely | |
|  |  | Never | |
|  |  | Refused | |
| 2.2  [P2] | Did/do your parents/guardians really know what you were doing with your free time when you were not at school or work? | Always | |
|  |  | Most of the time | |
|  |  | Sometimes | |
|  |  | Rarely | |
|  |  | Never | |
|  |  | Refused | |
| 3 |  | | |
| 3.1  [P3] | How often did/do your parents/guardians not give you enough food even when they could easily have done so? | Many times | |
|  |  | A few times | |
|  |  | Once | |
|  |  | Never | |
|  |  | Refused | |
| 3.2  [P4] | Were/are your parents/guardians too drunk or intoxicated by drugs to take care of you? | Many times | |
|  |  | A few times | |
|  |  | Once | |
|  |  | Never | |
|  |  | Refused | |
| 3.3  [P5] | How often did/do your parents/guardians not send you to school even when it was/is available? | Many times | |
|  |  | A few times | |
|  |  | Once | |
|  |  | Never | |
|  |  | Refused | |
| 4 | FAMILY ENVIRONMENT | | |
|  | *ASK ALL*  During your childhood (first 18 years of your life) | | |
| 4.1  [F1] | Did/do you live with a household member who had/has a problem drinker or alcoholic, or misused street or prescription drugs? | | Yes |
|  |  |  | No |
|  |  |  | Refused |
| 4.2  [F2] | Did/do you live with a household member who was/is depressed, mentally ill or suicidal? | | Yes |
|  |  |  | No |
|  |  |  | Refused |
| 4.3  [F3] | Did/do you live with a household member who was ever sent to jail or prison? | | Yes |
|  |  |  | No |
|  |  |  | Refused |
| 4.4  [F4] | Were/are your parents separated or divorced? | | Yes |
|  |  |  | No |
|  |  |  | Not applicable |
|  |  |  | Refused |
| 4.5  [F5] | Did your mother, father or guardian die? | | Yes |
|  |  |  | No |
|  |  |  | Don't know / Not sure |
|  |  |  | Refused |
| *ASK ALL*  These next questions are about certain things you may actually have heard or seen IN YOUR HOME. These are things that may have been done to another household member but not necessarily to you. | | | |

| During your childhood (the first 18 years of your life)… | | | |
| --- | --- | --- | --- |
| 4.6  [F6] | Did/do you see or hear a parent or household member in your home being yelled at, screamed at, sworn at, insulted or humiliated? | | Many times |
|  |  |  | A few times |
|  |  |  | Once |
|  |  |  | Never |
|  |  |  | Refused |
| 4.7  [F7] | Did/do you see or hear a parent or household member in your home being slapped, kicked, punched or beaten up? | | Many times |
|  |  |  | A few times |
|  |  |  | Once |
|  |  |  | Never |
|  |  |  | Refused |
| 4.8  [F8] | Did/do you see or hear a parent or household member in your home being hit or cut with an object, such as a stick (or cane), bottle, club, knife, whip etc.? | | Many times |
|  |  |  | A few times |
|  |  |  | Once |
|  |  |  | Never |
|  |  |  | Refused |
| *ONLY ASK THOSE 18 AND OLDER*  These next questions are about certain things YOU may have experienced.  During your childhood (the first 18 years of your life)… | | | |
| 5 |  | | |
| 5.1  [A1] | Did/does a parent, guardian or other household member yell, scream or swear at you, insult or humiliate you? | Many times | |
|  |  | A few times | |
|  |  | Once | |
|  |  | Never | |
|  |  | Refused | |
| 5.2  [A2] | Did/does a parent, guardian or other household member threaten to, or actually, abandon you or throw you out of the house? | Many times | |
|  |  | A few times | |
|  |  | Once | |
|  |  | Never | |
|  |  | Refused | |
| 5.3  [A3] | Did/does a parent, guardian or other household member spank, slap, kick, punch or beat you up? | Many times | |
|  |  | A few times | |
|  |  | Once | |
|  |  | Never | |
|  |  | Refused | |
| 5.4  [A4] | Did/does a parent, guardian or other household member hit or cut you with an object, such as a stick (or cane), bottle, club, knife, whip etc? | Many times | |
|  |  | A few times | |
|  |  | Once | |
|  |  | Never | |
|  |  | Refused | |

| 6 | PEER VIOLENCE | | | |
| --- | --- | --- | --- | --- |
| *ASK ALL*  These next questions are about BEING BULLIED during your childhood. Bullying is when a young person or group for young people say or do bad and unpleasant things to another young person. It is also bullying when a young person is teased a lot in an unpleasant way or when a young person is left out of things on purpose. It is not bullying when two young people of about the same strength or power, argue or fight or when teasing is done in a friendly and fun way. During your childhood, the (first 18 years of your life)… | | | | |
| 6.1  [V1] | How often were/are you bullied? | | | Many times |
|  |  |  |  | A few times |
|  |  |  |  | Once |
|  |  |  |  | Never *(Go to Q.V3)* |
|  |  |  |  | Refused |
| 6.2  [V2] | How were you bullied most often? | | | I was hit, kicked, pushed, shoved around, or locked indoors |
|  |  |  |  | I was made fun of because of my race, nationality or colour |
|  |  |  |  | I was made fun of because of my religion |
|  |  |  |  | I was made fun of with sexual jokes, comments, or gestures |
|  |  |  |  | I was left out of activities on purpose or completely ignored |
|  |  |  |  | I was made fun of because of how my body or face looked |
|  |  |  |  | I was bullied in some other way |
|  |  |  |  | Refused |
| *ASK ALL*  This next question is about PHYSICAL FIGHTS. A physical fight occurs when two young people of about the same strength or power choose to fight each other. | | | | |
| During your childhood (the first 18 years of your life) . . . | | | | |
| 6.3  [V3] | How often were you in a physical fight? | | | Many times |
|  |  |  |  | A few times |
|  |  |  |  | Once |
|  |  |  |  | Never |
|  |  |  |  | Refused |
| 7 | | EXPOSURE TO WAR/COLLECTIVE VIOLENCE | | |
|  | | *ASK ALL*  These questions are about whether YOU did or did not experience any of the following events when you were a child. The events are all to do with collective violence, including wars, terrorism, political or ethnic conflicts, genocide, repression, disappearances, torture and organized violent crime such as banditry and gang warfare.  During your childhood (the first 18 years of your life . . .) | | |
| 7.1  [V4] | | Have you been forced to go and live in another place due to any of these events? | Many times | |
|  |  |  | A few times | |
|  |  |  | Once | |
|  |  |  | Never | |
|  |  |  | Refused | |
| 7.2  [V5] | | Did/do you experience the deliberate destruction of your home due to any of these events? | Many times | |
|  |  |  | A few times | |
|  |  |  | Once | |
|  |  |  | Never | |
|  |  |  | Refused | |
| 7.3  [V6] | | Have you beaten up by soldiers, police, militia, or gangs? | Many times | |
|  |  |  | A few times | |
|  |  |  | Once | |
|  |  |  | Never | |
|  |  |  | Refused | |
| 7.4  [V7] | | Was/is a family member or friend killed or beaten up by soldiers, police, militia, or gangs? | Many times | |
|  |  |  | A few times | |
|  |  |  | Once | |
|  |  |  | Never | |
|  |  |  | Refused | |

**Not listed in the appendix B table of content**

**E-Nav Intervention Form**

Date **__ __/__ __/__ __ __ __ (dd/mm/yyyy)**

Study ID

Clinic ID

**Initial Encounter**

My name is ____________ and I am peer navigator for A4A. I’m here to listen and help you with your HIV care journey, which can have easy and hard days. I’m looking forward to talking with you today and in future sessions. I’m going to ask you a few questions. Feel free to respond openly and honestly, the information will not be shared with the clinic or others outside of this study. Please also ask me any questions you may have at any time.

How is your day going?

Tell me a little about yourself?

What support systems do you have to help you with your HIV care?

Follow-up Probes ***(Examine the strengths, resilience and coping mechanisms for the AYA; to include some of the following)***

| Family  Friends (Peers)Support groups  Clinic (Health Workers)  School (Teachers and support staff) | Community  Social Media  Other…. |
| --- | --- |

What are some of the issues you have experienced while accessing HIV care and treatment?

Most people living with HIV know that taking ‘every pill, every day” (good adherence) and attending all their clinic visits is important, but sometimes it is difficult. What are some of the challenges you have with taking your medications or coming to the clinic?

What are the causes of the issues you have identified?

How did you handle the issues you identified?

What are your health/HIV treatment goals for the future?

What are your immediate next steps to address some of the issues you have raised?

What are your general goals for the future?

***Recap with the AYA on the main points covered during the session.***

***(For the peer lead; pick one or two points discussed above)***

**WHO**-

Who will help you remember to take your medicine every day at the same time?

Who will remind you to come to clinic appointments?

**WHAT**

What medicines are you taking?

What is your dose and how often will you take it?

What will you do when you are about to run out of your medicines?

What will you do if you miss a dose?

**WHEN**

When will you take your medicines?

When is your next clinic visit?

**WHERE**

Where will you take your doses of ARVs (e.g. at school, at home, at work, etc.)?

Where will you store your ARVs?

**HOW**

How will you remember to take your medicines every day and at the same time?

What reminder system will you use?

***Recap with the AYA on the main points covered during the session. Consider AYA needs. Choose the most relevant one or two (possible action in italic)***

HIV/ART knowledge-> ***provide education, address myths and misconceptions***

ART side effects *->* ***connect to clinic provider***

Strengthen support system _> ***connect to OTZ, adolescent peer leads, other support groups***

Disclosure support -> ***discuss approach to disclosure and post-disclosure support***

Food/financial insecurity -> ***referral to OVC or CBO***

Transport -> ***strategize with AYA***

Clinic logistics *->* ***support re-scheduling, coach*** ***on effective patient-provider visits***

Adherence*->* ***medication fatigue, forgetfulness****,* ***lack of disclosure***

Mental Health or Substance use -> ***provide counseling messages, refer as needed***

***If client is doing well:***

- *Praise them for good job*
- *Encourage the client to reach out and talk to you if there are any problems*
- *Emphasize importance of sharing challenges*

***Plan for next encounter*:**

When should we talk again*? (Minimum monthly*)

What is your preferred manner of checking in? *(SMS, WhatsApp, FB, messenger e.t.c)*

**Subsequent Encounters**

Review discussion during the previous session. Review goal(s)/plans

Examine progress on the plans from last session

Review emerging barriers/challenges to health goal

**Additional Notes**

**E-Nav Measurement Form**

| Date of form completion  __ __ / __ __ / __ __ __ __ (DD-MM-YYYY) | HH: MM  ⚪ a.m.  ⚪ p.m. |
| --- | --- |

**Section A**: **Administrative**

Clinic ID

Study ID-

**Navigator Initials**

__ __ / __ __ / __ __ __ __ (DD-MM-YYYY)

**Date of randomization**

**Type of visit**

**Initial contact at randomization (complete B, C, D, and if complete go to E2-5 and F)**

**Follow up contact attempt (complete C, D, E1-5, and F)**

**B. Contact Preferences**

**Preferred contact method, select top three in order of preference**

| □ Phone call  □ SMS  □ Whats app  □ Snapchat | □ In-clinic visit  □ Home visit  □ School visit  □ Other (specify)________________ |
| --- | --- |

**C. Navigator Contact Attempts**

**Number of contact attempts**

**Method of contact attempts for this session**

| □ Phone call  □ SMS  □ Whats app  □ Snapchat | □ In-clinic visit  □ Home visit  □ School visit  □ Other (specify)________________ |
| --- | --- |

**Contact Attempt Outcome**

1. Specify person contacted AYA or Informant
   1. If AYA contacted, is AYA in care elsewhere?
      1. In care elsewhere? Yes No 🡪 if no go to section D
         1. If yes, specify facility. …………………
            1. Include CCC ID for new facility.
            2. Last viral load result _______
            3. Last viral load date __ __ / __ __ / __ __ __ __

(DD-MM-YYYY)

- - - 1. Is AYA going to continue at new facility?
         1. If yes, first visit and last visit dates

First visit date at new facility __ __ / __ __ / __ __ __ __

(DD-MM-YYYY)

Last visit data at new facility __ __ / __ __ / __ __ __ __

(DD-MM-YYYY)

🡪 **end**

- - - - 1. If no, 🡪 go to section **D**
    1. Not in care elsewhere 🡪 go to section **D**
  1. If informant contacted
     1. Vital status of AYA?
        1. If alive 🡪ii
        2. If died 🡪iv
     2. AYA moved? 🡪
        1. If did not move 🡪iii
        2. If moved, date of move: ___/____/______DD-MMM-YYYY 🡪 **end**
     3. AYA taking medication

If Yes 🡪 the end

If No🡪 go to E

- - 1. If died, date of death: ___/____/______DD-MMM-YYYY 🡪 **end**
  1. Unable to reach AYA or informant 🡪 **end**

**Section D: Assessing AYA’s willingness for IP-NAV engagement:**

AYA response to IP-Nav interaction today

- 1. Yes, the patient agrees go to IP-Nav Intervention 🡪 go to E
  2. No, the patient would like to discuss more before deciding 🡪 go to F
  3. Yes, the patient agrees, but does not have time right now 🡪 go to F
  4. No, the patient refused any further contact with the navigator 🡪 **end**

**Section E: Reviewing Progress from previous interaction**

1. ***Mark based on the participant response depending on which category he/she is referring to (Medical, Psychosocial, Work and Family factor), based on participant’s assessment of progress made. Do not read list to participant.***

Progress status

___Unchanged

___A little progress made

___Some progress made

___Situation resolved

___Other (specify) __________________________

1. **Barrier Assessment**

***Use the table below to assess barriers to care and treatment as part of the intervention form when discussing barriers.***

**Table 1: Assessment of barriers to care and treatment**

| Barriers | | | |
| --- | --- | --- | --- |
| What are your current barriers to receiving care? Mark all barriers you perceive to be present with “X” | | | |
| **Medical**  ___ Felt too sick to come to clinic  ___ Side effects from pills  ___ Pill burden  ___ Want a break from pills  ___ Hospitalized  ___Other medical reason: Specify  **Access to Care**  ___ Transportation problems  ___ Funds to get to the clinic  ___ Didn’t have enough time to for the clinic  ___ Other access to care reasons, specify: | **Clinic Factor**  ___ The clinic ran out of medications  ___ The staff was not nice to the patient  ___ Too many requirements before you are seen by the clinician  ___ The waiting area was uncomfortable  ___The clinic was too crowded  ___ I have to wait too long during visits | **Work and Family**  ___ School/Work interference  ___ No caregiver  ___ Attended funeral  ___ Other family obligation  ___ Parents /guardian/partner didn’t want me to attend clinic  ___Gender based violence  ___Unemployment  ___ Other School/family/work reason, specify:  **Relocated**  ___ Moved/migrated away to another region  Reasons for relocating e.g. school, work, | **Psychosocial**  ___ Felt well, didn’t see need to visit clinic  ___ Disclosure  ___ Afraid clinic would scold me for missing appointment  ___ Because I went to someone who tried / is trying to cure me by prayer / religious rituals.  ___ Because I saw / am seeing a traditional healer instead  ___ I gave up hope because of depression  ___ Alcohol consumption  ___ Forgot appointment  ___Sexual abuse  ___Stigma and discrimination  ___Anxiety  ___Unplanned pregnancy |
| *Additional notes on barriers* | | | |
| *Notes on strengths identified* | | | |

***Consider the following needs for AYAs aged 14 to 19 years.***

1. **Strengthening resiliency**

**Table 2*: Determining interventions for the barrier(s) identified and building a strengthening AYA resiliency***

*The IP-NAV works with AYA to identify interventions that will help the AYA navigate the identified barriers and build a strengthening AYA resiliency, see the table below.*

*Use this form when at the planning stage in the intervention form.*

| **Plan / Response** | | | | | |
| --- | --- | --- | --- | --- | --- |
| *Social support* | *Information* | | *Attitudes and beliefs* | *Building a strengthening AYA Resiliency* | *Material support* |
| ⚪ how to get more social support  ⚪ intervene with influential person  ⚪ intervene with /parent /guardian/spouse  ⚪ Adolescent /couples counseling  ⚪ intervene with church/pastor  ⚪ intervene with police  ⚪ encouragement /support provided by Navigator  ⚪ active listening and empathy  ⚪ Parental love and emotional care  ⚪ Recognition  ⚪ Guidance and protection  ⚪ Love and support from partner/companion  ⚪ active listening and empathy | ⚪ HIV basics  ⚪ consequences of untreated HIV  ⚪ adherence and resistance  ⚪ side effects of ART  ⚪ pre-conception counseling  ⚪ prevention with positive  ⚪ Family planning  ⚪ Cervical cancer screening  ⚪ PreP  ⚪ assisted partner notification  ⚪ Awareness and exposure of sexuality | | ⚪ attitudes about spiritual healing  ⚪ attitudes about herbal remedies  ⚪ patient role  ⚪ denial  ⚪ motivational interviewing | ⚪ Safe disclosure  ⚪ Developing coping strategies to navigate home, school, clinic and any other issues that may arise  ⚪ Reinforce positive living  ⚪ Strengthening emotional/mental well being  ⚪ Engaging in healthy activities like sports, games, dance  ⚪ Fostering supportive relationships  ⚪ Being open minded  ⚪ Building communication skills  ⚪ Being a role model  ⚪ Self-advocacy  ⚪ Planning ahead  ⚪ Goal setting (work, school) | ⚪ School/home delivery of medicine in liaison with clinic  ***logistics at clinic***  ⚪ arrange special time for visit with clinic  ⚪ intervene / mediate with clinic staff after conflict  ⚪ assist with transfer where necessary  ⚪ assist with gaining confidence in clinic navigation and processes.  ⚪ Assisting with support to transition to adult clinic  ⚪ navigation tapering, preparing patient to continue without navigation support |
| *Describe other strategies used:* | | | | | |
| *Additional notes on intervention plan* | | | | | |
| Referrals   - Where referred - Referred for what - Outcome of the referral | | [***Please mark all the referral places the AYA was sent to***  1. SRH Services-   - Family Planning - Cervical cancer screening - Abortion Care - STI Treatment - Others   2. GBV center  3. Partners   - Mwendo - Sunburst   4.CBO’s  ⚪ small cash for Airtime  ⚪ Food  ⚪ Clothing  ⚪ Shelter  ⚪ Medical care  ⚪ Access to secondary or higher education  5. Government education offices  6. Social services office  7. Home/Orphanage | | | |

1. *Was this an intervention session* Yes No
2. *If yes, did you (Nav) fill the Intervention form*? Yes No – explain ____________

**Section F: Plan Documentation**

___Another in-person visit, date specified: _____-________-______

___Another in-person visit planned, no specific date specified

___No future in-person visit planned: communication will continue and home visit if needed

___No future in-person visit planned: communication stopped because patient no longer wants intervention

___No future in-person visit planned: communication stopped because patient is well re-engaged and PAC contact is no longer needed

___Other, specify: ________________________________________________

**IP-NAV Intervention Form**

Date **__ __/__ __/__ __ __ __ (dd/mm/yyyy)**

Study ID

Clinic ID

**Initial Encounter**

My name is ____________ and I am peer navigator for A4A. I’m here to listen and help you with your HIV care journey, which can have easy and hard days. I’m looking forward to talking with you today and in future sessions. I’m going to ask you a few questions. Feel free to respond openly and honestly, the information will not be shared with the clinic or others outside of this study. Please also ask me any questions you may have at any time.

How is your day going?

Tell me a little about yourself?

What support systems do you have to help you with your HIV care?

What support systems do you have in your HIV care?

Follow-up Probes ***(Examine the strengths, resilience and coping mechanisms for the AYA; to include some of the following)***

| Family  Friends (Peers)Support groups  Clinic (Health Workers)  School (Teachers and support staff) | Community  Social Media  Other…. |
| --- | --- |

What are some of the issues you have experienced while accessing HIV care and treatment?

Most people living with HIV know that taking ‘every pill, every day” (good adherence) and attending all their clinic visits is important, but sometimes it is difficult. What are some of the challenges you have with taking your medications or coming to the clinic?

What are the causes of the issues you have identified?

How did you handle the issues you identified?

What are your health/HIV treatment goals for the future?

What are your immediate next steps to address some of the issues you have raised?

What are your general goals for the future?

***Recap with the AYA on the main points covered during the session***

***Review Who, What, When, Where, How of HIV medications and clinic visits****:*

**WHO**-

Who will help you remember to take your medicine every day at the same time?

Who will remind you to come to clinic appointments?

**WHAT**

What medicines are you taking?

What is your dose and how often will you take it?

What will you do when you are about to run out of your medicines?

What will you do if you miss a dose?

**WHEN**

When will you take your medicines?

When is your next clinic visit?

**WHERE**

Where will you take your doses of ARVs (e.g. at school, at home, at work, etc.)?

Where will you store your ARVs?

**HOW**

How will you remember to take your medicines every day and at the same time?

What reminder system will you use?

***Recap with the AYA on the main points covered during the session. Consider AYA needs***. *(****Possible action in italic)***

HIV/ART knowledge-> *p****rovide education, address myths and misconceptions***

ART side effects ***-> connect to clinic provider***

Strengthen support system _> ***connect to OTZ, adolescent peer leads, other support groups***

Disclosure support -> ***discuss approach to disclosure and post-disclosure support***

Food/financial insecurity -> ***referral to OVC or CBO***

Transport -> ***strategize with AYA***

Clinic logistics *->* ***support re-scheduling, coach on effective patient-provider visits***

Adherence*->* ***medication fatigue, forgetfulness, lack of disclosure***

Mental Health or Substance use -> ***provide counseling messages, refer as needed***

***If client is doing well:***

- *Praise them for good job*
- *Encourage the client to reach out and talk to you if there are any problems*
- *Emphasize importance of sharing challenges*

***Plan for next encounter***:

When should we talk again*? (Minimum monthly*)

What is your preferred manner of checking in? *(SMS, WhatsApp, FB, messenger e.t.c)*

***Recap with the AYA on the main points covered during the session. (For the peer lead; pick one or two points discussed above)***

**Subsequent Encounters**

Review discussion during the previous session. Review goal(s)/plans

Examine progress on the plans from last session

Review emerging barriers/challenges to health goal

**Additional Notes**

**IP-Nav Measurement Form**

| Date of form completion  __ __ / __ __ / __ __ __ __ (DD-MM-YYYY) | HH: MM  ⚪ a.m.  ⚪ p.m. |
| --- | --- |

**Section A**: **Administrative**

Clinic ID

Study ID-

**Navigator Initials**

__ __ / __ __ / __ __ __ __ (DD-MM-YYYY)

**Date of re-randomization**

**Type of visit**

**Initial contact at re-randomization (complete B, C, D, and if complete go to E2-5 and F)**

**Follow up contact attempt (complete C, D, E1-5, and F)**

**B. Contact Preferences**

**Preferred contact method, select top three in order of preference**

| □ Phone call  □ SMS  □ Whats app  □ Snapchat | □ In-clinic visit  □ Home visit  □ School visit  □ Other (specify)________________ |
| --- | --- |

**C. Navigator Contact Attempts**

**Number of contact attempts**

**Method of contact attempts for this session**

| □ Phone call  □ SMS  □ Whats app  □ Snapchat | □ In-clinic visit  □ Home visit  □ School visit  □ Other (specify)________________ |
| --- | --- |

**Contact Attempt Outcome**

1. Specify person contacted AYA or Informant
   1. If AYA contacted, is AYA in care elsewhere?
      1. In care elsewhere? Yes No 🡪 if no go to section D
         1. If yes, specify facility. …………………
            1. Include CCC ID for new facility.
            2. Last viral load result _______
            3. Last viral load date __ __ / __ __ / __ __ __ __

(DD-MM-YYYY)

- - - 1. Is AYA going to continue at new facility?
         1. If yes, first visit and last visit dates

First visit date at new facility __ __ / __ __ / __ __ __ __

(DD-MM-YYYY)

Last visit data at new facility __ __ / __ __ / __ __ __ __

(DD-MM-YYYY)

🡪 **end**

- - - - 1. If no, 🡪 go to section **D**
    1. Not in care elsewhere 🡪 go to section **D**
  1. If informant contacted
     1. Vital status of AYA?
        1. If alive 🡪ii
        2. If died 🡪iv
     2. AYA moved? 🡪
        1. If did not move 🡪iii
        2. If moved, date of move: ___/____/______DD-MMM-YYYY 🡪 **end**
     3. AYA taking medication

If Yes 🡪 the end

If No🡪 go to E

- - 1. If died, date of death: ___/____/______DD-MMM-YYYY 🡪 **end**
  1. Unable to reach AYA or informant 🡪 **end**

**Section D: Assessing AYA’s willingness for IP-NAV engagement:**

AYA response to IP-Nav interaction today

- 1. Yes, the patient agrees go to IP-Nav Intervention 🡪 go to E
  2. No, the patient would like to discuss more before deciding 🡪 go to F
  3. Yes, the patient agrees, but does not have time right now 🡪 go to F
  4. No, the patient refused any further contact with the navigator 🡪 **end**

**Section E: Reviewing Progress from previous interaction**

1. ***Mark based on the participant response depending on which category he/she is referring to (Medical, Psychosocial, Work and Family factor), based on participant’s assessment of progress made. Do not read list to participant.***

Progress status

___Unchanged

___A little progress made

___Some progress made

___Situation resolved

___Other (specify) __________________________

1. **Barrier Assessment**

***Use the table below to assess barriers to care and treatment as part of the intervention form when discussing barriers.***

**Table 1: Assessment of barriers to care and treatment**

| Barriers | | | |
| --- | --- | --- | --- |
| What are your current barriers to receiving care? Mark all barriers you perceive to be present with “X” | | | |
| **Medical**  ___ Felt too sick to come to clinic  ___ Side effects from pills  ___ Pill burden  ___ Want a break from pills  ___ Hospitalized  ___Other medical reason: Specify  **Access to Care**  ___ Transportation problems  ___ Funds to get to the clinic  ___ Didn’t have enough time to for the clinic  ___ Other access to care reasons, specify: | **Clinic Factor**  ___ The clinic ran out of medications  ___ The staff was not nice to the patient  ___ Too many requirements before you are seen by the clinician  ___ The waiting area was uncomfortable  ___The clinic was too crowded  ___ I have to wait too long during visits | **Work and Family**  ___ School/Work interference  ___ No caregiver  ___ Attended funeral  ___ Other family obligation  ___ Parents /guardian/partner didn’t want me to attend clinic  ___Gender based violence  ___Unemployment  ___ Other School/family/work reason, specify:  **Relocated**  ___ Moved/migrated away to another region  Reasons for relocating e.g. school, work, | **Psychosocial**  ___ Felt well, didn’t see need to visit clinic  ___ Disclosure  ___ Afraid clinic would scold me for missing appointment  ___ Because I went to someone who tried / is trying to cure me by prayer / religious rituals.  ___ Because I saw / am seeing a traditional healer instead  ___ I gave up hope because of depression  ___ Alcohol consumption  ___ Forgot appointment  ___Sexual abuse  ___Stigma and discrimination  ___Anxiety  ___Unplanned pregnancy |
| *Additional notes on barriers* | | | |
| *Notes on strengths identified* | | | |

***Consider the following needs for AYAs aged 14 to 19 years.***

1. **Strengthening resiliency**

**Table 2*: Determining interventions for the barrier(s) identified and building a strengthening AYA resiliency***

*The IP-NAV works with AYA to identify interventions that will help the AYA navigate the identified barriers and build a strengthening AYA resiliency, see the table below.*

*Use this form when at the planning stage in the intervention form.*

| **Plan / Response** | | | | | |
| --- | --- | --- | --- | --- | --- |
| *Social support* | *Information* | | *Attitudes and beliefs* | *Building a strengthening AYA Resiliency* | *Material support* |
| ⚪ how to get more social support  ⚪ intervene with influential person  ⚪ intervene with /parent /guardian/spouse  ⚪ Adolescent /couples counseling  ⚪ intervene with church/pastor  ⚪ intervene with police  ⚪ encouragement /support provided by Navigator  ⚪ active listening and empathy  ⚪ Parental love and emotional care  ⚪ Recognition  ⚪ Guidance and protection  ⚪ Love and support from partner/companion  ⚪ active listening and empathy | ⚪ HIV basics  ⚪ consequences of untreated HIV  ⚪ adherence and resistance  ⚪ side effects of ART  ⚪ pre-conception counseling  ⚪ prevention with positive  ⚪ Family planning  ⚪ Cervical cancer screening  ⚪ PreP  ⚪ assisted partner notification  ⚪ Awareness and exposure of sexuality | | ⚪ attitudes about spiritual healing  ⚪ attitudes about herbal remedies  ⚪ patient role  ⚪ denial  ⚪ motivational interviewing | ⚪ Safe disclosure  ⚪ Developing coping strategies to navigate home, school, clinic and any other issues that may arise  ⚪ Reinforce positive living  ⚪ Strengthening emotional/mental well being  ⚪ Engaging in healthy activities like sports, games, dance  ⚪ Fostering supportive relationships  ⚪ Being open minded  ⚪ Building communication skills  ⚪ Being a role model  ⚪ Self-advocacy  ⚪ Planning ahead  ⚪ Goal setting (work, school) | ⚪ School/home delivery of medicine in liaison with clinic  ***logistics at clinic***  ⚪ arrange special time for visit with clinic  ⚪ intervene / mediate with clinic staff after conflict  ⚪ assist with transfer where necessary  ⚪ assist with gaining confidence in clinic navigation and processes.  ⚪ Assisting with support to transition to adult clinic  ⚪ navigation tapering, preparing patient to continue without navigation support |
| *Describe other strategies used:* | | | | | |
| *Additional notes on intervention plan* | | | | | |
| Referrals   - Where referred - Referred for what - Outcome of the referral | | [***Please mark all the referral places the AYA was sent to***  1. SRH Services-   - Family Planning - Cervical cancer screening - Abortion Care - STI Treatment - Others   2. GBV center  3. Partners   - Mwendo - Sunburst   4.CBO’s  ⚪ small cash for Airtime  ⚪ Food  ⚪ Clothing  ⚪ Shelter  ⚪ Medical care  ⚪ Access to secondary or higher education  5. Government education offices  6. Social services office  7. Home/Orphanage | | | |

1. *Was this an intervention session* Yes No
2. *If yes, did you (Nav) fill the Intervention form*? Yes No – explain ____________

**Section F: Plan Documentation**

___Another in-person visit, date specified: _____-________-______

___Another in-person visit planned, no specific date specified

___No future in-person visit planned: communication will continue and home visit if needed

___No future in-person visit planned: communication stopped because patient no longer wants intervention

___No future in-person visit planned: communication stopped because patient is well re-engaged and PAC contact is no longer needed

___Other, specify: ________________________________________________

**Appendix B24**

**FOCUS GROUP DISCUSSION GUIDES**

**Electronic navigation (E-Nav)-English**

1. What would you say is the experience of adolescent and young people enrolled in HIV care in this community?
2. What are some of the challenges adolescent and young people face receiving care in this community? Can you describe a case of an adolescent or young person with challenges on ART?
3. Many adolescent and young people are unable to return to clinic for their follow up visits and therefore become lost to programs, what do you think are some of the reasons?
4. How are social support or friendship groups formed? Who constitutes the groups? What things or information is shared and discussed in such groups? How do members of these groups meet/interact?
5. What would be most helpful in a peer supporter?

*Possible probes to include:*

- 1. To give accurate information
  2. To share knowledge
  3. Introduce you to other people living with HIV
  4. Someone to help problem solve my own issues
  5. Someone to advocate for you at clinic
  6. To walk with me to get referral services (counseling, community support, Marie Stopes -IP Nav only)

1. Do you think engaging adolescent and young people in frequent communications through social media messages, SMS and phone calls to discuss their healthcare needs and other challenges can help them remain in care?
   1. How do you think it will help?
   2. Why do you think it will not help?
2. What education and counseling topics do you think will need be covered in automated SMS health messages and phone call or message conversations between navigators (peer support) with adolescent and young people?
3. How frequent should the contact for (1) automated SMS health messages; (2) phone calls or messages with navigators and, (3) face-to-face at clinic be?
4. What do you think should be the content of the automated SMS health messages and phone call or message conversations with navigators who are aiming to encourage adolescent and young people to keep their clinic appointments and adhere to their medication?
5. What characteristics/qualities should a navigator/counsellor that initiates chatting through phone call and messages possess? Gender- or age-merged?
6. What are your favorite Apps for messaging with friends? What are some problems with these messaging Apps that you use? How can these problems be addressed?
7. What language would be most preferred by adolescent and young people for communication? Why do you think this is the case?
8. What is the preferred time(s) for delivering messages or phone calls to adolescents and young adults? Why do you think these are the best times?
9. What is the preferred frequency for delivering messages or phone calls to adolescents and young adults? Why do you think these are the best frequency?
10. What do you think is the best time to start discussions that aim at (1) encouraging adolescent and young people to keep their clinic appointments; (2) support them deal with challenges they go through in an effort to remain in care?
11. How do you think we can reduce the risk of accidental disclosure when using SMS and phone conversations as means of communication?
12. How do you think HIV and HIV-related words should be coded to reduce the risk of inadvertent disclosure?
13. What are your thoughts regarding the acceptable tone for delivering phone calls to adolescents and young people?
14. How do you think an electronic navigator can best support an adolescent through the transition from an adolescent centre to an adult HIV center?
15. What are your additional thoughts about how automated SMS health messages and phone calls and messages with navigators can be used to support adolescent and young people to remain in care?
16. What challenges to you anticipate with automated SMS health messages and communication with navigators through messages and phone calls?

**Conditional cash transfer (CCT)**

1. What would you say is the experience of adolescent and young people enrolled in HIV care in this community?
2. What are some of the challenges adolescent and young people face receiving care in this community?
3. Many adolescent and young people are unable to return to clinic for their follow up visits and therefore become lost to programs, what do you think are some of the reasons?
4. Describe to me the financial situations of young people aged 14-19?
5. Do adolescents age 14-19 have money?
6. Where do they get money? Can they earn money?
7. Is it different for males and females? (chama, merry go round, loan apps)
8. Do they borrow money? Do they have debts?
9. Where do they borrow money?
10. How do they use money?
11. Describe to me the financial situations of young people aged 20-24
    1. Do young people 20-24 have money?
    2. Is it different for males and females? Can they earn money?
    3. Do they borrow money? Do they have debts?
    4. Where do they get money?
    5. How do they use money?
12. If adolescents and young people have money,
    1. How do they share the money?
    2. Who do they share with? Is this different for males and females?
    3. Do parents know when young people have money?
    4. Do spouses/partners/boyfriends/girlfriends know when you have money?
    5. Who makes decisions about how the money is spent?
13. What do you think about giving adolescents conditional cash transfer that motivates appointment attendance and viral suppression? (Probes: How will it help? Why do you think it will not help?)
14. What is the appropriate amount to encourage the behavior (Clinic attendance and viral suppression)?

- For each visit?
- For each viral load result (2 per year)?

b. Why do you think this is an appropriate amount?

1. What concerns do you have about giving adolescents and young people money to support good HIV health outcomes? *Probes- incentive to fail, misuse of money, coercion*

9. To whom do you think this money should be disbursed to (Caregiver or AYA)?

1. Why do you think the money should be disbursed to this person?
2. How do we manage CTCs for younger participants?
3. At what age should we disburse the CCT to caregivers and not AYAs (patient)?
4. If given to the caregivers, how do we ensure that the AYA patient benefits from it?

10. What would be the appropriate means to disburse this money (cash? m-pesa? Save it?) Why do you think so?

1. What do you think can be done if someone comes to collect drugs on the patient’s behalf when a CTC would have been disbursed to the patient?
2. What if the AYA does not have their own phone line or unable to receive the funds directly?
3. Is a savings plan distributed near the end or applied to education, etc. motivating and feasible? Why do you think so?
4. To what extend do you think the money disbursed will be used to support the adolescent and young people to remain in care?
5. How will this money be used to help the adolescent and young people to remain in care?
6. What activities do you think will be supported with this money?
7. What challenges to you anticipate with receiving this money?
8. What are your thoughts about how this money can be better used to support adolescent and young people to remain in care?
9. AYA represent a small population within the larger context of HIV care and treatment, many may know each other through clinic support groups, OTZ, and school. How can we provide CCT without altering people’s behavior because they know somebody is observing themor negative effect in the other arms like SMS or SOC?

**In-person navigation (IP-nav)**

1. What would you say is the experience of adolescent and young people enrolled in HIV care in this community?
2. What are some of the challenges adolescent and young people face while receiving care in this community?
3. Many adolescent and young people are unable to return to clinic for their follow up visits and therefore become lost to programs, what do you think are some of the reasons for this?
4. Do you think assigning peer navigators (peer support) to adolescent and young people to support in navigating the challenges in they encounter and discuss other healthcare needs can help them remain in care?
   1. How do you think this will help?
   2. Why do you think it will not help?
5. What should be the characteristics/qualities of the peer navigator/counsellor? (gender/age)?
6. What education and counseling topics do you think will need be covered when adolescent and young people meet with peer navigators?
7. How frequent should these meetings be? Where should these meetings take place?
8. How long should these meetings be? What should inform the length of these meetings?
9. What is the most appropriate way/means of peer navigators contacting these adolescent and young people?
10. Who within the adolescent and young people households/circles should know about the existence of this peer navigator and their role?
11. How do you think we can reduce the risk of accidental disclosure when peer navigators are trying to contact the adolescent and young people?
12. How do you think an in-person navigator can best support an adolescent through the transition from an adolescent center to an adult HIV center?
13. What are your thoughts about how this in-person peer navigation can be used to support adolescent and young people to remain in care?
14. What challenges do you anticipate with an in-person peer navigator supporting adolescents and young people to remain in care?

**Appendix B25**

**FOCUS GROUP DISCUSSION GUIDES (Kiswahili)**

Miongozo Ya Majadiliano Ya Vikundi

**Urambazaji wa Kielektroniki (E-Nav)**

1. Je, ungesema ni nini mwonjo wa wanaobaleghe na vijana ambao wamesajiliwa katika utunzaji wa HIV katika jamii hii?
2. Ni changamoto zipi wanaobaleghe na vijana wanapitia kwa kupata utunzaji katika jamii hii ? Unaweza kueleza kesi ya anayebaleghe au kijana aliye na changamoto katika ART?
3. Wanaobaleghe wengi na vijana hawawezi kurudi kwa kliniki kwa matembezi yao ya kufuatilia hivyo basi kupotelea miradi, Unafikiria baadhi ya sababu ni zipi?
4. Vikundi vya usaidizi wa kijamii au vya urafiki huundwa vipi? Nani huunda vikundi hivi? Ni mambo au habari gani vikundi kama hivi hushiriki na kujadili? Washiriki wa vikundi hivi hukutana/huingiliana vipi?
5. Je, ni nini kwa msaidizi wa rika ingekuwa ya kusaidia zaidi?
   1. *Chocha za uwezekano ni pamoja na:*
   2. Kutoa habari sahihi
   3. Kushiriki maarifa
   4. Kukujulisha kwa watu wengine wanaoishi na HIV
   5. Mtu wa kusaidia kutatua shida na maswala yangu
   6. Mtu wa kukutetea katika kliniki
   7. Kutembea nami ili nipate huduma za kuelekezwa (ushauri, usaidizi wa kijamii, Marie Stopes -IP Nav pekee)
6. Je, unafikiri kuhusisha wanaobaleghe na vijana katika mawasiliano ya kila mara kupitia ujumbe wa mitandao ya kijamii, ujumbe fupi wa simu na kuwapigia simu kujadili mahitaji yao ya kiafya na changamoto zingine zaweza kuwasaidia kusalia kwa utunzaji?
   1. Unafikiri itasaidia vipi?
   2. Kwa nini unafikiri haitasaidia?
7. Je, unafikiri mada yapi ya elimu na ushauri yatahitajika kupitiwa katika ujumbe fupi wa kiafya ya kujiendesha na kupiga simu au mazungumzo ya ujumbe kati ya wasaidizi (usaidizi wa rika) na wanaobaleghe na vijana?
8. Ni mara ngapi mawasiliano ya (1) ujumbe fupi wa kiafya wa kujiendesha; (2) kupiga simu au ujumbe na wasaidizi na, (3) ana kwa ana katika kliniki zafaa?
9. Je, unafikiri nini inapasa kuwa maudhui katika ujumbe fupi wa kiafya wa kujiendesha na kupiga simu au mazungumzo ya ujumbe na wasaidizi ambao wana nia ya kuwahimiza wanaobaleghe na vijana kuweka miadi yao ya kliniki na kuambatana na dawa zao?
10. Je, msaidizi/mshauri anayeanzisha mazungumzo kupitia kwa kupiga simu na ujumbe anafaa kuwa na tabia/sifa gani? Jinsia au kuunganishwa kiumri?
11. Je, ni zana tumizi zipi za kutuma ujumbe unazopenda na marafiki? Je ni zipi baadhi za shida za hizi zana tumizi za ujumbe unazotumia? Je, hizi shida zinaweza kushughulikiwa vipi?
12. Ni lugha gani ingependelewa zaidi na wanaobaleghe na vijana katika mawasiliano? Kwa nini unafikiri ni hivi?
13. Ni wakati gani unaofaa wa kuwasilisha ujumbe au kupigia simu wanaobaleghe na vijana wazima? Kwa nini unafikiria nyakati hizi ndizo nzuri zaidi?
14. Ni idadi gani ya kuwasilisha ujumbe au kupigia simu wanaobaleghe na vijana wazima ingependelewa? Kwa nini unafikiri hii ndio idadi bora ?
15. Unafikiri ni wakati gani unafaa zaidi kuanzisha majadiliano yanayolenga (1) kuwahimiza wanaobaleghe na vijana kuweka miada yao ya kliniki; (2) kuwasaidia kupambana na changamoto wanazopitia katika juhudi za kusalia kwa utunzaji?
16. Unafikiri tunaweza kupunguza vipi hatari ya kufichuliwa kiajali tunapotumia ujumbe fupi na mazungumzo ya simu kama njia ya mawasiliano?
17. Unafikiria ni vipi HIV na misamiati inayohusiana na HIV inafaa kufichwa ili kupunguza hatari ya yasiyojulikana kufichuliwa?
18. Maoni yako ni yapi kuhusu toni inayokubalika katika kupiga simu kwa wanaobaleghe na vijana?
19. Unafikiria kinyago cha kielektroniki kinaweza kumsaidia vipi kwa njia bora anayebaleghe kupitia ubadilishaji kutoka kwa kituo cha wanaobaleghe hadi kituo cha HIV cha watu wazima?
20. Ni yapi maoni yako ya ziada kuhusu jinsi ujumbe fupi wa kiafya wa kujiendesha na simu, na ujumbe wa wasaidizi yanaweza kutumika kuwasaidia wanaobaleghe na vijana kusalia kwa utunzaji?
21. Unatarajia changamoto zipi na ujumbe fupi wa kiafya wa kujiendesha na mawasiliano wa wasaidizi kupitia ujumbe na kupiga simu?

**Pesa za Uhamishaji wa Kimasharti (CCT)**

1. Ni nini ungesema ni mwonjo wa wanaobaleghe na vijana waliosajiliwa kwa utunzaji wa HIV katika jamii hii?
2. Ni zipi baadhi ya changamoto wanaobaleghe na vijana wanapitia kwa kupata utunzaji katika jamii hii?
3. Wanaobaleghe wengi na vijana hawawezi kurudi kwa kliniki kwa matembezi yao ya kufuatilia hivyo basi kupotelea miradi, Unafikiria baadhi ya sababu ni zipi?
4. Nieleze hali ya kifedha ya vijana wenye umri wa miaka 14-19?
   1. Je, wanaobaleghe wenye umri wa miaka 14-19 wako na pesa?
   2. Wanapata pesa wapi? Wanaweza kupata pesa?
   3. Je, ni tofauti kwa waume na wake? (chama, jasi, zana za mikopo)
   4. Wanakopa pesa? Wako na madeni?
   5. Wanakopa pesa kutoka wapi?
   6. Wanatumia pesa vipi?
5. Nieleze hali ya kifedha ya vijana wenye umri wa miaka 20-24
   1. Je, vijana wenye umri wa miaka 20-24 wako na pesa?
   2. Je, ni tofauti kwa waume na wake? Wanaweza kupata pesa?
   3. Wanakopa pesa? Wako na madeni?
   4. Wanapata pesa wapi?
   5. Wanatumia pesa vipi?
6. Ikiwa wanaobaleghe na vijana wako na pesa,
   1. Wanagawana hizo pesa vipi?
   2. Wanagawana na akina nani? Hii ni tofauti kwa waume na wake?
   3. Wazazi hujua vijana wakiwa na pesa?
   4. Je, wenzi wa ndoa/washirika/marafiki wa kiume/marafiki wa kike hujua ukiwa na pesa?
   5. Nani hutoa uamuzi jinsi pesa hizo zinatumiwa?

7. Unafikiriaje kuhusu kuwapa wanaobaleghe pesa za uhamishaji wa kimasharti zitakazowahamasisha kuhudhuria miadi na kukandamiza virusi? (Chocha: Itasaidiaje? Kwa nini unafikiri haitasaidia?)

1. Ni kiasi kipi kinafaa kuhamasisha tabia (kuhudhuria kliniki na kukandamiza virusi)?

- Kwa kila ziara?
- Kwa kila matokeo ya viwango vya virusi (2 kila mwaka)?

b. Kwa nini unafikiri hikini kiasi mwafaka?

8. Una shaka gani kuhusu kuwapa wanaobaleghe na vijana pesa kusaidia katika matokeo mazuri ya kiafya ya HIV? *Chocha- kichocheo cha kutofaulu, matumizi mabaya ya pesa, kulazimishwa*

9. Unafikiri hizi pesa zinafaa kutumiwa nani (Mtunzaji ama AYA)?

- 1. Kwa nini unafikiri pesa zinafaa kutumiwa huyu mtu?
  2. Tutasimamiaje pesa za uhamishaji wa kimasharti( CCTs) za washirika wachanga?
  3. Tunaweza kutuma pesa za uhamishaji wa kimasharti (CCT )katika umri upi kwa watunzaji na si AYAs (wagonjwa)?
  4. Ikiwa zimepewa watunzaji, tutahakikisha vipi kuwa AYA mgonjwa amefaidika nazo?

10. Ni njia gani ingekuwa mwafaka kutuma pesa hizi (pesa taslimu? m-pesa? Kuweka akiba?) Kwa nini unafikiria hivyo?

1. Unafikiria nini inaweza kufanywa ikiwa mtu atakuja kuchukua dawa kwa niaba ya mgonjwa ikiwa pesa za uhamishaji wa masharti (CCT) zitakuwa zimetumiwa mgonjwa?
2. Je, ikiwa AYA hana laini yake ya simu au hawezi kupokea pesa moja kwa moja?
3. Je, mpango wa akiba inayosambazwa karibu mwisho ama kutumika katika elimu, nk. yachochea na kuwezesha? Kwa nini unafikiria hivyo?

11. Ni kwa kiwango gani unafikiria pesa zilizotumwa zitatumika kusaidia wanaobaleghe na vijana kusalia kwa utunzaji?

11. Hizi pesa zitatumika vipi kusaidia wanaobaleghe na vijana kusalia kwa utunzaji?

12. Ni shughuli gani unadhani pesa hizi zitafadhili?

13. Je, unatarajia changamoto zipi kwa kupokea pesa hizi?

Mawazo yako ni yapi kuhusu jinsi pesa hizi zinaweza kutumika vizuri ili kusaidia wanaobaleghe na vijana kusalia kwa utunzaji?

14. AYA inawakilisha idadi ndogo ya watu katika muktadha mkubwa wa utunzaji wa HIV na matibabu, wengi wanaweza kujuana kupitia kwa vikundi vya usaidizi vya kliniki, OTZ, na shule. Tunawezaje kutoa pesa za uhamishaji wa kimasharti (CCT) bila kubadilisha tabia za watu kwa sababu wanajua mtu anawaangalia au athari hasi katika mikondo mingine kama ujumbe fupi ama SOC?

**Urambazaji wa Kibinafsi (IP-nav)**

1. Ni nini ungesema ni mwonjo wa wanaobaleghe na vijana waliosajiliwa kwa utunzaji wa HIV katika jamii hii?
2. Ni zipi baadhi ya changamoto wanaobaleghe na vijana wanapitia kwa kupata utunzaji katika jamii hii?
3. Wanaobaleghe wengi na vijana hawawezi kurudi kwa kliniki kwa matembezi yao ya kufuatilia hivyo basi kupotelea miradi, Unafikiria baadhi ya sababu ni zipi?
4. Je, unafikiri kuwapa wanaobaleghe na vijana wasaidizi wa rika (usaidizi wa rika) kuwasaidia kuangalia changamoto wanazopitia na kujadili mahitaji mengine ya utunzaji wa afya inaweza kuwasaidia kusalia kwa utunzaji?
   1. Unafikiri hii itasaidia vipi?
   2. Kwa nini unafikiri haitasaidia?
5. Yapi yanafaa kuwa tabia/sifa za msaidizi wa rika/mshauri? (jinsia/umri)?
6. Je, unafikiri ni mada yapi ya elimu na ushauri yatahitaji kushugulikiwa wanaobaleghe na vijana watakapokutana na wasaidizi wa rika?
7. Mikutano hii inafaa kuwa mara ngapi? Mikutano hii inafaa kufanywa wapi?
8. Mikutano hii inafaa kuwa ya muda gani? Ni nini inafaa kufahamisha urefu wa mikutano hii?
9. Ni njia /mbinu gani mwafaka kwa wasaidizi wa rika kuwasiliana na wanaobaleghe na vijana hawa?
10. Nani ndani ya kaya/ duru za wanaobaleghe na vijana anafaa kujua kuhusu kuwepo kwa huyu msaidizi wa rika na jukumu lao?
11. Unafikiri tunaweza kupunguza vipi hatari ya kufichuliwa kiajali wakati wasaidizi wa rika wanajaribu kuwasiliana na wanaobaleghe na vijana?
12. Unafikiri ni vipi msaidizi wa kibinafsi anaweza kusaidia kwa ubora anayebaleghe kupitia ubadilishaji kutoka kwa kituo cha wanaobaleghe hadi kituo cha HIV cha watu wazima?
13. Maoni yako ni yapi kuhusu jinsi huu usaidizi wa rika wa kibinafsi unaweza
    1. kutumika kusaidia wanaobaleghe na vijana kusalia kwa utunzaji?
14. Unatarajia changamoto zipi na msaidizi wa rika wa kibinafsi kusaidia wanaobaleghe na vijana kusalia kwa utunzaji?

**Appendix B26**

**FOCUS GROUP DISCUSSION GUIDES (Dholuo)**

**Electronic navigation (E-Nav)**

1. Ang’ó ma inyalo wacho ni rowere kod jomadongo matindo ma ni e thieth mar kute mag Ayaki neno e oganda ni?
2. Moko kuom pek ma ojende kod jomadongo matindo neno ka gikawo thieth e oganda ni gin mage? Be inyalo pimo kes mar rawera kata ojana man gi pek kuom yedhe magayo kute mag Ayaki?
3. Thoth ojende kod jomatindo madongo ok duog e klinik ne limbe gi moluwo kendo omiyo gilal ne chenro, iparo ni ang’o gini momiyo mae timore?
4. Ere kaka sir mar tudruok kata grube mag osiep iloso? Gin jok mage maloso grube? Gin ang’o kata weche isumo kata twake egrube makamano? Ere kaka jokanyo mag grubegi romo/tudore?
5. Gin ang’o mabiro konyo jakony mar mbese ahinya?
   1. *Gik ma inyalo non oriw:*
   2. Chiwo weche makare
   3. Sumo rieko
   4. Yangi ne jomamoko modak gi kute mag Ayaki
   5. Ng’at ma konya loyo chandruok maga awuon
   6. Ng’at ma chung’ motegno ne raticha eklinik
   7. Mondo owuoth koda mondo ayud kony ma orae (hocho, siri mar oganda, Marie Stopes – Jakony Mbese (IP Nav) kende)
6. Bende iparo ni keto rowere kod jomadongo matindo etudruok ma kinde ka kinde kokalo eyor tudruok mag mbui, ote machwok mag sim kod goyo sim mondo mar twak edwachgi mag thieth kod pek mamoko nyalo konyogi duong’ ethieth?
   1. Ere kaka iparo ni obiro konyo?
   2. Ang’o ma omiyo iparo ni ok bi konyo?
7. Gin weche mage mag puonj kod hocho ma iparo ni onego obed e ote machwok mag thieth ma ioro gi kompyuta kata e sim kata twak e kind mbese ma hoyo ji (sir mbese) kod rowere kod jomatindo?
8. Tudruok mar (1) ote machwok mag thieth ma ioro gi kompyuta (2) goyo sime kata ote ma ioro kod mbese ma hocho kod, (3) wang’ gi wang’ eklinik nyalo bedo moluwore machalo nade?
9. Ang’o ma iparo ni onego obed e ote machwok mag thieth ma ioro gi kompyuta kod wuoyo e sim gi jochiw kony gi hocho ma dwaro jiwo rowere kod jomatindo biro e limbe gi mag klinik mochan kendo mwonyo yedhe gi kaka dwarore?
10. Gin gik mage/kido ma jahocho ma chako wuoyo e sim kata ote machwok onego obedgo? Ma oket machal e kit chuech (dichuo kata dhako) kata higni?
11. Gin gik oro ote mage ma ihero orogo ote ne osiepe? Gin chandruok mage gi gik oro ote ma itiyogo? Ere kaka chandruoge gi inyalo tim?
12. What En dhok mane ma rowere kod jomatindo biro hero ahinya mar tudruok? Ang’o ma omiyo iparo ni en kamano?
13. Gin seche mage ma rawere gi jomadong’o matindo ohero ni oyud ote kata gone gi sim? Ang’o momiyo iparo ni magi ekinde mabeyo mogik?
14. En mang’eny moluwore marom nade ma rowere gi jomadongo matindo ohero ni gi yud ote kata gonegi sim? Ang'o momiyo iparo ni mang’eny moluwore kama emaber mogik?
15. Iparo ni en saa mane maber mogik e chako twak ma dwaro (1) jiwo rowere kod jomatindo e biro e limbe gi mag klinik ma ochan; (2) siro gi mondo gigo pek ma gikale mondo omi gisik ethieth?
16. Ere kaka iparo ni wanyalo dwoko piny thuolo mar both yango chal mar kute duoko seche ma watiyo kod ote machwok kata wuoyo e sim kaka yor tudruok?
17. Ere kaka iparo ni kute mag ayaki kata weche motudore gi kute mag ayaki onego oket mopondo mondo odwok chien thuolo mar bedo gi yango chal mar kute ma ok ochan?
18. In gi paro mage kuom duol mowinjore ne goyo simbe ne rowere gi jomadongo matindo?
19. Ere kaka iparo ni kony mar yor thieth ekit lony masani (elektronik) nyalo siro rawera kowuok ekar thieth mar rowere kadhie kar thieth mar kute mag Ayaki mar jomadongo?
20. Ere pachi ma idwaro medo kuom kaka ote machwok mag thieth ma ioro gi kompyuta kod goyo sime gi ote machwok ma ioro gi johocho inyalo ti godo e jiwo rowere kod jomatindo bedo e thieth?
21. Gin pek mage ma igeno ni nyalo bedo gi ote machwok mag weche thieth ma ioro e sim kod tudruok gi johocho e yor ote machwok kod goyo sim?

**Conditional cash transfer (CCT)**

1. Ang’o ma inyalo wacho ni rowere kod jomatindo man e thieth mar kute mag ayaki kalo e e oganda ni?
2. Ere moko kuom pek ma rowere kod jomatindo neno e yudo thieth e oganda ni?
3. Thoth rowere kod jomatindo ok duog e klinik ne limbe gi moluwo kendo gilal ne chenro , ang’o ma iparo ni miyo mano timore?
4. Pimna chal mar yore mag pesa mar jomatindo man gi higni 14-19?
   - 1. Bende rowere manie higni 14-19 nigi pesa?
   1. Gi yudo pesa kune? Be gi nyalo loso/yudo pesa?
   2. Be opogore kuom chuo gi mon (chama, merry go round, gik kawo ola)
   3. Be giolo pesa? Be gin kod gope?
   4. Gi olo pesa kowuok kanye?
   5. Ere kaka gi tiyo kod pesa?

6. Ka rowere gi jomatindo nigi pesa,

- 1. Ere kaka gipogo pesa?
  2. Gipogo gi ng’a? Bende ma opogore kuom chuo gi mon?
  3. Bende jonyuol ng’eyo ekinde ma jomatindo nigi pesa?
  4. Bende joot/johera/osiepe ma yowuoyi/osiepe ma nyiri ng’eyo kinde ma in gi pesa?
  5. En ng’a mang’ado kaka itiyo gi pesa?

7. Ang’o miparo kuom miyo rowere manyonge michiwo gi chik majiwo biro ekar thieth gi thiro kute? (Non: Ere kaka obiro konyo? Ang’o momiyo iparo ni ok obi konyo?)

1. En omenda marom nade mar jiwo timni (Biro eklinik gi thiro kute)?

- Ne limbe kalimbe?
- Ne duoko kaduoko mar pek mar kute (2 ehiga)?

1. Ang’o momiyo iparo ni mae emanyonge maber kichiwo?

8. Gin weche mage ma in go kuom miyo rowere gi jomatindo pesa mondo osir duoko mabeyo mag thieth? *Non- kony dhi ruombore piny, tiyo gi pesa marach, achune*

9. Iparo ni ng’a ma onego omi pesa (jarit kata AYA)?

- 1. Ang’o ma omiyo iparo ni pesa onego omi ng’ano?
  2. Ere kaka watayo Manyonge Michiwo gi Chik (CCTs) mar jochiwre matindo?
  3. En ehiga mane ma onego wachiw omenda mar CCT ne jochiw rit to ok ne Rowere gi Jomadongo Matindo (AYA) (jatuo)?
  4. Ka omi jochiw rit, ere kaka waneno ni jatuo ma Rawera gi Jomadongo Matindo (AYA) yudo ber mowuok kuome?

10. En yo mane ma nyalo bedo maber e oro pesa ni (pesa aching’? m-pesa?) Ang’o momiyo iparo kamano?

- - 1. Ang’o miparo ni inyalo tim ka ng’ato obiro omo yedhe eloo jatuo ka Omenda michiwo gi chik (CTC) ne onego mi jatuo?
    2. To ka AYA onge gi laini simbe owuon kata ka ok onyal yudo manyonge achiel kachiel?
    3. Bende chenro mar kuno/kungo mochwal machiegni gi giko kata oket eyor somo, gmk. Jipo kendo nyalore? Ang’o momiyo iparo kamano?

11. Iparo ni pesa ma oor no ibiro ti godo maromo nadi e konyo rowere kod jomatindo bedo ethieth?

12. Ere kaka pesa ni ibiro ti godo e konyo rowere kod jomatindo mondo gimed bedo e thieth?

13. Gin gik mage ma iparo ni onego otim kod pesa ni?

14. Gin pek mage ma igeno ne ka ji yudo pesa ni?

15. Ang’o ma iparo kuom kaka pesa ni inyalo ti godo maber e siro rowere kod jomatindo mondo omed bedo ethieth?

16. AYA chung’ eloo kwan oganda matin kuom oganda maduong korka rit gi thieth mar kute mag Ayaki, mang’eny nyalo ng’eyore kokalo e kweth mag siruok, OTZ, kod skul. Ere kaka wanyalo chiwo CCT ma ok wagwenyo timbe ji nikech ging’eyo ni ng’ato ng’iyogi kata gik maricho mawuok kuom bede mmamoko kaka ote machuok kata SOC?

**In-person navigation (IP-nav)**

1. Ang’o ma inyalo wacho ni rowere kod joamtindo ma orwak e thieth mar kute mag ayaki kalo e e oganda ni?
2. Ere moko kuom pek ma rowere kod jomatindo neno ka giyudo thieth e oganda ni?
3. Thoth rowere kod jomatindo ok duog e klinik ne limbe gi moluwo kendo gilal ne chenro, ang’o ma iparo ni omiyo ma timore?
4. Bende iparo ni keto mbese ma hocho ne rowere kod jomatindo mondo osir gi e kalo pek ma giyudo kod twak kod dwaro mag thieth mamoko nyalo konyo gi siko ethieth?
   1. Ere kaka iparo ni ma biro konyo?
   2. Ang’o ma omiyo iparo ni ok obi konyo?
5. Gin gik mage/kido ma onego wang’i kuom ng’ama mbese ma hocho? (kit chuech/higni)?
6. Gin weche mage mag puonj kod hocho ma iparo ni onego owuo e ka rowere gi jomatindo oromo kod mbese ma hocho?
7. Onego bed giromo moluwore maromo nade? Ere kama romo gi onego timre?
8. Romo gi onego obed kuom thuolo maromo nadi? En ang’o ma onego omi wangé thuolo ma romo gi onego okaw?
9. En yo mane maber mogik ma mbese ma hocho onego otudre gi rowere kod jomatindogi?
10. En ng’a e ute ma rowere kod jomatindogo odakie ma onego ong’e ni mbese ma hocho nitie kod tich ma gitimo?
11. Iparo ni wanyalo dwoko chien thuolo mar both yango chal mar kute ka mbese ma hocho temo tudruok kod rowere kod jomatindo?
12. Ere kaka iparo ni ng’at makonyo eyor thieth ma dhano aching’ nyalo siro maber rawera wuoke kar yudo thieth mar kute mag Ayaki mar rowere kodhie mar jomadongo?
13. Pachi gin ang’o kuom kaka mbese ma johocho ka ji romo wang’ gi wang’ aching’ inyalo ti godo eketo rowere gi jomatindo mondo omed bedo e thieth?
14. Gin pek mage ma igeno yudo ka mbese ma hocho wang’ gi wang’siro rowere kod jomatindo mondo omed bedo e thieth?

**Appendix B27**

**IN-DEPTH INTERVIEW GUIDES**

**Electronic navigation (E-Nav)-English**

- **Can you tell me about the experience you have had since you were first enrolled in HIV care?**

Probe:

- - What things have worked well for you to ensure you continue with care?
  - What things have not worked well for you in HIV care?
  - How have you tried to deal with the things that have not worked well to ensure you continue receiving care?
- **You have been receiving** automated SMS health messages and phone call or message conversations with a navigators (peer support) **from the study to help you remain in care and adhere to your medication. Could you tell me what you think about these automated SMS and conversations?**

Probe: timing, frequency, content, tone of voice

- - How do you like the time you receive the study automated SMS and phone calls or messages with the navigator? What do you like specifically about the time you receive SMS and phone calls or messages?
  - How do you like the frequency of the automated SMS and phone calls or messages with the navigator? What do you like specifically about the frequency?
  - How do you like the content of the automated SMS and the phone calls or messages with the navgiator? What do you like specifically about the content?
  - How do you like the tone of voice/attitude of the caller?
- **How has the automated SMS and the phone calls or message conversations with the navigator you have been receiving helped you?**
- Probe for:
  - Reminder for clinic appointments
  - Reminder to take medicine
  - Help navigate healthcare system
  - Build skills and knowledge
  - Help create trust
- **How would you say the automated SMS and phone calls or messages conversations with the navigator have helped you change as a person?**

Probe:

- - In what ways have you changed because of these automated SMS and phone calls or messages with the navigator?
  - In what ways have your circumstances changed because of these automated SMS and phone calls or messages with the navigator?
- **Describe to me how satisfied you are with this program (intervention) that sends the automated SMS and the phone calls or message conversations with the navigator that provide you with information and skills you require to navigate your healthcare.**
- Probe:
  - What specific aspects of the program do you like?
  - What aspects don’t you like? Why?
  - If you were to change the aspects of this program that you don’t like, in what way would you change it?
- **Tell me how this intervention of sending automated SMS and conversing with the navigator over a mobile phone can be made more impactful to help you?**
- **Let’s talk a little more about the peer navigation part of the intervention.**
- **Can you describe to me the relationship you have with this peer navigator?**

**Probe:**

- - Has this relationship changed over time? Tell me more about that.
  - Who, among your family, friends and even workmates, knows about the relationship you have with your peer navigator?
  - Who else would you tell about this peer navigator and the relationship the two of you have?
- **How has the peer navigator assigned to you helped you?**

Probe:

- - Social impact, education achievements (school/college), improved health outcome (clinic attendance, diet, take medicines, refer for other services), re-unit/support other family members.
  - Tell me about any changes you have seen since you started interacting with this peer navigator.
  - How has this interaction with the peer navigator helped you in keeping your clinic appointments and taking your medication?
- **How satisfied are you with this intervention where you are assigned a peer navigator to communicate over the phone to help you navigate your healthcare as well as discuss personal issues?**

Probe:

- - On a scale of 1 (not satisfied) to 10 (very satisfied), how satisfied are you?
  - What made you pick this value?
- **Since you started interacting with this peer navigator, has there been any incidence where you were not satisfied with the way the peer navigator handled the situation? Tell me more about this incidence.**
- **Tell me about how this peer navigator assigned to you can more impactful to you?**
- **Do you have any other thoughts about the automated SMS or peer navigation intervention over the phone?**

**Conditional cash transfer (CCT)**

- **Can you talk to me about the experience you have had since you first enrolled in HIV care?**

Probe:

- - What things have worked well for you to ensure you continue with care?
  - What things have not worked well for you in HIV care?
  - How have you tried to deal with the things that have not worked well to ensure you continue receiving care?
- **You are among the adolescents and young people who were selected to receive some money to help you remain in care. How has it been like receiving this money?**

Probe:

- - How many times have you received this money?
  - How often do you receive the money?
  - Describe to me how you felt like when you received the first disbursement?
  - What did you use this first disbursement for?
- **How has this program of disbursing the money to adolescent and young people helped you?**

Probe for:

- - Social impact, education achievements (school/college), improved health outcome (clinic attendance, food, medicines), support other family members.
  - Tell me about any changes you have seen since you started receiving this money.
  - How has this money helped you in keeping your clinic appointments and taking your medication?
- **How satisfied are you with this program where money is provided to help you navigate your healthcare?**

Probe:

- - On a scale of 1 (not satisfied) to 10 (very satisfied), how satisfied are you?
  - Explain what made you pick this value.
- **Since you started receiving this money, has there been any incidence where you were not satisfied with the way the money disbursed to you was used? Tell me more about this incidence.**
  - Probe:
  - What happened?
  - How else would you want the money used?
- **Tell me about how this money sent to you can be made more impactful in your life.**
- **Do you have any other thoughts about the intervention?**

**In-person navigation (IP-nav)**

- **Can you talk to me about the experience you have had since you first enrolled in HIV care?**

Probe:

- - What things have worked well for you to ensure you continue with care?
  - What things have not worked well for you in HIV care?
  - How have you tried to deal with the things that have not worked well to ensure you continue receiving care?
- **You were assigned a peer navigator by the study to help you navigate your healthcare. Could you tell me more about this person and the relationship you are having?**
- Probe:
  - Nature of relationship; appropriateness of timing and frequency of meetings; content of the discussions during meetings
  - Where and how did you meet for the first time? What was going through your mind before you met for the first time?
  - How long do your meetings usually take?
  - When you meet, what things do you usually discuss?
- **Can you describe to me the relationship you have with this peer navigator?**

**Probe:**

- - Has this relationship changed over time? Tell me more about that.
  - Who, among your family, friends and even workmates, knows about the relationship you have with your peer navigator?
  - Who else would you tell about this peer navigator and the relationship the two of you have?
- **How has the peer navigator assigned to you helped you?**

Probe:

- - Social impact, education achievements (school/college), improved health outcome (clinic attendance, diet, take medicines, refer for other services), re-unit/support other family members.
  - Tell me about any changes you have seen since you started interacting with this peer navigator.
  - How has this interaction with the peer navigator helped you in keeping your clinic appointments and taking your medication?
- **How satisfied are you with this intervention where you are assigned a peer navigator to help you navigate your healthcare as well as discuss personal issues?**

Probe:

- - On a scale of 1 (not satisfied) to 10 (very satisfied), how satisfied are you?
  - What made you pick this value?
- **Since you started interacting with this peer navigator, has there been any incidence where you were not satisfied with the way the peer navigator handled the situation? Tell me more about this incidence.**
- **Tell me about how this peer navigator assigned to you can more impactful to you.**
- **Do you have any other thoughts about the intervention?**

**Appendix B28**

**IN-DEPTH INTERVIEW GUIDES (Kiswahili)**

**Electronic navigation (E-Nav)**

- **Je unaweza kunieleza kuhusu yale ambayo umeona na kupitia tangu uliposajiliwa kwanza kwa matibabu ya virusi vya ukimwi ?**

Probe:

- - Je ni mambo yapi yamefanya kwako vizuri kuhakikisha umeendelea na matibabu?
  - Je ni mambo yapi hayajafanya vyema kwako kwa matbabu yako ya virusi vya ukimwi ?
  - Je ni vipi umejaribu kupambana na vitu ambavyo havijafanya vyema kuhakikisha eundelee kupokea matibabu?
- **Umekuwa ukipokea** arafa za afya zinazotumwa na kompyuta na kupigiwa simu au kuwasiliana kwa njia ya ujumbe fupi na wasaidizi (usaidizi na wanarika) **kutoka kwa utafiti kukusaidia ubaki kwenye matibabu na kumeza dawa zako jinsi inavyotarajiwa . Je unaweza kuniambia ni nini unafikiria kuhusu haya arafa za afya yanatumwa kwa njia ya kompyuta na mazungumzo?**

Probe: timing, frequency, content, tone of voice

- - Je unapenda kiasi gani sa yenye unapokea arafa za afya za utafiti zinazotumwa kwa njia ya kompyuta na kupigiwa simu au kutumiwa arafa na msaidizi? Jin i nini haswa umependa kuhusu saa yeney unapokea arafa fupi na kupigiwa simu au kupata ujumbe?
  - Je umependa kiasi gani marudio ya arafa fupi ya kutumwa kwa njia ya kompyuta na kupigiwa simu au kutumiwa jumbe na msaidizi ? Je ni nini umependa haswa kuhusu marudio hiyo?
  - Je unapenda vipi yaliomo kwenye arafa fupu zinatumwa kwa njia ya kompyuta na kupigiwa simu na ujumbe kutoka kwa msaidizi ? Je ni ni haswa umependa kuhusu yaliomo?
  - Je umependa vipi sauti/mtazamo ya anayepiga simu ?
- **Je ni vipi arafa fupi za simu na kupigiwa simu au ujumbe a arafa na msaidizi yenye umekuwa ukipokea zilikusaidia?**
- Probe for:
  - Kukumbusha tembezi za kliniki zilizopangwa
  - Kukumbusha kuemza dawa
  - Kisaidia kupitia kwa mfumo wa matibabu
  - Kujenga ustadi na maarifa
  - Kusaidi kutengeneza uaminifi
- **Je ungesema vipi arafa fupi za simu zinazotumwa kwa kompyuta na kupigiwa simu au jumbe za mawasiliano na msaidizi yamesaidia kukubadilisha kama mtu binafsi?**

Probe:

- - Je ni kwa njia gani umebadilika kwa sababu ya haya arafa ya kutumwa kwa nji aya kompyuta na kupigiwa simu au jumbe za msaidizi ?
  - Je ni kwa njia gani hali yako imebadilika kwa sababu ya haya arafa fupi zinazotumwa ka njia ya kompyuta na kupigiwa simu au jumbe kutoka kwa msaidizi ?
- **Nieleze uliridhika kwa njia gani ni hii mikakati yenye inatumia arafa fupi inayotumwa na kompyuta na kupigiwa simu au jumbe za mawasiliano na msaidizi mwenye anakupa habari and ustadi yenye unahitaji kupata matibabu yako.**
- Probe:
  - Je ni sehemu gani haswa ya mkakati huu umependa?
  - Je ni sehemu gani hupendi ? Kwa nini?
  - Ikiwa ungebadili sehemu zingine ya huu mradi yneye hupendi , ni kwa njia gani ungeibadili?
- **Nieleze kuhusu mkakati ya kutuma arafa fupi kwa njia ya kompyuta na kuzungumza na msaidizi kwa simu inawezafanywa kuwa na athari mzuri zaidi ya kukusaidia?**
- **Tuzungumze kidogo kuhusu sehemu ya mikakati ya kuingilia kati ya usaidizi ya wanarika**
- **Je unaweza kunielezea husiano uliyo nayo na msaidizi mwanarika?**

**Probe:**

- - Je huu husiano umebadilika kwa muda? Niambie zaidi kuhusu hiyo.
  - Nani, kati ja jamii, marafiki na hata wafanyikazi wenzako , anajua husiano uluyonayo na msaidizi mwanarika?
  - Je ni nani mwingine unaweza kumwambia kuhusu huyu mwanarika msaidizi na husiano iliyoko kati yenu wawili?
- **Je ni vipi msaidizi mwanarika uliyepewa amekusaidia ?**

Probe:

- - Ustawi wa kijamii, kufaulu kielimu (school/college), kuimarika kwa matokeo ya kiafya (kuhudhuria kliniki, utaratibu bora wa mlo, kumeza madawa, kukuelekeza kwa hudumu zingine), kuweka pamoja/kusaidia watu wa familia .
  - Niambie kuhusu mabadiliko yoyote yenye umeona tangu uanze kuhusiana na huyu msaidizi mwanarika.
  - Je ni vipi huu husiano na mwanarika msaidizi imekusaidia kuja kwa tembezi zako za kliniki zenye zimepangwa na kumeza dawa zako?
- **Je umetosheleka ki vipi na mkakati ya kuingilia kati yenye ulipewa mwanarika msaidizi kuwasiliana na wewe kwa simu kukusaidia uweze kupitie mambo za matibabu na kujadili na wewe mamabo yako ya kibinafsi?**

Probe:

- - Katika mizani ya 1 (hujatosheleka) hadi 10 (umetosheleka zaidi ), ulitosheleka vipi?
  - Je ni nini ilifanya ukachagua hii namba?
- **Tangu aunze kuhusiana na huyu mwanarika msaidizi , je kumekuwa na kitu chochote yenye haukutosheleka na njia yenye mwanarika msaidizi alivoshughulikia hii hali? Niambie zaidi kuhusu hii jambo.**
- **Niambie kuhusu jinsi huyu mwanarika msaidizi uliyepewa anaweza kuwa na mabadiliko kubwa kwako?**
- **Je uko na fikira zingine kuhusu mikakati za arafa fupi zinazotumwa na kompyuta au usaidizi ya wanarika kwa simu?**

**Conditional cash transfer (CCT)**

- **Je unaweza kuzungumza na mimi kuhusu yale umepitia tangu usajiliwe kwa matibabu ya virusi vya ukimwi ?**

Probe:

- - J eni vitu gani vilikufanyia vizuri kuhakikisha umeendelea na matibabu?
  - Je ni vitu gani havikufanya vyemo kwako kwa matibabu ya virusi vya ukimwi ?
  - Ni vipi umejaribu kupambana na vitu venye havijafanya vizuri kendelea kupokea matibabu?
- **Wewe ni baadhi ya vijana na vijana wenye wamebaleghe walichaguliwa kupokea pesa kidogo ili uendelee kubaki kwa matibabu. Je imekuwa vipi kupokea hii pesa?**

Probe:

- - Ni mara ngapi umepokea hii pesa?
  - Ni mara ngapi ulipokea hii pesa?
  - Nieleze kuhusu jinsi ulivyohisi utoaji ya kwanza?
  - Je ulitumia hii pesa ya kwanza kwa njia gani?
- **Je ni vipi hii mkakati ya kupeana pesa kwa vijana na vijana wenye wamebaleghe umekusaidia?**

Probe for:

- - Ustawi wa kijamii, kufaulu kielimu (school/college), kuimarika kwa matokeo ya kiafya (kuhudhuria kliniki, utaratibu bora wa mlo, kumeza madawa, kusaidia watu wa familia .
  - Niambie kuhusu mabadiliko yoyote tangu uanze kupokea hii pesa.
  - Ni vipi hii pesa imekusaidia kufika kwa tembezi zako za kliniki na kumeza dawa zako?
- **Je umeridhika ki vipi na mradi huu yenye pesa inapeanwa kukusaidia kupokea matibabu?**

Probe:

- - Kwa mizani ya 1 (hujaridhika) hadi 10 (umeridhika zaidi), umeridhika ki vipi?
  - Eleza ni nini ilifanya uchague hii nambari.
- **Tangu uanze kupokea hii pesa, je kumekuwa na kitu chochote amabapo haikuridhika na jinsi pesa ilipeanwa kwako ilitumika? Nieleze zaidi kuhusu hili jambo.**
  - Probe:
  - Nini ilifanyika?
  - Je ni vipi ungependa hiyo pesa itumike?
- **Niambie kuhusu jinsi hii pesa inatumwa kwako inawekufanywa iwe ya manufaa zaidi kwa maisha yako.**
- **Je uko na maoni zingine kuhusu mkakati huu?**

**Urambazaji wa Kibinafsi (IP-nav)**

- **Unaweza kuzungumza nami kuhusu mwonjo ambao umekuwa nao tangu ujisajili kwa mara ya kwanza katika utunzaji wa HIV?**

Chocha:

- - Ni mambo yapi yamekuendea vizuri kuhakikisha unaendelea na utunzaji?
  - Ni mambo yapi yamekuendea vizuri katika utunzaji wa HIV?
  - Umejaribu kupambana vipi na mambo ambayo hayajakuendea vyema ili kuhakikisha unaendelea kupokea utunzaji?
- **Ulipewa msaidizi wa rika na mradi kukusaidia kudhibiti utunzaji wako wa afya. Je, unaweza kuniambia zaidi kuhusu mtu huyu na uhusiano mlionao?**
- Chocha:
  - Aina ya uhusiano; usahihi wa wakati na marudio ya mikutano; yaliyomo kwenye mahojiano wakati wa mikutano
  - Mlikutana wapi na vipi mwanzoni? Ni yapi yalikuwa yakipita akilini mwako kabla mkutane kwa mara ya kwanza?
  - Mikutano yenu huchukua muda gani kwa kawaida?
  - Mnapokutana, kwa kawaida nyinyi hujadili mambo yapi?
- **Je, unaweza kunielezea uhusiano ulionao na huyu msaidizi wa rika?**

**Chocha:**

- - Je, uhusiano huu umebadilika na wakati? Niambie zaidi kuhusu hayo.
  - Ni nani kati ya familia yako, marafiki, na hata wenzako wa kazini, wanajua kuhusu uhusiano wako na msaidizi wako wa rika?
  - Ni nani mwingine unaweza kumwambia kuhusu huyu msaidizi wa rika na uhusiano mlionao nyinyi wawili?
- **Je, msaidizi wa rika uliyopewa amekusaidia vipi?**

Chocha:

- - Matokeo ya kijamii, mafanikio ya kielimu (shule/chuo), kuimarika kwa matokeo ya kiafya (mahudhurio ya kliniki, maakuli, kumeza dawa, kuelekezwa kwa huduma zingine), kuunganisha tena/kusaidia washiriki wengine wa familia.
  - Niambie kuhusu mabadiliko ambayo umeyaona tangu uanze mwingiliano na huyu msaidizi wa rika.
  - Je, mwingiliano huu na msaidizi wa rika umekusaidiaje kutunza miadi yako ya kliniki na kumeza dawa zako?
- **Umeridhika vipi na uingiliaji kati huu ambapo umepewa msaidizi wa rika akusaidie kusogezea afya yako na kujadili maswala ya kibinafsi?**

Chocha:

- - Katika mizani ya 1 (kutoridhika) hadi 10 (kuridhika sana), umeridhika vipi?
  - Ni nini ilikufanya uchague kiasi hiki?
- **Tangu uanze mwingiliano na huyu msaidizi wa rika, kumekuwa na tukio lolote ambapo hukuridhika na vile msaidizi wa rika alilishughulikia hali hiyo? Niambie zaidi kuhusu tukio hili.**
- **Niambie vile huyu msaidizi wa rika uliyopewa anaweza kuwa na matokeo bora kwako.**
- **Una mawazo mengine yoyote kuhusu kuingilia kati?**

**Appendix B29**

**IN-DEPTH INTERVIEW GUIDES (Dholuo)**

**Electronic navigation (E-Nav)**

- **Bende inyalo nyisa kuom gigo ma iseneno chakre ne orwaki mokwongo e thieth mar kute mag ayaki?**

Probe:

- - Gin gik mae ma osetimore maber ne in mondo one ni imedo dhi nyime gi thieth?
  - Gin gik mage ma ok osetiyo ni maber e thieth mar kute mag ayaki?
  - Ere kaka isetemo timo mondo ikal gigi ma ok osebedo katiyo maber mondo omi idhi nyime gi yudo thieth?
- **Isebedo ka iyudo** ote machwok man gi weche thieth ma oioro gi kompyuta kod sime ma iwuoyo e kata twak kod mbese ma jo hocho e sime utomated SMS health messages and phone call or message conversations with a navigators (sir ma ichiwo gi mbese) k**owuok e nonro mondo okonyi imed bedo e thieth kendo imwony yedhe gi kaka ochan. Nyisa gima iparo kuom ote machwok me ioro gi kompyuta go kod wuoyo ma usebedo ka uwuoyo?**

Probe: timing, frequency, content, tone of voice

- - Ere kaka ihero saa ma iyudo e ote machwok mag nonro ma ioro gi kompyuta kod simbe ma igoyo kata ote machwok ma ioro kod mbese ma hocho?
  - Ere kaka ihero luwruok mar biro ma ote machwok ma ioro gi kompyuta kod sime ma igoyo ni kata ote mag ioro kod mbese ma hocho? En angó ma ihero hie hie kuom kaka gibiro moluwore?
  - Ihero weche ma ni e ote machwok ma ioro gi kompyuta kod sime ma igoyo kod ote ma ioro kod mbese ma hocho? En angó hie hie ma ihero kuom gik ma ni e ote go?
  - Ihero maromo nadi duol mar ng’ama gocho/kaka okawi ?
- **Ere kaka ote machwok ma ioro gi kompyuta kod simbe ma igoyo kod ote ma ioro gi ja hocho ma isebedo ka iyudo osekonyi?**
- Probe for:
  - Poaro ni limbe mochan mag klinik
  - Paro ni mondo imwony yath
  - Konyi yudo kaka iwuotho e yor thieth
  - Medo rieko kod ngéyo
  - Miyo igeno ji
- **Inyalo wacho ni ote machwok ma ioro gi kompyuta kod sime ma igoyo kata ote ma ioro kod jogo ma konyo ji e weche thieth osemiyi iloko ngimani iwuon?**

Probe:

- - E yore mage ma isebedo gi lokruok nikech ote machwok ma ioro gi kompyuta gi kod sime ma igoyo kata ote ma ioro gi jogo ma konyo ji e weche thieth?
  - Gin e yore mage ma ngimani oselokore nikech ote achwok ma ioro gi kompyuta gi kod sime ma igoyo kata ote ma ioro gi jogo ma konyo ji e weche thieth?
- **Nyisa no imor maromo nadi gi chenro ma oro ote machwok ma ioro gi kompyuta kod sime ma igoyo kata ote ma ioro kod ng’ama konyo ji e weche thieth ma miyi weche kod rieko ma idwaro mondo ine kaka iyudo thieth mari.**
- Probe:
  - Gin bath chenro ni mage ma ihero?
  - Gin bathe mage ma ok ihero ? Nang’o?
  - Ka onego ilok bath chenro ni moko ma ok ihero , en yo mane ma inyalo loke godo?
- **Nyisa kaka chenro ni maoro ote machwok e yor kompyuta kod wuoyo gi jal ma konyo ji e weche thieth e simb ong’we yamo nyalo bedo gima konyi ahinya?**
- **Wawuo ane mathoth matin kuom bath chenro mar kony ma ichiwo gi mbese e weche thieth .**
- **Bende inyalo nyisa tudruok ma in go kod mbasni ni ma konyi e weche thieth?**

**Probe:**

- - Bende tudruok ni oselokore kaka thuolo dhi? Nyisa mathoth kuom mano.
  - Ngáno, kuom jo anyuola ni , osiepe kod joma itiyo godo , ma ong’eyo ni in gi mbasni ni ma konyo e weche thieth?
  - En ng’ano ma diher nyiso kuom mbasni ma konyi e weche thieth kod tudruok man e kindu ?
- **Ere kaka mbasni ma konyi e weche thieth ma omiyi osekonyi ?**

Probe:

- - Keto maber ngimani mar tudruok, timo maber e somo (sikul/kolej), dwoko maber mar thieth (dhi e klinik , chiemo maber, mwonyo yedhe, ori ne kony mamoko ), riwo kendo/siro jo anyuola mamoko .
  - Nyisa kuom lokruok ma iseneno chakre ciak tudri kod mabsni ma konyi e weche thieth ni.
  - Ere kaka tudruok ni kod mbasni ma konyi e weche thieth osekonyo dhi e limbe gi mag klinik mochan kendo mwonyo yedhe gi?
- **Imor maromo nadi gi chenro ma imiyi mbasni ma konyi e weche thieth mondo otudre kodi gi sime mondo okonyi kaka iyudo thieth mari kod twak kuom weche gi ma iye ?**

Probe:

- - E ratil mar 1 (ok imor ) nyaka 10 (imor ahinya ), imor maromo nade?
  - What made you pick this value?
- **Chakre ichak tudri kod mbasni ma konyi e weche thieth ni , bende gimoro amora osetimore ma ne ok imor gi kaka mbasni ma konyi e weche thieth ne otimo e thuolo no? Nyisa mathoth kuom gima ne otimore ni.**
- **Nyisa kuom kaka mbasni ma konyi e weche thieth ma omiyi ni nyalo medo kelo lokruok e ngimani ?**
- **Bende in gi paro mamoko kuom ote machwk mag sime ma ioro gi kompyuta kata chenro mar konyo ma mbasni ma konyi e weche thieth e yor sime?**

**Conditional cash transfer (CCT)**

- **Bende inyalo wuoyo koda kuom gik ma iseneno chakre orwaki mokwongo thieth mar kute mag ayaki ?**

Probe:

- - Gin gik mage ma osetiyo maber ne in mondo one ni idhi nyime kod thieth ?
  - Gin gik mage ma ok osetiyo maber ne in e thieth mar kute mar ayaki ?
  - Bende osetemo timo gimoro amora kuom gik ma ok osetiyo maber mondo ine ni idhi nyime yudo thieth?
- **In achiel kuom rowere kod jomatindo mane oyier mondo oyud pesa matin mondo okony gi gibed e thieth. Osebedo machal nadi yudo pesa ni?**

Probe:

- - Iseyudo pesa ni nyadidi?
  - Iyudo ga pesa no moluwore machalo nadi?
  - Nyisa kaka ne iwinjo kane iyudo chudo mokwongo ?
  - Angó mane itimo kod mane iyudo mokwongo?
- **Ere kaka chenro mar chiwo pesa ne rowere kod jomatindo osekonyi?**

Probe for:

- - Keto maber ngimani mar tudruok, timo maber e somo (school/college), keto maber dwoko mag thieth (dhi e klinik, chiemo, yedhe), konyo jomamoko e anyuola u.
  - Nyisa kuom lokruok ma iseneno chakre ichak yudo pesa ni .
  - Ere kaka pesa ni osekonyo e miyo idhi e limbe mochan mag klinik kod mwonyo yedhe gi?
- **Imor maromo nadi kod chenro ni kama pesa ichiwo mondo okonyi kalo e weche thieth ?**

Probe:

- - E ratil mar 1 (ok amor ) nyaka 10 (amor ahinya ), ne imor maromo nadi ?
  - Ler na gima omiyo iyiero namba no.
- **Chakre ichak yudo pesa ni , bende gimor amora osetimore ma ne ok imor godo e yo mane pesa ma omiyi ne oti godo ? Nyisa mathoth kuom gima ne otimore ni .**
  - Probe:
  - Angó mane otimore?
  - Ere yo machielo ma inyalo dwaro mondo pesa no oti godo?
- **Nyisa kaka pesa ma oorni ni inyalo ket mondo okel lokruok maduong’e ngimani.**
- **Bende in gi paro mamoko kuom chenro mar konyo ni ?**

**In-person navigation (IP-nav)**

- **Bende inyalo nyisa kuom gik ma iseneno chakre rwaki mokwongo e thieth mar kute mag ayaki?**

Probe:

- - Gin gik mage ma osetiyo maber ne in mondo one ni imedo dhi nyime gi thieth?
  - Gin gik mage ma pok otiyo ni maber e thieth mar kute mag ayaki ?
  - Ere kaka isetemo tieko gigo ma pok otiyo maber mondo ine ni imedo dhi e thieth?
- **Ne imiyi mbese ma johocho gi nonro mondo okonyi kaka inyalo yudo thieth mari. Bende inyalo nyisa mathoth kuom ng’atni kod tudruok ma un go kode?**
- Probe:
  - Kit tudruok; ber mar seche ma ubedo e romo kod kaka giluwore; gik ma uwuoyo e e kinde romo gi
  - Kanye kod ne uromo kanye mane uromo mokwongo? Ang’o mane obiro e pachi kapok ne uromo mokwongo?
  - Romo u go ne kawo ga thuolo maromo nadi?
  - Ka uromo , gin gik mage mane uwuoyo e ?
- **Bende inyalo lerona tudruok ma un godo gi mbasni ma konyi e weche thieth ni?**

**Probe:**

- - Bende tudruok ni oselokore kaka ndalo dhi ? Nyisa mathoth kuom mano.
  - Ng’ano, kuon jo anyuola ni , osiepe kod jok ma utiyo godo , ma ong’eyo kuom tudruok man e kindi kod mbasni ma konyi e weche thieth?
  - En ng’ano kendo ma diher mar nyiso kuom tudruok man e kindi kod mbasni ma konyi e weche thieth ni ?
- **Ere kaka mbasni ma konyi e weche thieth mane omiyi mondo okonyi ?**

Probe:

- - Lokruok e yor tudruok , timo maber e somo (school/college), dwoko maber mag thieth (dhi e klinik , chiemo maber, mwonyo yedhe , ori ne kony mamoko ), kelo machiegni /siro jomamoko e anyuola.
  - Nyisa kuom lokruok moro amora ma iseneno chakre ichak tudri kod mbasni ma konyi e weche thieth.
  - Ere kaka tudruok ni kod mbasni ma konyi e weche thieth osekonyi e dhi e limbe mochan mag klinik kod mwonyo yedhe gi?
- **Ne imor maromo nadi kod chenro mar konyo ni mane omiyi mbasni ma konyi e weche thieth mondo okonyi e weche thieth kendo otwag kodi e weche ma magi ma iye?**

Probe:

- - E ratil mar 1 (ok amor ) nyaka 10 (amor ahinya), imor maromo nadi ?
  - En ang’o ma omiyo iyiero namba ni ?
- **Chakre ichak bedo kod tudruok gi mbasni ma konyi e weche thieth ni , bende gimoro amora iseneno ma ne ok imor kaka ne mbasni ma konyi e weche thieth ne otimo ? Nyisa mathoth kuom gima ne otimore ni.**
- **Nyisa kaka mbasni ma konyi e weche thieth ma ne omiyi ni nyalo kelo lokruok maduong’ ne in.**
- **Bende in gi paro moko kuom chenro no?**

**Appendix B30**

​​​​**PEER NAVIGATION INTERVIEW & FOCUS GROUPS GUIDES**

**With A4A participants (adolescents and young adults living with HIV), Peer Navigators, and clinic and research staff**

**1. Interview with A4A Participants who are receiving or have received Peer Navigation as part of the A4A Study.**

**I. INTRODUCTION**

My name is ___________________. I am working with the Kenya Medical Research Institute on a project aiming to improve HIV outcomes among adolescents and young adults living with HIV here in Kenya.

We would like to talk to you about your to understand your experience with Peer Navigation. Specifically, we would like to understand  things that you liked or didn’t like, things that were helpful or unhelpful, and suggestions you may have to improve the intervention.  We also want to understand how Peer Navigation  might help adolescents and young adults deal with stress, take their medications, and return to the health care facility for ongoing medical care. Everything you share during this interview will be kept confidential. The information that you provide will be used to inform efforts to strengthen Peer Navigation in the future.

Remember, you don’t have to talk about anything you don’t want to and you may stop participating at any time. This discussion will take around one and a half hours. If you have questions you want to ask on other topics, I can assist you to find answers after the interview is over.

(Go through consent form if not already done. Ask permission to audio record the discussion, and if they agree, start the audio recorder AFTER the introductions.  This guide includes the topics to be covered and questions that may be helpful in facilitating the interview. You do NOT have to ask all the questions or follow the order given in the guide. Major topic areas and questions are indicated.

**II. DISCUSSION TOPICS**

**Peer Navigation Exposure**

- Did you receive Peer Navigation in-person, by phone, or both?
- How long had you been taking ART when you started receiving Peer Navigation?
- About how many months did you receive Peer Navigation?

**PEER NAVIGATION Experience**

- Did you find PEER NAVIGATION helpful? If yes, how so? If no, why not?
- What parts of PEER NAVIGATION did you find most helpful?
- Were their topics or other things that were not included that you think should be?
- What is the most important skill you think you learned as part of PEER NAVIGATION?
- Did anything good happen as a result of your participation in PEER NAVIGATION? If so, please explain?
- Did you include your partner, parent, or another person in any of your PEER NAVIGATION sessions? Why or why not?
- If yes, who did you included and how was that experience?
- Was there anything about PEER NAVIGATION that was not helpful or that was harmful to you? If so, please describe?
- Did you have any concerns about HIV disclosure related to PEER NAVIGATION? If so, please explain.
- Did any issues arise about HIV disclosure due to your participating in PEER NAVIGATION? If so, please explain.
- Did anything else happen as a result of PEER NAVIGATION that you did not like? If so, please explain.
- Did you experience any challenges with taking your medications, missing clinic visits, or having a high viral load before or while receiving PEER NAVIGATION? If so, did PEER NAVIGATION help? Why or why not?
- Did you experience any challenges with your mood or stress before or while you were receiving PEER NAVIGATION If so, did PEER NAVIGATION help? Why or why not?
- Were you referred to a counselor or someone else by your PEER NAVIGATION Helper? If so, how was this experience? Did you get the help you needed?
- Would you recommend PEER NAVIGATION to others?

**PEER NAVIGATOR experience**

- How did you like working with a PEER NAVIGATOR ?
- Did you trust your PEER NAVIGATOR Helper? Why or why not?
- What things did your PEER NAVIGATOR do or not do that affected your trust in them?
- What would you change about your PEER NAVIGATOR if you could?

**PEER NAVIGATION Delivery Format**

- If you received PEER NAVIGATION in-person, what did you like most about meeting a PEER NAVIGATOR in-person?
- If you received PEER NAVIGATION by phone, what did you like most about mobile PEER NAVIGATION?
- What did you like the least about in-person or mobile PEER NAVIGATION?
- How would you improve or change the way PEER NAVIGATION is delivered?

**IV.  CLOSING**

Thank you very much for your time. Your responses will be very helpful for improving PEER NAVIGATION for other women in the future.

(Correct any important misconceptions and provide referrals to PMTCT or HIV services, if appropriate.)

**2. Interview with A4A Peer Navigators who are or have worked as a Peer Navigator as part of the A4A Study.**

**2. INTRODUCTION**

My name is ___________________. I am working with the Kenya Medical Research Institute on a project aiming to improve HIV outcomes among adolescents and young adults living with HIV here in Kenya.

We would like to talk to you about your to understand your experience delivering Peer Navigation. Specifically, we would like to understand  things that you liked or didn’t like, things that were helpful or unhelpful, and suggestions you may have to improve the intervention.  We also want to understand how Peer Navigation  might help adolescents and young adults deal with stress, take their medications, and return to the health care facility for ongoing medical care. Everything you share during this interview will be kept confidential. The information that you provide will be used to inform efforts to strengthen Peer Navigation in the future.

Remember, you don’t have to talk about anything you don’t want to and you may stop participating at any time. This discussion will take around one and a half hours. If you have questions you want to ask on other topics, I can assist you to find answers after the interview is over.

(Go through consent form if not already done. Ask permission to audio record the discussion, and if they agree, start the audio recorder AFTER the introductions.  This guide includes the topics to be covered and questions that may be helpful in facilitating the interview. You do NOT have to ask all the questions or follow the order given in the guide. Major topic areas and questions are indicated.

**II. DISCUSSION TOPICS**

**Peer Navigation Exposure**

- For how long have you worked as a Peer Navigation (months/years)?
- Did you have prior experience as a peer navigator/peer educator before becoming a peer navigator?

**PEER NAVIGATION Experience**

- Do you think Peer Navigation was helpful for AYA? If yes, how so? If no, why not?
- What parts of Peer Navigation do you think were most helpful?
- Were their topics or other things that were not included that you think should be?
- What is the most important skill you think AYA learned as part of Peer Navigation?
- Share a story of a good outcome for a participant that was a result of Peer Navigation (your case or someone else)?
- How often did Peer Navigation include a partner, parent, or another person? Was it helpful to involve other people? Why or why not?
- Share a story of a unsuccessful outcome for a participant that was a result of Peer Navigation (your case or someone else)?
- Was there anything about Peer Navigation that was not helpful or that was harmful to participants? If so, please describe?
- Did any issues arise about HIV disclosure due to Peer Navigation that you are aware of? If so, please explain.
- Did anything else positive or negative happen as a result of Peer Navigation? If so, please explain.
- For AYA experiencing challenges with taking medications, missing clinic visits, or having a high viral load, how did Peer Navigation help or not help?
- For AYA experiencing challenges with mood, stigma, or stress, did Peer Navigation help? Why or why not?
- What AYA challenges did Peer Navigation address best?
- What AYA challenges were most difficult for Peer Navigation to address?
- What were benefits and challenges of electronic peer navigation as compared to in-person navigation?
- Would you recommend Peer Navigation to others?

**PEER NAVIGATOR experience**

- How did you like working as a Peer Navigator ?
- What parts of peer navigator training were most helpful?
- How do you think the initial training for Peer Navigators could be improved?
- What parts of ongoing training/debriefs were most helpful?
- How do you think ongoing training/debriefs for Peer Navigators could be improved?
- Did you feel supported as a Peer Navigator? Please explain why or why not?
- What more could be done to support Peer Navigators like you?
- Did anything good happen to you personally as a result of your work as a Peer Navigator? If so, please explain?
- What would you change about working as a Peer Navigator if you could?
- What else would you like to share about your work as a Peer Navigator?

**IV.  CLOSING**

Thank you very much for your time. Your responses will be very helpful for improving PEER NAVIGATION for other AYA in the future.

(Correct any important misconceptions and provide referrals, if appropriate.)

**3. FOCUS GROUP PARTICIPANT CHARACTERISTICS FORM**

PLACE:

MODERATOR:

NOTE TAKER:

DATE:

Beginning time: Ending time:

TYPE OF GROUP:

CHARACTERISTICS OF PARTICIPANTS

|  | 1 | 2 | 3 | 4 | 5 | 6 | 7 |
| --- | --- | --- | --- | --- | --- | --- | --- |
| Age |  |  |  |  |  |  |  |
| Gender |  |  |  |  |  |  |  |
| Educational Level |  |  |  |  |  |  |  |
| Current job / role |  |  |  |  |  |  |  |
| Experience working in PMTCT/ANC/HIV? (years) |  |  |  |  |  |  |  |
| Experience working with PEER NAVIGATION (months) |  |  |  |  |  |  |  |

**3. Focus Group Discussion (clinic and research staff)**

**I. INTRODUCTION**

My name is ___________________. I am working with the Kenya Medical Research Institute on the A4A Study. We are currently working on a project aimed at understanding how Peer Navigation implementation worked and how the intervention can be improved.

We would like to talk to you as someone who was involved in Peer Navigation implementation. We think that you may be a good source of information on how Peer Navigation works or needs to be improved.

Everything you share during this focus group will be kept confidential. The information that you provide will be used to inform efforts to improve Peer Navigation for youth with HIV in Kenya.

Remember, you don’t have to talk about anything you don’t want to and you may stop participating at any time. This discussion will take around one and a half  to two hours. If you have questions you want to ask on other topics, I can assist you to find answers after the focus group discussion is over.

(Complete consenting if not already done. Ask permission to audio record the discussion, and if they agree, start the audio recorder AFTER the introductions part of the discussion. This guide includes the topics to be covered and questions that may be helpful in facilitating the focus group discussion. You do NOT have to ask all the questions or follow the order given in the guide. Major topic areas and questions are indicated.

**II. INTRODUCTIONS**

First let’s get acquainted.  Let’s go around the circle and each person can introduce themselves. You can tell us your first name (or name you would like to use in this group discussion), what type of service provider you are, and anything else about yourself that you would like to tell the group.  (If the group agreed to the tape recording, you may start recording after this section of the discussion.)

**III. DISCUSSION TOPICS**

We implemented Peer Navigation with adolescents and young adults living with HIV (AYA). We are interested in your perceptions of how Peer Navigation implementation went.

- What worked well?
- Did Peer Navigation seem to help AYA? If so, in what ways?
- Did Peer Navigation seem to improve medication adherence and HIV outcomes?
- Did Peer Navigation seem to improve mental well being?
- If Peer Navigation was beneficial, how do you think it helped women?
- Peer Navigation was delivered in-person and as mobile Peer Navigation? What were benefits and challenges of these different modes of delivery?
- How effective were Peer Navigators? Why/why not were they effective?
- What didn’t work well?
- What were challenges to implementation that arose?
- What needs to be changed?
- What is missing?
- Did any unexpected benefits to Peer Navigation? For AYA? For the clinic/staff?
- Where their any unexpected adverse effects of Peer Navigation? For AYA? For the clinic/staff?
- How else could we improve Peer Navigation?
- Would you recommend continuing Peer Navigation? How would it be sustained at the clinic?

**IV.  CLOSING**

Thank you very much for your time. Your responses will be very helpful for improving the health of Kenyan families.

(Correct any important misconceptions and provide referrals, if appropriate.)

**Appendix B31**

**Appendix B32**

**Appendix B33**

**Recruitment Script for Aim 3 Interviews and Focus Group Discussion**

**Instructions:** This script is to be used by study staff for recruitment of study participants on the phone, when recruiting eligible participants to participate in a focus group discussion.

Section A: A4A Participants

Section B: Peer Navigators

Section C: Clinic or Research Staff

********************************************************************************************************************************

**Section A:**

**Phone:** “Hello, my name is ________. I work with the Kenya Medical Research Institute, and I am calling about a research study that you may be eligible to participate in. You have been a participant in the A4A Study who has received peer navigation either by phone, in-person, or both. We would like to understand your experiences and thoughts about peer navigation by doing an interview with you. We also want to know about things you would change to improve peer navigation. If you are interested in learning more about the study, I can arrange a time for you to meet with someone from the study. If you are not interested in the study, that is fine and no one from the study will contact you about it in the future. Your participation in the study will not affect the care you receive at the clinic.

Are you interested in learning more about this study?”

********************************************************************************************************************************

**Section B:**

**Phone:** “Hello, my name is ________. I work with the Kenya Medical Research Institute, and I am calling about a research study that you may be eligible to participate in if you agree. You have been a Peer Navigator as part of the A4A Study. We would like to understand your experiences and thoughts about peer navigation by doing an interview with you. We also want to know about things you would change to improve peer navigation. If you are interested in learning more about the study, I can arrange a time for you to meet with someone from the study. If you are not interested in the study, that is fine. Your participation in the study will not affect the your work on the A4A Study.

Are you interested in learning more about this study?”

*********************************************************************************************************************************

**Section C:**

**Phone:** ““Hello, my name is ________. I am working with the Kenya Medical Research Institute on a study to learn more about how peer navigation might improve HIV outcomes among adolescents and young adults living with HIV here in Kenya. This purpose of this study is learn more about the opinions and experiences of health care providers and researchers implementing peer navigation for youth in Kenya. If you agree to join the study, you will be asked to participate in a meeting with up to 8 other people. The meeting may be conducted in-person, or virtually (e.g., via Zoom) and will last for about 1.5-2 hours and will be audio-recorded. If you are interested in learning more about the study, I can share more information now or at a later time. If you are not interested in the study, that is fine and no one from the study will contact you about it in the future. Your participation in the study will not affect your work at the clinic or with the A4A Study.

Are you interested in learning more about this study?”

**Email:** Dear [Participant name or relevant affiliation],

We invite you to participate in an important study to learn more about the intervention called Problem Management Plus and how it supports pregnant and postpartum women living with HIV who are at risk of HIV care disengagement and treatment failure. Additional details of this study are provided in the consent form attached to this email.

If you choose to participate in this study, you will be asked to participate in a focus group discussion with about 8 other people.

For the discussion:

• You will meet in-person or virtually (e.g., via Zoom)

• The discussion will last approximately 1.5-2 hours

• It will be audio-recorded

• You will receive a KSh 2000 reimbursement

Thank you in advance for contributing to this important effort. Please respond to this email to note your interest in participating, or to request further information.  We are happy to answer any questions you may have.

[Letter will be signed by the research team]

**Appendix B34**

**Appendix B35**

**Appendix B36**

A4A_incentives_DCE

Start

Introduction
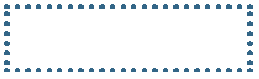


Welcome to the A4A ADAPT for Adolescents survey. Your responses will help us determine what incentives youth most prefer. There are 21 question in total.

The first 12 survey questions will ask about the characteristics of the AYA surveyed. The next 9 survey question will ask the AYA to choose between two clinics with different incentive strategies.

Next

DateDCEAdministered
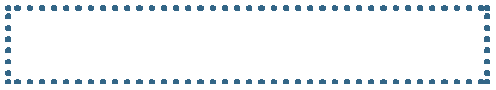


Date DCE was administered to AYA

DateDCEAdministered_UserInput(hidden) DateDCEAdministered_Unix(hidden)

DateDCEAdministered_Year(hidden) DateDCEAdministered_Month(hidden)
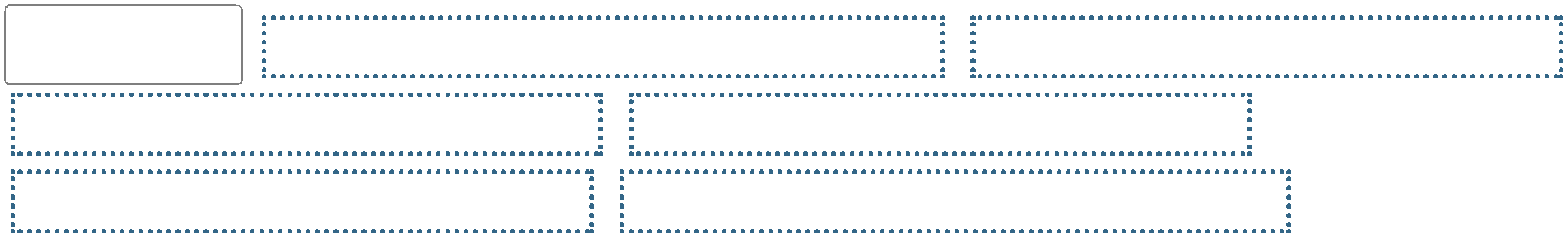


DateDCEAdministered_Day(hidden) DateDCEAdministered_Readable(hidden)

Back Next

0% 100%

RAadministrator
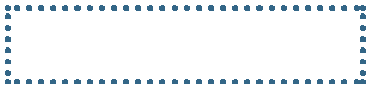


Initials of RA administrator

Back Next

0% 100%

Did the AYA consent to participating in the survey?

Consent=1
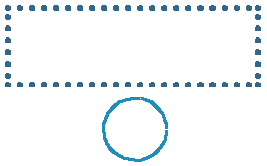


Yes

Consent=2
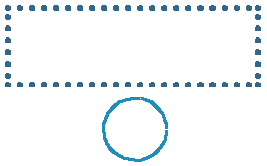


No

Back Next

0% 100%

Please fill out the AYA's name below

First Name

MiddleName
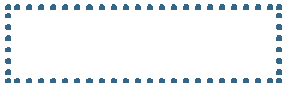


Middle Name

LastName
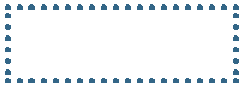


Last Name

Back Next

0% 100%

Clinic ID of AYA

-

Back Next

0% 100%

What is the AYA's date of birth? (If not known please leave the question blank)


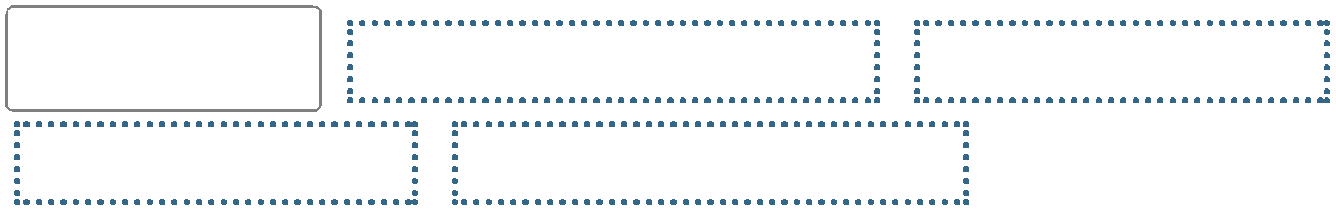
DOB_UserInput(hidden) DOB_Unix(hidden) DOB_Year(hidden) DOB_Month(hidden)
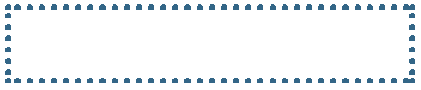

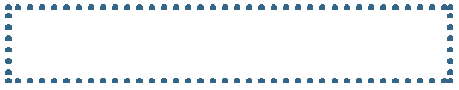


DOB_Day(hidden) DOB_Readable(hidden)

Back Next

0% 100%

How many years old is the AYA today?

Age=1

14-17 years

Age=2

18-21 years

Age=3

21-24 years

Back Next

0% 100%

Gender of AYA

Gender=1
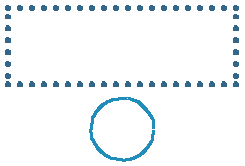


Female

Gender=2
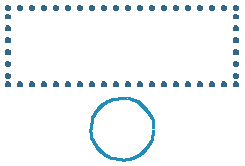


Male

Gender=3
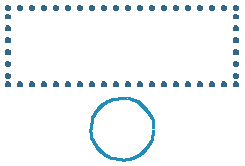


Other/non-conforming

Back Next

0% 100%

Is the AYA currently in school?

SchoolingStatus=1
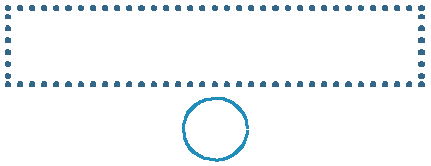


Yes


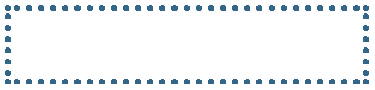


SchoolingStatus=2
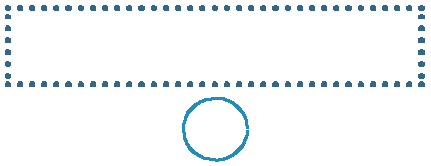


No

Back Next

0% 100%

What level of school is the AYA currently enrolled in?

SchoolLevel=1
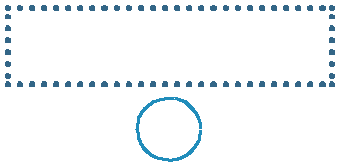


Primary School

SchoolLevel=2
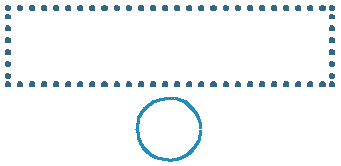


Secondary School

SchoolLevel=3
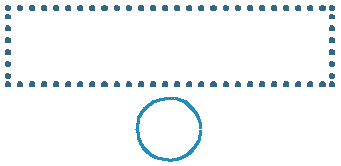


University


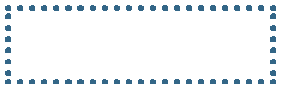


Back Next

0% 100%

Where does the AYA and there family/caregivers live?

UrbanRural=1
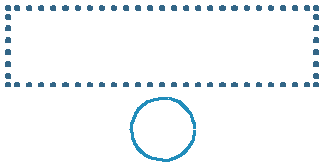


In Kisumu town

UrbanRural=2
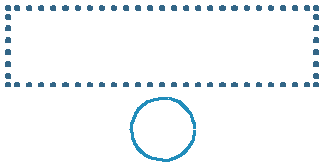


Outside Kisumu town

UrbanRural=3 UrbanRural_3_other
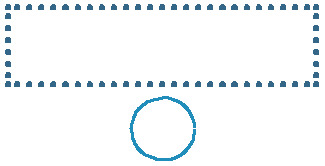

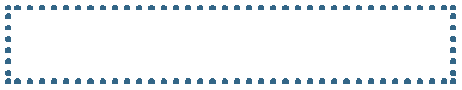


Other (please specify)

Back Next

0% 100%

In the last 12 months how often did the AYA's household not have enough food?

SocioeconomicStatus=1
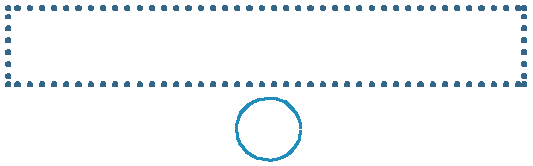


Never

SocioeconomicStatus=2
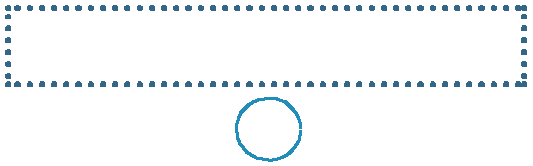


Sometimes

SocioeconomicStatus=3
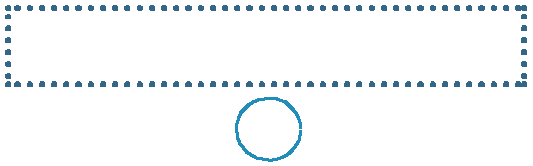


Always


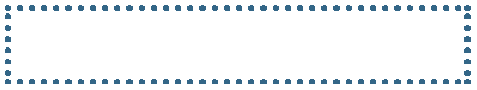


Back Next

0% 100%

What are the number of rooms used for sleeping in the AYA's residence?

SESQ2=1
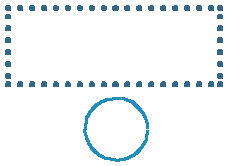


One

SESQ2=2
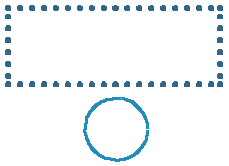


Two

SESQ2=3
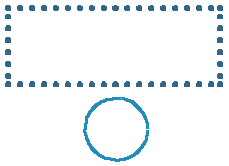


Three or more

Back Next

0% 100%

Transition
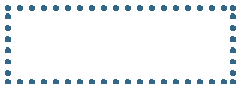


You will now present the choice experiment options to the AYA by asking them to select between two clinics with different incentive strategies.

Back Next

0%

100%

If you could choose to attend one of two clinics that provide monetary gifts at

clinic visits, which of these two clinics would you choose?

Clinic A

Clinic B

When you receive the gift

Who is eligible for the gift

Who collects the gift

The value of the gift

How is the gift distributed

At each clinic visit

Only youth who attend clinic visits on time & are virally suppressed

Only you


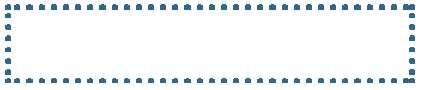


100 KSH

mPesa mobile payment

At the end of each year (saved at each clinic visit)

Only youth who attend clinic visits on time & are virally suppressed

You or a person you have elected

(e.g. spouse or caregiver)

500 KSH

Cash

A4ADCE_Random1

Select

A4ADCE_Random1

Select


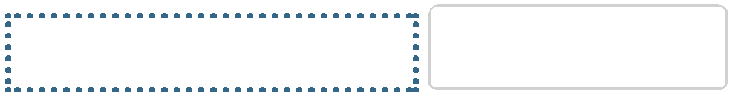


(1 of 9)

Back Next

If you could choose to attend one of two clinics that provide monetary gifts at

clinic visits, which of these two clinics would you choose?

Clinic A

Clinic B

When you receive the gift

Who is eligible for the gift

Who collects the gift

The value of the gift

How is the gift distributed

At each clinic visit

All youth attending the ART clinic

Only you


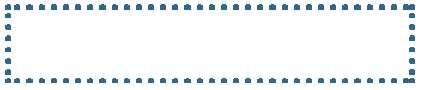


500 KSH Airtime

At the end of each year (saved at each clinic visit)

All youth attending the ART clinic

You or a person you have elected

(e.g. spouse or caregiver)

300 KSH

Tusky's shopping voucher (non- expiring)

A4ADCE_Random2

Select

A4ADCE_Random2

Select


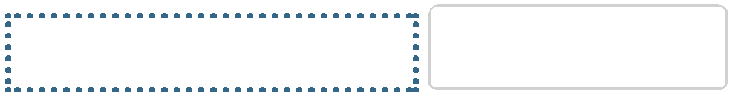


(2 of 9)

Back Next

If you could choose to attend one of two clinics that provide monetary gifts at

clinic visits, which of these two clinics would you choose?

Clinic A

Clinic B

When you receive the gift

Who is eligible for the gift

Who collects the gift

The value of the gift

How is the gift distributed

At the end of each year (saved at each clinic visit)

Only youth who attend clinic visits on time & are virally suppressed

Only you


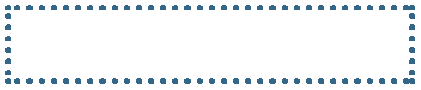


100 KSH Airtime

At the end of each year (saved at each clinic visit)

All youth attending the ART clinic

Only you

300 KSH Cash

A4ADCE_Random3

Select

A4ADCE_Random3

Select


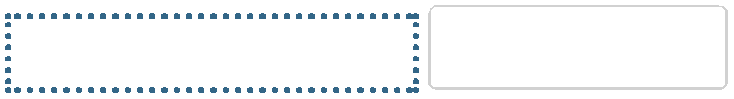


(3 of 9)

Back Next

If you could choose to attend one of two clinics that provide monetary gifts at

clinic visits, which of these two clinics would you choose?

Clinic A

Clinic B

When you receive the gift

Who is eligible for the gift

Who collects the gift

The value of the gift

How is the gift distributed

At each clinic visit

All youth attending the ART clinic

You or a person you have elected

(e.g. spouse or caregiver)


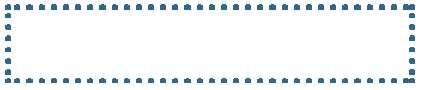


500 KSH

Airtime

At each clinic visit

Only youth who attend clinic visits on time & are virally suppressed

You or a person you have elected

(e.g. spouse or caregiver)

100 KSH

Cash

A4ADCE_Random4

Select

A4ADCE_Random4

Select


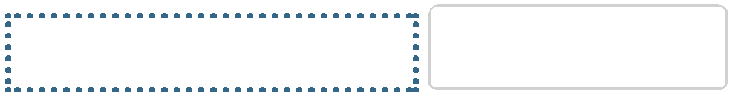


(4 of 9)

Back Next

If you could choose to attend one of two clinics that provide monetary gifts at

clinic visits, which of these two clinics would you choose?

Clinic A

Clinic B

When you receive the gift

Who is eligible for the gift

Who collects the gift

The value of the gift

How is the gift distributed

At each clinic visit

All youth attending the ART clinic

You or a person you have elected

(e.g. spouse or caregiver)


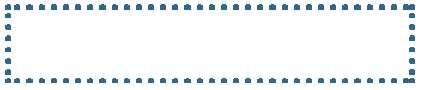


500 KSH

mPesa mobile payment

At each clinic visit

Only youth who attend clinic visits on time & are virally suppressed

Only you

300 KSH

mPesa mobile payment

A4ADCE_Random5

Select

A4ADCE_Random5

Select


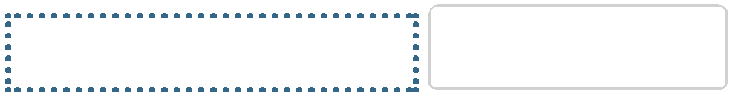


(5 of 9)

Back Next

If you could choose to attend one of two clinics that provide monetary gifts at

clinic visits, which of these two clinics would you choose?

Clinic A

Clinic B

When you receive the gift

Who is eligible for the gift

Who collects the gift

The value of the gift

How is the gift distributed

At the end of each year (saved at each clinic visit)

All youth attending the ART clinic

Only you


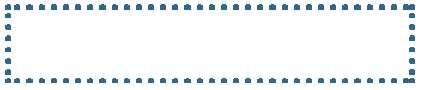


100 KSH Airtime

At the end of each year (saved at each clinic visit)

Only youth who attend clinic visits on time & are virally suppressed

You or a person you have elected

(e.g. spouse or caregiver)

500 KSH

Tusky's shopping voucher (non- expiring)

A4ADCE_Random6

Select

A4ADCE_Random6

Select


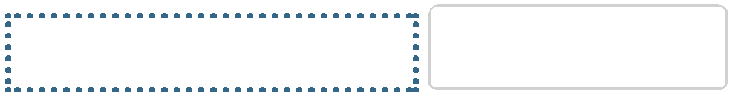


(6 of 9)

Back Next

If you could choose to attend one of two clinics that provide monetary gifts at

clinic visits, which of these two clinics would you choose?

Clinic A

Clinic B

When you receive the gift

Who is eligible for the gift

Who collects the gift

The value of the gift

How is the gift distributed

At the end of each year (saved at each clinic visit)

Only youth who attend clinic visits on time & are virally suppressed

You or a person you have elected

(e.g. spouse or caregiver)


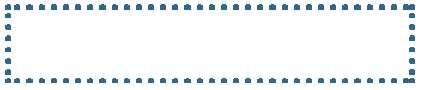


300 KSH

Airtime

At each clinic visit

All youth attending the ART clinic

Only you

300 KSH

Tusky's shopping voucher (non- expiring)

A4ADCE_Random7

Select

A4ADCE_Random7

Select


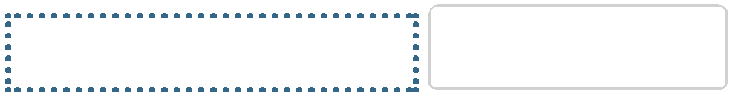


(7 of 9)

Back Next

If you could choose to attend one of two clinics that provide monetary gifts at

clinic visits, which of these two clinics would you choose?

Clinic A

Clinic B

When you receive the gift

Who is eligible for the gift

Who collects the gift

The value of the gift

How is the gift distributed

At each clinic visit

All youth attending the ART clinic

You or a person you have elected

(e.g. spouse or caregiver)


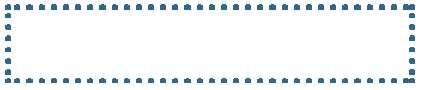


100 KSH

Cash

At each clinic visit

Only youth who attend clinic visits on time & are virally suppressed

Only you

500 KSH

Tusky's shopping voucher (non- expiring)

A4ADCE_Random8

Select

A4ADCE_Random8

Select


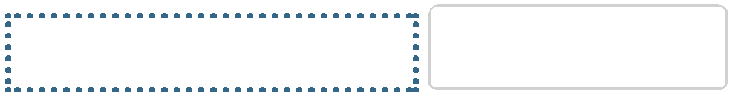


(8 of 9)

Back Next

If you could choose to attend one of two clinics that provide monetary gifts at

clinic visits, which of these two clinics would you choose?

Clinic A

Clinic B

When you receive the gift

Who is eligible for the gift

Who collects the gift

The value of the gift

How is the gift distributed

At the end of each year (saved at each clinic visit)

All youth attending the ART clinic

Only you


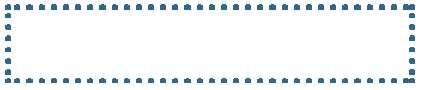


500 KSH Cash

At the end of each year (saved at each clinic visit)

Only youth who attend clinic visits on time & are virally suppressed

You or a person you have elected

(e.g. spouse or caregiver)

100 KSH

Tusky's shopping voucher (non- expiring)

A4ADCE_Random9

Select

A4ADCE_Random9

Select


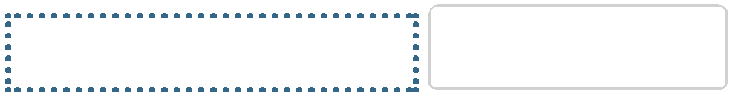


(9 of 9)

Back Next

Ending

Thank you for taking this survey.

0% 100%

Disqualified
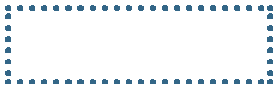


Thank you for taking this survey. Unfortunately, if the AYA does not consent to participating they are disqualified from completing the survey.

Back Next

0% 100%

DCE DATA COLLECTION FORM-KISWAHILI

Anza

Utangulizi

### Karibu kwenye A4A ADAPT ya Kuchungua Wanaobaleghe. Majibu yako yatatusaidia kuamua ni nini kichocheo vijana wanapendelea zaidi. Kuna maswali 21 kwa jumla. Maswali 12 ya kwanza ya kuchungua yatauliza kuhusu sifa za AYA aliyekaguliwa. Maswali 9 yanaofuata ya kuchungua yatauliza AYA kuchagua kati ya kliniki mbili zilizo na mikakati tofauti ya uhamasishaji.

Ifuatayo

TareheDCEIlitolewa

### Tarehe DCE ilitolewa kwa AYA


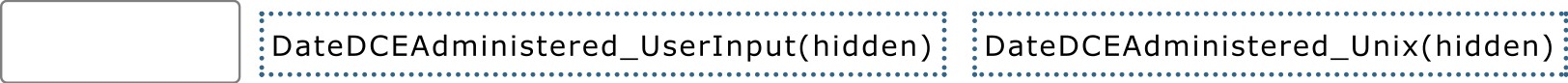

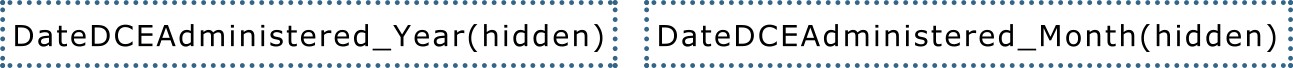

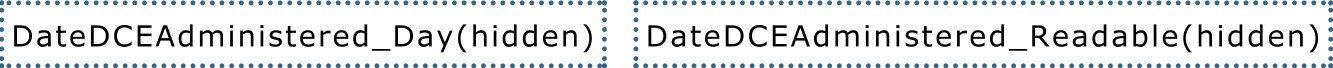


Nyuma

Ifuatayo

0% 1 00%

RAmsimamizi

#### Saini za msimamizi wa RA


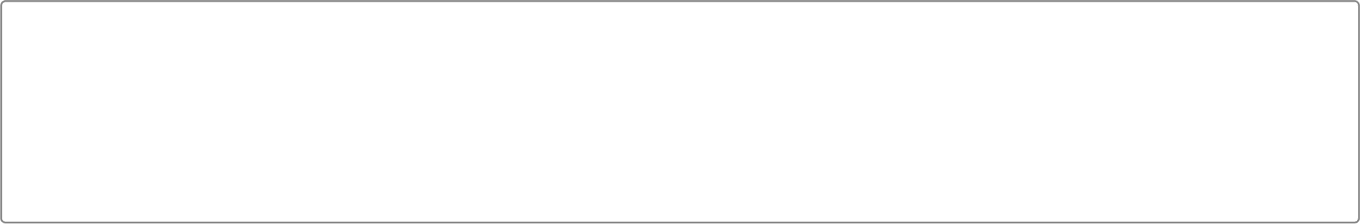


Ifuatayo

0% 100%

Idhini

Je! AYA aliidhinisha kushiriki katika kuchungua?

Idhini =1 11

NDIYO

Idhini = 2

HAPANA

Nyuma

Ifuatayo

0% 1 00%

JinaAYA

### Tafadhali jaza jina la AYA hapa chini

#### Jina la Kwanza

Jina la Kati

#### Jina la Kati

Jina la Mwisho

Jina la Mwisho

Ifuatayo

0% 100%

**Kitambulisho cha Kliniki**


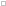


Kitambulisho cha Kliniki cha AYA

-

Nyuma

Ifuatayo Ifuatayo

0% 100%

TYK

Je! Ni ipi tarehe ya kuzaliwa ya AYA? (Ikiwa haijulikani tafadhali acha swali wazi


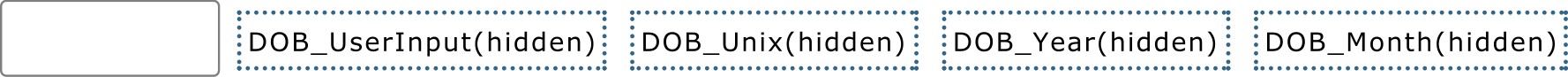


"" "" "" "" "" "" "" "" "" "" "" "" "" "" "" "" "" "" "" "" "" "" "" "" "" "" "" "" "" "" "" "" "" "" "" "" "" "" "" "" "" "" "" "" "" "" "" "" "" "" "" "" "" "" "" "" "" "" "" "" "" "" "" "" "" "".

**TYK_Siku (siri)**

**TYK_Inasomeka (siri)**

Nyuma

Ifuatayo

0% 100%
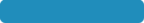


Umri

AYA ana umri wa miaka mingapi leo?

Miaka = 1

Miaka 14 -17

Miaka 18 - 21

Miaka = 2

Miaka = 3

Miaka 21 -24

Ifuatayo

0% 1 00%

Jinsia

#### Jinsia ya AYA

Jinsia = 1

Kike

Jinsia = 2

Kiume

Jinsia = 3

Nyingine/isiyofanana

Nyuma

Ifuatayo

0% 100%
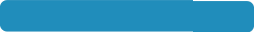


Hali ya Masomo

Je! AYA yuko shuleni sasa hivi?

Hali ya Masomo = 1

Ndiyo

Hali ya Masomo = 2

Hapana

Ifuatayo

0% 1 00%

Kiwango cha shule

mmmmmmmmmmmmmmmmamasomo

Je! AYA amejisajili katika kiwango kipi cha shule kwa sasa?

Kiwango cha shule = 1

Shule ya Msingi

Kiwango cha shule = 2

Shule ya Upili

Kiwango cha shule = 3

Chuo Kikuu

Nyuma

Ifuatayo

0% 1 00%

MjiniKijijini

AYA na familia yao / mlezi wanaishi wapi?

.

MjiniKijijini = 1

Katika mji wa Kisumu

MjiniKijijini = 2

Nje ya mji wa Kisumu

MjiniKijijini_3_nyingine

MjiniKijijini = 3

Nyingine (tafadhali taja)
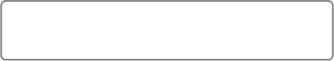


Nyuma

Ifuatayo

0% 100%

Uchumi wa kijamii

Katika miezi 12 iliyopita ni mara ngapi nyumba ya AYA haikuwa na chakula cha kutosha?

Hali ya uchumi wa kijamii=1

Kamwe

Hali ya uchumi wa kijamii=2

Wakati mwingine

Hali ya uchumi wa kijamii=3

Kila mara

Nyuma

Ifuatayo

0^0^  100%

SESQ2

Je! Ni idadi gani ya vyumba vinatumika kwa malazi katika makazi ya AYA?

SESQ2= 1

Moja

SESQ2=2

Mbili

SESQ2=3

Tatu au zaidi

Ifuatayo

0% 1 00%

Mpito

Sasa utawasilisha chaguzi za majaribio ya kuchagua kwa AYA kwa kuwauliza wachague kati ya kliniki mbili zilizo na mikakati tofauti ya uhamasishaji.

Ifuatayo

0 0/ o 100%
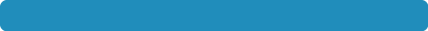


‘[

A4ADCE_Nasibu 1

Ikiwa unaweza kuchagua kuhudhuria moja ya kliniki mbili ambazo hutoa zawadi ya pesa katika ziara za kliniki, ni ipi kati ya kliniki hizi mbili ungechagua?

### Kliniki A Kliniki B




**Unapopokea zawadi**

**Nani anastahiki zawadi hiyo**

Katika kila ziara ya kliniki

Ni vijana tu ambao wanahudhuria ziara za kliniki kwa wakati na wanakandamiza virusi

Mwishoni mwa kila mwaka (kilichohifadhiwa katika kila ziara ya kliniki)

Ni vijana tu ambao wanahudhuria ziara za kliniki kwa wakati na wanakandamiza virusi

**Nani huchukua** Wewe pekee **zawadi**

##### Wewe au mtu uliyemchagua (km mwenzi au mlezi)

**Thamani ya zawadi**

**Zawadi zinagawanywa vipi**

(1 ya 9)

Shilingi 100

Malipo ya simu ya mPesa

:

A4ADCE_Nasibu1

Chagua

,



0%

Ifuatayo

Shilingi 500

Pesa taslimu

;

A4ADCE_Nasibu1___

Chagua



100%

A4ADCE__Nasibu2 2_

Ikiwa unaweza kuchagua kuhudhuria moja ya kliniki mbili ambazo hutoa zawadi ya pesa katika ziara za kliniki, ni ipi kati ya kliniki hizi mbili ungechagua?



### Kliniki A Kliniki B



**Unapopokea zawadi**

**Nani anastahiki zawadi hiyo**

Katika kila ziara ya kliniki

Vijana wote wanaohudhuria kliniki ya ART

Mwishoni mwa kila mwaka (kilichohifadhiwa katika kila ziara ya kliniki)

Vijana wote wanaohudhuria

kliniki ya ART

**Nani huchukua** Wewe pekee **zawadi**

##### Wewe au mtu uliyemchagua (km mwenzi au mlezi)

**Thamani ya** **zawadi**

**Zawadi zinagawanywa vipi**

(2 of 9)

Shilingi 500

Mjazo

:

A4ADCE_Nasibu2

Chagua

,



0%
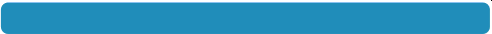


Shilingi 300

Vocha ya ununuzi yaTuskys

(isiyomaliza muda wake)

;

A4ADCE_Nasibu2

Chagua



100%

Ifuatayo

A4ADCE_Nasibu3

Ikiwa unaweza kuchagua kuhudhuria moja ya kliniki mbili ambazo hutoa zawadi ya pesa katika ziara za kliniki, ni ipi kati ya kliniki hizi mbili ungechagua?



### Kliniki A Kliniki B



**Unapopokea zawadi**

**Nani anastahiki zawadi hiyo**

Mwishoni mwa kila mwaka (kilichohifadhiwa katika kila ziara ya kliniki)

Ni vijana tu ambao wanahudhuria ziara za kliniki kwa wakati na wanakandamiza virusi

Mwishoni mwa kila mwaka (kilichohifadhiwa katika kila ziara ya kliniki)

Vijana wote wanaohudhuria

kliniki ya ART

**Nani huchukua** Wewe pekee **zawadi**

Wewe pekee

**Thamani ya** **zawadi**

**Zawadi zinagawanywa vipi?**

(3 kati ya 9)

Shilingi 100

Mjazo

A4ADCE_Nasibu3

Chagua

Shilingi 300



Pesa taslimu

A4ADCE_Nasibu3

Chagua



Ifuatayo

0% 100%

A4ADCE_Nasibu4

Ikiwa unaweza kuchagua kuhudhuria moja ya kliniki mbili ambazo hutoa zawadi ya pesa katika ziara za kliniki, ni ipi kati ya kliniki hizi mbili ungechagua?



### Kliniki A Kliniki B



**Unapopokea zawadi**

**Nani anastahiki zawadi hiyo**

Katika kila ziara ya kliniki

Vijana wote wanaohudhuria kliniki ya ART

Katika kila ziara ya kliniki

Ni vijana tu ambao wanahudhuria ziara za kliniki kwa wakati na wanakandamiza virusi

**Nani huchukua** Wewe au mtu uliyemchagua **zawadi** (km mwenzi au mlezi)

##### Wewe au mtu uliyemchagua (km mwenzi au mlezi)

**Thamani ya** **zawadi**

**Zawadi zinagawanywa** **vipi**

(4 of 9)

Shilingi 500

Mjazo

A4ADCE_Nasibu4

Chagua

:

0%

Ifuatayo

Shilingi 100

Pesa taslimu

A4ADCE_Nasibu4

Chagua

;

100%

A4ADCE_Nasibu5

Ikiwa unaweza kuchagua kuhudhuria moja ya kliniki mbili ambazo hutoa zawadi ya pesa katika ziara za kliniki, ni ipi kati ya kliniki hizi mbili ungechagua?

### Kliniki A Kliniki B

**Unapopokea zawadi**

**Nani anastahiki zawadi hiyo**

Katika kila ziara ya kliniki

Vijana wote wanaohudhuria kliniki ya ART

Katika kila ziara ya kliniki

Ni vijana tu ambao wanahudhuria ziara za kliniki kwa wakati na wanakandamiza virusi

**Nani huchukua** Wewe au mtu uliyemchagua **zawadi** (km mwenzi au mlezi)

Wewe pekee

**Thamani ya** **zawadi**

**Zawadi** **zinagawanywa** **vipi**

(5 of 9)

Shilingi 500

Malipo ya simu ya mPesa

A4ADCE_Nasibu5

Chagua

,

0%

Ifuatayo

Shilingi 300

Malipo ya simu ya mPesa

A4ADCE_Nasibu5

Chagua

100%

A4ADCE_Nasibu6

Ikiwa unaweza kuchagua kuhudhuria moja ya kliniki mbili ambazo hutoa zawadi ya pesa katika ziara za kliniki, ni ipi kati ya kliniki hizi mbili ungechagua?

### Kliniki A Klinik B

**Unapopokea zawadi**

**Nani anastahiki zawadi hiyo**

Mwishoni mwa kila mwaka (kilichohifadhiwa katika kila ziara ya kliniki)

Vijana wote wanaohudhuria kliniki ya ART

Mwishoni mwa kila mwaka (kilichohifadhiwa katika kila ziara ya kliniki)

Ni vijana tu ambao wanahudhuria ziara za kliniki kwa wakati na wanakandamiza virusi

**Nani huchukua** Wewe pekee **zawadi**

##### Wewe au mtu uliyemchagua (km mwenzi au mlezi)

**Thamani ya** **zawadi**

**Zawadi zinagawanywa** **vipi**

(6 of 9)

Shilingi 100

Mjazo

A4ADCE_Nasibu6

Chagua

:

0%

Ifuatayo

Shilingi 500

Vocha ya ununuzi yaTuskys

(isiyomaliza muda wake)

A4ADCE_Nasibu6

Chagua

;

100%

A4ADCE_Nasibu7

Ikiwa unaweza kuchagua kuhudhuria moja ya kliniki mbili ambazo hutoa zawadi ya pesa katika ziara za kliniki, ni ipi kati ya kliniki hizi mbili ungechagua?

### Kliniki A Kliniki B

**Unapopokea zawadi**

**Nani anastahiki zawadi hiyo**

Mwishoni mwa kila mwaka (kilichohifadhiwa katika kila ziara ya kliniki)

Ni vijana tu ambao wanahudhuria ziara za kliniki kwa wakati na wanakandamiza virusi

Katika kila ziara ya kliniki

Vijana wote wanaohudhuria

kliniki ya ART

**Nani huchukua** Wewe au mtu uliyemchagua **zawadi** (km mwenzi au mlezi)

Wewe pekee

**Thamani ya zawadi**

**Zawadi**

**Zinagawanywa vipi**

(7 of 9)

Shilingi 300

Mjazo

A4ADCE_Nasibu7

Chagua

:

,

0%

Shilingi 300

Vocha ya ununuzi ya Tuskys

(isiyomaliza muda wake)

A4ADCE_Nasibu7

Chagua

;

100%

Ifuatayo

A4ADCE_Nasibu8

Ikiwa unaweza kuchagua kuhudhuria moja ya kliniki mbili ambazo hutoa zawadi ya pesa katika ziara za kliniki, ni ipi kati ya kliniki hizi mbili ungechagua?

### Kliniki A Kliniki B

**Unapopokea zawadi**

**Nani anastahiki zawadi hiyo**

Katika kila ziara ya kliniki

Vijana wote wanaohudhuria kliniki ya ART

Katika kila ziara ya kliniki

Ni vijana tu ambao wanahudhuria ziara za kliniki kwa wakati na wanakandamiza virusi

**Nani huchukua** Wewe au mtu uliyemchagua **zawadi** (km mwenzi au mlezi)

Wewe pekee

**Thamani ya**

**zawadi**

**Zawadi** **zinagawanywa**

**vipi**

A4ADCE_Nasibu8

(8 of 9)

Shilingi 100

Pesa taslimu

:

Chagua

0%

Shilingi 500

Vocha ya ununuzi ya Tuskys (isiyomaliza muda wake)

;

A4ADCE_Nasibu8

Chagua

100%

Ifuatayo

A4ADCE_Nasibu9

Ikiwa unaweza kuchagua kuhudhuria moja ya kliniki mbili ambazo hutoa zawadi ya pesa katika ziara za kliniki, ni ipi kati ya kliniki hizi mbili ungechagua?

###

### Kliniki A Kliniki B

**Unapopokea zawadi**

**Nani anastahiki zawadi hiyo**

Mwishoni mwa kila mwaka (kilichohifadhiwa katika kila ziara ya kliniki)

Vijana wote wanaohudhuria kliniki ya ART

Mwishoni mwa kila mwaka (kilichohifadhiwa katika kila ziara ya kliniki)

Ni vijana tu ambao wanahudhuria ziara za kliniki kwa wakati na wanakandamiza virusi

**Nani huchukua** Wewe pekee **zawadi**

##### Wewe au mtu uliyemchagua (km mwenzi au mtunza)

**Thamani ya zawadi**

**Zawadi**

**Zinagawanywa vipi**

(9 ya 9)

Shilingi 500

Pesa taslimu

A4ADCE_Nasibu9

Chagua

: •

,

0%

Shilingi 100

Vocha ya ununuzi ya Tuskys (isiyomaliza muda wake)

A4ADCE_Nasibu9

Chagua

;

100%

Ifuatayo

**TAMATI**

Asante kwa kuchukua uchunguo huu.

0% 1 00%

**Kutohitimu**

Asante kwa kuchukua uchunguo huu. Kwa bahati mbaya ikiwa AYA hatoi idhini yakushiriki hawaruhusiwi kukamilisha huu uchunguo.

Ifuatayo

0% 100%

DCE DATA COLLECTION FORM-DHOLUO

Start

Introduction

Machiegni e A4A ADAPT epenjo mar nonro mar Rowere. Duokoni biro konyowa fwenyo mich kata kony ma ojende ohero ahinya. Gin penjo 21 koriwo tee.

Penjo mag nonro 12 mokuongo biro penjo kit Rawera gi Jomadongo matindo (AYA) ma openj penjo mag nonro.

Penjo 9 mag nonro moluwo biro penjo Rawera gi Jomadongo matindo (AYA) mondo oyier ekind klinige ariyo man kod yore mag kony mopogore.

Next

Tarik ma DCE ne otimne Rawera/Ng’a Maduong matin (AYA)

DateDCEAdministered_UserInput(hidden) DateDCEAdministered_Unix(hidden) DateDCEAdministered_Year(hidden) DateDCEAdministered_Month(hidden)

DateDCEAdministered_Day(hidden) DateDCEAdministered_Readable(hidden)

Back

Next

0% 100%

Nukta mag Jatich Nonro matayo penjo

Back

Next

0% 100%

Bende ne Rawera/Ng’a Maduong matin (AYA) oyie mondo oduoko penjo mag nonro?

Ee

Consent=1

Ooyo

Consent=2

Back

Next

0% 100%

Yie ipong nying’ Rawera/Ng’a Maduong matin (AYA) piny ka

Nying Mokuongo

MiddleName

Nying Madiere

LastName

Nying Mogik

Back

Next

0% 100%

Namba mar Klinik mar AYA

-

Back

Next

0% 100%

Tarik mar nyuol mar Rawera/Ng’a maduong matin (AYA)? (Ka ok ong’e yie iwe penjo nono)

DOB_UserInput(hidden) DOB_Unix(hidden)

DOB_Day(hidden) DOB_Readable(hidden)

DOB_Year(hidden)

DOB_Month(hidden)

Back

Next

0% 100%

Rawera/Ng’a maduong matin (AYA) ni gi higni adi kawuono?

higni 14-17

Age=1

higni 18-21

Age=2

higni 21-24

Age=3

Back

Next

0% 100%

Kit Chwech mar Rawera/Ng’a maduong matin (AYA)

Dhako

Gender=1

Dichuo

Gender=2

Mamoko/gath

Gender=3

Back

Next

0% 100%

Bende Rawera/Ng’a maduong matin (AYA) nie skul sani?

Ee

SchoolingStatus=1

Ooyo

SchoolingStatus=2

Back

Next

0% 100%

En okang’ mane mar skul ma Rawera/Ng’a maduong Matin (AYA) nitie gisani?

Skund Piraimari

SchoolLevel=1

Skund Sekondar

SchoolLevel=2

Mbalariany

SchoolLevel=3

Back

Next

0% 100%

En kanye ma Rawera/Ng’a maduong Matin (AYA) gi joode/jorit odake?

Ei boma ma Kisumo

UrbanRural=1

Oko mar boma ma Kisumo

UrbanRural=2

UrbanRural=3

UrbanRural_3_other

Mamoko (yie iler)

Back

Next

0% 100%

Edweche 12 mokalo en mang’eny marom nade mane od Rawera/Ng’a maduong matin (AYA) ne onge gi chiemo moromo?

Podi

SocioeconomicStatus=1

Seche moko

SocioeconomicStatus=2

Kinde duto

SocioeconomicStatus=3

Back

Next

0% 100%

Kwan ute adi mitiyogo gi Rawera/Ng’a maduong Matin (AYA) mar nindo ekar dakne?

Achiel

SESQ2=1

Ariyo

SESQ2=2

Adek kata Mang’eny

SESQ2=3

Back

Next

0% 100%

Koro ibiro chiwo yiero mag temo ne Rawera/Ng’a maduong matin (AYA) ka ipenjogi mondo gi yier ekind klinik ariyo man kod yor kony/mich mopogore.

Back

Next

0% 100%

Ka inyalo yiero dhie eklinik achiel ekind klinige ariyo machiwo mich mag pesa ekinde mag limbe eklinik, mane kuom klinigegi mi ibiro yiero?

Select

A4ADCE_Random1

Yor chudo mar sim ma mPesa

Siling ma Kenya 100

In kendi

Mana ne ojende mabiro elimbe mag klinik ewang’ saa & kute othir kende

Elimbe kalimbe mar klinik

**Klinik A**

**Kinde ma iyudo emich**

Select

A4ADCE_Random1

Pesa

In kata ng’ama iyiero (k.k. jaodi kata jarit)

Siling ma Kenya 500

Mana ne ojende mabiro elimbe mag klinik ewang’ saa & kute othir kende

Egiko mar higa kahiga (okan elimbe kalimbe mag klinik)

**Klinik B**

**En ng’a mowinjore mi mich**

**Ng’a makawo mich**

**En mich marom nade**

**Mich ipogo nade**

(1 of 9)

Back

Next

0% 100%

Ka inyalo yiero dhie eklinik achiel ekind klinige ariyo machiwo mich mag pesa ekinde mag limbe eklinik, mane kuom klinigegi mi ibiro yiero?

Select

A4ADCE_Random2

Pesa wuoyo esim (Airtime)

Siling ma Kenya 500

In kendi

Ne ojende duto mabire eklinik mar Yedhe Magayo kute mag Ayaki (ART)

Elimbe kalimbe mar klinik

**Klinik A**

Select

A4ADCE_Random2

Risit mar nyiepo Tuskys (ma kindene ok rum)

In kata ng’ama iyiero (k.k. jaodi kata jarit)

Siling ma Kenya 300

Ne ojende duto mabire eklinik mar Yedhe Magayo kute mag Ayaki (ART)

Egiko mar higa kahiga (okan elimbe kalimbe mag klinik)

**Klinik B**

**Kinde ma iyudo emich**

**En ng’a mowinjore mi mich**

**Ng’a makawo mich**

**En mich marom nade**

**Mich ipogo nade**

(2 of 9)

Back

Next

0% 100%

Ka inyalo yiero dhie eklinik achiel ekind klinige ariyo machiwo mich mag pesa ekinde mag limbe eklinik, mane kuom klinigegi mi ibiro yiero?

Select

A4ADCE_Random3

Pesa wuoyo esim (Airtime)

Siling ma Kenya 100

In kendi

Mana ne ojende mobire elimbe mag klinik ewang’ saa & kute othir kende

Egiko mar higa kahiga (okan elimbe kalimbe mag klinik)

**Klinik A**

Select

A4ADCE_Random3

Pesa

Siling ma Kenya 300

In kendi

Ne ojende duto mabire eklinik mar Yedhe Magayo kute mag Ayaki (ART)

Egiko mar higa kahiga (okan elimbe kalimbe mag klinik)

**Klinik B**

**Kinde ma iyudo emich**

**En ng’a mowinjore mi mich**

**Ng’a makawo mich**

**En mich marom nade**

**Mich ipogo nade**

(3 of 9)

Back

Next

0% 100%

Ka inyalo yiero dhie eklinik achiel ekind klinige ariyo machiwo mich mag pesa ekinde mag limbe eklinik, mane kuom klinigegi mi ibiro yiero?

Select

A4ADCE_Random4

Pesa wuoyo esim (Airtime)

In kata ng’ama iyiero (k.k. jaodi kata jarit)

Siling ma Kenya 500

Ne ojende duto mabire eklinik mar Yedhe Magayo kute mag Ayaki (ART)

Elimbe kalimbe mar klinik

**Klinik A**

Select

A4ADCE_Random4

Pesa

In kata ng’ama iyiero (k.k. jaodi kata jarit)

Siling ma Kenya 100

Mana ne ojende mobiro elimbe mag klinik ewang’ saa & kute othir kende

Elimbe kalimbe mar klinik

**Klinik B**

**Kinde ma iyudo emich**

**En ng’a mowinjore mi mich**

**Ng’a makawo mich**

**En mich marom nade**

**Mich ipogo nade**

(4 of 9)

Back

Next

0% 100%

Ka inyalo yiero dhie eklinik achiel ekind klinige ariyo machiwo mich mag pesa ekinde mag limbe eklinik, mane kuom klinigegi mi ibiro yiero?

Select

A4ADCE_Random5

Yor chudo mar sim ma mPesa

In kata ng’ama iyiero (k.k. jaodi kata jarit)

Siling ma Kenya 500

Ne ojende duto mabire eklinik mar Yedhe Magayo kute mag Ayaki (ART)

Elimbe kalimbe mar klinik

**Klinik A**

Select

A4ADCE_Random5

Yor chudo mar sim ma mPesa

Siling ma Kenya 300

In kendi

Mana ne ojende mobiro elimbe mag klinik ewang’ saa & kute othir kende

Elimbe kalimbe mar klinik

**Klinik B**

**Kinde ma iyudo emich**

**En ng’a mowinjore mi mich**

**Ng’a makawo mich**

**En mich marom nade**

**Mich ipogo nade**

(5 of 9)

Back

Next

0% 100%

Ka inyalo yiero dhie eklinik achiel ekind klinige ariyo machiwo mich mag pesa ekinde mag limbe eklinik, mane kuom klinigegi mi ibiro yiero?

Select

A4ADCE_Random6

Pesa wuoyo esim (Airtime)

Siling ma Kenya 100

In kendi

Ne ojende duto mabire eklinik mar Yedhe Magayo kute mag Ayaki (ART)

Egiko mar higa kahiga (okan elimbe kalimbe mag klinik)

**Klinik A**

**Kinde ma iyudo emich**

Select

A4ADCE_Random6

Risit mar nyiepo Tuskys (ma kindene ok rum)

In kata ng’ama iyiero (k.k. jaodi kata jarit)

Siling ma Kenya 500

Mana ne ojende mabiro elimbe mag klinik ewang’ saa & kute othir kende

Egiko mar higa kahiga (okan elimbe kalimbe mag klinik)

**Klinik B**

**En ng’a mowinjore mi mich**

**Ng’a makawo mich**

**En mich marom nade**

**Mich ipogo nade**

(6 of 9)

Back

Next

0% 100%

Ka inyalo yiero dhie eklinik achiel ekind klinige ariyo machiwo mich mag pesa ekinde mag limbe eklinik, mane kuom klinigegi mi ibiro yiero?

Select

A4ADCE_Random7

Pesa wuoyo esim (Airtime)

In kata ng’ama iyiero (k.k. jaodi kata jarit)

Siling ma Kenya 300

Mana ne ojana mobiro elimbe mag klinik ewang’ saa & kute othir kende

Egiko mar higa kahiga (okan elimbe kalimbe mag klinik)

**Klinik A**

Select

A4ADCE_Random7

Risit mar nyiepo Tuskys (ma kindene ok rum)

Siling ma Kenya 300

In kendi

Mana ne ojende mabiro elimbe mag klinik ewang’ saa & kute othir kende

Elimbe kalimbe mar klinik

**Klinik B**

**Kinde ma iyudo emich**

**En ng’a mowinjore mi mich**

**Ng’a makawo mich**

**En mich marom nade**

**Mich ipogo nade**

(7 of 9)

Back

Next

0% 100%

Ka inyalo yiero dhie eklinik achiel ekind klinige ariyo machiwo mich mag pesa ekinde mag limbe eklinik, mane kuom klinigegi mi ibiro yiero?

Select

A4ADCE_Random8

Pesa

In kata ng’ama iyiero (k.k. jaodi kata jarit)

Siling ma Kenya 100

Ojende duto mabiro eklinik mar Yedhe mag gayo Kute mag Ayaki (ART)

Elimbe kalimbe mar klinik

**Klinik A**

Select

A4ADCE_Random8

Risit mar nyiepo Tuskys (ma kindene ok rum)

Siling ma Kenya 500

In kendi

Mana ne ojende mabiro elimbe mag klinik ewang’ saa & kute othir kende

Elimbe kalimbe mar klinik

**Klinik B**

**Kinde ma iyudo emich**

**En ng’a mowinjore mi mich**

**Ng’a makawo mich**

**En mich marom nade**

**Mich ipogo nade**

(8 of 9)

Back

Next

0% 100%

Ka inyalo yiero dhie eklinik achiel ekind klinige ariyo machiwo mich mag pesa ekinde mag limbe eklinik, mane kuom klinigegi mi ibiro yiero?

Select

A4ADCE_Random9

Pesa

Siling ma Kenya 500

In kendi

Ne ojende duto mabire eklinik mar Yedhe Magayo kute mag Ayaki (ART)

Egiko mar higa kahiga (okan elimbe kalimbe mag klinik)

**Klinik A**

Select

A4ADCE_Random9

Risit mar nyiepo Tusky's (ma kinde mare ok rum)

In kata ng’ama iyiero (k.k. jaodi kata jarit)

Siling Ma Kenya 100

Mana ne ojende mobiro elimbe mag klinik ewang’ saa & kute othir kende

Egiko mar higa kahiga (okan elimbe kalimbe mag klinik)

**Klinik B**

**Kinde ma iyudo emich**

**En ng’a mowinjore mi mich**

**Ng’a makawo mich**

**En mich marom nade**

**Mich ipogo nade**

(9 of 9)

Back

Next

0% 100%

Erokamano kuom duoko penjo nonroni.

0% 100%

Erokamano kuom duoko penjo nonroni. Kuom hawi marach ka AYA ok oyie mar bedo enonro to ok onyal tieko duoko penjo mag nonro.

Back

Next

0% 100%

NOT LISTED IN APPENDIX

**Adverse Childhood Experiences International Questionnaire (ACE-IQ)**

|  | HABARI YA DEMOGRAFIA | |
| --- | --- | --- |
| 1 | NDOA | |
| 1.2  [M2] | IkiwaUmewai kuolewa?ulikuwa umri gani mara ya kwanza kuolewa? | Umri [ ][ ] |
|  |  | sijaiwai olewa [Enda M5] |
|  |  | Kukataa Kujibu [Enda M5] |
| 1.3  [M3] | Je Wakati wa ndo yako ya kwanza,ulimchagua mchumba wako? | Ndio *(Enda M5)* |
|  |  | La |
|  |  | Sijui / Sina uhakika |
|  |  | Kataa kujibu |
| 1.4  [M4] | Ikiwa haukujichagulia mchumba wako wa ndoa yako ya kwanza,ulipeana ruhusa yako kwa uamuzi huo? | Ndio |
|  |  | La |
|  |  | Kataa kujibu |
| 1.5  [M5] | Ikiwa wewe ni mzazi ulikuwa wa umri gani ulipopata mtoto wako wa kwanza? | Umri [ ][ ] |
|  |  | Haitumiki |
|  |  | ataa kujibu |

| 2 | UHUSIANO NA WAZAZI/WATUNZI | | |
| --- | --- | --- | --- |
|  | *ULIZA YOTE*  Wakati wa utoto wako( miaka 18 ya kwanza ya maisha yako) | | |
| 2.1  [P1] | Je!Wazazi/walezi wako waliwai elewa shida/wasiwasi zako | Kila mara | |
|  |  | Mara nyingi | |
|  |  | Saa zingine | |
|  |  | Nadra | |
|  |  | Hata kamwe | |
|  |  | Kata kujibu | |
| 2.2  [P2] | Wazazi/walezi wako walijua/najua jinsi unavyotumia mda wako wa ziada/kupumzika ukiwa nje ya shule/kazini? | Kila mara | |
|  |  | Mara nyingi | |
|  |  | Saa zingine | |
|  |  | Nadra | |
|  |  | Hata kamwe | |
|  |  | Kataa kujibu | |
| 3 |  | | |
| 3.1  [P3] | Ni mara ngapi wazazi /walezi wako hawakukupa chakula kinachotosha ingawa walikuwa na uwezo wa kufanya hivo. | Mara mingi | |
|  |  | Mara chache | |
|  |  | Mara moja | |
|  |  | Nadra | |
|  |  | Kataa kujibu | |
| 3.2  [P4] | Wazazi/walezi wako walikuwa/ni walevi au kutumia mihadarati kiasi kwamba hawawezi.hawangeweza kukuhudumia? | Mara mingi | |
|  |  | Mara chache | |
|  |  | Mara moja | |
|  |  | Nadra | |
|  |  | Kata kujibu | |
| 3.3  [P5] | Ni mara ngapi wazazi/walezi wako hakufadhili kuenda shulekwako hata ingawa shule ilikuwepo? | Mara mingi | |
|  |  | Mara chache | |
|  |  | Mara moja | |
|  |  | Nadra | |
|  |  | Kata kujibu | |
| 4 | MAZINGIRA YA KIFAMILIA | | |
|  | *ULIZA YOTE*  Katika utoto wako(miaka 18 ya kwanza ya uhai wako) | | |
| 4.1  [F1] | Uliishi/unaishi na mmjoa wa familia ambaye alikuwa/yuko na shida ya ulevi au utumizi mbaya wa mihadarati au dawa za maagizo? | | Ndio |
|  |  |  | La |
|  |  |  | Kataa kujibu |
| 4.2  [F2] | Ulikuwa au unaishi na mmoja wa familia yako ambaye alikuwa/yuko na unyogovu, ugonjwa wa ki akili au maudhui ya kujiodoa uhai? | | Ndio |
|  |  |  | La |
|  |  |  | Kataa kujibu |
| 4.3  [F3] | Umewai/unaishi na mmoja wa familia yako ambaye amewai tumikia kifungo cha jela? | | Ndio |
|  |  |  | La |
|  |  |  | Kataa kujibu |
| 4.4  [F4] | Wazazi wako wamewai tengana/achana? wameachana? | | Ndio |
|  |  |  | La |
|  |  |  | Haitakikani hapa |
|  |  |  | Kataa kujibu |
| 4.5  [F5] | Mama,baba au mlezi aliaga dunia? | | Ndio |
|  |  |  | La |
|  |  |  | Sijui/sina uhakika |
|  |  |  | Kataa kujibu |
| *ULIZA YOTE*    Maswali yafuatayo ni kuhusu vitu ambavyo umeviskia au kuona nyumbani mwenu. Vitu hivi huenda vilifanyika kwa mmoja katika familia sio lazima iwe wewe. | | | |

| Wakati wa utoto wako(miaka 18 ya kwanza ya uhai wako) | | | |
| --- | --- | --- | --- |
| 4.6  [F6] | uliwai/unashudia au kusikia mzazi au mmoja katika familia pale nyumbani akikemewa,kupigiwa kelele kuapiwa,kutukanwa and kuaibishwa? | | Mara nyingi |
|  |  |  | Mara chache |
|  |  |  | Mara moja |
|  |  |  | Nadra |
|  |  |  | Kataa kujibu |
| 4.7  [F7] | Uliwai/unashuhudia au kusisikia mazazi au mmoja wa familia pale nyumbani akipigwa makofi,kupigwa teke,kupigwa ngumi na kupigwa? | | Mara nyingi |
|  |  |  | Mara chache |
|  |  |  | Mara moja |
|  |  |  | Nadra |
|  |  |  | Kataa kujibu |
| 4.8  [F8] | Uliwai/unashuhudia au kusisikia mazazi au mmoja wa familia pale nyumbani akigongwa au kukatwa na kifaa kama vile kijiti/kiboko,chupa,bakora,kisu au mjeledi? | | Mara mingi |
|  |  |  | Mara chache |
|  |  |  | Mara moja |
|  |  |  | Nadra |
|  |  |  | kataa kujibu |
| *ULIZA WENYE MIAKA 18 NA ZAIDI*  Yafuatayo ni maswali kuhusu vitu ambavo huenda umevishuhudia.  Wakati wako wa utoto(miaka 18 ya kwanza ya uhai wako) | | | |
| 5 |  | | |
| 5.1  [A1] | Mzazi /mlezi wako au mmojawa familia aliwai/unakupigia kelele au kukuhapia kukukemewa, kukuaibisha? | Mara mingi | |
|  |  | Mara chache | |
|  |  | Mara moja | |
|  |  | Nadra | |
|  |  | Kataa kujibu | |
| 5.2  [A2] | Mzazi /mlezi au mmoja wa familia amewai kukutishia kukuacha au kukufurusha nyumbani? | Mara mingi | |
|  |  | Mara chache | |
|  |  | Mara moja | |
|  |  | Nadra | |
|  |  | Kataa kujibu | |
| 5.3  [A3] | Mzazi /mlezi au mmoja wa familia amewai kukuzaba makofi kukupiga teke,ngumi au kukupiga tu? | Mara mingi | |
|  |  | Mara kidogo | |
|  |  | Mara moja | |
|  |  | Nadra | |
|  |  | Kataa kujibu | |
| 5.4  [A4] | Mzazi /mlezi wako au mmoja wa familia pale nyumbani amewai kukugonga au kukatwa na kifaa kama vile kijiti/kiboko,chupa,bakora,kisu au nyaunyo? | Mara mingi | |
|  |  | Mara chache | |
|  |  | Mara moja | |
|  |  | Nadra | |
|  |  | Kataa kujibu | |

| 6 | VURUGU ZA RIKA | | | |
| --- | --- | --- | --- | --- |
| *ULIZA YOTE*  Maswali yafuatayo ni kuhusu kuzuwa/kuteswa wakati wa utoto wako.uonevu ni wakati vijana husema au kutendea mwenzao vitu amabavyo havifuraishi.pi ni uonevu wakati kijana anapotapeliwa au kuwachwa nje kwa jambo kimakusudi. Sio uonevu wakati vijana wawili wenye nguvu sawa wanazozana au kupimana nguvuau kutaniana kwa njia ya upendo  Wakati wako wa ototo( miaka18 yako ya kwanza ya uhai wako) | | | | |
| 6.1  [V1] | Ni mara ngapi uliwadhulumiwa/unateswa na wenzako? | | | Mara mingi |
|  |  |  |  | Mara chache |
|  |  |  |  | Mara moja |
|  |  |  |  | Sijawai (*enda.swali 3)* |
|  |  |  |  | Kataa kujibu |
| 6.2  [V2] | Ulikuwa unaonewa vipi mara nyingi? | | | Nilipigwa nikashungushwa ,nikapigwa teke na kufungiwa kwa nyumba |
|  |  |  |  | Nilichekwa kwa sababu ya rangi yangu au uraia wangu |
|  |  |  |  | nilichekwa kwa sababu ya dini yangu |
|  |  |  |  | nilichekwa na utani wa ngono na ishara |
|  |  |  |  | niliwachwa nje ya shughuli kwa kusudu na kupuuzwa kabisa |
|  |  |  |  | nilichekwa kwa sababu ya maumbile yangu ya mwili na uso |
|  |  |  |  | Nilidhulumiwa kwa njia ziinginezo |
|  |  |  |  | kata kujibu. |
| *Uliza yoote*  Vita hufanyika wakati vijana wawili wenye nguvu sawa wanaamua kupigana wenyewe. | | | | |
| Wakati wako wa utoto( miaka 18 ya kwanza ya uhai wako) . . .  Maaswali yafuatayo ni kuhusu vita | | | | |
| 6.3  [V3] | Umekuwa kw avita mara ngapi? | | | mara mingi |
|  |  |  |  | mara chache |
|  |  |  |  | mara moja |
|  |  |  |  | Nadra |
|  |  |  |  | Kata kujibu |
| 7 | | MKUSUDIO WA VITA NA UKATILI | | |
|  | | *ULIZA YOTE*  Msawali yafuatayo ni kuhusyu ikiwa ulishuhudia au haukushuhudia wakati wa utoto wako.Tokeo hizi ni kuhusu jumla ya vita ugaidi gasia za kisiasa au kijamii mauaji unyanyasaji,kupotea kuteswa au vita vilivyopangwa kama uwizi na ugaidi.  Wakati wa ototo wako(tmiaka yako (miak18 ya kwanza ya uhai wako.) | | |
| 7.1  [V4] | | Je umelazimishwa kuenda kuishi kuingine kwa sababu ya hafla zozte hizi? | Mara mingi | |
|  |  |  | Mara chache | |
|  |  |  | Mara moja | |
|  |  |  | Nadra | |
|  |  |  | Kataa kujibu | |
| 7.2  [V5] | | Uliwai/una shuhudia uharibifu wa nyumba yenu kwa kusudia kutokana na matokeo haya? | Mara migi | |
|  |  |  | Mara kidogo | |
|  |  |  | Mara moja | |
|  |  |  | Nadra | |
|  |  |  | Kataa kujibu | |
| 7.3  [V6] | | Umewai kupigwa na polisi,wanajeshi, kundi la militia au kundi haramu? | Mara nyingi | |
|  |  |  | Mara kidogo | |
|  |  |  | Mara moja | |
|  |  |  | Nnadra | |
|  |  |  | Kataa kujibu | |
| 7.4  [V7] | | Kulikuwa/kuna mmoja wa familia /rafiki yako aliuwawa au kupigwa na wanajeshi,polisi,kundi la militia au kundi haramu? | Mara nyingi | |
|  |  |  | Mara kidogo | |
|  |  |  | Mara moja | |
|  |  |  | Nadra | |
|  |  |  | kataa kujibu | |

**Comprehensive ART Adherence Measurement**

*Wakati huu nitakuuliza maswali kuhusu uzingatiaji.*

| 1.Je!Umejiandikisha katika mpango wa lishe □Ndio □La | |
| --- | --- |
| Maswali ya kuzingatia | |
| 2. Unapata shida kuchunga wakati wa dawa  □Ndio □ La  Wakati upi? Jioni □ Jumamosi na jumapili  Siku za wiki □:___Zinginezo_________________ | Je! Unapata shida kuchunga wakati wa dawa  □Ndio □ La  Ni shida gani ukonazo(eleza) |
| 4. Saa zingine ni vigumu kumeza dawa. Unaweza kuwa hujameza dawa kwa sababu zifuatazo:  nilisaha kumeza dawa  □Nilikuwa shuleni/kazini/mabali na nyumbani □ Nimekataa kumeza dawa  □ Sikutaka watu waone □ Dawa huwa chungu kwangu  □ Dawa hunifanya nijihisi mgonjwa □ Zinginezo eleza  □ Nilijiskia mgonjwa/nilikuwa natapika  □ Siwezi meza bila chakula  □. Hakuna mojayapo . | |
| 5. Shida katika kliniki wakati mwingine hufanya kuchukua dawa kuwa ngumu kila siku. Yafuatayo yamekuwa shida kwako?  □ Dawa kukosa kwenye kituo cha dawa    Dawa zipi □ ARVs □Septrin □ (jumuisha)  □Nilimaliza /niliishiwa na dawa  □ Wakati wa kungoja dawa ulikuwa mrefu nikaondoka  □) Zingine(eleza)_____________________  □ Hakuna mojawapo hapo juu | |
| Sasa tutakuliiza maswali kuhusu kukosa vipimo vya dawa.Tukisema kipimo tunamaanisha dawa ambazo unafaa kumeza asubuhi na jioni. Ikiwa ulimeza dawa asubuhi na ukasahau hata tembe moja ya jioni,inamaanisha ulimeza vipimo vyako vya subuhi na ukakosa vya jioni. Ikiwa ulikosa tembe zako za asubuhi na za jioni pia basi umekosa vipimo vyako viwili. Ikiwa una mswali yeyote kuhusu vipimo vya dawa tafadhali uliza tunavoendelea. | |
| Kwa wiki iliyopitaa  Ni siku ngapi ulikosa kipimo ki moja . □0 □1 □2 □3 □4 □5 □6 □7 □ sijui  bNi siku ngapi ulimeza dawa ukiwa umechelewa kwa saa moja □0 □1 □2 □3 □4 □5 □6 □7  □ sijui  .  c.Ni vipimo ngapi Zaidi au sirinji Zaidi za dawa ulitumia _________________________ □ sijui | |
| Ni vipimo ngapi za dawa ulikosa kwa mwezi uliopita? ___________ □ sijui | |

**Tathmini ya jumla ya afya ya watoto (GHAC)**

Maswali ya ubora wa maisha kwa vijana

3. Tangu ziara yako ya mwisho, yafuatayo yamekutendeka?

Katika usajili: yeyote kati ya haya yafuatayo yamefanyika kwa miezi 12 iliyopita?

x. Wewe,Mzazi, au mpenzi kupoteza kazi	◻ Ndio	◻ La
y. Wewe,Mzazi, au mpenzi kuanza kazi	◻ Ndio	◻ No
z. Mmoja wa familia kuondoka nyumbani	◻ Ndio	◻ No
aa. Kupoteza nyumba au ilibidi kuhama	◻ Ndio	◻ No
bb. Kupotesa usaidizi,kama vile malezi ya	◻ Ndio	◻ No

Mfanyikazi wa kijamii.

cc. Kuondoka kliniki ya vijana kwenda kliniki wa watu wazima	◻ Ndio	◻ No
dd. Kupoteza bima	◻ Ndio	◻ No
ee. Mmoja wa familia kulazwa	◻ Ndio	◻ No
ff. Mmoja wa familia kuwa mgonjwa	◻ Ndio ◻ No
gg. Kubadilisha mlezi	◻ Ndio	◻ No
hh. Kuachana na mpenzi au wazazi	◻ Ndio	◻ No
ii. Talaka kutoka kwa mpenzi au ya wazazi	◻ Ndio	◻ No
jj. Jkufungwa jela kwako,mlezi au mpenzi	◻ Ndio	◻ No
kk. Kuolewa au mlezi kuoa/kuolewa	◻ Ndio	◻ No
ll. Kuzaliwa kwa mdogo wako	◻ Ndio	◻ No
mm. Kuzaliwa kwa mtoto	◻ Ndio◻ No
nn. Mama kuanza kufanya kazi	◻ Ndio	◻ No
oo. Kuanza shule/college/chuo kikuu	◻ Ndio	◻ No
pp. Anza shule mpya/chuo cha mafunzo/chuo kikuu	◻ Ndio	◻ No
qq. maliza/acha shule /chuo cha mafunzo/chuo kikuu	◻ Ndio	◻ No
rr. Mzazi Kubadili hali ya kifedha	◻ Ndio	◻ No
ss. Kumpoteza rafiki wako wa karibu(wa kijana)	◻ Ndio	◻ No
tt. Kifo cha mmoja wa familia	◻ Ndio	◻ No

Ikiwa kifo kwa familia,nani aliaga?	◻ Mama

◻ Baba

◻ Ndugu/dada

(ndugu/dada wa kando)

◻ Babu/nyanya

◻ Mchumba/mpenzi

◻ mwingine wa familia

4. Nikiwa na shida au jambolinalonisumbua,ninao marafiki na wana familia ambao wanaweza kunisadia. Unaweza sema maneno haya:

◻ Ni ukweli	◻ Labda ni ukweli	◻ Labda si kweli	◻ Ni uongo

| *I. Viwango vya jumala vya Afya* | | |
| --- | --- | --- |
| Taarifa hizi zinauliza kuhusu afya na tabia zako. kwa kiwango 1-10 (1 kuwa mbaya zaidi na 10 kuwa nzuri zaidi.) je umejiskia aje kwa wastani kwa miezi mitatu iliyopita? | | |
| Kuhisi | Vibaya zaidi | Vizuri zaidi |
| Kwa ujumla | 1 2 3 4 5 6 7 8 9 10 | |
| Kimwili | 1 2 3 4 5 6 7 8 9 10 | |
| Kihisia | 1 2 3 4 5 6 7 8 9 10 | |
| Kuhusu shuguli zao za kawaida kama shule ,kazi,au kazi za nyumbani | 1 2 3 4 5 6 7 8 9 10 | |

*II.Hali ya maambukizi*

Maswali haya yanauliza kuhusu ufahamu wako wa afya.Jaza nafasi zilizo wazi mahali inapofaa.

1. Ulikuwa umri gani ulipoambiwa kuwa una virusi vya ukimwi?

____miaka, ____miezi	□ siwezi kumbuka / sijui

2. Nani alijadili utambuzi huu na wewe?

□ mlezi wa msingi	□ wanafamilia wengine

□ Timu ya huduma wa afya: nani___________□ wengine: Nani:___________

3. Nani alishiriki katika majadiliano ya utambuzi?

□ Mlezi wa msingi	□ Wanafamilia wengine: nani:___________

□ Timu ya huduma ya afya: nani___________	□ Wengine: nani:___________

4. Uliskia aje kuhusu majadiliano haya ya utambuzi huu?

□ Nilihisi majadiliano yalikuwa yakusadia

□ Nilihisi nina msaada wa kutosha kutoka kwa timu ya utunzaji wa afya.

□ Nilielewa kilichoelezwa

□ Sikuhisi kuwa nilielewa kilichoelezwa

□ Sikuhisi timu ya faya ilinisadia vya kutosha.

□ Nilihisi kuwa majadiliano yalikuwa magumu saana

□ Mengine...

5. Wakati uliambiwa utambuzi wako wa kwanza Je!

| g. Kusikitisha? | □ Ndio □ la |
| --- | --- |
| h. Maudhui/furaha? | □ Ndio □ La |
| i. Kuogopa? | □ Ndio □ La |
| j. Kutolewa mzigo? | □ Ndio □ La |
| k. Hasira? | □ Ndio □ La |
| l. Hakukuwa na hisia? | □ Ndio □ La |

7. Unahisije sasa kuhusu maisha na hali yako ya afya?

| g. kusikitisha? | □ Ndio □ La |
| --- | --- |
| h. Maudhui/furaha? | □ Ndio □ La |
| i. Kuogopa? | □ Ndio □ La |
| j. Kutolewa mzigo? | □ Ndio □ La |
| k. Hasira? | □ Ndio □ La |
| l. Hakukuwa na hisia? | □ Ndio □ La |

8. Je kumekuwa nini kizuri juu yakutambua hali yako ya kiafya?

| g. Ninashirikiana Zaidi kuhusu huduma ya matibabu na kuchukua dawa | □ Ndio □ La |
| --- | --- |
| h. Niko wazi kabisa kwa familia na marafiki | □ Ndio □ La |
| i. Naelewa ni kwanini mimi ni mgonjwa | □ Ndio □ La |
| j. Naelewa jukumu la kuzuia kuambukiza wengine | □ Ndio □ La |
| k. Kuambiwa ukweli kumeboresha uhusiano wangu na familia. | □ Ndio □ La |
| l. Mengine… | □ Ndio □ La  (Eleza)_____________________________________ |

9. Je kuna shida zozote zifuatazo kwa sababu umejua hali yako?

| i. Niliwaambia marafiki na majirani na wengine ambao hawapaswi kujua | □ Ndio □ La |
| --- | --- |
| j. Niliwaambia wanafamilia ambao hawafai kujua | □ Ndio □ La |
| k. Nina huzuni | □ Ndio □ La |
| l. Nogopa kifo | □ Ndio □ La |
| m. Tabia yangu ni mbaya zaidi | □ Ndio □ La |
| n. Sishirikiani saana nahuduma ya matibabu na kuchukua dawa | □ Ndio □ La |
| o. Nimeachwa nje au kubaguliwa | □ Ndio □ La |
| p. mengine? | □ Ndio □ La (eleza)_____________ |

| III. *vizuizi na mapendeleo* |  |
| --- | --- |
| Kufikiria kuhusu mahudhurio yako ya kliniki na ukandamizaji wa virusi changamoto zakokubwa zaidi ni gani? Tafadhali weka kwa nafazi 5 bora Zaidi ukianza na changamoto kubwa zaidi kutoka kwa orodha | 2. 2. 3. 4. 5.   Changamoto kubwa zaidi. Changamoto ya 0 ya 5 |
| Ukifikiria watu wengi wenye rika yako wanahudhuria kliniki na ukandamizaji wa virusi changamoto zao kubwa ni gani? Tafadhali weka kwa nafazi 5 bora Zaidi ukianza na changamoto kubwa Zaidi kutoka kw a orodha | 2. 2. 3. 4. 5.   Changamoto kubwa zaidi. Changamoto ya 0 ya 5 |
| 9. **Kupata utunzaji**    a. Shida na uchukuzi    b. Nauli ya kufika kliniki    c. Sina wakati wa kutosha wa kliniki    d. Ufikiaji mwingine wa kupata utunzi, (eleza) ______________) 10. **Kazi,shule ,Familia**     a. Kuingiliwa kazini     b. Kuingilia ratiba ya shule kwa kuende ujazaji wa dawa     c. Kuingilia ratiba ya shule kwa kuendea matibabu     d. Kijana/mlezi alihudhuria mazishi     e. Kijana/mlezi alikuwa na majukumuya kifamilia     f. Mwenzi hakutaka nihudhurie kiliniki     g. Ndio, kuzaa, mlezi kubadilika (eleza ______________)     h. Sababu zingine za kazi/kifamilia, eleza   **3.Matibabu**   11. Felt too sick to come to clinic     a. Mlezi alihisi mgonjwa saana kuhudhuria kliniki     b. Madhar kutokana na vidonge/tembe     c. Mzigo wa kumeza tembe     d. Nilitaka kupumzikia tembe     e. Mimba /kujifungua     f. Sababu zingine za kimatibabau eleza:     g. ___ kulazwa hospitalini🡪 Tarehe ya kulazwa: ______/________/________     h. siku /mwezi/mwaka     i. Jina la hospitali     j. Sababu ya kulazwa 12. **Ubora wa Kliniki**     a. Kliniki iliishiwa na dawa     b. Mpito kwenda kwa kliniki ya watu wazima     c. Wafanyikazi hawakuwa wazurui     d. Huduma haikuwa nzuri     e. Mahitaji mengi saana ya usimamaizi     f. Sehemu ya kusubiri haikuwa nzuri     g. Kliniki hiyo ilikuwa imejaa mno     h. Lazima nisubiri saana wakati wa ziara     i. Sikutaka kwenda kwenye darasa la uhifadhi 13. **kisaikologia na jamii**     a. Nlijiskia vizuri sikuona haja ya kwenda hospitalini     b. Kuhudhuria kliniki kulihatarisha kufichua hali yaVirusi kwa mtu wa jamii     c. Niliogopa kliniki itanikemea kwa kukosa ziaara     d. Kwa sababu nilienda kwa mtu ambaye anajaribu kuniponya / jaribu kuniponya kwa maombi / mila ya kidini     e. Kwa sababu niliona/naona mganga wa kienyeji     f. Nilihisi huzuni na bila matumaini     g. Unywaji wa pombe     h. Nilisahau tarehe ya ziara     i. Mmoja wa familia au mtu wamaana alisema mtoto anafaa kuacha kuenda hospitalini 14. *Sijui* 15. *Kata kuenda kliniki* | |
|  |  |
| Ikiwa unajitahidi kukaa katika utunzi wa virusi vya ukimwi( kwa mfano kutembea kwenye kliniki,kumeza dawa kila siku,kukandamiza virusi ni msaada gani unaweza kukusaidia kabisa( weka 3 bora na 1 iwe chaguo bora zaidi) | Msaada wa rika langu  Msaada wa rika kutoka kwa mtu wa ndani  Msaada mdogo wa kifedha  Yikumbusho vya maandishi  Mengine (eleza) ___________________  Hakuna msaada wa ziada |
| Ikiwa mtu wa umri wako anajitahidi kukaa katika utunzi wa virusi vya ukimwi ni msaada aina gani unaweza kuwa bora zaidi kwao (weka 3 bora na 1 iwe chaguo bora zaidi) | Msaada wa rika langu  Msaada wa rika kutoka kwa mtu wa ndani  Msaada mdogo wa kifedha  Kumbusho vya maandishi  Mengine (eleza) ___________________  No Hakuna msaada wa ziada |

*Ustawi wa kisaikologia*

Taarifa hizi zinahuzisha shida za tabia ambazo vijana na vinaja wakubwa wanazo. Unavyosoma kila sentensi, amua ni kifungu kipi kinaeleza tabia yako kwa miezi 3 iliyopita

Nikifikria kuhusu tabia yangu kwa miezi 3 iliyopita……

29. Nina mabadiliko ya ghafla katika mhemko/hisia zangu

□ mara nyingi ni kweli	□ wakati mwinge ni kweli	□ Si kweli

30. Nahisi kuwa hauka mtu anayenipenda

□ mara nyingi ni kweli	□ wakati mwinge ni kweli	□ Si kweli

31. Mimi ni mkali na nina wasiwasi

□ mara nyingi ni kweli	□ wakati mwinge ni kweli	□ Si kweli

32. Ninadanganya au kusema uwongo

□ mara nyingi ni kweli	□ wakati mwinge ni kweli	□ Si kweli

33. Ninaogopa saana au kuwa na wasiwasi

□ mara nyingi ni kweli	□ wakati mwinge ni kweli	□ Si kweli

34. Ninabishana saana

□ mara nyingi ni kweli	□ wakati mwinge ni kweli	□ Si kweli

35. Nina shida kuzingatioa/siwezi zingatia kwa mda mrefu

□ mara nyingi ni kweli	□ wakati mwinge ni kweli	□ Si kweli

36. Ninachanganyikiwa kiurahisi nahisi kuwa na ukungu

□ mara nyingi ni kweli	□ wakati mwinge ni kweli	□ Si kweli

37. Mimi huwa mnyanyasaji mkatili au mwovu kwa wengine

□ mara nyingi ni kweli	□ wakati mwinge ni kweli	□ Si kweli

38. Mimi sio mtiifu nyumbani

□ mara nyingi ni kweli	wakati mwinge ni kweli	□ Si kweli

39. Mimi sio mtiifu shuleni

□ mara nyingi ni kweli	□ wakati mwinge ni kweli	□ Si kweli

40. Sihisi huruma baada ya kutenda kosa

□ mara nyingi ni kweli	□ wakati mwinge ni kweli	□ Si kweli

41. Nina shida kuelewana na wengine

□ mara nyingi ni kweli	□ wakati mwinge ni kweli	□ Si kweli

42. Nina shida kuelewana na walimu

□ mara nyingi ni kweli	□ wakati mwinge ni kweli	□ Si kweli

43. Nina msukumo wa kufanya jambo bila kufikiria

□ mara nyingi ni kweli	□ wakati mwinge ni kweli	□ Si kweli

44. Najiona sina thamani/nahisi duni

□ mara nyingi ni kweli	□ wakati mwinge ni kweli	□ Si kweli

45. siko kama wengine

□ mara nyingi ni kweli	□ wakati mwinge ni kweli	□ Si kweli

46. Nina ugumu kutoa mawazo yangu kwenye jambo fulani

□ mara nyingi ni kweli	□ wakati mwinge ni kweli	□ Si kweli

47. Sina utulivu/najishughulisha kupita kiasi siwezi kaa kimya

□ mara nyingi ni kweli	□ wakati mwinge ni kweli	□ Si kweli

48. Ni mkaidi na hasira/kukasirika

□ mara nyingi ni kweli □ wakati mwinge ni kweli	□ Si kweli

49. Nina hasira kali saana na hupoteza kwa urahisi

□ mara nyingi ni kweli □ wakati mwinge ni kweli	□ Si kweli

50. Sina furaha,huzuni

□ mara nyingi ni kweli □ wakati mwinge ni kweli	□ Si kweli

51. Nimejitenga,sishirikiani na wengine

□ mara nyingi ni kweli □ wakati mwinge ni kweli	□ Si kweli

52. Nahisi wengine wananionea

□ mara nyingi ni kweli □ wakati mwinge ni kweli	□ Si kweli

53. Ninajuhusisha na wototo wanoleta shida

□ mara nyingi ni kweli □ wakati mwinge ni kweli	□ Si kweli

54. Ni msiri na kuweka mambo ya kwangu kivyangu

□ mara nyingi ni kweli □ wakati mwinge ni kweli	□ Si kweli

55. Nina wasiwasi saana

□ mara nyingi ni kweli □ wakati mwinge ni kweli	□ Si kweli

56. Nina tegemea wengine saana

□ mara nyingi ni kweli □ wakati mwinge ni kweli	□ Si kweli

Hojaji ya maswali yaAfya ya Mgonjwa( PHQ-9)

| Maswali- | Majibu | | | |
| --- | --- | --- | --- | --- |
| Kwa wiki mbili zilizopita ni mara ngapi umesumbuliwa na yeyote kati ya shida zifuatazo( tumia“✔kuonyesha jibu lako) | Hapana kabisa | Kwa siku kadhaa | Zaidi ya nusu ya siku | karibu kila siku |
| 1.Mvuto kidogo au furaha kufanya vitu | 0 | 1 | 2 | 3 |
| 2.Kuhisi kuchoka,huzuni kukosa matumaini | 0 | 1 | 2 | 3 |
|  | kama zote ni 0 enda mwisho la sivyo elekea 3 | | | |
| 3.Shida kulala au kupata usingizi au kulala usingizi mzito sana | 0 | 1 | 2 | 3 |
| 4. Kuhisi uchovu au kuwa mnyonge | 0 | 1 | 2 | 3 |
| 5.Kukosa hamu ya chakula au kula chakula kidogo | 0 | 1 | 2 | 3 |
| 6. Kuhisi vibaya kujihusu auumeshindwa kujikimmu au kukimu familia yako | 0 | 1 | 2 | 3 |
| 7. Shida kuwa makini kwa jambo kama kusoma au kusoma gazeti au kutizama runinga | 0 | 1 | 2 | 3 |
| 8. Kutembea au kuongea polepole ikawa watu wamejua? Au kinyume kuwa na wasiwasi na kuzunguka mara mingi isiyo kawaida | 0 | 1 | 2 | 3 |
| 9. Fikira kuwa itakuwa vyema ufe au kujiumiza kwa njia fulani kuliko kuwa hai | 0 | 1 | 2 | 3 |

Jumla ya Alama

Alama 10-19= mwelekeze kwa kliniki kwa ushauri

Alama 20 nazidi=mwelekeze kwa mtaalamu wa kisaria(psychaiatrist)

| Demografia Usimamizi--------------------------------  Kitambulisho cha Utafiti wa ADAPT ………. Tarehe ya mahojiano----------------  Majina ya anayefanya mahojiano haya……. Alama ya anyefanya mahojiano------ |  |  |  |  |  |  |  |  |  |  |  |  |  |  |  |  |  |
| --- | --- | --- | --- | --- | --- | --- | --- | --- | --- | --- | --- | --- | --- | --- | --- | --- | --- |
|  | Kwa siku 30 zilizopita,uliugua kutokana na madhara yeyote au kuumia kama vile kukohoa,homa, kuhara,kuumia kutokana na ajali au ugonjwa wowote?  1 Ndio ugonjwa  2 Ndio ajali  3 Ndio ugonjwa na ajali  4 La **àEndakwa sehemu ya Kulazwa** | Anza na tukio lilokuwa kali zaidi ni ugonjwa upi au jeraha gani uliopta?  *Rekodi mpaka mara 3 ugonjwa /dalili kwa kipindi (angalia orodha ya nambari pamoja na zingine zilizotajwa)* | Ulienda wapi kutafuta matibabu  Ikiwa haukutafuta matibabau ni sababu gani ilikufanya usiende kutafuta  *Rekodi hadi nambari 3 wanotoa hudumakila kipindi; ikiwa hakuna huduma/matibabau iliyotafutwatumia nambari :”hakuna” kurekodi sababu na zinginezo( eleza)* | JUmla wa kati uliotumika kutafuta huduma ya matibabu(pesa taslimu) (kwa silingi za kenya)  Ikiwa utunzaji uliipokewa bila malipo andika 0 | Jumla ya masaa ilyotumika kutafuta na kupata matibabau. | Jumla ya masaa nilipoteza kukosa shule,kazi au kazi za nyumbanibkutokana na ugonjwa/jeraha hili | Kulikuwa na ugonjwa au jeraha uliugua kwa siku 30 zilizopita?  1. Ndio *à Enda mstari ufuatao, Swali 3a, kwa kipindi kijacho kali saana hadi vipindi 3 kwa kila mtu*  2. La àEnda kwa sehemu ya kulazwa. |  |  |  |  |  |  |  |  |  |  |
|  |  |  |  | Safari ya kutafuta matibabu(safari ya kwenda na kurudi) | Ada ya ushauri wa wagonjwa wa nje | madawa | Upimaji wa mahabara | Nauli ya kulazwa hospitalini ikijumuisha chakula | Matumizi mengine ya afya |  |  |  |  |  |  |  |  |
| 1 | 2 | 3a | 3b | 3c | 4a | 4b | 4c | 5 | 6 | 7 | 8 | 9 | 10 | 11 | 12 | 13 |  |
| Sehemu11 |  |  |  |  |  |  |  |  |  |  |  |  |  |  |  |  |  |
| Sehemu 2 |  |  |  |  |  |  |  |  |  |  |  |  |  |  |  |  |  |
| Sehemu 3 |  |  |  |  |  |  |  |  |  |  |  |  |  |  |  |  |  |

| Kwa mwaka mmoja uliopita ni mara ngapi ulilazwa hospitalini( kukaa hospitalini Usiku)  Kama “0” *à Enda kwa utathmini* | 2. Kulazwa hospitalini kwa hivi karibu  - Ilikuwa nini uchunguzi/sababu ya kulazwa hospitalini?*Andika erekodi kuu kwa kila sababu ya kulazwa*  - Ni siku ngapi ulimaliza/ulilazwa hospitalini?  *Rekodi hadi kulazwa 3 ukianza naya hivi karibuni.* | | | | | |
| --- | --- | --- | --- | --- | --- | --- |
|  | Kulazwa1(ya hivi karibuni) | | Kulazwa 2 | | Kulazwa 3. | |
|  | a. utambuzi | b. Siku | c. utambuzi | d. siku | e.Utambuzi | f. siku |
|  |  |  |  |  |  |  |
|  |  |  |  |  |  |  |
|  |  |  |  |  |  |  |
|  |  |  |  |  |  |  |
| Nambari ya dalili na magonjwa   38. Maumivu ya tumbo 39. Ajali/kiwewe 40. kuukmwa na mifupa 41. Ugumu kupumua 42. kuchomeka 43. saratani 44. baridi kuhisi( joto na baridi) 45. Kukohoa 46. Kukohoa damu 47. Shida ya meno 48. Ugonjwa wa kisukari 49. Kuendesha/kuhara saana 50. Kuhara sugu(Zaidi ya mwezi moja) 51. kuzimia 52. Joto jingi 53. Joto kila mara kurudia 54. kuvunjika 55. Maumivu ya mwili kwa ujumla | 56. Vidonda vya sehemu ya siri 57. Ukimwi 58. Maumivu ya kuumwa kichwa vikali 59. Shinikizo la damu 60. Malaria 61. Surua 62. Ugonjwa wa ki akili 63. Maumivu ukipitisha mkojo 64. Homa ya mapavu(Pneumonia) 65. Yanayohusiana na mimba/ujauzito 66. Koo kuuma 67. Upele wa ngozi 68. Kifua kikuu 69. Vidonda vya tumbo 70. kutapika 71. Unyonge 72. Kupoteza kilo saana 73. Kidonda 74. Zinginezo (eleza) | | | Nambari ya wanotoa huduma(provider codes)   11. Hospitali 12. Nyumbani (familia) 13. Nyumbani (mganga wa kienyeji 14. Mganga wa kienyeji (nje ya kaya home) 15. Kliniki ya kibinafsi 16. Kituo cha afya 17. Duka la kuuza dawa 18. Duka la dawa 19. Duka la kawaida 20. nyinginezo(eleza ulikopata matibabau)   21. Hakuna – Gharama kubwa sana kutafuta matibabu  22hakuna –Mbali sana na kituo cha afya/muuguzi.  23Hakuna – sikujiskia mgonjwa saana kiasicha kutafuta matibabau  24.Hakuna – nilikuwa na majukumu mengine (e.g., shule,kazini,kazi za nyumbani, etc.)  Hakuna –sababu nyinginezo(eleza sababu ya kutotafuta matibabu) | | |
|  |  | | | | | |

**A4A. B16: Hojaji ya matumizi ya pombe na madawa ya kulevya**

| Ningependa kukuuliza maswali kuhusu pombe,vileo na madawa ya kulevya ambavo uliwai chukua/tumia bila kupewa na daktari wako. Majibu yako hayataambiwa mtu yeyote,hata wazazi/walezi wako | | | | | |
| --- | --- | --- | --- | --- | --- |
| Sehemu ya kwanza: Uchunguzi wa pombe kwa ujumla | | | | | |
| Je!umewai kunywa kinywaji kilicho na pombe? | Ndio | La | Sijui | Kataa kujibu |  |
| Sehemu ya pili: Hojaji 10 za hojaji | | | | | |
| Tafadhali chagua jibu ambalo ni sahihi kwako | | | | | |
| 1. Ni mara ngapi unakunywa kinywaji kilicho na pombe? | Nadra | kila mwezi au chini | mara mbili kwa mwezi | mara mbili au tatu kwa mwezi | mara nne au Zaidi kila mwezi |
| 2. Unakunywa vinywaji ngapi vya pombe siku ya kawaida unapokunywa? | 1au 2 | 3 au 4 | 5 au 6 | 7 mpaka 9 | 10 na zaidi |
| 3. Ni mara ngapi unakunywa vinywaji sita kwa hafla ya unywaji? | Nadra | chini ya mwezi | kila mwezi | kila wiki | kila siku/karibu kila siku |
| 4. Ni mara ngapi katika mwaka uliopita ulipata kuwa hauwezi wacha kunywa pombe unapoanza? | Nadra | chini ya mwezi | kila mwezi | kila wiki | kila siku/karibu kila siku |
| 5. Ni mara ngapi kwa mwaka mmoja uliopita kile ambacho kawaida kilitarajiwa kutoka kwako haukutimiza kwa sababu ya kunywa pombe? | Nadra | chini ya mwezi | kila mwezi | kila wiki | kila siku/karibu kila siku |
| 6. NI mara ngapi kwa mwaka mmoja uliopita umehitaji kinywaji cha kwanza asubuhi ili uweze kujitambua vyema kufuatia kikao cha kunywa saana? | Nadra | chini ya mwezi | kila mwezi | kila wiki | kila siku/karibu kila siku |
| 7. Ni mara ngapi kwa mwaka mmoja uliopita umekuwa na hisia ya hatia au majuto baada ya kunywa pombe? | Nadra | chini ya mwezi | kila mwezi | kila wiki | kila siku/karibu kila siku |
| 8. NI mara nagapi katika mwaka mmoja uliopita umeshindwa kukumbuka yaliyotokea usiku uliopita kwa sababu ya kunywa pombe? | Nadra | chini ya mwezi | kila mwezi | kila wiki | kila siku/karibu kila siku |
| 9. Je! wewe au mtu mwingine ameumia kutokana na unywaji wa pombe? | La | | Ndio:,lakini sio mwaka huu | | Ndio:kwa mwaka uliopita |
| 10. Je jamaa, rafiki au daktari au mfanyikazi mwingine wa afya amejali kuhusu unywaji wako wa pombe au kupendeka upunguze/uwache? | La | | Ndio: lakini sio mwaka uliopita | | Ndio :mwaka huu |
| Utaratubu wakufunga magodi/jumuisha | | | | | |
| Maswali 1-8 yanafungwa/sahihishwa hivi 0, 1, 2, 3, au 4. Maswali 9 na 10 yanafungwa/jumuishwa 0, 2, au 4 pekee. Majibu ya kujumuisha ni kama yafuatayo. | | | | | |
| Swali la 1 | 0(Nadra) | 1(chini ya mwezi) | 2(kila mwezi) | 3(kila wiki) | 4(kila siku/karibu kila siku) |
| Maswali 2 | 0(1au 2) | 1(3au4) | 2(5au6) | 3(7mpaka 9) | 4(10 na zaidi) |
| Maswali 3-8 | 0(Nadra) | 1.(chini ya mwezi) | 2.(kila mwezi) | 3.(Kila wiki) | 4(kila siku/karibu kila siku) |
| Masawli 9-10 | 0(La) | | 2(Ndio,lakini sio mwaka huu) | | 4(Ndio kwa mwaka uliopita) |
| Alama ya chini(kwa wasiokunywa)ni 0 na alama ya juu/inyowezekana nai 40  Alama ya 8 au zaidi inaonyesha uwezekano mkubwa wa unywaji pimbe hatari. | | | | | |
| Sehemu ya 3: Utumizi wa madawa ya kulevya( imeridhiwa kutoka KIAS) | | | | | |
| 1. Ushawai jaribu madawa kama Miraa, Bhangi, Glue, Mandrax, Cocaine, Heroin na  Mengineyo? | La | | Ndio | Sijui | Kataa kujibu |
| Umewai jaribu yafuatayo? | Jibu | | | Ikiwa umejari uliyatumia mwisho lini? | |
| 2.Khaat / Miraa | □ Ndio  □ La  □ Sijui  □ kata kujibu | | | □ Mwezi huu  □ Miezi 2-6 ilyopita  □ Miezi 6-12 ilyopita  □ Zaidi yam waka mmoja uliopita  □ sijui  □ Kataa kujibu | |
|  |  |  |  |  |  |
| 3. Glue / Petroli /  Gundi | □ Ndio  □ La  □ Sijui  □ kata kujibu | | | □ Mwezi huu  □ Miezi 2-6 ilyopita  □ Miezi 6-12 ilyopita  □ Zaidi yam waka mmoja uliopita  □ sijui  □ Kataa kujibu | |
| 4. Bhangi / Bangi | □ Ndio  □ La  □ Sijui  □ kata kujibu | | | □ Mwezi huu  □ Miezi 2-6 ilyopita  □ Miezi 6-12 ilyopita  □ Zaidi yam waka mmoja uliopita  □ sijui  Kataa kujibu | |
| 5. Mandrax | □ Ndio  □ La  □ Sijui  □ kata kujibu | | | □ Mwezi huu  □ Miezi 2-6 ilyopita  □ Miezi 6-12 ilyopita  □ Zaidi yam waka mmoja uliopita  □ sijui  □ Kataa kujibu | |
| 6. Cocaine | □ Ndio  □ La  □ Sijui  □ kata kujibu | | | □ Mwezi huu  □ Miezi 2-6 ilyopita  □ Miezi 6-12 ilyopita  □ Zaidi yam waka mmoja uliopita  □ sijui  □ Kataa kujibu | |
| 7. Heroin | □ Ndio  □ La  □ Sijui  □ kata kujibu | | | □ Mwezi huu  □ Miezi 2-6 ilyopita  □ Miezi 6-12 ilyopita  □ Zaidi yam waka mmoja uliopita  □ sijui  □ Kataa kujibu | |
| 8. Zingine (Eleza), |  | | |  | |
|  |  |  |  |  |  |

A4A – Hojaji ya Demografia na jamii (Socio-Demographic Questionnaire )

| Kamilisha zote kwenye ukurasa huu kabla ya kuanza mahojiano. | | | |
| --- | --- | --- | --- |
| SD-A-ID | Nambari ya kutambulisha mshiriki | □□□-□□□ | |
| SD-A-TOVUTI | Pahali pakufanyia utafiti | Tovuti 1  Tovuti 2  Tovuti 3  Tovuti4  Tovuti 5  Tovuti 6 | 1  2  3  4  5  6 |
| SD-A-HUSISHO | Hali ya kushirikishwa kwa mshiriki | Uhifadhiwa katika huduma  Kupotea kwa kufwatiliwa | 1  2 |
| SD-AJINSIA | Jinsi ya mshiriki | Kiume  Kike | 1  2 |
| SD-A-LUGHA | Lugha ya mahojiano | kizungu  Kiswahili  Dholuo | 1  2  3 |
| SD-A-TAREHE | Tarehe ya kutathmini | SIKU MWEZI MWAKA | |
| SD-A-RA | Nambari za mfanyikazi wautafiti |  | |

*MAELEKEZO:* *Maagizo ya muhojaji yako kwenye italiki—haya ni ya matumizi yako na haifai kusomwa kwa sauti. Ikiwa ni lazima unaweza soma chaguzi za maswali ili kuhimiza muhojiwa. Soma maswali yote na usizitize kwenye maandishi yasiyo ya italiki.Tafadhali soma habari yoote ilivyoandikwa. Usisome kwa sauti majibu ya”Sijui’ na ‘kataa kujibu”. Zungusha namba karibu na jibu lasijui” kama muhojiwa amejibu Hajui Zungusha namba karibu na jibu la sijui” kama muhojiwa amesema hataki kujibu. Kwa maswali yanayokuhitaji wewe kuandika jibu badala ya kuzungusha kwenye jibu katika fomu au mhojiwa anapojibu kwa mengine na kupeana jibu lake tafadhali andika jibu lake kwa herufi kubwa kwa makini iwezekanavyo.*

*UTANGULIZI:* **Asante saana kwa kukubali kushiriki katika utafiti huu. Baadhi ya maswali nitakuuliza ni ya kibinafsi huenda yakakufanya uhisi aibu/usiwe huru. Unaweza kataa kujibu maswali ambayo haujiskii kujibu Unaweza pia kuniuliza maswali ikiwa hauelewi.Unaweza pia kusimamisha mahojiano wakati wowote ukiamua kuwa hautaki kuendelea kushiriki.Tafadhali kumbuka kuwa majibu yako ya maswali yanatambulika tu kwa nambari na yatawekwa siri. Majina yako hayatakuwa kwenye nakala hii.**

| Nambari | **MASWALI NAMAELEZO** | MAJIBU |  | RUKA |
| --- | --- | --- | --- | --- |
| ***DEMOGRAFIA***  Wacha tuanze na maswali ya kimsingi kukuhusu | | | | |
| 65. | Una miaka mingapi leo  *Ingiza jibu kwa miaka Mhojiwa akijibu “sijui”ulizia kwa miaka mitanoikaribiayoAkikataa kujibu ingiza99.Mshiriki akipeana mwaka kuzaliwa,piaga hesabu ujue umri mshiriki akipeana tarehe pekee ya kuzaliwa ingiza jibu/habari kwa nafasi iliyopeanwa na hesabu umri baada ya mahojiano* | Miaka  DIA MÊS ANO  *SIKU YA KUZALIWA*  SIKU MWEZI MWAKA |  |  |
| 66. | Je! Kwa sasa unaenda shule? | Ndio  La  *sijui*  *Kataakujibu* | 1  2  *88*  *99* | *kama 1, 88,99 🡪 swali la4* |
| 67. | Ni nini sababu ya kutoenda shule? (shule ya msingi,chuo/chuo kikuu) | Nimekuwa mgonjwa  Sipendi shule  Lazima nichunge wana familia  Hakuna fedha za kutosha  Shule iko mabli  Nafanya kazi  Nishamaliza masomo*.*  *Sijui*  *Kataa kujibu* | 1  2  3  4  5  6  *7*  *88*  *99* |  |
| 68. | Je umefikia kiwango gani cha juu kabisa cha masomo?  *Usisome chaguzi. Zungusha jibu moja.* | Hakuna  Shule ya msingi (darasa1 – 8)  Shule ya upili (kidato cha 1-4)  Chuo kikuu,chuo taasisi yamafunzo  Chuo kikuu  *Sijui*  *Kataa kujibu* | 1  2  3  4  5  *88*  *99* |  |
| 69. | Je! Kwa sasa uko kwa uhusiano na mtu yeyote? Huyu anaweza kuwa mwenzi wako au mtu unayemuita mpenzi wako?. | Ndio  La  *Sijui*  *Kataa kujibu* | 1  2  *88*  *99* | kama 2 🡪 swali 6 |
| 70. | Uliza ikiwa ako kwenye uhusiano) wenzi wangapi? |  | 1  2  3  4  5  88  99 |  |
| 71. | Je! Uhusiano wako ni gani na mpenzi wako? *Ikiwa zaidi ya mpenzi mmoja tafuta uhusiano uliopo na mwenzi mkuu)* | Tumeoana ,tunailishi pamoja  Tumeoana hatuishi pamoja  Hatujaoana lakini tunaishi pamoja  Hatuishi pamoja  kutengwa /talaka  mjane  *sijui*  *kataa kujibu* | 1  2  3  4  *5*  *6*  *88*  *99* |  |
| 72. ~~1a~~ | Je! Ni shughuli gani kuu ambayo huchukua siku yako?  *Usisome chaguzi. Zungusha jibu moja.*  *.* | Mwanafunzi  Sina ajira  Mke nyumbani  Mkulima  Muuzaji (k.m,soko,mitaa)  Mfanyikazi wa nyumba (k.m. Msafishaji, Mpishi, yaya)  Mtaalamu(k.m mwalimu,muuguzi))  Msusi/mpambaji  Mlinzi/usalama  Mjenzi  Dereva  Mengine______________________  *sijui*  *kataa kujibu* | 1  2  3  4  5  6  7 8  9  10  11  12  *88*  *99* |  |
| MAHUSIANO YA MTOTO NA MLEZI NA MUUNDO WA KAYA  Sasa ningepanda kukuuliza maswali kadhaa juu ya kaya. | | | | |
| 73. | Je! kuzaliwa kwako ni namba ngapi katika familia? | Mzaliwa wa kwanza  Mzaliwa wa pili  Mzaliwa wa kwana  Mzaliwa wa nne  Mzaliwa wa tao  Mzaliwa wa mwisho  Mengine (eleza ___)  *taja nambari ya kuzaliwa ikiwa sio 1-5 au wa mwisho*  Sijui  Kataa kujibu | 1  2  3  4  5  6  7  88  99 |  |
| 74. | Ni nini kinaeleza hali yako ya sasa ya maisha  *Ruhusu mhojiwa aeleze na RA aingize majibu sahihi. Usisome chaguzii kwa sauti* | Kaya na wanfamilia wa karibu inaweza Jumuiisha wanfamalia kando)  Kaya na wanafamilia wa kando (lakini Sio wanafamilia wa karibu))  Kaya na wasio wa familia k.v Kituo/shule ya bweni  Eleza _______________  Wasio na makaazi/ / kuishi mitaani  *Sijui*  *Kataa kujibu* | 1  2  3  4  5  *88*  *99* |  |
| 75. | Nani anachukuwa jukumu kubwa kwa ustawi na utunzi wako? | Mimi  Mama  Baba  Shangazi/Mjomba  Babu  Ndugu  Mwenzi  Jamaa wengine  Mlezi asiye jamaa  *Sijui*  *Kataa kujibu* | 1  2  3  4  5  6  7  8  *9*  *88*  *99* | *kama 1, 88,99 🡪 swali la 12* |
| 76. | Anaishi katika nyumba moja na wewe? | Ndio  La  *Sijui*  *Kataa kujibu* | 1  2  *88*  *99* |  |
| 77. | Pamoja na wewe ikiwa unazidi miaka 18ni watu wangapi miaka 18 na Zaidi wanaishi katika kaya yako? | Watuwazimaa    *sijui*  *kataa kujibu* | 88  99 |  |
| 78. | Pamoja na wewe ikiwa una miaka chini ya 18ni watoto wangapi chini ya miaka 18 wanaoishi katika kaya yako? | Watoto  *Sijui*  *Kataa kujibu* | *88*  *99* |  |
| 79. | Katika wastani wa mwezi kwa mapatao na mapato megine familia yako hutengeneza?(jumuisha njia zoote za mapato)  *Ikiwa mshiriki anahitaji usaidizisoma chaguzi kwa sauti. Zungusha jibu moja weka kwenye nafasi uliyopewa ikiwa mshiriki atajibu Sijui’ weka 88. Mshiriki akikataa kujibu weka 99’.* | chini ya elfu  Elfu 1-elfu 3  Elfu 4-8  Elfu 8-15000  Zaidi ya elfu 15000  *Siuji*  *Kataa kujibu* | 1  2  3  4  5  *88*  *99* |  |
| 80. | Kwa mwezi ni mara ngapi kaya yako haikuwa na chakula cha kutosha?  *Zungusha jibu moja* | Kamwe  Wakati mwingine  Kila mara  *Sijui*  *Kataa kujibu* | 1  2  3  *88*  *99* |  |
| 81. | Ni nini chanzo kuu cha maji yakunywa kwa kaya yako?  *Soma chaguo kwa sautizungusha jibu moja.* | Kisima cha umma  Kisima cha kibinafsi  Mfereji wa umma  Maji ya bomba moja kwa moja kwenye makaazi  Mengine, _____________________  *Ssijui*  *Kataa kujibu* | 1  2  3  4  5  *88*  *99* |  |
| 82. | Munatumia umeme nyumbani kwenu?  *Zungusha jibu moja* | Ndio  La  *Sijui*  *Kataa kujibu* | 1  2  *88*  *99* |  |
| 83. | Je! una simu yarununu ambayo ni yako binafsi  *Zungusha jibu moja.* | Ndio  La  *Sijui*  *Kataa kujibu* | 1  2  *88*  *99* | *If yes, Q23* |
| 84. | *Kama ni la kwa kuwa na simu binafsi: Je! Unashirikiana simu/unapata simu ya pamoja inayoshirikishwa?*  *Zungusha jibu moja* | Ndio  La  *Sijui*  *Kataa kujibu* | 1  2  *88*  *99* |  |
| *HISTORIA YA MAPENZI NA UHUSIANO*  Unaweza pata seti ya maswali haya kuwa ya kibinafsi na ni yeti.Uaminifu wako unathaminiwana tafadhali kumbuka kuwa habari hii ni ya siri.Tungependa kukuuliza maswali kuhusu uhusiano wako na uzoefu wako wa ngono? | | | | |
| 85. | Unajua ngono ni nini?  *Zungusha jibu moja* | Ndio  La  *Sijui*  *Kataa kujibu* | 1  2  *88*  *99* |  |
| 86. | Umewahi shiriki ngono?  *Zungusha jibu moja* | Ndio  La  *Sijui*  *Kataa kujibu* | 1  2  *88*  *99* | Ikiwa 2 🡪 swali34 |
| 87. | Mara ya kwanza kushiriki ngono ulikuwa na umri gani? | Miaka  DIA MÊS ANO  *Sijui*  *Kataa kujibu* | *88*  *99* |  |
| 88. | Mara ya kuanza kushiriki ngono, ilikuwa kwa hiari yako au ulilazimishwa?  Zu*ngusha jibu moja* | Nilitaka  Nililazimishwa  *Sijui*  *Kataa kujibu* | 1  2  *88*  *99* |  |
| 89. | Kwa jumla ni watu wangapi tofauti umeshiriki nao ngono? Tafadhali toa nadhani yako bora. | Nambari  *Sijui*  *Kataa kujibu* | *88*  *99* |  |
| 90. | Mara ya mwisho ulishiriki ngono ulitumia kinga?  *Zungusha jibu moja* | Ndio  La  Sikumbuki  *Sijui*  *Kataa kujibu* | 1  2  3  *88*  *99* |  |
| 91. | Je! Kwa sasa wewe na mpenzi wako kuna nji yeyote ya kupanga uzazi /kuchelewesha kushika mimba munayotumia?  *Zungusha jibu moja* | Ndio  La  Sina mpenzi/hairuhusiwi hapa  *Sijui*  *Kataa kujibu* | 1  2  3  *88*  *99* | ikiwa 2, 3, 88, 99 🡪 swali 31 |
| 92. | Ikiwa unatumia njia ya kuchelewesha/kuzui kupata ujauzito ni njia gani wewe na mpenzi wako munatumia?  *zungusha yoote yanokubalika* | IUD/Coil  Sindano  Vidonge vya kuzuia mimba  Kondomu  Njia za asili  Uondoaji  Kutoshiriki ngono  Ksafisha kwa uke  Kusafisha kwa uume  Eleza______________  *Sijui*  *kataa kujibu* | 1  2  3  4  5  6  7  8  9  10  *88*  *99* |  |
| 93. | Hivi sasa una ujauzito?  *Zungusha jibu moja* | Ndio  La  *sijui*  *kataa kujibu* | 1  2  88  99 |  |
| 94. | Umewai kuwa na ujauzito?  *Zungusha jibu moja* | Ndio  La  *Sijui*  *Kataa kujibu* | 1  2  *88*  *99* | ikiwa 2 🡪 swali34  *kwa wanawake tu* |
| 95. | Ikiwa umewahi kuwa mjamzitO umejifungua watoto wangapi? | *Sijui*  *Kataa kujibu* | *88*  *99* | *kwa wanawake tu* |
| 96. | Watoto wako wana umri gani? | *Andika sijui 88 au kataa kujibu 99 inavotakikana kwa kila mtoto*  Umri  Umri  Umri  Umri  *Ongeza maelezo ikiwa twakimo haziingiani.* |  |  |
| 97. | Je! Umewai pata chanjo ya kuzuia saratani ya kizazi(chanjo ya HPV)  *Weka alama ya ndio ikiwa ulipata dosi mokja(inweza kuwa mbili au tatu ikitegemea umri ulipopewa)* | *Ndio*  *La*  *Sijui*  *Kataa Kujibu* | 1  2  88  99 | *Kwa wanawake tu* |
| *HALI YA AFYA YA WANAFAMILIA*  *MAAGIZO:*  Maswali machache yafuatayo ni kuhusu afya yako na afya ya wanafamilia kwa nyumba yako. | | | | |
| 32 | Kwa ufahamu wako mbali na wewenani mwingine katika familia amepatikana na virusi vya ukimwi?  *Zungusha majibu yoote yaliyosemwa*  *Uliza:* ‘yeyote?’  *Ikiwa mhojiwa atataja jibu ambalo halijapewa*  *Eleza kando na nyingine* | Hakuna  Baba ya mushiriki  Mama ya mushiriki  Ndugu za mshiriki  Jamaa/wengine wa AYA  Mpenzi/mwenzi wa AYA  Nyingine______________________  *Sijui*  *Kataa kujibu* | 1  2  3  4  5  6  7  88  99 | *Ikiwa= 1 🡪 swali34* |
| 33. | Kwa ufahamu wako mbali na wewenani mwingine katika familia yuko kwenye matibabu ya virusi vya ukimwi?  *Zungusha majibu yoote yaliyosemwa*  *.*  *Uliza:* ‘yeyote?’  ’ *Ikiwa mhojiwa atataja jibu ambalo halijapewa* | Hakuna  Baba ya kijana  Mama ya kijana  Ndugu za kijanai  Jamaa/wengine wa kijana  Mpenzi/mwenzi wa kijana  Nyingine______________________  *sijui*  *kataa kujibu* | 1  2  3  4  5  6  7  88  99 |  |
| *UTUNZI WA AFYA NA TABIA ZA KLINIKI*  Tungependa kukuuliza maswali kadhaa kuhusu kliniki ya huduma ya afya ambayo unahudhuria au uliwai kuhudhuria hapo zamani. | | | | |
| 34 | Ni nani anyekupeleka kliniki kw amatibabu ya virusi vya ukimwi?  *Zungusha majibu yoote yaliyosemwa* | Mimi  Mama  Baba  Shangazi/Mjomba  Babu  Ndugu  Mwenzi  Jamaa wengine  Mlezi asiye jamaa  *Sijui*  *Kataa kujibu* | 1  2  3  4  5  6  7  8  9  10  *88*  *99* |  |
| 35. | Inakuchukua muda ganikusafiri kwenda kwenye kliniki yako ya huduma ya virusi vya ukimwi.Tafadhali jumuisha wakati uliotumia kungojea usafiri navilevile wakati ulitumia kusafiri?  **Ikiwa kijana haendi kliniki kwa sasa uliza kuhusu wakati alipokuwa akienda kliniki*  *Ikingiza lkkiasi cha muda kwa nafasi iliyotolewa.Ikiwa wakati ni chini y asana moja,ingiza 00 halafu nambari ya dakaki. Ikiwa jibu la mushiriki ni ‘sijui’ ingiza 88 Ikiwa jibu lamushiriki ni ‘kukataa kujibu’ ingiza 99* | Masaa : Dakika  *Sijui*  *Kataa kujibu* | *88*  *99* |  |
| 36. | Inakuchukua pesa ngapi kusafiri safari moja?  ** Ikiwa kijana haendi kliniki kwa sasa uliza kuhusu wakati alipokuwa akienda kliniki*  *Ingiza kiasi kwa nafasi iliyipeanwa. Ikiwa jibu la mushiriki ni ‘sijui’ ingiza 8899 Ikiwa jibu lamushiriki ni kukataa kujibu ingiza 9999* | shilingi za kenya  *Sijui*  *Kataa kujibu* | *88*  *99* |  |
| 37. | *Kwa kijana aliyepotea kwa kufwatiliwa*  Kwanini uliacha kukuja kliniki?  *Zungusha majibu yoote yaliyosemwa.*    ***ikiwa kijana amehifadhiwa katika utunzaji usiulize swali hili,nenda kwa swali lilofuata* | **Upatikanaji wa huduma**  Shida za usafiri  Fedha za kufika kliniki  Sikuwa na wakati wa kutosha kuenda kliniki  Sababu nyingine za ufikiaji utunzi, (eleza______________)  **Kazi,Shule,Familia**  Kuingiliwa na kazi  Kuingiliwa na shule kwa kuchukua dawa  Kuingiliwa na shule kwa kutafuta matibabau  Kijana/mlezi kuhudhuria mazishi  Kijana/mlezi alikuwa na shughulizigine za kifamilia  Mpenzi hakutaka nihudhurie kliniki  Ndoa,uzaaji,mlezi kubadilishwa (eleza ______________)  Sababu nyingine za familia/kazi taja  **Matibabu**  Felt too sick to come to clinic  Mlezi alihisi mgonjwa sana kukuja kliniki  Madhara kutokana na vidonge  Shida ya vidonge  Kutaka kupumzikia vidonge  Mimba /kuzaa  Sababu nyingine za kimatibabau, eleza:  ___ kulazwa hospitali🡪 tarehe ya kulazwa: ______/________/________  DD /MM /YYYY  Jina la hospitali  Sababu ya kulazwa    **Ubora wa kliniki**  Kliniki iliishiwa na dawa  Mpito kutoka kliniki ya vijana kwenda ya watu wazima  Wafanyikazi hawakuwa wazuri  Huduma haikuwa nzuri  Mahitaji mengi sana ya wasimamizi  Sehemu ya kusubiri haikuwa nzuri  kliniki ilikuwa imejaa  Lazima nisubiri kwa mda mrefu wakati wa ziara  Sikutaka kwenda kwenye darasa lauhifadhi    **Jamii na sikologia**  Kujiskia mzima,hakuona haja ya kwenda hospitalini  Kuhudhuria kliniki kulihatarisha ufunuo wa virujsi kawa mtu katika jamii  Niliogopa kliniki itanikemea kwa kukosa ziara.  Kwa sababu nilienda kwa mtu ambaye alijaribu/anajaribu kuniponya kupitia kwa maombi/sala za kidini  kwa sababu badala yake naona/niliona mganga wa kienyeji  Niliona sina tumaini/kuhisi huzuni  Unywaji wa pombe  Nilisahau ziara  Jamaa wa familia au mtu mwingine muhimu alisema mtoto anafaa kuacha kuenda hospitalini  *sijui*  *kataa kujibu* | 1  2  3  4  5  6  7  8  9  10  11  12  13  14  15  16  17  18  19  20  21  22  23  24  25  26  27  28  29  30  30  31  32  33  34  35  36  37  88  99 | *inatumika tu kwa wagonjwa waliopotea kwa huduma*  *chaguo nyingi*  *Ikiwa mtoto amehifadhiwa katika utunzaji 🡪*  *sehemu ya unyanyapaa* |

| **UNYANYAPAA**  Seti inayofuata ya maswali nikuhusu mawazo na hisia zako juu ya jinsi watu wanakutendea wakijua una virusi vya ukimwi. Tafadhali kumbuka kuwa hakuna jibu sahihi au sio sahihi na chochote unachosema kitaifadhiwa kwa faragha. Ikiwa yeyote ya maswali haya yanakufanya usijiskie huru niambie tu na tutaruka swali hilo. | | | | | |
| --- | --- | --- | --- | --- | --- |
| Nambari | Maswali na maagizo | majibu | | ruka | Nambari. |
| 98. | Unawasiwasi gani kwamba watu wengine katika jamii kama vile majirani wako watapata kujua hali yako ya Ukimwi bila wewe kuwaambia?  *Soma chaguzi kwa sauti. Zungusha jibu moja.* | wasiwasi saana  waiwasi kidogo tu  hakuna wasiwasi  *sijui*  *kataa kujibu* | | 1  2  3  *88*  *99* |  |
| Nitaenda kusoma mfululizo wa maswali. Tafadhali katika kila moja niambie ikiwa unakubali au haukubaliani nayo | | kubali | sikubali | *sijui* | *kataa kujibu* |
| 99. | Nimepoteza marafiki kutokana na hali yangu ya virusi vya ukimwi. | 1 | 2 | *88* | *99* |
| 100. | Nimeitwa majina,kudhulumiwa,na kutukanwa kutokana na hali yangu ya virusi vya ukimwi | 1 | 2 | *88* | *99* |
| 101. | Nimepatau baguzi nyumbani kwa sababu ya hali yangu ya Virusi vya ukimwi | 1 | 2 | *88* | *99* |
| 102. | Nimepata ubaguzi shuleni kwa sababu ya hali yangu ya Virusi vya ukimwi | 1 | 2 | *88* | *99* |
| 103. | Nimepata ubaguzi katika jamii kwa sababu ya hali yangu ya Virusi vya ukimwi. | 1 | 2 | *88* | *99* |
| 104. | Nimepata ubaguzi kwenye kliniki kwa sababu ya hali yangu ya Virusi vya ukimwi. | 1 | 2 | *88* | *99* |
| 105. | Unyanyapaa wa virusi vya ukimwi umenifadhaisha na kuleta wasiwasi | 1 | 2 | *88* | *99* |
| 106. | Unyanyapaa wa virusi vya ukimwi umenifanya nihisi huzuni | 1 | 2 | *88* | *99* |
| 107. | Nachagua kutocheza na wengine,kutumia wakati na wengine,kwenda mahali kwa sababu ya hali yangu ya virusi vya ukimwi | 1 | 2 | *88* | *99* |
| 108. | Nahisi ni muhimu kuweka hali yangu ya virusi vya ukimwi kuwa siri. | 1 | 2 | *88* | *99* |
| 109. | Mustakabadhi na au matumaini yangu yamebadilika kwa njia mbaya kwa sababu ya hali yangu ya virusi vya ukimwi | 1 | 2 | *88* | *99* |
| 110. | Wakati mwingine mimi huchelewesha kumeza dawa ndio wengine wasione nikimeza | 1 | 2 | *88* | *99* |
| 111. | Watu wengi katika jamii wanafikiria Ukimwi ni ugonjwa wa tabia chafu/mbaya. | 1 | 2 | *88* | *99* |

| **UFAHAMU NA IMANI YA VIRUSI VYA UKIMWI/UKIMWI**  *MASWALI HAYA YANATAZAMIA UFAHAMU NA IMANI YAKO YA VIRUSI VYA UKIMWI/UKIMWI*  Ningependa kujua kuhusu Imani na mawazo yako juu ya virusi vya ukimwi/ukimwi na huduma na matibabu yanayopatikana kwa watu walio na virusi vya ukimwi. Nitasoma safu ya taarifa na ningependa uniambie ikiwa unakubali au haukubaliani na kila mmoja yao.Tafadhali kumbuka kuwa hakuna jibu sahihi/sio sahihi nayoote utakayosema watawekwa siri na haitaathiri utunzi wa virusi vya ukimwi unopokea. Ikiwa baadhi ya maswali haya yanakufanya kutohisi huru,niambie na tutaruka hilo swali | | | | | |
| --- | --- | --- | --- | --- | --- |
| *Soma kila taarifa kwa mshiriki ikifwatiwa na unakubali au haukubaliani?* | | kukubali | kutokubali | sijui | kataa kujibu |
| 112. | Watu wenye virusi vya ukimwi wanaweza ishi maishamarefu yenye afya wakimeza dawa za virusi vya ukimwi. | 1 | 2 | *88* | *99* |
| 113. | Dawa ya virusi vya ukimwi haifanyi kazi | 1 | 2 | *88* | *99* |
| 114. | Watu wanomeza dawa za virusi vya ukimwi ni vigumu kuambukiza virusi kwa wenzi wao wakati wa ngono. | 1 | 2 | *88* | *99* |
| 115. | Baada ya kupimwa virusi vya ukimwi hakuna haja kutafuta huduma ya virusi vya ukimwi mara moja ikiwa unajihisi mzima. | 1 | 2 | *88* | *99* |
| 116. | Dawa za mitishamba sio nzuri kutibu virusi vya ukimwi/ukimwi. | 1 | 2 | *88* | *99* |
| 117. | Dawa za virusi vya ukimwi yaweza saidia wanawake kujifungua watoto wasio na virusi vya ukimwi. | 1 | 2 | *88* | *99* |
| 118. | Ikiwa watu watatii maagizo juu ya kumeza dawa za virusi vya ukimwi watakuwa na afya bora. | 1 | 2 | *88* | *99* |
| 119. | Ukimwi haiui kila mtu kwa sababu ya dawa za virusi vya ukimwi. | 1 | 2 | *88* | *99* |
| 120. | Waganga wa kienyeji wameponya watu walio na virusi vya ukimwi. | 1 | 2 | *88* | *99* |
| 121. | Ni wakati tu mtu aliye na virusi vya ukimwi anapohisi vibaya anapaswa kwenda hospitalini | 1 | 2 | *88* | *99* |
| 122. | Dawa za virusi vya ukimwi is hazifai kumezwa kwa sababu zina athari mbaya. | 1 | 2 | *88* | *99* |
| 123. | Dawa za virusi vya ukimwi itakufanya uwe tasa. | 1 | 2 | *88* | *99* |
| 124. | Watu wenye virusi vya ukimwi wanaweza kuishi maisha marefu na yenye afya ikiwa watatibiwa na dawa za kienyeji | 1 | 2 | 3 | *99* |
| 125. | Bora mtu aliye navirusi vya ukimwi ajihisi mwenye afya,hakuna haja ya kwenda hospitalini | 1 | 2 | 3 | *99* |
| 126. | Virusi vya ukimwi/ukimwi sio kali wakati watu wanakunywa dawa za kienyeji. | 1 | 2 | 3 | *99* |
| 127. | Dawa za virusi vya ukimwi zinapeanwa tu kwa wenye virusi na wanjihisi wagonjwa. | 1 | 2 | 3 | *99* |
| 128. | Dawa za virusi vya ukimwi hazitanisaidia kuishi muda mrefu. | 1 | 2 | 3 | *99* |

*Maoni ya mhojaji*

*Mahojiano yalikuwa -----------utaratibu	_____ sio kawaida --------------	kama sio kawaida mbona?:*

*Je! Mshiriki alikuwana athari mbaya katika mahojiano? ______ Ndio	______ La*

*Kama Ndio eleza*

*Maoni mengine:*

**A4AUtafiti wa kuridhika na uzoefu wa Uingiliaji (Intervention Satisfaction and Experience Survey)**

Ningependa kukuuliza kuhusu huduma kadhaa ambazo umekuwa ukipokea hapa kwenye kliniki kama sehemu ya utafiti. Majibu yako ya uaminifu yatatusaidia kufanya huduma ziwe bora kwakusaidia vijana na watu wazima katika matunzo na matibabu.

**Urambazaji wa ki elektroniki**

| 1. Katika mwezi 12 iliyopita wakati ulikuwa katika utafiti,je!ulipokea msaada wa ujumbe mfupi na urambazaji wa rika(peer navigation)?kwa njia ya simu? | - Ndio –> endelea 1a. - La –> ruka hadi 2. - Sijui - Kataa kujibu | | | | | | |
| --- | --- | --- | --- | --- | --- | --- | --- |
| Ningependa uniambie umeridhika vipi na sehemu Fulani za utafiti ukitumia kiwango kifuatacho:  Kuridhika saana, kuridhika wala kuridhika au kutoridhika, kutoridhika, kutoridhika saana. | | | | | | | |
| a. Umeridhika vipi na: | *Soma kw asauti* | | | | | *Usisome kwa sauti* | |
|  | Kuridhika saana | kuridhika | wala kuridhika au kutoridhika | kutoridhika | kutoridhika saana | sijui | Kataa kujibu |
| b. Urahisi wa kuwasiliana kutumia simu ? | 🌕 | 🌕 | 🌕 | 🌕 | 🌕 | 🌕 | 🌕 |
| c. Njia ya siri ya mwasiliano kupitia ujumbe mfupi wa ki otomatiki? | 🌕 | 🌕 | 🌕 | 🌕 | 🌕 | 🌕 | 🌕 |
| d. Njia ya siri ya mwasiliano ya urambazaji? | 🌕 | 🌕 | 🌕 | 🌕 | 🌕 | 🌕 | 🌕 |
| e. Urafiki ujumbe mfupi wa kiotomatiki? | 🌕 | 🌕 | 🌕 | 🌕 | 🌕 | 🌕 | 🌕 |
| F Urafiki kwa mawasiliano na habaria? | 🌕 | 🌕 | 🌕 | 🌕 | 🌕 | 🌕 | 🌕 |
| g. Uwezo wa kufikia mhabaria? | 🌕 | 🌕 | 🌕 | 🌕 | 🌕 | 🌕 | 🌕 |
| h. Uaminifu wa habaria? | 🌕 | 🌕 | 🌕 | 🌕 | 🌕 | 🌕 | 🌕 |

| i. Usaidizi wa ujumbe mfupi wa kiotomatiki kwa kumeza dawa zako? | 🌕 | 🌕 | 🌕 | 🌕 | 🌕 | 🌕 | 🌕 |
| --- | --- | --- | --- | --- | --- | --- | --- |
| j. Usaidizi wa habaria kwa kumeza dawa zako? | 🌕 | 🌕 | 🌕 | 🌕 | 🌕 | 🌕 | 🌕 |
| k. usaidizi wa ujumbe mfupi wa ki otomatiki kukusaidia kukumbuka kuhudhuria ziara ya kliniki? | 🌕 | 🌕 | 🌕 | 🌕 | 🌕 | 🌕 | 🌕 |
| l. Usaidizi wa baharia kwako kukuja kliniki? | 🌕 | 🌕 | 🌕 | 🌕 | 🌕 | 🌕 | 🌕 |
| m. Uwezo wako wa kupata habari Zaidi au kuzungumza na mtu kwenye kliniki kuhusu afya yako? | 🌕 | 🌕 | 🌕 | 🌕 | 🌕 | 🌕 | 🌕 |
| n. Kwa jumla umeridhakaje na ujumbe mfupi wa kiotomatiki na usaidizi wa urambazaji uliopokea? | 🌕 | 🌕 | 🌕 | 🌕 | 🌕 | 🌕 | 🌕 |
| o. Kwa jumla umeridhikaje na ujumbe mfupi wa ikotomatiki na usaidizi wa urambazaji uliopokea kwa afya na kukuwa kwako? | 🌕 | 🌕 | 🌕 | 🌕 | 🌕 | 🌕 | 🌕 |
|  | Kuvutiwa saana | kuvutiwa | Kuvutiwa kwa kiasi | Kuvutiwa kidogo tu | Sina mvuto kabisa | sijui | Kataa kujibu |
| p. Je ungekuwa na nia ya kuendelea kupokea urambazaji wa ki eletroniki (ujumbe mfupi wa kiotomatiki na urambazaji kupitia simu ya rununu siku za usoni ikiwa itawezekama? | 🌕 | 🌕 | 🌕 | 🌕 | 🌕 | 🌕 | 🌕 |

| q. Je! Kwa ujumla ni programu gani ulitumia kuwasiliana na baharia? | - Ujumbe wa kawaida wa simu - Whats App - Ujumbe wa facebook - snapchat - Zingine(eleza _______________) - sijui - Kataa kujibu | | | | | | |
| --- | --- | --- | --- | --- | --- | --- | --- |
| r. Ni mara ngai unaweza sema wewe na baharia muliwasiliana? | - Kila siku - Mara chache kwa wiki - Kila wiki - Mara chache kwa wiki - Kila mwezi - Chini ya mwezi - sijui - Kataa kujibu | | | | | | |
| s. kwa jumla ni mara ngapi unaweza sema wewe na baharia muliwasiliana? | - Mara20 na zaidi - Mara 10-19 - Mara 5-9 - Mara 1-4 - sijui - Kataa kujibu | | | | | | |
| t. Je! Unaweza kuesema wewe nabaharia ilikuwa ya mara kwa mara saana masafa sahihi au hayakuwa ya kutosha? | - Mara nyingi saana - Masafa sahih ya kutosha - Sio ya mara ya kutosha - Sijui - Kataa kujibu | | | | | | |
| u. Je! Unaeza sema kuwa mawasiliano yako mengi na baharia yalikuwa kupitia ujumbe mfupi,kuongea au yalikuwa kiwango sawa? | - Mazungumzo ya ujumbe - Mazungumzo ya kuongea - Karibu kiasi sawa cha kila moja - sijui - Kataa kujibu | | | | | | |
| v. Uliridhika kiasi gani ya njia ya msingi ya mawasiliano? | - Ridhika saana - kuridhika - Wala kuridhika au kutoridhika - sikuridhika - Sijaridhika saana - sijui - Kata kujibu | | | | | | |
| w. Je unaweza sema majadiliano na baharia yalikuwa ya kuchana saana? | - Chana kabisa - Chana saana - Chana | | | | | | |

| Chana kaisi, sio chana, sio chana kabisa | - sio chana - sio chana kabisa - sijui - kataa kujibu |
| --- | --- |
| x. Ni mara ngapi ulipokea ujumbe mfupi? | - Kila siku - Kila wiki - Kila mwezi - Chini yamwezi   🌕 sijui   - Kataa kujibu |
| y. Je! Unaweza sema ujumbe mfupi ulikuwa wa kawaida saana,masafa sahihi au haukuwa wa kutosha mara kwa mara? | - Ulikuwa wa mara kwa mara - Masafa sahihi - Haukuwa wa mara kwa mara - sijui - Kataa kujibu |
| z. Ni gani ambayo imekuwa msaada zaidi kwa afya yako na ustawi,ujumbe mfupi wa kiotomatiki,mawasiliano na baharia au ujumbe mfupi wa kiotomatiki na mawasilianona baharia? | - Ujumbe mfupi wa kiotomatiki - Mawasiliano na baharia - Ujumbe wa kiotomatiki namawasiliano na baharia - Wala yeyote - sijui - Kataa kujibu |
| aa. Je! Kuna mtu yeyote aliwai ona jumbe fupi za eletroniki kutokwa kwa utafiti ambazo hukutaka waone? | - Ndio - La - Sijui - Kataa kujibu |
| bb. Je! Hii ilisababisha matokeo yeyote mabaya? | - Ndio - La - sijui - kataa kujibu |
| *cc.* Ikiwa ni Ndio, ni matokeo gani yalitokea? *Sahihisha yote yaliyofanyika* | - Kugundua nina virusi vya ukimwi - Kuulizwa maswali mengi ambayo yalinifanya nisijihisi huru - Ugomvi kati yako na wazazi/walezi wangu - Ugomvi kati yako na mchumba/mpenzi wangu - Ugomvi kati yako na rafiki - zinginezo (eleza: _____________________) - sijui - kataa kujibu |
| *Vidokezo kutokana na mahojiano juu ya urambazaji wa kielektroniki(E-navigation )* |  |

**Urambazaji wa kibinafsi) In-Person Navigation**

| 2. Katika miezi 12 iliyopita wakati ulikuwa katika utafiti,je!ulipokea msaada wa urambazaji wa kibinafsi wa rika? | - Ndio –> endelea kwa 2a. - La –> ruka kwenda 3 - Sijui - Kataa kujibu | | | | | | |
| --- | --- | --- | --- | --- | --- | --- | --- |
| Ningependa uniambie umeridhika vipi na sehemu Fulani za utafiti ukitumia kiwango kifuatacho:  Kuridhika saana, kuridhika wala kuridhika au kutoridhika, kutoridhika, kutoridhika saana. | | | | | | | |
| a. Je! Uliridhika aje na: | *Soma kwa sauti* | | | | | *Usisome kwa sauti* | |
|  | Ridhika kabisaa | ridhika | Ridhika au kutoridhika | sikuridhika | Sikuridhika kabisa | sijui | Kataa kujibu |
| b. Urahisi wa kuwasiliana na habaria? | 🌕 | 🌕 | 🌕 | 🌕 | 🌕 | 🌕 | 🌕 |
| c. Njia ya siri yamawasiliano na vipindi na na habaria? | 🌕 | 🌕 | 🌕 | 🌕 | 🌕 | 🌕 | 🌕 |
| d. Urafiki wa habaria? | 🌕 | 🌕 | 🌕 | 🌕 | 🌕 | 🌕 | 🌕 |
| e. Kufikia uwezo wa habaria? | 🌕 | 🌕 | 🌕 | 🌕 | 🌕 | 🌕 | 🌕 |
| f. Uaminifu wa habaria? | 🌕 | 🌕 | 🌕 | 🌕 | 🌕 | 🌕 | 🌕 |
| g. Usaidizi wa habaria kwako kwa kumeza dawa ? | 🌕 | 🌕 | 🌕 | 🌕 | 🌕 | 🌕 | 🌕 |
| h. Usaidizi wa habaria kwako kwa kukumbuka kuja kwenye ziara yako kliniki | 🌕 | 🌕 | 🌕 | 🌕 | 🌕 | 🌕 | 🌕 |

| i. Uwezo wako wakupata habari zaidi au kuzungumza na mtu katika kliniki kuhusu afya yako? | 🌕 | 🌕 | 🌕 | 🌕 | 🌕 | 🌕 | 🌕 |
| --- | --- | --- | --- | --- | --- | --- | --- |
| j. Kwa ujumla,umeridhika aje na msaada uliopokea kutoka kwa baharia? | 🌕 | 🌕 | 🌕 | 🌕 | 🌕 | 🌕 | 🌕 |
| k. . Kwa jumla umeridhakaje na usaidizi wa habaria kwa afya yako na ustawi? | 🌕 | 🌕 | 🌕 | 🌕 | 🌕 | 🌕 | 🌕 |
|  | Inavutia saana | Inavutia | Inavutia kiasi | Inavutia kidogo tu | Haivutii kabisa | sijui | Kataa kujibu |
| l. Je ungekuwa na nia ya kuendelea kupokea usaidizi wa urambazaji wa kibinafsi siku za usoni ikiwa itawezekana? | 🌕 | 🌕 | 🌕 | 🌕 | 🌕 | 🌕 | 🌕 |
| m. Je! Ulikutana wapi kikawaida na baharia? | - Kwenye kituo cha afya - Nyumbani - Shuleni - Nyinginezo (eleza____) - Sijui - Kataa kujibu | | |  |  |  |  |
| n. Ni mara ngapi wewe na baharia mulikutana kibinafsi? | - Kila siku - Marachache kwa wiki - Kila wiki - Mara chache kw amwezi - Kila mwezi - Chini ya mwezi 🌕 sijui - Kataa kujibu | | |  |  |  |  |
| o. Nimara ngapi kwa jumla unaweza sema wewe na baharia mulikutana? | - Mara 20 na zidi - mara10-19 - Mara 5-9 | | |  |  |  |  |

|  | - Mara 1-4 - sijui - Kataa kujibu |
| --- | --- |
| p. Je! Unaweza sema ziara na baharia zilikuwa mara kwa mara, mara sahihi,sio mara kwa mara ya kutosha? | - Mara kwa mara - Mara sahihi - Sio mara sawa - sijui - kataa kujibu |
| Je! Unaweza sema mazungumzo yako mengi na baharia yalikuwa kupitia kuongea au ujumbe mfupi kwenye simu kuongea kibinafsi au juu ya kiwango sawa kila moja? | - Kuongea na pia ujumbe mfupi kwenye simu - Kuongea kibinafsi - Karibu kiasi sawa ya kila moja - sijui - Kataa kujibu |
| r. Uliridhika kiasi gani na njia ya msingi ya kuwasiliana? | - Ridhika saana - Ridhika - Kuridha wala kutoridhika - Sikuridhika - Sikuridhika saana - Sijui - Kataa kujibu |
| s. Je! Unaweza sema mawasiliano yako na baharia yalikuwa mazuri saana ,mazuri ,mazuri kidogo ,hayakuwa mazuri? | - Ni mzuri kabisaa - Mzuri sana - Mzuri - Sio mzuri kiodgo - Sio mzuri kabisa - Sijui - Kataa kujibu |
| t. Kuna mtu yeyote aliwai ona ujumbe mfupi au kusikia mawasiliano katai yako na habaria ambayo haukutaka mtu asikie/aone? | - Ndio - La - Sijui - Kataa kujibu |
|  |  |
| u. Je! Hii ilisababisha madhara yeyote? | - Ndio - La - sijui - kataa kujibu |
| *v.* Ikiwa ni ndio,aina gani ya madhara?( *sahihisha yoote yaliyotendeka*) | - Kugundua nin virusi vya ukimwi - Kuulizwa maswali mengi ambayo yalinifanya nisijihisi huru - Ugomvi kati yako na wazazi/walezi wangu - Ugomvi kati yako na mchumba/mpenzi wangu - Ugomvi kati yako na rafiki - Zinginezo (eleza: _____________________) - Sijui - Kataa kujibu |
| *Vidokezo vya urambazaji na mhojiwa wa kibinafsi* |  |

| 3. Katika mwezi 12 iliyopita wakati ulikuwa katika utafiti,je!ulipokea msaada wa fedha kwa khudhuria ziara ya kliniki iliyodhibitishwa au kukuwa na viwango vya chini vya virusi? | - Ndio –> endelea kwa 2a. - La –> ruka kwenda 3 - sijui - kataa kujibu | | | | | | |
| --- | --- | --- | --- | --- | --- | --- | --- |
| Ningependa uniambie umeridhika vipi na sehemu Fulani za utafiti ukitumia kiwango kifuatacho:  Kuridhika saana, kuridhika wala kuridhika au kutoridhika, kutoridhika, kutoridhika saana. . | | | | | | | |
| a. Umeridhika vipi na: | *Soma kwa sauti* | | | | | *Usisome kwa sauti* | |
|  | Kuridhika saana | kuridhika | Kuridhika wala kutoridhika | Sikuridhika | Kutoridhika  Kabisa | sijui | Kataa kujibu |
| b. Urahisi wa kupokea pesa kutoka kwa utafiti | 🌕 | 🌕 | 🌕 | 🌕 | 🌕 | 🌕 | 🌕 |
| c. Njia ya siri jinsi pesa zilivyopeanwa? | 🌕 | 🌕 | 🌕 | 🌕 | 🌕 | 🌕 | 🌕 |

**Conditional Cash Transfer**

| d. | Urafiki wa mfanyikazi wa utafiti anaye peana pesa ? | 🌕 | 🌕 | 🌕 | 🌕 | 🌕 | 🌕 | 🌕 |
| --- | --- | --- | --- | --- | --- | --- | --- | --- |
| e. | Uwezo wa kufikia mfanyikazi wa utafiti anayetoa pesa?? | 🌕 | 🌕 | 🌕 | 🌕 | 🌕 | 🌕 | 🌕 |
| f. | Uaminifu wa mfanyikazi wa utafiti anayetoa pesa? | 🌕 | 🌕 | 🌕 | 🌕 | 🌕 | 🌕 | 🌕 |
| g. | Usaidizi wa mfanyikazi wa utafiti anayetoa pesa? | 🌕 | 🌕 | 🌕 | 🌕 | 🌕 | 🌕 | 🌕 |
| h. | Usaidizi wa fedha kwako kumeza dawa zako? | 🌕 | 🌕 | 🌕 | 🌕 | 🌕 | 🌕 | 🌕 |
| i. | Usaidizi wa fedha kwako kukumbuka kuhudhuria ziara zako kwenye kliniki? | 🌕 | 🌕 | 🌕 | 🌕 | 🌕 | 🌕 | 🌕 |
| j. | Uwezo wako wa kupata habari zaidi au kuzungumza na mtu kwenye kliniki kuhusu afya yako? | 🌕 | 🌕 | 🌕 | 🌕 | 🌕 | 🌕 | 🌕 |
| k. | Kwa kumla uliridhika aje na pesa ulizopokea? | 🌕 | 🌕 | 🌕 | 🌕 | 🌕 | 🌕 | 🌕 |
| l. | Kwa ujumla uliridhikaje na usaidizi wa pesa uliyopokea kwa afya yako na ustawi? | 🌕 | 🌕 | 🌕 | 🌕 | 🌕 | 🌕 | 🌕 |

|  | Inavutia saana | inavutia | Inavutia kiasi | Inavutia kidogo tu | Haivutii kabisa | sijui | Kata kujibu |
| --- | --- | --- | --- | --- | --- | --- | --- |
| m. Je ungekuwa na nia ya kuendelea kupokea usaidizi wa pesa kwa siku za usoni ikiwa itawezekama? | 🌕 | 🌕 | 🌕 | 🌕 | 🌕 | 🌕 | 🌕 |
| n. Je ulipokea kiwango cha pesa ulichotarajia kutokwa kwa utafiti? | - Ndio - La - Sijui - Kataa kujibu | |  |  |  |  |  |
| o. Ni pesa ngapi ulipokea kutoka kwa utafiti katika kila ziara iliyodhihirishwa? | - Chini ya mia 500 - Mia 500 - Zaidi ya mia 500 - Sijui - Kataa kujibu | |  |  |  |  |  |
| p. Ni mara ngapi baharia alikutana nawe ? | - Kila siku - Mara chache kwa wiki - Kila wiki - Mara chache kwa mwezi - Kila mwezi - Chini ya mwezi - Sijui - Kataa kujibu | |  |  |  |  |  |
| q. Kwa ujumla ni mapokezi ngapi ya pesa ulipata? | - Mara 10-19 - Mara 5-9 - Mara 1-4 - sijui - kataa kujibu | |  |  |  |  |  |
| r. Je! Kulikuwa na ziara za kiliniki ambazo haukupokea pesa na ilistahili wewe kupokea pesa? | - Ndio - La - Sijui - Kataa kujibu | |  |  |  |  |  |
| s. Ikiwa ni ndio,utafiti ulikuelezea sababu? | - Ndio - La - Sijui - Kataa kujibu | |  |  |  |  |  |

| t. Je! Unaweza sema kuwa mapokezi ya pesa yalikuwa marakwa mara,inavyostahili,sio mara ya kutosha? | - Mara kwa mara - Inavyostahili - Sio mara ya kutosha - Sijui - Kataa kujibu |
| --- | --- |
| u. Je unaweza sema uzoefu wako na mchakato wa kupewa pesa ulikuw amzuri saana,mzuri kabisa,mzuri,sio mzuri,sio mzuri saana? | - Ni mzuri kabisaa - Mzuri sana - Mzuri - Sio mzuri kiodgo - Sio mzuri kabisa - Sijui - Kataa kujibu |
| v. Je unaweza sema pesa ulizopokea zilitosha kugharamia gharama ya kliniki? | - Ndio - La - Sijui - Kataa kujibu |
| w. Kwa hii hakuna jibu sahihi au baya;unaweza sema kuwa pesa ulizopokea zilitumika kwa nauli ya kuenda kliniki,chakula, au nauli pamoja na chakula na mengineyo au haikutumika kwa hayo tuliyoyasema? | - Nauli ya kuenda nakurudi kliniki - Nauli kkenda nakurudi na chakula - Mengineo (eleza______________________) - Hakuna hata moja hapo juu - Sijui - Kataa kujibu |
| x. Je ulipata changamoto yeyote kwa mchakato wa kupeana pesa? | - Ndio - La - Sijui - Kataa kujibu |
| y. Je mchakato wa kupeana pesa ulileta madhara yeyote mabaya? | - Ndio - La - Sijui - Kataa kujibu |
| *z.* Ikiwa ni ndio nimatokeo yapi mbaya yalitokea? *Sahihisha yoote yanostahili)* | - Kugundua nin virusi vya ukimwi - Kuulizwa maswali mengi ambayo yalinifanya nisijihisi huru - Ugomvi kati yako na wazazi/walezi wangu - Ugomvi kati yako na mchumba/mpenzi wangu - Ugomvi kati yako na rafiki - Mengine (eleza: _____________________) - Sijui - Kataa kujibu |
| *Melezo ya mhojiwa Kuhusu uhamisha pesa kwa masharti?* |  |

| 4. Tafadhali fikiria kuhusu huduma za afya ambazo umepokea nje ya utafiti wa A4A:    Wakati ulikuwa umeandikishwa kataka utafiti wa A4A je uliwapokea yafuatayo kutokwa kwa utafiti nyinginezo au programu zingine?Usaidizi wa pesa, jumbe fupi wa kawaida, msaada wa ushauri nasanaa kwa simu/nyumbani na mhudumu wa afya, mfanyikazi wa afya ya jamii au watafiti wengine? Ikiwa ndio ni yapi?*sahihisha yote yanayokubalika* | - Ndio 🡪 sahihisha yote yanayokubalika - La 🡪 enda d - sijui 🡪 enda d - kataa kujibu 🡪 enda d      - Sahihisha yote yanayokubalika - Ujumbe mfupi wa kawaida 🡪 enda a - Usiadizi wa pesa 🡪 enda b - Ushauri wa sanaa 🡪 enda c - Hakuna (tu ikiwa hakuna hapo juu iliyosahihishwa) 🡪 enda d - Sijui 🡪 enda d - Kataa kujibu 🡪 enda d |
| --- | --- |
| a. Ujumbe mfupi Zaidi ya A4A |  |
| i. Mara ngapi? | - Kila siku - Kila wiki - Kila mwezi - Chini ya mwezi - Sijui - Kataa kujibu |
| ii. Je mada ni ipi? (*Sahihisha yoote yanayotumika)* | - Ukumbusho wa ziara - Kukoza ziara tafadhali rudi - Kukuza afya - Majibu yakliniki - Mengine (eleza______________) |
| iii. Ni shirika ua utafiti gani unaotuma? | - Kliniki ninapopokea utunzi wangu - Tafiti zingine (eleza jina___________) - sijui - kataa kujibu |
| b. Usaidizi wa pesa (*Zaidi ya utafiti waA4A*) |  |
| i. Mara ngapi? | 🌕 Kila siku |

|  | - Kila wiki - Kila mwezi - Chini ya mwezi - Sijui - Kataa kujibu |
| --- | --- |
| ii. Uhamisho wapesa ni kiasi gani katika shilingi ya kenya? | - Mia 100 - Mia 200 - Mia 300 - Mia 400 - Mia 500 - Mia 600 - Mia 700 - Nyingine (eleza __________) - Sijui - kataa kujibu |
| iii. Uhamisho wa pesa ni wa nini? (*Sahihisha yote yanayotumika)* | - Kuhudhuria ziara - Kulipia usafiri - Ya wakati kwenye kliniki - Virusi vilivyokandamizwa - Nyingine (eleza ____________) |
| iv. Ni shirika au utafiti gani unaopeana pesa? | - Kliniki ninapopokea utunzi - Tafiti nyingine (eleza jina___________) - Sijui - Kataa kujibu |
| c. Usaidizi wa kibinafsi wa rika (nje ya utafiti wa A4A) | |
| i. Ni mara ngapi? | - Kila siku - Kila wiki - Kila mwezi - Chini ya mwezi - Sijui - Kataa kujibu |
| ii. Je kwa jumla mumekutana kibinafsi au kupitia kwenye simu? | - Simu - Kibinafsi kwenye kliniki/nyumbani - Kwa simu nakibinafsi - sijui - kataa kujibu |
| iii. Eleza msaada wa baharia wa ushauri | _________________________________ |
| iv. Ni shirika au utafiti gani unaopena pesa?? | - Kliniki ninapopokea utunzi - Tafiti nyingine (eleza jina___________) - Sijui - Kataa kujibu |
| d. Je umeandikishwa katika utafiti wowote zaidi ya utafiti wa A4A? | - Ndio 🡪 ikiwa ndio,sahihisha yote yanayotumika - Laa - Sijui - Kataa kujibu |
| i. Ikiwa ndio,eleza jina na utafiti unamoshiriki? | ______________________________ |
| ii. Je iko kaunti ndogo na kituo gani cha afya? | Jina la kituo cha afya ( nyingine kama sio kituo ch afya)  ____________________  Kaunti ndogo ________________  Kaunti ________________ |
| Maoni mengine |  |

CHAKULA NA MALEZI BORA. Tarehe --/--/----	saa ya kuanza --:--	Asubuhi,machana,jioni.

| UTANGULIZI | SOMA KWA SAUTI: Asante kwa kuzungumza nami leo Tunaenda kumaliza utafiti ambao ulisajiliwa Nitakuliza maswali kukuhusu, nyumba yako ya kila siku.Tutaongea juu ya chakula ,afya matibabu yako ya virusi vya Ukimwi na fedha .Majibu yako yote yatakuwa ya siri tafadhali fahamu hayo yote unapo jibu maswali hayo kwa ukweli iwezekanavyo .Kama unaswali yoyote hau haujaela swali nonapo kuuliza ,tafadhali niambie iliniweze kukueleza vizuri.   Tuanze na maswali kuhusu matumizi ya chakula kwa nyumba yako . Ningependa kujua jinsi chakula na njia zinazotumika kupata chakula hiki inaathiri maisha yako. Haya maswali yanahitaji ufikiri nyuma zaidi ya wiki nne zilizopita. |  | | |
| --- | --- | --- | --- | --- |
| A1 | Kwa wiki nne zilizopita ulikuwa na wasiwasi nyumba haita kuwa na chakula cha kutosha ? | 0. La !! Ruka kwensa A2  1. Ndio | | |
| A1.1 | Ni mara ngapi hii imefanyika? | Mara chache , mara moja au mbili kwas wiki nne zilizopita | | |
|  |  | 2. Saa zingine mara tatu hadi kumi hivi. | | |
|  |  | 3. Mara mingi zaidi ya mara kumi kwa wiki nne hivi | | |
|  |  |  | | |
| A2 | Kwa wiki nne zilizopita kuna uwezekano wewe au yeyote kwa familia yako hamukuweza kula aina ya vyakula mungependa kula kwa sababu ya ukosefu wa fedha? | 0. La !! Enda kwa A3  1. Ndio | | |
| A2.1 | Ni mara ngapi hii imefanyika? | Mara chache mara moja au mbili kwa wiki nne zilizo pita | | |
|  |  | Saa zingine mara tatu hadi kumi hivi kwa wiki nne zilizopita | | |
|  |  | Mara mingi zaidi ya mara kumi kwa wiki nne zilizopita | | |
| A3 | Kwa wiki nne zilizopita wewe au yoyote kwa familia yako ilibidi ale chakula aina chache kwa sababu ya ukosefu wa fedha? | 0. La ! Enda kwa A4  1.Ndio | | |
| A3.1 |  | Mara chache mara moja au mbili kwa wiki nne zilizo pita | | |
|  |  | Saa zingine mara tatu hadi kumi hivi kwa wiki nne zilizopita. | | |
|  |  | Mara mingi zaidi ya mara kumi kwa wiki nne zilizopita | | |
| A4 | kwa wiki nne zilizopita wewe au yoyote kwa familia yako ilibidi ale chakula ambacho hakukipenda kwa sababu ya ukosefu wa fedha ? | La enda kwa A5  1.ndiyo | | |
| A4.1 | Hii Ilifanyika mara ngapi? | Mara chache mara moja au mbili kwa wiki nne zilizo pita | | |
|  |  | Saa zingine mara tatu hadi kumi hivi kwa wiki nne zilizopita | | |
|  |  | Mara mingi zaidi ya mara kumi kwa wiki nne zilizopita | | |
|  |  |  |  |  |
| A5 | Kwa wiki nne zilizopita wewe au yoyote kwa familia ilibidi akule chakula kidogo ya ile angependa kula kwa sababu chakula kilikuwa hakitoshi? | La! enda kwa A6  1.Ndio | | |
| A5.1 | Hii Ilifanyika mara ngapi? | Mara chache mara moja au mbili kwa wiki nne zilizo pita | | |
|  |  | Saa zingine mara tatu hadi kumi hivi kwa wiki nne zilizopita | | |
|  |  | Mara mingi zaidi ya mara kumi kwa wiki nne zilizopita | | |
| A6 | Kwa wiki nne zilizopita wewe au yoyote  kwa familia ilibidi akule chakula chache  ya ile angependa kula kwa sababu  chakula kilikuwa hakitoshi? | La! Enda kwa A7  1.Ndio | | |
|  | Hii Ilifanyika mara ngapi? | Mara chache mara moja au mbili kwa wiki nne zilizo pita | | |
|  |  | Saa zingine mara tatu hadi kumi hivi kwa wiki nne zilizopita | | |
|  |  | Mara mingi zaidi ya mara kumi kwa wiki nne zilizopita | | |
| A7 | kwa wiki nne zilizopita,kulikuwa na siku ambapo mulikosa chakula | La! Enda kwa A8  1.Ndio | | |
| A7.1 | Hii ilifanyika mara ngapi? | Mara chache mara moja au mbili kwa wiki nne zilizopita. | | |
|  |  | Saa zingine mara tatu hadi kumi hivi kwa wiki nne zilizopita. | | |
|  |  | Mara mingi zaidi ya mara kumi kwa wiki nne zilizopita. | | |
| A8 | Kwawiki nne zilizopita uliwai lala njaa juu ya kukosa chakula? | 0! Enda A9  1.Ndio | | |
| A8.1 | Hii ilifanyika mara ngapi? | Mara chache mara moja au mbili kwa wiki nne zilizo pita | | |
|  |  | Saa zingine mara tatu hadi kumi hivi kwa wiki nne zilizopita. | | |
|  |  | Mara mingi zaidi ya mara kumi kwa wiki nne zilizopita. | | |
| A9 | Kwa wiki nne zilizopita uliwai shinda njaa mchana yoote na kulala njaa kwa sababu ya kukosa chakula? | La--mwisho  1.ndiyo | | |
| A9.1 | Hii ilifanyika mara ngapi? | Mara chache mara moja au mbili kwa wiki nne zilizo pita  Saa zingine mara tatu hadi kumi hivi kwa wiki nne zilizopita  Mara mingi zaidi ya mara kumi kwa wiki nne zilizopita | | |

| Kwa wiki mbili zilizopita, ni mara ngapi umesumbuliwa na shida zifuatazo?  (Tumia ✔ kuonyesha jibu sahihi) | Hakuna kabisa | Siku kadhaa | Zaidi ya nusu ya siku | Karibu kila siku |
| --- | --- | --- | --- | --- |
| 1. kuhisi wasiwasi na au pembeni | 0 | 1 | 2 | 3 |
| 2. kutokuwa na uwezo wa kudhibiti au kuwacha wasiwasi | 0 | 1 | 2 | 3 |

Jumuisha shida ya wasiwasi GAD 7 (Generalize Anxiety Disorder GAD 7)

| Kwa wiki mbili zilizopita ni mara ngapi  Umesumbuliwa na shida zifuatazo | Sina uhakikakabisa | Siku zoote | Nusu y asiku zoote | Karibu kila siku |
| --- | --- | --- | --- | --- |
| 1. Kuhisi wasiwasi au wasiwasi kuwa pembeni? | 0 | 1 | 2 | 3 |
| 2. 2.kutokuwa na uwezo wa kuwacha au kudhibiti   Wasiwasi? | 0 | 1 | 2 | 3 |
| 3. Kuhofia saana juu ya vitu tofauti? | 0 | 1 | 2 | 3 |
| 4. Shida kupumzika? | 0 | 1 | 2 | 3 |
| 5. kuwa na wasiwasi kwamba ni vigumu kukaa kimya? | 0 | 1 | 2 | 3 |
| 6. Kukasirika kwa urahisi au kukerwa? | 0 | 1 | 2 | 3 |
| 7. Kuhisi uofu kana kwamba kitu kibaya kitatokea/kutendeka | 0 | 1 | 2 | 3 |
|  |  |  |  |  |

Ongeza alama kwa kila safu	+	+	+	+

Jumla ya alama (ongeza alama zote kwa safuwima) =

Ukizikagua hizi shida zoote ni ugumu gani hizi shida zimekuhathiri usifanye kazi yako,kutunza vitu nyumbani au kupatana na watu wengine?

Sio vigumu-------------------------

Vigumu kiasi------------------

Ni vigumu---------------------

Vigumu kabisa-------------------

Source: Spitzer RL, Kroenke K, Williams JBW, Lowe B. A brief measure for assessing generalized anxiety

disorder. *Arch Inern Med.* 2006;166:1092-1097.

**A4A – Socio-Demographic Questionnaire**

| COMPLETE ALL OF THE INFORMATION ON THIS PAGE BEFORE BEGINNING THE INTERVIEW. | | | |
| --- | --- | --- | --- |
| SD-A-ID | Study subject identification number | □□□-□□□ | |
| SD-A-SITE | Study site | Site 1  Site 2  Site 3  Site 4  Site 5  Site 6 | 1  2  3  4  5  6 |
| SD-A-ENGAGE | Engagement status  (of participant) | Retained in care  Lost to follow up | 1  2 |
| SD-A-SEX | Study subject sex | Male  Female | 1  2 |
| SD-A-LANG | Language of the interview | English  Kiswahili  Dholuo | 1  2  3 |
| SD-A-DATE | Date of assessment | DAY MONTH YEAR | |
| SD-A-RA | Research assistant initials |  | |

*INSTRUCTIONS:* *Interviewer instructions are in italic print—these are for your use and should not be read aloud. If necessary, you may read the options of questions to prompt the respondent. Read all of the questions and bolded non-italic print aloud to the respondent. Please read all information as it is written. Do not read aloud the answer options of “Don’t know” or “Refused”. Only circle the number next to the option “Don’t know” if the respondent states he/she doesn’t know. Only circle the number next to the “Refused” option if the respondent states he/she does not want to answer. For questions that require you to write an answer rather than circle a response category on the form, or when respondent answers “Other” and provides his/her own answer, please record respondent’s comments IN CAPITAL LETTERS as directly and carefully as possible.*

*CHIKE MILUWO:*

**Erokamano kendo kuom yie mondo idonj e nonro. Penjo moko adwa penji to gin maling ling kendo samoro ginyalo miyo ibedgi wichkuot kata ginyalo miyo kik ibed thuolo. Inyalo weyo duoko penjo ma oki dwar. Inyalo nyisa mondo alerni penjo ma okiwinji. Inyalo weyo nonro saa asaya ka okidwar dhi mbele. Kiyie to par ni duokoni e penjo gi ingeyo gi number momiyi kendo ikano gi maling ling. Nyingi okwandiki e obokeni.**

| No. | **QUESTIONS & INSTRUCTIONS** | RESPONSES |  | SKIPS |
| --- | --- | --- | --- | --- |
| ***DEMOGRAPHICS***  Wachak gi penjo matin mar ngei. | | | | |
| 1. | Sani in kod higni adi?  *Enter response in years. If respondent answers “don’t know”, probe to nearest 5 years. If s/he refuses to answer, enter 99. If the respondent gives the year of birth, calculate the age and enter. If the participant provides only the date of birth, enter the information in the space provided and calculate the age after the interview.* | Years  DIA MÊS ANO  *DATE OF BIRTH*  DAY MONTH YEAR |  |  |
| 2. | Sani be idhi kar tiegruok? | Kamano  Ooyo  *okangenyo*  *Adagi* | 1  2  *88*  *99* | *If 1, 88,99 🡪 Q4* |
| 3. | Ango moomiyo okidhi e kar tiegruok/ mbalariany? | Asebedo ka atuoI  Okahero kar tiegruok  An jarit mar achiel kuom joodwa  Onge omenda moromo  Kar tiegruok bor  Atiyo  Asetieko somba  *Okangeyo*  *Adagi* | 1  2  3  4  5  6  *7*  *88*  *99* |  |
| 4. | Sombi ochopo e kiwango mane?  *Kik isom duoko gi matek. Luor duoko achiel* | Okasomo  Kar tiegruok mapiny (1^st^ – 8^th^ class)  Kar tiegruok mediere (Form 1-4)  Kar tiegruok mamalo  Mbalariany  *Okangeyo*  *Adagi* | 1  2  3  4  5  *88*  *99* |  |
| 5. | Sani be in e osiep moro amora? Mae nyalo bedo jaodi kata ngato minyalo luongo jaodi? | Kamano  Ooyo  Okangeyo  Adagi | 1  2  *88*  *99* | If 2 🡪 Q6 |
| 6. | (*Penje ka en e osiep gi ngato*)  In gi ji adi miriwori godo/ jaodi? |  | 1  2  3  4  5  88  99 |  |
| 7. | En Osiep mane manitie e kindi gi jaodi?  *(Penj kae gi ji moingo achiel, non matut kuom osiepe maduong.* | Osenyuoma/ asenyombo wadak kanyakla  Osekenda/ asekendo ok wadak kanyakla  Wadak kanyakla to ok wasekendore  Ok wadak kanyakla  Wawere/ keny nokethore  Jaoda nosetho  *Okangeyo*  *Adagi* | 1  2  3  4  *5*  *6*  *88*  *99* |  |
|  | Ent ich mane mitimo ma kawo thuoloni godiochieng?  *Kik isomo duoko gi. Luor achiel* | An nyathi tiegruok  Aonge tich  An chi ot  An japur  Auso e (chiro, e ndara)  An jatich mar ot(aluoko ot, atedo, arito nyithindo)  Ondika tich masomo (japuonj, Juoga aktarin  Aloso wich/ angiyo wach ber  Arito  Atiyo kar kedo  Ariembo mtokni  Mamoko______________________  *Akia*  *Adagi* | 1  2  3  4  5  6  7 8  9  10  11  12  *88*  *99* |  |
| OSIEP MAE KIND NYATHI GI JARIT KAKA OBEDO  Sani adwa penji penjo kuom odi | | | | |
| 8. | Ere kaka upangoru kuom nyuol e odu? | 1^st^ born  2^nd^ born  3^rd^ born  4^th^ born  5^th^ born  Last born  Moko (wachi ___)  *Specify the birth order number if not 1-5 or last born*  Okangeyo  Adagi | 1  2  3  4  5  6  7  88  99 |  |
| 9. | Ere yoo maber ma inyalo pimonwa kaka udak?  *Allow respondent to describe and RA to code appropriate response. Do not read options aloud.* | Wadak e ot achiel gi jonyuol kod nyithindwa  Wadak e ot achiel gi jonyuol wa, nyithindwa kod wedewa  Wadak e ot achiel to ok wan wede wadak kar tiegruok  Mamoko wachi:_______________  Waonge ot wadak e ndara  *Okangeyo*  *Adagi* | 12  3  4  5  *6*  *7*  *88*  *99* |  |
| 10. | Ngat mane ma okawo patik mar riti kendo ngiyo kaka idak? | An  Minwa  wuonwa  Waya/ Nera  Kwara/ Dana  Nyithindwa  Jaoda  Wedena mamoko  Jaritna, ma ok watna  *Okangeyo*  *Adagi* | 1  2  3  4  5  6  7  8  *9*  *88*  *99* | *If 1, 88,99 🡪 Q12* |
| 11. | Be odak kodi e ot midakeno? | Kamano  Ooyo  *Okangeyo*  *Adagi* | 1  2  *88*  *99* |  |
| 12. | Ka ikwanori kisechopo higni 18, Un ji adi madongo modak e odno sani? | Adult  *Okangeyo*  *Adagi* | 88  99 |  |
| 13. | Kwanri ka pok ichopo higni 18, Un nyithindo adi ma pok ochopo e od no sani? | Nyithindo  *Okangeyo*  *Adagi* | *88*  *99* |  |
| 14. | Kigoyo kwano e dwe, iyudo pesa adi e yor yuto mari, to yuto mane ma jo odi loso? (Riw anena yuto go tee)  *If the participant needs help, read options aloud. Circle one response. Enter amount in the space provided. If respondent answers “Don’t know”, enter 88. If respondent refuses to answer, enter 99.* | Less than 1000 Kshs  1000 – 3000 Kshs  4000 – 8000 Kshs  8000 to 15000 Kshs  More than 15000 Kshs  *Okangeyo*  *Adagi* | 1  2  3  4  5  *88*  *99* |  |
| 15. | E seche 12 mokalo, ndalo adi ma jooogi osebedo maonge chiemo moromo?  *Luor duoko achiel* | Podi  Samoro  Seche tee  *Okangeyo*  *Adagi* | 1  2  3  *88*  *99* |  |
| 16. | Pii modh ugolo Kanye e odu kanyo?  *Som matek duoko mondik go kae iluor achiel* | Bugo mokunyne jii tee  Bugo makunya matiyo godo kenda  Fereji moketne molosne jii tee  Fereji ma aloso madonjo e oda  Mamoko, _____________________  *Okangeyo*  *Adagi* | 1  2  3  4  5  *88*  *99* |  |
| 17. | Be itiyo gi taya e dala?  *Luor achiel kuom duoko miwacho.* | Eee  Ooyo  *Okangeyo*  *Adagi* | 1  2  *88*  *99* |  |
| 18. | Be in kod ongwe yamo ma mari?  *Luor duoko mowinjore* | Kamano  Ooyo  *Okangeyo*  *Adagi* | 1  2  *88*  *99* | *If yes, Q23* |
| 19. | *Ka ionge ongwe yamo ma mare: Bin utiyo gi ongwe yamo achiel kanyakla, to bende iyudo thuolo mar tiyo ongwe yamo mutiyo godo kanyakla?* | Kamano  Ooyo  *Okangeyo*  *Adagi* | 1  2  *88*  *99* |  |
| *SEXUAL HISTORY AND RELATIONSHIPTERUOK KOD OSIEP*  Inyalo yudo ka penjo ma apenji gi gin kuom ngimani kendo malingling. Agoyoni erokamano kuom adiera mari kendo wecheni idhi kan maling ling. Wadwa penji penjo kuom osiepi kod riwruok mar osiep. | | | | |
| 20. | Be ingeyo gima riwruok ter ruok/ riwruok en?  *Luor duoko achiel* | Kamano  Ooyo  *Okangeyo*  *Adagi* | 1  2  *88*  *99* |  |
| 21. | Be is iseriwori gin nyako/ wuoi?  *Luor duoko achiel* | Kamano  Ooyo  *Okangeyo*  *Adagi* | 1  2  *88*  *99* | If 2 🡪 Q34 |
| 22. | Ne in kod higni adi mane iriwori mokuongo? | Higa  DIA MÊS ANO  *Okangeyo*  *Adagi* | *88*  *99* |  |
| 23. | Mane iriwogi gi nyako / wuoi mokuongo, ne en hero mari koso nochuni godo?  *Luor duoko achiel* | Nadwaro  Nochuna  *Okangeyo*  *Adagi* | 1  2  *88*  *99* |  |
| 24. | Kikwano, niseriwori gi ji adi mopogore? Kiyie to miwa paro mowinjore | Number  *Okangeyo*  *Adagi* | *88*  *99* |  |
| 25. | Bin nitiyo gi rabo yunga mane iriwori mogik?  *Luor duoko achiel* | Kamano  Ooyo  Okanyal paro  *Okangeyo*  *Adagi* | 1  2  3  *88*  *99* |  |
| 26. | Be in kata jaodi sani tiyo gi yo moro mar gengo gamo remo?  *Luor duoko achiel* | Kamano  Ooyo No  Oonge ngato moriwore godo/ ok ondiki Be intiere gi ja ngetni?  *Okangeyo*  *Adagi* | 1  2  3  *88*  *99* | If 2, 3, 88, 99 🡪 Q31 |
| 27. | Ka itiyo gi yoo ma leo kata magengo gamo remo, en yoo mane mitiyo godo kata ma jaodi tiyo godo?    *Luor mowacho tee* | Gima isoyo eduong  Mine mar gengo gamo remo  Sindan  Yath amuonya  Rabo yunga Condom  Yoo ma silili  Golo duong sama jiriwore  Weyo riwruok  Golo dhako e ot  Golo dichuo e ot  Mamoko______________  *Okangeyo*  *Adagi* | 1  2  3  4  5  6  7  8  9  10  *88*  *99* |  |
| 28. | Iyach sani?  *Luor duoko achiel* | Kamano  Ooyo  *Okangeyo*  *Adagi* | 1  2  88  99 |  |
| 29. | Be nise ga gamo remo e ngimani?  *Luor achiel* | Kamano  Ooyo  *Okangeyo*  *okangeyo*  kwan | 1  2  *88*  *99* | If 2 🡪 Q34  *Mae mar mine kende For females only* |
| 30. | Ka nisegamo remo, isenyuolo nyithindo adi? | *Okangeyo*  *Adagi* | *88*  *99* | *For females only* |
| 31. | Nyithindi nigi higni adi sani? | *Code don’t know 88 or refused 99 per child as needed*  Higa  Higa  Higa  Higa  Inyalo ndiko kae koponi kwan ok donji |  |  |
| 32. | Bende osechuoyi sandan magengo tuo mamako duong mine?  *Ndik kamano kosechuoye sandan(nyalo bedo sindan achiel kata ariyo kaluore gi hike)* | *Kamano*  *Ooyo*  *Okangeyo*  *Adagi* | 1  2  88  99 | *Ask only females* |
| *NGIMA MAR JOGO ODAK E ODNO*  *CHENRO MILUWO:*  Penjo ma matin ma adwa penji gin kuom ngima ni kod ngima mar jo odi | | | | |
| 32 | Kuon ngeyo mari, mopogore kodi, ngato mane kendo mosepim moyud gi kute mag ayaki?  *Luor duoko tee mowacho*  *Non malongo ka nitie* ngato moro kendo?  *Ka nitie duoko ma okondik kanyo, ndik kama ondikni duoko mamoko* | Onge kata achiel  Wuon AYA’s  Min AYA’s  Nyithindgi AYA’s  Wede AYA’s mamoko  Jaod AYA’s  Mamoko _______________________  *Okangeyo*  *Adagi* | 1  2  3  4  5  6  7  88  99 | *If = 1 🡪 Q34* |
| 33. | Kuom ngeyo mari, mopogore kodi, nga kendo matiyo gi yedhe kute ayaki?  *Luor duoko tee mowacho*  *N*on matut: Nga kendo?  *ka jachiw duoko owacho duoko maongo kae, ndiki kar duoko mamoko* | Onge  Wuon AYA’s  Min AYA’s  Nyithindgi AYA’s  Wede AYA’s  Jaod /osiep AYA’s  Duoko mamoko__________________  *Okangeyo*  *Adagi* | 1  2  3  4  5  6  7  88  99 |  |
| *CHAL MAR KAR THIETH KATA OSUPTAL*  Wadwa penji penjo moko kuom kar thieth midhie kata mane indhie ndalo machon | | | | |
| 34 | Ngat mane materi ga kar thieth kao yien?  *Luo duoko tee mowacho* | An  Minwa  Wuonwa  Waya / nera  Kwara/ dana  Nyithindwa  Wedena mamoko  Jaoda/osiepa  Jaritna, ngato ma ok watna  Onge kar thieth ma adhie sani  *Okangeyo*  *Adagi* | 1  2  3  4  5  6  7  8  9  10  *88*  *99* |  |
| 35. | Ikawo saa marom nade mondo ichop kar thieth kawo yedhe ni mag kute mar ayaki, dhi ogalko. Kiyie to wach thuolo mane ikao kirito kaka ichopo e osuptal nyaka saa mane iwuotho michopo kar thieth.  **Ka rawera sani ok dhi kar thieth, penji kama ne nyathi dhi kawe yath.*  *Enter the amount of time in the space provided. If the time is less than 1 hour, enter 00 and then the number of minutes. If respondent answers “Don’t know”, enter 88. If respondent refuses to answer, enter 99 99.* | HOURS : MINUTES  *Okangeyo*  *Adagi* | *88*  *99* |  |
| 36. | Iparoni omenda adi manyalo chopi kar thieth kidhi ogalko?    *Ka rewera ok dhi kar thieth penj ka nyathi be dhi kao yien.*  *Enter amount in the space provided. If respondent answers “Don’t know”, enter 8888. If respondent refuses to answer, enter 9999.* | Sendi mar kenya  *Okangeyo*  *Adagi* | *88*  *99* |  |
| 37. | *:Ne rewera mane oweyo dhi mbele gi thieth:*    Ango mane omiyo iweyo dhi mbele gi kao yien kar thieth?  *Luor duoko tee mochiwo*    *Ka nyathi dhi mbele gi kao yath, kik ipenj penjo ni, dhi e penjo machielo.* | **Yore mag dhi kawo yien**  koso yoo ma achop godo e osuptal  Omenda ma chopa kar thieth  Ne aonge thuolo moroma chopo kar thieth.  Weche mamoko (wachi ______________)  **Tich,kar tiegruok kod jo ot.**  Kethona tich  School interference w/refill pick up Lewo skul ka adhi kao yath  Lewo ne skul ka adhi e osuptal  Ketho tiegruok ka adhi manyo yien  Jarit AYA/ ka odhi e liel  Jarit AYA ne nigi tije mag ode mamoko  Jaoda ne ok dwar ni mondo adhi e kar thieth  Dak, nyuol jarit olokore  (Mamoko wachi______________)  Jo ot mamoko/ tich, yier mowinjore  **Medical Thieth**  Felt too sick to come to clinic  Jaritna netuo matek mmane ok onyal biro kel e thieth. osuptal  Yath ne inya  Pek mar yudo yath  Nadwaro yweyo ne yath  Nayach/ nanyuol  Weche tuoche mamoko, wachi  Wech mamoko, wachi___  Orwaki e e kar thieth  🡪 Tarik moketi e osuptal: ______/________/________  DD /MM /YYYY  Nying osuptal :  Gimomiyo orwaki kar thieth:    **Chal mar kar thieth**  Yedhe orumo e kar thieth  Loko kar thieth kowuok ir rawera kadhi kuom ngato maduong  Jo kar thieth ne ok ber  Rit ne ok ber  Ne idwaro jok matelo ne yath mangeny  Kuma ne irite ajuoga  Kar thieth ne opong  Nyaka arit aminga ka abiro neno ajuoga  I didn’t want to go to retention class nokadwar dhi e klas ma mipuonjo ji wach yath  **Wich kod ngiyo**  Nawinjo maber , ne onge tiende dhi kae yath.  Ka adhi kao yatha jogweng biro ngeyo ni an gi kute mag ayaki.  Jo kar thiethl biro dhao na ka alewo ne neno lsaktar.  Ne adhi kuom ja lemo mane temo thiedha kod lamo.  Nikech aneno/ asebedo ka aneno jajuoga  Awinjo ka aonge genokendo piny ochama  Madho kongo mangeny  Wiya nowil gi odiochienga mar kao yath.  Jo odwa kod jogo ma otelona owacho ni kik nyathino dhi mbele gi neno ajuona.  *Okangeyo*  *Adagi* | 1  2  3  4  5  6  7  8  9  10  11  12  13  14  15  16  17  18  19  20  21  22  23  24  25  26  27  28  29  30  30  31  32  33  34  35  36  37  88  99 | *Ne jonyuol mane oweyo kao yien*  *Multiple select*  *If child retained in care 🡪 mana ka nyathi dhi mbele gi yath*  *Stigma section* |

| LUORO  Penjo ma adwa penji gi gin kuom pachi kendo kaka iwinjo kaka ji timore kodi ka giwinjo ni in kod kute mag ayaki. Kiyie to par ni onge duoko marach kata maber kendo duoko michiwo wadhi kano maling ling. Ka nitie achil kuom penjo gi ma ok mori to wachi mondo akal penjo no. | | | | | |
| --- | --- | --- | --- | --- | --- |
| No. | Questions & Instructions | Responses | | Skips | No. |
| 33. | Be in gi kibaji ni ji kata jirandi biro fwenyo chal mari koluwore gi kute mag ayaki ka ok inyiso gi?  *Som ne matek duoko kae iluor duoko momiyi* | An gi kibaji  Aluor  Okaluor  *Okangeyo*  *Adagi* | | 1  2  3  *88*  *99* |  |
| Adwaro somo ni weche gi. Kiyie to nyisa ka iyie kata idagi | | Agree | Disagree | *Don’t know* | *Refused* |
| 34. | Asewit osiepe kaluwore gi kute mag ayaki | 1 | 2 | *88* | *99* |
| 35. | Oseluonga nyinge, osesanda kendo oseyanya nikech an kod kute mag ayaki. | 1 | 2 | *88* | *99* |
| 36. | Osejara e dala kae nikech an kod kute mag ayaki | 1 | 2 | *88* | *99* |
| 37. | Osechaya kar tiegruok nikech chal mara mar kute mag ayaki | 1 | 2 | *88* | *99* |
| 38. | Osejara e gweng nikech chal na mar kute mag ayaki. | 1 | 2 | *88* | *99* |
| 39. | Osejara kar thieth nikech chal mara mar kute mag ayaki. | 1 | 2 | *88* | *99* |
| 40. | Ajara mar kute mag ayaki osemiyo abed gi midhusi kendo siso. | 1 | 2 | *88* | *99* |
| 41. | Ajara mar kute osemiyo abedo gi paro kod kuyo | 1 | 2 | *88* | *99* |
| 42. | Nayiero mondo kik atug gi jomamoko, okabed kod jii kendo okawuothi nikech chalna mar kute. | 1 | 2 | *88* | *99* |
| 43. | Aneno ni onengo aket chal na maling ling | 1 | 2 | *88* | *99* |
| 44. | Paro na mambele oselokore marach nikech chalna mar kute | 1 | 2 | *88* | *99* |
| 45. | Amuonyo yadha kolewo nikech okadwar ni mondo jii onena ka amuonyo. | 1 | 2 | *88* | *99* |
| 46. | Thoth jii e gweng paro ni yudo kute mag ayaki to timbe ni richo kata ngato ma yombe yombe. | 1 | 2 | *88* | *99* |

| H/A PARO KOD YIE  PENJO GI WUOYO KUOM PARO KOD GENO MAR H/A  Agombo ngeyo yie mari kod pachi kuom kute mag ayaki, kuom thieth kuom kony magichiwo kod thieth magichiwo ne jok manigi kute mag ayaki. Abiro somo ni weche moko kae kendo daher mondo inyisa ka iyie kata okiyie kodgi achiel kachiel. Kiyie to par ni onge duoko maber kata marach, gimoro amora miwacho wadhi kano maling ling kendo ok dhi kethoni yor thieth miyudo kodok korka kute mag ayaki. Kaponi nitie penjo maok miyi ibed thuolo to nyisa mondo akal penjono. | | | | | |
| --- | --- | --- | --- | --- | --- |
| *Som weche gi ahiel kachiel ne ja nonro, then ilu gi “be iyie koso idagi”* | | Agree | Disagree | Don’t know | Refused |
| 47. | Ngato ma nigi kute mag ayaki nyalo dak aminga kendo gi ngima maber mana ka gimuonyo yath | 1 | 2 | *88* | *99* |
| 48. | Yedhe mag ayaki ok kony | 1 | 2 | *88* | *99* |
| 49. | Jomoko mamuonyo yedhe mag ayaki ok nyal miyo jaode kute mag ayaki sama giriwore. | 1 | 2 | *88* | *99* |
| 50. | Kopimi moyudi gi kute, onge tiende chako kao yien gi eseche go ka oponi iwinjo maber. | 1 | 2 | *88* | *99* |
| 51. | Yedhe nyaluo ok ber kaka andila mathiedho kute mag ayaki | 1 | 2 | *88* | *99* |
| 52. | Yedhe kute mag ayaki nyalo konyo mine manyuol nyithindo maonge kute. | 1 | 2 | *88* | *99* |
| 53. | Ka ji oluwo yore mag muonyo ngima gi biro bedo maber. | 1 | 2 | *88* | *99* |
| 54. | Ayaki tinde ok neki nikech yath mimuonyo. | 1 | 2 | *88* | *99* |
| 55. | Ajuoke tinde thiedho jok manigi kute mag ayaki. | 1 | 2 | *88* | *99* |
| 56. | Ngato manigi kute mag ayaki nyalo chako dhi kar thieth mana kowinjo marach. | 1 | 2 | *88* | *99* |
| 57. | Yedhe ayaki onge tiende muonyo nikech rach mokelo e del. | 1 | 2 | *88* | *99* |
| 58. | Yadh ayaki biro keti buoch | 1 | 2 | *88* | *99* |
| 59. | Jogo manigi kute mag ayaki nyalo dak aminga ka giyudo thieth kuwuok kuom nyadh nyaluo. | 1 | 2 | 3 | *99* |
| 60. | Ka ngato manigi kute mag ayaki winjo maber onge tiende dhi kar thieth. | 1 | 2 | 3 | *99* |
| 61. | Kute mag ayaki ok lich ruok ka jii kaone yedhe nyaluo | 1 | 2 | 3 | *99* |
| 62. | ART is only given to persons with HIV who are feeling ill. Yedhe mag ayaki jogo manigi kute mag ayaki matuo | 1 | 2 | 3 | *99* |
| 63. | Yedhe mag ayaki ok bi miyo adag aminga | 1 | 2 | 3 | *99* |

*wach mar ja taa nonro :*

*Ja ta nonro ne en ______ otimo ango	_____ okotim ango	ka nokotimo ango momiyo:*

*Bende ja nonro ne nigi paro marach sama nimiye penjo?? ______ kamano	______ ooyo*

*Ka kamano wachi:*

*Gimoro amora mineno molombo wangi/ ndik kae:*

*Koro adhi penji penjo moko kuom kaka imuonyo yath.*

| 1. Be orwaki echenro mar pith maber mar kaka ichiemo ? □ Ee □ Ooyo | |
| --- | --- |
| OBOKE MAR PENJO MAG NONRO EWI KAKA IMUONYO YATH | |
| 2. Bende in ga gi chandruoge mag rito saa gi yedhe?  □ Ee □ Ooyo  Kinde mage? □ Okinyi □ Odhiambo □Giko juma  □Odiechienge mag juma □Mamoko:____________________ | 3. Be in ga gi chandruoge gi muonyo yath?  □ Ee □ Ooyo  Gin chandruoge mage ma in go? (ler) |
| 4. Seche moko muonyo yath nyalo bedo matek. Bende iseweyo ma ok imuonyo yedhe nikech mora amora kuom wechegi:  □ Wiya nowil muonyo yath □ Abedo mool gi muonyo yath pile kapile.  □ Ne an eskul/tich/ne aonge dala □ Na adagi muonyo yath  □ Ne ok dwa ni jomoko one □ Ayudo ka yedhe kech ahinya  □ Yath miyo awinjo ka atuo □ Mamoko (ler):_________________________  □ Na awinjo katuo kata ng’ok  □ Ok anyal muonyo maonge chiemo  □Onge moro amora kuom man malo | |
| 5. Seche moko, chandruoge eklinik miyo muonyo yedhe gi odiechieng’ kodiechieng’ bedo matek. Be moro amora kuom gigi osebedo chandruok ne in:  □ Yath ne onge ekar yath *Yath mane?* □ Yedhe Magayo kute mag Ayaki □ Septrin □ Mamoko (riw abx)  □ Na atieko kata na atieko yedhe  □ Kinde mar rito ne bor ahinya kendo ne awuok  □ Mamoko(ler) __________________________  □ Onge kuom man malogi | |
| Wadhi penji penjo matin kuom koso muonyo yedhe kaka dwarore. Koso muonyo yath kaka dwarore “dos” nyiso ni yath moro amora monego imuonyo okinyi kata odhiambo. Ka ne imuonyo yedhegi to ikoso ma ok imuonyo kata yath achiel godhiambo, mano biro bedo ni imuonyo dos achiel to ikoso dos achiel. Ka ikoso muonyo yath moro amora okinyi kendo ikoso muonyo kendo godhiambo, mano biro bedo ni ikoso muonyo dos ariyo. Ka in gi penjo kuom muonyo yath kaka owinjore “dos”, yie ipenj kaka wamedo dhi. | |
| 6. Ejuma makalo,  a. Kuom odiechienge adi mane ikoso muonyo yath katin to achiel? □0 □1 □2 □3 □4 □5 □6 □7 □ Akia  b. Kuom odiechienge adi mane imuonyo yath molewo gi saa mohewo saa achiel? □0 □1 □2 □3 □4 □5 □6 □7 □ Akia  c. How many extra doses or syringes of medicine did you take?Ni imuonyo kata madho yedhe mang’eny mohewo mane onego imuony kata sirenj adi? _________________________ □ Don’t know Akia | |
| 7. Gin yedhe “dos” adi mane ikoso muonyo edwe mokadho? ___________ □ Akia | |

**ADAPT-R Enhanced Retrospective Review – hospitalizations**

Demographics

3. Adapt study identifier
4. Names

Administrative

2. Date of interview
3. Interviewer initials

**Healthcare utilization in the past 30 days**

| Tuo kata inyruok e ndalo 30 mokalo | | | | | | | | | | | | | | | | |
| --- | --- | --- | --- | --- | --- | --- | --- | --- | --- | --- | --- | --- | --- | --- | --- | --- |
|  | Endalo 30 mokalo, bende nichandori gi tuo kata inyruok kaka aonda, athunga, diep kata masira moro amora Kata tuo moro amora?  1 Kamano, tuo  Yes, illness  2 Kamano, inyruok  Yes, injury  3 Kamano, tuo gi inyruok  Yes, illness and injury  4 Ooyo  **àDhi e penjo mar wod** | Kawachako gi gik maracha kabisa en tuo abila mane mochami?  Wach tuoche adek manigi ranyisi gi manitiere e obokewani.  *Ndik tuoche 3 gi ranyisi gi. Nee nyinge mondiki* | | | Nidhi kar thieth makanye?Where did you go for treatment?  Ka onge thieth If no treatment, ango mane omoni dhi e thieth?was sought, why didn’t you go for treatment?  *Wach tuoche adek , ka onge thieth mane otim ti gi wach mar onge kar ndiko gimomiyo.* | | | Omenda adi mane ichulo sama nithiedhiTotal amount spent for medical care (e omenda mar Kenya)  Ka kony nochiw nono ndik “O” | | | | | | Ndik kwan seche mane ikao kithiedhi. | Kwan tee seche mane iketho ma ok ma pile kuom tiegruok, e tich kata e ot | Bin nitie tuo/ inyruok mane otimi ndalo 30 mokalo?  Kamano 🡪 *Dhi e laini machielo, penjo 3a e penjo marach moloyo kanyo nyaka 3kuom ngato.*  Ooyo 🡪 Dhi e penjo mar rwako nfgato e wodi |
|  |  |  |  |  |  |  |  | Nidhi manyo thieth. (Dhi gi duogo) | omeda mar neno ajuoga | Yedhe | Dhi e pim | Chulo omenda mar rwako ngato e wodi. *(otingo chiemo)* | Gik mamoko msg ngima |  |  |  |
| 1 | 2 | 3a | 3b | 3c | 4a | 4b | 4c | 5 | 6 | 7 | 8 | 9 | 10 | 11 | 12 | 13 |
| Episode 1 |  |  |  |  |  |  |  |  |  |  |  |  |  |  |  |  |
| Episode 2 |  |  |  |  |  |  |  |  |  |  |  |  |  |  |  |  |
| Episode 3 |  |  |  |  |  |  |  |  |  |  |  |  |  |  |  |  |

**Hospitalizations in the past 12 months**

| E higa achiel mokaloni, di di ma oserwaki e ward? Nabedo kar thieth (Otieno mangima?)  Ka duoko en “0” *à Tiek penjo* | 2. Starting from the most recent hospitalization, chak gi dak e osuptal machiegni  - Ne oyud ni ango matimi mane orwaki e ward? *Ndik tuo mane oyudi/ chal mar tuo mane orwake ne e wod*.  - How many days did you spend in the hospital?  *Ndiki kar thieth 3 , chak gi machiegni* | | | | | | | |
| --- | --- | --- | --- | --- | --- | --- | --- | --- |
|  | Od thieth 1 (most recent) | | | Od thieth 2 | | | Od thieth 3 | |
|  | a. Tuo | | b. Ndalo | c. Tuo | d. Ndalo | | e. Tuo | f. Ndalo |
|  |  | |  |  |  | |  |  |
|  |  | |  |  |  | |  |  |
|  |  | |  |  |  | |  |  |
| Tuoche kos ranyisi magi:   75. Piny ich maremo 76. Masira 77. Tuo mar tielo 78. Gamo yweyo 79. Wang 80. Cancer 81. Winjo koyo kata liet 82. Fuolo 83. Fuolo remo 84. Lak 85. Tuo sukari 86. Diewo matin 87. Diewo (mangeny, dwe1 kata mokalo) 88. Goruok piny ma paro lal 89. Del machwakore 90. Koyo kod liet(mabiro to lal saa tee) 91. Tur 92. Del maremo 93. Adhola e duong | | 94. Kute mag ayaki 95. Wich bar (malit) 96. Tuo mar adundo 97. Tuo mikelo gi suna 98. Tuo mamamo nyithindo 99. Wich mowilore 100. Rem kilayo 101. Tuo obo mag kor 102. Tuoche maluore gi gamo remo 103. Duol malit 104. Del makethore 105. Tuo mar kor 106. Leny 107. Ngok 108. Del bedo mayom yom 109. Wito ratil 110. Adhola 111. Mamoko (Wachi) | | | | Provider codes   21. Kar thieth 22. Dala (Jo ot) 23. Dala ajuoga 24. Ajuoga, oko mar dala 25. Karthieth michulo 26. Kar thieth mar sirikal 27. Duka yath 28. Kar yien 29. Kar nyiepo 30. Mamoko(Wach kar thieth nitie) 25. Onge –Omenda ni malo okanyal dhi manye thieth 26. Onge – Obor gi kama anyalo yude ja thieth 27. Onge –Nokawinjo ka auo. 28. Onge – Ne an kod tije mamoko kaka mar tiegruok, tich mondika, tije ot. 29. Onge – MamokoOther reason (Wach gimomiyo nokidhi manyo thieth. | | |

**General Health Assessment for Children (GHAC)**

**Quality of Life Questionnaire – Adolescent**

5. Since the last visit, have any of the following occurred?

At enrollment: Have any of the following occurred within the last 12 months?

Chakre chieng mane ibiro mogik, be gik ma kamagi osetimore ni?

Saa mane ichako nonro, be gigi osetimore ni e dweche 12 gi?

uu. In, janyuol kata jaodi osewito tije?	◻ Kamano	◻ Ooyo
vv. In, janyuol kata jaodi ochako tich manyien?	◻ Kamano	◻ Ooyo
ww. Ngato osewuok oweyo dalau?	◻ Kamano	◻ Ooyo
xx. Wito ot kata dar	◻ Kamano	◻ Ooyo
yy. Wito kony kaka kony mag chiemo	◻ Kamano	◻ Ooyo

kuom migao mar bedo kanyakla

zz. Wuok e migao mar rawera gi donjo e migao mar

joma dongo	◻ Kamano	◻Ooyo

aaa. Wito migao machudo e yor thieth	◻ Kamano	◻ Ooyo
bbb. Ngato osebedo matuo morwak kaer thieth?	◻ Kamano	◻ Ooyo
ccc. Achiel kuomu osebedo matuo matek?	◻ Kamano	◻ Ooyo
ddd. Wilo jarit	◻ Kamano	◻ Ooyo
eee. Pogruok kuom joot kata jonyuol	◻ Kamano	◻ Ooyo
fff. Ketho keny mar jomosedak kata jonyuol	◻ Kamano	◻ Ooyo
ggg. Oserwaki e od ongeche kata jaritni, jaodi	◻ Kamano	◻ Ooyo
hhh. Okendi/	ikendo kata jarit ni	◻ Kamano	◻ Ooyo
iii. Nyuolo nyithindu	◻ Kamamo	◻ Ooyo
jjj. Nyuolo nyathi	◻ Kamano	◻ Ooyo
kkk. Miyo ochako tich	◻ Kamano	◻ Ooyo
lll. Chako kkar tiegruok kata mbalariany	◻ Kamano	◻ Ooyo
mmm. Dhi kar tiegruok mapiny/ mamalo kod mbalariany manyien ◻ Kamano	◻ Ooyo
nnn. Tieko tiegruok mapiny/,mamalo/mbalariany sikul/ mbalariany ◻ Kamano ◻ Ooyo
ooo. Wilo yoo mar yudo pesa kuom jonyuol	◻ Kamano	◻ Ooyo
ppp. Wito osiepni machiegni kodi (kuom AYA)	◻ Kamano	◻ Ooyo
qqq. Wito achiel kuom jo odu	◻ Kamano	◻ Ooyo

Ka nitie liel e odu, nga notho?

◻ Minwa

◻ wuonwa

◻ Owadwa/nyaminwa

Owadwa/nyaminwa ma bathe

◻ Dawa/ kwara

◻ Jaoda/ Osiepa

◻ Ngato machielo mar anyuola

6. Sama an gi chandruok matek kata gimoro mathago chunya matek, an kod osiepe kata jo odwa ma anyalo dhi irgi mondo okonya. Bende inyalo wacho ni weche gi:

◻ Adieri makende	◻ Nyalo bedo adieri	◻ Nyalobedo miriambo	◻ Miriambo makende

| *I. Inyalo pimo weche gi nango e ratin?* | | |
| --- | --- | --- |
| Weche gi penjo kuom ngimani kod timbeni. E ratin mar 1 nyaka 10 (1 bedo marach moloyo kae 10 maber kabisa. Ere kaka isebedo ga kiwinjo manie diere, en dweche adek mokalo?) | | |
| Kaka iwinjo | The Very Worst | The Very Best |
| Kaka ineno gi? | 1 2 3 4 5 6 7 8 9 10 | |
| E ringre ni? | 1 2 3 4 5 6 7 8 9 10 | |
| Epachi | 1 2 3 4 5 6 7 8 9 10 | |
| Kuom gik ma itimo pile pile kaka tiegruok, tich kod tije ot. | 1 2 3 4 5 6 7 8 9 10 | |

*II. Chal mar yudo tuo*

Adwa penji penjo gi kuom pachi e yor ngima. Chiw duoko kama owinjore

1. Ne in kod higni adi mane onyisi ni ituo?

____Higa, ____Dwe	□ okanyal paro/okangeyo

2.Nga nonyisi wach tuo ni?

□ Ja ritna mokuongo	□ jo odwa mamoko

□ Achiel kuom laktar, nga?___________□ Mamoko: Nga:___________

3. Nga gini mane obedo sama niwuoyo kodi e wach tuoni?

□ Ja ritna mokuongo	□ Jo odwa mamoko, nga:___________

□ Jo kar thieth: Nga___________	□ Jomamoko: Nga:___________

4.Iwinjo nade kuom gik mane uwacho kuom tuoni??

□ Nawinjo ka wuoyo no nokonya.

□ Nawinjo ka ayudo kony mangeny kowuok kuom jo kar thieth.

□ Nawinjo gima nolerna

□ Nokawinjo gikmane olerna.

□ Nokawinjo kar thieth nokonya moromo

□ Nawinjo ka wuoyo no netek

□ Mamoko

5. Mane onyisi ni ituo mokuong, Ne iwinjo nade?:

| m. Nikuyo? | □ Kamano □ Ooyo |
| --- | --- |
| n. Ne in kare/ ninom? | □ Kamano □ Ooyo |
| o. Nibuok? | □ Kamano □ Ooyo |
| p. Niyweyo? | □ Kamano □ Ooyo |
| q. Ne iyi owang? | □ Kamano □ Ooyo |
| r. Ne piny chalre ni? | □ Kamano □ Ooyo |

7.

Sani koro iwinjo nade kuom ngimani kaka idhi mbele gi yien?

| m. Ikuyo? | □ Kamano □ Ooyo |
| --- | --- |
| n. Ne in kare/ amor? | □ Kamano □ Ooyo |
| o. Nibuok? | □ Kamano □ Ooyo |
| p. Ne an maber | □ Kamano □ Ooyo |
| q. Ne iyi owang? | □ Kamano □ Ooyo |
| r. Onge pogruok | □ Kamano □ Ooyo |

8. Ango mosebedoni maber chakre ninge chal mari?

| m. Sani ayie kuom wach yedhe kendo amuonyo gi. | □ Kamano □ Ooyo |
| --- | --- |
| n. Ayangora ne jo odwa kata ne osiepe na. | □ Kamano □ Ooyo |
| o. Angeyo gimomiyo atuo. | □ Kamano □ Ooyo |
| p. Angeyo tija mar geno gik ami jomoko kute. | □ Kamano □ Ooyo |
| q. Wacho adieri osetego hera mekinda gi jo odwa. | □ Kamano □ Ooyo |
| r. Mamoko | □ Kamano □ Ooyo  wachi_____________________________________ |

9. Be intie gi achiel kuom chandruok gi nikech ingeyo gima chami?

| q. Nanyiso osiepa, ja gwenga kata jok mane okonengo anyisi. | □ Kamano □ Adagi |
| --- | --- |
| r. Nanyiso jo odwa mane okonengo anyisi. | □ Kamano □ Adagi |
| s. An gi lit kod paro mangeny | □ Kamano □ Adagi |
| t. Aluoro tho | □ Kamano □ Adagi |
| u. Timbe na richo kabisa | □ Kamano □ Adagi |
| v. Okamiyo yath kata thieth thuolo mowinjore. | □ Kamano □ Adagi |
| w. Ji ok dwara kendo ojara | □ Kamano □ Adagi |
| x. Mamoko? | □ Kamano □ Adagi wachi:_____________ |

| III. Gengo kata gima ngato ohero |  |
| --- | --- |
| Kiparo kama idhi gak awe thieth kod duoko teko mag kute ayaki piny, ando miparoni ma thagi ga kabisa. Kiyie to wach abich machandi bendo mokuongo obed athagi moloyo. | 3. 2. 3. 4. 5.   Paro maduong. Paro 5^th^ madongo |
| Kiparo jok ma nie hiki ma bended hi kao yath maduoko teko mar kute mag ayaki piny, iparo ni ango ma nyalo bedo chandruok gi? Kiyie to wach abich mathago gi kendo mokuongo obed mathagogi moloyo. | 3. 2. 3. 4. 5.   Paro maduong. Paro 5^th^ madongo |
| 16. **Yoo chomo kar thieth**     a. Yoo mar chopo kar thieth tek.     b. Omenda mar chopo kar thieth     c. Naonge thuolo moro mar chopo kar thieth.     d. Yore mamoo, (wachi ______________) 17. **Tich, tiegruok kod jo ot. Work,**     a. Mona tich     b. Mona tiegruok gi wach dhi medo yath.     c. Mona tiegruok gi wach dhi e thieth.     d. AYA/ Jaritna odhi e liel     e. AYA/ ja ritna ne nigi tije mamoko     f. Jaoda ne ok dwar ni adhi e od thieth     g. Keny, nyuol, wilo jarit (wachi ______________)     h. Jo ot mamoko/tije mamoko, wachi 18. **Thieth** 19. Felt too sick to come to clinic     a. Jaritna netuo matek nokonyal chopo kar thieth.     b. Yedhe go ne inya     c. Yath ne ngeny     d. Naol gi muonyo yath     e. Nayach/ nanyuol.     f. Weche mamoko mag thieth, wachgi     g. ___ Osuptal 🡪 Tarik morwake e ward: ______/________/_____DD /MM /YYYY     h. Nying osuptal:     i. Gima omiyo norwaki e osuptal: 20. **Chal mar kar thieth**     a. Yath nosumo kar thieth     b. Dar kiwuok kar thieth mar rawero kidhi e mar joma dongo     c. Jo tich ma kar thieth nok beyo     d. Thieth nok ber     e. Jo taa ji nidwaro mangeny     f. Kar rito laktar nokber     g. Kar thieth nopong     h. Chuno ni nyaka arit aminga     i. Nokadwar dhi kar puonjruok mar kae yien 21. **Psycho-social**     a. Nawinjo maber, nokaneno tiende dhi kar thieth     b. Dhi kar thieth biro jo gweng ngeyo ni an kod kute mag ayaki     c. Naluok ni jo kar thieth biro dhaona gimomiyo alewo ne thieth.     d. Nadhi ir ngato mane temo theidha e yor lemo     e. Nikech aneno ajuoga     f. Naonge geno /paro mangeny     g. Madho kongo     h. Wiya nowil gi wach thieth     i. Jo odwa gi osiepe machiegni nowacho ni nyathi owe dhi kar thieth. 22. *O kangeyo* 23. *Adagi* | |
|  |  |
| Ka itemo keto pachi modich gi wach thieth mar ayaki kaka masira, dhi e od thieth, muonyo yien pile, duoko teko mar kute mag ayaki piny) En yor kony mane ma ineno ni nyalo konyi? Wach anena adek to ichak gi mokuongo midwaro moloyo gi tee | Kony mar jo ongwe yamo  Kony mar jogo ma mongere  Kony e yor omenda  Oro barua e ongwe yamo  Mamoko (wachi) ___________________  Onge yoo oro machielo |
| Ka nitie mbasni matemo keto pache modich gi thieth mar kute mag ayaki, kony mane ma nyalo bedo ne maber? Wach kony adek kichako gi mokuongo obedo gombone maduong. | Kony mar jo ongwe yamo  Ony mar mbese mosiepe  Kony matin mar omenda  Paro ne gi e ongwe yamo  Mamoko (Wachi) ___________________  No Onde kony moro kendo |

*IV. Psychological Well-Being*

Weche gi wuoyo kuom timbe rawere kod jogo madongo. Sama isomo weche gi yier ni wch mane ma lero timbeni e dweche adek mokalo.

Par timbeni e dweche adek mokalo.....

57. Pacha kata gombona tinde olokore

□ Ngenyne adiera	□ Samoro adiera	□ Ok en adiera

58. Aneno ni onge ngat ma ohera

□ Ngenyne adiera	□ Samoro adiera	□ Ok adiera

59. Samoro an malo, aluor kata an gi paro

□ Ngeny adieri	□ Samoro adieri	□ Ok en adieri

60. Ariambo kendo okawach adiera

□ Ngenyne adieri	□ Samoro adieri	□ Ok en adieri

61. An gi luoro ahinya kata ilo

□ Ngenyne adieri	□ Samoro adieri	□ Ok en adieri

62. Amino wach ahinya

□ Ngenyne adieri	□ Samoro adieri	□ Ok en adieri

63. An git chandruok mar keto pacha e gimoro, okanyal keto pacha kuom saa mangeny.

□ Ngenyne adieri	□ Samoro adieri	□ Ok en adieri

64. Angeny ahinya kendo pacha lal

□ Ngenyne adieri	□ Samoro adieri	□ Ok en adieri

65. An ja njre, awuor ne jomamoko

□ Ngenyne adieri	□ Samoro adieri	□ Ok en adieri

66. Aonge luoro dala

□ Ngenyne adieri	□ Samoro adieri	□ Ok en adiera

67. I am disobedient at school Aonge luoro kar tiegruok

□ Ngenyne adiera	□ Samoro adiera	□ Ok en adiera

68. Aonge muoro ka asetimora marach

□ Ongenyne adiera	□ Samoro adiera	□ Ok en adiera

69. An gi chandruok bedo gi ji kanyakla

□ Ngenyne adiera	□ Samoro adiera	□ Ok en adiera

70. An gi chandruok bedo gi jopuonj kanyakla

□ Ngenyne adiera	□ Samoro adiera	□ Ok en adiera

71. An gi ichwang kaka, atimo gimoro ka pok aparo

□ Ngenyne adiera	□ Samoro adiera	□ Ok en adiera

72. I feel worthless or inferior Awiinjo ka aonge nyalo kendo ayomyom.

□ Ngenyne adiera	□ Samoro adiera	□ Ok adiera

73. Jii mangeny okohera

□ Ngenyne adiera	□ Samoro adiera	□ Ok en adiera

74. An gi chandruok golo pacha e gikmoko.

□ Ngenyne adiera	□ Samoro adiera	□ Ok en adiera

75. Bedo mos tama, kendo tekona ngeny okanyal bedo piny

□ Ngenyne adiera	□ Samoro adiera	□ Ok en adiera

76. Wiya tek, atheko kendo chunya rach

□ Ngenyne adiera	□ Samoro adiera	□ Ok en adiera

77. An gi ich wach matek kendo tama ga duoko piny

□ Ngenyne adiera	□ Samoro adiera	□ Ok en adiera

78. Okamor, akuyo kendo paro thaga

□ Ngenyne adiera	□ Samoro adiera	□ Ok en adiera

79. Abedo ga kenda okahera kama jii nitie

□ Ngenyne adiera	□ Samoro adiera	□ Ok en adiera

80. Awinjo ka jomoko dwa oma

□ Ngenyne adiera	□ Samoro adiera	□ Ok en adiera

81. Ahero bedo gi nyithindo ma ohero kaelo dhao

□ Ngenyne adiera	□ Samoro adiera	□ Ok en adiera

82. An ja malingling kendo wechena aling godo

□ Ngenyne adiera	□ Samoro adiera	□ Ok en adiera

83. Aparo mangeny

□ Ngenyne adiera	□ Samoro adiera	□ Ok en adiera

84. Ageno kuom ji mamoko

□ Ngenyne adiera	□ Samoro adiera	□ Ok en adiera

PATIENT HEALTH QUESTIONNAIRE-9 ( P H Q - 9 )

Luor duoko mowinjore.

| Penjo | Duoko | | | |
| --- | --- | --- | --- | --- |
| Kuom jumbe 2, en mang’eny marom nade ma moro kuom chandruok moluwogi osechandi? (Ti gi “✔” mondo inyis duoko mari) | Onge kata matin | Odiechienge mang’eny | Mang’eny ne nus odiechienge | Machiegni gi odiechieng’ kodiechieng’ |
| 1. Bedo gi chuny mool kata koso mor etimo gik moko | 0 | 1 | 2 | 3 |
| 2. Winjo ka olo, paro mang’eny, kata bedo maonge geno | 0 | 1 | 2 | 3 |
|  | If #1 and #2 are both 0, go to end.  Otherwise proceed to #3 | | | |
| 3. In gi chandruok nindo kawi kata nindo, kata nindo mang’eny ahinya | 0 | 1 | 2 | 3 |
| 4. Winjo olo kata bedo gi teko matin | 0 | 1 | 2 | 3 |
| 5. Dhok marach kata chiemo mang’eny | 0 | 1 | 2 | 3 |
| 6. Winjo marach gi in iwuon — kata ni in ng’at ma ok nyal kata ni ok itimo gima onego itim ne in kata ne joodi | 0 | 1 | 2 | 3 |
| 7. Chandruok keto pachi kuom gik moko, kaka somo gaset kata neno wang’ jowi | 0 | 1 | 2 | 3 |
| 8. Sudo kata wuoyo mos ma jomoko nyalo bedo ne nyalo fwenyo? Kata ataro — chapni kata koso bedo mos ma isebedo ka iwuodho ka iluorori mathoth moloyo mapile | 0 | 1 | 2 | 3 |
| 9. Paro ni inyalo bedo maber moloyo ka itho kata mar hinyri iwuon eyo moro | 0 | 1 | 2 | 3 |

Total score:

Score 10 to 19 = refer to clinic for counseling

Score 20 or higher = refer to clinic for psychiatrist referral

Adverse Childhood Experiences International Questionnaire (ACE-IQ)

|  | DEMOGRAPHIC INFORMATION | |
| --- | --- | --- |
| 1 | KENY | |
| 1.2  [M2] | Bende osekendi/ isekendo? Nokendi/ nikendi mokuongo ka in kod higni adi? | Higa [ ][ ] |
|  |  | Pok okenda/ pok akendo [Go to M5] |
|  |  | Adagi [Go to M5] |
| 1.3  [M3] | Sama nikendo/ nokendi mokuongo bende niyiero jaodi? | Kamano *(Go to M5)* |
|  |  | Ooyo |
|  |  | Okangeyo/ ok an gi adiera |
|  |  | Adagi |
| 1.4  [M4] | Sama ne ikendo/ okendi ka ne ok iyiero jaodi bende ne ichiwo yieni kuom yiero no? | Kamano |
|  |  | Ooyo |
|  |  | Adagi |
| 1.5  [M5] | Ka in miyo kata wuoro, ne in kod higni adi mane iyudo nyathi mokuongo? | Higa[ ][ ] |
|  |  | Not applicable |
|  |  | Adagi |

| 2 | WINJRUOK GI JANYUOL/ JARIT | | |
| --- | --- | --- | --- |
|  | *PENJ TEE*  Ndalo mane in nyathi (higni matin ne 18 e ngimani…) | | |
| 2.1  [P1] | Bende jonyuolni ongeyo chandruokni kata paro magi? | Ndalo mathoth | |
|  |  | Kinde machwok | |
|  |  | Samoro | |
|  |  | Kadicihiel | |
|  |  | Podi | |
|  |  | Adagi | |
| 2.2  [P2] | Bende jonyuol ni ongeyo gima itimo sani ma in gi thuolo sama ok in e skul or tich? | Ndalo mathoth | |
|  |  | Kinde machwok | |
|  |  | Samoro | |
|  |  | Kadichiel | |
|  |  | Podi | |
|  |  | Adagi | |
| 3 |  | | |
| 3.1  [P3] | En di di ma janyuolni ok miyi chiemo moromo ka e sama nonengo obedni negisetimo kamano? | Ndalo mathoth | |
|  |  | Kinde machwok | |
|  |  | Dichiel | |
|  |  | Podi | |
|  |  | Adagi | |
| 3.2  [P4] | Be jonyuolni ne omer kata negitiyo gi yedhe mamero ji mane omono gi riti? | Ndalo mathoth | |
|  |  | Kinde machwok | |
|  |  | Dichiel | |
|  |  | Podi | |
|  |  | Adagi | |
| 3.3  [P5] | En ndalo adi ma jonyuolni nokoori e kar tiegruok to tiegruok ne dhi mbele? | Ndalo mathoth | |
|  |  | Kinde machwok | |
|  |  | Dichiel | |
|  |  | Podi | |
|  |  | Adagi | |
| 4 | DAK MAR ANYUOLA | | |
|  | *PENJ TEE*  Ndalo mane in nyathi (tin ni nyaka ichop higni 18 e ngima ni) | | |
| 4.1  [F1] | Bende i dak gi ngato e ot mani gi madho kongo, kata mane tiyo gi yath e yoo mok ma ok owinjore? | | kamano |
|  |  |  | Ooyo |
|  |  |  | Adagi |
| 4.2  [F2] | Bende ne idak gi jatich manigi paro mangeny, kata in, wiye ringo neko kata mani paro mar deyore? | | Kamano |
|  |  |  | Ooyo |
|  |  |  | Adagi |
| 4.3  [F3] | Bende isedak/ be idak gi ngato e ot mane oseor e e jela kata ? | | Kamano |
|  |  |  | Ooyo |
|  |  |  | Adagi |
| 4.4  [F4] | Be jonyuolni nopogore kata nowere? | | Kamano |
|  |  |  | Ooyo |
|  |  |  | Not applicable |
|  |  |  | Adagi |
| 4.5  [F5] | Be mama mamani, babani kata ja rit mari notho? | | Kamano |
|  |  |  | Ooyo |
|  |  |  | okangeyo / okan gi adiera |
|  |  |  | Adagi |
| *PENJ TEE*  Magi penjo ma samoro nisewinjo kata niseneno e dalau. magi gikma nyalobedo ni osetimore ne ngato machielo midak godo okonyiso ni nyaka bed in. | | | |

| Ndalo mane in nyathi (tin ni ka pok ichopo higni 18) | | | |
| --- | --- | --- | --- |
| 4.6  [F6] | Be nineno/ ineno kata winjo jonyuol kata ngatma udak godo kagoyo koko, okok, hangore, oyany kata ochai? | | Ndalo mathoth |
|  |  |  | Kinde mathin |
|  |  |  | Dichiel |
|  |  |  | Podi |
|  |  |  | adagi |
| 4.7  [F7] | Be nineno/ ineno kata winjo janyuol kata ngat mudak godo e ot kopadi, ogwe, ogo adhong kata ogo? | | Ndalo mathoth |
|  |  |  | Kinde machwok |
|  |  |  | Dichiel |
|  |  |  | Podi |
|  |  |  | Adagi |
| 4.8  [F8] | Be nineno/ineno kata iwinjo janyuol kata ngat mudak godo e ot mogo kata ongadi, gima machalo kende (kata olando) chupa, pala,nyaunyo | | Ndalo mathoth |
|  |  |  | Kinde machwok |
|  |  |  | Dichiel |
|  |  |  | Podi |
|  |  |  | adagi |
| *PENJ JOK MA OSECHOPO HIGNI 18 KADHI MBELE KENDE*  Penjo mabiro gi gin kuom gik ma nyalo bedo ni nosetimore kuomi.  Ndalo mane in nyathi (chakre tin ni nyaka ichopo higni 18…) | | | |
| 5 |  | | |
| 5.1  [A1] | Bende janyuol, jarit kata ngato machielo maun godo e ot ogoyo koko, ogoyo nduru kata ohangore ne in kata ochai? | Ndalo mathoth | |
|  |  | Kinde machwok | |
|  |  | Dichiel | |
|  |  | Podi | |
|  |  | Adagi | |
| 5.2  [A2] | Bende jonyuol, jarit kata ngato midak godo osebuogi kata osejwangi kata oriembi e ot? | Ndalo mathoth | |
|  |  | Kinde machuok | |
|  |  | Dichiel | |
|  |  | Podi | |
|  |  | Adagi | |
| 5.3  [A3] | Bende janyuol, jarit kata ngato midak godo osepadi, gwei, goi adhong kata goi? | Ndalo mathoth | |
|  |  | Kinde machuok | |
|  |  | Dichiel | |
|  |  | Podi | |
|  |  | Adagi | |
| 5.4  [A4] | Bende janyuol jarit kata ngato midak godo osegoi kata ngadi gi kede kata olando, chupa, pala nyaunyo? | Ndalo mathoth | |
|  |  | kinde machuok | |
|  |  | Dichiel | |
|  |  | Podi | |
|  |  | Adagi | |

| 6 | LWENY MAG RIKA | | | |
| --- | --- | --- | --- | --- |
| *PENJ TEE:*  Penjo mabiro gi gin mag anjawo ndalo thin ni. Anjawo en ka ngato matin ni kata jogo matindoni wacho gimarac, gima ok mori ne ngato matin ni. Kendo anjawo en ka ngato matin ni sandi mangeny e yoo ma ok mori kata ka ngato matin ni oweyo owuok gi gik ma ok ochomo yoo. Ok en anjao ka ji ariyo matindo manigi teko maromre lwerore kata dhao kata ka temo njao nyawadgi e yoo mar osiep gi tugo.  Ndalo mane in nyathi (Ka ne pok ichopo hogni 18 e ngimani)… | | | | |
| 6.1  [V1] | Ninjawi ga marom nade? | | | Ndalo mathoth |
|  |  |  |  | Kinde machwok |
|  |  |  |  | Dichiel |
|  |  |  |  | Podi *(Go to Q.V3)* |
|  |  |  |  | Adagi |
| 6.2  [V2] | Nisandi ga nango thothne? | | | Nigoya, igweya, idhira, irunda kata ilorona dhot. |
|  |  |  |  | Nogo koda ngero nikech kaka achalo, gi piny ma awuoke kod chal mar denda. |
|  |  |  |  | Nogo koda ngero nikech kanisa ma aleme. |
|  |  |  |  | Nogo koda ngero gi weche mag angera mar terruok, wuoyo kod wuoyo ma ok oti gi dhok. |
|  |  |  |  | Noweya e tich mane itimo kagingeyo kata nigigole kabisa |
|  |  |  |  | Nogo koda ngero nikech kaka denda kata wanga chalo |
|  |  |  |  | Ne ojara e yore mopogore |
|  |  |  |  | Adagi |
| *PENJ TEE*  Penjo mabiro gi mag dhao amena. Dhap amen timore sama jii ariyo matindo manigi teko maromreoyiero ni goyo nyawadgi. | | | | |
| Ndalo mande in nyathi (ka pok ichopo higni 18 e ngimani ) . . . | | | | |
| 6.3  [V3] | Di di mane udhao dhap amen? | | | Ndalo mathoth |
|  |  |  |  | Kinde manok |
|  |  |  |  | Dichiel |
|  |  |  |  | Podi |
|  |  |  |  | Adagi |
| 7 | | NENO TULO | | |
|  | | *PENJ TEE*  Penjo gi adwa ngeyo ka nineno kata nokineno gik mane otimore e ndalo mane in nyathi. Gik mane otimore gi ne gin kuom dhao, lweny, tula siasa kod kod dhao mar dhok, neko, lal gi ji, sendo kod loso dhao ji jo anjore.  Ndalo mane in nyathi (chakre tin ni nyaka higni 18 e ngima ni…) | | |
| 7.1  [V4] | | Bende nosechuni mondo idhi idag kamoro nikech gikmane otimore go? | Ndalo mangeny | |
|  |  |  | Kinde manok | |
|  |  |  | Dichiel | |
|  |  |  | Podi | |
|  |  |  | Adagi | |
| 7.2  [V5] | | Did/do you experience the deliberate destruction of your home due to any of these events? Bende noseriembi kata ketho dalani nikech gik mane otimore go? | Ndalo mangeny | |
|  |  |  | Kinde manok | |
|  |  |  | Dichiel | |
|  |  |  | Podi | |
|  |  |  | Adadgi | |
| 7.3  [V6] | | Bende osegoi gi obila, jolweny kata jo njore? | Ndalo mangeny | |
|  |  |  | kinde manok | |
|  |  |  | Dichiel | |
|  |  |  | Podi | |
|  |  |  | Adagi | |
| 7.4  [V7] | | Nitie ja odu kata osiepi mane onegi, ogo gi obila, jolweny kata jo njore? | Ndalo mathoth | |
|  |  |  | Ndalo manok | |
|  |  |  | Dichiel | |
|  |  |  | Podi | |
|  |  |  | Adagi | |

GAD-2

| Chiegni  Onge Odiechien _Mang’eny_ odiechieng **Kuom jumbe 2 mokalo, en mang’eny marom nade** ^kata ge^ ne nus ^ka^ **chandruoge moluwogi osechandi?** matin mang’eny _mar_ odiechieng  odiechien g  *(Ti gi “✔” mondo onyis duoko ni)* | | | | |
| --- | --- | --- | --- | --- |
| 1.  Winjo ka chunyi oluor, luoro, kata chunyi dang’ni | 0 | 1 | 2 | 3 |
| 2.Bedo ni ok inyal weyo kata tayo parruok | 0 | 1 | 2 | 3 |

Generalized Anxiety Disorder 7-item (GAD-7) scale

| Kuom jumbe 2 mokalo, en mang’eny marom  nade ma chandruoge moluwogi osechandi? Mang’eny Machiegni Onge gi Odiechien ne nus mar Odiechieng   1. Winjo ka chunyi oluor, luoro, kata chunyi _adier kata ge odiechieng ko_   ^dang’ni^ matin mang’eny e odiiechieng   2. Koso weyo kata tayo parruok ^0 1 2 3^ 3. Paro mang’eny kuom gik mopogore opogore ^0 1 2 3^ 4. In gi chandruok mar kawo yueyo ^0 1 2 3^ 5. Bedo ka idang’ni matek bedo mos ^0 1 2 3^ 6. Iyi wang’ piyo kata chwanyruok ^0 1 2 3^ 7. Winjo kiluor ka gima gimoro marach 0 1 2 3   nyalo timre  *Add the score for each column* ^0 1 2 3^ | |
| --- | --- |
| Total Score *(add your column scores)* = | + + + |
|  |  |

Ka ne igwelo chandruok moro amora, en matek marom nade ma magi ne omiyo itimo tijegi, rito makare gik moko dala, kata miyo iwinjri gi jomoko maber?

Ok tek kata matin	Tek ediere kanyo	Tek ahinya

Tek mang’eny ahinya

Source: Spitzer RL, Kroenke K, Williams JBW, Lowe B. A brief measure for assessing generalized anxiety disorder. *Arch Inern Med.* 2006;166:1092-1097.

**A4A – Alcohol and Drug Use Questionnaire**

*Your answers will not be told to anyone, even your parents.Daher mar penji penjo moko kuom kong’o gi yedhe kata gik mameroji ma dipo ni ne itiyogo ma ne ok omiyi gi laktar.*

**Part 1: General Alcohol Screening**

1. Be isega madho math man kod kong’o?
   - Ee	◻	Ooyo	◻	Akia	◻ Odagi

**Part 2: AUDIT-10 Questionnaire**

Yie iyier duoko en adier ne in.

1. En mang’eny marom nade ma imadhe math man kod kong’o?

- Podi	◻ Dwe ka dwe kata matin
- Diriyo nyaka ding’wen edwe
- Diriyo nyaka didek ejuma
- Ding’wen kata nyading’eny ejuma

2. Gin kwan marom nade mag math man kod kong’o ma imadho e odiechieng’ mapile ekinde ma imedhe?

- 1 kata 2	◻	3 kata 4	◻	5 kata 6	◻	7 kata 9	◻ 10 kata mang’eny

3. En mang’eny marom nade ma imadhe math auchiel kata mang’eny enyasi achiel?

- Podi	◻ Matin ne dwe ka dwe
- Dwe ka dwe	◻ Juma ka juma
- Odiechieng ko odiechieng kata machiegni gi Odiechieng ka odiechieng

4. En mang’eny marom nade ehiga mokalo ma iseyude ni ne ok inyal chungo metho tek ni isechako?

- ^Podi^	◻ Matin ne dwe ka dwe
- Dwe ka dwe	◻ Juma ka juma
- Odiechieng ko odiechieng kata machiegni gi Odiechieng ka odiechieng

5. En mang’eny marom nade ehiga mokalo ma iserem timo gima onego itim pile nikech medho?

- ^Podi^	◻ Matin ne dwe ka dwe
- Dwe ka dwe	◻ Juma ka juma
- Odiechieng ko odiechieng kata machiegni gi Odiechieng ka odiechieng

6. En mang’eny marom nade ehiga mokalo ma isedware math mokuongo gokinyi mar konyi idhi nyime bang’ kinde mar metho mapek?

- ^Podi^	◻ Matin ne dwe ka dwe
- Dwe ka dwe	◻ Juma ka juma
- Odiechieng ko odiechieng kata machiegni gi Odiechieng kodiechieng

7. En mang’eny marom nade ehiga mokalo ma isebede gi winjo mar chich ni itimo gi marach kata chuny mar kwa ng’uono bang’ metho?
   - Podi	◻	Matin ne dwe ka dwe	◻	Dwe ka dwe◻	Juma ka juma	◻	Odiechieng kodiechieng kata machiegni gi odiechieng kodiechieng

*Version September 6 2018*

8. En mang’eny marom nade ehiga mokalo ma isebede ka ok inyal paro gima ne otimore otieno motelo nikech ne imetho?

- ^Podi^	◻ Matin ne dwe ka dwe
- Dwe ka dwe	◻ Juma ka juma
- Odiechieng kodiechieng kata machiegni gi Odiechieng ka odiechieng

9. Be in kata ng’at machielo osehinyore kowuok emethoni?
   - Ooyo	◻	Ee, to ok ehiga mokalo	◻ Ee, ehiga mokalo
10. Bi wat kata osiep, kata laktar kata jatich mar thieth mamoko, osewuoye kata sumi paro ni iduok piny methoni?

- Ooyo	◻	Ee, to ok ehiga mokalo	◻ Ee, ehiga mokalo

Procedure for Scoring AUDIT

Questions 1-8 are scored 0, 1, 2, 3, or 4. Questions 9 and 10 are scored 0, 2, or 4 only. The response coding is as follows:

|  | 0 | 1 | 2 | 3 | 4 |
| --- | --- | --- | --- | --- | --- |
| Question 1 | Never | Monthly  or less | Two to  four times  per month | Two to  three times  per week | Four or more  times per week |
| Question 2 | 1 or 2 | 3 or 4 | 5 or 6 | 7 to 9 | 10 or more |
| Questions 3-8 | Never | Less than  Monthly | Monthly | Weekly | Daily or  almost daily |
| Questions 9-10 | No |  | Yes, but  not in the  last year |  | Yes, during  the last year |

The minimum score (for non-drinkers) is 0 and the maximum possible score is 40.

A score of 8 or more indicates a strong likelihood of hazardous or harmful alcohol consumption.

**Part 3: Drug Use (adapted from KIAS)**

1. Be isegatemo yedhe kaka Mira, Nyasore, Gam, Mandrax, Koken, Eroin gi mamoko?
   - Ee	◻	Ooyo	◻	Akia	◻ Odagi

|  | **Be isetemo moluwogi?** | | **IKa ee, ne en karang’o mogik mane itiye**  **kode?** |
| --- | --- | --- | --- |
| 2. | Mira | - Ee - Ooyo - Akia - Odagi | - Dweni - Dweche 2-6 mokalo - Dweche 6-12 mokalo - Mang’eny ne higa 1 mokalo - Akia |

*Version September 6 2018*

|  |  |  | □ Odagi |
| --- | --- | --- | --- |
| 3. | Gam / Petrol /  Gundi | - Ee - Ooyo - Akia - Odagi | - Dweni - Dweche 2-6 mokalo - Dweche 6-12 mokalo - Mang’eny ne higa 1 mokalo - Akia - Odagi |
| 4. | Nyasore / Njaga | - Ee - Ooyo - Akia - Odagi | - Dweni - Dweche 2-6 mokalo - Dweche 6-12 mokalo - Mang’eny ne higa 1 mokalo - Akia - Odagi |
| 5. | Yath ma mero ji mar Mandrax | - Ee - Ooyo - Akia - Odagi | - Dweni - Dweche 2-6 mokalo - Dweche 6-12 mokalo - Mang’eny ne higa 1 mokalo - Akia - Odagi |
| 6. | Yath ma mero ji mar Koken | - Ee - Ooyo - Akia - Odagi | - Dweni - Dweche 2-6 mokalo - Dweche 6-12 mokalo - Mang’eny ne higa 1 mokalo - Akia - Odagi |
| 7. | Yath ma meroji mar Eroin | - Ee - Ooyo - Akia - Odagi | - Dweni - Dweche 2-6 mokalo - Dweche 6-12 mokalo - Mang’eny ne higa 1 mokalo - Akia - Odagi |
| 8. | Mamoko, ler  _  __ | - Ee - Ooyo - Akia - Odagi | - Dweni - Dweche 2-6 mokalo - Dweche 6-12 mokalo - Mang’eny ne higa 1 mokalo - Akia - Odagi |

**A4A Intervention Satisfaction and Experience Survey**

Daher mar penji ewi kony ma dipo ni ne iyudo eklinik ka kaka gima bende ne itimo enonroni. Duokoni madier biro konyowa keto kony maber mondo okony rowere gi jomadongo matindo man e rit gi thieth.

**Hocho mar gik matiyo gi stima**

| 1. Kuom dweche 12 mokalo, ka ne in enonroni, be ne iyudo ote machuok mar sim mochan gi sir mar Jasir (bas) kokale esimb ong’we yamo/ chenro mar sim(App)? | | | | | | Ee –> dhi nyime nyaka 1a. Ooyo–> kal nyaka 2.  Akia  Odagi duoko | | |
| --- | --- | --- | --- | --- | --- | --- | --- | --- |
| Daher ni inyisa kaka lemo moko mag nonro no oromi ki itiyo gi ratil moluwoni: | | | | | | | | |
| a.  Ere kaka magi oseromi: | *Som matek* | | | | | | *Kik isom matek* | |
|  | Oromo ahinya | Oromo | Ediere | Ok oromo | Ok oromo ahinya | | Akia | Odagi duoko |
| b.  Yot mar tudruok e simb ong’we yamo/Ap? |  |  |  |  |  | |  |  |
| c.  Yoo maling’ling’ mar tudruok mar ote machuok mag sim ma ochan? |  |  |  |  |  | |  |  |
| d.  Yoo maling’ling’ mar tudruok mar jahocho mar mbese? |  |  |  |  |  | |  |  |
| e.  Kaka ote machuok mar sim mochan ne mar osiep? |  |  |  |  |  | |  |  |
| f. |  |  |  |  |  | |  |  |

| Yor osiep mar tudruok gi jahocho mar mbese? |  |  |  |  |  |  |  |
| --- | --- | --- | --- | --- | --- | --- | --- |
| g. |  |  |  |  |  |  |  |
| Bedo ni ne inyalo  dhi ir jahocho mayot ka ionge gi luoro? |  |  |  |  |  |  |  |
| h. |  |  |  |  |  |  |  |
| Bedo ni jahocho  ne jaratiro? |  |  |  |  |  |  |  |

A4A Intervention Experience Survey 17 October 2019

| i. |  |  |  |  |  |  |  |
| --- | --- | --- | --- | --- | --- | --- | --- |
| Nyalo mar ote  machuok mar sim mochan konyi kuom muonyo yath? |  |  |  |  |  |  |  |
| j. |  |  |  |  |  |  |  |
| Kony mar jahocho  mar mbese mondo imuony yath? |  |  |  |  |  |  |  |
| k. |  |  |  |  |  |  |  |
| Kony mar ote  machuok mochan mar konyi ipar biro eklinik? |  |  |  |  |  |  |  |
| l. |  |  |  |  |  |  |  |
| Kony mar jahocho mondo okonyi ipar biro eklinik? |  |  |  |  |  |  |  |
| m. |  |  |  |  |  |  |  |
| Nyalo mari mar yudo weche  mang’eny kata wuoyo gi ng’ato eklinik kuom ngimani? |  |  |  |  |  |  |  |
| n. |  |  |  |  |  |  |  |
| Duto tee, ote  machuok mar sim mochan gi sir mar jahocho mane iyudo oromi marom nade? |  |  |  |  |  |  |  |
| o. |  |  |  |  |  |  |  |
| Duto tee, kony mar ote machuok mar sim gi jahocho  engimani gi gik madich gi ngimani |  |  |  |  |  |  |  |

| maber no oromi marom nade? |  |  |  |  |  |  |  |
| --- | --- | --- | --- | --- | --- | --- | --- |
|  | Daher mang’eny  ahinya ahinya | Daher mang’eny  ahinya | Daher ediere | Daher matin | Ok daher kata matin | Akia | Odagi duoko |
| p. |  |  |  |  |  |  |  |
| Be ibiro hero mar dhi nyime gi yudo sir mar gik teko mar stima (ote machuok mar sim mochan gi sir kokalo esim/App ekinde mabiro, ka dipo ni nyalore? |  |  |  |  |  |  |  |

A4A Intervention Experience Survey 17 October 2019

| q. | Oro ote machuok mar sim | mapile |  |  |  |  |  |
| --- | --- | --- | --- | --- | --- | --- | --- |
| En App mane mar sim mane itiyogo duto tee etudruok gi  jahocho? | What app |  |  |  |  |  |  |
|  | FB messenger | |  |  |  |  |  |
|  | Snapchat |  |  |  |  |  |  |
|  | Mamoko ( ) | | |  |  |  |  |
|  | Akia |  | |  |  |  |  |
|  | Odagi duoko | |  |  |  |  |  |
| r. | Odiechieng kodiechieng | | |  |  |  |  |
| En mang’eny marom nade ma di iwach ni in kod jahocho ne  utudoru? | Nya di matin ejuma | |  |  |  |  |  |
|  | Juma ka juma | |  |  |  |  |  |
|  | Nya di matin edwe | |  |  |  |  |  |
|  | Dwe kadwe | |  |  |  |  |  |
|  | Matin ne dwe | |  |  |  |  |  |
|  | Akia |  |  |  |  |  |  |
|  | Odagi duoko | |  |  |  |  |  |
| s. | di 20 kata di mang’eny | | |  |  |  |  |
| En kwan mar nyadidi koriwo te ma diwach ni in kod jahocho ne  utudoru? | di 10-19 |  |  |  |  |  |  |
|  | di 5-9 |  |  |  |  |  |  |
|  | di 1-4 |  |  |  |  |  |  |
|  | Akia |  |  |  |  |  |  |
|  | Odagi duoko | |  |  |  |  |  |
| t. | Mang’eny ahinya | |  |  |  |  |  |
| Di iwach ni tudruok ni gi jahocho ne  mang’eny ahinya, mang’eny maber, kata ok ne mang’eny  moromo? | Mang’eny maber | |  |  |  |  |  |
|  | Ok mang’eny moromo | | |  |  |  |  |
|  | Akia |  |  |  |  |  |  |
|  | Odagi duoko | |  |  |  |  |  |
| u. | Mbekni kolale ote machuok | | |  |  |  |  |
| Be inyalo wacho ni ni thoth mbekni gi jahocho ne gin ote machuok, wuoyo, kata ma ote gi wuoyo  ne dwa rom? | Mbekni kokale wuoyo | | |  |  |  |  |
|  | Ote machuok gi wuoyo ne dwa rom | | |  |  |  |  |

|  | Akia |
| --- | --- |
|  | Odagi duoko |
| v. | Ne oroma ahinya |
| Yor tudruok mokuongo ne oromi  marom nade? | Ne oroma |
|  | Ne nie ediere |
|  | Ne ok oroma |
|  | Ne ok oroma ahinya |
|  | Akia |
|  | Odagi duoko |
| w. | Ber ahinya ahinya |
| Be inyalo wacho ni tudruok gi jahocho ne ber ahinya ahinya, ber ahinya, ber, ok ber ahinya kata onge ber kata matin? | Ber ahinya |
|  | Ber |

A4A Intervention Experience Survey 17 October 2019

|  | Ok ber ahinya |
| --- | --- |
|  | Ok ber kata matin |
|  | Akia |
|  | Odagi duoko |
| x. | Odiechieng’ kodiechieng |
| En mang’eny marom nade mane iyude Ote machuok  mar Sim? | Juma ka juma |
|  | Dwe kadwe |
|  | Matin ne dwe kadwe |
|  | Akia |
|  | Odagi duoko |
| y. | Mang’eny ahinya |
| Be di iwach ni ote machuok mag sim ne mang’eny  ahinya, mang’eny maber, kata ok  mang’eny moromo? | Mang’eny maber |
|  | Ok mang’eny moromo |
|  | Akia |
|  | Odagi duoko |
| z. | Ote machuok mochan mabiro kende |
| En mane ma osekonyo ahinya ngimani gi ber mari, ote machuok mochan mabiro kende, tudruok gi jahocho, ote machuok mochan gi jahocho, kata onge  ekind ariyogo? | Tudruok gi jahocho |
|  | Ote machuok mochan mabiro kende gi tudruok gi jahocho |
|  | Onge ekind ariyogo |
|  | Akia |
|  | Odagi duoko |
| aa. | Ee |
| Be ne ng’at ang’ata oneno ote machuok mar yor stima mowuok enonro mane ok idwa ni  gine? | Ooyo |
|  | Akia |

|  | Odagi duoko |
| --- | --- |
| bb. | Ee |
| Be ma no okelo  duoko marach? | Ooyo |
|  | Akia |
|  | Odagi duoko |
| *cc.* | £ Fwenyo ni an kod kute mag Ayaki |
| Ka ee, en kit duoko marach mage? | £ Penjo penjo mang’eny ahinya mane omiyo ok abedo thuolo |
| *Chuo* | £ Dhao gi jonyuol/jarit |
|  | £ Dhao gi jahera/jaot |
| Tee mowinjore | £ Dhao gi osiep/wat |
|  | £ Mamoko (ler: ) |
|  | £ Akia |
|  | £ Odagi duoko |

A4A Intervention Experience Survey 17 October 2019

| *Interviewer notes on*  *e-Navigation* |  |
| --- | --- |

**Hocho mar dhano wang’ gi wang’**

| 2. | Ee –> dhi nyime nyaka 2a. | | |  |  |  |  |
| --- | --- | --- | --- | --- | --- | --- | --- |
| Kuom dweche 12 mokalo, ka ne in enonro, be ne iyudo sir mar jahocho mar mbese mane  obiro en owuon? | Ooyo –> chikri nyaka 3 | |  |  |  |  |  |
|  | Akia |  |  |  |  |  |  |
|  | Odagi duoko | |  |  |  |  |  |
| Daher mondo inyisa kaka bath lemo moko mag nonro oseromi kitiyo gi ratil moluwoni: oroma | | | | | | | |
| ahinya, oroma, ediere, ok oroma, ok oroma ahinya. | | | | | | | |
|  | *Som matek* | | | | | *Kik isom matek* | |
| a. | Oroma ahinya | Oroma | Ediere | Ok oroma | Ok oroma ahinya | Akia | Odagi duoko |
| Ere kaka magi oseromi: |  |  |  |  |  |  |  |
| b. |  |  |  |  |  |  |  |
| Yot mar tudruok  gi jahocho? |  |  |  |  |  |  |  |
| c. |  |  |  |  |  |  |  |
| Yoo  maling’ling’ mar tudruok mar hocho/kinde mag hocho? |  |  |  |  |  |  |  |
| d. |  |  |  |  |  |  |  |
| Yor osiep mar  tudruok gi jahocho? |  |  |  |  |  |  |  |
| e. |  |  |  |  |  |  |  |
| Bedo ni ne inyalo  dhi ir jahocho mayot ka ionge gi luoro? |  |  |  |  |  |  |  |
|  |  |  |  |  |  |  |  |
| f. |  |  |  |  |  |  |  |

| Bedo ni jahocho ne jaratiro? |  |  |  |  |  |  |  |
| --- | --- | --- | --- | --- | --- | --- | --- |
| g. |  |  |  |  |  |  |  |
| Kony mar  jahocho mondo imuony yath? |  |  |  |  |  |  |  |
| h. |  |  |  |  |  |  |  |
| Kony mar  jakony mar mbese mondo okonyi ipar biro eklinik? |  |  |  |  |  |  |  |

A4A Intervention Experience Survey 17 October 2019

| i. |  |  |  |  |  |  |  |
| --- | --- | --- | --- | --- | --- | --- | --- |
| Nyalo mari mar  yudo weche mang’eny kata  wuoyo gi ng’ato eklinik kuom ngimani? |  |  |  |  |  |  |  |
| j. |  |  |  |  |  |  |  |
| Duto tee, sir mar  jahocho mane iyudo ne oromi marom nade? |  |  |  |  |  |  |  |
| k. |  |  |  |  |  |  |  |
| Duto tee, kony  mar jahocho engimani gi gik madich gi ngimani maber no oromi marom nade? |  |  |  |  |  |  |  |
|  | Daher  mang’eny ahinya ahinya | Daher | Daher | Daher | Ok daher kata matin | Akia | Odagi |
|  |  | ahinya | ediere | matin |  |  | duoko |
| l. |  |  |  |  |  |  |  |
| Be ibiro hero  mar dhi nyime gi yudo sir mar jahocho owuon ekinde mabiro, ka dipo ni nyalore? |  |  |  |  |  |  |  |
| m. | £ Kar od thieth | | | | | | |

|  | £ Edalana |
| --- | --- |
| Duto tee ne uromo gi jahocho kanye? | £ Eskunda |
|  | £ Mamoko (ler ) |
| (*check all that* | £ Akia |
| *apply*)? | £ Odagi duoko |
| n. | Odiechieng  kodiechieng |
| En mang’eny marom nade mane in gi jahocho ne uromo wang’ gi  wang’? | Ndalo matin e juma |
|  | Juma  kajuma |
|  | Ndalo matin edwe |
|  | Dwe ka  dwe |
|  | Matin ne dwe ka dwe |
|  | Akia |
|  | Odagi duoko |
| o. | di 20 kata nyadi  mang’eny |
| En kwan mar nyadidi koriwo te ma diwach ni  in kod jahocho ne uromo? | di 10-19 |
|  | di 5-9 |

A4A Intervention Experience Survey 17 October 2019

|  | di 1-4 |
| --- | --- |
|  | Akia |
|  | Odagi duoko |
| p. | Machiegni machiegni mangeny |
| Di iwach ni limbe gi jahocho ne machiegni machiengni  mang’eny ahinya, mang’eny  maber, kata ok ne mang’eny  moromo? | Machiegni machiegni mang’eny maber |
|  | Machiegni machiegni mang’eny ma ok oromo |
|  | Akia |
|  | Odagi duoko |
| q. | Wuoyo/oro ote esim |
| Be inyalo wacho ni ni thoth mbekni gi jahocho ne gin ote machuok, wuoyo, kata ma  ote gi wuoyo ne dwa rom? | Wuoyo wang’ gi wang’ |
|  | Kinde ma dwa romre mar moro ka moro |
|  | Akia |
|  | Odagi duoko |
| r. | Oroma ahinya |
| Yor tudruok mokuongo ne oromi marom  nade? | Oroma |
|  | Ediere |
|  | Ok oroma |
|  | Ok oroma ahinya |
|  | Akia |
|  | Odagi duoko |
| s. | Ber ahinya ahinya |
| Be inyalo wacho  ni tudruok gi | Ber ahinya |

| jahocho ne ber ahinya ahinya, ber ahinya, ber, ok ber ahinya kata onge ber kata matin? | Ber |
| --- | --- |
|  | Ok ber ahinya |
|  | Ok ber kata matin |
|  | Akia |
|  | Odagi duoko |
| t. | Ee |
| Be ng’at ang’ata ne oneno ote machuok kata owinjo mbekniu gi jahocho mane ok idwa ni gi  nee kata winji? | Ooyo |
|  | Akia |
|  | Odagi duoko |

A4A Intervention Experience Survey 17 October 2019

| u. | Ee |
| --- | --- |
| Be ma no okelo  duoko marach? | Ooyo |
|  | Akia |
|  | Odagi duoko |
| *v.* | £ Fwenyo ni an kod kute mag Ayaki |
| Ka ee, en kit  duoko marach mage? | £ Penjo penjo mang’eny ahinya mane omiyo ok abedo thuolo |
|  | £ Dhao gi jonyuol/jarit |
|  | £ Dhao gi jahera/jaot |
| *Ket alama kuom* | £ Dhao gi osiep/wat |
| *duto mowinjore* | £ Mamoko (ler: ) |
|  | £ Akia |
|  | £ Odagi duoko |
| *Interviewer notes* |  |
| *on in-person* |  |
| *Navigation* |  |

**Pesa Mioro koluore gi Chike**

| 3. | Ee –> dhi nyime nyaka  3a. | |  |  |  |  |  |
| --- | --- | --- | --- | --- | --- | --- | --- |
| Edweche 12 mokalo, kane in enonro, be ni iyudo pesa mar biro eklinik kochiki kata kuom bedo gi pek mar kute  mother? | Ooyo –> chikri nyaka 4 | |  |  |  |  |  |
|  | Akia |  |  |  |  |  |  |
|  | Odagi duoko | |  |  |  |  |  |
| Daher mondo inyisa kaka bath lemo moko mag nonro oseromi kitiyo gi ratil moluwoni: oroma | | | | | | | |
| ahinya, oroma, ediere, ok oroma, ok oroma ahinya. | | | | | | | |
|  | *Som matek* | | | | | *Kik isom matek* | |
| a. | Oroma ahinya | Oroma | Ediere | Ok oroma | Ok oroma ahinya | Akia | Odagi duoko |
| Ere kaka magi  oseromi: |  |  |  |  |  |  |  |
| b. |  |  |  |  |  |  |  |

| Yot mar yudo pesa kowuok enonro. |  |  |  |  |  |  |  |
| --- | --- | --- | --- | --- | --- | --- | --- |
| c. |  |  |  |  |  |  |  |
| Yoo  maling’ling’ mane pesa mioro ni ichiwonie? |  |  |  |  |  |  |  |

A4A Intervention Experience Survey 17 October 2019

| d. |  |  |  |  |  |  |  |
| --- | --- | --- | --- | --- | --- | --- | --- |
| Yor osiep mar  jatich mane chiwo pesa? |  |  |  |  |  |  |  |
| e. |  |  |  |  |  |  |  |
| Bedo ni ne  inyalo dhi ir jatich mane chiwo pesa mayot ka ionge gi luoro? |  |  |  |  |  |  |  |
| f. |  |  |  |  |  |  |  |
| Bedo ni jatich  machiwo pesa ne jaratiro? |  |  |  |  |  |  |  |
| g. |  |  |  |  |  |  |  |
| Yor kony mar  jatich machiwo pesa? |  |  |  |  |  |  |  |
| h. |  |  |  |  |  |  |  |
| Kony mar jatich  machiwo pesa mondo imuony yath? |  |  |  |  |  |  |  |
| i. |  |  |  |  |  |  |  |
| Kony mar jatich  machiwo pesa mondo omi ipar biro eklinik? |  |  |  |  |  |  |  |
| j. |  |  |  |  |  |  |  |
| Nyalo mari mar yudo weche  mang’eny kata wuoyo gi ng’ato eklinik kuom  ngimani? |  |  |  |  |  |  |  |

| k. |  |  |  |  |  |  |  |
| --- | --- | --- | --- | --- | --- | --- | --- |
| Duto tee, pesa mioro mane iyudo noromi marom nade? |  |  |  |  |  |  |  |
| l. |  |  |  |  |  |  |  |
| Duto tee, kony  mar pesa mioro mane iyudo engimani gi gik madich gi ngimani maber no oromi marom nade? |  |  |  |  |  |  |  |

A4A Intervention Experience Survey 17 October 2019

|  | Daher | Daher mang’eny  ahinya | Daher | Daher | Ok daher | Akia | Odagi |
| --- | --- | --- | --- | --- | --- | --- | --- |
|  | mang’eny ahinya ahinya |  | ediere | matin | kata matin |  | duoko |
| m. |  |  |  |  |  |  |  |
| Be ibiro hero  mar dhi nyime gi yudo pesa mioro ekinde mabiro, ka dipo ni nyalore? |  |  |  |  |  |  |  |
| n. | Ee |  |  |  |  |  |  |
| Be ni iyudo kwan pesa mane igeno kowuok  enonro? | Ooyo |  |  |  |  |  |  |
|  | Akia |  |  |  |  |  |  |
|  | Odagi duoko | |  |  |  |  |  |
| o. | Matin ne Siling ma Kenya 500 | |  |  |  |  |  |
| Ne iyudo pesa adi kowuok enonro elimbe  ka limbe mochiki? | Siling ma Kenya 500 |  |  |  |  |  |  |
|  | Mang’eny ne Siling ma  Kenya 500 | |  |  |  |  |  |
|  | Akia |  |  |  |  |  |  |
|  | Odagi duoko | |  |  |  |  |  |
| p. | Odiechieng kodiechieng |  |  |  |  |  |  |
| En mang’eny marom nade mane in gi jahocho oromo  wang’ gi wang’? | Nyadi matin ejuma | |  |  |  |  |  |
|  | Juma  kajuma |  |  |  |  |  |  |
|  | Nyadi matin edwe | |  |  |  |  |  |
|  | Dwe kadwe |  |  |  |  |  |  |
|  | Matin ne dwe kadwe | |  |  |  |  |  |
|  | Akia |  |  |  |  |  |  |
|  | Odagi duoko | |  |  |  |  |  |
| q. | di 10-19 | | | | | | |

| Ne iyudo pesa mioro di di duto tee? | di 5-9 |
| --- | --- |
|  | di 1-4 |
|  | Akia |
|  | Odagi duoko |
| r. | Ee |
| Be ne nitie limbe mag klinik moro amora mane ok iyude pesa mioro kane  onego iyudi? | Ooyo |
|  | Akia |
|  | Odagi duoko |
| s. | Ee |
| Ka ee, be nonro no oleroni gima  omiyo? | Ooyo |
|  | Akia |
|  | Odagi duoko |

A4A Intervention Experience Survey 17 October 2019

| t. | Mang’eny ahinya |
| --- | --- |
| Di iwach ni pes mioro ne machiegni machiegni  mang’eny ahinya, mang’eny  maber, kata ok  ne mang’eny moromo? | Mang’eny maber |
|  | Ok mang’eny moromo |
|  | Akia |
|  | Odagi duoko |
| u. | Ber ahinya ahinya |
| Be diwach ni lony mari gi okang mar pesa mioro ne ber ahinya ahinya, ber ahinya, ber, ok ber ahinya kata ok ber kata  matin? | Ber ahinya |
|  | Ber |
|  | Ok ber ahinya |
|  | Ok ber kata matin |
|  | Akia |
|  | Odagi duoko |
| v. | Ee |
| Be inyalo wacho ni pesa mioro ne pesa moromo  kawo ting’ mari nyaka eklinik? | Ooyo |
|  | Akia |
|  | Odagi duoko |
| w. | Yor wuoth ki idhi/kiwuok eklinik |
| Onge duoko maber kata marach ne ma; be inyalo wacho  ni pesa mioro ne | Chiemo |

| thothne itiyogo e yor wuoth ki idhi/kiwuok eklinik, chiemo, kuom yor wuoth gi chiemo, gi machielo, kata onge kuom mamalogi tee? | Yor wuoth ki idhi/kiwuok eklinik gi chiemo |
| --- | --- |
|  | Gi machielo (ler ) |
|  | Onge moro amora man malo ka |
|  | Akia |
|  | Odagi duoko |
| x. | Ee |
| Be ne eneno pek moro amora gi  okang’ mar pesa mioro? | Ooyo |
|  | Akia |
|  | Odagi duoko |

A4A Intervention Experience Survey 17 October 2019

| y. |  | Ee |  |
| --- | --- | --- | --- |
| Be okang’ mar pesa mioro okelo duoko  moro amora marach? |  | Ooyo |  |
|  |  | Akia |  |
|  |  | Odagi duoko |  |
| *z.* |  | £ Fwenyo ni an kod kute mag  Ayaki |  |
| Ka ee, en kit duoko marach mane? |  | £ Penjo penjo mang’eny ahinya  mane omiyo ok abedo thuolo |  |
|  |  | £ Dhao gi jonyuol/jarit |  |
|  |  | £ Dhao gi jahera/jaot |  |
| *Ket eduto* |  | £ Dhao gi osiep/wat |  |
| *mowinjore* |  | £ Mamoko (ler: ) | |
|  |  | £ Akia | |
|  |  | £ Odagi duoko |  |
| *Interviewer notes* |  |  | |
| *on conditional cash* |  |  |  |
| *transfers* |  |  |  |
|  | | | |
| 4. |  |  | Ee → ka Ee, ket alama eduto mowinjore. |
| Yie ipar kuom kony mag thieth ma iseyudo oko  mar nonro mar A4A: | | | Ooyo → dhie **d** |
|  | | | Akia → dhie **d** |
| Kinde mane intie A4A, be iseyude moro amora kowuok enoro moko kata migape: sir mar pesa, ote machuok mioro moluore, sir mar hocho esim kata ekar od thieth/dala gi jatich klinik, jarit theith mar oganda, kata jotim nonro moko? Ka ee, mage?  *Ka ee, keta lama kuom duto mowinjore* | | | Odagi duoko → dhie **d** |
|  |  |  | *Ket alama eduto mowinjore* |
|  | | | £ Ote machuok moluore → dhie **a** |
|  | | | £ Sir mar pesa → dhie **b** |
|  | | | £ Sir mar hocho → dhie **c** |
|  | | | £ Onge (mana ka onge mochuo malo kanyo) → dhie **d** |
|  | | | £ Akia→ dhie **d** |
|  | | | £ Odagi duoko → dhie **d** |
| a. Ote machuok (*mopogore gi nonro mar A4A*) | | | |
| i. | Mang’eny marom nade? | | Odiechieng’ kodiechieng’ |
|  |  | | Juma ka juma |
|  |  | | Dwe kadwe |
|  |  | | Matin ne dwe kadwe |
|  |  | | Akia |
|  |  | | Odagi duoko |
| ii. |  |  | £ Paro chieng mochiki eklinik |
|  | Ote machuok go ne wuoyo kuom  ang’o? *Ket alama kuom duto mowinjore* | | £ Koso dhi eklinik chieng mochiki, yie iduogi |

|  | | £ Lando gige ngima |
| --- | --- | --- |
|  |  | £ Duko mag klinik |
|  |  | £ Mamoko (ler ) |
| iii. |  | Eklinik ma yude thieth |
|  | Migao kata nonro mane mane oro  gi? | Nonro mamoko (ler nying ) |
|  |  | Akia |
|  |  | Odagi duoko |
| b. Sir mar pesa (*mopogore gi nonro mar A4A*) | | |
| i. | Mang’eny marom nade? | Odiechieng kodiechieng |

A4A Intervention Experience Survey 17 October 2019

|  | Juma kajuma |
| --- | --- |
|  | Dwe kadwe |
|  | Matin ne dwe kadwe |
|  | Akia |
|  | Odagi duoko |
| ii. Pesa mioroni en siling adi ma Kenya? | **Siling ma Kenya** 100 |
|  | **Siling ma Kenya** 200 |
|  | **Siling ma Kenya** 300 |
|  | **Siling ma Kenya** 400 |
|  | **Siling ma Kenya** 500 |
|  | **Siling ma Kenya** 600 |
|  | **Siling ma Kenya** 700 |
|  | Mamoko (ler ) |
|  | Akia |
|  | Odagi duoko |
| iii. Pesa mioro en mar ang’o? | £ Biro eklinik kochiki |
| *Keta lama eduto tee mowinjore* | £ Pesa miduoko mar wuoth |
|  | £ Kinde mikao eklinik |
|  | £ Pek mar kute mothir |
|  | £ Mamoko(ler) |
| iv. Migao kata nonro mane ma chiwo pesa | Klinik kama ayude thieth |
|  | Nonro moro (ler nying ) |
|  | Akia |
|  | Odagi duoko |
| c. Sir mar jahocho mar mbese (*moro mopogore gi nonro mar A4A)* | |
| i. | Odiechieng kodiechieng |
| Monuore mang’eny marom nade? | Nya di matin ejuma |
|  | Juma ka juma |
|  | Nya di matin edwe |
|  | Dwe kadwe |
|  | Matin ne dwe |
|  | Akia |
|  | Odagi duoko |
| ii. Be duto tee ne uromo wang’ gi wang’ koso esim? | Sim |
|  | Ng’ato owuon eklinik/dala |
|  | Sim kod ng’ato owuon |
|  | Akia |
|  | Odagi duoko |
| iii. |  |
| Nyisa maler ewi jahocho/sir mar  hocho? |  |
| iv. En migao kata nonro ma chiwo pesa? | Eklinik ka ayude rit mara |
|  | Nonro mamoko (ler nying ) |
|  | Akia |
|  | Odagi duoko |
| d. | Ee→ ka Ee, ket alama eduto te mowinjore piny. |
| Be in enonro moro amora mopogore gi  A4A? | Ooyo |
|  | Akia |
|  | Odagi duoko |
| i. Ka ee, yie iler kata wach nying’ nonro ma intie |  |

| Ka ee, yie iler kata wach nying’ nonro ma intie |  |
| --- | --- |

A4A Intervention Experience Survey 17 October 2019

| ii. | Nying kar od thieth (kat nying machielo ka ok kar od thieth |
| --- | --- |
| En e sub-kaunti gi kar od thieth  mane? |  |
|  | Sub-kaunti |
|  | Kaunti |
| Weche mamoko |  |

A4A Food Insecurity

SOM MATEK*:* Er*okamano kuom wuoyo koda kawuono. Kawuono, wadhi pong’o penjo mag nonro ma intie. Abiro penjo penjo kuomi, odi, gi ngima ma odiechieng kodiechieng’. Wabiro wuoyo kuom chiemo, ngima, thieth mari mar kute mag Ayaki, gi weche pesa. Duokogi biro dong malingling chutho, koro yie ikan ma ewiyi ka iduoko penjogi ma adier ma nyalore. Ka in gi penjo kata ka gimoro amora ma apenji ok ler, yie ichunga kendo abiro timo berna ahinya mondo achiw ler mang’eny. We wachak kod penjo moko kuom chamo chiemo e OT. Adwa ng’eyo chiemo gi gik mitiyogo madwarore mar yudo chiemo timo engimani. Penjo gi dwaro paro chien kuom JUMBE ANG’WEN mokalo.*

| Na | **Penjo** | **Yiero mar Duoko** |
| --- | --- | --- |
|  |  | 0. Ooyo ! Kal nyaka A2 |
| A 1 | Kuom jumbe angwen mokalo, bende ne chunyi no ochandore gi  paro ni ok ibi bedo gi chiemo moromo? | 1. Ee |
| A1.1 | Ma ne otimore mang’eny marom nade? | 1. Matin; dichiel kata diriyo ejumbe ang’wen mokalo |
|  |  | 2. Seche moko; didek nyaka dipar ejumbe ang’wen  mokalo |
|  |  | 3. Mang’eny; mang’eny ne dipar ejumbe ang’wen  mokalo |
| A2 |  | 0. No ! Chikri nyaka A3 |
|  | Kuom jumbe ang'wen mokalo, bende ne ok inyalo chamo kit  chiemo mane ihero nikech koso gik minyalo ti go? | 1. Ee |
| A2.1 | Ma ne otimore mang’eny marom nade? | 1. Matin; dichiel kata diriyo ejumbe ang’wen mokalo |
|  |  | 2. Seche moko; didek nyaka dipar ejumbe ang’wen  mokalo |
|  |  | 3. Mang’eny; mang’eny ne dipar ejumbe ang’wen  mokalo |
| A3 |  | 0. No ! Chikri nyaka A4 |
|  |  | 1. Ee |
|  | Kuom jumbe ang’wen mokalo, bende ne ochuno ni icham kit  chiemo manok kaluore gi koso gik minyalo ti go? |  |
| A3.1 | Ma ne otimore mang’eny marom nade? | 1. Matin; dichiel kata diriyo ejumbe ang’wen mokalo |
|  |  | 2. Seche moko; didek nyaka dipar ejumbe ang’wen  mokalo |
|  |  | 3. Mang’eny; mang’eny ne dipar ejumbe ang’wen  mokalo |
| A4 | Kuom jumbe ang’wen mokalo, be ne ochuno ni icham chiemo moko mane ok idwa chamo nikech onge mar gik mitiyogo mar yudo kit chiemo moko? | 0. No ! Chikri nyaka A5 |
|  |  | 1. Ee |

Namba

….|	|

….|	|

….|	|

….|	|

….|	|

….|	|

….|	|

| A4.1 | Ma ne otimore mang’eny marom nade? |
| --- | --- |

1. Matin; dichiel kata diriyo ejumbe ang’wen mokalo

| A5 | Kuom jumbe ang’wen mokalo, be no ochuno ni icham chiemo  matin ne mane iwinjo ka idwaro nikech ne onge chiemo moromo? |
| --- | --- |
| A5.1 | Ma ne otimore mang’eny marom nade? |
| A6 | Kuom jumbe ang’wen mokalo, be ne ochuno ni icham chiemo ndalo matin e odiehieng’ nikech ne onge chiemo moromo? |
| A6.1 | Ma ne otimore mang’eny marom nade? |
| A7 | Kuom jumbe ang’wen mokalo, be ne nitie chieng’ mane onge kit chiemo moro amora e odi nikech onge mar gik mitiyogo e yudo chiemo? |
| A7.1 | Ma ne otimore mang’eny marom nade? |
| A8 | Kuom jumbe ang’wen mokalo, mane idhi nindo kech gotieno nikech ne onge chiemo moromo? |
| A8.1 | Ma ne otimore mang’eny marom nade? |
| A9 | Kuom jumbe ang’wen mokalo, mane iriyo odiechieng’ tee gotieno ma ok ichamo gimoro amora nikech ne onge chiemo moromo? |
| A9.1 | Ma ne otimore mang’eny marom nade? |
| version 21 October 2019 | |

2. Seche moko; didek nyaka dipar ejumbe ang’wen mokalo
3. Mang’eny; mang’eny ne dipar ejumbe ang’wen mokalo
0. No ! Chikri nyaka A6
1. Ee
1. Matin; dichiel kata diriyo ejumbe ang’wen mokalo
2. Seche moko; didek nyaka dipar ejumbe ang’wen mokalo
3. Mang’eny; mang’eny ne dipar ejumbe ang’wen mokalo
0. Ooyo ! Chikri nyaka Q7
1. Ee
4. Matin; dichiel kata diriyo ejumbe ang’wen mokalo
5. Seche moko; didek nyaka dipar ejumbe ang’wen mokalo
6. Mang’eny; mang’eny ne dipar ejumbe ang’wen mokalo 0 = Ooyo (chikri nyaka Q8)

1 = Ee

1=Matin (dichiel nyaka diriyo ejumbe ang’wen mokalo)

2 = Seche moko (didek nyaka dipar ejumbe ang’wen)

3 = Mang’eny (mang’eny ne dipar kuom jumbe ang’wen mokalo)

0 = Ooyo (Chikri nyaka Q9) 1 = Ee

1=Matin (dichiel nyaka diriyo ejumbe ang’wen mokalo) 2 = Seche moko (didek nyaka dipar kuom ejumbe ang’wen) 3 = Mang’eny (mang’eny ne dipar kuom jumbe ang’wen mokalo)

0 = Ooyo (penjo orumo) 1 = Ee

1=Matin (dichiel kata diriyo kuom jumbe ang’wen

mokalo)

2 = Seche moko (didek nyaka dipar kuom jumbe ang’wen mokalo)

3 = Mang’eny (mang’eny ne dipar kuom jumbe ang’wen mokalo)

| **Appendix B37_A4A Extended Outcome Investigation and Classification form** | | | | | | | |
| --- | --- | --- | --- | --- | --- | --- | --- |
| **Study ID: Unique Patient Number:**  **Date:** ___________ **VL investigation:** ☐ Month 6 post rerandomization ☐Year 1 ☐ Year 2  DD-MMM-YYYY | | | | | | | |
| **Anniversary Date:** ___________  DD-MMM-YYYY  **Date of Enrolment:** ___________  DD-MMM-YYYY | | | | **Viral Load (VL) window:** | | | |
|  |  |  |  | Start date: _________________  DD-MMM-YYYY | | | End date: _________________  DD-MMM-YYYY |
| **Section A: Patient status during VL window** | | | | | | | |
| **Died before or during VL window?** | | | ☐ No ↓ | | ☐ Yes: Death date_________________  DD-MMM-YYYY  *Fill ADAPT OA form and discontinuation form Move to Section D* | | |
| **Withdrew from ADAPT study before or during VL window?** | | | ☐ No ↓ | | ☐ Yes: Withdrawal date_________________  DD-MMM-YYYY  *Fill ADAPT withdrawal form* *Move to Section D* | | |
| **Transferred out of ADAPT clinics before or during the VL window?** | | | ☐ No  *Move to Section C* | | ☐ Yes: Transfer-out date_________________  DD-MMM-YYYY  *Fill discontinuation form, if needed Move to Section B* | | |
| **Section B: Transfer-out patient** | | | | | | | |
| **Type of transfer out:** ☐ Self Transfer ☐ Official Transfer | | | | | | | |
| **Can you confirm the patient attended a new clinic?** | ☐ No → | ☐ Staff is certain patient is/was not in care during window  ☐ Staff has no knowledge of patient whereabouts *Move to Section D* | | | | | |
|  | ☐ Yes ↓  *Attempt to get VL data. If able, enter data in Redcap. STOP HERE*  *If unable to get VL information within the window period→* | ☐ New clinic refused to provide any additional information on the patient  ☐ New clinic has not taken VL because patient not on treatment, or recently started  treatment, or patient enrolled as new at the clinic  ☐ New clinic has some evidence patient is in care but cannot obtain VL  ☐ No VL result available, reason unknown  ☐ New clinic certain patient is/was not in care in the VL window  ☐ National outage of viral load reagents    ☐ *New clinic obtained VL outside window period (VL date and result) Move to Section D* | | | | | |
| **Section C: Non-transfer patient** | | | | | | | |
| **Did patient have a visit to ADAPT clinic in the VL window?** | ☐ No → | ☐ Staff is certain patient is/was not in care during window  *If patient transferred filled Section B. If patient died, fill Section A.*  ☐ Staff have some evidence patient is in care, but cannot obtain VL  ☐ Staff has no knowledge of patient whereabouts *Move to Section D* | | | | | |
|  | ☐ Yes → | ☐ Clinician refused to order because patient not on treatment or recently restarted  ☐ Clinician refused to order because ADAPT window did not match time points for  clinical VL  ☐ Clinician did not order, reason unknown  ☐ Clinician ordered, but no evidence VL ever collected  ☐ No VL ordered, but known to staff to be off medication completely  ☒ National outage of viral load reagents  *Move to Section D* | | | | | |
| **Section D: Data sources (select multiple choices)** | | | | | | | |
| **Data sources** | ☐ Clinic patient file ☐ Clinic Lab Log ☐ New clinic staff *(transfer patients only)*  ☐ Clinic staff (e.g. SSD, clinical team, Lab technicians) ☐ A4A records (e.g. Study files, logs, diaries)  ☐ IP-Nav knowledge ☐ E-navigator knowledge ☐ A4A LTFU investigator  □CM team/Desk review team □VL/ eid website | | | | | | |
| **Compiled by:** | Initials: _______________________ | | | | | Date:_______________________  DD-MMM-YYYY | |
|  |  | | | | |  | |

**Provide additional notes and summary of patient situation with handwritten notes on the back of this form à à**

**Version 2.0 2022.01.18**

Appendices C:	Letters of support

NASCOP

KEMRI

Kisumu MOH

FACES

Translation Certificates

**CERTIFICATE OF TRANSLATION**

I **Mary Jow** do hereby testify that I translated the **English versions** of **A4A English Verbal Assent Script for DCE; A4A English Verbal Consent Script for DCE; English Verbal Parental Script for DCE; English Focus Group Discussion Guides; English IDI Guide and, A4A Incentives DCE Tool into Kiswahili for A4A Adapt R Study**. I certify that these are accurate and true translations to the best of my ability.

Signed:___________________	Date: July 5, 2020

Address_ P.O.Box 31-40132 RABUOR Email: [maryjow2009@gmail.com](mailto:maryjow2009@gmail.com)

I_**Harriet Fridah Adhiambo** do hereby testify that I have reviewed the **Kiswahili versions** for the above named documents and I have compared them with the English versions of the same documents and found they were properly translated into **Kiswahili**.

Signed: Date: 5^th^ July 2020

Address: 40100-614	Email:_fridahadhiambo@gmail.com

Appendices D	Consent forms

| **Consent List** | **English version** | **Kiswahili version** | **Dholuo version** |
| --- | --- | --- | --- |
| FGD consent  FGD parental consent  FGD assent | 22 June 2020, v3.0  22 June 2020, v3.0  22 June 2020, v3.0 |  |  |
| *Workshop*  Verbal consent script  Verbal parental consent script  Verbal assent script | 22 June 2020, v3.0  22 June 2020, v3.0  22 June 2020, v3.0 |  |  |
| Overall consent  Overall parental consent  Overall assent | 22 June 2020, v3.0  22 June 2020, v3.0  22 June 2020, v3.0 |  |  |
| IDI consent  IDI parental consent  IDI assent | 22 June 2020, v3.0  22 June 2020, v3.0  22 June 2020, v3.0 |  |  |
| DCE Consent script  DCE parental consent script  DCE assent script | 22 June 2020, v3.0  22 June 2020, v3.0  22 June 2020, v3.0 |  |  |
| Peer navigation interview and FGD consent | 14 November 2023 v1.0 |  |  |

**OVERALL ENGLISH ASSENT FORM**

**KENYA MEDICAL RESEARCH INSTITUTE**

**WASHINGTON UNIVERSITY, ST. LOUIS**

**ASSENT TO PARTICIPATE IN A RESEARCH STUDY *FOR PARTICIPANTS 14 - 17 YEARS OLD***

**Study Title:** *Adaptive Strategies to Prevent and Treat Failures of Retention in Adolescent HIV Care (A4A Study)*

INVESTIGATORS AND INSTITUTIONAL AFFILIATIONS:

| Researcher | Institution | Contact |
| --- | --- | --- |
| Dr. Elizabeth Bukusi | Kenya Medical Research Institute | +254 733-617 503 |
| Eliud Akama | Kenya Medical Research Institute | +254 728-590 376 |
| Dr. Elvin Geng | Washington University, St. Louis | +1-415-430-5589 (USA) |
| Dr. Lisa Abuogi | University of Colorado, Denver | +1-303-724-7430 (USA) |
| Dr. Zachary Kwena | Kenya Medical Research Institute | +254 722-386 714 |
| Starley Shade | University of California, San Francisco | +1-415-794-4354 (USA) |
| Lina Montoya | University of Notrh Carolina at Chapel Hill | +1-4105857470 (USA) |

**Study Location:** Kisumu County

You are being asked to take part in a research study. The box below tells you important things you should think about before deciding to join the study. We will provide more detailed information below the box. Please ask questions about any of the information before you decide whether to participate. You may also wish to talk to others (for example, your family, friends, or your doctor) about this study, before agreeing to join.

- **Voluntary Assent**. You are being asked to volunteer for a research study. It is up to you whether you choose to participate or not. There are no penalties and you will not lose anything if you decide not to join or if after you join, you decide to quit.
- **Purpose**. We are doing this research to find out which combination of strategies results in the best engagement in HIV care among adolescents and young adults. Staying in care and getting viral load to zero is very important to make sure youth living with HIV stay healthy. But, adolescents and young adults face a lot of barriers to adherence and retention. In this study we are trying to learn how to give adolescents and young adults the strategy that helps them stay in care and stay at zero viral load.
- **Participants.** About 880 adolescents and young adults between the ages of 14-24 years who are living with HIV in Kisumu County will be in this study.
- **Duration.** Your participation in the study will take about 4	years.
- **Procedures and Activities.**

In this study, you will be screened and randomly assigned to either one of the two initial intervention strategies, either standard of care or electronic peer navigation (automated SMS messages and peer support through mobile phone). If you miss your clinic appointment by 14 days OR have a high viral load, you will be randomly re-assigned to either standard of care, conditional cash transfer (receiving money), or in-person peer navigator.

We will ask you survey questions about your living situation, health and social well-being, and HIV knowledge and adherence. The survey will take place at enrollment, if you are re-assigned (if you miss a clinic visit by more than 14 days OR have a high viral load), and after you have been in the study for 1 year and again at 2 years. We will also ask you questions about your satisfaction and experiences with interventions you may have received if you are re-assigned, after you have been in the study for 1 year and again at 2 years. The survey will be conducted at the health facility but may be conducted in the community if needed. The survey will take about 45-60 minutes.

We will also take routine clinic data from your medical records. If your viral load is not taken at the clinic when it is routinely due, the study may contact you and take blood for viral load testing about every 6 months.

- **Risks.**
- Pain or discomfort from blood draw.
- Accidental disclosure of HIV status. However, we will however put safety measures in place to ensure your HIV status is protected.
- **Benefits**. You may not receive any direct benefits from participating in this study. You may receive SMS text messages and peer navigation through mobile phone, cash transfer, or an in-person peer navigator. These interventions may help you stay in HIV care and take your medications well and that in turn may help your health. In addition, the lessons we learn from this study will help improve adolescent HIV care in general.
- **Alternatives.** You are free to choose not to participate in the study. If you decide not to take part in this study, there will be no penalty to you. You will not lose any of your regular benefits, and you can still get your care from your clinic the way you usually do.

**Procedures and Activities**

First, your parent or caregiver will be asked for permission for you to be in this study. In the event your parent/caregiver is not present during ths recruitment, the Research Assistant will contact your parent/caregiver to consent over the phone. If your parent or caregiver does not agree, you cannot be in the study. If your parent or caregiver does agree, and you agree too, here’s what will happen next:

***At study enrollment***

We will ask you questions about your living situation, health and social well-being, and HIV knowledge and adherence. **This survey of questions will take about 45-60 minutes.**

You will be assigned to one of two engagement-in-care intervention strategies. Selection of the intervention that you will receive will be random, a process similar to flipping a coin. The intervention strategies are:

1. **“Standard of care”** which is that if you fail to make your appointment, the clinic’s usual procedure of looking for you to remind you to come for your visit will take place. Or if you have a high viral load the clinic will counsel you to get your viral load to zero.
2. **E-navigation,** which is peer support through mobile phone and automated SMS messages. SMS text messages will be sent to you regularly with focus on health promotion. A peer navigator will help you, through information and counseling to engage in care. Interactions with the navigator will be through mobile phone messaging or conversations. This communication can be on your favorite App, for example through regular phone messaging, What’s App, Snapchat, or Facebook Messenger. Communication will be approximately weekly for the first two months and then monthly, with the possibility to talk more or less often if needed.

Standard of care or e-navigation intervention will last for one year if you do not miss a clinic visit by more than 14 days less OR have a high viral load.

We will continue to follow you in this study for a period of two years, following study enrollment.

*If you miss a clinic visit by more than 14 days OR have a high viral load > 1000 copies/ml:*

If you miss your clinic visit by more than 14 days OR have a high viral load, regardless of which intervention you first received, we will look for you in the community. If we are able to contact you, we will ask you the survey questions again, questions about your satisfaction and experiences with interventions you may have received, reasons you did not come back, and if you have transferred to another health facility. This survey may be conducted at the health facility or in the community. This survey will take about 45-60 minutes. If we cannot find you, we will attempt to speak with a contact if you have provided one to determine your whereabouts.

If we are unable to contact you, we will randomly re-assign you after a missed appointment to one of the following three interventions to help you re-engage in care:

1. **“Standard of care”.** which is that you will receive information about the importance of continuing care and encouragement to return to clinic. If we are not able to contact you in person, the clinic will try to reach you by phone or a home visit which is standard clinic practice. Or if you have a high viral load the clinic will counsel you to get your viral load to zero.
2. **Conditional cash transfer.** If you are assigned to conditional cash transfer, the study staff will issue Kshs. 500/= to you for attending your scheduled clinic visit within 3 days and another Kshs. 500 for having a suppressed viral load up to 2 times within a year. You may opt to receive the money through Mpesa or cash.
3. **In-person peer navigator.** If you are assigned to in-person peer navigation, you will be assigned an in-person peer navigator matched to your gender and approximate age. This will be a person from the clinic who will seek to help you, through information, counseling and support in your community, to stay in care and get to zero viral load. This person will interact with you in person and possibly by phone. This person will first meet with you and your caregiver or parent at your home (or other location you prefer) and then meet with you approximately monthly at the location you choose. Meetings may be more or less often than monthly, but not more often than weekly. If you experience continued difficulties like more missed visits or high viral load, this person will continue to work with you to help you overcome these difficulties.

Standard of care, conditional cash transfer, or in-person navigation intervention will last for one year following re-assignment.

We will continue to follow you in this study for a period of two years, following study enrollment.

*One year and two years after study enrollment*

We will ask you survey questions about your living situation, health and social well-being, and HIV knowledge and adherence, and satisfaction and experiences with interventions you may have received in the study at both one and two years after study enrollment. This survey will take about 45-60 minutes.

*Throughout the study*

You will continue to receive your health care at the clinic as usual. Data from your routine clinic visits will be taken from your medical records (eg. enrollment date, health status including viral load, and ART initiation date).

If your clinic has not tested the level of HIV virus in your body as per the Kenya national guidelines, we will ask you to give some blood for testing of the level of HIV virus in your body, which will require about 5 mL (about one teaspoon) of blood about every 6 months. If you are at clinic during that time, the blood will be taken in a routine way at the health facility. If you are not coming to the clinic, we will look for you and ask you to provide a sample to someone who can draw blood outside of the health facility. The viral load results will be available to you at the clinic.

We will also look at your clinical records to determine information about your care such as your clinic visits, in order to know whether we need to look for you. We will use information recorded in your clinical record to understand how these factors affect your ability to continue to come to care.

Should we make any changes to the study, you will be informed. Also, if new information becomes available as you are in the study that you need to know, we will tell you.

**Risks**

We plan to check the level of HIV virus in your body one time in this study if the clinic has not already done so. There is a risk of pain or discomfort from the blood draw. There is the rare risk of infection from blood draw. For more information about risks and side effects, ask one of the doctors at the clinic.

There is a possibility that someone may see the SMS messages we send to you. SMS messages will not contain any mention of HIV and so even if someone were to find your phone they would not learn anything about your HIV status. However, this may potentially be embarrassing to you, and we would like you to be aware of this risk before you decide to join the study.

There is also a risk that some of the questions we will ask about your feeling and experiences in care might make you uncomfortable. You are free to decline to answer any questions you do not wish to answer or to stop participating at any time.

Another risk is that others could find out that you are participating in this research study or see your private medical or health information, such as your HIV status. We will do everything we can to make sure this does not happen. The study staff and peer navigators are specially trained to keep your information private and to protect your information so others don’t find out.

**Benefits**

You may not receive any direct benefits from participating in this study. You may receive SMS text messages and peer navigation through mobile phone, cash transfer, or an in-person peer navigator. These interventions may help you engage in care and that in turn may help your health. At the end of the study, the results will help us determine the best strategy or combination of strategies to use for helping people remain engaged in care.

**Reimbursement**

**You will be given a transport reimbursement of Kshs. 500/= upon study enrollment.**

**If you are assigned to the conditional cash transfer intervention you will receive Kshs. 500/= for attending scheduled clinic visits within 3 days and having a suppressed viral load up to 2 times in one year.**

**Alternatives**

You are free to choose not to participate in the study. If you decide not to take part in this study, there will be no penalty to you. You will not lose any of your regular benefits, and you can still get your care from your clinic the way you usually do.

**Confidentiality**

We will do our best to ensure that the personal information gathered for this study is kept private. However, we cannot guarantee total privacy. Your personal information may be given out if required by law. If information from this study is published or presented at scientific meetings, your name and other personal information will not be used.

Government or university staff sometimes review studies such as this one to make sure they are being done safely and legally. If a review of this study takes place, your records may be examined. The reviewers will protect your privacy. The study records will not be used to put you at legal risk of harm. Organizations that may look at and/or copy your research records for research, quality assurance, and data analysis include:

- The Washington University, St. Louis
- The Kenya Medical Research Institute
- University of California San Francisco
- University of Colorado, Denver
- University of California, Berkeley

Participation in research may involve a loss of privacy, however we will put safety measures in place to protect your confidentiality. A medical record will be created because of your participation in this study. Your consent form and some of your research test results will be included in this record. Therefore, your other health care providers may see your test results and become aware of your participation. Hospital regulations require that all health care providers treat information in medical records confidentially. Any additional information about the study will be provided to you including the final study results.

**Participation**

You can decide to stop at any time without penalty or loss of benefits to which you are otherwise entitled. Just tell the study researcher or staff person right away if you wish to stop being in the study. You may stop being in the study by texting “STOP” to a study number we will provide you. Also, the study researcher may stop you from taking part in this study at any time if he or she believes it is in your best interest, if you do not follow the study rules, or if the study is stopped.

**Sponsorship**

The study is sponsored by the National Institutes of Health. None of the investigators have any financial conflict of interests. In situations where the study may be terminated at the discretion of the investigator or study sponsor, you will be informed the next course of action.

**Contact**

You can talk to the researcher(s) about any questions, concerns, or complaints you have about this study. Contact our study staff at 0733-819 835. If you wish to ask questions about the study or your rights as a research participant to someone other than the researchers or if you wish to voice any problems or concerns you may have about the study, you may contact Secretary of the Ethical Review Committee, Kenya Medical Research Institute at seru@kemri.org or P.O. BOX 54840-00200 Tel. 020-2722541, 0717719477. You may also contact the Washington University Human Research Protection Office at +1-314-747-6800.These committees are concerned with the protection of volunteers in research projects.

**CONSENT**

You have been given a copy of this consent form to keep.

**PARTICIPATION IN RESEARCH IS VOLUNTARY.** You have the right to decline to be in this study, or to withdraw from it at any point without penalty or loss of benefits to which you are otherwise entitled.

**Participant’s Statement**

This study described above has been explained to me. I volunteer to take part in this research. I have had a chance to ask questions. If I have future questions about the research, I can ask one of the investigators listed above. If I have questions about my rights as a research participant, I can contact those listed above.

I agree for my information to be used for future research and shared with other researchers without my additional consent with/without identifiers.

If you wish to participate in this study, you should sign below.

Date	Participant's Name and Signature for Consent

Date	Person Obtaining Consent (Name and signature)

***If illiterate***

I have witnessed the accurate reading of the consent form to the participant and the individual has had the opportunity to ask questions. I confirm that the individual has given consent freely**.**

Name of witness_____________________ AND

Signature of witness ***______________________***

Name of participant __________________ AND

Thumbprint of participant

Date ________________________

Copies to:	1. Investigators

2. Study participant

**ENGLISH OVERALL INFORMED CONSENT FORM**

**KENYA MEDICAL RESEARCH INSTITUTE**

**WASHINGTON UNIVERSITY, ST. LOUIS**

CONSENT TO PARTICIPATE IN A RESEARCH STUDY

**Study Title:** *Adaptive Strategies to Prevent and Treat Failures of Retention in Adolescent HIV Care (A4A Study)*

INVESTIGATORS AND INSTITUTIONAL AFFILIATIONS:

| Researcher | Institution | Contact |
| --- | --- | --- |
| Dr. Elizabeth Bukusi | Kenya Medical Research Institute | +254 733-617 503 |
| Eliud Akama | Kenya Medical Research Institute | +254 728-590 376 |
| Dr. Elvin Geng | Washington University, Missouri | +1-415-430-5589 (USA) |
| Dr. Lisa Abuogi | University of Colorado, Denver | +1-303-724-7430 (USA) |
| Dr. Zachary Kwena | Kenya Medical Research Institute | +254 722-386 714 |
| Starley Shade | University of California, San Francisco | +1-415 794-4354 (USA) |
| Lina Montoya | University of Notrh Carolina at Chapel Hill | +1-4105857470 (USA) |

**Study Location:** Kisumu County

You are being asked to take part in a research study. The box below tells you important things you should think about before deciding to join the study. We will provide more detailed information below the box. Please ask questions about any of the information before you decide whether to participate. You may also wish to talk to others (for example, your family, friends, or your doctor) about this study, before agreeing to join.

- **Voluntary Consent**. You are being asked to volunteer for a research study. It is up to you whether you choose to participate or not. There are no penalties and you will not lose anything if you decide not to join or if after you join, you decide to quit.

- **Purpose**. We are doing this research to find out which combination of strategies results in the best engagement in HIV care among adolescents and young adults. Staying in care and getting viral load to zero is very important to make sure youth living with HIV stay healthy. But, adolescents and young adults face a lot of barriers to adherence and retention. In this study we are trying to learn how to give adolescents and young adults the strategy that helps them stay in care and stay at zero viral load.
- **Participants.** About 880 adolescents and young adults between the ages of 14-24 years who are living with HIV in Kisumu County will be in this study.
- **Duration.** Your participation in the study will take about 4	years.
- **Procedures and Activities.**

In this study, you will be screened and randomly assigned to one of the two initial intervention strategies, either standard of care or electronic peer navigation (automated SMS messages and peer support through mobile phone). If you miss your clinic appointment by more than 14 days OR have a high viral load, you will be randomly re-assigned to either standard of care, conditional cash transfer (receiving money), or in-person peer navigator.

We will ask you survey questions about your living situation, health and social well-being, and HIV knowledge and adherence. The survey will take place at enrollment, if re-assigned (if you miss a clinic visit by more than 14 days OR have a high viral load), and after you have been in the study for 1 year and again at 2 years. We will also ask you questions about your satisfaction and experiences with interventions you may have received if you are re-assigned, after you have been in the study for 1 year and again at 2 years. The survey will be conducted at the health facility but may be conducted in the community if needed. The survey will take about 45-60 minutes.

We will also take routine clinic data from your medical records. If your viral load is not taken at the clinic when it is routinely due, the study may contact you and take blood for viral load testing about every 6 months.

- **Risks.**
- Pain or discomfort from blood draw.
- Accidental disclosure of HIV status. However, we will put safety measures in place to ensure your HIV status is protected.
- **Benefits**. You may not receive any direct benefits from participating in this study. You may receive SMS text messages, cash transfer, or a peer support navigator. These interventions may help you stay in HIV care and take your medications well and that in turn may help your health. In addition, the lessons we learn from this study will help improve adolescent HIV care in general.
- **Alternatives.** You are free to choose not to participate in the study. If you decide not to take part in this study, there will be no penalty to you. You will not lose any of your regular benefits, and you can still get your care from your clinic the way you usually do.

**Procedures and Activities**

If you agree to participate in this study, the following procedures will occur:

*At study enrollment*

We will ask you questions about your living situation, health and social well-being, and HIV knowledge and adherence. This survey will take about 45-60 minutes.

You will be assigned to one of two engagement-in-care intervention strategies. Selection of the intervention that you will receive will be random, a process similar to flipping a coin. The intervention strategies are:

3. **“Standard of care”** which is that if you fail to make your appointment, the clinic’s usual procedure of looking for you to remind you to come for your visit will take place. Or if you have a high viral load the clinic will counsel you to get your viral load to zero.
4. **E-navigation,** which is peer support through mobile phone and automated SMS messages. SMS text messages will be sent to you regularly with a focus on health promotion. A peer navigator will help you, through information and counseling to stay in care. Interactions with the navigator will be through mobile phone messaging or conversations. This communication can be on your favorite App, for example through regular phone messaging, What’s App, Snapchat, or Facebook Messenger. Communication will be approximately weekly for the first two months and then monthly, with the possibility to talk more or less often if needed.

Standard of care or e-navigation intervention will last for one year if you do not miss a clinic visit by more than 14 days OR have a high viral load.

We will continue to follow you in this study for a period of two years, following study enrollment.

*If you miss a clinic visit by more than 14 days OR have a viral load ≥ 1000 copies/ml:*

If you miss your clinic visit by more than 14 days OR have a high viral load, regardless of which intervention you first received, we will look for you in the community. If we are able to contact you, we will ask you the survey questions again, questions about your satisfaction and experiences with interventions you may have received, reasons you did not come back, and if you have transferred to another health facility. This will take about 45-60 minutes. This survey may be conducted at the health facility or in the community. If we cannot find you, we will attempt to speak with a contact if you have provided one to determine your whereabouts.

If we are unable to contact you, we will randomly re-assign you after a missed appointment to one of the following three interventions to help you re-engage in care:

4. **“Standard of care”** which is that you will receive information about the importance of continuing care and encouragement to return to clinic. If we are not able to contact you in person, the clinic will try to reach you by phone or a home visit which is standard clinic practice. Or if you have a high viral load the clinic will counsel you to get your viral load to zero.
5. **Conditional cash transfer.** If you are assigned to conditional cash transfer, the study staff will issue Kshs. 500/= to you for attending your scheduled clinic visit within 3 days and another Kshs. 500 for having a suppressed viral load up to 2 times in a year. You may opt to receive the money through Mpesa or cash.
6. **In-person peer navigator.** If you are assigned to in-person navigation, you will be assigned an in-person peer navigator matched to your gender and approximate age. This will be a person from the clinic who will seek to help you, through information, counseling and support in your community, to stay in care and get to zero viral load. This person will interact with you in person and possibly by phone. This person will first meet with you at your home (or other location you prefer) and then meet with you approximately monthly at the location you choose. Meetings may be more or less often than monthly, but not more often than weekly. If you experience continued difficulties like more missed visits or high viral load, this person will continue to work with you to help you overcome these difficulties.

Standard of care, conditional cash transfer, or in-person navigation intervention will last for one year following re-assignment.

We will continue to follow you in this study for a period of two years, following study enrollment.

*One year and two years after study enrollment*

We will ask you survey questions about your living situation, health and social well-being, and HIV knowledge and adherence, and satisfaction and experiences with interventions you may have received in the study at both one and two years after study enrollment. This survey will take about 45-60 minutes.

*Throughout the study*

You will continue to receive your health care at the clinic as usual. Data from your routine clinic visits will be taken from your medical records (e.g. enrollment date, health status including viral load, and ART initiation date).

If your clinic has not tested the level of HIV virus in your body as per the Kenya national guidelines, we will ask you to give some blood for testing of the level of HIV virus in your body, which will require about 5 mL (about one teaspoon) of blood around every 6 months. If you are at clinic during that time, the blood will be taken in a routine way at the health facility. If you are not coming to the clinic, we will look for you and ask you to provide a sample to someone who can draw blood outside of the health facility. The viral load results will be available to you at the clinic.

We will also look at your clinical records to determine information about your care such as your clinic visits, in order to know whether we need to look for you. We will use information recorded in your clinical record to understand how these factors affect your ability to continue to come to care.

Should we make any changes to the study, you will be informed. Also, if new information becomes available as you are in the study that you need to know, we will tell you.

**Risks**

We plan to check the level of HIV virus in your body one time in this study if the clinic has not already done so. There is a risk of pain or discomfort from the blood draw. There is the rare risk of infection from blood draw. For more information about risks and side effects, ask one of the doctors at the clinic.

There is a possibility that someone may see the SMS messages we send to you. SMS messages will not contain any mention of HIV and so even if someone were to find your phone they would not learn anything about your HIV status. However, this may potentially be embarrassing to you, and we would like you to be aware of this risk before you decide to join the study.

There is also a risk that some of the questions we will ask about your feeling and experiences in care might make you uncomfortable. You are free to decline to answer any questions you do not wish to answer or to stop participating at any time.

Another risk is that others could find out that you are participating in this research study or see your private medical or health information, such as your HIV status. We will do everything we can to make sure this does not happen. The study staff and peer navigators are specially trained to keep your information private and to protect your information so others don’t find out.

**Benefits**

You may not receive any direct benefits from participating in this study. You may receive SMS text messages and peer navigation through mobile phone, cash transfer, or an in-person peer navigator. These interventions may help you engage in care and that in turn may help your health. At the end of the study, the results will help us determine the best strategy or combination of strategies to use for helping people remain engaged in care.

**Reimbursement**

You will be given a transport reimbursement of Kshs. 500/= upon study enrollment.

If you are assigned to the conditional cash transfer intervention you will receive Kshs. 500/= for attending scheduled clinic visits within 3 days and having a suppressed viral load up to 2 times in one year.

**Alternatives**

You are free to choose not to participate in the study. If you decide not to take part in this study, there will be no penalty to you. You will not lose any of your regular benefits, and you can still get your care from your clinic the way you usually do.

**Confidentiality**

We will do our best to ensure that the personal information gathered for this study is kept private. However, we cannot guarantee total privacy. Your personal information may be given out if required by law. If information from this study is published or presented at scientific meetings, your name and other personal information will not be used.

Government or university staff sometimes review studies such as this one to make sure they are being done safely and legally. If a review of this study takes place, your records may be examined. The reviewers will protect your privacy. The study records will not be used to put you at legal risk of harm. Organizations that may look at and/or copy your research records for research, quality assurance, and data analysis include:

- The Washington University, St. Louis
- The Kenya Medical Research Institute
- University of California San Francisco
- University of Colorado, Denver
- University of California, Berkeley

Participation in research may involve a loss of privacy, however we will put safety measures in place to protect your confidentiality. A medical record will be created because of your participation in this study. Your consent form and some of your research test results will be included in this record. Therefore, your other health care providers may see your test results and become aware of your participation. Hospital regulations require that all health care providers treat information in medical records confidentially. Any additional information about the study will be provided to you including the final study results.

**Participation**

You can decide to stop at any time without penalty or loss of benefits to which you are otherwise entitled. Just tell the study researcher or staff person right away if you wish to stop being in the study. You may stop being in the study by texting “STOP” to a study number we will provide you. Also, the study researcher may stop you from taking part in this study at any time if he or she believes it is in your best interest, if you do not follow the study rules, or if the study is stopped.

**Sponsorship**

The study is sponsored by the National Institutes of Health. None of the investigators have any financial conflict of interests. In situations where the study may be terminated at the discretion of the investigator or study sponsor, you will be informed the next course of action.

**Contact**

You can talk to the researcher(s) about any questions, concerns, or complaints you have about this study. Contact our study staff at 0733-819 835. If you wish to ask questions about the study or your rights as a research participant to someone other than the researchers or if you wish to voice any problems or concerns you may have about the study, you may contact Secretary of the Ethical Review Committee, Kenya Medical Research Institute at seru@kemri.org or P.O. BOX 54840-00200 Tel. 020-2722541, 0717719477. You may also contact the Washington University Human Research Protection Office at +1-314-747-6800.These committees are concerned with the protection of volunteers in research projects.

**CONSENT**

You have been given a copy of this consent form to keep.

**PARTICIPATION IN RESEARCH IS VOLUNTARY.** You have the right to decline to be in this study, or to withdraw from it at any point without penalty or loss of benefits to which you are otherwise entitled.

**Participant’s Statement**

This study described above has been explained to me. I volunteer to take part in this research. I have had a chance to ask questions. If I have future questions about the research, I can ask one of the investigators listed above. If I have questions about my rights as a research participant, I can contact those listed above.

I agree for my information to be used for future research and shared with other researchers without my additional consent with/without identifiers.

If you wish to participate in this study, you should sign below.

Date	Participant's Name and Signature for Consent

Date	Person Obtaining Consent (Name and signature)

***If illiterate***

I have witnessed the accurate reading of the consent form to the participant and the individual has had the opportunity to ask questions. I confirm that the individual has given consent freely**.**

Name of witness_____________________ AND

Signature of witness ***______________________***

Name of participant __________________ AND

Thumbprint of participant

Date ________________________

Copies to:	1. Investigators

2. Study participant

**ENGLISH OVERALL PARENTAL CONSENT FORM**

**KENYA MEDICAL RESEARCH INSTITUTE**

**WASHINGTON UNIVERSITY, ST. LOUIS**

**PARENTS/GUARDIAN PERMISSION FORM FOR RESEARCH PARTICIPANTS 14 – 17 YEARS OLD**

**Study Title:** *Adaptive Strategies to Prevent and Treat Failures of Retention in Adolescent HIV Care (A4A Study)*

INVESTIGATORS AND INSTITUTIONAL AFFILIATIONS:

| Researcher | Institution | Contact |
| --- | --- | --- |
| Dr. Elizabeth Bukusi | Kenya Medical Research Institute | +254 733-617 503 |
| Eliud Akama | Kenya Medical Research Institute | +254 728-590 376 |
| Dr. Elvin Geng | Washington University, Missouri | +1-415-430-5589 (USA) |
| Dr. Lisa Abuogi | University of Colorado, Denver | +1-303-724-7430 (USA) |
| Dr. Zachary Kwena | Kenya Medical Research Institute | +254 722-386 714 |
| Starley Shade | University of California, San Francisco | +1-415 794-4354 (USA) |
| Lina Montoya | University of Notrh Carolina at Chapel Hill | +1-4105857470 (USA) |

**Study Location:** Kisumu County

We are asking your child to take part in a research study. The box below tells you important things you should think about before giving your child permission to join the study. We will provide detailed information below the box. Please ask questions about any of the information before you give permission. You may also wish to talk to others (for example, your family, friends, or your doctor) about this study, before consenting.

- **Voluntary Consent**. You are being asked to allow your child to participate in a research study. Your child will not face any penalties for not participating in the study or withdrawing from the study at any point.
- **Purpose**. We are doing this research to find out which combination of strategies results in the best engagement in HIV care among adolescents and young adults. Staying in care and getting viral load to zero is very important to make sure youth living with HIV stay healthy. But, adolescents and young adults face a lot of barriers adherence and retention. In this study we are trying to learn how to give adolescents and young adults the strategy that works best for them, at the time when it will be most helpful.
- **Participants.** About 880 adolescents and young adults between the ages of 14-24 years who are living with HIV in Kisumu County will be in this study.
- **Duration.** Your child will participate in the study for about 4	years.
- **Procedures and Activities.**

In this study, your child will be screened and randomly assigned to one of the two initial interventions strategies, either standard of care or electronic peer navigation (automated SMS messages and peer support through mobile phone). If your child misses his/her clinic appointment by 14 days OR has a high viral load, he/she will be randomly re-assigned to either standard of care, conditional cash transfer (receiving money), or in-person peer navigator.

Your child will be asked survey questions about his/her living situation, health and social well-being, and HIV knowledge and adherence. The survey will take place at enrollment, if re-assigned (if misses a clinic visit by more than 14 days OR has a high viral load) and after you have been in the study for 1 year and again at 2 years. We will also ask your child questions about his/her satisfaction and experiences with interventions he/she may have received, if he/she is re-assigned, after he/she has been in the study for 1 year and again at 2 years. The survey will be conducted at the health facility but may be conducted in the community if needed. The survey will take about 45-60 minutes.

We will also take routine clinic data from his/her medical records. If his/her viral load is not taken at the clinic when it is routinely due, the study may contact him/her and take blood for viral load testing about every 6 months.

- **Risks.**
- Pain or discomfort from blood draw.
- Accidental disclosure of HIV status. However, we will safety measures in place to ensure your child’s HIV status is protected.
- **Benefits**. Your child may not receive any direct benefits from participating in this study. Your child may receive SMS text messages Your child may receive SMS text messages and peer navigation through mobile phone, cash transfer, or an in-person peer navigator. These interventions may help your child stay in care and take medications well and that in turn may help his/her health. In addition, the lessons we learn from this study will help improve adolescent HIV care in general.
- **Alternatives.** You are free to choose not to allow your child to participate in the study. If you decide not to allow, there will be no penalty to your child. Your child will not lose any of your regular benefits, and he/she can still get your care from their clinic the way they usually do.

**Procedures and Activities**

If you agree to have your child to participate in this study, and he/she agrees too, the following procedures will occur:

*At study enrollment*

We will ask your child questions about his/her living situation, health and social well-being, and HIV knowledge and adherence. This survey will take about 45-60 minutes.

He/she will be assigned to one of two engagement-in-care intervention strategies. Selection of the intervention that he/she will receive will be random, a process similar to flipping a coin. The intervention strategies are:

5. **“Standard of care”** which is that if he/she fails to make their appointment, the clinic’s usual procedure of looking for him/her will apply. Or if your child has a high viral load the clinic will counsel him/her to get his/her viral load to zero.
6. **E-navigation,** which is peer support through mobile phone and automated SMS messages. SMS text messages will be sent to your child regularly with focus on health promotion. A peer navigator will help your child, through information and counseling to engage in care. Interactions with the navigator will be through mobile phone messaging or conversations. This communication can be on his/her favorite App, for example through regular phone messaging, What’s App, Snapchat, or Facebook Messenger. Communication will be approximately weekly for the first two months and then monthly, with the possibility to talk more or less often if needed.

Standard of care or e-navigation intervention will last for one year if your child does not miss a clinic visit by more than 14 days OR has a high viral load.

We will continue to follow your child in this study for a period of two years, following study enrollment.

*If your child misses a clinic visit by more than 14 days OR has a viral load ≥ 1000 copies/ml:*

If your child misses his/her clinic visit by more than 14 days OR has a high viral load, regardless of which intervention he/she first received, we will look for him/her in the community. If we are able to contact your child we will ask the survey questions again, questions about his/her satisfaction and experiences with interventions he/she may have received, reasons he/she did not come back, and if he/she has transferred to another health facility. This survey will take about 45-60 minutes. If we cannot find your child, we will attempt to speak with a contact you have provided to determine his/her whereabouts.

If we are unable to contact your child, we will randomly re-assign him/her on the 29^th^ day of missed appointment to one of the following three interventions to help your child re-engage in care:

- **“Standard of care”** which is that your child will receive information about the importance of continuing care and encouragement to return to clinic. If we are not able to contact your child in person, the clinic will try to reach you by phone or a home visit which is standard clinic practice. Or if your child has a high viral load the clinic will counsel him/her to get his/her viral load to zero.
- **Conditional cash transfer.** If your child is assigned to conditional cash transfer, the study staff will issue Kshs. 500/= to your child for attending his/her scheduled clinic visit within 3 days and another Kshs. 500 for having a suppressed viral load up to 2 times within a year. Your child may opt to receive the money through Mpesa or cash.
- **In-person peer navigator.** If you are child is assigned to in-person navigation, he/she will be assigned an in-person peer navigator matched to him/her on gender and approximate age. This will be a person from the clinic who will seek to help your child, through information, counseling and support in your community, to stay in care and get to zero viral load. This person will interact with your child in person and possibly by phone. This person will first meet with your child and you at your home (or other location you prefer) and then meet with your child approximately monthly at his/her preferred location. Meetings may be more or less often, but not more often than weekly. If your child experiences continued difficulties like more missed visits or high viral load, this person will continue to work with him/her to help overcome these difficulties.

Standard of care, conditional cash transfer, or in-person navigation intervention will last for one year following re-assignment.

We will continue to follow your child in this study for a period of two years, following study enrollment.

*One year and two years after study enrollment*

We will ask your child survey questions about his/her living situation, health and social well-being, and HIV knowledge and adherence, and satisfaction and experiences with interventions he/she may have received in the study at both one and two years after study enrollment. This survey will take about 45-60 minutes.

*Throughout the study*

Your child will continue to receive health care at the clinic as usual. Data from your routine clinic visits will be taken from your medical records (eg. enrollment date, health status including viral load, ART initiation date).

If the clinic has not tested the level of HIV virus in your child’s body as per the Kenya national guidelines, we will ask him/her to give some blood for testing of the level of HIV virus in his/her body, which will require about 5 mL (about one teaspoon) of blood around every 6 months. If your child is at clinic during that time, the blood will be taken in a routine way at the health facility. If he/she is not coming to the clinic, we will look for your child and ask him/her to provide a sample to someone who can draw blood outside of the health facility. The viral load results will be available at the clinic.

We will also look at your child’s clinical records to determine information about his/her care such as clinic visits, in order to know whether we need to look for him/her. We will use information recorded in your child’s clinical record to understand how these factors affect his/her ability to continue to come to care.

Should we make any changes to the study, you and your child will be informed. Or if your child has a high viral load the clinic will counsel him/her to get his/her viral load to zero.

**Risks**

We plan to check the level of HIV virus in your child’s body one time in this study if the clinic has not already done so. There is a risk of pain or discomfort from the blood draw. There is the rare risk of infection from blood draw. For more information about risks and side effects, ask one of the doctors at the clinic.

There is a possibility that someone may see the SMS messages we send to your child. SMS messages will not contain any mention of HIV and so even if someone were to find these messages they would not learn anything about your HIV status. However, this may potentially be embarrassing to both of you, and we would like you to be aware of this risk before you decide to allow your child to participate in the study.

There is also a risk that some of the questions we will ask your child about his/her feelings and experiences in care might make him/her uncomfortable. Your child is free to decline to answer any questions he/she does not wish to answer or to stop participating at any time.

Another risk is that others could find out that your child is participating in this research study or see his/her private medical or health information, such as his/her HIV status. We will do everything we can to make sure this does not happen. The study staff and peer navigators are specially trained to keep your child’s information private and to protect your information so others don’t find out.

**Benefits**

Your child may not receive any direct benefits from participating in this study. Your child may receive SMS text messages and peer navigation through mobile phone, cash transfer, or an in-person peer navigator. These interventions may help him/her engage in care and that in turn may help his/her health. At the end of the study, the results will help us determine the best strategy or combination of strategies to use for helping people remain engaged in care.

**Reimbursement**

Your child will be given a transport reimbursement of Kshs. 500/= upon study enrollment.

If your child is assigned to the conditional cash transfer intervention he/she will receive Kshs. 500/= for attending scheduled clinic visits within 3 days and having a suppressed viral load up to 2 times in one year.

**Alternatives**

Your child is free to choose not to participate in the study. If he/she decides not to take part in this study, there will be no penalty to him/her. He/she will not lose any regular benefits, and can still get care from his/her clinic the way he/she usually do.

**Confidentiality**

We will do our best to ensure that the personal information gathered for this study is kept private. However, we cannot guarantee total privacy. Your child’s personal information may be given out if required by law. If information from this study is published or presented at scientific meetings, your child’s name and other personal information will not be used.

Government or university staff sometimes review studies such as this one to make sure they are being done safely and legally. If a review of this study takes place, your child’s records may be examined. The reviewers will protect your child’s privacy. The study records will not be used to put your child at legal risk of harm. Organizations that may look at and/or copy your research records for research, quality assurance, and data analysis include:

- The Washington University, St. Louis
- The Kenya Medical Research Institute
- University of California San Francisco
- University of Colorado, Denver
- University of California, Berkeley

Participation in research may involve a loss of privacy, however we will put safety measure in place to protect your confidentiality. A medical record will be created because of your participation in this study. Your child’s consent form and some of the child’s research test results will be included in this record. Therefore, your child’s other health care providers may see his/her test results and become aware of your participation. Hospital regulations require that all health care providers treat information in medical records confidentially. Any additional information about the study will be provided to your child including the final study results.

**Participation**

Your child can decide to stop participating in the study at any time without penalty or loss of benefits to which he/she is otherwise entitled. Just tell the study researcher or staff person right away if your child wishes to stop being in the study. Your child may stop being in the study by texting “STOP” to a study number we will provide you. Also, the study researcher may stop your child from taking part in this study at any time if he or she believes it is in your child’s best interest, if your child does not follow the study rules, or if the study is stopped.

**Sponsorship**

The study is sponsored by the National Institutes of Health. None of the investigators have any financial conflict of interests. In situations where the study may be terminated at the discretion of the investigator or study sponsor, you will be informed the next course of action.

**Contact**

You can talk to the researcher(s) about any questions, concerns, or complaints you have about this study. Contact our study staff at 0733-819 835. If you wish to ask questions about the study or your rights as a research participant to someone other than the researchers or if you wish to voice any problems or concerns you may have about the study, you may contact Secretary of the Ethical Review Committee, Kenya Medical Research Institute at seru@kemri.org or P.O. BOX 54840-00200 Tel. 020-2722541, 0717719477. You may also contact the Washington University Human Research Protection Office at +1-314-747-6800. These committees are concerned with the protection of volunteers in research projects.

**CONSENT**

You have been given a copy of this consent form to keep.

**PARTICIPATION IN RESEARCH IS VOLUNTARY.** Your child has the right to decline to be in this study, or to withdraw from it at any point without penalty or loss of benefits to which he/she is otherwise entitled.

**Participant’s Statement**

This study described above has been explained to me. I volunteer to allow my child to participate in this research. I have had a chance to ask questions. If I have future questions about the research, I can ask one of the investigators listed above. If I have questions about my child’s rights as a research participant, I can contact those listed above.

I agree that the study uses my child’s information that excludes his name or clinic identification for future research and shared with other researchers without my additional consent.

If you allow your child to participate in this study, you should sign below.

Date	Participant’s Name

Date	Parent/Guardian's Name and Signature for Consent

Date	Person Obtaining Consent (Name and signature)

***If illiterate***

I have witnessed the accurate reading of the consent form to the participant and the individual has had the opportunity to ask questions. I confirm that the individual has given consent freely**.**

Name of witness_____________________ AND

Signature of witness __________________AND

Name of participant___________________AND

Thumbprint of participant

Date ________________________

Copies to:	1. Investigators

2. Study participant

***Adaptive Strategies to Prevent and Treat Failures of Retention in***

***Adolescent HIV Care (A4A Study)***

**ENGLISH A4A VERBAL ASSENT SCRIPT FOR DISCRETE CHOICE EXPERIMENT (DCE)*14 – 17 YEARS OLD***

INVESTIGATORS AND INSTITUTIONAL AFFILIATIONS:

| Researcher | Institution | Contact |
| --- | --- | --- |
| Dr. Elizabeth Bukusi | Kenya Medical Research Institute | +254 733-617 503 |
| Eliud Akama | Kenya Medical Research Institute | +254 728-590 376 |
| Dr. Elvin Geng | Washington University, Missouri | +1-415-430-5589 (USA) |
| Dr. Lisa Abuogi | University of Colorado, Denver | +1-303-724-7430 (USA) |
| Dr. Zachary Kwena | Kenya Medical Research Institute | +254 722-386 714 |
| Starley Shade | University of California, San Francisco | +1-415-794-4354 (USA) |
| Lina Montoya | University of Notrh Carolina at Chapel Hill | +1-4105857470 (USA) |

**Study Location:** Kisumu County

**Introduction**

I am [name of RA/APL], from Kenya Medical Research Institute (KEMRI). We are conducting a research study titled **An Adaptive Strategy for Preventing and Treating Lapses of Retention in HIV Care for Adolescents (A4A).** This study is being carried out by KEMRI and Washington University in St. Louis, University of Colorado in Denver, and University of California in Berkeley and San Francisco in the United States. Making sure that adolescents and youth living with HIV stay in HIV care and get their viral load to zero is very important to staying healthy. In this study, we are trying to learn how to give adolescents and young adults interventions that will help them stay in care and stay at zero viral load.

We are requesting you to participate in 30 minutes survey to help design the conditional cash transfer (CCT) intervention for this study. The purpose of this survey is to determine adolescent and young adults (AYA) preferences on incentives during HIV care treatment.

During the survey, the Research Assistant will read out the questions and show you a card which demonstrates to you the preferred options in each of the questions. You will choose your preference and he/she will enter this information in the study tablet. As an adolescent or young adult in this community, we would like to know your preferences on incentives to help us develop the best intervention strategies for adolescent and young adults.

Your participation is voluntary. If you do not wish to participate, you may stop at any time and there will be no penalty to you. You will not lose any of your regular benefits, and you can still get your care from your clinic the way you usually do. Responses will be completely anonymous; your name will not appear anywhere in the final write up.

Do you have any questions about the study?

If you would like a copy of this letter for your records, please let me know and I will [give you a copy now; email, mail, or fax it to you, etc.].

If you have any additional questions regarding the research, contact Dr. Elizabeth Ann Bukusi, Kenya Medical Research Institute, 0733617503 or Dr. Elvin Geng, Washington University, + 14154305589. If you have any questions regarding your rights as a research subject, please contact the Scientific and Ethics Review Committee of Kenya Medical Research Institute at 0717719477 or [seru@kemri.org](mailto:seru@kemri.org) OR Washington University Human Subjects Protection Program at +13147476800.

**Verbal Consent**

PARTICIPATION IN RESEARCH IS VOLUNTARY. You have the right to decline to be in this study, or to withdraw from it at any point without penalty or loss of benefits to which you are otherwise entitled.

Consents to study participation Does not consent to study participation

[Grab your reader’s attention with a great quote from the document or use this space to emphasize a key point. To place this text box anywhere on the page, just drag it.]

[Grab your reader’s attention with a great quote from the document or use this space to emphasize a key point. To place this text box anywhere on the page, just drag it.]

[Grab your reader’s attention with a great quote from the document or use this space to emphasize a key point. To place this text box anywhere on the page, just drag it.]

___________________________________________________________________

Printed Name of Person Obtaining Assent	Title

___________________________________________________________________

Signature of Person Obtaining Assent	Date

***Adaptive Strategies to Prevent and Treat Failures of Retention in***

***Adolescent HIV Care (A4A Study)***

**ENGLISH A4A VERBAL CONSENT SCRIPT FOR DISCRETE CHOICE EXPERIMENT (DCE)18 YEARS OLD AND ABOVE**

INVESTIGATORS AND INSTITUTIONAL AFFILIATIONS:

| Researcher | Institution | Contact |
| --- | --- | --- |
| Dr. Elizabeth Bukusi | Kenya Medical Research Institute | +254 733-617 503 |
| Dr. Elvin Geng | Washington University, Missouri | +1-415-430-5589 (USA) |
| Dr. Lisa Abuogi | University of Colorado, Denver | +1-303-724-7430 (USA) |
| Dr. Zachary Kwena | Kenya Medical Research Institute | +254 722-386 714 |
| Starley Shade | University of California, San Francisco | +1-415-794-4354 (USA) |
| Lina Montoya | University of Notrh Carolina at Chapel Hill | +1-4105857470 (USA) |

**Study Location:** Kisumu County

**Introduction**

I am [name of RA/APL], from Kenya Medical Research Institute (KEMRI). We are conducting a research study titled **An Adaptive Strategy for Preventing and Treating Lapses of Retention in HIV Care for Adolescents (A4A).** This study is being carried out by KEMRI and Washington University in St. Louis, University of Colorado in Denver, and University of California in Berkeley and San Francisco in the United States. Making sure that adolescents and youth living with HIV stay in HIV care and get their viral load to zero is very important to staying healthy. In this study, we are trying to learn how to give adolescents and young adults interventions that will help them stay in care and stay at zero viral load. We are requesting you to participate in 30 minutes survey to help design the conditional cash transfer (CCT) intervention for this study. The purpose of this survey is to determine adolescent and young adults (AYA) preferences on incentives during HIV care treatment.

During the survey, the Research Assistant will read out the questions and show you a card which demonstrates to you the preferred options in each of the questions. You will choose your preference and he/she will enter this information in the study tablet. As an adolescent or young adult in this community, we would like to know your preferences on incentives to help us develop the best intervention strategies for adolescent and young adults.

Your participation is voluntary. If you do not wish to participate, you may stop at any time and there will be no penalty to you. You will not lose any of your regular benefits, and you can still get your care from your clinic the way you usually do. Responses will be completely anonymous; your name will not appear anywhere in the final write up.

Do you have any questions about the study?

If you would like a copy of this letter for your records, please let me know and I will [give you a copy now; email, mail, or fax it to you, etc.].

If you have any additional questions regarding the research, contact Dr. Elizabeth Ann Bukusi, Kenya Medical Research Institute, 0733617503 or Dr. Elvin Geng, Washington University, + 14154305589. If you have any questions regarding your rights as a research subject, please contact the Scientific and Ethics Review Committee of Kenya Medical Research Institute at 0717719477 or [seru@kemri.org](mailto:seru@kemri.org) OR Washington University Human Subjects Protection Program at +13147476800.

**Verbal Consent**

PARTICIPATION IN RESEARCH IS VOLUNTARY. You have the right to decline to be in this study, or to withdraw from it at any point without penalty or loss of benefits to which you are otherwise entitled.

Consents to study participation Does not consent to study participation

[Grab your reader’s attention with a great quote from the document or use this space to emphasize a key point. To place this text box anywhere on the page, just drag it.]

[Grab your reader’s attention with a great quote from the document or use this space to emphasize a key point. To place this text box anywhere on the page, just drag it.]

[Grab your reader’s attention with a great quote from the document or use this space to emphasize a key point. To place this text box anywhere on the page, just drag it.]

___________________________________________________________________

Printed Name of Person Obtaining Consent	Title

___________________________________________________________________

Signature of Person Obtaining Consent	Date

***Adaptive Strategies to Prevent and Treat Failures of Retention in***

**Adolescent HIV Care (A4A Study)**

ENGLISH A4A PARENTAL VERBAL CONSENT SCRIPT FOR DISCRETE CHOICE EXPERIMENT (DCE)14-17 YEARS OLD

INVESTIGATORS AND INSTITUTIONAL AFFILIATIONS:

| Researcher | Institution | Contact |
| --- | --- | --- |
| Dr. Elizabeth Bukusi | Kenya Medical Research Institute | +254 733-617 503 |
| Dr. Elvin Geng | Washington University, Missouri | +1-415-430-5589 (USA) |
| Dr. Lisa Abuogi | University of Colorado, Denver | +1-303-724-7430 (USA) |
| Eliud Akama | Kenya Medical Research Institute | +254 728-590 376 |
| Dr. Zachary Kwena | Kenya Medical Research Institute | +254 722-386 714 |
| Starley Shade | University of California, San Francisco | +1-415-794-4354 (USA) |
| Lina Montoya | University of Notrh Carolina at Chapel Hill | +1-4105857470 (USA) |

**Study Location:** Kisumu County

**Introduction**

I am [name of RA/APL], from Kenya Medical Research Institute (KEMRI). We are conducting a research study titled **An Adaptive Strategy for Preventing and Treating Lapses of Retention in HIV Care for Adolescents (A4A).** This study is being carried out by KEMRI and Washington University in St. Louis, University of Colorado in Denver, and University of California in Berkeley and San Francisco in the United States. Making sure that adolescents and youth living with HIV stay in HIV care and get their viral load to zero is very important to staying healthy. In this study, we are trying to learn how to give adolescents and young adults interventions that will help them stay in care and stay at zero viral load.

We are requesting you toallow your child to participate in 30 minutes survey to help design the conditional cash transfer (CCT) intervention for this study. The purpose of this survey is to determine adolescent and young adults (AYA) preferences on incentives during HIV care treatment.

During the survey, the Research Assistant will read out the questions to your child and show him/her a card which demonstrates the preferred options in each of the questions. He/she will choose their preference and the Research Assistant will enter this information in the study tablet. As an adolescent or young adult in this community, we would like to know his/her preferences on incentives to help us develop the best intervention strategies for adolescent and young adults.

Your child’s participation is voluntary. If your child does not wish to participate, he/she may stop at any time and there will be no penalty to you.Your child will not lose any of the regular benefits, and can still get care from the clinic the way he/she usually does. Responses will be completely anonymous; your name will not appear anywhere in the final write up.

Do you have any questions about the study?

If you would like a copy of this letter for your records, please let me know and I will [give you a copy now; email, mail, or fax it to you, etc.].

If you have any additional questions regarding the research, contact Dr. Elizabeth Ann Bukusi, Kenya Medical Research Institute, 0733617503 or Dr. Elvin Geng, Washington University, + 14154305589. If you have any questions regarding your rights as a research subject, please contact the Scientific and Ethics Review Committee of Kenya Medical Research Institute at 0717719477 or [seru@kemri.org](mailto:seru@kemri.org) OR Washington University Human Subjects Protection Program at +13147476800.

**Verbal Consent**

PARTICIPATION IN RESEARCH IS VOLUNTARY. You have the right to decline to be in this study, or to withdraw from it at any point without penalty or loss of benefits to which you are otherwise entitled.

Consents to study participation Does not consent to study participation

[Grab your reader’s attention with a great quote from the document or use this space to emphasize a key point. To place this text box anywhere on the page, just drag it.]

[Grab your reader’s attention with a great quote from the document or use this space to emphasize a key point. To place this text box anywhere on the page, just drag it.]

[Grab your reader’s attention with a great quote from the document or use this space to emphasize a key point. To place this text box anywhere on the page, just drag it.]

___________________________________________________________________

Printed Name of Person Obtaining Consent	Title

___________________________________________________________________

Signature of Person Obtaining Consent	Date

***Adaptive Strategies to Prevent and Treat Failures of Retention in***

***Adolescent HIV Care (A4A Study)***

ENGLISH A4A VERBAL CONSENT SCRIPT FOR DESIGN WORKSHOP*18 – 24 YEARS OLD*

INVESTIGATORS AND INSTITUTIONAL AFFILIATIONS:

| Researcher | Institution | Contact |
| --- | --- | --- |
| Dr. Elizabeth Bukusi | Kenya Medical Research Institute | +254 733-617 503 |
| Eliud Akama | Kenya Medical Research Institute | +254 728-590 376 |
| Dr. Elvin Geng | Washington University, Missouri | +1-415-430-5589 (USA) |
| Dr. Lisa Abuogi | University of Colorado, Denver | +1-303-724-7430 (USA) |
| Dr. Zachary Kwena | Kenya Medical Research Institute | +254 722-386 714 |
| Starley Shade | University of California, San Francisco | +1-415-794-4354 (USA) |
| Lina Montoya | University of Notrh Carolina at Chapel Hill | +1-4105857470 (USA) |

**Study Location:** Kisumu County

**Introduction**

I am [name of RA], from Kenya Medical Research Institute (KEMRI). We are conducting a research study titled **An Adaptive Strategy for Preventing and Treating Lapses of Retention in HIV Care for Adolescents (A4A).** This study is being carried out by KEMRI and Washington University in St. Louis, University of Colorado in Denver, and University of California in Berkeley and San Francisco in the United States. Making sure that adolescents and youth living with HIV stay in HIV care and get their viral load to zero is very important to staying healthy. In this study, we are trying to learn how to give adolescents and young adults interventions that will help them stay in care and stay at zero viral load.

We are requesting you to participate in a one week workshop to help design the interventions for this study. The workshop will have sessions that focus on thinking through and improving the intervention strategies planned for this study.

During this workshop, we will first provide you with an overview of the study and then have sessions to discuss and design intervention plans. As an adolescent or young adult in this community, we would like your views and ideas to help us develop the best intervention strategies for adolescent and young adults. For example, deciding SMS wording and how peer navigators can best support adolescents and young adults by phone and in person.

Your participation is voluntary. If you do not wish to participate, you may stop at any time and there will be no penalty to you. You will not lose any of your regular benefits, and you can still get your care from your clinic the way you usually do. Responses will be completely anonymous; your name will not appear anywhere in the final write up.

There are minimal risks associated with participating in the one-week workshop. During the one-week workshop, we will not be able to guarantee confidentiality because we will be discussing information as a group. Therefore, if you would feel uncomfortable with any of your statements being shared with others in or outside the group, please do not share them during the process.

Do you have any questions about the study?

If you would like a copy of this letter for your records, please let me know and I will [give you a copy now; email, mail, or fax it to you, etc.].

If you have any additional questions regarding the research, contact Dr. Elizabeth Ann Bukusi, Kenya Medical Research Institute, 0733617503 or Dr. Elvin Geng, Washington University, + 14154305589. If you have any questions regarding your rights as a research subject, please contact the Scientific and Ethics Review Committee of Kenya Medical Research Institute at 0717719477 or [seru@kemri.org](mailto:seru@kemri.org) OR Washington University Human Subjects Protection Program at +13147476800.

**Verbal Consent**

PARTICIPATION IN RESEARCH IS VOLUNTARY. You have the right to decline to be in this study, or to withdraw from it at any point without penalty or loss of benefits to which you are otherwise entitled.

Consents to study participation Does not consent to study participation

[Grab your reader’s attention with a great quote from the document or use this space to emphasize a key point. To place this text box anywhere on the page, just drag it.]

[Grab your reader’s attention with a great quote from the document or use this space to emphasize a key point. To place this text box anywhere on the page, just drag it.]

[Grab your reader’s attention with a great quote from the document or use this space to emphasize a key point. To place this text box anywhere on the page, just drag it.]

___________________________________________________________________

Printed Name of Person Obtaining Consent	Title

___________________________________________________________________

Signature of Person Obtaining Consent	Date

***Adaptive Strategies to Prevent and Treat Failures of Retention in***

***Adolescent HIV Care (A4A Study)***

**ENGLISH A4A VERBAL PARENTAL CONSENT SCRIPT FOR DESIGN WORKSHOP*FOR PARENTS OF 14 – 17 YEAR OLDS***

INVESTIGATORS AND INSTITUTIONAL AFFILIATIONS:

| Researcher | Institution | Contact |
| --- | --- | --- |
| Dr. Elizabeth Bukusi | Kenya Medical Research Institute | +254 733-617 503 |
| Eliud Akama | Kenya Medical Research Institute | +254 728-590 376 |
| Dr. Elvin Geng | Washington University, Missouri | +1-415-430-5589 (USA) |
| Dr. Lisa Abuogi | University of Colorado, Denver | +1-303-724-7430 (USA) |
| Dr. Zachary Kwena | Kenya Medical Research Institute | +254 722-386 714 |
| Starley Shade | University of California, San Francisco | +1-415 794-4354 (USA) |
| Lina Montoya | University of Notrh Carolina at Chapel Hill | +1-4105857470 (USA) |

**Study Location:** Kisumu County

**Introduction**

I am [name of RA], from Kenya Medical Research Institute (KEMRI). We are conducting a research study titled **An Adaptive Strategy for Preventing and Treating Lapses of Retention in HIV Care for Adolescents (A4A).** This study is being carried out by KEMRI and Washington University in St. Louis, University of Colorado in Denver, and University of California in Berkeley and San Francisco in the United States. Making sure that adolescents and youth living with HIV stay in HIV care and get their viral load to zero is very important to staying healthy. In this study, we are trying to learn how to give adolescents and young adults interventions that will help them stay in care and stay at zero viral load.

We are requesting you to allow your child to participate in a one week workshop to help design the interventions for this study. The workshop will have sessions that focus on thinking through and improving the intervention strategies planned for this study.

During this workshop, we will first provide him/her with an overview of the study and then have sessions to discuss and design intervention plans. As an adolescent or young adult in this community, we would like his/her views and ideas to help us develop the best intervention strategies for adolescent and young adults. For example, deciding SMS wording and how peer navigators can best support adolescents and young adults by phone and in person.

Your child’s participation is voluntary. If he/she does not wish to participate, he/she may stop at any time and there will be no penalty. Your child will not lose any of the regular benefits, and he/she can still get care from the clinic the way he/she usually does. Responses will be completely anonymous; your name will not appear anywhere in the final write up.

There are minimal risks associated with participating in the one-week workshop. During the one-week workshop, we will not be able to guarantee confidentiality because we will be discussing information as a group. Therefore, if your child would feel uncomfortable with any of your statements being shared with others in or outside the group, please do not allow him/her to share them during the process.

Do you have any questions about the study?

If you would like a copy of this letter for your records, please let me know and I will [give you a copy now; email, mail, or fax it to you, etc.].

If you have any additional questions regarding the research, contact Dr. Elizabeth Ann Bukusi, Kenya Medical Research Institute, 0733617503 or Dr. Elvin Geng, Washington University, + 14154305589. If you have any questions regarding your rights as a research subject, please contact the Scientific and Ethics Review Committee of Kenya Medical Research Institute at 0717719477 or [seru@kemri.org](mailto:seru@kemri.org) OR Washington University Human Subjects Protection Program at +13147476800.

**Verbal Consent**

PARTICIPATION IN RESEARCH IS VOLUNTARY. You have the right to decline to be in this study, or to withdraw from it at any point without penalty or loss of benefits to which you are otherwise entitled.

Consents to study participation Does not consent to study participation

[Grab your reader’s attention with a great quote from the document or use this space to emphasize a key point. To place this text box anywhere on the page, just drag it.]

[Grab your reader’s attention with a great quote from the document or use this space to emphasize a key point. To place this text box anywhere on the page, just drag it.]

[Grab your reader’s attention with a great quote from the document or use this space to emphasize a key point. To place this text box anywhere on the page, just drag it.]

___________________________________________________________________

Printed Name of Person Obtaining Consent	Title

___________________________________________________________________

Signature of Person Obtaining Consent	Date

***Adaptive Strategies to Prevent and Treat Failures of Retention in***

***Adolescent HIV Care (A4A Study)***

ENGLISH A4A VERBAL ASSENT SCRIPT FOR DESIGN WORKSHOP14 – 17 YEARS OLD

INVESTIGATORS AND INSTITUTIONAL AFFILIATIONS:

| Researcher | Institution | Contact |
| --- | --- | --- |
| Dr. Elizabeth Bukusi | Kenya Medical Research Institute | +254 733-617 503 |
| Dr. Thomas Odeny | Kenya Medical Research Institute | +254 722-819 835 |
| Dr. Elvin Geng | Washington University, Missouri | +1-415-430-5589 (USA) |
| Dr. Lisa Abuogi | University of Colorado, Denver | +1-303-724-7430 (USA) |
| Dr. Zachary Kwena | Kenya Medical Research Institute | +254 722-386 714 |
| Starley Shade | University of California, San Francisco | +1-415-794-4354 (USA) |
| Lina Montoya | University of Notrh Carolina at Chapel Hill | +1-4105857470 (USA) |
| Maya Petersen | University of California, Berkeley | +1-510-643-0563 (USA) |

**Study Location:** Kisumu County

**Introduction**

I am [name of RA], from Kenya Medical Research Institute (KEMRI). We are conducting a research study titled **An Adaptive Strategy for Preventing and Treating Lapses of Retention in HIV Care for Adolescents (A4A).** This study is being carried out by KEMRI and Washington University in St. Louis, University of Colorado in Denver, and University of California in Berkeley and San Francisco in the United States. Making sure that adolescents and youth living with HIV stay in HIV care and get their viral load to zero is very important to staying healthy. In this study, we are trying to learn how to give adolescents and young adults interventions that will help them stay in care and stay at zero viral load.

We are requesting you to participate in a one-week workshop to help design the interventions for this study. The workshop will have sessions that focus on thinking through and improving the intervention strategies planned for this study.

During this workshop, we will first provide you with an overview of the study and then have sessions to discuss and design intervention plans. As an adolescent or young adult in this community, we would like your views and ideas to help us develop the best intervention strategies for adolescent and young adults. For example, deciding SMS wording and how peer navigators can best support adolescents and young adults by phone and in person.

Your participation is voluntary. If you do not wish to participate, you may stop at any time and there will be no penalty to you. You will not lose any of your regular benefits, and you can still get your care from your clinic the way you usually do. Responses will be completely anonymous; your name will not appear anywhere in the final write up.

There are minimal risks associated with participating in the one-week workshop. During the one-week workshop, we will not be able to guarantee confidentiality because we will be discussing information as a group. Therefore, if you would feel uncomfortable with any of your statements being shared with others in or outside the group, please do not share them during the process.

Do you have any questions about the study?

If you would like a copy of this letter for your records, please let me know and I will [give you a copy now; email, mail, or fax it to you, etc.].

If you have any additional questions regarding the research, contact Dr. Elizabeth Ann Bukusi, Kenya Medical Research Institute, 0733617503 or Dr. Elvin Geng, Washington University, + 14154305589. If you have any questions regarding your rights as a research subject, please contact the Scientific and Ethics Review Committee of Kenya Medical Research Institute at 0717719477 or [seru@kemri.org](mailto:seru@kemri.org) OR Washington University Human Subjects Protection Program at +13147476800.

Consents to study participation Does not consent to study participation

[Grab your reader’s attention with a great quote from the document or use this space to emphasize a key point. To place this text box anywhere on the page, just drag it.]

[Grab your reader’s attention with a great quote from the document or use this space to emphasize a key point. To place this text box anywhere on the page, just drag it.]

[Grab your reader’s attention with a great quote from the document or use this space to emphasize a key point. To place this text box anywhere on the page, just drag it.]

___________________________________________________________________

Printed Name of Person Obtaining Consent	Title

___________________________________________________________________

Signature of Person Obtaining Consent	Date

**ENGLISH ASSENT FORM (FGD) 14-17 YEARS OLD**

**KENYA MEDICAL RESEARCH INSTITUTE**

**WASHINGTON UNIVERSITY, ST. LOUIS**

ASSENT TO PARTICIPATE IN FOCUS GROUP DISCUSSION (FGD)

**Study Title:** *Adaptive Strategies to Prevent and Treat Failures of Retention in Adolescent HIV Care (A4A Study)*

INVESTIGATORS AND INSTITUTIONAL AFFILIATIONS:

| Researcher | Institution | Contact |
| --- | --- | --- |
| Dr. Elizabeth Bukusi | Kenya Medical Research Institute | +254 733-617 503 |
| Eliud Akama | Kenya Medical Research Institute | +254 728-590 376 |
| Dr. Elvin Geng | Washington University, St. Louis | +1-415-430-5589 (USA) |
| Dr. Lisa Abuogi | University of Colorado, Denver | +1-303-724-7430 (USA) |
| Dr. Zachary Kwena | Kenya Medical Research Institute | +254 722-386 714 |
| Starley Shade | University of California, San Francisco | +1-415-794-4354 (USA) |
| Lina Montoya | University of Notrh Carolina at Chapel Hill | +1-4105857470 (USA) |

**Study Location:** Kisumu County

We want to tell you about something we are doing called a research study. A research study is when doctors collect a lot of information to learn more about something. We are asking you to volunteer to take part in a focus group discussion which is part of a research study we are doing. The box below tells you important things you should think about before deciding to participate in this group discussion. We will provide more information below the box. Please ask questions about any of the information before you decide whether to participate or not. You may also wish to talk to others (for example, your family, friends, or the researchers) about this study, before agreeing to join.

- **Voluntary Consent**. You are being asked to volunteer to participate in a focus group discussion. It is up to you whether you choose to participate or not. There are no penalties and you will not lose anything if you decide not to join or if after you join, you decide to quit.
- **Purpose**. We are conducting three focus group discussions to gather adolescent and young adult thoughts about intervention strategies planned to help adolescents and young adults stay involved in HIV care. The planned interventions involve electronic and in-person peer navigation (similar to counseling) and cash transfers. Your views and information will help inform a special meeting about the interventions called a design workshop. You may be contacted about participating in the design workshop at a later stage.
- **Participants:** About 24 adolescents and young adults, 14 – 24 years old, and living with HIV in Kisumu county will participate in the focus group discussions. There will be 3 groups with about 8 in each group.
- **Procedures and Activities.** First**,** we will ask your parent or caregiver if he/she agrees with you participating. If he/she does not, then you may not participate. If you both agree, we will set up a time for you to join a focus group discussion. The discussion will focus on one of the three planned intervention strategies to help keep adolescents and young adults engaged in HIV care. Each focus group discussion will be conducted by a trained interviewer in a private place to protect your confidentiality. In addition, we will ask your permission to audio record the discussion, which we will then write down and review. Any information that could potentially identify you will not be included in the review. We expect the focus group discussion to last one and half hours.
- **Risks.** There is a small chance of accidental disclosure of HIV status. However, we will put safety measures in place to protect information about your HIV status.
- **Benefits**. You may not receive any direct benefits from participating in this study. By participating in this study you may contribute to efforts to promote the health of adolescents living with HIV.
- **Alternatives.** You are free to choose not to participate in the discussion. If you decide not to take part in this study, there will be no penalty to you. You will not lose any of your regular benefits, and you can still get your care from your clinic the way you usually do.

**Procedures and Activities**

In the event your parent/caregiver is not present during this recruitment, the Research Assistant will contact your parent/caregiver to consent over the phone.

If your parent or caregiver and you agree to participate, these things will happen:

1. We will ask you to sign or make a mark on this form as a sign that you have voluntarily accepted to participate in this research.
2. We will set up time with you to join a group of other adolescents and young adults to discuss how best to implement the planned intervention strategies.
3. You will be part of a discussion focused on one of three planned intervention strategies, either electronic navigation (automated SMS messages and peer support through mobile phones), in-person peer navigation support, or conditional cash transfer (receiving money).
4. You will be asked questions about experiences and challenges with HIV care, how often and what type of support is needed, and suggestions for how to best carry out the planned interventions.
5. Your views and information will help inform an intervention meeting called a design workshop.
6. The discussion led by a trained interviewer in private room to protect your confidentiality.
7. We will also ask you to allow us to record the interview and your answers so that we can write it down later and review.
8. Any information that could potentially identify you will not be included in the review.
9. The discussion is expected to take about one and a half hours.
10. You may be asked at a later date to participate in that one-week workshop. Let us know if you will be willing to participate.

**Risks**

There is a small chance of accidental disclosure of HIV status. However, we will put safety measures in place to protect information about your HIV status.

**Benefits**

You may not receive any direct benefits from participating in this study. However, your participation may help improve retention of adolescents in care.

**Reimbursement**

You will be given a transport reimbursement of Kshs. 500/=.

**Alternatives**

You are free to choose not to participate in the study. If you decide not to take part in this study, there will be no penalty to you. You will not lose any of your regular benefits, and you can still get your care from your clinic the way you usually do.

**Confidentiality**

We will do our best to ensure that the personal information gathered for this study is kept private. However, we cannot guarantee total privacy. Your personal information may be given out if required by law. If information from this study is published or presented at scientific meetings, your name and other personal information will not be used.

Government or university staff sometimes review studies such as this one to make sure they are being done safely and legally. If a review of this study takes place, your records may be examined. The reviewers will protect your privacy. The study records will not be used to put you at legal risk of harm. Organizations that may look at and/or copy your research records for research, quality assurance, and data analysis include:

- The Washington University, St. Louis
- The Kenya Medical Research Institute
- University of California San Francisco
- University of Colorado, Denver
- University of California, Berkeley

Participation in research may involve a loss of privacy, however we will put safety measures in place to protect your confidentiality. Any additional information about the study will be provided to you including the final study results.

**Participation**

You can decide to stop the interview at any time, just tell the study staff and they will stop you. In addition, the study staff can stop from taking part, if they feel it is in your best interest. You will not be penalized in any way should you choose to stop participating.

**Sponsorship**

The study is sponsored by the National Institutes of Health. None of the investigators have any financial conflict of interests. In situations where the study may be terminated at the discretion of the investigator or study sponsor, you will be informed the next course of action.

**Contact**

You can talk to the researcher(s) about any questions, concerns, or complaints you have about this study. Contact our study staff at 0733-819 835. If you wish to ask questions about the study or your rights as a research participant to someone other than the researchers or if you wish to voice any problems or concerns you may have about the study, you may contact Secretary of the Ethical Review Committee, Kenya Medical Research Institute at seru@kemri.org or P.O. BOX 54840-00200 Tel. 020-2722541, 0717719477. You may also contact the Washington University Human Research Protection Office at +1-314-747-6800. These committees are concerned with the protection of volunteers in research projects.

**CONSENT**

You have been given a copy of this consent form to keep.

**PARTICIPATION IN RESEARCH IS VOLUNTARY.** You have the right to decline to be in this study, or to withdraw from it at any point without penalty or loss of benefits to which you are otherwise entitled.

**Participant’s Statement**

The study described above has been explained to me. I volunteer to take part in this research. I have had a chance to ask questions. If I have future questions about the research, I can ask one of the investigators listed above. If I have questions about my rights as a research participant, I can contact those listed above.

I agree for my information to be used for future research and shared with other researchers without my additional consent with/without identifiers.

If you wish to participate in this study, you should sign below.

Date	Participant's Name and Signature for Consent

Date	Person Obtaining Consent (Name and signature)

***If illiterate***

I have witnessed the accurate reading of the consent form to the participant and the individual has had the opportunity to ask questions. I confirm that the individual has given consent freely**.**

Name of witness____________________ AND

Signature of witness ***____________****_____*AND

Name of participant__________________AND

**Thumbprint of participant**

**Date ________________________**

**Verbal Consent**

PARTICIPATION IN RESEARCH IS VOLUNTARY. You have the right to decline to be in this study, or to withdraw from it at any point without penalty or loss of benefits to which you are otherwise entitled.

Consents to study participation Does not consent to study participation

[Grab your reader’s attention with a great quote from the document or use this space to emphasize a key point. To place this text box anywhere on the page, just drag it.]

[Grab your reader’s attention with a great quote from the document or use this space to emphasize a key point. To place this text box anywhere on the page, just drag it.]

[Grab your reader’s attention with a great quote from the document or use this space to emphasize a key point. To place this text box anywhere on the page, just drag it.]

___________________________________________________________________

Printed Name of Participant	Date

___________________________________________________________________

Signature of Person Obtaining Assent	Title

**Permission to audio-record discussion**

I give permission for the group discussion in which I will participate to be recorded: Yes ☐	No ☐

**Permission to be contacted for a** week’s **workshop**

I accept to be contacted to participate in a week’s workshop: Yes ☐	No ☐

Copies to:	1. Investigators

2. Study participant

**ENGLISH CONSENT FORM (FGD) 18-24 YEARS OLD**

**KENYA MEDICAL RESEARCH INSTITUTE**

**WASHINGTON UNIVERSITY, ST. LOUIS**

**CONSENT TO PARTICIPATE IN FOCUS GROUP DISCUSSION (FGD)**

**Study Title:** *Adaptive Strategies to Prevent and Treat Failures of Retention in Adolescent HIV Care (A4A Study)*

INVESTIGATORS AND INSTITUTIONAL AFFILIATIONS:

| Researcher | Institution | Contact |
| --- | --- | --- |
| Dr. Elizabeth Bukusi | Kenya Medical Research Institute | +254 733-617 503 |
| Eliud Akama | Kenya Medical Research Institute | +254 728-590 376 |
| Dr. Elvin Geng | Washington University, Missouri | +1-415-430-5589 (USA) |
| Dr. Lisa Abuogi | University of Colorado, Denver | +1-303-724-7430 (USA) |
| Dr. Zachary Kwena | Kenya Medical Research Institute | +254 722-386 714 |
| Starley Shade | University of California, San Francisco | +1-415-794-4354 (USA) |
| Lina Montoya | University of Notrh Carolina at Chapel Hill | +1-4105857470 (USA) |

**Study Location:** Kisumu County

You are being asked to volunteer to take part in a focus group discussion. The box below tells you important things you should think about before deciding to participate in this group discussion. We will provide more detailed information below the box. Please ask questions about any of the information before you decide whether to participate or not. You may also wish to talk to others (for example, your family, friends, or the researchers) about this study, before agreeing to join.

- **Voluntary Consent**. You are being asked to volunteer to participate in a focus group discussion. It is up to you whether you choose to participate or not. There are no penalties and you will not lose anything if you decide not to join or if after you join, you decide to quit.
- **Purpose**. We are conducting three focus group discussions to gather adolescent and young adult thoughts about intervention strategies planned to help adolescents and young adults stay involved in HIV care. The planned interventions involve electronic and in-person peer navigation (similar to counseling) and cash transfers. Your views and information will help inform a special meeting about the interventions called a design workshop. You may be contacted about participating in the design workshop at a later stage.
- **Participants:** About 24 adolescents and young adults, 14 – 24 years old, and living with HIV in Kisumu county will participate in the focus group discussions. There will be 3 groups with about 8 in each group.
- **Procedures and Activities.** If you agree to participate, we will set up a time for you to join a focus group discussion. The discussion will focus on one of the three planned intervention strategies to help keep adolescents and young adults engaged in HIV care. Each focus group discussion will be conducted by a trained interviewer in a private place to protect your confidentiality. In addition, we will ask your permission to audio record the discussion, which we will then write down and review. Any information that could potential identify you will not be included in the review. We expect the focus group discussion to last one and half hours.
- **Risks.** There is a small chance of accidental disclosure of HIV status. However, we will put safety measures in place to protect information about your HIV status.
- **Benefits**. You may not receive any direct benefits from participating in this study. By participating in this study you may contribute to efforts to promote the health of adolescents living with HIV.
- **Alternatives.** You are free to choose not to participate in the discussion. If you decide not to take part in this study, there will be no penalty to you. You will not lose any of your regular benefits, and you can still get your care from your clinic the way you usually do.

**Procedures and Activities**

If you agree, the following things will happen:

1. We will ask you to sign or make a mark on this form as a sign that you have voluntarily accepted to participate in this research.
2. We will set up a time with you to join a group of other adolescents and young adults to discuss how best to carry out the planned intervention strategies.
3. You will be part of a discussion focused on one of three planned intervention strategies, either electronic navigation (automated SMS messages and peer support through mobile phones), in-person peer navigation support, or conditional cash transfer (receiving money).
4. You will be asked questions about experiences and challenges with HIV care, how often and what type of support is needed, and suggestions for how to best carry out the planned interventions.
5. Your views and information will help inform an intervention meeting called a design workshop.
6. The discussion will led by a trained interviewer in private room to protect your confidentiality.
7. We will also ask you to allow us to record the interview and your answers so that we can write it down later and review.
8. Any information that could potentially identify you will not be included in the review.
9. The discussion is expected to take about one and a half hours.
10. You may be asked at a later date to participate in that one-week workshop. Let us know if you will be willing to participate.

**Risks**

There is a small chance of accidental disclosure of HIV status. However, we will put safety measures in place to protect information about your HIV status.

**Benefits**

You may not receive any direct benefits from participating in this study. However, your participation may help improve retention of adolescents in care.

**Reimbursement**

You will be given a transport reimbursement of Kshs. 500/=.

**Alternatives**

You are free to choose not to participate in the study. If you decide not to take part in this study, there will be no penalty to you. You will not lose any of your regular benefits, and you can still get your care from your clinic the way you usually do.

**Confidentiality**

We will do our best to ensure that the personal information gathered for this study is kept private. However, we cannot guarantee total privacy. Your personal information may be given out if required by law. If information from this study is published or presented at scientific meetings, your name and other personal information will not be used.

Government or university staff sometimes review studies such as this one to make sure they are being done safely and legally. If a review of this study takes place, your records may be examined. The reviewers will protect your privacy. The study records will not be used to put you at legal risk of harm. Organizations that may look at and/or copy your research records for research, quality assurance, and data analysis include:

- The Washington University, St. Louis
- The Kenya Medical Research Institute
- University of California San Francisco
- University of Colorado, Denver
- University of California, Berkeley

Participation in research may involve a loss of privacy, however we will put safety measures in place to protect your confidentiality. Any additional information about the study will be provided to you including the final study results.

**Participation**

You can decide to stop the interview at any time, just tell the study staff and they will stop you. In addition, the study staff can stop from taking part, if they feel it is in your best interest. You will not be penalized in any way should you choose to stop participating.

**Sponsorship**

The study is sponsored by the National Institutes of Health. None of the investigators have any financial conflict of interests. In situations where the study may be terminated at the discretion of the investigator or study sponsor, you will be informed the next course of action.

**Contact**

You can talk to the researcher(s) about any questions, concerns, or complaints you have about this study. Contact our study staff at 0733-819 835. If you wish to ask questions about the study or your rights as a research participant to someone other than the researchers or if you wish to voice any problems or concerns you may have about the study, you may contact Secretary of the Ethical Review Committee, Kenya Medical Research Institute at seru@kemri.org or P.O. BOX 54840-00200 Tel. 020-2722541, 0717719477. You may also contact the Washington University Human Research Protection Office at +1-314-747-6800.These committees are concerned with the protection of volunteers in research projects.

**CONSENT**

You have been given a copy of this consent form to keep.

**PARTICIPATION IN RESEARCH IS VOLUNTARY.** You have the right to decline to be in this study, or to withdraw from it at any point without penalty or loss of benefits to which you are otherwise entitled.

**Participant’s Statement**

The study described above has been explained to me. I volunteer to take part in this research. I have had a chance to ask questions. If I have future questions about the research, I can ask one of the investigators listed above. If I have questions about my rights as a research participant, I can contact those listed above.

I agree for my information to be used for future research and shared with other researchers without my additional consent with/without identifiers.

If you wish to participate in this study, you should sign below.

Date	Participant's Name and Signature for Consent

Date	Person Obtaining Consent (Name and signature)

***If illiterate***

I have witnessed the accurate reading of the consent form to the participant and the individual has had the opportunity to ask questions. I confirm that the individual has given consent freely**.**

Name of witness_____________________ AND

Signature of witness *__________________*AND

Name of participant __________________AND

**Thumbprint of participant**

**Date ________________________**

**Verbal Consent**

PARTICIPATION IN RESEARCH IS VOLUNTARY. You have the right to decline to be in this study, or to withdraw from it at any point without penalty or loss of benefits to which you are otherwise entitled.

Consents to study participation Does not consent to study participation

[Grab your reader’s attention with a great quote from the document or use this space to emphasize a key point. To place this text box anywhere on the page, just drag it.]

[Grab your reader’s attention with a great quote from the document or use this space to emphasize a key point. To place this text box anywhere on the page, just drag it.]

[Grab your reader’s attention with a great quote from the document or use this space to emphasize a key point. To place this text box anywhere on the page, just drag it.]

___________________________________________________________________

Printed Name of Participant	Date

___________________________________________________________________

Signature of Person Obtaining Consent	Title

**Permission to audio-record discussion**

I give permission for the group discussion in which I will participate to be recorded: Yes ☐	No ☐

**Permission to be contacted for a** week’s **workshop**

I accept to be contacted to participate in a **week’s** workshop: Yes ☐	No ☐

Copies to:	1. Investigators

2. Study participant

**ENGLISH PARENTAL CONSENT FORM**

**KENYA MEDICAL RESEARCH INSTITUTE**

**WASHINGTON UNIVERSITY, ST. LOUIS**

**PARENTAL CONSENT FOR FOCUS GROUP DISCUSSIONS (FGD)**

**Study Title:** *Adaptive Strategies to Prevent and Treat Failures of Retention in Adolescent HIV Care (A4A Study)*

INVESTIGATORS AND INSTITUTIONAL AFFILIATIONS:

| Researcher | Institution | Contact |
| --- | --- | --- |
| Dr. Elizabeth Bukusi | Kenya Medical Research Institute | +254 733-617 503 |
| Eliud Akama | Kenya Medical Research Institute | +254 728-590 376 |
| Dr. Elvin Geng | Washington University, St. Louis | +1-415-430-5589 (USA) |
| Dr. Lisa Abuogi | University of Colorado, Denver | +1- 303-724-7430  (USA) |
| Dr. Zachary Kwena | Kenya Medical Research Institute | +254 722-386 714 |
| Starley Shade | University of California, San Francisco | +1-415-794-4354 (USA) |
| Lina Montoya | University of Notrh Carolina at Chapel Hill | +1-4105857470 (USA) |

**Study Location:** Kisumu County

You are being asked to allow your child to take part in a research study that involves participating in focus group discussions. The box below tells you important things you should think about before deciding to allow your child to participate in the discussion. We will provide more detailed information below the box. Please ask questions about any of the information before you decide whether to allow your child to participate or not. You may also wish to talk to others (for example, your family, friends, or the researchers) about this study, before agreeing to join.

- **Voluntary Consent**. You are being asked to allow your child to participate in a focus group discussion. It is up to you whether you choose to allow your child to participate or not. There are no penalties and you will not lose anything if you decide not to allow your child to participate.
- **Purpose**. We are conducting three focus group discussions to gather adolescent and young adult thoughts about interventions strategies planned to help adolescents and young adults stay involved in HIV care. The planned interventions involve electronic and in-person peer navigation (similar to counseling) and cash transfers. Your child’s views and information will help inform a special meeting about the interventions called a design workshop. Your child may be contacted about participating in the design workshop at a later stage.
- **Participants:** About 24 adolescents and young adults, 14 – 24 years old, and living with HIV in Kisumu county will participate in the focus group discussions. There will be 3 groups with about 8 in each group.
- **Procedures and Activities.** In this study, if you agree to allow your child to participate in the focus group discussion, we will also ask for your child’s permission. If you both agree, we will set up a time for your child to join a focus group discussion. The discussion will focus on one of three planned intervention strategies to help keep adolescents and young adults engaged in HIV care. Each discussion will be conducted by a trained interviewer in a private place to protect your child’s confidentiality. In addition, we are asking your permission to audio record the discussion, which we will then write down and review. Any information that could potential identify your child will not be included in the review. We expect the focus group discussions to last one and half hours.

**Risks.** There is a small chance of accidental disclosure of HIV status. However, we will put safety measures in place to protect information about your child’s HIV status.

- **Benefits**. Your child may not receive any direct benefits from participating in this study. By participating in this study he/she may contribute to efforts to promote the health of adolescents living with HIV.
- **Alternatives.** You are free to choose not to allow your child to participate in the discussions. If you decide not to allow your child to take part in this study, there will be no penalty to him/her. He/she will not lose any of your regular benefits, and can still get his/her care from the clinic the way you usually do.

**Procedures and Activities**

If you agree to allow your child to participate, and your child agrees too, the following things will occur:

1. We will ask you to sign or make a mark on this form as a sign that you have voluntarily accepted to participate in this research and seek your child’s permission.
2. We will set up time with him/her to join a group of other adolescents to discuss how best to implement the planned study intervention strategies.
3. Your child will be part of discussion focused on one of three planned intervention strategies, either electronic navigation (automated SMS messages and peer support through mobile phones), in-person peer navigation support, or conditional cash transfer (receiving money).
4. He/she will be asked questions about experiences and challenges with HIV care, how often and what type of support is needed, and suggestions for how to best carry out the planned interventions.
5. His/her views and information will help inform an intervention meeting called a design workshop.
6. The discussion led by a trained interviewer in private room to protect confidentiality.
7. We will ask him/her to allow us to record the interview and his/her answers so that we can write it down later and review.
8. Any information that could potentially identify your child will not be included in the review.
9. The discussion is expected to take about one and a half hours.
10. Your child may be asked at a later date to participate in that one week workshop. Let us know if you will be willing to allow your child to participate.

**Risks**

There is a small chance of accidental disclosure of HIV status. However, we will put safety measure in place to protect information about your child’s HIV status.

**Benefits**

Your child may not receive any direct benefits from participating in this study. However, your child’s participation may help improve retention of adolescents in care.

**Reimbursement**

Your child will be given a transport reimbursement of Kshs. 500/=.

**Alternatives**

You are free to choose not to allow your child to participate in the study. If you decide not to allow your child to take part in this study, there will be no penalty to him/her. He/she will not lose any of the regular benefits, and can still get his/her care from the clinic the way you usually do.

**Confidentiality**

We will do our best to ensure that the personal information gathered for this study is kept private. However, we cannot guarantee total privacy. Your child’s personal information may be given out if required by law. If information from this study is published or presented at scientific meetings, your or your child’s name and other personal information will not be used.

Government or university staff sometimes review studies such as this one to make sure they are being done safely and legally. If a review of this study takes place, your records may be examined. The reviewers will protect your privacy. The study records will not be used to put you at legal risk of harm. Organizations that may look at and/or copy your research records for research, quality assurance, and data analysis include:

- The Washington University, St. Louis
- The Kenya Medical Research Institute
- University of California San Francisco
- University of Colorado, Denver
- University of California, Berkeley

Participation in research may involve a loss of privacy, however we will put safety measures in place to protect confidentiality. Any additional information about the study will be provided to you including the final study results.

**Participation**

Your child can decide to stop the discussion at any time, just tell the study staff and they will stop him/her. In addition, the study staff can stop him/her from taking part, if they feel it is in their best interest. He/she will not be penalized in any way should he/she choose to stop participating.

**Sponsorship**

The study is sponsored by the National Institutes of Health. None of the investigators have any financial conflict of interests. In situations where the study may be terminated at the discretion of the investigator or study sponsor, you will be informed the next course of action.

**Contact**

You can talk to the researcher(s) about any questions, concerns, or complaints you have about this study. Contact our study staff at 0733-819 835. If you wish to ask questions about the study or your rights as a research participant to someone other than the researchers or if you wish to voice any problems or concerns you may have about the study, you may contact Secretary of the Ethical Review Committee, Kenya Medical Research Institute at seru@kemri.org or P.O. BOX 54840-00200 Tel. 020-2722541, 0717719477. You may also contact the Washington University Human Research Protection Office at +1-314-747-6800.These committees are concerned with the protection of volunteers in research projects.

**CONSENT**

You have been given a copy of this consent form to keep.

**PARTICIPATION IN RESEARCH IS VOLUNTARY.** You have the right to decline to be in this study, or to withdraw from it at any point without penalty or loss of benefits to which you are otherwise entitled.

**Participant’s Statement**

The study described above has been explained to me. I agree to allow my child to take part in this research. I have had a chance to ask questions. If I have future questions about the research, I can ask one of the investigators listed above. If I have questions about my rights as a research participant, I can contact those listed above.

I agree for my child’s information to be used for future research and shared with other researchers without my additional consent with/without identifiers.

If you wish to allow your child to participate in this study, you should sign below.

Date	Participant's Name

Date	Caregiver/Parent's Name and Signature for Consent

Date	Person Obtaining Consent (Name and signature)

***If illiterate***

I have witnessed the accurate reading of the consent form to the participant and the individual has had the opportunity to ask questions. I confirm that the individual has given consent freely**.**

Name of witness_______________________ AND

Signature of witness ____________________AND

Name of Caregiver/Parent _______________AND

Thumbprint of Caregiver/Parent

**Date ________________________**

**Verbal Consent**

PARTICIPATION IN RESEARCH IS VOLUNTARY. You have the right to decline to be in this study, or to withdraw from it at any point without penalty or loss of benefits to which you are otherwise entitled.

Consents to study participation Does not consent to study participation

[Grab your reader’s attention with a great quote from the document or use this space to emphasize a key point. To place this text box anywhere on the page, just drag it.]

[Grab your reader’s attention with a great quote from the document or use this space to emphasize a key point. To place this text box anywhere on the page, just drag it.]

[Grab your reader’s attention with a great quote from the document or use this space to emphasize a key point. To place this text box anywhere on the page, just drag it.]

___________________________________________________________________

Printed Name of Participant	Date

___________________________________________________________________

Signature of Person Obtaining Assent	Title

**Permission to audio-record discussion**

I give permission for the group discussion in which my child will participate to be recorded: Yes ☐	No ☐

**Permission to be contacted for a** week’s **workshop**

I accept my child to be contacted to participate in a **week’s** workshop:

Yes ☐	No ☐

Copies to:	1. Investigators

2. Study participant

**KENYA MEDICAL RESEARCH INSTITUTE**

**WASHINGTON UNIVERSITY, ST LOUIS**

**ENGLISH A4A ASSENT TO PARTICIPATE IN AN INDEPTH INTERVEIW (IDI)*(14 - 17 YEARS OLD)***

**Study Title:** *Adaptive Strategies to Prevent and Treat Failures of Retention in Adolescent HIV Care (A4A Study)*

INVESTIGATORS AND INSTITUTIONAL AFFILIATIONS:

| Researcher | Institution | Contact |
| --- | --- | --- |
| Dr. Elizabeth Bukusi | Kenya Medical Research Institute | +254 733-617 503 |
| Eliud Akama | Kenya Medical Research Institute | +254 728-590 376 |
| Dr. Elvin Geng | Washington University, St Louis | +1-415-430-5589 (USA) |
| Dr. Lisa Abuogi | University of Colorado, Denver | + 1- 303-724-7430 (USA) |
| Dr. Zachary Kwena | Kenya Medical Research Institute | +254 722-386 714 |
| Starley Shade | University of California, San Francisco | +1-415-794-4354 (USA) |
| Lina Montoya | University of Notrh Carolina at Chapel Hill | +1-4105857470 (USA) |

**Study Location:** Kisumu County

We want to tell you about something we are doing called a research study. A research study is when doctors collect a lot of information to learn more about something. We are asking you to volunteer to take part in an interview which is part of a research study we are conducting. The box below tells you important things you should think about before deciding to participate in this interview or not. We will provide more detailed information below the box. Please ask questions about any of the information before you decide whether to participate. You may also wish to talk to others (for example, your family, friends, or the researchers) about this study, before agreeing to join.

- **Voluntary Consent**. You are being asked to volunteer to participate in an interview. It is up to you whether you choose to participate or not. There are no penalties and you will not lose anything if you decide not to join or if after you join, you decide to quit.
- **Purpose**. We are conducting interviews to assess AYA experiences with the interventions in this study. We would like to understand your thoughts, satisfaction, and experiences with the intervention received. Your views and information will help us understand how the intervention is working.
- **Participants:** About 60 adolescents and young adults, 14 – 24 years old, who are enrolled in the A4A study in Kisumu county, will participate in the interviews.
- **Procedures and Activities.** If you agree to participate, we will set up a time to interview you. The interview will be conducted by a trained interviewer in a private place to protect your confidentiality. You will be asked questions about your satisfaction and experiences with an intervention you received, either electronic peer navigation (automated SMS messages and peer counseling through mobile phones), in-person peer navigation, or conditional cash transfer. We will ask you about one of the three interventions only. In addition, we will ask your permission to audio record the interview, which we will then write down and review. Any information that could potential identify you will not be included in the review. We expect the interview to last about 45-60 minutes.
- **Risks.** There is a small chance of accidental disclosure of HIV status. However, we will put safety measures in place to protect information about your HIV status.
- **Benefits**. You may not receive any direct benefits from participating in this study. By participating in this study you may contribute to efforts to promote the health of adolescents living with HIV.
- **Alternatives.** You are free to choose not to participate in the study. If you decide not to take part in this study, there will be no penalty to you. You will not lose any of your regular benefits, and you can still get your care from your clinic the way you usually do.

**Procedures and Activities**

In the event your parent/caregiver is not present during this recruitment, the Research Assistant will contact your parent/caregiver to consent over the phone.

If you agree, the following things will happen:

1. We will ask you to sign or make a mark on this form as a sign that you have voluntarily accepted to participate in this research.
2. We will set up time to interview you.
3. We will ask you questions about your thoughts, satisfaction, and experiences with an intervention you received, which will either be electronic navigation, in-person navigation, or conditional cash transfer.
4. Your views and information will help us understand how the intervention is working.
5. The interview will be led by a trained interviewer in private room to protect your confidentiality.
6. We will ask you to allow us to record the interview and your answers so that we can write it down later and review.
7. Any information that could potentially identify you will not be included in the review.
8. The interview will last about 45-60 minutes.

**Risks**

There is a small chance of accidental disclosure of HIV status. However, we will put safety measure in place protect information about your HIV status.

**Benefits**

You may not receive any direct benefits from participating in this study. However, your participation may help improve retention of adolescents in care.

**Reimbursement**

You will be given a transport reimbursement of Kshs. 500/=.

**Alternatives**

You are free to choose not to participate in the study. If you decide not to take part in this study, there will be no penalty to you. You will not lose any of your regular benefits, and you can still get your care from your clinic the way you usually do.

**Confidentiality**

We will do our best to ensure that the personal information gathered for this study is kept private. However, we cannot guarantee total privacy. Your personal information may be given out if required by law. If information from this study is published or presented at scientific meetings, your name and other personal information will not be used.

Government or university staff sometimes review studies such as this one to make sure they are being done safely and legally. If a review of this study takes place, your records may be examined. The reviewers will protect your privacy. The study records will not be used to put you at legal risk of harm. Organizations that may look at and/or copy your research records for research, quality assurance, and data analysis include:

- The Washington University, St Louis
- The Kenya Medical Research Institute
- University of California San Francisco
- University of Colorado, Denver
- University of California, Berkeley

Participation in research may involve a loss of privacy, however we will put safety measures in place to protect your confidentiality . Any additional information about the study will be provided to you including the final study results.

**Participation**

You can decide to stop the interview at any time, just tell the study staff and they will stop you. In addition, the study staff can stop from taking part, if its in your best interest. You will not be penalized in any way should you choose to stop participating.

**Sponsorship**

The study is sponsored by the National Institutes of Health. None of the investigators have any financial conflict of interests. In situations where the study may be terminated at the discretion of the investigator or study sponsor, you will be informed the next course of action.

**Contact**

You can talk to the researcher(s) about any questions, concerns, or complaints you have about this study. Contact our study staff at 0733-819 835. If you wish to ask questions about the study or your rights as a research participant to someone other than the researchers or if you wish to voice any problems or concerns you may have about the study, you may contact Secretary of the Ethical Review Committee, Kenya Medical Research Institute at seru@kemri.org or P.O. BOX 54840-00200 Tel. 020-2722541, 0717719477. You may also contact the Washington University Human Research Protection Office at +1-314-747-6800 These committees are concerned with the protection of volunteers in research projects.

**CONSENT**

You have been given a copy of this consent form to keep.

**PARTICIPATION IN RESEARCH IS VOLUNTARY.** You have the right to decline to be in this study, or to withdraw from it at any point without penalty or loss of benefits to which you are otherwise entitled.

**Participant’s Statement**

The study described above has been explained to me. I volunteer to take part in this research. I have had a chance to ask questions. If I have future questions about the research, I can ask one of the investigators listed above. If I have questions about my rights as a research participant, I can contact those listed above.

I agree for my information to be used for future research and shared with other researchers without my additional consent with/without identifiers.

If you wish to participate in this study, you should sign below.

Date	Participant's Name and Signature for Consent

Date	Person Obtaining Consent (Name and signature)

***If illiterate***

I have witnessed the accurate reading of the consent form to the participant and the individual has had the opportunity to ask questions. I confirm that the individual has given consent freely**.**

Name of witness_____________________ AND

Signature of witness *__________________*AND

Name of participant __________________AND

**Thumbprint of participant**

**Date ________________________**

**Permission to audio-record discussion**

I give permission for the group discussion in which I will participate to be recorded: Yes ☐	No ☐

Copies to:	1. Investigators

2. Study participant

**KENYA MEDICAL RESEARCH INSTITUTE**

**WASHINGTON UNIVERSITY, ST LOUIS**

**ENGLISH CONSENT TO PARTICIPATE IN AN INDEPTH INTERVEIW (IDI)*(18 - 24 YEARS OLD)***

**Study Title:** *Adaptive Strategies to Prevent and Treat Failures of Retention in Adolescent HIV Care (A4A Study)*

INVESTIGATORS AND INSTITUTIONAL AFFILIATIONS:

| Researcher | Institution | Contact |
| --- | --- | --- |
| Dr. Elizabeth Bukusi | Kenya Medical Research Institute | +254 733-617 503 |
| Eliud Akama | Kenya Medical Research Institute | +254 728-590 376 |
| Dr. Elvin Geng | Washington University, St Louis | +1-415-430-5589 (USA) |
| Dr. Lisa Abuogi | University of Colorado, Denver | + 1- 303-724-7430 (USA) |
| Dr. Zachary Kwena | Kenya Medical Research Institute | +254 722-386 714 |
| Starley Shade | University of California, San Francisco | +1-415-794-4354 (USA) |
| Lina Montoya | University of Notrh Carolina at Chapel Hill | +1-4105857470 (USA) |

**Study Location:** Kisumu County

You are being asked to volunteer to take part in an interview. The box below tells you important things you should think about before deciding to participate in this interview. Please ask questions about any of the information before you decide whether to participate or not. You may also wish to talk to others (for example, your family, friends, or the researchers) about this study, before agreeing to join.

- **Voluntary Consent**. You are being asked to volunteer to participate in an interview. It is up to you whether you choose to participate or not. There are no penalties and you will not lose anything if you decide not to join or if after you join, you decide to quit.
- **Purpose**. We are conducting interviews to assess adolescent and young adult experiences with the interventions in this study. We would like to understand your thoughts, satisfaction, and experiences with an intervention you received. Your views and information will help us understand how the intervention is working.
- **Participants:** About 60 adolescents and young adults, 14 – 24 years old, who are enrolled in the A4A study in Kisumu county, will participate in the interviews.
- **Procedures and Activities.** If you agree to participate, we will set up a time to interview you. The interview will be conducted by a trained interviewer in a private place to protect your confidentiality. You will be asked questions about your satisfaction and experiences with an intervention you received, either electronic peer navigation (automated SMS messages and peer counseling through mobile phones), in-person peer navigation, or conditional cash transfer. We will ask you about one of the three interventions only. In addition, we will ask your permission to audio record the interview, which we will then write down and review. Any information that could potential identify you will not be included in the review. We expect the interview to last about 45-60 minutes.
- **Risks.** There is a small chance of accidental disclosure of HIV status. However, we will put safety measures in place to protect information about your HIV status.
- **Benefits**. You may not receive any direct benefits from participating in this study. By participating in this study, you may contribute to efforts to promote the health of adolescents living with HIV.
- **Alternatives.** You are free to choose not to participate in the study. If you decide not to take part in this study, there will be no penalty to you. You will not lose any of your regular benefits, and you can still get your care from your clinic the way you usually do.

**Procedures and Activities**

If you agree, the following things will happen:

1. We will ask you to sign or make a mark on this form as a sign that you have voluntarily accepted to participate in this research.
2. We will set up time to interview you.
3. We will ask you questions about your thoughts, satisfaction, and experiences with an intervention you received, which will either be electronic navigation, in-person navigation, or conditional cash transfer.
4. Your views and information will help us understand how the intervention is working.
5. The interview will be led by a trained interviewer in private room to protect your confidentiality.
6. We will ask you to allow us to record the interview and your answers so that we can write it down later and review.
7. Any information that could potentially identify you will not be included in the review.
8. The interview will last about 45-60 minutes.

**Risks**

There is a small chance of accidental disclosure of HIV status. However, we will put safety measure in place protect information about your HIV status.

**Benefits**

You may not receive any direct benefits from participating in this study. However, your participation may help improve retention of adolescents in care.

**Reimbursement**

You will be given a transport reimbursement of Kshs. 500/=.

**Alternatives**

You are free to choose not to participate in the study. If you decide not to take part in this study, there will be no penalty to you. You will not lose any of your regular benefits, and you can still get your care from your clinic the way you usually do.

**Confidentiality**

We will do our best to ensure that the personal information gathered for this study is kept private. However, we cannot guarantee total privacy. Your personal information may be given out if required by law. If information from this study is published or presented at scientific meetings, your name and other personal information will not be used.

Government or university staff sometimes review studies such as this one to make sure they are being done safely and legally. If a review of this study takes place, your records may be examined. The reviewers will protect your privacy. The study records will not be used to put you at legal risk of harm. Organizations that may look at and/or copy your research records for research, quality assurance, and data analysis include:

- The Washington University, St Louis
- The Kenya Medical Research Institute
- University of California San Francisco
- University of Colorado, Denver
- University of California, Berkeley

Participation in research may involve a loss of privacy, however we will put safety measures in place to protect your confidentiality. Any additional information about the study will be provided to you including the final study results.

**Participation**

You can decide to stop the interview at any time, just tell the study staff and they will stop you. In addition, the study staff can stop from taking part, if its in your best interest. You will not be penalized in any way should you choose to stop participating.

**Sponsorship**

The study is sponsored by the National Institutes of Health. None of the investigators have any financial conflict of interests. In situations where the study may be terminated at the discretion of the investigator or study sponsor, you will be informed the next course of action.

**Contact**

You can talk to the researcher(s) about any questions, concerns, or complaints you have about this study. Contact our study staff at 0733-819 835. If you wish to ask questions about the study or your rights as a research participant to someone other than the researchers or if you wish to voice any problems or concerns you may have about the study, you may contact Secretary of the Ethical Review Committee, Kenya Medical Research Institute at seru@kemri.org or P.O. BOX 54840-00200 Tel. 020-2722541, 0717719477. You may also contact the Washington University Human Research Protection Office at +1-314-747-6800 These committees are concerned with the protection of volunteers in research projects.

**CONSENT**

You have been given a copy of this consent form to keep.

**PARTICIPATION IN RESEARCH IS VOLUNTARY.** You have the right to decline to be in this study, or to withdraw from it at any point without penalty or loss of benefits to which you are otherwise entitled.

**Participant’s Statement**

The study described above has been explained to me. I volunteer to take part in this research. I have had a chance to ask questions. If I have future questions about the research, I can ask one of the investigators listed above. If I have questions about my rights as a research participant, I can contact those listed above.

I agree for my information to be used for future research and shared with other researchers without my additional consent with/without identifiers.

If you wish to participate in this study, you should sign below.

Date	Participant's Name and Signature for Consent

Date	Person Obtaining Consent (Name and signature)

***If illiterate***

I have witnessed the accurate reading of the consent form to the participant and the individual has had the opportunity to ask questions. I confirm that the individual has given consent freely**.**

Name of witness_____________________ AND

Signature of witness *__________________*AND

Name of participant __________________AND

**Thumbprint of participant**

**Date ________________________**

**Permission to audio-record discussion**

I give permission for the group discussion in which I will participate to be recorded: Yes ☐	No ☐

Copies to:	1. Investigators

2. Study participant

**KENYA MEDICAL RESEARCH INSTITUTE**

**WASHINGTON UNIVERSITY, ST. LOUIS**

**ENGLISH PARENTAL CONSENT FOR IN-DEPTH INTERVIEWS**

**Study Title:** *Adaptive Strategies to Prevent and Treat Failures of Retention in Adolescent HIV Care (A4A Study)*

INVESTIGATORS AND INSTITUTIONAL AFFILIATIONS:

| Researcher | Institution | Contact |
| --- | --- | --- |
| Dr. Elizabeth Bukusi | Kenya Medical Research Institute | +254 733-617 503 |
| Eliud Akama | Kenya Medical Research Institute | +254 728-590 376 |
| Dr. Elvin Geng | Washington University, St. Louis | +1-415-430-5589 (USA) |
| Dr. Lisa Abuogi | University of Colorado, Denver | +1-303-724-7430 (USA) |
| Dr. Zachary Kwena | Kenya Medical Research Institute | +254 722-386 714 |
| Starley Shade | University of California, San Francisco | +1-415-794-4354 (USA) |
| Lina Montoya | University of Notrh Carolina at Chapel Hill | +1-4105857470 (USA) |

**Study Location:** Kisumu County

You are being asked to allow your child to take part in an interview. The box below tells you important things you should think about before deciding to allow your child to participate in the interview. We will provide more detailed information below the box. Please ask questions about any of the information before you decide whether to allow your child to participate. You may also wish to talk to others (for example, your family, friends, or the researchers) about this study, before agreeing to join.

- **Voluntary Consent**. You are being asked to allow your child to participate in an interview. It is up to you whether you choose to allow your child to participate or not. There are no penalties and you will not lose anything if you decide not to join or if after you join, you decide to quit.

**Purpose**. We are conducting interviews to assess adolescent and young adult experiences with the interventions in this study. We would like to understand your child’s thoughts, satisfaction, and experiences with an intervention he/she received. Your child’s views and information will help us understand how the intervention is working.

- **Participants:** About 60 adolescents and young adults, 14 – 24 years old, who are enrolled in the A4A study in Kisumu county, will participate in the interviews.
- **Procedures and Activities.** If you agree to allow your child to participate in the interview, we will also ask for your child’s permission. If you both agree to participate, we will set up a time for your child to be interviewed. The interview will be conducted by a trained interviewer in a private place to protect your child’s confidentiality. Your child will be asked questions about his/her satisfaction and experiences with an intervention he/she received, either electronic peer navigation (automated SMS messages and peer counseling through mobile phones), in-person peer navigation, or conditional cash transfer. We will ask your child about one of the three interventions only. In addition, we are asking your permission to audio record the discussion, which we will then write down and review. Any information that could potential identify your child will not be included in the review. We expect the interview to last about 45-60 minutes.
- **Risks.** There is a small chance of accidental disclosure of HIV status. However, we will put safety measures in place to protect information about your child’s HIV status.
- **Benefits**. Your child may not receive any direct benefits from participating in this study. By participating in this study he/she may contribute to efforts to promote the health of adolescents living with HIV.
- **Alternatives.** You are free to choose not to allow your child to participate in the interviews. If you decide not to allow your child to take part in this study, there will be no penalty to him/her. He/she will not lose any of your regular benefits, and can still get his/her care from the clinic the way you usually do.

**Procedures and Activities**

If you agree to allow your child to participate, and your child agrees too, the following things will occur:

11. We will ask you to sign or make a mark on this form as a sign that you have voluntarily accepted to participate in this research and seek your child’s permission.
12. We will set up time to interview your child.
13. We will ask your child questions about his/her thoughts, satisfaction, and experiences with an intervention he/she was assigned to receive, which will either be electronic navigation, in-person navigation, or conditional cash transfer.
14. Your child’s views and information will help us understand how the intervention is working.
15. The interview will be led by a trained interviewer in private room to protect your confidentiality.
16. We will ask him/her to allow us to record the interview and his/her answers so that we can write it down later and review.
17. Any information that could potentially identify your child will not be included in the review.
18. The interview will last about 45-60 minutes.

**Risks**

There is a small chance of accidental disclosure of HIV status. However, we will put safety measure in place to protect information about your child’s HIV status.

**Benefits**

Your child may not receive any direct benefits from participating in this study. However, your child’s participation may help improve retention of adolescents in care.

**Reimbursement**

Your child will be given a transport reimbursement of Kshs. 500/=.

**Alternatives**

You are free to choose not to allow your child to participate in the study. If you decide not to allow your child to take part in this study, there will be no penalty to him/her. He/she will not lose any of the regular benefits, and can still get his/her care from the clinic the way you usually do.

**Confidentiality**

We will do our best to ensure that the personal information gathered for this study is kept private. However, we cannot guarantee total privacy. Your child’s personal information may be given out if required by law. If information from this study is published or presented at scientific meetings, your name and other personal information will not be used.

Government or university staff sometimes review studies such as this one to make sure they are being done safely and legally. If a review of this study takes place, your records may be examined. The reviewers will protect your privacy. The study records will not be used to put you at legal risk of harm. Organizations that may look at and/or copy your research records for research, quality assurance, and data analysis include:

- The Washington University, St. Louis
- The Kenya Medical Research Institute
- University of California San Francisco
- University of Colorado, Denver
- University of California, Berkeley

Participation in research may involve a loss of privacy, however we will put safety measures in place to protect your child’s confidentiality. Any additional information about the study will be provided to you including the final study results.

**Participation**

Your child can decide to stop the interview at any time, just tell the study staff and they will stop him/her. In addition, the study staff can stop him/her from taking part, if its in the best interest. He/she will not be penalized in any way should he/she choose to stop participating.

**Sponsorship**

The study is sponsored by the National Institutes of Health. None of the investigators have any financial conflict of interests. In situations where the study may be terminated at the discretion of the investigator or study sponsor, you will be informed the next course of action.

**Contact**

You can talk to the researcher(s) about any questions, concerns, or complaints you have about this study. Contact our study staff at 0733-819 835. If you wish to ask questions about the study or your rights as a research participant to someone other than the researchers or if you wish to voice any problems or concerns you may have about the study, you may contact Secretary of the Ethical Review Committee, Kenya Medical Research Institute at seru@kemri.org or P.O. BOX 54840-00200 Tel. 020-2722541, 0717719477. You may also contact the Washington University Human Research Protection Office at +1-314-747-6800.These committees are concerned with the protection of volunteers in research projects.

**CONSENT**

You have been given a copy of this consent form to keep.

**PARTICIPATION IN RESEARCH IS VOLUNTARY.** You have the right to decline to be in this study, or to withdraw from it at any point without penalty or loss of benefits to which you are otherwise entitled.

**Participant’s Statement**

This study described above has been explained to me. I agree to allow my child to take part in this research. I have had a chance to ask questions. If I have future questions about the research, I can ask one of the investigators listed above. If I have questions about my rights as a research participant, I can contact those listed above.

I agree for my child’s information to be used for future research and shared with other researchers without my additional consent with/without identifiers.

If you wish to allow your child to participate in this study, you should sign below.

Date	Participant's Name and Signature for Consent

Date	Person Obtaining Consent (Name and signature)

***If illiterate***

I have witnessed the accurate reading of the consent form to the participant and the individual has had the opportunity to ask questions. I confirm that the individual has given consent freely**.**

Name of witness_____________________ AND

Signature of witness *__________________*AND

Name of participant __________________AND

**Thumbprint of participant**

**Date ________________________**

**Permission to audio-record discussion**

I give permission for the group discussion in which my child will participate to be recorded: Yes ☐	No ☐

Copies to:	1. Investigators

2. Study participant

**Consent Translations**

***Adaptive Strategies to Prevent and Treat Failures of Retention in***

***Adolescent HIV Care (A4A Study)***

**KISWAHILI A4A NAKALA YA IDHINI YA MATAMSHI KWA JARIBIO LA CHAGUO (DCE)MIAKA 18 NA ZAIDI**

WATAFITI NA TAASISI SHIRIKISHI:

| MTAFITI | TAASISI | MAWASILIANO |
| --- | --- | --- |
| Dkt. Elizabeth Bukusi | Taasisi ya Kenya ya Utafiti wa Kimatibabu, | +254 733-617 503 |
| Dkt. Elvin Geng | Chuo Kikuu cha Washington, Missouri | +1-415-430-5589 (MAREKANI) |
| Dkt. Lisa Abuogi | Chuo Kikuu cha Colorado, Denver | +1-303-724-7430(MAREKANI) |
| Dkt. Zachary Kwena | Taasisi ya Kenya ya Utafiti wa kimatibabu, | +254 722-386 714 |
| Starley Shade | Chuo Kikuu cha California, San Francisco | +1-415-794-4354 (MAREKANI) |
| Lina Montoya | University of Notrh Carolina at Chapel Hill | +1-4105857470 (USA) |
| Eliud Akama | Taasisi ya Kenya ya Utafiti wa Kimatibabu, | +254 728-590 376 |

**Eneo la Utafiti:** Kaunti ya Kisumu

**Utangulizi**

Mimi ni [jina la RA/APL], kutoka Taasisi ya Kenya ya Utafiti wa Kimatibabu, (KEMRI). Tunafanya mradi wa utafiti wenye anwani: **An Adaptive Strategy for Preventing and Treating Lapses of Retention in HIV Care for Adolescents (A4A).** Utafiti huu unafanywa na Taasisi ya Kenya ya Utafiti wa Kimatibabu,KEMRI na Chuo Kikuu cha Washington iliyo St. Louis, Chuo Kikuu cha Colorado iliyo Denver, Vyuo vikuu vya California huko Berkeley na San Francisco vilivyo Marekani. Kuhakikisha kuwa vijana na vijana wazima wanaoishi na HIV wanasalia kwenye utunzaji wa HIV na kupunguza viwango vyao vya virusi hadi sufuri ni muhimu sana kwa kuendelea kuwa na afya. Katika utafiti huu, tunajaribu kujifunza jinsi ya kuwapa vijana na vijana wazima mikakati itakayowasaidia kusalia kwenye utunzaji na kusalia na viwango vya virusi vya sufuri.

Tunakuomba ushiriki katika pelelezi ya dakika 30 kusaidia kuunda mikakati ya **Kimasharti ya Uhamishaji wa Pesa (CCT)** ya mradi huu. Kusudi la pelelezi hii ni kujua mapendeleo ya vijana na vijana wazima (AYA) kuhusu marupurupu wakati wa utunzaji na matibabu ya HIV.

Wakati wa pelelezi hii, Mtafiti Msaidizi atasoma maswali na kukuonyesha kadi inayokuonyesha machaguo yaliyopendekezwa katika kila swali. Utachagua upendeleo wako na yeye ataingiza habari hii kwenye kibao cha utafiti. Kama vijana na vijana wazima katika jamii hii, tungependa kujua mapendeleo yako kuhusu marupurupu kutusaidia kuunda mikakati bora ya kuingilia kati kwa vijana na vijana wazima.

Kushiriki kwako ni kwa hiari. Ikiwa hutaki kushiriki, unaweza kuwacha wakati wowote na hakutakuwa na adhabu yoyote kwako. Hutapoteza faida zako zozote za kawaida na bado unaweza kupata utunzaji wako kutoka kwa kliniki yako kama kawaida. Majibu hayatajulikana kabisa; jina lako halitatokea popote katika maandiko ya mwisho.

Je, una maswali yoyote kuhusu mradi huu?

Ikiwa utahitaji nakala ya barua hii kwa rekodi zako, tafadhali nijulishe [nitakupa nakala sasa;kupitia kwa barua pepe, barua, ama faksi na kadhalika].

Ikiwa una maswali zaidi kuhusu utafiti, wasiliana na Dkt. Elizabeth Ann Bukusi, Taasisi ya Kenya ya Utafiti wa Kimatibabu (KEMRI), 0733617503 ama Dkt. Elvin Geng, Chuo Kikuu cha Washington, + 14154305589. Ikiwa una maswali yoyote kuhusu haki zako kama mshiriki wa utafiti, tafadhali wasiliana na Kamati ya Maadili na Marudio ya Kisayansi (SERU) ya Taasisi ya Kenya ya Utafiti wa Kimatibabu katika 0717719477 ama [seru@kemri.org](mailto:seru@kemri.org) ama Mpango wa Ulinzi wa Wanadamu, Chuo Kikuu cha Washington katika +13147476800.

Jina la Mfanyakazi wa Mradi Anayesimamia Idhini (chapisha)	Wadhifa/Anwani

Sahihi ya Mfanyakazi wa Mradi Anayesimamia Idhini	Tarehe

***Adaptive Strategies to Prevent and Treat Failures of Retention in***

***Adolescent HIV Care (A4A Study)***

**DHOLUO A4A VERBAL CONSENT SCRIPT FOR DISCRETE CHOICE EXPERIMENT (DCE)18 YEARS OLD AND ABOVE**

JOTIM NORO KOD MIGEPE MA GITUDORE GO:

| Jatim Nonro | Migao | Yor Tudruok |
| --- | --- | --- |
| Laktar Elizabeth Bukusi | Migao Mar Nonro marThieth mar Kenya | +254 733-617 503 |
| Laktar Elvin Geng | Mbalariany Mar Washington, Missouri | +1-415-430-5589 (USA) |
| Laktar Lisa Abuogi | Mbalariany Mar Colorado, Denver | +1-303-724-7430 (USA) |
| Laktar Zachary Kwena | Migao Mar Nonro marThieth mar Kenya | +254 722-386 714 |
| Starley Shade | Mbalariany Mar California, San Francisco | +1-415-794-4354 (USA) |
| Lina Montoya | University of Notrh Carolina at Chapel Hill | +1-4105857470 (USA) |

**Kama itimo e Nonro:** Kisumu County

**Wach motelo**

An [nying RA/APL], awuok e Migao mar Nonro mar Thieth e Kenya (KEMRI). Watimo nonro ma iluongo ni **An Adaptive Strategy for Preventing and Treating Lapses of Retention in HIV Care for Adolescents (A4A).** Nonro ni itimo gi KEMRI kod Mbalariany mar Washington man St. Louis, Mbalariany mar Colorado man Denver, gi Mbalariany mar California man Berkeley kod San Francisco e piny Amerka. Neno ni rowere kod jomadongo matindo man gi kute mag ayaki omedo bedo e rit mar kute mag ayaki kendo keto kwan mar gi mag kute mag Ayaki e remo bedo ma ok nyal ne en gimaduong’e keto gi ka gingima. E nonro ni, watemo ng’eyo kaka wanyalo miyo rowere kod jomadongo matindo chenro ma biro konyo gi bedo e rit kendo bedo gi kwan kute maok nyal ne. Wakwayi mondo ibed e duoko penjo mar dakika 30 mar konyo loso chenro mar Oro Manyonge man gi Chik (Conditional Cash Transfer) ne nonro ni . Nonroni itimo mondo ofweny ni gin yiero mage mag kony ma rowere gi jomadongo matindo ohero ekinde mag rit gi thieth mag kute mag Ayaki.

Ekinde duoko penjo, jatij norno biro somoni penjo kendo tangóni kadi mabiro nyisi yiero mihero epenjo kapenjo. Ibiro yiero yiero mihero kendo Jatij nonro biro rwako wechegi etablet mar nonro. Kaka rawera kata ng’a maduong’ matin e oganda ni, dwaher ngéyo gik mihero kuom kony michiwo mondo okonywa loso yore mabeyo mogik ne rowere kod jomadongo matindo.

Bedo mari e nonro en kuom chiwruok. Ka ok diher mar bedo, inyalo wuok e saa asaya kendo onge kum ma biro bedo ne in. Ok ibi wito ber ma iyudo ga ma pile, kendo podi inyalo yudo rit kawuok e klinik mari kaka iyude ga. Dwoko duto ok bi bedo gi nying; nyingi ok bi nenore kamoro amora e ndiko ma otim mogik.

Be in gi penjo moro amora kuom nonro ni?

Ka diher yudo oboke mar barua ni mondo ikan, yie inyisa kendo abiro [miyi obokeno sani; oro ni go e barup mbuyi, barua, kata ore e yor faks, gmk.].

Ka in gi penjo mamoko momedore kotudore gi nonro , tudri kod Laktar Elizabeth Ann Bukusi, mar Migao mar timo Nonro marThiieth epiny Kenya, 0733617503 kata Laktar Elvin Geng, Mbalariany ma Washington, + 14154305589. Ka in gi penjo moro amora kotudore gi ratiro magi kaka jachiwre mar nonro, yie itudri kod Komiti mar Sayans gi Nono Chike mar Kar timo nonro mar Thieth ma Kenya e 0717719477 kata [seru@kemri.org](mailto:seru@kemri.org) KATA Chenro mar Rito Jochiwre mag Nonro mar Mbalariany ma Washington e +13147476800.

Nying Jatij Nonro ma Tayo kawo Yie (ndiki)	Tije/Nying Tije

Seyi Jatij Nonro ma Tayo kawo Yie	Tarik

***Adaptive Strategies to Prevent and Treat Failures of Retention in***

***Adolescent HIV Care (A4A Study)***

**KISWAHILI A4A NAKALA YA IDHINI YA MATAMSHI KWA JARIBIO LA CHAGUO (DCE)*MIAKA 14 – 17***

WATAFITI NA TAASISI SHIRIKISHI:

| Mtafiti | Taasisi | Mawasiliano |
| --- | --- | --- |
| Dkt. Elizabeth Bukusi | Taasisi ya Kenya ya Utafiti wa Kimatibabu | +254 733-617 503 |
| Eliud Akama | Taasisi ya Kenya ya Utafiti wa Kimatibabu | +254 728-590 376 |
| Dkt. Elvin Geng | Chuo Kikuu cha Washington , Missouri | +1-415-430-5589 (MAREKANI) |
| Dkt. Lisa Abuogi | Chuo Kikuu cha Colorado, Denver | +1-303-724-7430(MAREKANI) |
| Dkt. Zachary Kwena | Taasisi ya Kenya ya Utafiti wa Kimatibabu | +254 722-386 714 |
| Starley Shade | Chuo Kikuu cha California, San Francisco | +1-415-794-4354 (MAREKANI) |
| Lina Montoya | University of Notrh Carolina at Chapel Hill | +1-4105857470 (USA) |

**Eneo la Utafiti:** Kaunti ya Kisumu

**Utangulizi**

Mimi ni [jina la RA/APL], kutoka Taasisi ya Kenya ya Utafiti wa Kimatibabu, (KEMRI). Tunafanya mradi wa utafiti wenye anwani: **An Adaptive Strategy for Preventing and Treating Lapses of Retention in HIV Care for Adolescents (A4A).** Utafiti huu unafanywa na Taasisi ya Kenya ya Utafiti wa Kimatibabu, KEMRI na Chuo Kikuu cha Washington iliyo St. Louis, Chuo Kikuu cha Colorado iliyo Denver, Vyuo vikuu vya California huko Berkeley na San Francisco vilivyo Marekani. Kuhakikisha kuwa vijana na vijana wazima wanaoishi na HIV wanasalia kwenye utunzaji wa HIV na kupunguza viwango vyao vya virusi hadi sufuri ni muhimu sana kwa kuendelea kuwa na afya. Katika utafiti huu, tunajaribu kujifunza jinsi ya kuwapa vijana na vijana wazima mikakati itakayowasaidia kusalia kwenye utunzaji na kusalia na viwango vya virusi vya sufuri.

Tunakuomba ushiriki katika pelelezi ya dakika 30 kusaidia kuunda mikakati ya **Kimasharti ya Uhamishaji wa Pesa (CCT)** ya mradi huu. Kusudi la pelelezi hii ni kujua mapendeleo ya vijana na vijana wazima (AYA) kuhusu marupurupu wakati wa utunzaji na matibabu ya HIV.

Wakati wa pelelezi hii, Mtafiti Msaidizi atasoma maswali na kukuonyesha kadi inayokuonyesha machaguo yaliyopendekezwa katika kila swali. Utachagua upendeleo wako na yeye ataingiza habari hii kwenye kibao cha utafiti. Kama vijana na vijana wazima katika jamii hii, tungependa kujua mapendeleo yako kuhusu marupurupu kutusaidia kuunda mikakati bora ya kuingilia kati kwa vijana na vijana wazima.

Kushiriki kwako ni kwa hiari. Ikiwa hutaki kushiriki, unaweza kuwacha wakati wowote na hakutakuwa na adhabu yoyote kwako. Hutapoteza faida zako zozote za kawaida na bado unaweza kupata utunzaji wako kutoka kwa kliniki yako kama kawaida. Majibu hayatajulikana kabisa; jina lako halitatokea popote katika maandiko ya mwisho.

Je, una maswali yoyote kuhusu mradi huu?

Ikiwa utahitaji nakala ya barua hii kwa rekodi zako, tafadhali nijulishe [nitakupa nakala sasa;kupitia kwa barua pepe, barua, ama faksi na kadhalika].

Ikiwa una maswali zaidi kuhusu utafiti, wasiliana na Dkt. Elizabeth Ann Bukusi, Taasisi ya Kenya ya Utafiti wa Kimatibabu (KEMRI), 0733617503 ama Dkt. Elvin Geng, Chuo Kikuu cha Washington, + 14154305589. Ikiwa una maswali yoyote kuhusu haki zako kama mshiriki wa utafiti, tafadhali wasiliana na Kamati ya Maadili na Marudio ya Kisayansi (SERU) ya Taasisi ya Kenya ya Utafiti wa Kimatibabu katika 0717719477 ama [seru@kemri.org](mailto:seru@kemri.org) ama Mpango wa Ulinzi wa Wanadamu, Chuo Kikuu cha Washington katika +13147476800.

Jina la Mfanyakazi wa Mradi Anayesimamia Idhini (chapisha)	Wadhifa/Anwani

Sahihi ya Mfanyakazi wa Mradi Anayesimamia Idhini	Tarehe

***Adaptive Strategies to Prevent and Treat Failures of Retention in***

***Adolescent HIV Care (A4A Study)***

**DHOLUO A4A VERBAL ASSENT SCRIPT FOR DISCRETE CHOICE EXPERIMENT (DCE)*14 – 17 YEARS OLD***

JOTIM NORO KOD MIGEPE MA GITUDORE GO:

| Jatim Nonro | Migao | Yor Tudruok |
| --- | --- | --- |
| Laktar Elizabeth Bukusi | Migao Mar Nonro marThieth mar Kenya | +254 733-617 503 |
| Laktar Elvin Geng | Mbalariany Mar Washington, Missouri | +1-415-430-5589 (USA) |
| Laktar Lisa Abuogi | Mbalariany Mar Colorado, Denver | +1-303-724-7430 (USA) |
| Laktar Zachary Kwena | Migao Mar Nonro marThieth mar Kenya | +254 722-386 714 |
| Starley Shade | Mbalariany Mar California, San Francisco | +1-415-794-4354 (USA) |
| Lina Montoya | University of Notrh Carolina at Chapel Hill | +1-4105857470 (USA) |

**Kama itimo e Nonro:** Kisumu County

**Wach motelo**

An [nying RA/APL], awuok e Migao mar Nonro mar Thieth e Kenya (KEMRI). Watimo nonro ma iluongo ni **An Adaptive Strategy for Preventing and Treating Lapses of Retention in HIV Care for Adolescents (A4A).** Nonro ni itimo gi KEMRI kod Mbalariany mar Washington man St. Louis, Mbalariany mar Colorado man Denver, gi Mbalariany mar California man Berkeley kod San Francisco e piny Amerka. Neno ni rowere kod jomadongo matindo man gi kute mag ayaki omedo bedo e rit mar kute mag ayaki kendo keto kwan mar gi mag kute mag Ayaki e remo bedo ma ok nyal ne en gimaduong’e keto gi ka gingima. E nonro ni, watemo ng’eyo kaka wanyalo miyo rowere kod jomadongo matindo chenro ma biro konyo gi bedo e rit kendo bedo gi kwan kute maok nyal ne.

Wakwayi mondo ibed e duoko penjo mar dakika 30 mar konyo loso chenro mar Oro Manyonge man gi Chik (Conditional Cash Transfer) ne nonro ni . Nonroni itimo mondo ofweny ni gin yiero mage mag kony ma rowere gi jomadongo matindo ohero ekinde mag rit gi thieth mag kute mag Ayaki.

Ekinde duoko penjo, jatij norno biro somoni penjo kendo tangóni kadi mabiro nyisi yiero mihero epenjo kapenjo. Ibiro yiero yiero mihero kendo Jatij nonro biro rwako wechegi etablet mar nonro. Kaka rawera kata ng’a maduong’ matin e oganda ni, dwaher ngéyo gik mihero kuom kony michiwo mondo okonywa loso yore mabeyo mogik ne rowere kod jomadongo matindo.

Bedo mari e nonro en kuom chiwruok. Ka ok diher mar bedo, inyalo wuok e saa asaya kendo onge kum ma biro bedo ne in. Ok ibi wito ber ma iyudo ga ma pile, kendo podi inyalo yudo rit kawuok e klinik mari kaka iyude ga. Dwoko duto ok bi bedo gi nying; nyingi ok bi nenore kamoro amora e ndiko ma otim mogik.

Be in gi penjo moro amora kuom nonro ni?

Ka diher yudo oboke mar barua ni mondo ikan, yie inyisa kendo abiro [miyi obokeno sani; oro ni go e barup mbuyi, barua, kata ore e yor faks, gmk.].

Ka in gi penjo mamoko momedore kotudore gi nonro , tudri kod Laktar Elizabeth Ann Bukusi, mar Migao mar timo Nonro marThiieth epiny Kenya, 0733617503 kata Laktar Elvin Geng, Mbalariany ma Washington, + 14154305589. Ka in gi penjo moro amora kotudore gi ratiro magi kaka jachiwre mar nonro, yie itudri kod Komiti mar Sayans gi Nono Chike mar Kar timo nonro mar Thieth ma Kenya e 0717719477 kata [seru@kemri.org](mailto:seru@kemri.org) KATA Chenro mar Rito Jochiwre mag Nonro mar Mbalariany ma Washington e +13147476800.

Nying Jatij Nonro ma Tayo kawo Yie (printed)	Tije/Nying Tije

Seyi Jatij Nonro ma Tayo kawo Yie

Tarik

***Adaptive Strategies to Prevent and Treat Failures of Retention in***

***Adolescent HIV Care (A4A Study)***

**KISWAHILI A4A NAKALA YA IDHINI YA WAZAZI YA MATAMSHI KWA JARIBIO LA CHAGUO (DCE)MIAKA 14-17**

WATAFITI NA TAASISI SHIRIKISHI:

| Mtafiti | Taasisi | Mawasiliano |
| --- | --- | --- |
| Dkt. Elizabeth Bukusi | Taasisi ya Kenya ya Utafiti wa Kimatibabu | +254 733-617 503 |
| Dkt. Elvin Geng | Chuo Kikuu cha Washington, Missouri | +1-415-430-5589 (MAREKANI) |
| Dkt. Lisa Abuogi | Chuo Kikuu cha Colorado, Denver | +1-303-724-7430(MAREKANI) |
| Dkt. Zachary Kwena | Taasisi ya Kenya ya Utafiti wa Kimatibabu | +254 722-386 714 |
| Starley Shade | Chuo Kikuu cha California, San Francisco | +1-415-794-4354 (MAREKANI) |
| Eliud Akama | Taasisi ya Kenya ya Utafiti wa Kimatibabu | +254 728-590 376 |
| Lina Montoya | University of Notrh Carolina at Chapel Hill | +1-4105857470 (USA) |

**Eneo la Mradi:** Kaunti ya Kisumu

**Utangulizi**

Mimi ni [jina la RA/APL], kutoka Taasisi ya Kenya ya Utafiti wa Kimatibabu, (KEMRI). Tunafanya mradi wa utafiti wenye anwani: **An Adaptive Strategy for Preventing and Treating Lapses of Retention in HIV Care for Adolescents (A4A).** Utafiti huu unafanywa na Taasisi ya Kenya ya Utafiti wa Kimatibabu,KEMRI na Chuo Kikuu cha Washington iliyo St. Louis, Chuo Kikuu cha Colorado iliyo Denver, Vyuo vikuu vya California huko Berkeley na San Francisco vilivyo Marekani. Kuhakikisha kuwa vijana na vijana wazima wanaoishi na HIV wanasalia kwenye utunzaji wa HIV na kupunguza viwango vyao vya virusi hadi sufuri ni muhimu sana kwa kuendelea kuwa na afya. Katika utafiti huu, tunajaribu kujifunza jinsi ya kuwapa vijana na vijana wazima mikakati itakayowasaidia kusalia kwenye utunzaji na kusalia na viwango vya virusi vya sufuri.

Tunakuomba uruhusu mtoto wako ashiriki katika pelelezi ya dakika 30 kusaidia kuunda mikakati ya **Kimasharti ya Uhamishaji wa Pesa (CCT)** ya mradi huu. Kusudi la pelelezi hii ni kujua mapendeleo ya vijana na vijana wazima (AYA) kuhusu marupurupu wakati wa utunzaji na matibabu ya HIV.

Wakati wa pelelezi hii, Mtafiti Msaidizi atasoma maswali na kumwonyesha kadi inayomwonyesha machaguo yaliyopendekezwa katika kila swali. Atachagua upendeleo wake na Mtafiti Msaidizi ataingiza habari hii kwenye kibao cha utafiti. Kama vijana na vijana wazima katika jamii hii, tungependa kujua mapendeleo yao kuhusu marupurupu ili kutusaidia kuunda mikakati bora ya kuingilia kati kwa vijana na vijana wazima.

Kushiriki kwake ni kwa hiari. Ikiwa hataki kushiriki, anaweza kuwacha wakati wowote na hakutakuwa na adhabu yoyote kwake. Hatapoteza faida zake zozote za kawaida na bado anaweza kupata utunzaji wake kutoka kwa kliniki yake kama kawaida. Majibu hayatajulikana kabisa; jina lake halitatokea popote katika maandiko ya mwisho.

Je, una maswali yoyote kuhusu mradi huu?

Ikiwa utahitaji nakala ya barua hii kwa rekodi zako, tafadhali nijulishe [nitakupa nakala sasa;kupitia kwa barua pepe, barua, ama faksi na kadhalika].

Ikiwa una maswali zaidi kuhusu utafiti, wasiliana na Dkt. Elizabeth Ann Bukusi, Taasisi ya Kenya ya Utafiti wa Kimatibabu (KEMRI), 0733617503 ama Dkt. Elvin Geng, Chuo Kikuu cha Washington, + 14154305589. Ikiwa una maswali yoyote kuhusu haki zako kama mshiriki wa utafiti, tafadhali wasiliana na Kamati ya Maadili na Marudio ya Kisayansi (SERU) ya Taasisi ya Kenya ya Utafiti wa Kimatibabu katika 0717719477 ama [seru@kemri.org](mailto:seru@kemri.org) ama Mpango wa Ulinzi wa Wanadamu, Chuo Kikuu cha Washington katika +13147476800.

Jina la Mfanyakazi wa Mradi Anayesimamia Idhini (chapisha)	Wadhifa/Anwani

Sahihi ya Mfanyakazi wa Mradi Anayesimamia Idhini	Tarehe

***Adaptive Strategies to Prevent and Treat Failures of Retention in***

***Adolescent HIV Care (A4A Study)***

**DHOLUO A4A PARENTAL VERBAL CONSENT SCRIPT FOR DISCRETE CHOICE EXPERIMENT (DCE)14-17 YEARS OLD**

JOTIM NORO KOD MIGEPE MA GITUDORE GO:

| Jatim Nonro | Migao | Yor Tudruok |
| --- | --- | --- |
| Laktar Elizabeth Bukusi | Migao Mar Nonro marThieth mar Kenya | +254 733-617 503 |
| Laktar Elvin Geng | Mbalariany Mar Washington, Missouri | +1-415-430-5589 (USA) |
| Laktar Lisa Abuogi | Mbalariany Mar Colorado, Denver | +1-303-724-7430 (USA) |
| Laktar Zachary Kwena | Migao Mar Nonro marThieth mar Kenya | +254 722-386 714 |
| Starley Shade | Mbalariany Mar California, San Francisco | +1-415-794-4354 (USA) |
| Lina Montoya | University of Notrh Carolina at Chapel Hill | +1-4105857470 (USA) |

**Kama itimo e Nonro:** Kisumu County

**Wach motelo**

An [name of RA/APL], ma owuok Kenya Medical Research Institute (KEMRI). Watimo nonro ma iluongo ni **An Adaptive Strategy for Preventing and Treating Lapses of Retention in HIV Care for Adolescents (A4A).** Nonro ni itimo kod KEMRI gi Washington University man St. Louis, University of Colorado man Denver, kod University of California man Berkeley kod San Francisco man Amerka. Neno ni rowere kod jamadongo matindo man gi kute mag ayaki obedo e thieth mar kute mag ayaki kendo keto kwan mar kute bedo ma ok nyal ne gin gimaduong’ e keto gi bedo ka gingima. E nonro ni , watemo ng’eyo kaka inyalo mi rowere kod jomadongo matindo chenro ma nyalo konyogi bedo e thieth kendo bedo gi kwan kute ma ok nyal fwenyo.

Wakwayi mondo ibed e duoko penjo mar dakika 30 mar konyo loso chenro mar Oro Manyonge man gi Chik (Conditional Cash Transfer) ne nonro ni . Nonroni itimo mondo ofweny ni gin yiero mage mag kony ma rowere gi jomadongo matindo ohero ekinde mag rit gi thieth mag kute mag Ayaki.

Ekinde duoko penjo, jatij norno biro someone nyathini penjo kendo tangóne kadi mabiro nyise yiero mohero epenjo kapenjo. Ibiro yiero yiero mohero kendo Jatij nonro biro rwako wechegi etablet mar nonro. Kaka rawera kata ng’a maduong’ matin e oganda ni, dwaher ngéyo gik mihero kuom kony michiwo mondo okonywa loso yore mabeyo mogik ne rowere kod jomadongo matindo.

Bedo nyathini enonro en kuom chiwruok. Ka nyathini ok ohero mar bedo enonoro, onyalo wuok e saa asaya kendo onge kum ma biro bedo ne in. Nyathini ok bi wito ber ma oyudo ga ma pile, kendo pod onyalo yudo rit kawuok e klinik mare kaka oyudo ga. Dwoko duto ok bi bedo gi nying; nyingi ok bi nenore kamoro amora e ndiko ma otim mogik.

Be in gi penjo moro amora kuom nonro ni?

Ka diher yudo oboke mar barua ni mondo ikan, yie inyisa kendo abiro [miyi obokeno sani; oro ni go e barup mbuyi, barua, kata ore e yor faks, gmk.].

Ka in gi penjo mamoko momedore kotudore gi nonro, tudri kod Laktar Elizabeth Ann Bukusi, mar Migao mar timo Nonro marThiieth epiny Kenya, 0733617503 kata Laktar Elvin Geng, Mbalariany ma Washington, + 14154305589. Ka in gi penjo moro amora kotudore gi ratiro magi kaka jachiwre mar nonro, yie itudri kod Komiti mar Sayans gi Nono Chike mar Kar timo nonro mar Thieth ma Kenya e 0717719477 kata [seru@kemri.org](mailto:seru@kemri.org) KATA Chenro mar Rito Jochiwre mag Nonro mar Mbalariany ma Washington e +13147476800.

Nying Jatij Nonro ma Tayo kawo Yie (ndiki)	Tije/Nying Tije

Seyi Jatij Nonro ma Tayo kawo Yie	Tarik

**DHOLUO ASSENT FORM (FGD) 14-17 YEARS OLD**

**KENYA MEDICAL RESEARCH INSTITUTE**

**WASHINGTON UNIVERSITY, ST. LOUIS**

**SIRO YIE MAR BEDO E TWAK MAR KANYAKLA (FGD)**

**Study Title:** *Adaptive Strategies to Prevent and Treat Failures of Retention in Adolescent HIV Care (A4A Study)*

JOTIM NONRO KOD MIGEPE MA GITUDORE GODO:

| Jatim Nonro | Migao | Yor Tudruok |
| --- | --- | --- |
| Laktar Elizabeth Bukusi | Kenya Medical Research Institute | +254 733-617 503 |
| Laktar Elvin Geng | Washington University, St. Louis | +1-415-430-5589 (USA) |
| Laktar Lisa Abuogi | University of Colorado, Denver | +1-303-724-7430 (USA) |
| Laktar Zachary Kwena | Kenya Medical Research Institute | +254 722-386 714 |
| Starley Shade | University of California, San Francisco | +1-415-794-4354 (USA) |
| Lina Montoya | University of Notrh Carolina at Chapel Hill | +1-4105857470 (USA) |
| Eliud Akama | Kenya Medical Research Institute | +254 728-590 376 |

**Kama itimo e Nonro:** Kisumu County

Wadwaro nyisi kuom gimoro ma watimo ma iluongo ni nonro . Nonro en ka lakteche choko weche mathoth mondo ging’e mathoth kuom gimoro . Wakwayi mondo ichiwri mar bedo e twak mar kanyakla ma en bath nonro ma watimo. Boksi man piny kanyo no nyisi gik ber ma onego ipar kapok iyiero mar bedo a twak mar kanyakla ni . Wabiro chiwo wach momedore e boksi man piny kanyo. Yie ipenj penjo kuom wach moro amora kapok iyiero ka idonjo kata ok idonj. Bende inyalo hero mar wuoyo kod jomamoko (kuom ranyisi, jo anyuola ni, osiepe, kata jotim nonro ) kuom nonro ni, kapok iyie mar donjo.

- **Yie kuom chiwruok**. Ikwayi mondo ichiwri mar bedo e twak mar kanyakla . En kuomi mar yiero ka idwaro bedo kata ok idwar . Onge kum kendo ok ibi wito gimoro amora ka iyiero ni ok idonj kata bang’e ka isedonjo, inyalo yiero mar wuok.
- **Gima omiyo itime**. Watimo twak ma kanyakla adek mar choko pach rowere kod jomadongo matindo kuom yore ma ichano mag konyo rowere gi jomadongo matindo bedo e thieth mar kute mag ayaki. Chenro ma ochan go biro bedo gi kony mar mbese ma itimo e yor kompyuta kod ma itimo wang’gi wang’ (chal gi hocho) kod chiwo pesa. Pachi kod weche ma ichiwo biro konyo chiwo paro e romo ma kende kuom yore mag konyo go ma iluongo ni design workshop. Bende inyalo tudruok kodi kuom bedo e twak mar loso chenro e thuolo ma mbele.
- **Jochiwre:** Madirom rowere kod jomadongo matindo 24, man e kind higni 14 – 24, kendo odak gi kute mag ayaki e Kisumu county biro bedo e twak mag kanyakla. Wabiro bedo gi grube 3 kod ji madirom 8 e grup ka grup.
- **Chenro kod gik ma itimo.** Mokwongo**,** wabiro kwayo janyuolni kata jarit ni ka oyie mondo idonj . Ka ok oyie , to ok inyalo donjo. Ka uduto uyie , wabiro keto saa ne in mar bedo e twak mar kanyakla. Twak biro chimo achiel kuom yore konyo adek ma ochan mar konyo rowere kod jamadongo matindo medo edo e thieth mar kute mag ayaki. Twak mar kanyakla moro ka moro ibiro tim kod jal ma tayo twak ma otiegi e kama opondo mondo orit maling’ling’mari . E wi mano, wabiro kwayo thuolo mari mondo wamak weche ma iwacho e twak , ma bang’mano to wabiro ndiko kendo ng’iyo. Wach moro amora ma nyalo fwenyi ok bi bedo e ndiko ma ingíyo godo twak. Wabiro geno ni twak ma kanyakla biro bedo kuom saa achiel gi nus.
- **Rach.** Nitie thuolo matin mar fwenyruok ma chal mari mar kute mag ayaki. Kata kamano, wabiro keto chenro mag rito weche kuom chal mari mar kute mag ayaki.
- **Ber**. Ok inyal yudo ber mochimore kodi tir kuom bedo e nonro ni. Kuom bedo e nonro ni inyalo chiwo e gigo ma itimo mondo oket ngima mar rowere ma odak gi kute mag ayaki mondo obed maber.
- **Yiero mamoko.** In thuolo mar yiero mondo kik ibed e twak. Ka iyiero mondo kik ibed e nonro ni , onge kum ma ibiro miyi. Ok ibi wito ber moro amora ma iyudo ga mapile , kendo inyalo podi yudo thieth e klinik mari kaka itimo ga.

**Chenro kod gik ma itimo**

Ka janyuol kata jarit mari kod in uyie mar bedo , gigi biro timore:

Wabiro kwayi mondo iket seyi kata iket ranyisi e andike ni kaka ranyisi ni iyie kuom chiwruok mari mar bedo e nonro ni .

Wabiro chiki saa ma ibiro bedo gi rowere kod jamdongo matindo mamoko mondo utwag kuom yo maber mogik ma inyalo tim godo chenro ma konyo go. Ibiro bedo e twak ma ngíyo achiel kuom yore mag konyo ma ochan go, konyo e yor kompyuta (ote machwok ma ioro e yor kompyuta kod kony ma ichiwo gi mbese e yor sime), kony ma ichiwo gi mbese ka gibiro iri giwegi, kata pes ama ichiwo ka itimo gima idwaro (yudo pesa).

Ibiro penji penjo kuom gik ma ineno kod pek ma ikalo e thieth mar kute mag ayaki , ma luwore machalo nadi kod kit keny ma dwarore , kod yo maber mogik ma inyalo tim godo yore mag konyo go.

**Pachi kod weche ma ichiwo biro konyo e loso bura mar loso chenro ma iluongo ni design workshop.**

Twak ma itayo gi jal ma tayo twak ma otiegi e kama opondo mondo orit maling’ling’mari.

Bende wabiro kwayi mondo iyienwa wamak twak kod dwoko magi mondo omi wandike bang’e kendo wang’iye.

Wach moro amora ma nyalo fwenyi ok bi riw e weche ma ing’iyo go.

Twak ni igeno ni biro bedo kuom saa achiel gi nus.

**Inyalo kwayi e thuolo mabiro mondo ibed e twak mar jumaa achiel. Yie inyiswa ka idwaro bedo e twak no.**

**Rach**

Nitie thuolo matin mar ji ngéyo chal ni mar kute mag ayaki ka ok ochan. Kata kamano, wabiro keto chenro ma biro rito weche kuom chal mari mar kute mag ayaki.

**Ber**

Nyalore ni ok ibi yudo ber moro amora mochimore kodi tir kuom bedo e nonro ni. Kata kamano, bedo mari nyalo konyo keto maber bedo mar rowere e thieth.

**Dwoko pesa**

**Ibiro dwokni pesa wuoth mar silings ma Kenya 500/=.**

**Yiero mamoko**

In thuolo mar yiero mondo kik ibed e nonro. Ka iyiero mondo kik ibed e nonro ni, onge kum ma ibiro miyi. Ok ibi wito moro amora kuom ber ma iyudo ga pile, kendo podi inyalo yudo thieth mari kowuok e klinik mari kaka ijayudo ga.

**Maling’ling’**

Wabiro timo kar nyalowa mondo wane ni weche gi manyalo fweny I ma ochok ne nonro ni oket mopondo . Kata kamano, ok wanyal singo maling’ling’ma chutho . Weche gi manyalo fwenyi inyalo chiw ka idwaro e chik. Ka weche ma owuok e nonro ni ogo chapa kata osom e buche mag sayans, nyingi kod weche gi mamoko ma nyalo fwenyi ok bi ti godo .

Jotij sirkal kata mbalariany seche moko ng’iyo ga nonro kaka ma e mondo gine ni itimo gi e yo makare kendo kaluwore gi chik. Ka ng’iyo nonro ni otimore , andike gi ibiro ng’i. Jogo ma ng’iyo nonro gi biro rito maling’ling’mari. Andike mag nonro ok bi ti godo e kelo ni rach e yor chik. Migepe ma nyalo ng’iyo kendo/kata kopo andike gi mag nonro ne nonro, ngíyo kaka itimo tich , kod ng’inyo weche mochok oriwo:

- The Washington University, St. Louis
- The Kenya Medical Research Institute
- University of California San Francisco
- University of Colorado, Denver
- University of California, Berkeley

Bedo e nonro nyalo bedo gi wito maling’ling’, kata kamano wabiro keto yore mag rito maling’ling’mari . Wach moro amora momedore kuom nonro ibiro miyi koriwo nyaka dwok mogik mag nonro.

**Bedo e nonro**

Inyalo yiero mondo iwuog e twak e saa asaya , nyis anyisa jatij nonro kendo gibiro goli. E wi mano, jotij nonro nyalo goli, ka gineno ni in kuom ber mari mogik. Ok bi miyi kum e yo moro amora ka di po ni iyiero mar wuok.

**Joma ochiwo pesa**

Nonro ni ochung’ne gi National Institutes of Health. Onge moro amora kom jotim nonro man malo kanyo man kod chachni moro amora motudore gi yore pesa ma miyo dwaro mar gi nyalo chachni gi nonro. E thuolo ma nonro nyalo bedo ni ochung kowuok kuom yiero mar jatim nonro kata jachiw pesa, ibiro nyisi gima biro timore tok mano.

**Yor tudruok**

Inyalo wuoyo kod jotim nonro kuom penjo, parruok, kata ywagruok moro amora ma in go kuom nonro ni. Tudri kod jatij wa mar nonro e 0733-819 835. Ka diher mar penjo penjo kuom nonro kata ratiro magi kaka jachiwre ne ng’ato machielo kopogore gi jotim nonro kata diher mar wacho chandruok kata parruok moro amora ma in go kuom nonro , inyalo tudri gi Jagoro mar Ethical Review Committee, Kenya Medical Research Institute e seru@kemri.org kata P.O. BOX 54840-00200 Sime. 020-2722541, 0717719477. Bende inyalo tudri kod Washington University Human Research Protection Office e +1-314-747-6800.Komiti gi odich gi rito jochiwre mag nonro.

**YIE**

Osemiyi kopi mar andike mar yie ni mondo ikan.

**BEDO E NONRO EN KUOM CHIWRUOK.** In gi ratiro mar weyo bedo e nonro ni, kata wuok e saa asaya maonge kum moro amora kata wito ber ma dine iyudo.

**Wach ma oa kuom Jachiwre**

Nonro ni ma owuo kuome malo kanyo oselerna. Achiwora mar bedo e nonro ni. Aseyudo thuolo mar penjo penjo. Ka an gi penjo e kinde mabiro kuom nonro ni, anyalo penjo jotim nonro ma ondik malo kanyo. Ka an gi penjo kuom ratiro maga kaka jachiwre nonro, anyalo tudra kod jok ma ondik malo kanyo.

Ayie mondo weche maga ma ochok oti godo e nonro ma kinde mabiro kendo ochiw ne jotim nonro mamoko maonge yie machielo kod/maonge gigo manyalo fwenya .

Ka diher mar bedo e nonro ni, onego iket seyi piny kanyo.

Tarik	Nying kod Seyi Jachiwre Ne Yie

Tarik	Ng’at ma Kawo Yie (Nying gi seyi)

***Ka ok onyal somo kata ndiko***

Aseneno somo mar andike mar yie ni e yo ma kare ne jachiwre kendo ng’atno osebedo gi thuolo mar penjo penjo. Asiro ni ng’atno ochiwo yie mare ka en thuolo**.**

Nying janeno ____________________ KOD

Seyi janeno ***____________****_____*KOD

Nying jachiwre __________________AND

**Alama lith lwedo jachiwre**

**Tarik ________________________**

**Thuolo mar mako weche mag twak**

Achiwo thuolo ne twak mar kanyakla ma abiro bedo e mondo omak**: Ee ☐	Ooyo ☐**

**Thuolo mar mondo otudruok kodi ne twak mar jumaa achiel**

**Ayie mondo otudruok koda mondo abed e twak mar ndalo 3: Ee ☐	Ooyo ☐**

Kopi ne :	1. Jotim Nonro

2. Jachiwre nonro

**DHOLUO CONSENT FORM (FGD) 18-24 YEARS OLD**

**KENYA MEDICAL RESEARCH INSTITUTE**

**WASHINGTON UNIVERSITY, ST. LOUIS**

**YIE MAR BEDO E TWAK MAR KANYAKLA (FGD)**

**Study Title:** *Adaptive Strategies to Prevent and Treat Failures of Retention in Adolescent HIV Care (A4A Study)*

JOTIM NONRO KOD MIGEPE MA GITUDORE GODO:

| Jatim Nonro | Migao | Yor Tudruok |
| --- | --- | --- |
| Laktar Elizabeth Bukusi | Kenya Medical Research Institute | +254 733-617 503 |
| Laktar Elvin Geng | Washington University, St. Louis | +1-415-430-5589 (USA) |
| Laktar Lisa Abuogi | University of Colorado, Denver | +1-303-724-7430 (USA) |
| Laktar Zachary Kwena | Kenya Medical Research Institute | +254 722-386 714 |
| Starley Shade | University of California, San Francisco | +1-415-794-4354 (USA) |
| Lina Montoya | University of Notrh Carolina at Chapel Hill | +1-4105857470 (USA) |
| Eliud Akama | Kenya Medical Research Institute | +254 728-590 376 |

**Kama itimo e Nonro:** Kisumu County

Ikwayi mondo ichiwre ne bedo e twak mar kanyakla . Boksi man piny kanyo no nyisi gik mabe rma onego ipar kapok iyiero mar bedo e twak mar kanyakla ni . Wabiro chiwo wach matut e bwwo boksi man piny kanyo. Yie ipenj penjo kuom gimoro amora kapok iyiero ka ibedo kata ok ibed. Bende inyalo dwaro wuoyo gi jomoko (kuom ranyisi , jo anyuola u , osiepe, kata jotim nonro) kuom nonro ni , kapok iyie mar donjo.

- **Yie kuom Chiwruok**. Ikwayi mondo ichiwri mar bedo e twak mar kanyakla . En kuomi mar yiero ka idwaro bedo kata ok idwar . Onge kum kendo ok ibi wito gimoro amora ka iyiero ni ok idonj kata bang’e ka isedonjo, inyalo yiero mar wuok.

**Gima omiyo itime**. Watimo twak ma kanyakla adek mar choko pach rowere kod jomadongo matindo kuom yore ma ichano mag konyo rowere gi jomadongo matindo bedo e thieth mar kute mag ayaki . Chenro ma ochan go biro bedo gi kony mar mbese ma itimo e yor kompyuta kod ma itimo wang’gi wang’ (chal gi hocho) kod chiwo pesa . Pachi kod weche ma ichiwo biro konyo chiwo paro e romo ma kende kuom yore mag konyo go ma iluongo ni design workshop. Bende inyalo tudruok kodi kuom bedo e twak mar loso chenro e thuolo ma mbele.

- **Jochiwre:** Madirom rowere kod jomadongo matindo 24, man e kind higni 14 – 24, kendo odak gi kute mag ayaki e Kisumu county biro bedo e twak mag kanyakla. Wabiro bedo gi grube 3 kod ji madirom 8 e grup ka grup.
- **Chenro kod gik ma itimo.** Mokwongo**,** wabiro kwayo janyuolni kata jarit ni ka oyie mondo idonj . Ka ok oyie , to ok inyalo donjo. Ka uduto uyie , wabiro keto saa ne in mar bedo e twak mar kanyakla. Twak biro chimo achiel kuom yore konyo adek ma ochan mar konyo rowere kod jamadongo matindo medo edo e thieth mar kute mag ayaki. Twak mar kanyakla moro ka moro ibiro tim kod jal ma tayo twak ma otiegi e kama opondo mondo orit maling’ling’mari . E wi mano, wabiro kwayo thuolo mari mondo wamak weche ma iwacho e twak , ma bang’mano to wabiro ndiko kendo ng’iyo. Wach moro amora ma nyalo fwenyi ok bi bedo e ndiko ma ingíyo godo twak. Wabiro geno ni twak ma kanyakla biro bedo kuom saa achiel gi nus.
- **Rach.** Nitie thuolo matin mar fwenyruok ma chal mari mar kute mag ayaki. Kata kamano, wabiro keto chenro mag rito weche kuom chal mari mar kute mag ayaki.
- **Ber**. Ok inyal yudo ber mochimore kodi tir kuom bedo e nonro ni. Kuom bedo e nonro ni inyalo chiwo e gigo ma itimo mondo oket ngima mar rowere ma odak gi kute mag ayaki mondo obed maber.
- **Yiero mamoko.** In thuolo mar yiero mondo kik ibed e twak. Ka iyiero mondo kik ibed e nonro ni , onge kum ma ibiro miyi. Ok ibi wito ber moro amora ma iyudo ga mapile , kendo inyalo podi yudo thieth e klinik mari kaka itimo ga.

**Chenro kod gik ma itimo**

Ka iyie , gik ma oluwo gi biro timore:

1. Wabiro kwayi mondo iket seyi kata ranyisi e andike ni kaka ranyisi ni iyie kuom chiwruok mari mondo ibed e nonro .
2. Wabiro keto saa ma ibiro bedo e kanyakla mar rowere kod jomadongo matindo mamoko mondo utwag yo maber mogik ma inyalo tim go chenro mag konyo go.
3. Ibiro bedo e twak ma ngíyo achiel kuom yore mag konyo ma ochan go, konyo e yor kompyuta (ote machwok ma ioro e yor kompyuta kod kony ma ichiwo gi mbese e yor sime), kony ma ichiwo gi mbese ka gibiro iri giwegi, kata pes ama ichiwo ka itimo gima idwaro (yudo pesa).
4. Ibiro penji penjo kuom gik ma ineno kod pek ma ikalo e thieth mar kute mag ayaki , ma luwore machalo nadi kod kit keny ma dwarore , kod yo maber mogik ma inyalo tim godo yore mag konyo go.
5. **Pachi kod weche ma ichiwo biro konyo e loso bura mar loso chenro ma iluongo ni design workshop.**
6. Twak ma itayo gi jal ma tayo twak ma otiegi e kama opondo mondo orit maling’ling’mari.
7. Bende wabiro kwayi mondo iyienwa wamak twak kod dwoko magi mondo omi wandike bang’e kendo wang’iye.
8. Wach moro amora ma nyalo fwenyi ok bi riw e weche ma ing’iyo go.
9. Twak ni igeno ni biro bedo kuom saa achiel gi nus.
10. **Inyalo kwayi e thuolo mabiro mondo ibed e twak mar jumaa achiel. Yie inyiswa ka idwaro bedo e twak no.**

**Rach**

Nitie thuolo matin mar ji ngéyo chal ni mar kute mag ayaki ka ok ochan. Kata kamano, wabiro keto chenro ma biro rito weche kuom chal mari mar kute mag ayaki.

**Ber**

Nyalore ni ok ibi yudo ber moro amora mochimore kodi tir kuom bedo e nonro ni. Kata kamano, bedo mari nyalo konyo keto maber bedo mar rowere e thieth.

**Dwoko pesa**

**Ibiro dwokni pesa wuoth mar silings ma Kenya 500/=.**

**Yiero mamoko**

In thuolo mar yiero mondo kik ibed e nonro. Ka iyiero mondo kik ibed e nonro ni, onge kum ma ibiro miyi. Ok ibi wito moro amora kuom ber ma iyudo ga pile, kendo podi inyalo yudo thieth mari kowuok e klinik mari kaka ijayudo ga.

**Maling’ling’**

Wabiro timo kar nyalowa mondo wane ni weche gi manyalo fweny I ma ochok ne nonro ni oket mopondo . Kata kamano, ok wanyal singo maling’ling’ma chutho . Weche gi manyalo fwenyi inyalo chiw ka idwaro e chik. Ka weche ma owuok e nonro ni ogo chapa kata osom e buche mag sayans, nyingi kod weche gi mamoko ma nyalo fwenyi ok bi ti godo .

Jotij sirkal kata mbalariany seche moko ng’iyo ga nonro kaka ma e mondo gine ni itimo gi e yo makare kendo kaluwore gi chik. Ka ng’iyo nonro ni otimore , andike gi ibiro ng’i. Jogo ma ng’iyo nonro gi biro rito maling’ling’mari. Andike mag nonro ok bi ti godo e kelo ni rach e yor chik. Migepe ma nyalo ng’iyo kendo/kata kopo andike gi mag nonro ne nonro, ngíyo kaka itimo tich, kod ng’inyo weche mochok oriwo:

- The Washington University, St. Louis
- The Kenya Medical Research Institute
- University of California San Francisco
- University of Colorado, Denver
- University of California, Berkeley

Bedo e nonro nyalo bedo gi wito maling’ling’, kata kamano wabiro keto yore mag rito maling’ling’mari . Wach moro amora momedore kuom nonro ibiro miyi koriwo nyaka dwok mogik mag nonro.

**Bedo e nonro**

Inyalo yiero mondo iwuog e twak e saa asaya , nyis anyisa jatij nonro kendo gibiro goli. E wi mano, jotij nonro nyalo goli, ka gineno ni in kuom ber mari mogik. Ok bi miyi kum e yo moro amora ka di po ni iyiero mar wuok.

**Joma ochiwo pesa**

Nonro ni ochung’ne gi National Institutes of Health. Onge moro amora kom jotim nonro man malo kanyo man kod chachni moro amora motudore gi yore pesa ma miyo dwaro mar gi nyalo chachni gi nonro. E thuolo ma nonro nyalo bedo ni ochung kowuok kuom yiero mar jatim nonro kata jachiw pesa, ibiro nyisi gima biro timore tok mano.

**Yor tudruok**

Inyalo wuoyo kod jotim nonro kuom penjo, parruok, kata ywagruok moro amora ma in go kuom nonro ni. Tudri kod jatij wa mar nonro e 0733-819 835. Ka diher mar penjo penjo kuom nonro kata ratiro magi kaka jachiwre ne ng’ato machielo kopogore gi jotim nonro kata diher mar wacho chandruok kata parruok moro amora ma in go kuom nonro , inyalo tudri gi Jagoro mar Ethical Review Committee, Kenya Medical Research Institute e seru@kemri.org kata P.O. BOX 54840-00200 Sime. 020-2722541, 0717719477. Bende inyalo tudri kod Washington University Human Research Protection Office e +1-314-747-6800.Komiti gi odich gi rito jochiwre mag nonro.

**YIE**

Osemiyi kopi mar andike mar yie ni mondo ikan.

**BEDO E NONRO EN KUOM CHIWRUOK.** In gi ratiro mar weyo bedo e nonro ni, kata wuok e saa asaya maonge kum moro amora kata wito ber ma dine iyudo.

**Wach ma oa kuom Jachiwre**

Nonro ni ma owuo kuome malo kanyo oselerna. Achiwora mar bedo e nonro ni. Aseyudo thuolo mar penjo penjo. Ka an gi penjo e kinde mabiro kuom nonro ni, anyalo penjo jotim nonro ma ondik malo kanyo. Ka an gi penjo kuom ratiro maga kaka jachiwre nonro, anyalo tudra kod jok ma ondik malo kanyo.

Ayie mondo weche maga ma ochok oti godo e nonro ma kinde mabiro kendo ochiw ne jotim nonro mamoko maonge yie machielo kod/maonge gigo manyalo fwenya .

Ka diher mar bedo e nonro ni, onego iket seyi piny kanyo.

Tarik	Nying kod Seyi Jachiwre Ne Yie

Tarik	Ng’at ma Kawo Yie (Nying gi seyi )

***Ka ok onyal somo kata ndiko***

Aseneno somo mar andike mar yie ni e yo ma kare ne jachiwre kendo ng’atno osebedo gi thuolo mar penjo penjo. Asiro ni ng’atno ochiwo yie mare ka en thuolo**.**

Nying janeno _____________________ KOD

Seyi janeno *__________________*KOD

Nying jachiwre __________________KOD

**Alama lith lwedo mathuon mar jachiwre**

**Tarik ________________________**

**Thuolo mar mako weche mag twak**

Achiwo thuolo ne twak mar kanyakla ma abiro bedo e mondo omak**: Ee ☐	Ooyo ☐**

**Thuolo mar mondo otudruok kodi ne twak mar jumaa achiel**

**Ayie mondo otudruok koda mondo abed e twak mar ndalo 3: Ee ☐	Ooyo ☐**

Kopi ne:	1. Jotim nonro

2. Jachiwre nonro

**Study Title: *Adaptive Strategies to Prevent and Treat Failures of Retention in Adolescent HIV Care (A4A Study)***

**LUO PARENTAL CONSENT FORM**

**KENYA MEDICAL RESEARCH INSTITUTE**

**WASHINGTON UNIVERSITY, ST. LOUIS**

**YIE MAR JANYUOL NE TWAK MAR KANYAKLA (FGD)**

JOTIM NONRO KOD MIGEPE MA GITUDORE GODO:

| Jatim Nonro | Migao | Yor Tudruok |
| --- | --- | --- |
| Laktar Elizabeth Bukusi | Kenya Medical Research Institute | +254 733-617 503 |
| Laktar Elvin Geng | Washington University, St. Louis | +1-415-430-5589 (USA) |
| Laktar Lisa Abuogi | University of Colorado, Denver | +1-303-724-7430 (USA) |
| Laktar Zachary Kwena | Kenya Medical Research Institute | +254 722-386 714 |
| Starley Shade | University of California, San Francisco | +1-415-794-4354 (USA) |
| Lina Montoya | University of Notrh Carolina at Chapel Hill | +1-4105857470 (USA) |
| Eliud Akama | Kenya Medical Research Institute | +254 728-590 376 |

**Kama itimo e nonro :** Kisumu County

Ikwayi mondo iyie nyathini obed e nonro ma oriwo bedo e twak mag kanyakla. Boksi man piny no nyisi gik ma onego ipar kapok iyiero mar yie ne nyathini mondo odonj e twak. Wabiro chiwo wach matut e bwo boksi no. Yie ipenj penjo kuom wach moro amora kapok iyiero mar yie ne nyathini mondo obed kata kik obed twak. Bende inyalo hero mar wuoyo kod jomamoko (kuom ranyisi , anyuola ni , osiepe, kata jotim nonro) kuom nonro ni , kapok iyie mondo odonj.

- **Yie kuom chiwruok**. Ikwayi mondo iyie ne nyathini obed e twak mar kanyakla . En kuomi mondo ka iyiero mar yie ne nyathini mondo obed kata kik obed e twak. Onge kuom ne in kendo ok ibi wito gimoro amora ka iyiero mar ni ok idwar ni nyathini obedi e twak.
- **Gima omiyi itime**. Watimo twak mag kanyakla adek mondo wachok pach rowere kod joamdongo matindo kuom chenro ma ochan keto mag konyo rowere kod jomadongo matindo medo bedo e thieth mar kute mag ayaki. Chenro ma ochan go biro bedo gi kony mar mbese ma itimo e yor kompyuta kod ma itimo wang’gi wang’ (chal gi hocho) kod chiwo pesa . Pach nyathini kod weche ma ochiwo biro konyo chiwo wach ma ibiro ti godo e romo ma kende kuom chenro go ma iluongo ni design workshop. Inyalo tudruok gi nyathini kuom bedo e design workshop e thuolo ma bangé.
- **Jochiwre:** Madirom rowere kod jomadongo matindo 24, man e kind higni 14 – 24, kendo odak gi kute mag ayaki e Kisumu county biro bedo e twak mag kanyakla. Wabiro bedo gi grube 3 kod ji madirom 8 e grup ka grup.

**Chenro kod gik ma itimo.** E nonro ni , ka iyie mar mondo nyathini obed e twak mar kanyakla , bende wabiro kwayi nyathini ka oyie. Ka uduto uyie , wabiro keto saa saa ma nyathini biro bedo e twak mar kanyakla. Twak biro ng’iyo achiel kuom yore ma ochan mondo okony keto rowere kod jomadongo matindo omed bedo e thieth mar kute mag ayaki. Twak ka twak ibiro tim kod ngát ma tayo twak ma otiegi e kama opondo mondo orit maling’ling’mar nyathini . E win mano, wakwayo thuolo mondo iyie wamak weche ma iwacho e twak , ma wabiro ndiko piny kendo ng’iyo. Wach moro amora ma nyalo fwenyo nyathini ok bi riw e weche ma ing’iyo gi. Wageno ni twak mag kanyala go biro bedo kuom saa achiel gi nus.

- **Rach.** Nitie thuolo matin mar fwenyruok ma chal mar kute mag ayaki. Kata kamano, wabiro keto chenro mag rito wach kuom chal mar nyathini mar kute mag ayaki.
- **Ber**. Nyalore ni nyathini ok bi yudo ber mochimore kode tir kuom bedo e nonro ni. Kuom bedo e nonro ni okonyo e jiwo yore ma gero ngima mag rowere ma odak kod kute mag ayaki .
- **Yiero mamoko.** In thuolo mar yiero mondo kik iyie ni nyathini mondo obed e twak. Ka iyiero ni nyathini kik bed e nonro ni , onge kum ma ibiro miye . Ok obi wito ber ma ojayudo , kendo podi onyalo yudo thieth mare e klinik kaka oyudo ga .

**Chenro kod gik ma itimo**

Ka iyie mar chiwo thuolo ne nyathini mondo odonj, kendo nyathini bende oyie, gik moluwo gi biro timore:

1. Wabiro kwayi mondo iket seyi kata alama e andike ni kaka ranyisi ni kuom chiwruok ni iyie mar bedo e nonro kendo mondo wakwa nyathini thuolo.
2. Wabiro keto thuolo mondo obed gi rawera mamoko mondo gitwag kuom yo maber mogik ma inyalo tim godo chenro mag nonro mag konyo ma ochan.
3. Nyathini biro bedo e twak ma ngíyo achiel kuom yore adek ma ochan mag konyo , konyo ji e yor sime/kompyuta (ote machwok ma ioro gi kompyuta kod siro ma itimo gi mbese e yor sime), kony ma ichiwo gi mbese ka gibiro iri giwegi, kata pesa ma ichiwo ka itimo gima idwaro (yudo pesa).
4. Ibiro penje penjo kuom gik ma oseneno kod pek ma osebedo godo e thieth mar kute mag ayaki , moluwore machalo nade kod kit kony ma darore , gi paro kuom kaka inyalo tim chenro mochan go.
5. **Pache kod weche ma ochiwo biro konyo loso bura ma ilos go chenro mar konyo ma iluongo ni .**
6. Twak ma itayo gi jal ma tayo twak ma otiegi e kama opondo mondo orit maling’ling’.
7. Wabiro kwaye mondo oyienwa wamak twak kod dwoko mage mondo wandik gi piny kendo wang’i gi bang’e .
8. Wach moro amora ma nyalo fwenyo nyathini ok bi bedo e ndiko ma ing’iyo no.
9. Twak ni igeno ni biro kawo madirom saa achiel gi nus .
10. **Nyathini inyalo kwa e thuolo mabiro mondo obed e twak mar jumaa achiel. Nyiswa mondo wangé ka ibiro dwaro weyo nyathini mondo obed e twag no.**

**Rach**

Nitie thuolo matin mar fwenyruok ma chal mar kute mag ayaki . Kata kamano, wabiro keto chenro mag neno ni maling’ling’orit weche kuom chal mar nyathini mar kute mag ayaki.

**Ber**

Nyalore ni nyathini ok nyalo yudo ber mochimore kode tir kuom bedo e nonro ni. Kata kamano, bedo mar nyathini nyalo keto maber medo bedo mar rowere e thieth.

**Dwoko pesa**

**Nyathini ibiro dwokni pesa mar wuoth mar silings ma Kenya 500/=.**

**Yiero mamoko**

In thuolo mar yiero mondo kik iyie ne nyathini odonj e nonro. Ka iyiero ni ok iyie mondo nyathini obed e nonro ni, onge kum ma ibiro miye . Ok obi wito moro amora kuom bero ma oyudo ga, kendo podi onyalo yudo thieth mare ka owok e klinik kaka oyudo ga.

**Maling’ling’**

Wabiro timo kar nyalo wa mondo wane ni weche duto ma ochok ne nonro ni oket mopondo. Kata kamano, ok wanyal singo maling’ling’ma chutho. Weche nyathini ma nyalo fwenye inyalo chiw ka idwaro e yor chik. Ka weche ma owuok e nonro ni ogo chapa kata osom e buche mag sayans , nyingi kata nying nyathini kod weche mamoko ma nyalo fwenyo u ok bi ti godo.

Jotij sirkal kata mbalariany seche moko ng’iyo ga nonro kaka ma e mondo gine ni itimo gi e yo makare kendo kaluwore gi chik. Ka ng’iyo nonro ni otimore , andike gi ibiro ng’i. Jogo ma ng’iyo nonro gi biro rito maling’ling’mari. Andike mag nonro ok bi ti godo e kelo ni rach e yor chik. Migepe ma nyalo ng’iyo kendo/kata kopo andike gi mag nonro ne nonro, ngíyo kaka itimo tich, kod ng’inyo weche mochok oriwo:

- The Washington University, St. Louis
- The Kenya Medical Research Institute
- University of California San Francisco
- University of Colorado, Denver
- University of California, Berkeley

Bedo e nonro nyalo bedo gi wito maling’ling’, kata kamano wabiro keto yore mag rito maling’ling’mari. Wach moro amora momedore kuom nonro ibiro miyi koriwo nyaka dwoko mogik mag nonro.

**Bedo e nonro**

Nyathini nyalo yiero mar weyo twak e saa asaya , nyisi anyisa jatij nonro kendo gibiro gole. E wi mano, jatij nonro nyalo gole e nonro, ka gineno in en kuom ber mare. Ok be miye kum e yo moro amora ka oyiero mondo owe bedo e nonro.

**Joma ochiwo pesa**

Nonro ni ochung’ne gi National Institutes of Health. Onge moro amora kom jotim nonro man malo kanyo man kod chachni moro amora motudore gi yore pesa ma miyo dwaro mar gi nyalo chachni gi nonro. E thuolo ma nonro nyalo bedo ni ochung kowuok kuom yiero mar jatim nonro kata jachiw pesa, ibiro nyisi gima biro timore tok mano.

**Yor tudruok**

Inyalo wuoyo kod jotim nonro kuom penjo, parruok, kata ywagruok moro amora ma in go kuom nonro ni. Tudri kod jatij wa mar nonro e 0733-819 835. Ka diher mar penjo penjo kuom nonro kata ratiro magi kaka jachiwre ne ng’ato machielo kopogore gi jotim nonro kata diher mar wacho chandruok kata parruok moro amora ma in go kuom nonro , inyalo tudri gi Jagoro mar Ethical Review Committee, Kenya Medical Research Institute e seru@kemri.org kata P.O. BOX 54840-00200 Sime. 020-2722541, 0717719477. Bende inyalo tudri kod Washington University Human Research Protection Office e +1-314-747-6800.Komiti gi odich gi rito jochiwre mag nonro.

**YIE**

Osemiyi kopi mar andike mar yie ni mondo ikan.

**BEDO E NONRO EN KUOM CHIWRUOK.** In gi ratiro mar weyo bedo e nonro ni, kata wuok e saa asaya maonge kum moro amora kata wito ber ma dine iyudo..

**Wach ma oa kuom jachiwre**

Oselerna wach kuom nonro ma ondik mao kanyo . Ayie mar chiwo thuolo mondo nyathina obed e nonro ni. Asebedo gi thuolo mar penjo penjo. Ka an gi penjo e kinde mabiro kuom nonro , anyalo penjo achiel kuom jotim nonro mondik malo kanyo . Ka an gi penjo kuom ratiro maga kaka jachiwre nonro, anyalo tudra gi jogo ma ondik malo kanyo.

Ayie ni weche ma ochok kuom nyathina mondo oti godo ne nonro ma kinde mabiro kendo ochiw ne jotim nonro mamoko maonge yie machielo mara ka en gi weche ma fwenye kata ma ok nyal fwenye .

Ka diher mar yie ne nyathini mondo obed e nonro ni, onego iket seyi piny kanyo.

Tarik	Nying Jachiwre

Tarik	Nying gi Seyi Jarit/Janyuol ne Yie

Tarik	Ng’ama Kawo Yie (Nying kod seyi )

***Ka ok onyal somo kata ndiko***

Aseneno somo mar andike mar yie ni e yo ma kare ne jachiwre kendo ng’atno osebedo gi thuolo mar penjo penjo. Asiro ni ng’atno ochiwo yie mare ka en thuolo**.**

Nying janeno _______________________ KOD

Seyi janeno ____________________KOD

Nying Jarit/Janyuol _______________KOD

Alama lithlwedo ma Thuon

**Tarik ________________________**

**Thuolo mar mako twak**

Achiwo thuolo mar bedo e twak mar kanyakla ma nyathina biro bedo e mondo omaki **: Ee ☐	Ooyo ☐**

**Thuolo mar tudruok kode ne twak mar jumaa achiel**

**Ayie mondo nyathina obe otudruok godo mondo obed e twak mar ndalo 3:**

**Ee ☐	Ooyo ☐**

Kopi ne :	1. Jotim Nonro

2. Jachiwre nonro

**KISWAHILI ASSENT FORM (FGD) 14-17 YEARS OLD**

**KENYA MEDICAL RESEARCH INSTITUTE**

**WASHINGTON UNIVERSITY, ST. LOUIS**

**ASSENT TO PARTICIPATE IN FOCUS GROUP DISCUSSION (FGD)**

WATAFITI NA TAASISI SHIRIKISHI:

| Mtafiti | Taasisi | Mawasiliano |
| --- | --- | --- |
| Dkt. Elizabeth Bukusi | Kenya Medical Research Institute | +254 733-617 503 |
| Dkt. Elvin Geng | Washington University, Missouri | +1-415-430-5589 (USA) |
| Dkt. Lisa Abuogi | University of Colorado, Denver | +1-303-724-7430 (USA) |
| Dkt. Zachary Kwena | Kenya Medical Research Institute | +254 722-386 714 |
| Starley Shade | University of California, San Francisco | +1-415 794-4354 (USA) |
| Lina Montoya | University of Notrh Carolina at Chapel Hill | +1-4105857470 (USA) |
| Eliud Akama | Kenya Medical Research Institute | +254 728-590 376 |

**Eneo la utafiti:** Kisumu County

Tungependa kukueleza kuhusu kitu yenye tunafanya inayojulikana kama utafiti . Utafiti ni wakati daktari anakusanya habari kujifunza zaidi kuhusu kitu. Tunakuomba ujitolee kushiriki kwa majadiliano ya kikundi ilyosehemu ya utafiti yenye tunafanya . Unaombwa kujitolea kushiriki kwa majadiliano ya kikundi. Sanduku iliyoko hapo chini inakueleza kuhusu mambo muhimu unayohitaji kufikira kabla ya kuamua kushiriki utafiti. Tutakupa habari zaidi chini ya sanduku hilo. Tafadhali uliza maswali kuhusu habari yoyote kabla ya kuamua ikiwa utashiriki au la. Pia ungependa kuzungumza na wengine (kwa mfano, familia yako, marafiki, au daktari wako) kuhusu utafiti, kabla ya kuamua kushiriki.

- **Idhini kwa hiari**. Unaulizwa kujitolea kushiriki kwa majadiliano ya kikundi. Ni juu yako kuamua kushiriki au la. Hakutakuwa na adhabu na hautapoteza chochote ikiwa umeamua kutojiunga au baada ya kijiunga, umeamua kujiondoa.
- **Lengo**. Tunafanya majadiliano ya vikundi tatu kukusanya fikira za vijana na vijana wenye wamebaleghe kuhusu mikakati uya kuingilia kati zilizopangwa kusaidia vijana na vijana wenye wamebaleghe kundelea kuwa kwa matibabu ya virusi vya ukimwi . Mikakati yaliyopangwa yanahusisha usaidizi wa kielektroniki na kupitia mwanarika binafsi (inafanana na ushauri) na kupeana pesa. Maoni yako na habari zitasaidia kuarifu mkutano spesheli kuhusu mikakati ya kuingilia kati inayoitwa warsha ya kuunda (design workshop). Tunaweza kuwasiliana na wewe kuhusu kushiriki kwa warsha ya kuunda katika hatua ya baadaye.
- **Washiriki:** Takriban vijana na vijana waliyobaleghe 24, kati ya miaka 14 – 24, na wanaishi na virusi vya ukimwi katika Kauti ya Kisumu watashiriki kwa majadiliano ya vikundi . Kutakuwa na vikundi 3 yenye watu 8 kwa kila kikundi.
- **Taratibu na shughuli.** Ikiwa umekubali kushiriki, tutatenga muda yaw ewe kujiunga na majadiliano ya kikundi. Majadiliano yataangazia moja kati ya mikakati ya kuingilia kati yaliyopangwa tatu kusaidia kuweka vijananna vijana wenye wamebaleghe kwa matibabu ya virusi vya ukimwi. Kila jadiliano ya kikundi itafanywa na msimamzi aliyeelimishwa kwa pahala pa kibinafsi ili kulinda usiri wako. Zaidi ya hayo, tutakuomba ruhusa ya kurekodi mahojiano, ambacho baadaye tutaandika na kuyaangalia. Habari yoyote inayoweza kukutambua hayatajumuishwa kwa uangali hiyo. Tunatarajia majadiliano ya kikundi kuchukua muda wa saa moja na nusu.
- **Hatari.** Kuna hatari kidogo ya kufichuliwa kwa hali ya virusi vya ukimwi. Walakini, tutaweka mikakati ya kulinda habari kuhusu hali yako ya virusi vya ukimwi.
- **Faida**. Kuna uwezekano kuwa hutapata faida zozote kutokana na kushiriki kwa utafiti huu. Kwa kushiriki kwa utafiti huu unaweza kuchangia juhudi za kuinua afya ya vijana wanaoishi na virusi vya ukimwi.
- **Chaguo mbadala.** Uko huru kuamua kustoshiriki kwa majadiliano. Ukiamua kutoshiriki kwa utafiti huu, hakutakuwa na adhabu kwako. Hautapoteza faida zako za kawaida, na bado unaweza kupokea matibabu kwa kliniki yako jinsi umekuwa ukipokea.

**Taratibu na shughuli**

Ikiwa umekubali, vitu vufuatazo zitafanyika:

1. Tutakuomba uweke sahihi au uweke alama kwenye fomu hii kama ishara ya kuwa kwa hiari umekubali kushiriki kwa huu utafiti .
2. Tutaweka saa ya kujiunga na kikundi cha vijana na vijana wenye wamebaleghe kujdili njia bora zaidi ya kutekeleza mikakati ya kuingilia kati yenye yamepangwa.
3. Utakuwa sehemu ya majadiliano yanayoangazia moja kati ya mikakakti tatu yaliyopangwa , kwa njia ya usaidizi wa kielektroniki (arafa fupi zinazotumwa kwa njia ya kompyuta na usaidizi ya wanarika kwa njia ya simu), usaidizi wa ana kwa ana na mawanarika , au kupeana pesa ukitii marsharti (kupokea pesa).
4. Utaulizwa maswali kuhusu uliyokumbana nayo na changamoto na matibabu ya virus vya ukimwi , ni mara ngapi nan i aina gani ya usaidizi inahitajika , na pendekezo kuhusu njia gani bora kutekeleza mikakati ya kuingilia kati .
5. **Maoni yako na habari yatatusaidia kujulisha mkutano wa mikakati inayohitwa warsha ya kuunda (design workshop.)**
6. Majadiliano yataendeshwa na msamizi aliyehitimu kwenye chumba cha kibinafsi kulinda usiri wako.
7. Pia tutauliza ikiwa utaturuhusu kurekodi mahojiano na maajibu zako ili tuweze kuziandika chini alafu tuziangalie baadaye.
8. Ahabari yoyote yenye inaweza kukutambua haitajumuishwa kwa uangalizi .
9. Majadialiano yanatarajiwa kuchukua takriban saa moja na nusu .
10. **Unaweza kuulizwa tarehe ya badaye ushiriiki kwa warsha ya wiki moja. Tujulishe ikiwa utaweza kushiriki.**

**Hatari**

Kuna nafasi kidogo ya kufichuliwa kiajali hali ya virusi vya ukimwi. Walakini, tutaweka mikakati ya usalama ya kulinda hali yako ya virusi vya ukimwi.

**Faida**

Kuna uwezekano hutapokea faida ya kibinafsi kutokana na kushiriki kwa utafiti huu. Walakini, kushirkik kwako inaweza kuimarisha uhifadhi wa vijana kwa matibabu.

**Kurudishiwa pesa**

**Utarudishiwa pesa ya nauli ya shilingi ya Kenya 500/=.**

**Chaguo mbadala**

Uko huru kuamua kutoshirki kwa utafiti. Ikiwa umeamua kutoshiriki kwa utafiti huu, hakutakuwa na adhabu kwako. Hautapoteza faida zako zozote za kawaida, na pia badi unaweza kupata matibabu yako kutoka kwa kliniki jinsi umekuwa ukifanya.

**Usiri**

Tutafanya kadri ya uwezo wetu kuhakikisha habari za kibinfasi yalikusanywa kwa minajili ya utafiti imewekwa siri. Walakini, hatuwezi kuhakikisha usiri kamilifu. Habari zako za kibinfasi yanaweza kupeanwa ikihitajika kisheria. Ikiwa habari kutoka kwa utafiti huu umechapishwa au kusomwa kwenye mikutano ya sayansi, jina lako au habari zako zingine za kibinafsi hazitatumika.

Wafanyikazi wa serikali na vyuo vikuu wakati mwingine hungalia utafiti kama huu kuhakikisha yanafanywa kwa usalama na kulingana na sheria. Ikiwa utafiti huu umeangaliwa, rekodi zako zitachunguzwa. Wachunguzi hao watalinda ubinafsi wako. Rekodi za utafiti hazitatumika kukuweka kwa hatari ya kisheria. Mashirika yanaweza kuangalia na/au kunakili rekodi zako za utafiti kwa ajili ya utafiti, utenda kazi, na uchambuzi wa deta yanajumuisha:

- The Washington University, St. Louis
- The Kenya Medical Research Institute
- University of California San Francisco
- University of Colorado, Denver
- University of California, Berkeley

Kushiriki kwa utafiti unaweza kuwa na kupoteza ubinafsi , walakini tutaweka mikakati ya kulinda usiri wako. Habari yoyote ya ziada kuhusu utafiti utapewa ikujumuisha matokeo ya mwisho wa utafiti.

**Kushiriki**

Unaweza kuacha kushiriki kwa utafiti wakati wowote, ambia tu mfanyikazi wa utafiti na watakuondoa. Zaidi ya hayo, wafanyikazi wa utafiti wanaweza kukuondoa kwa utafiti, ikiwa wanahisi kwamba ni kwa maslahi yako. Hautaadhibiwa kwa njia yoyote ile kam ataamua kuacha kushiriki.

**Ufadhili**

Utafiti umefadhiliwa na National Institutes of Health. Hakuna yeyote kwa watafiti waliyo na mgongano wa maslahi ya kifedha. Katika hali yenye utafiti umekomeshwa kutokana na maamuzi ya mtafiti au mfadhili wa utafiti, utaelezwa kuhusu hatua ya kuchukiliwa baada ya hiyo.

**Mawasiliano**

Unaweza kuzungumza na m(wa)tafiti kuhusu maswali, wasiwasi, au malalamishi yoyote uliyonayo kuhusu utafiti huu. Wasiliana na mfanyikazi wetu wa utafiti katika 0733-819 835. Ikiwa ungependa kuuliza maswali kuhusu uatfiti au haki zako kama mshiriki wa utafiti kwa mtu mwingine kando ya watafiti au ukiwa ungependa kuzungumzia shida au wasiwasi ambazo uanweza kuwa nazo kuhusu utafiti, unaweza kuwasiliana na Katibu wa Ethical Review Committee, Kenya Medical Research Institute kwa seru@kemri.org au P.O. BOX 54840-00200 Tel. 020-2722541, 0717719477. Pa unaweza kuwasiliana na Washington University Human Research Protection Office kwa +1-314-747-6800.Haya kamati yanshughulika na kulinda haki za wanaojitolea kwa miradi za utafiti.

**IDHINI**

Umepewa nakala ya fomu hii ya idhini ili uweke.

**KUSHIRIKI KWA UTAFITI NI KWA HIARI.** Uko na haki ya kukataa kushiriki utafiti huu, au kujiondoa wakati wowote bila adhabu yoyote au kupoteza faida ambazo ungepata.

**Taarifa ya mshiriki**

Utafiti huu unaelezwa hapo juu nimeshaelezwa kuihusu. Najitolea kushiriki kwa utafiti huu. Nimekuwa na nafasi ya kuuliza maswali. Nikiwana maswali za siku zijazo kuhusu utafiti huu, ninaweza kuuliza watafiti waliondikwa hapo juu. Nikiwa na maswali kuhusu haki zangu kama mshiriki wa utafiti, ninaweza kuwasiliana na waliandikwa hapo juu.

Nimekubali habari zangu yatumike kwa minajili ya utafiti za siku zijazo na kupeanwa watafiti wengine bila ya kuongezea idhini na/bila vitambulishi.

Ikiwa ungependa kishiriki kwa utafiti huu, unafaa kuweka sahihi hapo chini.

Tarehe	Jina na Sahihi la Mshiriki kwa Idhini

Tarehe	Mtu Anayechukua Idhini (Jina na sahihi)

***Ikiwa hawezi kusoma na kuandika***

Nimeshuhudia kusomewa kwa njia sawa hii fomu ya idhini kwa mshiriki na mtu mwenyewe amekuwa na nafasi ya kuuliza maswali. Nathibitisha kuwa mtu mwenyewe amepeana idhini yaki akiwa huru**.**

Jina la shahidi ____________________ NA

Sahiha ya shahidi ***____________****_____*NA

Jina la mshiriki __________________NA

**Alama ya kidole gumba cha mshiriki**

**Tarehe ________________________**

**Ruhusa ya kurekodi majadiliano**

Ninatoa ruhusa kwa majadiliano ya kikundi yenye nitashiriki irekodiwe**:Ndio ☐	La ☐**

**Ruhusa ya kuwasiliana na mimi kuhusu warsha ya wiki moja**

**Nimekubali kutafutwa kushiriki kwa warsha ya siku 3: Ndio ☐	La ☐**

Nakala kwa:	1. Watafiti

2. Mshiriki wa utafiti

**Study Title:** *Adaptive Strategies to Prevent and Treat Failures of Retention in Adolescent HIV Care (A4A Study)*

**KISWAHILI CONSENT FORM (FGD) 18-24 YEARS OLD**

**KENYA MEDICAL RESEARCH INSTITUTE**

**WASHINGTON UNIVERSITY, ST. LOUIS**

**IDHINI YA KUSHIRIKI KWA MAJADILIANO YA KIKUNDI (FGD)**

WATAFITI NA TAASISI SHIRIKISHI:

| Mtafiti | Taasisi | Mawasiliano |
| --- | --- | --- |
| Dkt. Elizabeth Bukusi | Kenya Medical Research Institute | +254 733-617 503 |
| Dkt. Elvin Geng | Washington University, Missouri | +1-415-430-5589 (USA) |
| Dkt. Lisa Abuogi | University of Colorado, Denver | +1-303-724-7430 (USA) |
| Dkt. Zachary Kwena | Kenya Medical Research Institute | +254 722-386 714 |
| Starley Shade | University of California, San Francisco | +1-415 794-4354 (USA) |
| Lina Montoya | University of Notrh Carolina at Chapel Hill | +1-4105857470 (USA) |
| Eliud Akama | Kenya Medical Research institute | +254 728-590 376 |

**Eneo la utafiti :** Kisumu County

Unaombwa kujitolea kushiriki kwa majadiliano ya kikundi. Sanduku iliyoko hapo chini inakueleza kuhusu mambo muhimu unayohitaji kufikira kabla ya kuamua kushiriki utafiti. Tutakupa habari zaidi chini ya sanduku hilo. Tafadhali uliza maswali kuhusu habari yoyote kabla ya kuamua ikiwa utashiriki au la. Pia ungependa kuzungumza na wengine (kwa mfano, familia yako, marafiki, au daktari wako) kuhusu utafiti, kabla ya kuamua kushiriki.

- **Idhini kwa hiari**. Unaulizwa kujitolea kushiriki kwa majadiliano ya kikundi. Ni juu yako kuamua kushiriki au la. Hakutakuwa na adhabu na hautapoteza chochote ikiwa umeamua kutojiunga au baada ya kijiunga, umeamua kujiondoa.
- **Lengo**. Tunafanya majadiliano ya vikundi tatu kukusanya fikira za vijana na vijana wenye wamebaleghe kuhusu mikakati uya kuingilia kati zilizopangwa kusaidia vijana na vijana wenye wamebaleghe kundelea kuwa kwa matibabu ya virusi vya ukimwi . Mikakati yaliyopangwa yanahusisha usaidizi wa kielektroniki na kupitia mwanarika binafsi (inafanana na ushauri) na kupeana pesa. Maoni yako na habari zitasaidia kuarifu mkutano spesheli kuhusu mikakati ya kuingilia kati inayoitwa warsha ya kuunda (design workshop). Tunaweza kuwasiliana na wewe kuhusu kushiriki kwa warsha ya kuunda katika hatua ya baadaye.
- **Washiriki:** Takriban vijana na vijana waliyobaleghe 24, kati ya miaka 14 – 24, na wanaishi na virusi vya ukimwi katika Kauti ya Kisumu watashiriki kwa majadiliano ya vikundi . Kutakuwa na vikundi 3 yenye watu 8 kwa kila kikundi.
- **Taratibu na shughuli.** Ikiwa umekubali kushiriki, tutatenga muda yaw ewe kujiunga na majadiliano ya kikundi. Majadiliano yataangazia moja kati ya mikakati ya kuingilia kati yaliyopangwa tatu kusaidia kuweka vijananna vijana wenye wamebaleghe kwa matibabu ya virusi vya ukimwi. Kila jadiliano ya kikundi itafanywa na msimamzi aliyeelimishwa kwa pahala pa kibinafsi ili kulinda usiri wako. Zaidi ya hayo, tutakuomba ruhusa ya kurekodi mahojiano, ambacho baadaye tutaandika na kuyaangalia. Habari yoyote inayoweza kukutambua hayatajumuishwa kwa uangali hiyo. Tunatarajia majadiliano ya kikundi kuchukua muda wa saa moja na nusu.
- **Hatari.** Kuna hatari kidogo ya kufichuliwa kwa hali ya virusi vya ukimwi. Walakini, tutaweka mikakati ya kulinda habari kuhusu hali yako ya virusi vya ukimwi.
- **Faida**. Kuna uwezekano kuwa hutapata faida zozote kutokana na kushiriki kwa utafiti huu. Kwa kushiriki kwa utafiti huu unaweza kuchangia juhudi za kuinua afya ya vijana wanaoishi na virusi vya ukimwi.
- **Chaguo mbadala.** Uko huru kuamua kustoshiriki kwa majadiliano. Ukiamua kutoshiriki kwa utafiti huu, hakutakuwa na adhabu kwako. Hautapoteza faida zako za kawaida, na bado unaweza kupokea matibabu kwa kliniki yako jinsi umekuwa ukipokea.

**Taratibu na shughuli**

Ikiwa umekubali, vitu vufuatazo zitafanyika:

1. Tutakuomba uweke sahihi au uweke alama kwenye fomu hii kama ishara ya kuwa kwa hiari umekubali kushiriki kwa huu utafiti .
2. Tutaweka saa ya kujiunga na kikundi cha vijana na vijana wenye wamebaleghe kujdili njia bora zaidi ya kutekeleza mikakati ya kuingilia kati yenye yamepangwa.
3. Utakuwa sehemu ya majadiliano yanayoangazia moja kati ya mikakakti tatu yaliyopangwa , kwa njia ya usaidizi wa kielektroniki (arafa fupi zinazotumwa kwa njia ya kompyuta na usaidizi ya wanarika kwa njia ya simu), usaidizi wa ana kwa ana na mawanarika , au kupeana pesa ukitii marsharti (kupokea pesa).
4. Utaulizwa maswali kuhusu uliyokumbana nayo na changamoto na matibabu ya virus vya ukimwi , ni mara ngapi nan i aina gani ya usaidizi inahitajika , na pendekezo kuhusu njia gani bora kutekeleza mikakati ya kuingilia kati .
5. **Maoni yako na habari yatatusaidia kujulisha mkutano wa mikakati inayohitwa warsha ya kuunda (design workshop.)**
6. Majadiliano yataendeshwa na msamizi aliyehitimu kwenye chumba cha kibinafsi kulinda usiri wako.
7. Pia tutauliza ikiwa utaturuhusu kurekodi mahojiano na maajibu zako ili tuweze kuziandika chini alafu tuziangalie baadaye.
8. Ahabari yoyote yenye inaweza kukutambua haitajumuishwa kwa uangalizi .
9. Majadialiano yanatarajiwa kuchukua takriban saa moja na nusu .
10. **Unaweza kuulizwa tarehe ya badaye ushiriiki kwa warsha ya wiki moja. Tujulishe ikiwa utaweza kushiriki.**

**Hatari**

Kuna nafasi kidogo ya kufichuliwa kiajali hali ya virusi vya ukimwi. Walakini, tutaweka mikakati ya usalama ya kulinda hali yako ya virusi vya ukimwi.

**Faida**

Kuna uwezekano hutapokea faida ya kibinafsi kutokana na kushiriki kwa utafiti huu. Walakini, kushirkik kwako inaweza kuimarisha uhifadhi wa vijana kwa matibabu.

**Kurudishiwa pesa**

**Utarudishiwa pesa ya nauli ya shilingi ya Kenya 500/=.**

**Chaguo mbadala**

Uko huru kuamua kutoshirki kwa utafiti. Ikiwa umeamua kutoshiriki kwa utafiti huu, hakutakuwa na adhabu kwako. Hautapoteza faida zako zozote za kawaida, na pia badi unaweza kupata matibabu yako kutoka kwa kliniki jinsi umekuwa ukifanya.

**Usiri**

Tutafanya kadri ya uwezo wetu kuhakikisha habari za kibinfasi yalikusanywa kwa minajili ya utafiti imewekwa siri. Walakini, hatuwezi kuhakikisha usiri kamilifu. Habari zako za kibinfasi yanaweza kupeanwa ikihitajika kisheria. Ikiwa habari kutoka kwa utafiti huu umechapishwa au kusomwa kwenye mikutano ya sayansi, jina lako au habari zako zingine za kibinafsi hazitatumika.

Wafanyikazi wa serikali na vyuo vikuu wakati mwingine hungalia utafiti kama huu kuhakikisha yanafanywa kwa usalama na kulingana na sheria . Ikiwa utafiti huu umeangaliwa, rekodi zako zitachunguzwa. Wachunguzi hao watalinda ubinafsi wako. Rekodi za utafiti hazitatumika kukuweka kwa hatari ya kisheria. Mashirika yanaweza kuangalia na/au kunakili rekodi zako za utafiti kwa ajili ya utafiti, utenda kazi, na uchambuzi wa deta yanajumuisha:

- The Washington University, St. Louis
- The Kenya Medical Research Institute
- University of California San Francisco
- University of Colorado, Denver
- University of California, Berkeley

Kushiriki kwa utafiti unaweza kuwa na kupoteza ubinafsi , walakini tutaweka mikakati ya kulinda usiri wako. Habari yoyote ya ziada kuhusu utafiti utapewa ikujumuisha matokeo ya mwisho ya utafiti.

**Kushiriki**

Unaweza kuacha kushiriki kwa utafiti wakati wowote, ambia tu mfanyikazi wa utafiti na watakuondoa. Zaidi ya hayo, wafanyikazi wa utafiti wanaweza kukuondoa kwa utafiti, ikiwa wanahisi kwamba ni kwa maslahi yako. Hautaadhibiwa kwa njia yoyote ile kam ataamua kuacha kushiriki.

**Ufadhili**

Utafiti huu umefadhiliwa na National Institutes of Health. Hakuna yeyote kwa watafiti waliyo na mgongano wa maslahi ya kifedha. Katika hali yenye utafiti umekomeshwa kutokana na maamuzi ya mtafiti au mfadhili wa utafiti, utaelezwa kuhusu hatua ya kuchukiliwa baada ya hiyo.

**Mawasiliano**

Unaweza kuzungumza na m(wa)tafiti kuhusu maswali, wasiwasi, au malalamishi yoyote uliyonayo kuhusu utafiti huu. Wasiliana na mfanyikazi wetu wa utafiti katika 0733-819 835. Ikiwa ungependa kuuliza maswali kuhusu uatfiti au haki zako kama mshiriki wa utafiti kwa mtu mwingine kando ya watafiti au ukiwa ungependa kuzungumzia shida au wasiwasi ambazo uanweza kuwa nazo kuhusu utafiti, unaweza kuwasiliana na Katibu wa Ethical Review Committee, Kenya Medical Research Institute kwa seru@kemri.org au P.O. BOX 54840-00200 Tel. 020-2722541, 0717719477. Pa unaweza kuwasiliana na Washington University Human Research Protection Office kwa +1-314-747-6800.Haya kamati yanshughulika na kulinda haki za wanaojitolea kwa miradi za utafiti.

**IDHINI**

Umepewa nakala ya fomu hii ya idhini ili uweke.

**KUSHIRIKI KWA UTAFITI NI KWA HIARI.** Uko na haki ya kukataa kushiriki utafiti huu, au kujiondoa wakati wowote bila adhabu yoyote au kupoteza faida ambazo ungepata.

**Taarifa ya mshiriki**

Utafiti huu unaelezwa hapo juu nimeshaelezwa kuihusu. Najitolea kushiriki kwa utafiti huu. Nimekuwa na nafasi ya kuuliza maswali. Nikiwana maswali za siku zijazo kuhusu utafiti huu, ninaweza kuuliza watafiti waliondikwa hapo juu. Nikiwa na maswali kuhusu haki zangu kama mshiriki wa utafiti, ninaweza kuwasiliana na waliandikwa hapo juu.

Nimekubali habari zangu yatumike kwa minajili ya utafiti za siku zijazo na kupeanwa watafiti wengine bila ya kuongezea idhini na/bila vitambulishi.

Ikiwa ungependa kishiriki kwa utafiti huu, unafaa kuweka sahihi hapo chini.

Tarehe	Jina na Sahihi la Mshiriki kwa Idhini

Tarehe	Mtu Anayechukua Idhini (Jina na sahihi)

***Ikiwa hawezi kusoma na kuandika***

Nimeshuhudia kusomewa kwa njia sawa hii fomu ya idhini kwa mshiriki na mtu mwenyewe amekuwa na nafasi ya kuuliza maswali. Nathibitisha kuwa mtu mwenyewe amepeana idhini yaki akiwa huru**.**

Jina la shahidi _____________________ NA

Sahihi ya shahidi *__________________*AND

Jina la mshiriki __________________NA

**Alama ya kidole gumba ya mshiriki**

**Tarehe ________________________**

**Ruhusa ya kurekodi majadiliano**

Ninatoa ruhusa kwa majadiliano ya kikundi yenye nitashiriki irekodiwe**: Ndio ☐	La ☐**

**Ruhusa ya kuwasiliana na mimi kuhusu warsha ya wiki moja**

**Nimekubali kutafutwa kushiriki kwa warsha ya siku 3: Ndio ☐	La ☐**

Nakala kwa:	1. Watafiti

2. Mshiriki wa utafiti

**Study Title:** *Adaptive Strategies to Prevent and Treat Failures of Retention in Adolescent HIV Care (A4A Study)*

**KISWAHILI PARENTAL CONSENT FORM**

**KENYA MEDICAL RESEARCH INSTITUTE**

**WASHINGTON UNIVERSITY, ST. LOUIS**

**IDHINI YA MZAZI YA MAJADILIANO YA KIKUNDI (FGD)**

WATAFITI NA TAASISI SHIRIKISHI:

| Mtafiti | Taasisi | Mawasiliano |
| --- | --- | --- |
| Dkt. Elizabeth Bukusi | Kenya Medical Research Institute | +254 733-617 503 |
| Dkt. Elvin Geng | Washington University, Missouri | +1-415-430-5589 (USA) |
| Dkt. Lisa Abuogi | University of Colorado, Denver | +1-303-724-7430 (USA) |
| Dkt. Zachary Kwena | Kenya Medical Research Institute | +254 722-386 714 |
| Starley Shade | University of California, San Francisco | +1-415 794-4354 (USA) |
| Lina Montoya | University of Notrh Carolina at Chapel Hill | +1-4105857470 (USA) |
| Eliud Akama | Kenya Medical Research Institute | +254 728-590 376 |

**Eneo la utafiti:** Kisumu County

Unaombwa kuruhusu mtoto wako kushiriki kwa utafiti inayohusisha kushiriki kwa majadiano ya makundi . Sanduku iliyoko hapo chini inakueleza kuhusu mambo muhimu unayohitaji kufikira kabla ya kuamua kushiriki utafiti. Tutakupa habari zaidi chini ya sanduku hilo. Tafadhali uliza maswali kuhusu habari yoyote kabla ya kuamua ikiwa utashiriki au la. Pia ungependa kuzungumza na wengine (kwa mfano, familia yako, marafiki, au daktari wako) kuhusu utafiti, kabla ya kuamua kushiriki.

- **Idhini kwa hiari** . Unaombwa kuruhus mtoto wako ashiriki kwa majadiliano ya kikundi . Ni juu yako kuamua kuruhusu mtoto wako kushirki au la. Hakutakuwa na adhabu na hautapoteza chochote ikiwa umeamua kutoruhusu mtoto wako kushiriki
- **Lengo**. Tunafanya majadiliano ya vikundi tatu kukusanya fikira za vijana na vijana wenye wamebaleghe kuhusu mikakati ya kuingilia kati yanayopangwa kusaidia vijana na vijana wenye wamebaleghe kuendelea kuwakwa matibabu ya virusi vya ukimwi . Mikakati yaliyopangwa yatahusisha usaidizi wa kielektroniki na wanarika wa ana kwa ana (sawa na ushauri ) na kupeana pesa . Maoni ya mtoto wako na habari yatasaidia kujulisha mkutano spesheli kuhusu mikakati ya kuingilia kati inayoitwa warsha ya kuunda (design workshop). Mtoto wako anaweza kutafutwa kuhusu kushiriki kwa warsha ya kuunda kwa wakati wa baadaye .
- **Washiriki:** Takriban vijana na vijana waliyobaleghe 24, wa umri wa miaka 14 – 24 , na wnaishi na virusi vya ukimwi katika kaunti ya Kisumu watashiriki kwa majadialiano ya kikundi . Kutakuwa na vikundi 3 yenye watu 8 katika kila kikundi.

11. **Taratibu na shughuli.** Katika utafiti huu, ikiwa umekubali kuruhusu mtoto wako kushiriki kwa majadiliano ya vikundi , pia tutaomba ruhusa ya mtoto wako . Ukikubali , tutatenga saa kwa mtoto wako kujiunga na majadiliano ya kikundi. Majadiliano yataangazia maoja kati ya mikakati yaliyopangwa tatu kuweka vijana na vijana waliyobaleghe kuendelea na matibabu ya virusi vya ukimwi . Kila jadiliano itatekelezwa na mtu anayehitimu mahala pa binafsi kulinda usiri wa mtoto wako. Karibu na hayo, tunakuomba ruhusa kurekodi mahojiano , yenye tutaandika chini na kuangalia . Habari yoyote yeney inaweza kumtambua mtoto wako hayatajumuishwa kwa uangalizi huu. Tunatarajia haya mahojiano ya vikundi kukaa kwa muda wa saa na nusu.

**Hatari.** Kuan nafasi kidogo ya kufichulika kimakosa hali ya virusi vya ukimwi. Walakini, tutaweka mikakati ya kulinda habari kuhusu hali ya mtoto wako ya virusi vya ukimwi .

- **Faida**. Kuna uwezekano mtoto wako hatapokea faida yoyote ya kibinafsi kwa kushiriki kwa utafiti huu. Kwa kushiriki kwa utafiti huu anaweza kuchangia juhudi za kukuza afya ya vijana wanaoishi na virusi vya ukimwi .
- **Chaguo mbadala.** Uko huru kuamua kutoruhusu mtoto wako kushiriki kwa majadiliano. Ukiamua kutoruhusu mtoto wako kushiriki kwa utafiti huu, hakutakuwa na adhabu yoyote kwake . Hatapoteza faida zenu za kawaida , na pia kupata matibabu yake kwa kliniki jinsi amekuwa ukipata .

**Taratibu na shughuli**

Ukikubali kuruhusu mtoto wako kushiriki , na mtoto wako amekubali pia , yafuatayo yatafanyika:

1. Tutakuuliza uweke sahihi au uweke alama kwa fomu hii kama ishara kuwa umejitolea kushiriki kwa utafiti huu and tutatafuta ruhusa ya mtoto wako.
2. Tutapanga wakati yake ya kujiunga na kikundi cha vijana kijadili njia bora zaidi ya kutekeleza mikakati ya kuingilia kati yaliyopangwa .
3. Mtoto wako atakuwa sehemu ya majadiliano yanayoangazia moja kati ya mikakati ya kuingilia kati yaliyopangwa , kwa njia ya usaidizi wa kielektroniki (arafa fupi zinatumwa kwa njia ya kompyuta na usaidizi ya wanarika kupitia simu ya rununu), usaidizi wa ana kwa ana na wanarika, au kupeana pesa ukitimiza masharti (kupokea pesa).
4. Pia ataulizwa maswali kuhusu aliyokumbana nayo na changamoto na matibabu ya virusi vya ukimwi , mara ngapi na aina gani ya usaidizi , na maoni jinsi ya kutekeleza kwa njia bora zaidi mikakti yaliyopangwa.
5. **Maoni zake na habari yatasaidia kujulisha mkutano wa mikakati ya kuingilia kati inayoitwa warsha ya kuunda (design workshop.)**
6. Majadiliano yataongozwa na mtu anayehitimu kwa chumba cha kibinafsi kulinda usiri .
7. Tutamuuliza kuturuhusu kurekodi mahojiano na majibu zake ili tuweze kuiandika chini na kuangalia baadaye .
8. Habari yoyote yenye inaweza kutambulisha mtoto wako hayatajumuishwa kwa uangalizi.
9. Majadiliana yanakusudiwa kuchukua takriban muda wa saa moja na nusu .
10. **Mtoto wako anaweza kuulizwa kwa tarehe ya baadaye kushiriki kwa warsha ya wiki moja. Tujulishe ikiwa ungependa kuruhusu mtoto wako kushiriki.**

**Hatari**

Kuna uwezekano mdogo ya kufichuliwa kwa makosa hali ya virus ivy aulimwi . Walakini, tutaweka mikakati ya kulinda habari kuhusu hali ya mtoto wako ya virusi vya ukimwi.

**Faida**

Kuna uwezekano mtoto wako hatapokea faida ya kibinafsi kwa kishiriki kwa utafiti huu. Walakini, kushiriki kwa mtoto wako inaweza kusaidia kuboresha kuhifadhi vijana kwa matibabu .

**Kurudisha pesa**

**Mtoto wako atarudishiwa pea ya kusafiri ya shilingi ya Kenya 500/=.**

**Chaguo mbadala**

Uko huru kuamua kutoruhusu mtoto wako kushiriki kwa utafiti . Ikiwa umeamua kutoruhusu mtoto wako kushiriki kwa utafiti huu, hakutakuwa na adhabu kwake . Hatapoteza faida yoyote yake ya kawaida , na anaweza kuendela kupokea matibabu yake kutoka kwa kliniki vile nyinyi hufanya .

**Usiri**

Tutafanya kadri ya uwezo wetu kuhakikisha kuwa habri za kibinafsi yanayokusanywa kwa utafiti huu yamewekwa siri. Walakini, hatuwezi kuhakikisha usiri kamilifu. Habri a mtoto wako za kibinafsi yanaweza kupeanwa ikihitajika kisheria. Ikiwa habari kutoka kwa huu utafiti imechapishwa au kusomwa kwa mikutano ya kisayansi , jina lako au la mtoto wako na habari za kibinafsi hayatatumika.

Wafanyikazi wa serikali na vyuo vikuu wakati mwingine hungalia utafiti kama huu kuhakikisha yanafanywa kwa usalama na kulingana na sheria . Ikiwa utafiti huu umeangaliwa, rekodi zako zitachunguzwa. Wachunguzi hao watalinda ubinafsi wako. Rekodi za utafiti hazitatumika kukuweka kwa hatari ya kisheria. Mashirika yanaweza kuangalia na/au kunakili rekodi zako za utafiti kwa ajili ya utafiti, utenda kazi, na uchambuzi wa deta yanajumuisha::

- The Washington University, St. Louis
- The Kenya Medical Research Institute
- University of California San Francisco
- University of Colorado, Denver
- University of California, Berkeley

Kushiriki kwa utafiti inaweza kuhusisha kupoteza ubinafsi , walakini tutaweka mikakati ya kulinda usiri. Habari yoyote ya ziada kuhusu utafiti huu itapeanwa kwako ikijumuisha matokeo ya mwisho ya utafiti .

**Ushiriki**

Mtoto wako anaweza kuamua kukomesha mahojiano wakati wowote , ambia tu wafanyikazi wa utafiti na watamkomesha . Zaidi ya hayo, mfanyikazi wa uatfiti anaeza kumkomesha kishiriki , ikiwa wanahisi ni kwa maslahi bora yake. Hataadhibiwa kwa njia yoyote ikiwa ameamua kukomesha kushiriki .

**Ufadhili**

Utafiti huu umefadhiliwa na National Institutes of Health. Hakuna yeyote kwa watafiti waliyo na mgongano wa maslahi ya kifedha. Katika hali yenye utafiti umekomeshwa kutokana na maamuzi ya mtafiti au mfadhili wa utafiti, utaelezwa kuhusu hatua ya kuchukiliwa baada ya hiyo.

**Mawasiliano**

Unaweza kuzungumza na m(wa)tafiti kuhusu maswali, wasiwasi, au malalamishi yoyote uliyonayo kuhusu utafiti huu. Wasiliana na mfanyikazi wetu wa utafiti katika 0733-819 835. Ikiwa ungependa kuuliza maswali kuhusu uatfiti au haki zako kama mshiriki wa utafiti kwa mtu mwingine kando ya watafiti au ukiwa ungependa kuzungumzia shida au wasiwasi ambazo uanweza kuwa nazo kuhusu utafiti, unaweza kuwasiliana na Katibu wa Ethical Review Committee, Kenya Medical Research Institute kwa seru@kemri.org au P.O. BOX 54840-00200 Tel. 020-2722541, 0717719477. Pa unaweza kuwasiliana na Washington University Human Research Protection Office kwa +1-314-747-6800.Haya kamati yanshughulika na kulinda haki za wanaojitolea kwa miradi za utafiti.

**IDHINI**

Umepewa nakala ya fomu hii ya idhini ili uweke.

**KUSHIRIKI KWA UTAFITI NI KWA HIARI.** Uko na haki ya kukataa kushiriki utafiti huu, au kujiondoa wakati wowote bila adhabu yoyote au kupoteza faida ambazo ungepata.

**Taarifa ya mshiriki**

Utafiti huu unaelezwa hapo juu nimeshaelezwa kuihusu. Najitolea kushiriki kwa utafiti huu. Nimekuwa na nafasi ya kuuliza maswali. Nikiwana maswali za siku zijazo kuhusu utafiti huu, ninaweza kuuliza watafiti waliondikwa hapo juu. Nikiwa na maswali kuhusu haki zangu kama mshiriki wa utafiti, ninaweza kuwasiliana na waliandikwa hapo juu.

Nimekubali habari ya mtoto wangu yatumike kwa utafiti za siku zijazo na kupeanwakwa watafiti wengine bila idhini yangu ya kuongezea na vitambulishi au bila .

Ikiwa ungependa kuruhusu mtoto wako kushiriki kwa utafiti huu, unahitajika kuweka sahihi hapo chini.

Tarehe	Jina la Mshiriki

Tarehe	Jina na Sahihi ya Mtunzi/Mzazi

Tarehe	Mtu Anayechukua Idhini (Jina na sahihi)

***Ikiwa hawezi kusoma na kuandika***

Nimeshuhudia kusomewa kwa njia sawa hii fomu ya idhini kwa mshiriki na mtu mwenyewe amekuwa na nafasi ya kuuliza maswali. Nathibitisha kuwa mtu mwenyewe amepeana idhini yaki akiwa huru**.**

Jina la shahidi _______________________ NA

Sahihi ya shahidi ____________________NA

Jina la Mtunzi/Mzazi _______________NA

Alama ya kidole gumba ya Mtunzi/Mzazi

**Tarehe ________________________**

**Ruhusa ya kurekodi mahojiano**

Napeana ruhusa kwa majadialiano ya kikundi yenye mtoto wangu atashiriki kurekodiwa**: Ndio ☐	La ☐**

**Ruhusa ya mawasiliano ya warsha ya wiki moja**

**Ninarushusu mwasiliane na mtoto wangu kushiriki kwa warsha ya wiki moja:**

**Ndio ☐	La ☐**

Nakala kwa:	1. Watafiti

2. Mshiriki wa utafiti

***Adaptive Strategies to Prevent and Treat Failures of Retention in***

***Adolescent HIV Care (A4A Study)***

**DHOLUO A4A VERBAL ASSENT SCRIPT FOR DESIGN WORKSHOP*14 – 17 YEARS OLD***

JOTIM NORO KOD MIGEPE MA GITUDORE GO:

| Jatim Nonro | Migao | Yor Tudruok |
| --- | --- | --- |
| Laktar Elizabeth Bukusi | Kenya Medical Research Institute | +254 733-617 503 |
| Laktar Elvin Geng | Washington University, Missouri | +1-415-430-5589 (USA) |
| Laktar Lisa Abuogi | University of Colorado, Denver | +1-303-724-7430 (USA) |
| Laktar Zachary Kwena | Kenya Medical Research Institute | +254 722-386 714 |
| Starley Shade | University of California, San Francisco | +1-415-794-4354 (USA) |
| Lina Montoya | University of Notrh Carolina at Chapel Hill | +1-4105857470 (USA) |
| Eliud Akama | Kenya Medical Research Institute | +254 728-590 376 |

**Kama itimo e Nonro:** Kisumu County

**Wach motelo**

An [name of RA], awuok e Kenya Medical Research Institute (KEMRI). Watimo nonro ma iluongo ni **An Adaptive Strategy for Preventing and Treating Lapses of Retention in HIV Care for Adolescents (A4A).** Nonro ni itimo gi KEMRI kod Washington University man St. Louis, University of Colorado man Denver, gi University of California man Berkeley kod San Francisco e piny Amerka. Neno ni rowere kod jomadongo matindo man gi kute mag ayaki omedo bedo e rit mar kute mag ayaki kendo keto kwan mar gi mag kute mag ayaki e remo bedo ma ok nyal ne en gimaduong’e keto gi ka gingima. E nonro ni, watemo ng’eyo kaka wanyalo miyo rowere kod jomadongo matindo chenro ma biro konyo gi bedo e rit kendo bedo gi kwan kute mak nyal ne.

Wakwayi mondo ibed e twak mar juma achiel mar konyo loso chenro mag nonro ni . Twak gi biro bedo gi okenge ma himo paro kuom kendo keto maber chenro ma ochan ne nonro ni.

E kinde twak ni, wabiro nyisi weche kuom nonro ni bang’e to wabedo gi okenge mag twak kendo los chenro mag gigo ma inyalo tim a nyalo konyo. Kaka rawera kata ng’amaduong’ matin e oganda ni, dwaher winjo pachi kod kaka ineno weche mondo okonywa loso yore mabeyo mogik ne rowere kod jomadongo matindo. Kuom ranyisi, yiero weche ma iketo e ote machwok mar sime( SMS) kodo kaka mbese matiego ji nyalo siro rowere kod jomadongo matindo e yorsim to kod kagibiro gin giwegi.

Bedo mari e nonro en kuom chiwruok. Ka ok diher mar bedo, inyalo wuok e saa asaya kendo onge kum ma biro bedo ne in. Ok ibi wito ber ma iyudo ga ma pile , kendo podi inyalo yudo rit kawuok e klinik mari kaka iyude ga. Dwoko duto ok bi bedo gi nying; nyingi ok bi neno kamoro amora e ndiko ma otim mogik.

Nitie rach matin ma otudore kod bedo e twak mar jumaa achiel. E kinde twak mar jumaa achiel, ok wabi nyalo singo maling’ling’ nikech wabiro bedo ka watwak kuom weche kaka kanyaklamar ji. Nikech mano, ka inyalo winjo ka ok in thuolo gi wach moro amora ma iwacho mondo onyis jomoko e kanyakla kata oko mar kanyakla, kik iwach gi e kinde ma twak dhi nyime.

Bende in gi penjo moro aora kuom nonro ni?

Ka diher yudo kopi mar barua ni mondo ikan, yie inyisa kendo abiro [miyi kopi no sani; oro ni go e barup mbuyi, barua, kata ore e yor faks, e kind mamoko.].

Ka in gi penjo mamoko momedore kotudore gi nonro , tudri kod Laktar Elizabeth Ann Bukusi, Kenya Medical Research Institute, 0733617503 kata Laktar Elvin Geng, Washington University, + 14154305589. Ka in gi penjo moro amora kotudore gi ratiro magi kaka jachiwre mar nonro , yie itudri kod Scientific and Ethics Review Committee mar Kenya Medical Research Institute e 0717719477 kata [seru@kemri.org](mailto:seru@kemri.org) KATA Washington University Human Subjects Protection Program e +13147476800.

Nying Jatij Nonro ma Tayo kawo Yie (printed)	Tije/Nying Tije

Seyi Jatij Nonro ma Tayo kawo Yie	Tarik

***Adaptive Strategies to Prevent and Treat Failures of Retention in***

***Adolescent HIV Care (A4A Study)***

**DHOLUO A4A VERBAL CONSENT SCRIPT FOR DESIGN WORKSHOP*JO HIGNI 18 – 24***

JOTIM NONRO GI MIGEPE MA GITUDORE GODO:

| Jatim Nonro | Migao | Yor Tudruok |
| --- | --- | --- |
| Laktar Elizabeth Bukusi | Kenya Medical Research Institute | +254 733-617 503 |
| Laktar Elvin Geng | Washington University, Missouri | +1-415-430-5589 (USA) |
| Laktar Lisa Abuogi | University of Colorado, Denver | +1-303-724-7430 (USA) |
| Laktar Zachary Kwena | Kenya Medical Research Institute | +254 722-386 714 |
| Starley Shade | University of California, San Francisco | +1-415-794-4354 (USA) |
| Lina Montoya | University of Notrh Carolina at Chapel Hill | +1-4105857470 (USA) |
| Eliud Akama | Kenya Medical Research Institute | +254 728-590 376 |

**Kama itimo e Nonro:** Kisumu County

**Wach motelo**

An [name of RA], awuok e Kenya Medical Research Institute (KEMRI). Watimo nonro ma iluongo ni **An Adaptive Strategy for Preventing and Treating Lapses of Retention in HIV Care for Adolescents (A4A).** Nonro ni itimo kod KEMRI kod Washington University man St. Louis, University of Colorado man Denver, gi University of California man Berkeley kod San Francisco e piny Amerka. Neno ni rowere kod jomadongo matindo man gi kute mag ayaki bedo e thieth mar kute mag ayaki kendo keto kwan kute man e rembgi ma ok nyal fweny en gimaduong’ e keto gi ka gibedo mangima. E nonro ni, watemo ng’eyo kaka inyalo mi rowere kod jomadongo matindo chenro ma biro konyo gi bedo e thieth kendo keto kwan mar kute bedo ma ok nyal ne e remo.

Wakwayi mondo ibed e twak mar juma achiel mar konyo loso chenro mar nonro ni . Twak ni biro bedo gi chenro moko mag paro kuom kendo neno kaka iketo maber chenro ma ochan ne nonro ni .

E kinde nonro ni, wabiro kwongo chiwo ne in weche kuom nonro kendo bang’ mano chenr mag twak kendo chano chenro mag konyo. Kaka rawera kata ng’amaduong’ matin e oganda ni , dwaher pachi mondo okonywa e loso yo maber mogik mar konyo rowere kod jomadongo matindo. Kuom ranyisi, yiero weche ma itiyo godo e ote machwok mar sime (SMS) kod kaka mbese ma konyo nyalo siro rowere kod jomadongo matindo e yor sime kata ka gibiro gin giwegi.

Bedo ni e nonro en kuom chiwruok. Ka ok diher mar bedo, inyalo weyo e saa asaya kendo onge kum ma ibiro miyi. Ok ibi wito ber ma iyudo ma ma pile, kendo inyalo yudo thieth mari kawuok e klinik mana kaka ijayudo ga. Dwoko gi biro bedo maonge nying chutho; nyingi ok bi bedo kamoro amora e ndiko mogik.

Nitie rach moko matin motudore gi bedo e twak mar jumaa achiel . E kinde twak mar jumaa achiel, ok wabi singo maling’ling’ nikech wabiro twak kuom weche kaka kanyakla. Nikech mano, ka inyalo winjo ka ok in thuolo gi moro amora kuom gik ma iwacho mondo onyis jomoko e kanyakla kata oko markanyakla, yie kik iwach gi e kinde mo.

Bende in gi penjo moro amora kuom nonro ni?

Ka diher yudo kopi mar barua ni mondo ikan , yie inyisa kendo abiro [miyi kopi sani; oro ni e barup mbuyi, barua, kata ore e yor faks ne in, etc.].

Ka in gi penjo mamoko momedore kotudore gi nonro ni , tudri kod Laktar Elizabeth Ann Bukusi, Kenya Medical Research Institute, 0733617503 kata Laktar Elvin Geng, Washington University, + 14154305589. Ka in gi penjo moro amora motudore gi ratiro magi kaka jachiwre mar nonro , yie itudri kod Scientific and Ethics Review Committee of Kenya Medical Research Institute e 0717719477 kata [seru@kemri.org](mailto:seru@kemri.org) KATA Washington University Human Subjects Protection Program e +13147476800.

Nying Jatij Nonro ma Tayo kawo Yie (printed)	Tije/Nying Tije

Seyi Jatij Nonro ma Tayo kawo Yie	Tarik

***Adaptive Strategies to Prevent and Treat Failures of Retention in***

***Adolescent HIV Care (A4A Study)***

**DHOLUO A4A VERBAL CONSENT SCRIPT FOR DESIGN WORKSHOP*FOR PARENTS OF 14 – 17 YEAR OLDS***

JOTIM NONRO KOD MIGEPE MA GIN GO GI TUDRUOK:

| Jatim Nonro | Migao | Yor Tudruok |
| --- | --- | --- |
| Laktar Elizabeth Bukusi | Kenya Medical Research Institute | +254 733-617 503 |
| Laktar Elvin Geng | Washington University, Missouri | +1-415-430-5589 (USA) |
| Laktar Lisa Abuogi | University of Colorado, Denver | +1-303-724-7430 (USA) |
| Laktar Zachary Kwena | Kenya Medical Research Institute | +254 722-386 714 |
| Starley Shade | University of California, San Francisco | +1-415 794-4354 (USA) |
| Lina Montoya | University of Notrh Carolina at Chapel Hill | +1-4105857470 (USA) |
| Eliud Akama | Kenya Medical Research Institute | +254 728-590 376 |

**Kama itimo e Nonro:** Kisumu County

**Wach motelo**

An [name of RA], ma owuok Kenya Medical Research Institute (KEMRI). Watimo nonro ma iluongo ni **An Adaptive Strategy for Preventing and Treating Lapses of Retention in HIV Care for Adolescents (A4A).** Nonro ni itimo kod KEMRI gi Washington University man St. Louis, University of Colorado man Denver, kod University of California man Berkeley kod San Francisco man Amerka. Neno ni rowere kod jamadongo matindo man gi kute mag ayaki obedo e thieth mar kute mag ayaki kendo keto kwan mar kute bedo ma ok nyal ne gin gimaduong’ e keto gi bedo ka gingima. E nonro ni , watemo ng’eyo kaka inyalo mi rowere kod jomadongo matindo chenro ma nyalo konyogi bedo e thieth kendo bedo gi kwan kute ma ok nyal fwenyo.

Wakwayi mondo iyie ne nyathini mondo obed e twak mar juma achiel. Twak ni biro bedo gi chenro moko mag paro kuom kendo neno kaka iketo maber chenro ma ochan ne nonro ni .

E kinde nonro ni, wabiro kwongo chiwo ne in weche kuom nonro kendo bang’ mano chenr mag twak kendo chano chenro mag konyo. Kaka rawera kata ng’amaduong’ matin e oganda ni , dwaher pachi mondo okonywa e loso yo maber mogik mar konyo rowere kod jomadongo matindo. Kuom ranyisi, yiero weche ma itiyo godo e ote machwok mar sime (SMS) kod kaka mbese ma konyo nyalo siro rowere kod jomadongo matindo e yor sime kata ka gibiro gin giwegi.

Bedo ni e nonro en kuom chiwruok. Ka ok diher mar bedo, inyalo weyo e saa asaya kendo onge kum ma ibiro miyi. Ok ibi wito ber ma iyudo ma ma pile, kendo inyalo yudo thieth mari kawuok e klinik mana kaka ijayudo ga. Dwoko gi biro bedo maonge nying chutho; nyingi ok bi bedo kamoro amora e ndiko mogik.

Nitie rach moko matin motudore gi bedo e twak mar jumaa achiel ni. E kinde twak mar jumaa achiel, ok wabi singo maling’ling’ nikech wabiro twak kuom weche kaka kanyakla. Nikech mano, ka inyalo winjo ka ok in thuolo gi moro amora kuom gik ma iwacho mondo onyis jomoko e kanyakla kata oko markanyakla, yie kik iwach gi e kinde mo.

Bende in gi penjo moro amora kuom nonro ni?

Ka diher yudo kopi mar barua ni mondo ikan , yie inyisa kendo abiro [miyi kopi sani; oro ni e barup mbuyi, barua, kata ore e yor faks ne in, etc.].

Ka in gi penjo mamoko momedore kotudore gi nonro ni , tudri kod Laktar Elizabeth Ann Bukusi, Kenya Medical Research Institute, 0733617503 kata Laktar Elvin Geng, Washington University, + 14154305589. Ka in gi penjo moro amora motudore gi ratiro magi kaka jachiwre mar nonro , yie itudri kod Scientific and Ethics Review Committee of Kenya Medical Research Institute e 0717719477 kata [seru@kemri.org](mailto:seru@kemri.org) KATA Washington University Human Subjects Protection Program e +13147476800.

Nying Jatij Nonro ma Tayo kawo Yie (printed)	Tije/Nying Tije

Seyi Jatij Nonro ma Tayo kawo Yie	Tarik

***Adaptive Strategies to Prevent and Treat Failures of Retention in***

***Adolescent HIV Care (A4A Study)***

**KISWAHILI A4A VERBAL ASSENT SCRIPT FOR DESIGN WORKSHOP*14 – 17 YEARS OLD***

WATAFITI NA TAASISI SHIRIKISHI:

| Mtafiti | Taasisi | Mawasiliano |
| --- | --- | --- |
| Dkt. Elizabeth Bukusi | Kenya Medical Research Institute | +254 733-617 503 |
| Dkt. Elvin Geng | Washington University, Missouri | +1-415-430-5589 (USA) |
| Dkt. Lisa Abuogi | University of Colorado, Denver | +1-303-724-7430 (USA) |
| Dkt. Zachary Kwena | Kenya Medical Research Institute | +254 722-386 714 |
| Starley Shade | University of California, San Francisco | +1-415 794-4354 (USA) |
| Lina Montoya | University of Notrh Carolina at Chapel Hill | +1-4105857470 (USA) |
| Eliud Akama | Kenya Medical Research Institute | +254 728-590 376 |

**Eneo la utafiti :** Kisumu County

**Utangulizi**

Mimi ni [name of RA], kutoka Kenya Medical Research Institute (KEMRI). Tunafanya utafiti inayojulikana kama **An Adaptive Strategy for Preventing and Treating Lapses of Retention in HIV Care for Adolescents (A4A).** Utafiti huu unafanywa na KEMRI na Washington University iliyoko St. Louis, University of Colorado iliyoko Denver, na University of California iliyiko Berkeley na San Francisco nchini Marekani. Kuhakisha kuwa vijana na vijana wabelehe wanaoishi na virusi vya ukimwi kubaki kwa matibabu ya virusi vya ukimwi na kufanya kiwango chao cha virusi kirudi hadi sufuri ni muhimu zaidi kwa kuwa wenye afya. Katika utafiti huu, tunajaribu kujua jinsi ya kupeana vijana na vijana waliyobalehe mikakati ya kuingilia kati yatakayoweza kuwasiadia kuendelea kupata matibabu na kubakisha kiwango cha virusi kuwa sufuri.

Tunakuomba mshiriki kwa warsha ya wiki moja kusaidia kuunda mikakati ya kuingilia kati kwa minajili ya utafiti huu . Warsha itakuwa na vikao zitakazoangazia kufikiria kuhusu na kuboresha mikakati ya kuingilia kati yanayaopangwa kwa utafiti huu.

Wakati wa warsha huu, tutakupa kwanza mukhtasari juu ya utafiti na baadaye kuwa na vipindi vya kujadili na kuunda mipango ya mikakati ya kuingilia kati. Kama kijana au mtu mzima mdogo katika hii jamii, tungependa mtazamo wako na maoni yako kutusaidia kuunda mikakati ya kuingilia kati bora zaidi kwa vijana na vijana waliyobaleghe. Kwa mfano , kuamua maneno ya arafa fupi na jinsi wasiadizi wanarika wanweza kusaidia vijana na vijana waliyobaleghe kwa njia ya simu kwa kibinafsi.

Kushiriki ni kwa hiari . Ikiwa hungependa kushiriki , unaweza kujiondoa wakati wowote na hakutakuwa na adhabu yoyote kwako . Hautapoteza faida zako na kawaida , na unaweza kuendelea kupokea matibabu yako kwa kliniki yako jinsi umekuwa ukipokea. Majibu yatakuwa bila vitambulishi; jina lako halitaonekana popote kwenye maandiko ya mwisho.

Kuna hatari za viwango vya chini yanayohusiana na kushiriki kwa warsha ya wiki moja. Wakatai wa warsha, hatutaweza kuhakikisha usiri kamilifu kwa sababu tutakuwa tunajadili mambo kama kikundi . Kwa hivo, ikiwa utahisi ukiwa hauko huru na matamshi yako yoyote kupeanwa kwa wengine ndani au nje ya kikundi , tafadhali usiyaseme wakati wa hii shughuli.

Je uko na swali lolote kuhusu utafiti?

Ikiwa ungependa kupata nakala ya barua hii kwa rekodi zako , tafadhali nijulishe na nita [kupa nakala sasa ; kukutumia kwa barua pepe, barua, au kipepesi , nk.].

Ukiwa na maswali zaidi kuhusu utafiti , wasiliana na Dkt. Elizabeth Ann Bukusi, Kenya Medical Research Institute, 0733617503 au Dkt. Elvin Geng, Washington University, + 14154305589. Ukiwa na swali lolote kuhusu haki zako kama mshiriki wa utafiti , tafadhali wasiliana na Scientific and Ethics Review Committee ya Kenya Medical Research Institute kwa 0717719477 au [seru@kemri.org](mailto:seru@kemri.org) AU Washington University Human Subjects Protection Program kwa +13147476800.

Jina la Mfanyikazi Anayechukua Idhini (printed)	Kazi/Jina

Sahihi ya Mfanyikazi wa Utafiti anayechukua Idhini	Tarehe

***Adaptive Strategies to Prevent and Treat Failures of Retention in***

***Adolescent HIV Care (A4A Study)***

**KISWAHILI A4A VERBAL CONSENT SCRIPT FOR DESIGN WORKSHOP*18 – 24 YEARS OLD***

WATAFITI NA TAASISI SHIRIKISHI:

| Mtafiti | Taasisi | Mawasiliano |
| --- | --- | --- |
| Dkt. Elizabeth Bukusi | Kenya Medical Research Institute | +254 733-617 503 |
| Dkt. Elvin Geng | Washington University, Missouri | +1-415-430-5589 (USA) |
| Dkt. Lisa Abuogi | University of Colorado, Denver | +1-303-724-7430 (USA) |
| Dkt. Zachary Kwena | Kenya Medical Research Institute | +254 722-386 714 |
| Starley Shade | University of California, San Francisco | +1-415 794-4354 (USA) |
| Lina Montoya | University of Notrh Carolina at Chapel Hill | +1-4105857470 (USA) |
| Eliud Akama | Kenya Medical Research Institute | +254 728-590 376 |

**Eneo la utafiti :** Kisumu County

**Utangulizi**

Mimi ni [name of RA], kutoka Kenya Medical Research Institute (KEMRI). Tunafanya utafiti inayojulikana kama **An Adaptive Strategy for Preventing and Treating Lapses of Retention in HIV Care for Adolescents (A4A).** Utafiti huu unafanywa na KEMRI na Washington University iliyoko St. Louis, University of Colorado iliyoko Denver, na University of California iliyiko Berkeley na San Francisco nchini Marekani . Kuhakisha kuwa vijana na vijana wabelehe wanaoishi na virusi vya ukimwi kubaki kwa matibabu ya virusi vya ukimwi na kufanya kiwango chao cha virusi kirudi hadi sufuri ni muhimu zaidi kwa kuwa wenye afya. Katika utafiti huu, tunajaribu kujua jinsi ya kupeana vijana na vijana waliyobalehe mikakati ya kuingilia kati yatakayoweza kuwasiadia kuendelea kupata matibabu na kubakisha kiwango cha virusi kuwa sufuri.

Tunakuomba mshiriki kwa warsha ya wiki moja kusaidia kuunda mikakati ya kuingilia kati kwa minajili ya utafiti huu . Warsha itakuwa na vikao zitakazoangazia kufikiria kuhusu na kuboresha mikakati ya kuingilia kati yanayaopangwa kwa utafiti huu.

Wakati wa warsha huu, tutakupa kwanza mukhtasari juu ya utafiti na baadaye kuwa na vipindi vya kujadili na kuunda mipango ya mikakati ya kuingilia kati. Kama kijana au mtu mzima mdogo katika hii jamii, tungependa mtazamo wako na maoni yako kutusaidia kuunda mikakati ya kuingilia kati bora zaidi kwa vijana na vijana waliyobaleghe. Kwa mfano, kuamua maneno ya arafa fupi na jinsi wasiadizi wanarika wanweza kusaidia vijana na vijana waliyobaleghe kwa njia ya simu kwa kibinafsi.

Kushiriki ni kwa hiari. Ikiwa hungependa kushiriki, unaweza kujiondoa wakati wowote na hakutakuwa na adhabu yoyote kwako . Hautapoteza faida zako na kawaida , na unaweza kuendelea kupokea matibabu yako kwa kliniki yako jinsi umekuwa ukipokea. Majibu yatakuwa bila vitambulishi; jina lako halitaonekana popote kwenye maandiko ya mwisho.

Kuna hatari za viwango vya chini yanayohusiana na kushiriki kwa warsha ya wiki moja. Wakati wa warsha , hatutaweza kuhakikisha usiri kamilifu kwa sababu tutakuwa tunajadili mambo kama kikundi . Kwa hivo, ikiwa utahisi ukiwa hauko huru na matamshi yako yoyote kupeanwa kwa wengine ndani au nje ya kikundi , tafadhali usiyaseme wakati wa hii shughuli.

Je uko na swali lolote kuhusu utafiti?

Ikiwa ungependa kupata nakala ya barua hii kwa rekodi zako , tafadhali nijulishe na nita [kupa nakala sasa ; kukutumia kwa barua pepe, barua, au kipepesi , nk.].

Ukiwa na maswali zaidi kuhusu utafiti , wasiliana na Dkt. Elizabeth Ann Bukusi, Kenya Medical Research Institute, 0733617503 au Dkt. Elvin Geng, Washington University, + 14154305589. Ukiwa na swali lolote kuhusu haki zako kama mshiriki wa utafiti , tafadhali wasiliana na Scientific and Ethics Review Committee ya Kenya Medical Research Institute kwa 0717719477 au [seru@kemri.org](mailto:seru@kemri.org) AU Washington University Human Subjects Protection Program kwa +13147476800.

Jina la Mfanyikazi Anayechukua Idhini (printed)	Kazi/Jina

Sahihi ya Mfanyikazi wa Utafiti anayechukua Idhini	Tarehe

***Adaptive Strategies to Prevent and Treat Failures of Retention in***

***Adolescent HIV Care (A4A Study)***

**KISWAHILI A4A VERBAL CONSENT SCRIPT FOR DESIGN WORKSHOP*FOR PARENTS OF 14 – 17 YEAR OLDS***

WATAFITI NA TAASISI SHIRIKISHI:

| Mtafiti | Taasisi | Mawasiliano |
| --- | --- | --- |
| Dkt. Elizabeth Bukusi | Kenya Medical Research Institute | +254 733-617 503 |
| Dkt. Elvin Geng | Washington University, Missouri | +1-415-430-5589 (USA) |
| Dkt. Lisa Abuogi | University of Colorado, Denver | +1-303-724-7430 (USA) |
| Dkt. Zachary Kwena | Kenya Medical Research Institute | +254 722-386 714 |
| Starley Shade | University of California, San Francisco | +1-415 794-4354 (USA) |
| Lina Montoya | University of Notrh Carolina at Chapel Hill | +1-4105857470 (USA) |
| Eliud Akama | Kenya Medical Research Institute | +254 728-590 376 |

**Eneo la utafiti:** Kisumu County

**Utangulizi**

Mimi ni [name of RA], kutoka Kenya Medical Research Institute (KEMRI). Tunafanya utafiti inayojulikana kama **An Adaptive Strategy for Preventing and Treating Lapses of Retention in HIV Care for Adolescents (A4A).** Utafiti huu unafanywa na KEMRI na Washington University iliyoko St. Louis, University of Colorado iliyoko Denver, na University of California iliyiko Berkeley na San Francisco nchini Marekani . Kuhakisha kuwa vijana na vijana wabelehe wanaoishi na virusi vya ukimwi kubaki kwa matibabu ya virusi vya ukimwi na kufanya kiwango chao cha virusi kirudi hadi sufuri ni muhimu zaidi kwa kuwa wenye afya. Katika utafiti huu, tunajaribu kujua jinsi ya kupeana vijana na vijana waliyobalehe mikakati ya kuingilia kati yatakayoweza kuwasiadia kuendelea kupata matibabu na kubakisha kiwango cha virusi kuwa sufuri.

Tunakuomba uruhusu mtoto wako kushiriki kwa warsha ya wiki moja ya kusaidia kuunda mikakati ya kuingilia kati kwa utafiti huu . Warsha itakuwa na vipindi vyenye vinaangazia kufikiria kuhusu na kuimarisha mikakati ya kuingilia kati yanayopangwa kwa uatfiti huu.

Wakati wa warsha hii, tutampa kwanza mukhtasari kuhusu utafiti huu na baadaye kuwa na mahojiano na kuunda mipango ya mikakati ya kuingilia kati . Kama kijana au kijana aliyebaleghe kwa hii jamii, tungependa kupata mtazamo wake na maoni yake ili zitusaidie kuunda mikakati ya kuingilia kati bora zaidi kwa vijana na vijana waliyobaleghe . Kwa mfano, kuamua maneno ya arafa fupi na jinsi wanarika wasaidizi wanaweza kusaidia vijana na vijana waliyobaleghe kwa njia ya simu na kibinafsi .

Kushiriki kwa mtoto wako ni kwa hiari. Ikiwa hangependa kushiriki , anaweza kujiondoa wakati wowote na hakutakuwa na adhabu . Mtoto hatapoteza faida zozote zenye amekuwa akipata kwa kawaida , na anaweza bado kupokea matibabu kutoka kwa kliniki jinsi yeye hufanya . Majibu yatakuwa kamwe bila vitambulishi ; jina lako halitaonekana popote kwenye maandiko ya mwisho.

Kuna hatari kidogo zaidi zinazohusishwa na kushiriki kwa warsha ya wiki moja. Wakati wa warsha ya wiki moja, hatutaweza kukuhakikishia usiri kamilifu kwa sababu tutakuwa tanajadili mambo kama kikundi. Kwa hivyo , ikiwa mtoto wako hatajihisi akiwa hayuko huru na matamshi yako yoyote kupeanwa kwa wengine ndani au nje ya kikundi , tafadhali usimruhusu kuyazungumzia wakati wa hii shughuli .

Je una swali lolote kuhusu utafiti?

Ikiwa ungependa kupata nakala ya barua hii kwa rekodi zako , tafadhali nijulishe na nita [kupa nakala sasa ; kukutumia kwa barua pepe, barua, au kipepesi , nk.].

Ukiwa na maswali zaidi kuhusu utafiti , wasiliana na Dkt. Elizabeth Ann Bukusi, Kenya Medical Research Institute, 0733617503 au Dkt. Elvin Geng, Washington University, + 14154305589. Ukiwa na swali lolote kuhusu haki zako kama mshiriki wa utafiti , tafadhali wasiliana na Scientific and Ethics Review Committee ya Kenya Medical Research Institute kwa 0717719477 au [seru@kemri.org](mailto:seru@kemri.org) AU Washington University Human Subjects Protection Program kwa +13147476800.

Jina la Mfanyikazi Anayechukua Idhini (printed)	Kazi/Jina

Sahihi ya Mfanyikazi wa Utafiti anayechukua Idhini	Tarehe

**KENYA MEDICAL RESEARCH INSTITUTE**

**WASHINGTON UNIVERSITY, ST. LOUIS**

**OVERALL DHOLUO ASSENT FORM**

**ASSENT TO PARTICIPATE IN A RESEARCH STUDY**

***FOR PARTICIPANTS 14 - 17 YEARS OLD***

**Study Title:** *Adaptive Strategies to Prevent and Treat Failures of Retention in Adolescent HIV Care (A4A Study)*

JOTIM NONRO KOD MIGEPE MA GITUDORE GODO:

| Jatim Nonro | Migao | Yor Tudruok |
| --- | --- | --- |
| Laktar Elizabeth Bukusi | Kenya Medical Research Institute | +254 733-617 503 |
| Laktar Elvin Geng | Washington University, St. Louis | +1-415-430-5589 (USA) |
| Laktar Lisa Abuogi | University of Colorado, Denver | +1-303-724-7430 (USA) |
| Laktar Zachary Kwena | Kenya Medical Research Institute | +254 722-386 714 |
| Starley Shade | University of California, San Francisco | +1-415-794-4354 (USA) |
| Lina Montoya | University of Notrh Carolina at Chapel Hill | +1-4105857470 (USA) |
| Eliud Akama | Kenya Medical Research Institute | +254 728-590 376 |

**Kama itimo e Nonro:** Kisumu County

Ikwayi mondo ibed e nonro . Boksi man piny kanyio nyisi gik mabeyo ma onego ing’e kapok iyiero mar donjo e nonro ni. Wabiro miyi weche matut e bwo boksi no. Yie ipenj penjo moro amora kuom weche go kapok iyiero mar donjo . Bende inyalo dwaro mar wuoyo gi jomoko (kuom ranyisi , jo anyuola u, osiepe, kata laktar mari ) kuom nonro ni, kapok iyie mar donjo.

- **Siro yie kuom chiwruok**. Ikwayi mondo ichiwri ne nonro. In ema iyiero ka idwa donjo kata ok idwar. Onge kum kendo onge gimoro amora ma ibiro wito ka iyiero ni ok idonj kata ka isedonjo, iyiero mar wuok.
- **Gima omiyo itime**. Watimo nonro ni mondo wang’e ni riwruok mag chenro mage ma kelo e tudruok maber mogik e thieth mar kute mag ayaki kuom rowere kod jomadongo matindo. Bedo e thieth kod dwoko chien kwan kute ma ok nyal kwanre en gimaduong’ahinya e neno ni jomatindo ma odak gi kute mag ayaki omedo bedo ka gingima. To, rowere kod jomadongo matindo bedo kod gigo ma mono gi kawo thieth kaka dwarore kendo medo bedo e thieth. E nonro watemo ngéyo kaka inyalo mi rowere kod jomadongo matindo yo ma nyalo konyo gi bedo e thieth kendo keto kwan kute obed maonge.
- **Jochiwre.** Madirom rowere kod jomadongo matindo 880 man jo higni kind 14-24 ma odak gi kute mag ayaki e Kisumu County biro bedo e nonro ni.
- **Thuolo.** Bedo ni e nonro ni biro kawo madirom higni 2.
- **Chenro kod gik ma itimo.**

E nonro ni, ibiro ng’iyi ka inyalo donjo e nonro kendo e yor radharadha keti e achiel kuom yore ariyo mokwongo mag konyo, ma en thieth mapile kata tudruok gi mbese e yor kompyuta(electronic peer navigation) (ote machwok me ioro gi kompyuta kod siro ma ichiwo kod jogo ma uchal godo e yor sime). Ka ilewo biro e limbe ni mar klinik kuom ndalo 14 KATA in kod kwan mar kute man malo, ibiro keti e yor radharadha e jogo mayudo thieth mapile maonge gima omedore , oro pesa ka itimo gima idwaro (yudo pesa), kata mbese gi ma wuoyo kodi en owuon.

Wabiro penji penjo kuom kaka idak, ngimani kod kaka idak gi ji, kod ngéyo ma in godo kuom kute mag ayaki gi kawo thieth kaka dwarore. Penjo gi biro bedo e thuolo ma irwaki e nonro, ka oketi e kidieny machielo (ka ilewo biro e klinik kuom ndalo 14 KATA in kod kwan kute man malo), kendo ka isebedo e nonro kuom higa 1 kendo bang’ higni 2. Bende wabiro penji penjo kuom kaka ineno kod gigo ma isekalo e kuom yore mag konyo ma iseyudo ka oketi e kidieny machielo, bang’ka isebedo e nonro kuom higa 1 kendo bang’higni 2. Penjo gi ibiro tim a kar thieth to inyalo tim gi e gweng’ka dwarore. Penjo gi biro kawo madirom dakika 45-60.

Bende wabiro kawo weche ma ochok ma pile e klinik kowuok e andike gi mag thieth. Ka kwan mari mar kute ok okaw e klinik ka oengo okawe , nonro nyalo tudre kodi kendo kawo remo ne pim mar kwan kute madirom dweche 6 ka dweche 6.

- **Rach.**
- Rem kata lit matin kowuok e golo remo.
- Fwenyruok ma ok ochan mar chal mar kute mag ayaki. Kata kamano, wabiro keto yore mag geng’o mondo wane ni chal mari mar kute mag ayaki orit.
- **Ber**. Ok inyal yudo ber mochimore kodi tir kuom bedo e nonro ni. Inyalo yudo ote machwok mar simo (SMS) kod tudruok gi mbese e yor sime, yudo pesa, kata bedo kod mbas ma jiwo ji ma ubet godo wang’gi wang’ . Chenro mag konyo gi nyalo konyi bedo e thieth mar kute mag ayaki knedo kawo yedhe gi maber kendo kaluwore gi mano bende nyalo konyo ngimani. Komedore, gik ma wafwenyo kuom nonro ni biro konyo keto maber thieth mar kute mag ayaki kuom rowere obed maber.
- **Yiero mamoko.** In thuolo mar yiero mondo kik ibed e nonro. Ka iyiero mar ni kik ibed e nonro ni, onge kum ma ibiro miyi. Ok ibi wito ber moro amora ma magi mapile , kendo podi inyalo yudo thieth e kilini mari kaka iyudo ga.

**Chenro kod gik ma itimo**

Mokwongo, janyuol kata jarit ni ibiro kwa thuolo mondo ibed e nonro ni. Ka dipo ni janyuol okobiro kodi e klinik, wabiro tudre kode e yor sime mondo wakwa thuolo mondo ibed e nonro. Ka janyuol kata jarit mari ok oyie , ok inya bedo e nonro . Ka janyuol kata jarit mari oyie, kendo in bedne iyie, ma e gima biro timore tok mano:

**E rwako ji e nonro**

Wabiro penji penjo kuom kaka idak , ngimani to gi kaka idak gi ji , kod ng’eyo kuom kute mag ayaki kod mwonyo yath kaka dwarore. Nonro mar penjo ni biro kawo madirom dakika 45-60**.**

Ibiro keti e achiel kuom yore ariyo mag jiwo jotuo. Yiero yo ma ibiro yudo itimo e yor radharadha, ya machal gi wito otonglo malo ka ing’iyo kama olwar godo. Yore mag konyo go gin:

**“Thieth mapile”** ma en ni ka ilewo ma ok ibiro kaka ochan, yo mapile mar klinik mar paroni mondo ibi e limbe ema biro timore. Kata ka in gi kwan kuet ma malo klinik biro hoyi mondo gidwok kwan kute obed piny mogik.

**E-navigation,** ma en kony mar mbese ma ukalo go e gimachal e yor simb ong’we yamo kod oro ote ma chowk ma itimo gi kompyuta. Ote machwok ibiro orni ma wuoyo kuom kaka inyalo rito ngamini. Jal ma nigi kuet mag ayaki kendo kawo yedhe ge kaka dwarore biro konyi , e yor miyi weche kod hocho mondo imed dhi nyime gi thieth. Tudruok gi jal ma nigi kute mag ayaki kendo mwonyo yedhe ge kaka dwarore biro bedo e yor ote machwok kata wuoyo e sime . Tudruok ni nyalo bedo e yor gige sime ma ihero , kuom ranyisi e yor ote machwok mar sime ma pile, What’s App, Snapchat, kata Facebook Messenger. Tudruok ni biro bedo machiegni e dweche ariyo mokwongo kendo bang’mano to dwe ka dwe , gi nyalo mar wuoyo mathoth mapile ka dwarore.

Yore konyo kaka thieth ma pile kata e-navigation biro bedo kuom higa achiel ka ok ilewo ne limbe kod ndalo mokadho 14 KATA in gi kwan ma malo mar kute.

Wabiro dhi nyime gi luwi e nonro kuom thuolo mar highni ariyo , bang’rwaki e nonro.

*Ka ilewo ne limbe klinik kod ndalo mokalo 14 KATA in gi kwan ma malo mar kute > 1000 copies/ml:*

Ka ilewo ni limbe klinik gi ndalo 14 KAT in gi kwan ma malo mar kute, kata obedo ne iyudo yor konyo mane mokwongo, wabiro manyi e gweng’. Ka wanyalo tudruok kodi, wabiro penji penjo kendo , penjo kuom kaka imor kod gigo ma ineno kuom yore mag konyo ma nyalo bedo ni iyudo , gik ma omiyo ne ok iduogo , kod ka isewilori ma idhi e kar thieth machielo. Nonro mar penjo ni inyalo tim e kar thieth kata e gweng’. Nonro mar penjo ni biro kawo madirom dakika 45-60. Ka ok wanyal yudi, wabiro temo wuoyo kod ngát mane ichiwo ni wanyalo wuoyo godo mondo wang’e kama intiere.

Ka okwatudre kodi , wabiro keti e yor radharadha kendo e kidieny achiel kuom adek mag yore ma konyo gi mondo okony dwaki kendo e thieth:

- **“Thieth ma pile”.** Ma en ni ibiro yudo weche kuom ber mar medo bedo e thieth kendo jip mondo iduog e klinik. Ka ok wanyalo tudre kodi iwuon, klinik biro temo mondo oyudi e yor sime kata limbe ma e dala ma en gima itimo e thieth mapile. Kata ka in gi kwan kute ma malo klinik biro hoyi mondo odwok kwan kute go piny mogik.
- **Chiwo pesa ka itimo gima idwaro.** Ka oketi e chiw pesa ka itimo gima idwaro , jotij nonro biro chiwo silings ma Kenya 500/= ne in kuom biro e limbe klinik eyi thuol mar ndalo 3 kendo silings 500/= machielo mar dwoko piny kwan mar kute nyadiriyo e higa. Inyalo yiero mar yudo pesa e yor Mpesa kata omwom ma imiyi e lweti.
- **Jal ma mbasni ma miyi kony kata hocho owuon.** Ka oketi e kidieny mar yudo kony kowuok kuom jogo ma osedak gi kute mag ayaki kendo mwonyo yedhe gi kaka dwarore , ibiro keti e kony ma ichiwo gi ngáma mbasni ma nigi kuet ayaki kendo mwonyo yedhe ge kaka dwarore gi ngáma chal kodi e york et kod higni ma dwa romre . Ma biro bedo ng’ato ma owuok e klinik ma biro dwaro konyi , e yor miyi weche , hocho kod siri e oganda u, mondo imed bedo e thieth kendo ibed gi kwan kute man piny ahinya. Ngátni biro tudre kodi en owuon kod ka nyalore e yor sime. Ngátni biro room kodi mondi kod jarit kata janyuol mari e odu kendo room kodi dwe ka dwe e kama iyiero . Romo gi nyalo bedo tin kata ngeny e dwe ka dwe, to ok ma okalo juma ka juma. Ka imedo bedo gi pek kaka limbe m aok ibiro e omedore kata kwan kute ni malo, ng’atni biro dhi nyime tiyo kodi mondo okonyo ikal pek gi.

Yore mag thieth ma pile, chiwo pesa ka itimo gima idwaro, kata ngáto ma konyi owuon biro bedo kuom higa achiel koluwo keti e kidieny machielo.

Wabiro dhi nyime luwi e nonro ni kuom thuoo mar higni ariyo , bang’rwaki e nonro.

*Higa achiel kod higni ariyo bang’ rwaki e nonro*

Wabiro penji penjo kuom kaka idak, ngimani kod kaka idak kod ji, kod ngéyo ma in godo kuom kute mag ayaki kod mwonyo yath akak dwarore, kod kaka imor gi kod gik ineno kuom yore mag konyo ma nyalo bedo ni ne iyudo e nonro e higa achiel kod higni ariyo bang’ka oserwaki e nonro. Nonro mar penjo ni biro kawo madirom dakika 45-60.

*E thuolo nonro duto*

Ibiro dhi nyime gi yudo thieth mari e klinik kaka pile. Weche ma ochok limbe mag klinik ma pile ibiro kaw kowuok e andike gi mag thieth (kaka. Tarik mane orwaki, chal mar ngimani koriwo nyaka kwan mar kute, kod tarik ma ochaki e yedhe mag ayaki).

Ka klinik ni pok opimo kwan mar kute mag ayaki e rembi kaka chenro mag serikal ma Kenya dwaro, wabiro kwayi mondo ichiw remo moko ne pim mar kiwango mar kute e dendi, ma biro dwaro mdirom mL5 (madirom ojiko achiel mar chae) mar remo dweche 6 ka dweche 6. Ka in e klinik e thuolo no, remo ibiro kaw e yo mapile no e kar thieth. Ka ok ibi e klinik, wabiro manyi kendo kwayi mondo ichiw gir pim ne ngáto ma nyalo kawo remo oko mar kar thieth. Dwoko mag pim mar kwan kute biro yudore ne in e klinik.

Bende wabiro ng’iyo andike gi mag thieth mondo wane weche kuom thieth mari kaka limbe gi mag klinik, mondo wang’e ka onego wamanyi. Wabiro tiyo gi weche ma ondik e andike gi mag klinik mondo wang’e kaka weche gi chacho nyalo mari mar medo biro e thieth.

Ka po ni waketo lokruok moro amora e nonro, ibiro nyisi. Bende, ka wach manyien ofwenyore kuom nonro ma onego ing’e, wabiro nyisi.

**Rach**

Wachano ng’iyo kiwango mar kute mag ayaki e dendi nyadichiel e nonro ni ka klinik pok otimo mano. Bende nitiere rach mar rem kata winjo lit kawuok e kawo remo. Nitiere rach ma thuolo matin ahinya mar gamo tuo kawuok e kawo remo. Ne weche mathoth kuom rach kod rach ma thenge, penj achiel kuom lakteche man e klinik.

Nitie nyalo ni ngáto nyalo neno ote machwok ma waoroni. Ote machwok ma sime go ok wacho gimoro amora kuom kute mag ayaki kendo ka ng’ato machielo nyalo yudo simbi ok gibing’enyo gimoro amora kuom chal mari mar kute mag ayaki. Kata kamano, mae nyalo kelo ni wichkuot, kendo dwaher mondo ing’e ni rach ni nyalo bedo kapok iyiero mar bedo e nonro.

Nitiere bende rach ni moko kuom penjo ma wapenjo kuom kaka iwinjo kod gigo ma isekalo e thieth nyalo miyo ok ibed thuolo. In thuolo mar weyo dwoko penjo moro amora ma ok diher dwoko kata weyo bedo e nonro e saa asaya.

Rach machielo en ni jomoko nyalo fwenyo ni in e nonro ni kata neno andike gi mag thieth kata weche gi mag thieth mopondo, kaka chal mari mar kute mag ayaki . Wabiro timo kar nyalowa mondo wane ni ma ok otimore. Jotij nonro kod mbese ma jiwo ji gin jok ma otieg e yo ma kende e keto weche gi mopondo kendo rito weche gi mondo jomoko kik ng’e.

**Ber**

Nyalore ni ok ibi yudo ber mochimore kodi tir kuom bedo e nonro ni. Inyalo yudo ote machwok kod kony mar mbese ma jiwo ji e yor ong’we yamo, chiwo pesa, kata ngát ma tayi ji ma biro en owuon . Chenro mag konyo gi nyalo konyi medo bedo e thieth kendo mano nyalo konyo ngimani. E giko nonro , dwoko biro konyowa ng’eyo yo maber mogik kata riwruok mar yore ma wanyalo tiyo godo e konyo ji mondo omed bedo ka gin e thieth.

**Dwoko pesa**

**Ibiro dwokni pesa mar wuoth mar silings 500/= ma Kenya seche ma orwaki e nonro .**

**Ka oketi e yor chiwo pesa ka itimo gima dwarore ibiro yudo silings . 500/= ne biro e limbe mag klinik ma ochan e kind thuolo mar ndalo 3 kendo bedo kod kwan mar kute mar ok fwenyore 2 e higa achiel.**

**Yore mamoko**

In thuolo mar yiero mondo kik ibed e nonro . Ka iyiero mondo ikik ibed e nonro ni, onge kum ma ibiro miyi . Ok ibi wito ber ma iyudo ga pile ka pile , kendo podi inyalo yudo thieth kowuok e klinik mari kaka ijayudo ga.

**Maling’ling’**

Wabiro timo ka nyaowa mondo wane ni weche gi manyalo fwenyi ma ochok ne nonro okan mopondo. Kata kamano, ok wanya singo maling’ling’ma chutho. Weche gi manyalo fwenyi inyalo chiw ka idwaro e yor chik. Ka wach ma owuok kuom nonro ni ondiki kata osom e buche mag syans, nyingi kod weche gi mamoko ma nyalo fwenyi ok bi ti godo.

Jotij serikal kata jotij mbalariany seche moko ngíyo nonro kaka mae mondo gine ni itimo gi e yo makare kendo kaluwore gi chik. Ka ng’icho e yo machal kama obedo, andike gi inyalo ng’i. Jok ma ng’iyo nonro gi biro rito maling’ling’ mari . Andike mag nonro ok bi ti godo e keti e rach motudore gi chik. Migepe ma nyalo ng’iyo kendo /kata kopo andike gi mag nonro e yor nonro, neno ni otim nonro e yo makare, kod ng’iyo weche ma ochoki oriwo:

- The Washington University, St. Louis
- The Kenya Medical Research Institute
- University of California San Francisco
- University of Colorado, Denver
- University of California, Berkeley

Bedo e nonro ni nyalo kelo wito maling’ling’, kata kamano wabiro keto yore mag neno ni warito maling’ling’ mari. Andike mar thieth ibiro los nikech bedo ni e nonro ni. Andike mar yie mari kod moko kuom pim magi mg nonro ibiro ket e andike ni. Nikech mano, jochiw thieth magi mamoko nyalo neno dwoko mag pim magi kendo bedo kod n’eyo kuom bedo mari e nonro. Chike mag osibtal dwaro ni jochiw thieth duto keto weche man e andike mag thieth maling’ling’. Wach moro amora momedore kuom nonro ibiro miyi koriwo nyaka dwoko mogik mag nonro.

**Bedo e nonro**

Inyalo yiero mar weyo bedo e nonro e saa asaya maonge kum kata wito ber ma dine iyudo. Nyis anyisa jatim nonro kata jatij nonro sanosano ni diher mar weyo bedo e nonro. Inyalo weyo bedo e nonro ka ioro ote machwok mondik ni “STOP” e namba ma wabiro miyi. Bende, jatim nonro nyalo goli e nonro ni e saa asaya ka oneno ni en kuom ber mari iwuon, ka ok iluw chike mag nonro, kata ka nonro ochungi.

**Jogo ma ochiwo pesa**

Nonro ni ochiw ne pesa kod National Institutes of Health. Onge moro amora kom jotim nonro man malo kanyo man kod chachni moro amora motudore gi yore pesa ma miyo dwaro mar gi nyalo chachni gi nonro. E thuolo ma nonro nyalo bedo ni ochung kowuok kuom yiero mar jatim nonro kata jachiw pesa, ibiro nyisi gima biro timore tok mano.

**Yor tudruok**

Inyalo wuoyo kod jotim nonro kuom penjo, parruok, kata ywagruok moro amora ma in go kuom nonro ni. Tudri kod jatij wa mar nonro e 0733-819 835. Ka diher mar penjo penjo kuom nonro kata ratiro magi kaka jachiwre ne ng’ato machielo kopogore gi jotim nonro kata diher mar wacho chandruok kata parruok moro amora ma in go kuom nonro , inyalo tudri gi Jagoro mar Ethical Review Committee, Kenya Medical Research Institute e seru@kemri.org kata P.O. BOX 54840-00200 Sime. 020-2722541, 0717719477. Bende inyalo tudri kod Washington University Human Research Protection Office e +1-314-747-6800.Komiti gi odich gi rito jochiwre mag nonro.

**YIE**

Osemiyi kopi mar andike mar yie ni mondo ikan.

**BEDO E NONRO EN KUOM CHIWRUOK.** In gi ratiro mar weyo bedo e nonro ni, kata wuok e saa asaya maonge kum moro amora kata wito ber ma dine iyudo.

**Wach ma oa kuom Jachiwre**

Nonro ni ma owuo kuome malo kanyo oselerna. Achiwora mar bedo e nonro ni. Aseyudo thuolo mar penjo penjo. Ka an gi penjo e kinde mabiro kuom nonro ni, anyalo penjo jotim nonro ma ondik malo kanyo. Ka an gi penjo kuom ratiro maga kaka jachiwre nonro, anyalo tudra kod jok ma ondik malo kanyo.

Ayie mondo weche maga ma ochok oti godo e nonro ma kinde mabiro kendo ochiw ne jotim nonro mamoko maonge yie machielo kod/maonge gigo manyalo fwenya.

Ka diher mar bedo e nonro ni, onego iket seyi piny kanyo.

Tarik	Nying Jachiwre kod Seyi mar Chiwo Yie

Tarik	Ng’at ma Kawo Yie (Nying gi Seyi)

***Ka ok onyal ndiko gi somo***

Aseneno somo mar andike mar yie ni e yo ma kare ne jachiwre kendo ng’atno osebedo gi thuolo mar penjo penjo. Asiro ni ng’atno ochiwo yie mare ka en thuolo**.**

Nying Janeno _____________________ KOD

Seyi janeno ***______________________***

Nying jachiwre __________________ KOD	Alama mar Lith Lwedo mathuon

Tarik ________________________

Kopi ne:	1. Jotim nonro

2. Jachiwre nonro

**DHOLUO OVERALL INFORMED CONSENT FORM**

**KENYA MEDICAL RESEARCH INSTITUTE**

**WASHINGTON UNIVERSITY, ST. LOUIS**

**CONSENT TO PARTICIPATE IN A RESEARCH STUDY**

**Study Title:** *Adaptive Strategies to Prevent and Treat Failures of Retention in Adolescent HIV Care (A4A Study)*

JOTIM NONRO KOD MIGEPE MA GITUDORE GODO:

| Jatim Nonro | Migao | Yor Tudruok |
| --- | --- | --- |
| Laktar Elizabeth Bukusi | Kenya Medical Research Institute | +254 733-617 503 |
| Laktar Elvin Geng | Washington University, Missouri | +1-415-430-5589 (USA) |
| Laktar Lisa Abuogi | University of Colorado, Denver | +1-303-724-7430 (USA) |
| Laktar Zachary Kwena | Kenya Medical Research Institute | +254 722-386 714 |
| Starley Shade | University of California, San Francisco | +1-415 794-4354 (USA) |
| Lina Montoya | University of Notrh Carolina at Chapel Hill | +1-4105857470 (USA) |
| Eliud Akama | Kenya Medical Research Institute | +254 728-590 376 |

**Kama itimo e Nonro:** Kisumu County

Ikwayi mondo ibed e nonro. Boksi ma piny no nyisi gik maber ma onego ipar kapok iyiero mar donjo e nonro . Wabiro miyi wach matut e bwo boksi no. Yie ipenj penjo kuom wach moro amora kapok iyiero mar donjo . Bende inyalo dwaro wuoyo gi jomoko (kuom ranyisi, anyuola ni, osiepe gi, kata laktar mari ) kuom nonro ni, kapok iyie mar bedo e nonro ni.

- **Yie kuom chiwruok** . Ikwayi mondo cihiwri ne nonro. En kuomi ka iyiero mar bedo koto weyo bedo. Onge kum kendo onge gimoro amora ibiro wit ka iyiero mar weyo donjo kata ka isedonjo , iyiero mar wuok.
- **Gima omiyo itimo**. Watimo nonro ni mondo wangé riwruok mane mar yore ma kelo yor tudruok maber e thieth mar kute mag ayaki kuom rowere kod jomadongo matindo. Bedo e thieth kendo keto kwan mar kute man e remo bedo maonge en gimaduong’ e neno ni rowere ma odak kod kute mag ayaki bedo ka gingima. To, rowere kod jomadongo matindo bedo kod gigo mathoth ma mono gi bedo e thieth kaka dwarore kendo bedo ka gimedo yudo thieth. E nonro ni watemo ng’eyo kaka inyalo mi rowere kod jomadongo matindo chenro ma konyo gi bedo e thieth kendo bedo gi kwan mar kute mag ayaki mapiny mogik.
- **Jochiwre.** Madirom rowere kod jomadongo matindo 880 man e kind higni ma odak gi kute mag ayaki e e Kisumu County biro bedo e nonro ni.
- **Thuolo.** Bedo ni e nonro ni biro kawo madirom higni 2.
- **Chenro kod gik ma itimo.**

E nonro ni, ibiro ng’I ka inyalo donjo e nonro kendo ibiro keti a achiel kuom yore ariyo mag konyo e yor radharadha , thieth mapile kata jipo ma itimo gi mbese e yor kompyuta(ote machwok ma ioro gi kompyuta kod kony ma ichiwo gi mbese e yor sime). Ka ilewo ne limbe mag klinik gi ndalo mokalo 14 KATA bedo gi kwan ma malo mar kute , ibiro keti e yor radharadha e kidieny mar thieth ma pile , pesa ma ichiwo ka itimo gima idwaro (yudo pesa), kata mbasni ma konyo ji ka obiro owuon .

Wabiro penji penjo kuom kaka idak, ngimani kod kaka idak gi ji, kod ngéyo ma in godo kuom kute mag ayaki gi kawo thieth kaka dwarore. Penjo gi biro bedo e thuolo ma irwaki e nonro, ka oketi e kidieny machielo (ka ilewo biro e klinik kuom ndalo 14 KATA in kod kwan kute man malo), kendo ka isebedo e nonro kuom higa 1 kendo bang’ higni 2. Bende wabiro penji penjo kuom kaka ineno kod gigo ma isekalo e kuom yore mag konyo ma iseyudo ka oketi e kidieny machielo, bang’ka isebedo e nonro kuom higa 1 kendo bang’higni 2. Penjo gi ibiro tim a kar thieth to inyalo tim gi e gweng’ka dwarore. Penjo gi biro kawo madirom dakika 45-60.

Bende wabiro kawo weche ma ochok ma pile e klinik kowuok e andike gi mag thieth. Ka kwan mari mar kute ok okaw e klinik ka oengo okawe , nonro nyalo tudre kodi kendo kawo remo ne pim mar kwan kute madirom dweche 6 ka dweche 6.

- **Rach.**
- Rem kata lit matin kowuok e golo remo.
- Fwenyruok ma ok ochan mar chal mar kute mag ayaki. Kata kamano, wabiro keto yore mag geng’o mondo wane ni chal mari mar kute mag ayaki orit.
- **Ber**. Ok inyal yudo ber mochimore kodi tir kuom bedo e nonro ni. Inyalo yudo ote machwok mar simo (SMS) kod tudruok gi mbese e yor sime, yudo pesa, kata bedo kod mbas ma jiwo ji ma ubet godo wang’gi wang’ . Chenro mag konyo gi nyalo konyi bedo e thieth mar kute mag ayaki knedo kawo yedhe gi maber kendo kaluwore gi mano bende nyalo konyo ngimani. Komedore, gik ma wafwenyo kuom nonro ni biro konyo keto maber thieth mar kute mag ayaki kuom rowere obed maber.
- **Yiero mamoko.** In thuolo mar yiero mondo kik ibed e nonro. Ka iyiero mar ni kik ibed e nonro ni, onge kum ma ibiro miyi. Ok ibi wito ber moro amora ma magi mapile , kendo podi inyalo yudo thieth e kilini mari kaka iyudo ga..

**Chenro kod gik ma itimo**

Ka iyie mar bedo e nonro ni, chenro moluwo gi biro timore:

**E rwako ji e nonro**

Wabiro penji penjo kuom kaka idak , ngimani to gi kaka idak gi ji , kod ng’eyo kuom kute mag ayaki kod mwonyo yath kaka dwarore. Nonro mar penjo ni biro kawo madirom dakika 45-60**.**

Ibiro keti e achiel kuom yore ariyo mag jiwo jotuo. Yiero yo ma ibiro yudo itimo e yor radharadha, ya machal gi wito otonglo malo ka ing’iyo kama olwar godo. Yore mag konyo go gin:

- **“Thieth mapile”** ma en ni ka ilewo ma ok ibiro kaka ochan, yo mapile mar klinik mar paroni mondo ibi e limbe ema biro timore. Kata ka in gi kwan kuet ma malo klinik biro hoyi mondo gidwok kwan kute obed piny mogik.
- **E-navigation,** ma en kony mar mbese ma ukalo go e gimachal e yor simb ong’we yamo kod oro ote ma chowk ma itimo gi kompyuta. Ote nachwok ibiro orni ma wuoyo kuom kaka inyalo rito ngamini. Jal ma nigi kuet mag ayaki kendo kawo yedhe ge kaka dwarore biro konyi , e yor miyi weche kod hocho mondo imed dhi nyime gi thieth. Tudruok gi jal ma nigi kute mag ayaki kendo mwonyo yedhe ge kaka dwarore biro bedo e yor ote machwok kata wuoyo e sime . Tudruok ni nyalo bedo e yor gige sime ma ihero , kuom ranyisi e yor ote machwok mar sime ma pile, What’s App, Snapchat, kata Facebook Messenger. Tudruok ni nyalo bedo tin kata nyeny e dweche ariyo mokwongo kendo bang’mano to dwe ka dwe , gi nyalo mar wuoyo mathoth mapile ka dwarore.

Yore konyo kaka thieth ma pile kata e-navigation biro bedo kuom higa achiel ka ok ilewo ne limbe kod ndalo mokadho 14 KATA in gi kwan ma malo mar kute.

Wabiro dhi nyime gi luwi e nonro kuom thuolo mar highni ariyo , bang’rwaki e nonro.

*Ka ilewo ne limbe klinik kod ndalo mokalo 14 KATA in gi kwan ma malo mar kute > 1000 copies/ml:*

Ka ilewo ni limbe klinik gi ndalo 14 KAT in gi kwan ma malo mar kute, kata obedo ne iyudo yor konyo mane mokwongo, wabiro manyi e gweng’. Ka wanyalo tudruok kodi, wabiro penji penjo kendo, penjo kuom kaka imor kod gigo ma ineno kuom yore mag konyo ma nyalo bedo ni iyudo , gik ma omiyo ne ok iduogo , kod ka isewilori ma idhi e kar thieth machielo. Nonro mar penjo ni inyalo tim e kar thieth kata e gweng’. Nonro mar penjo ni biro kawo madirom dakika 45-60. Ka ok wanyal yudi, wabiro temo wuoyo kod ngát mane ichiwo ni wanyalo wuoyo godo mondo wang’e kama intiere.

Ka okwatudre kodi, wabiro keti e yor radharadha kendo e kidieny achiel kuom adek mag yore ma konyo gi mondo okony dwaki kendo e thieth:

- **“Thieth ma pile”.** Ma en ni ibiro yudo weche kuom ber mar medo bedo e thieth kendo jip mondo iduog e klinik. Ka ok wanyalo tudre kodi iwuon, klinik biro temo mondo oyudi e yor sime kata limbe ma e dala ma en gima itimo e thieth mapile. Kata ka in gi kwan kute ma malo klinik biro hoyi mondo odwok kwan kute go piny mogik.
- **Chiwo pesa ka itimo gima idwaro.** Ka oketi e chiw pesa ka itimo gima idwaro , jotij nonro biro chiwo silings ma Kenya 500/= ne in kuom biro e limbe klinik eyi thuol mar ndalo 3 kendo silings 500/= machielo mar dwoko piny kwan mar kute nyadiriyo e higa. Inyalo yiero mar yudo pesa e yor Mpesa kata omwom ma imiyi e lweti.
- **Jal ma mbasni ma miyi kony kata hocho owuon.** Ka oketi e kidieny mar yudo kony kowuok kuom jogo ma osedak gi kute mag ayaki kendo mwonyo yedhe gi kaka dwarore , ibiro keti e kony ma ichiwo gi ngáma mbasni ma nigi kuet ayaki kendo mwonyo yedhe ge kaka dwarore gi ngáma chal kodi e york et kod higni ma dwa romre . Ma biro bedo ng’ato ma owuok e klinik ma biro dwaro konyi , e yor miyi weche , hocho kod siri e oganda u, mondo imed bedo e thieth kendo ibed gi kwan kute man piny ahinya. Ngátni biro tudre kodi en owuon kod ka nyalore e yor sime. Ngátni biro room kodi mondi kod jarit kata janyuol mari e odu kendo room kodi dwe ka dwe e kama iyiero . Romo gi nyalo biro piyo piyo moloyo dwe ka dwe, to ok ma okalo juma ka juma. Ka imedo bedo gi pek kaka limbe m aok ibiro e omedore kata kwan kute ni malo, ng’atni biro dhi nyime tiyo kodi mondo okonyo ikal pek gi.

Yore mag thieth ma pile, chiwo pesa ka itimo gima idwaro, kata ngáto ma konyi owuon biro bedo kuom higa achiel koluwo keti e kidieny machielo.

Wabiro dhi nyime luwi e nonro ni kuom thuoo mar higni ariyo , bang’rwaki e nonro.

*Higa achiel kod higni ariyo bang’ rwaki e nonro*

Wabiro penji penjo kuom kaka idak, ngimani kod kaka idak kod ji, kod ngéyo ma in godo kuom kute mag ayaki kod mwonyo yath akak dwarore, kod kaka imor gi kod gik ineno kuom yore mag konyo ma nyalo bedo ni ne iyudo e nonro e higa achiel kod higni ariyo bang’ka oserwaki e nonro. Nonro mar penjo ni biro kawo madirom dakika 45-60.

*E thuolo nonro duto*

Ibiro dhi nyime yudo theith mari e klinik kaka pile. Weche mochok kowuok e limbe klinik ma pile ibiro kaw kowuok e andike gi mag thieth (e.g. Tarik mane orwaki, chal mar ngimani koriwo nyaka kwan mar kute, kod tarik ma ochaki e yedhe mag ayaki).

Ka klinik ni pok opimo kwan mar kute mag ayaki e rembi kaka chenro mag serikal ma Kenya dwaro, wabiro kwayi mondo ichiw remo moko ne pim mar kiwango mar kute e dendi, ma biro dwaro mdirom mL5 (madirom ojiko achiel mar chae) mar remo dweche 6 ka dweche 6. Ka in e klinik e thuolo no, remo ibiro kaw e yo mapile no e kar thieth. Ka ok ibi e klinik, wabiro manyi kendo kwayi mondo ichiw gir pim ne ngáto ma nyalo kawo remo oko mar kar thieth. Dwoko mag pim mar kwan kute biro yudore ne in e klinik.

Bende wabiro ng’iyo andike gi mag thieth mondo wane weche kuom thieth mari kaka limbe gi mag klinik, mondo wang’e ka onego wamanyi. Wabiro tiyo gi weche ma ondik e andike gi mag klinik mondo wang’e kaka weche gi chacho nyalo mari mar medo biro e thieth.

Ka po ni waketo lokruok moro amora e nonro, ibiro nyisi. Bende, ka wach manyien ofwenyore kuom nonro ma onego ing’e, wabiro nyisi.

**Rach**

Wachano ng’iyo kiwango mar kute mag ayaki e dendi nyadichiel e nonro ni ka klinik pok otimo mano. Bende nitiere rach mar rem kata winjo lit kawuok e kawo remo. Nitiere rach ma thuolo matin ahinya mar gamo tuo kawuok e kawo remo. Ne weche mathoth kuom rach kod rach ma thenge, penj achiel kuom lakteche man e klinik.

Nitie nyalo ni ngáto nyalo neno ote machwok ma waoroni. Ote machwok ma sime go ok wacho gimoro amora kuom kute mag ayaki kendo ka ng’ato machielo nyalo yudo simbi ok gibing’enyo gimoro amora kuom chal mari mar kute mag ayaki . Kata kamano, mae nyalo kelo ni wichkuot , kendo dwaher mondo ing’e ni rach ni nyalo bedo kapok iyiero mar bedo e nonro.

Niere bende rach ni moko kuom penjo ma wapenjo kuom kaka iwinjo kod gigo ma isekalo e thieth nyalo miyo ok ibed thuolo. In thuolo mar weyo dwoko penjo moro amora ma ok diher dwoko kata weyo bedo e nonro e saa asaya.

Rach machielo en ni jomoko nyalo fwenyo ni in e nonro ni kata neno andike gi mag thieth kata weche gi mag thieth mopondo , kaka chal mari mar kute mag ayaki . Wabiro timo kar nyalowa mondo wane ni ma ok otimore. Jotij nonro kod mbese ma jiwo ji gin jok ma otieg e yo ma kende e keto weche gi mopondo kendo rito weche gi mondo jomoko kik ng’e.

**Ber**

Nyalore ni ok ibi yudo ber mochimore kodi tir kuom bedo e nonro ni. Inyalo yudo ote machwok kod kony mar mbese ma jiwo ji e yor ong’we yamo, chiwo pesa, kata ngát ma tayi ji ma biro en owuon . Chenro mag konyo gi nyalo konyi medo bedo e thieth kendo mano nyalo konyo ngimani. E giko nonro , dwoko biro konyowa ng’eyo yo maber mogik kata riwruok mar yore ma wanyalo tiyo godo e konyo ji mondo omed bedo ka gin e thieth.

**Dwoko pesa**

**Ibiro dwokni pesa mar wuoth mar silings 500/= ma Kenya seche ma orwaki e nonro .**

**Ka oketi e yor chiwo pesa ka itimo gima dwarore ibiro yudo silings . 500/= ne biro e limbe mag klinik ma ochan e kind thuolo mar ndalo 3 kendo bedo kod kwan mar kute mar ok fwenyore 2 e higa achiel.**

**Yore mamoko**

In thuolo mar yiero mondo kik ibed e nonro . Ka iyiero mondo ikik ibed e nonro ni, onge kum ma ibiro miyi . Ok ibi wito ber ma iyudo ga pile ka pile , kendo podi inyalo yudo thieth kowuok e klinik mari kaka ijayudo ga.

**Maling’ling’**

Wabiro timo ka nyaowa mondo wane ni weche gi manyalo fwenyi ma ochok ne nonro okan mopondo. Kata kamano, ok wanya singo maling’ling’ma chutho. Weche gi manyalo fwenyi inyalo chiw ka idwaro e yor chik. Ka wach ma owuok kuom nonro ni ondiki kata osom e buche mag syans, nyingi kod weche gi mamoko ma nyalo fwenyi ok bi ti godo.

Jotij serikal kata jotij mbalariany seche moko ngíyo nonro kaka mae mondo gine ni itimo gi e yo makare kendo kaluwore gi chik. Ka ng’icho e yo machal kama obedo, andike gi inyalo ng’i. Jok ma ng’iyo nonro gi biro rito maling’ling’ mari . Andike mag nonro ok bi ti godo e keti e rach motudore gi chik. Migepe ma nyalo ng’iyo kendo /kata kopo andike gi mag nonro e yor nonro, neno ni otim nonro e yo makare, kod ng’iyo weche ma ochoki oriwo:

- The Washington University, St. Louis
- The Kenya Medical Research Institute
- University of California San Francisco
- University of Colorado, Denver
- University of California, Berkeley

Bedo e nonro ni nyalo kelo wito maling’ling’, kata kamano wabiro keto yore mag neno ni warito maling’ling’ mari. Andike mar thieth ibiro los nikech bedo ni e nonro ni. Andike mar yie mari kod moko kuom pim magi mg nonro ibiro ket e andike ni. Nikech mano, jochiw thieth magi mamoko nyalo neno dwoko mag pim magi kendo bedo kod n’eyo kuom bedo mari e nonro. Chike mag osibtal dwaro ni jochiw thieth duto keto weche man e andike mag thieth maling’ling’. Wach moro amora momedore kuom nonro ibiro miyi koriwo nyaka dwoko mogik mag nonro.

**Bedo e nonro**

Inyalo yiero mar weyo bedo e nonro e saa asaya maonge kum kata wito ber ma dine iyudo. Nyis anyisa jatim nonro kata jatij nonro sanosano ni diher mar weyo bedo e nonro. Inyalo weyo bedo e nonro ka ioro ote machwok mondik ni “STOP” e namba ma wabiro miyi. Bende, jatim nonro nyalo goli e nonro ni e saa asaya ka oneno ni en kuom ber mari iwuon, ka ok iluw chike mag nonro, kata ka nonro ochungi.

**Jogo ma ochiwo pesa**

Nonro ni ochiw ne pesa kod National Institutes of Health. Onge moro amora kom jotim nonro man malo kanyo man kod chachni moro amora motudore gi yore pesa ma miyo dwaro mar gi nyalo chachni gi nonro. E thuolo ma nonro nyalo bedo ni ochung kowuok kuom yiero mar jatim nonro kata jachiw pesa, ibiro nyisi gima biro timore tik mano.

**Yor tudruok**

Inyalo wuoyo kod jitm nonro kuom penjo, parruok, kata ywagruok moro amora ma in go kuom nonro ni. Tudri kod jatij wa mar nonro e 0733-819 835. Ka diher mar penjo penjo kuom nonro kata ratiro magi kaka jachiwre ne ng’ato machielo kopogore gi jotim nonro kata diher mar wacho chandruok kata parruok moro amora ma in go kuom nonro, inyalo tudri gi Jagoro mar Ethical Review Committee, Kenya Medical Research Institute e seru@kemri.org kata P.O. BOX 54840-00200 Sime. 020-2722541, 0717719477. Bende inyalo tudri kod Washington University Human Research Protection Office e +1-314-747-6800.Komiti gi odich gi rito jochiwre mag nonro.

**YIE**

Osemiyi kopi mar andike mar yie ni mondo ikan.

**BEDO E NONRO EN KUOM CHIWRUOK.** In gi ratiro mar weyo bedo e nonro ni, kata wuok e saa asaya maonge kum moro amora kata wito ber ma dine iyudo.

**Wach ma oa kuom Jachiwre**

Nonro ni ma owuo kuome malo kanyo oselerna. Achiwora mar bedo e nonro ni. Aseyudo thuolo mar penjo penjo. Ka an gi penjo e kinde mabiro kuom nonro ni, anyalo penjo jotim nonro ma ondik malo kanyo. Ka an gi penjo kuom ratiro maga kaka jachiwre nonro, anyalo tudra kod jok ma ondik malo kanyo.

Ayie mondo weche maga ma ochok oti godo e nonro ma kinde mabiro kendo ochiw ne jotim nonro mamoko maonge yie machielo kod/maonge gigo manyalo fwenya .

Ka diher mar bedo e nonro ni, onego iket seyi piny kanyo.

Tarik	Nying Jachiwre kod Seyi mar Chiwo Yie

Tarik	Ng’at ma Kawo Yie (Nying gi Seyi)

***Ka ok onyal ndiko gi somo***

Aseneno somo mar andike mar yie ni e yo ma kare ne jachiwre kendo ng’atno osebedo gi thuolo mar penjo penjo. Asiro ni ng’atno ochiwo yie mare ka en thuolo**.**

Nying Janeno _____________________ KOD

Seyi janeno ***______________________***

Nying jachiwre __________________ KOD Alama mar Lith Lwedo mathuon

Tarik ________________________

Kopi ne:	1. Jotim nonro

2. Jachiwre nonro

**DHOLUO OVERALL PARENTAL CONSENT FORM**

**KENYA MEDICAL RESEARCH INSTITUTE**

**WASHINGTON UNIVERSITY, ST. LOUIS**

**PARENTS/GUARDIAN PERMISSION FORM**

**FOR RESEARCH PARTICIPANTS 14 – 17 YEARS OLD**

**Study Title:** *Adaptive Strategies to Prevent and Treat Failures of Retention in Adolescent HIV Care (A4A Study)*

JOTIM NONRO KOD MIGEPE MA GITUDORE GODO:

| Jatim Nonro | Migao | Yor Tudruok |
| --- | --- | --- |
| Laktar Elizabeth Bukusi | Kenya Medical Research Institute | +254 733-617 503 |
| Laktar Elvin Geng | Washington University, Missouri | +1-415-430-5589 (USA) |
| Laktar Lisa Abuogi | University of Colorado, Denver | +1-303-724-7430 (USA) |
| Laktar Zachary Kwena | Kenya Medical Research Institute | +254 722-386 714 |
| Starley Shade | University of California, San Francisco | +1-415 794-4354 (USA) |
| Lina Montoya | University of Notrh Carolina at Chapel Hill | +1-4105857470 (USA) |
| Eliud Akama | Kenya Medical Research Institute | +254 728-590 376 |

**Kama itmo e Nonro:** Kisumu County

Wakwayo nyathini mondo obed e nonro . Boksi man piny kanyo nyisi gik ma ber ma onego ipar kapok ichiwo thuolo ar nyathini bedo e nonro. Wabiro miyi wach matut e bwo boksi man piny ka. Yie ipenj penjo kuom wach moro amora kapok ichiwo thuolo. Bende inyalo dwaro mar wuoyo kod jomoko (kuom ranyisi , anyuola ni , osiepe, kata laktar mari) kuom nonro ni, kapok ichiwo yie.

- **Yie kuom chiwruok**. Ikwayi mondo iyie ne nyathini obed e nonro . Nyathini ok bi yudo kum moro amora kuom weyo bedo e nonro kata wuok e nonro e saa asaya.
- **Gima omiyo itime**. Watimo nonro ni mondo wangé riwruok mag yore ma miyo rowere kod jomadongo matindo medo bedo e thieth mar kute mag ayaki. Bedo e thieth kendo keto kwan mar kute man e remo bedo maonge en gimaduong’ e neno ni rowere ma odak kod kute mag ayaki bedo ka gingima. To, rowere kod jomadongo matindo bedo kod gigo mathoth ma mono gi bedo e thieth kaka dwarore kendo bedo ka gimedo yudo thieth. E nonro ni watemo ng’eyo kaka inyalo mi rowere kod jomadongo matindo chenro matiyo nigi maber mogik, e thuolo ma obiro konyo gi ahinya.
- **Jochiwre.** Madirom rowere kod jomadongo matindo 880 man e kind higni 14-24 ma odak gi kute mag ayaki e Kisumu County biro bedo e nonro ni.
- **Thuolo.** Nyathini biro bedo e nonro ni kuom thuolo madirom higni 2.
- **Chenro kod gik ma itimo.**

E nonro ni, nyathini ibiro ngí ka nyalo donjo e nonro kendo ibiro kete e yor radharadha e achiel kuom yore ariyo mag konyo ma itimo mokwongo, yor thieth ma pile kata kony mar mbese ma itimo e yor kompyuta/sime (ote machwok mag sime ma ioro gi kompyuta kod kony mar mbese ma itimo e yor simb ong’we yamo). Ka nyathini olewo ne limbe mare ma ochan mar klinik gi ndalo 14 KATA en gi kwan ma malo mar kute , ibiro kete e yor radharadha e yor thieth mapile , chiwo pesa ka ng’ato otimo gima idwaro (yudo pesa), kata bedo gi jal ma siro mbese wang’gi wang’ .

Nyathini ibiro penj penjo kuom kaka odak ,ngimane kod kaka odak kod ji , kod ng’eyo kuom kute mag ayaki kod mwonyo yath kaka dwarore. Nonro mar penjo ni biro bedo e kama irwaki e nonro , ka oketi e yor konyo machielo (ka olewo ne limbe klinik kod mokalo ndalo 14 KATA en gi kwan ma malo mag kute ) kendo bang’ka isebedo e nonro kuom higa 1 kendo bang’higni 2. Wabiro penjo nyathini penjo kuom kaka omor kod yore mag konyo gi kod gik ma osekalo e yore mag kony ma nyalo bedo ni oseyudo , ka okete e yor kony machielo , bang’ka osebedo e nonro kuom higa 1 kod kendo bang’higni 2 . Nonro mar penjo ni ibiro tim e kar thieth to bende inyalo time e gweng’ka dwarore. Nonro mar penjo ni biro kawo madirom dakika 45-60.

Wabiro kawo bende weche ma ochok e klinik kowuok e andike mage . Ka kwan mare mar kute pok okaw e klinik e thuolo ma onego bed ni ikawe , nonro nyalo tudre kode kendo kawo remo ne pim mar kwan mar kute e thuolo madirom dweche 6 ka dweche 6 .

- **Rach.**
- Rem kata lit matin kowuok e golo remo .
- Fwenyruok ma ok ochan mar chal mar kute mag ayaki. Kata kamano, wabiro keto yore mag geng’o mondo wane ni chal mar nyathini mar kute mag ayaki orit.
- **Ber**. Nyalore ni nyathini ok bi yudo ber mochimore kode tir kuom bedo e nonro ni. Nyathini nyalo yudo ote machwok mag sime kendo kony mar mbese e yor sime, chiwo pesa, kata hocho mar achiel ka achiel gi mbese ma siro ji . Yore mag konyo gi nyalo konyo nyathini mondo omed bedo e thieth kendo mwonyo yedhe ge maber kendo ma nyalo konyo ngimane . E wi mano , gigo ma wafwenyo kowuok kuom nonro ni biro konyo keto maber thieth mar rowere duto.
- **Yiero mamoko.** In thuolo mar yiero mar nyathini kik bed e nonro . Ka iyiero mondo kik odonj, onge kum ma ibiro mi nyathini . Nyathini ok bi wito ber ma oyudo ga mapile, kendo podi onyalo yudo thieth e klinik mare kaka ojayudo ga .

**Chenro gi gik ma itimo**

**Ka iyie mondo nyathini obed e nonro ni, kendo obe oyie , gik moluwo gi bir timore:**

**E rwako ji e nonro**

Wabiro penjo nyathini penjo kuom kaka odak, ngimane kod kaka odak kod ji , kod ng’eyo mare kuom kute mag ayaki gi mwonyo yath akak dwarore. Nonro mar penjo ni biro kawo madirom dakika 45-60**.**

Ibiro kete e achiel kuom yore ariyo mag konyo mondo ji omed bedo e thieth . Yiero mar yor konyo ma obiro bedo e biro bedo e yor radharadha , chenro machalo kod wito otonglo malo ka ing’iyo kama olawr godo. Yore mag konyo gi gin:

- **“Thieth ma pile”** ma en ni ka olewo ma ok obiro e limbe ne ma ochan , chenro mar klinik ma pile mar manye ema ibiro ti godo . Kata ka nyathini nigi kwan ma malo mar kute klinik biro hoye mondo oket kwan kute ne go odok piny mogik .
- **E-navigation,** ma en sir ma ichiwo gi mbese e yor simb ong’we yamo kod ote machwok mag sime ma ioro gi kompyuta. Ote machwok mag sime ibiro orne nyathini ma wuoyo kuom keto ngima ji bedo maber. Mbas ma tayo nyathini biro konyo nyathini , e yor miyo weche kod hocho mondo omed bedo e thieth. Tudruok gi mbasne ma ja hocho no biro bedo e yor ote ma ioro gi sime kata wuoyo e yor sime. Tudruok ni nyalo bedo e yor gir sim ma ohero , kuom ranyisi e yor ote machwok mar sim mapile, What’s App, Snapchat, kata Facebook Messenger. Tudruok nyalo bedo juma ka juma e dweche ariyo mokwongo kendo nyalo bedodwe ka dwe bang’ mano, gi nyalruok mar wuoyo moluwore ahinya ka dwarore.

Yor thieth mapile kata hocho mar mbese e yor kompyuta/sime biro bedo kuom higa achiel ka nyathini ok olewo ne limbe mag klinik gi thuolo mokalo ndalo 14 KATA nigi kwan ma malo mar kute .

Wabiro dhi nyime gi luwo nyathini e nonro ni kuom thuolo mar higni ariyo , bang’donjo e nonro.

*Ka nyathini olewo ne limbe klinik kod ndalo mokalo 14 KATA en gi kwan kute ≥ 1000 copies/ml:*

Ka nyathini olewo ne limbe ne mar klinik gi ndalo mokalo 14 KATA en gi kwan ma malo mar kute , kata obedo ni ne oyudo yor kony mane mokwongo , wabiro manye e gweng’ . Ka watudre kod nyathini wabiro penjo penjo mag nonro kendo , penjo kuom mor mare gi kod gik ma oseneno kuom yore mag kony ma nyalo bedo ni ne oseyudo , gik ma omiyo ok ne oduogo , kendo ka odar odhi e kar thieth machielo . Nonro mar penjo ni biro kawo madirom dakika 45-60. Ka ok wanyalo yudo nyathini , wabiro temo wuoyo kod ng’at ma ichiwo ni inyalo wuo godo mondo wang’e ni en kanye .

Ka okwatudre kod nyathini , wabiro kete e yo machielo mar kony e achiel kuom yore gi mag miyo nyathini odog e thieth	:

- **“Thieth ma pile”** ma en ni nyathini biro yudo weche kuom dhi nyime gi theith kod jip mar duogo e klinik. Ka ok wanyal tudruok gi nyathini owuon, klinik biro temo manyi e yor sime kata e dalal ma en gima itimo ga pile gi jo klinik. Kata ka nyathini nigi kwan ma malo mar kute klinik biro hoye mondo odwok kwan kute mage piny mogik .
- **Chiwo pesa ka itimo gima idwaro.** Ka nyathini oket e chiwo pesa ka itimo gima idwaro , jotij nonro biro chiwo silings ma Kenya 500/= ne nyathini kuom biro e limbe ne mochan eyi thuolo mar ndalo 3 kendo silings ma Kenya 500/= machielo mar keto kwan kute ge piny mogik 2 e higa . Nyathini nyalo yiero mar yudo pesa e yor Mpesa kata e lwedo.
- **Mbas ma tayo jowete ne wang’gi wang’.** Ka nyathini oket e yor mbas ma tayo jowete ne wang’gi wang’ , ibiro kete gi mbas ma tayo jowete ne wang’gi wang’machal kode e ket (dhako kata dichuo) kod higni . Mae biro bedo ngáto ma oa e klinik ma biro dwaro konyo nyathini , e yor chiwo wach , hocho kod siro ji e oganda u, mondo obed e thieth kendo obed gi kwan mar kute mapiny mogik. Ng’at ni biro tudre kod nyathini wang’gi wang’kod ka nyalore e yor sime. Ngát ni biro romo gi nyathini kod in e odu (kata kama uyiero ni ber nu) kendo romo kod nyathini dwae ka dwe e kama oyiero . Romo gi nyalo bedo moluwore machiegni , to ok mapiny ne juma ka juma. Ka nyathini obedo gi pek ma medo dhi nyime kaka limbe ma olewo mathoth kata kwam ma malo mar kute , ng’atni biro medo tiyo kode mondo okonye olo pek gi .

Thieth ma pile, pesa ma ichiwo ka itimo gima idwaro , kata hocho ma itimo ni gi ngáto owuon biro bedo kuom higa achiel bang’ka okete e yor konyo machielo .

Wabiro medo luow nyathini e nonro ni kuom thuolo mar higni ariyo , bang’rwake e nonro.

*Higa achielkod higni ariyo bang’rwake e nonro*

Wabiro penjo nyathini penjo mag nonro kuom kaka odak , ngimane kod kaka odak kod ji , kod ngéyo mare kuom kute mag ayaki kod mwonyo yath kaka dwarore kod kaka omor kod yore mag kony ma omiye kata gik ma oseneno e yore go ma nyalo bedo ni ne oyudo e nonro e higa achiel kod higni ariyo bang’ rwake e nonro . Penjo gi biro kawo madirom dakika 45-60.

*E kinde nonro duto*

Nyathini niro dhi nyime gi yudo thieth a klinik kak pile. Weche ma ochok e limbe gi mag klinik ma pile ibiro kaw kowuok e andike gi mag thieth (eg. Tarik ma orwake, chal ngimane koriwo nyaka kwan kute, tarik ma ochake e ART).

Ka klinik pok opimo kiwango mar kute mag ayaki e dend nyathini kaka dwarore gi chike mag piny Kenya , wabiro kwaye mondo omiwa remo moko ne pim mar kiwango mar kute man e dende , ma biro dwaro madirom mL5 (madirom ojiko achiel mar chae) mag remo e dweche 6 ka dweche 6. Ka nyathini ni e klinik e thuolo no, remo ibiro kawo e yo mapile e kar thieth. Ka ok obi e klinik , wabiro manyo nyathini kendo kwaye mondo ochiw remo mar pim ne ng’at ma nyalo golo remo oko mar kar thieth . Dwoko mag kwan kute biro yudore e klinik.

Bende wabiro ngíyo andike mag nyathini mag thieth mondo wane weche kuom thieth mare kaka limbe klinik , mondo wang’e ka bende onego wamanye . Wabiro tiyo gi weche ma ochok e andike mag nyathini mag theith mondo wang’e kaka gigi chacho nyalo mare mar biro e thieth .

Ka po ni waketo lokruok moko e nonro , in kod nyathini ibiro nyis u. Kata ka nyathini nigi kwan ma malo mar kute klinik biro hoye mondo omi kwan kute go odog piny mogik .

**Rach**

Wachano mar ngíyo kiwango mar kuet e dend nyathini nyadichiel e nonro ni ka klinik pok otimo mano. Nitiere rach mar rem kod lit sama ikawo remo. Nitiere thuolo matin mogik mar gamo tuo e kinde ma ikaow remo . Ne weche mathoth kuom rach kod rach ma thenge penj achiel kuom laktar man e klinik.

Nyalore ni ng’ato nyalo neno ote machwok ma waoro ne nyathini . Ote machwok ok bi bedo luongo kute mag ayaki kamoro amora kendo kata ka ngáto oyudo ote go ok gibing’eyo gimoro amora kuom chal mari mar kute mag ayaki. Kata kamano, mae nyalo kelo nu wichkuot , kendo dwaher ni mondo ingé ni rach ni nitie kapok iyie ne nyathini mondo obed e nonro .

Nitie bender ach ni moko kuom penjo ma wapenjo nyathini kuom kaka owinjo kod gika ma oseneno e thieth nyalo miyo obed ka ok en thuolo . Nyathini ni thuolo mar weyo dwoko penjo moro amora ma ok doher dwoko kata wuok e saa asaya .

Rach machielo en ni jomoko nyalo ng’eyo ni nyathini ni e nonro ni kata neno weche ge mag thieth ma iye kata weche kuom ngimane , kaka chal mare mar kute mag ayaki . Wabiro timo kar nyalowa mondo wane ni ma ok otimore. Jotij nonro kod mbese ma hoyo ji gin jok ma otieg e yo ma kende mondo girit weche nyathini ma nyalo fwenye kendo rito weche gi mondo jomamoko kik ng’e.

**Ber**

Nyalore ni nyathini ok bi yudo ber moro amora mochimore kode tir kuom bedo e nonro ni . Nyathini nyalo yudo ote machwok mag sime kod mbese ma hocho e yor sime, chiwo pesa , kata ja hocho ma mbas ma nene wang’gi wang’ . Yore mag kony gi nyalo konye medo bedo e thieth kendo nikech mano nyalo konyo ngimane . E giko nonro , dwoko go biro konyowa ng’eyo yo maber mogik kata riwruok mar yore ma inyalo ti godo e konyo ji mondo omed bedo e thieth.

**Dwoko pesa**

**Nyathini ibiro dwok ne pes wuoth mar silings ma Kenya 500/= e thuolo ma orwake e nonro.**

**Ka nyathini oket e yor kony mar chiwo pesa ka itimo gima idwaro obiro yudo silings ma Kenya 500/= ne biro e limbe klinik mochan eyi thuolo mar ndalo adek kendo en kod kwan mar kute man piny mogik nyaka 2 e higa achiel.**

**Yiero mamoko**

Nyathini ni thuolo mar yiero mondo kik obed e nonro . Ka oyiero mondo kik obed e nonro ni , onge kum ma ibiro miye . Ok obi wito ber ma oyudo ga , kendo podi onyalo yudo thieth e klinik mare kaka oja yudoga .

**Maling’ling’**

Wabiro timo ka nyalowa mondo wane ni weche gi manyalo fwenyi ma ochok e nonro ni oket mopondo . Kata kamano, ok wanyal singo maling’ling’ma chutho. Weche nyathini ma nyalo fwenye inyalo chiw ka dwarore e chik. Ka weche ma ochok e nonro ni ogo chapa kata osom e buche mag sayans , nying nyathini kod weche ge mamoko ma nyalo fwenye ok bi ti godo.

Jotij sirkal kata mag mbalariany seche moko ng’iyo ga nonro kaka ma mondo one ka bende itimo gi maber kendo kaluwore gi chik. Ka ngíyo nonro obedo , andike mag nyathini ibiro ng’i. Jok ma ng’iyo nonro go biro rito maling’ling mar nyathini . Andike mag nonro ok bi ti godo e keto nyathini e hinyruok e yor chik. Migepep ma nyalo ngíyo kata kopo andike mag nonro ne nonro, ng’iyo ka itimo nonro maber , kod ng’inyo weche nonro mochok oriwo:

- The Washington University, St. Louis
- The Kenya Medical Research Institute
- University of California San Francisco
- University of Colorado, Denver
- University of California, Berkeley

Bedo ni e nonro nyalo kelo wito maling’ling’, kata kamano wabiro timo keto chenro ma neno ni nyathini orit maling’ling’mare . Andike mar thieth ibiro los niekch bedo mari e nonro ni . Andike mar yie mar nyathini kod moko kuom dwoko mag pim mag nonro mag nyathini ibiro riw e andike ni . Nikech mano, jochwi thieth mag nyathini nyalo neno dwoko mage mag pim kendo ng’eyo ni in e nonro ni . Chike mag osibtal dwaro ni jochiw thieth duto keto weche man e andike mag thieth maling’ling’. Wach moro amora momedore kuom nonro ibiro chiw ne nyathini koriwo nyaka dwoko mogik mag nonro .

**Bedo e nonro**

Nyathini nyalo yiero mar weyo bedo e nonro e saa asaya maonge kum kata wito ber ma dine oyudo . Nyis anyisa jatim nonro kata jatij nonro sanosano ka nyathini dwaro weyo bedo e nonro . Nyathini nyalo wuok e nonro ka ooro ote ma oting’o “STOP” e namba nonro ma wabiro miyi. Bende, jatim nonro nyalo golo nyathini e nonro e saa asaya ka oneno ni en kuom ber mare , ka nyathini ok luw chike mag nonro , kata ka nonro ochungi.

**Jogo ma ochiwo pesa**

Nonro ni ochung’ne kod National Institutes of Health. Onge moro amora kom jotim nonro man malo kanyo man kod chachni moro amora motudore gi yore pesa ma miyo dwaro mar gi nyalo chachni gi nonro. E thuolo ma nonro nyalo bedo ni ochung kowuok kuom yiero mar jatim nonro kata jachiw pesa, ibiro nyisi gima biro timore tik mano.

**Yor tudruok**

Inyalo wuoyo kod jitm nonro kuom penjo, parruok, kata ywagruok moro amora ma in go kuom nonro ni. Tudri kod jatij wa mar nonro e 0733-819 835. Ka diher mar penjo penjo kuom nonro kata ratiro magi kaka jachiwre ne ng’ato machielo kopogore gi jotim nonro kata diher mar wacho chandruok kata parruok moro amora ma in go kuom nonro, inyalo tudri gi Jagoro mar Ethical Review Committee, Kenya Medical Research Institute e seru@kemri.org kata P.O. BOX 54840-00200 Sime. 020-2722541, 0717719477. Bende inyalo tudri kod Washington University Human Research Protection Office e +1-314-747-6800.Komiti gi odich gi rito jochiwre mag nonro.

**YIE**

Osemiyi kopi mar andike mar yie ni mondo ikan.

**BEDO E NONRO EN KUOM CHIWRUOK.**Nyathini nigi ratiro mar weyo bedo e nonro ni, kata wuok e saa asaya maonge kum kata wito ber ma dine oyudo .

**Wach ma oa kuom Jachiwre**

Nonro ni ma owuo kuome malo kanyo oselerna. Achiwora mar bedo e nonro ni. Aseyudo thuolo mar penjo penjo. Ka an gi penjo e kinde mabiro kuom nonro ni, anyalo penjo jotim nonro ma ondik malo kanyo. Ka an gi penjo kuom ratiro mag nyathin kaka jachiwre , anyalo tudra kod jok ma ondik malo kanyo.

Ayie ni nonro oti kod wech ma ochok mag nyathina ma onge nyinge kata namba ne mar klinik ne nonro ma kinde mabiro kendo ochiw ne jotim nonro mamoko maonge yie machielo ma achiwo.

Ka iyie mondo nyathini obed e nonro ni , onego iket seyi piny kanyo.

Tarik	Nying jachiwre

Tarik	Nying gi Seyi Janyuol/Jarit ne Yie

Tarik	Ng’at ma kawo Yie (Nying kod seyi )

***Ka ok onyal ndiko gi somo***

Aseneno somo mar andike mar yie ni e yo ma kare ne jachiwre kendo ng’atno osebedo gi thuolo mar penjo penjo. Asiro ni ng’atno ochiwo yie mare ka en thuolo**.**

Nying janeno _____________________ KOD

Seyi janeno __________________KOD

Nying jachiwre ___________________KOD

Alama lith Lwedo Mathuon mar jachiwre

Tarik ________________________

Kopi ne :	1. Jotim nonro

2. Jachiwre nonro

**OVERALL KISWAHILI ASSENT FORM**

**KENYA MEDICAL RESEARCH INSTITUTE**

**WASHINGTON UNIVERSITY, ST. LOUIS**

**ASSENT TO PARTICIPATE IN A RESEARCH STUDY**

**FOR PARTICIPANTS 14 - 17 YEARS OLD**

**Study Title:** *Adaptive Strategies to Prevent and Treat Failures of Retention in Adolescent HIV Care (A4A Study)*

WATAFITI NA TAASISI SHIRIKISHI:

| Mtafiti | Taasisi | Mawasiliano |
| --- | --- | --- |
| Dkt. Elizabeth Bukusi | Kenya Medical Research Institute | +254 733-617 503 |
| Dkt. Elvin Geng | Washington University, Missouri | +1-415-430-5589 (USA) |
| Dkt. Lisa Abuogi | University of Colorado, Denver | +1-303-724-7430 (USA) |
| Dkt. Zachary Kwena | Kenya Medical Research Institute | +254 722-386 714 |
| Starley Shade | University of California, San Francisco | +1-415 794-4354 (USA) |
| Lina Montoya | University of Notrh Carolina at Chapel Hill | +1-4105857470 (USA) |
| Eliud Akama | Kenya Medical Research Institute | +254 728-590 376 |

**Eneo la utafiti:** Kisumu County

Unaombwa ushiriki kwa utafiti. Sanduku iliyoko hapo chini inakueleza kuhusu mambo muhimu unayohitaji kufikira kabla ya kuamua kushiriki utafiti. Tutakupa habari zaidi chini ya sanduku hilo. Tafadhali uliza maswali kuhusu habari yoyote kabla ya kuamua ikiwa utashiriki au la. Pia ungependa kuzungumza na wengine (kwa mfano, familia yako, marafiki, au daktari wako) kuhusu utafiti, kabla ya kuamua kushiriki.

- **Idhini kwa hiari**. Unaombwa kujitolea kushiriki kwa utafiti. Ni juu yako ikiwa utaamua kushiriki au kutoshiriki. Hakuna adhabu kwako na hautapoteza chochote ikiwa utaamua kutoshiriki au baada ya kujiunga, ukaamua kujiondoa.
- **Lengo**. Tunafanya utafiti huu kujua mchanganyiko ipi ya mikakati inaleta matokeo bora zaidi ya kuhusisha vijana na wanarika kwa matibabu ya virusi vya ukimwi. Kusalia kwa matibabu na kuweka viwango vya virusi hadi sufuri ni muhimu zaidi kwa kuhakikisha kwamba vijana wanaoishi na virusi vya ukimwi waendelee kuwa wenye afya. Lakini, vijana na wanarika wanakumbana na vizuizi vingi dhidi ya kumeza dawa kulingana na maagizo na kusalia kwenye matibabu. Katika utafiti huu tunajaribu kujua jinsi tunavyoweza kuwapa vijana na wanarika mkakati inayowasaidia kusalia kwenye matibabu na kuwa na viwangi vya virusi vya sufuri.
- **Washiriki.** Takriban vijana na wanarika 880 wa umri kati ya miaka 14-24 wanaoishi na virusi vya ukimwi kwenye Kaunti ya Kisumu watakuwa kwa utafiti huu.
- **Muda.** Kushiriki kwako kwa utafiti utakuwa kwa muda wa miaka 2.
- **Taratibu na shughuli za utafiti.**

Katika huu utafiti, utakaguliwa kuona ikiwa unaweza kuingia utafiti na kwa njia ya nasibu utawekwa kwenye moja kati ya mikakati mbili za kwanza, njia ya matibabu ya kawaida au usaidizi kutoka kwa wanarika kwenye mambo ya kimatibabu kwa njia ya kielektroniki (arafa fupi zinazotumwa na kompyuta na usaidizi wa wanarika kupitia simu ya rununu). Ikiwa utakosa kufika kwenye tembezi la kliniki lililopangwa kwa zaidi ya siku 14 AU kuwa kiwango ya virusi yenye iko juu, utawekwa kwa njia ya nasibu kwenye njia ya matibabu ya kawaida, kutumiwa fedha ukitimiza masharti fulani (kupokea pesa), au mwanarika anayekusaidia kwa mambo ya matiababu anayekushughulikia mwenyewe .

Tutakuuliza maswali za kuchungua kuhusu hali yako ya kusihi, afya na ustawi wako wa kijamii, na unayoyajua kuhusu virusi vya ukimwi na upokezi wa matibabu jinsi inavyotakikana. Utafiti wa kuchungua utafanyaika wakati wa usajili, ikiwa umepewa (ikiwa umekosa tembezi la kliniki na siku zaidi ya 14 AU uko na idadi ya virusi iliyo juu), na baada ya kuwa kwa utafiti wa mwaka 1 na pia baada ya miaka 2. Tutakuuliza maswali kuhusu kuridhika kwako na yale umeyapitia na haya mikakati ya kuingilia kati yenye ulipokea ikiwa ulipewa upya, baada ya kuwa kwa utafiti kwa mwaka 1 na pia kwa miaka 2. Utafiti wa kuchungua utafanyika kwenye kituo cha afya lakini inaweza kufanywa katika eneo la kijamii ikiwa itahitajika. Utafiti wa kuchungua utachukua takriban dakika 45-60.

Tutachukua deta za kawaida za kliniki kutoka kwa rekodi zako za matibabu. Ikiwa viwango vya virusi havijachukuliwa kwenye kliniki wakati unafaa kufanywa, utafiti unawea kuwasiliana nawe na kuchukua damu ya kupima viwango vya virusi kwa takriban kila mwezi 6.

- **Hatari.**
- Uchungu au maumivu kutoka pahali walipochukulia damu.
- Kufichulika kiajali hali ya virusi vya ukimwi . Walakini, tutaweka mikakati ya usalama kuhakikisha kwamba hali yako ya virusi imelindwa.
- **Faida**. Kuna uwezekano hutapata faida yoyote kama wewe binafsi kwa kushiriki kwa utafiti huu. Unaweza kupokea arafa fupi za simu, kupewa pesa, au usaidizi wa mwanarika kwa mambo ya matibabu. Haya mikakati yanaweza kukusaidia uendelee kuwa kwa matibabu ya virus vya aukimwi na kumeza dawa zako vizuri na kwa sababu ya hayo kusaidia afya yako. Zaidi ya hayo, yale tutakayo jifunza kutoka kwa utafiti huu itatusaidia kuboresha matibabu ya virusi vya ukimwi kwa vijana kwa jumla.
- **Chaguzi mbadala.** Uko huru kuamua kutoshiriki kwa utafiti huu. Ukiamua kutoshiriki kwa utafiti huu, hakutakuwa na adhabu kwako. Hautapoteza faida zozote zako za kawaida, pia bado unaweza kupokea matibabu kutoka kwa kliniki yako jinsi umekuwa ukipokea.

**Taratibu na shughuli**

Kwanza, mzazi wako au mtunzi ataulizwa kutoa ruhusa ili uwe kwa utafiti huu. Iwapo mzazi au mtunzi hajaandamana nawe leo, tutawasiliana naye kwa njia ya rununu ili tupate idhini yake kabla ya kukuhusisha katika utafiti huu.Ikiwa mzazi wako aua mtunzi wako hajakubali , huwezi kuwa kwa utafiti . Ikiwa mzazi wako au mtunzi amekubali , na wewe pia umekubali , haya ndiyo yatakayofanyika baada ya haya:

**Siku ya Usajili**

Tutakuuliza maswali kuhusu jinsi unvayoishi, afya na ustawi wako wa kijamii, na unachojua kuhusu virusi vya ukimwi and kumeza dawa jinsi ulivyoagizwa. Utafiti wa kuchungua utachukua takriban dakika 45-60**.**

Utawekwa kwa moja kati ya mikakati miwili ya kuhusisha watu kwenye matibabu. Kuchaguliwa kwa mikakati ya kusaidia yenye utapokea itakuwa kwa nasibu, harakati ya kurusha shilingi na kuangalia inaanguka na upande gani. Mikakati za kuingilia kati ni:

- **“Matibabu ya kawaida”** yenye ni kwamba ukikosa kufika kwa tembezi la kliniki, taratibu za kliniki za kawaida za kukutafuta na kukumbusha uje kwa tembezi lako itafanyika. Au ukiwa na kiwango cha virusi cha juu kliniki itakushauri ili uweke viwango vya virusi view sufuri.
- **Kusaidiwa ka njia ya kielektroniki,** ambacho ni usaidizi wa wanarika kupitia simu za rununu na arafa fupi za simu zinazotumwa na kompyuta. Arafa fupi za simu yatumwa kwako mara kwa mara inazingatia kukuza afya. Msaidizi wa kiafya atakusaidia, kupitia habari na ushauri kuendelea na matibabu. Mwingiliano na msaidizi wa kiafya itakuwa kupitia kutuma arafa za simu au kuzungumza na yeye. Haya mawasiliano yanaweza kuwa kwa aplikesheni unayopenda, kwa mfano kupitia arafa za simu za kawaida, What’s App, Snapchat, au Facebook Messenger. Mawasiliano yatakuwa takriban kila wiki kwa muda wa miezi miwili ya kwanza na baadaye kila mwezi, na uwezo wa kuongea Zaidi au chini ikihitajika.

Njia ya Matibabu ya kawaida au kusaidiwa kwa njia ya kieletroniki yatakuwa kwa muda wa mwaka moja ikiwa hujakosa tembezi lako la kliniki na zaidi ya siku 14 AU uko na kiwango cha virusi kilicho juu.

Tutaendelea kukufuatilia katika utafiti huu kwa muda wa miaka miwili, kufuatilia kisajiliwa kwa utafiti.

*Ukikosa tembezi la kliniki kwa zaidia ya siku 14 AU uko na kiwango cha virusi ≥ 1000 kopi/ml:*

Ukikosa tembezi lako la kliniki na zaidi ya siku 14 AU uwe na kiwango cha juu cha virusi, bila kuangalia mikakati gani ulipokea kwanza, tutakutafuta unakoishi . Ikiwa tumefaulu kuwasiliana na wewe, tutakuuliza maswali ya kuchungua tena, maswali kuhusu kutosheleka kwako na yale uliyoyapitia na mikakati ya kuingilia kati ulizopokea, sababu za kutorudi, na ikiwa umehamia kituo kingine cha afya. Hii itachukua takriban dakika 45. Utafiti wa kuchugua huu utafanyika kwenye kituo cha afya au kwa jamii. Ikiwa hatutaweza kukupata, tutajaribu kuzungumza na yule ambaye ulipeana tuwasiliane naye kujua uliko.

Ikiwa hatutaweza kuwasiliana na wewe , tutakuweka tena kwa njia ya nasibu kwa moja kati ya mikakati ya kuingilia kati kukusaidia kurusi kwenye matibabu:

- **“Matibabu ya kawaida”** hii ni kwamba utpokea habari kuhusu umuhimu wa kuendelea na matibabu na kukuhimiza kurudi kwa klinik. Kama hatuwezi kuwasiliana na wewe kibinafsi, kliniki itajaribu kuwasiliana na wewe kwa njia ya simu au tembezi la nyumbani jinsi inavyofanywa kwa kawaida. Au kama uko na kiwango cha juu cha virusi kliniki watakushauri ili urudishe kiwango cha virusi hadi sufuri.
- **Kupewa pesa ukitimiza masharti.** Ikiwa umewekwa kwa kupewa pesa ukitimiza masharti, mfanyikazi wa utafiti atakupea shilingi ya Kenya 500/= kwako kwa kuhudhuria tembezi lako la kliniki iliyopangwa katika muda chini ya siku 3 na 500/= ingine kwa kuwa na kiwango cha virusi hadi kutoonekana 2 kwa mwaka. Unaweza kuchagua kupokea pesa kwa njia ya mpesa au pesa taslimu.
- **Msaidizi mwanarika wa kibinafsi.** Ikiwa umewekwa kwa usaidizi wa kimatibabu ya ana kwa ana, utapewa mwanarika anayekusaidia ana kwa ana akilinganishwa na jinsia na kukaribiana kwa rika. Huyu akuwa mtu kutoka kwa kliniki ambaye atataka kukusaidia, kupitia habari, ushauri na kukusaidia kwa jamii, ili uendelee kuwa kwa matibabu na kuwa na kiwango cha virusi cha sufuri. Huyu mtu atawasiliana na wewe ana kwa ana na ikiwezekana kwa simu. Huyu mtu atakutana na wewe kwanza nyumbani mwako (au pahala pengine yenye umependelea) na baadaye kukutana na wewe takriban kila mwezi katika pahala ilipopendelea. Mikutano yanaweza kuwa chache au mara mingi kuliko kila mwezi, lakini si zaidi ya kila wiki. Ukiendelea kuwa na changamoto zinazoendelea kama kukosa matembezi au kiwach cha virusi cha juu, huu mtu ataendelea kufanya na wewe kukusaidia uyashinde haya changamoto.

Mikakati ya matibabu ya kawaida, kupokea pesa kwa kutimiza masharti, au usaidizi wa ana kwa ana yataendelea kwa muda wa mwaka moja baada ya kuwekwa kwenye mkakati mpya.

Tutaendelea kukufuatilia kwenye utafiti huu kwa muda wa miaka miwili, kufuatia kusajiliwa kwa utafiti.

*Mwaka moja na miaka miwili baada ya kusajiliwa kwa utafiti*

Tutakuuliza maswali ya utafiti wa kuchungua kuhusu jinsi unavyoishi, afya and ustawi wako wa kijamiii, na unayojua kuhusu virusi vya ikumwi na kumeza dawa jinsi umeagizwa, na kutosheleka na yale umekumbana nayo na mikakati za kuingilia kati zenye unaweza kuwa umepata kwa utafiti kwa mwaka moja na miaka miwili baada ya usajili kwa utafiti . Utafiti huu wa kuchungua utachukua muda wa dakika 45-60.

*Wakati wote wa utafiti* Utaendelea kupokea matibabu yako kwenye kliniki kama kawaida. Deta kutoka kwa tembezi zako wa kliniki yatachukuliwa kutoka kwa rekodi zako za kimatibabu (k.m. tarehe ya usajili, hali ya afya ikijumuisha kiwango cha virusi, na tarehe ya kuazishwa ART).

Ikiwa kilinik yako haijapima kiwango cha virusi vya ukimwi kwenye mwili yako kulingana na miongozo ya kitaifa ya Kenya, tutauliza upeane damu kidogo ya kupima kiwango cha virusi vya ukimwi mwilini mwako, inayohitaji takriban mL5 (takriban kijiko kimoja cha chai) ya damu kwa karibu kila mwezi 6. Ukiwa kwenye kliniki wakati huo, damu itachukuliwa kwa njia ya kawaida kwenye kituo cha afya. Ikiwa huji kwenye kliniki, tutakutafuta na kuuliza upeane kipimo kwa mtu ambaye anaweza kutoa damu nje ya kituo cha afya. Matokeo ya kipimo cha kiwango cha virusi itapatikana kwako katika kliniki.

Tutaangalia rekodi zako za kliniki kuthibitisha habari kuhusu matibabu yako kama tembezi zako za kliniki, ili kujua ikiwa tunahitaji kukutafuta. Tutatumia habari zenye zimerekodiwa kwa rekodi zako za kliniki kuelewa jinsi haya mambo yanaathiri uwezo wako wa kuendelea kuja kwa matibabu.

Kama tumefanya mabadiliko yoyote kwenye utafiti, utaambiwa. Pia, ikiwa habari mpya imepatikana ikiwa uko kwa utafiti yenye unahitajika kujua, tutakuambia.

**Hatari**

Tunapanga kuangalia kiwango cha virusi vya ukimwi kwenye mwili wako mara moja ikiwa kliniki haijafanya hivo. Kuna hatari ya uchungu au maumivu kutokana na kutoa damu. Kuna hatari kidogo zaidi ya kupata maambukizi kutokana na kutoa damu. Kwa habari zaidi kuhusu hatari na madhara, uliza mmoja wa madaktari kwenye kliniki.

Kuna uwezekano kuwa mtu anaweza kuona arafa fupi tunazokutumia. Arafa fupi hayatakuwa na kutaja kwa vyovyote virusi vya ukimwi na hata mtu kipata simu yako hawatajua chochote juu ya hali yako ya virusi vya ukimwi. Walakini, hii inaweza kuiabisha, na tungependa ujulishwe kuhusu hii hatari kabla ya kuamua kujiunga na utafiti.

Kuna pia uwezekano kuwa baadhi ya maswali tutakuuliza kuhusu hisia zako na yale ambayo umekumbana nayo kwa matibabu yanaweza kukufanya kuhisi kama hauko huru. Uko huru kukataa kujibu swali yoyote ambacho hungependa kujibu au kuacha kushiriki wakati wowote.

Hatari nyingine is kwamba wengine wanweza kugundua kuwa unashiriki kwa utafiti huu au kuona habari zako za kimatibabu au za kiafya za kibinafsi, kama hali yako ya virus ivy aukimwi. Tutafanya kila tuwezalo kuhakikisha kwamba haya hayafanyiki. Wafanyikazi wa utafiti na wanarika wanaosaidia na mambo ya matibabu wamefundishwa kwa njia ya kipekee kuweka habari zako binafsi na kulinda habari zako ili watu wengine wasijue kukuhusu.

**Faida**

Kuna uwezekano kuwa hutapokea faida za kibinafsi kutokana na utafiti huu. Unaweza kupokea arafa fupi za simu na wanarika wanaokusiadia kwa mambo ya matibabu kupitia simu ya rununu, kupeana pesa, au kuwa na msaidizi kwa mambo ya matibabu mnaonana ana kwa ana. Haya mikakati ya kuingilia kati yanaweza kukusaidia kuendelea na matibabu na kwa sababu ya hiyo inaweza kusaidia afya yako. Katika mwisho wa utafiti, matokeo yatatusaidia kujua mikakati bora zaidi au mkusanyiko wa mikakati ya kutumia kusaidia watu waendelea kuwa kwa matibabu.

**Kurudisha pesa**

**Utarudishiwa pesa ya usafiri ya shlingi ya Kenya 500/= pindi tu unaposajiliwa kwa utafiti.**

**Ikiwa umewekwa kwa mikakati ya kuingilia kati ya kupeana pesa ukitimiza masharti utapokea shilingi ya Kenya 500/= kwa kuja kwa matembezi ya kliniki iliyopangwa ndani ya siku tatu na kuwa na kiwango cha virusi iliyothibitiwa hadi mara 2 kwa mwaka.**

**Chaguo mbadala**

Uko huru kuamua kutoshiriki kwa utafiti. Ukiamua kutoshiriki kwa utafiti huu, hakutakuwa na adhabu kwako. Hautapoteza faida zako zozote za kawaida, na badi pia unaweza kuokea matibabu yako jinsi umekua ukipokea kwenye kliniki.

**Usiri**

:

- The Washington University, St. Louis
- The Kenya Medical Research Institute
- University of California San Francisco
- University of Colorado, Denver
- University of California, Berkeley

Kushiriki kwa utafiti unaweza kuwa na kupoteza ubinafsi, walakini tutaweka mikakati ya usalama kulinda usiri wako. Rekodi ya kimatibabu itatengenezwa kwa sababu ya kushiriki kwako kwa utafiti huu. Fomu yako ya idhini na baadhi ya matokeo zako za utafiti zitajumuishwa kwa rekodi hii. Kwa hiyo, watao huduma wako wa kiafya wengine wanaweza kuona matokeo ya vipimo na kujua kuhusu kushiriki kwako kwa utafiti. Sheria za hospitali zinahitaji wahudumu wote wa afya kutunza habari zote kwenye rekodi zako za kimatibabu kwa usiri. Habari zozote za ziada kuhusu utafiti huu utapeanwa kwako ikijumuisha matokeo ya mwisho wa utafiti. **Kushiriki**

Unaweza kuamua kujiondoa kwa utafiti wakati wowote bila adhabu au kupoteza faida ambazo ungepata. Ambia tu mtafiti au mfanyikazi wa utafiti papo hapo ukiwa ungependa kujiondoa kwa utafiti. Unaweza kuacha kuwa kwa utafiti kwa kutuma neno “STOP” kwa nambari ya utafiti tutakachokupa. Pia, mtafiti anaweza kukuondoa kwa utafiti wakati wowote ikiwa ameamini kuwa na kwa maslahi yako bora, ikiwa hutafuata sheria a utafiti, au ikiwa utafiti umekomeshwa.

**Ufadhili**

Utafiti huu umefadhiliwa na National Institutes of Health. Hakuna yeyote kwa watafiti waliyo na mgongano wa maslahi ya kifedha. Katika hali yenye utafiti umekomeshwa kutokana na maamuzi ya mtafiti au mfadhili wa utafiti, utaelezwa kuhusu hatua ya kuchukiliwa baada ya hiyo.

**Mawasiliano**

Unaweza kuzungumza na m(wa)tafiti kuhusu maswali, wasiwasi, au malalamishi yoyote uliyonayo kuhusu utafiti huu. Wasiliana na mfanyikazi wetu wa utafiti katika 0733-819 835. Ikiwa ungependa kuuliza maswali kuhusu uatfiti au haki zako kama mshiriki wa utafiti kwa mtu mwingine kando ya watafiti au ukiwa ungependa kuzungumzia shida au wasiwasi ambazo uanweza kuwa nazo kuhusu utafiti, unaweza kuwasiliana na Katibu wa Ethical Review Committee, Kenya Medical Research Institute kwa seru@kemri.org au P.O. BOX 54840-00200 Tel. 020-2722541, 0717719477. Pa unaweza kuwasiliana na Washington University Human Research Protection Office kwa +1-314-747-6800.Haya kamati yanshughulika na kulinda haki za wanaojitolea kwa miradi za utafiti.

**IDHINI**

Umepewa nakala ya fomu hii ya idhini ili uweke.

**KUSHIRIKI KWA UTAFITI NI KWA HIARI.** Uko na haki ya kukataa kushiriki utafiti huu, au kujiondoa wakati wowote bila adhabu yoyote au kupoteza faida ambazo ungepata.

**Taarifa ya mshiriki**

Utafiti huu unaelezwa hapo juu nimeshaelezwa kuihusu. Najitolea kushiriki kwa utafiti huu. Nimekuwa na nafasi ya kuuliza maswali. Nikiwana maswali za siku zijazo kuhusu utafiti huu, ninaweza kuuliza watafiti waliondikwa hapo juu. Nikiwa na maswali kuhusu haki zangu kama mshiriki wa utafiti, ninaweza kuwasiliana na waliandikwa hapo juu.

Nimekubali habari zangu yatumike kwa minajili ya utafiti za siku zijazo na kupeanwa watafiti wengine bila ya kuongezea idhini na/bila vitambulishi.

Ikiwa ungependa kishiriki kwa utafiti huu, unafaa kuweka sahihi hapo chini.

Tarehe	Jina na Sahihi la Mshiriki

Tarehe	Mtu Anachukua Idhini (Jina na sahihi )

***Ikiwa hawezi kusoma na kuandika***

Nimeshuhudia kusomewa kwa njia sawa hii fomu ya idhini kwa mshiriki na mtu mwenyewe amekuwa na nafasi ya kuuliza maswali. Nathibitisha kuwa mtu mwenyewe amepeana idhini yaki akiwa huru**.**

Jina la shahidi _____________________ NA

Sahihi ya shahidi ***______________________***

Jina la mshiriki __________________ NA

Alama ya kidole gumba ya mshiriki

Tarehe ________________________

Nakala kwa:	1. Watafiti

3. Mshiriki wa utafiti

**KISWAHILI OVERALL INFORMED CONSENT FORM**

**KENYA MEDICAL RESEARCH INSTITUTE**

**WASHINGTON UNIVERSITY, ST. LOUIS**

**IDHINI YA KISHIRIKI UTAFITI**

**Study Title:** *Adaptive Strategies to Prevent and Treat Failures of Retention in Adolescent HIV Care (A4A Study)*

WATAFITI NA TAASISI SHIRIKISHI:

| Mtafiti | Taasisi | Mawasiliano |
| --- | --- | --- |
| Dkt. Elizabeth Bukusi | Kenya Medical Research Institute | +254 733-617 503 |
| Dkt. Elvin Geng | Washington University, Missouri | +1-415-430-5589 (USA) |
| Dkt. Lisa Abuogi | University of Colorado, Denver | +1-303-724-7430 (USA) |
| Dkt. Zachary Kwena | Kenya Medical Research Institute | +254 722-386 714 |
| Starley Shade | University of California, San Francisco | +1-415 794-4354 (USA) |
| Lina Montoya | University of Notrh Carolina at Chapel Hill | +1-4105857470 (USA) |
| Eliud Akama | Kenya Medical Research Institute | +254 728-590 376 |

**Eneo la utafiti:** Kisumu County

Unaombwa ushiriki kwa utafiti. Sanduku iliyoko hapo chini inakueleza kuhusu mambo muhimu unayohitaji kufikira kabla ya kuamua kushiriki utafiti. Tutakupa habari zaidi chini ya sanduku hilo. Tafadhali uliza maswali kuhusu habari yoyote kabla ya kuamua ikiwa utashiriki au la. Pia ungependa kuzungumza na wengine (kwa mfano, familia yako, marafiki, au daktari wako) kuhusu utafiti, kabla ya kuamua kushiriki.

- **Idhini kwa hiari**. Unaombwa kujitolea kushiriki kwa utafiti. Ni juu yako ikiwa utaamua kushiriki au kutoshiriki. Hakuna adhabu kwako na hautapoteza chochote ikiwa utaamua kutoshiriki au baada ya kujiunga, ukaamua kujiondoa.
- **Lengo**. Tunafanya utafiti huu kujua mchanganyiko ipi ya mikakati inaleta matokeo bora zaidi ya kuhusisha vijana na wanarika kwa matibabu ya virusi vya ukimwi. Kusalia kwa matibabu na kuweka viwango vya virusi hadi sufuri ni muhimu zaidi kwa kuhakikisha kwamba vijana wanaoishi na virusi vya ukimwi waendelee kuwa wenye afya. Lakini, vijana na wanarika wanakumbana na vizuizi vingi dhidi ya kumeza dawa kulingana na maagizo na kusalia kwenye matibabu. Katika utafiti huu tunajaribu kujua jinsi tunavyoweza kuwapa vijana na wanarika mkakati inayowasaidia kusalia kwenye matibabu na kuwa na viwangi vya virusi vya sufuri.
- **Washiriki.** Takriban vijana na vijana waliyobaleghe 880 wa umri kati ya miaka 14-24 wanaoishi na virusi vya ukimwi kwenye Kaunti ya Kisumu watakuwa kwa utafiti huu.
- **Muda.** Kushiriki kwako kwa utafiti utakuwa kwa muda wa miaka 2.
- **Taratibu na shughuli za utafiti.**

Katika huu utafiti, utakaguliwa kuona ikiwa unaweza kuingia utafiti na kwa njia ya nasibu utawekwa kwenye moja kati ya mikakati mbili za kwanza, njia ya matibabu ya kawaida au usaidizi kutoka kwa wanarika kwenye mambo ya kimatibabu kwa njia ya kielektroniki (arafa fupi zinazotumwa na kompyuta na usaidizi wa wanarika kupitia simu ya rununu). Ikiwa utakosa kufika kwenye tembezi la kliniki lililopangwa kwa zaidi ya siku 14 AU kuwa kiwango ya virusi yenye iko juu, utawekwa kwa njia ya nasibu kwenye njia ya matibabu ya kawaida, kutumiwa fedha ukitimiza masharti fulani (kupokea pesa), au mwanarika anayekusaidia kwa mambo ya matibabu anayekushughulikia mwenyewe .

Tutakuuliza maswali za kuchungua kuhusu hali yako ya kusihi, afya na ustawi wako wa kijamii, na unayoyajua kuhusu virusi vya ukimwi na upokezi wa matibabu jinsi inavyotakikana. Utafiti wa kuchungua utafanyaika wakati wa usajili, ikiwa umepewa (ikiwa umekosa tembezi la kliniki na siku zaidi ya 14 AU uko na idadi ya virusi iliyo juu), na baada ya kuwa kwa utafiti wa mwaka 1 na pia baada ya miaka 2. Tutakuuliza maswali kuhusu kuridhika kwako na yale umeyapitia na haya mikakati ya kuingilia kati yenye ulipokea ikiwa ulipewa upya, baada ya kuwa kwa utafiti kwa mwaka 1 na pia kwa miaka 2. Utafiti wa kuchungua utafanyika kwenye kituo cha afya lakini inaweza kufanywa katika eneo la kijamii ikiwa itahitajika. Utafiti wa kuchungua utachukua takriban dakika 45-60.

Tutachukua deta za kawaida za kliniki kutoka kwa rekodi zako za matibabu. Ikiwa viwango vya virusi havijachukuliwa kwenye kliniki wakati unafaa kufanywa, utafiti unawea kuwasiliana nawe na kuchukua damu ya kupima viwango vya virusi kwa takriban kila mwezi 6.

- **Hatari.**
- Uchungu au maumivu kutoka pahali walipochukulia damu.
- Kufichulika kiajali hali ya virusi vya ukimwi. Walakini, tutaweka mikakati ya usalama kuhakikisha kwamba hali yako ya virusi imelindwa.
- **Faida**. Kuna uwezekano hutapata faida yoyote kama wewe binafsi kwa kushiriki kwa utafiti huu. Unaweza kupokea arafa fupi za simu, kupewa pesa, au usaidizi wa mwanarika kwa mambo ya matibabu. Haya mikakati yanaweza kukusaidia uendelee kuwa kwa matibabu ya virus vya ukimwi na kumeza dawa zako vizuri na kwa sababu ya hayo kusaidia afya yako. Zaidi ya hayo, yale tutakayo jifunza kutoka kwa utafiti huu itatusaidia kuboresha matibabu ya virusi vya ukimwi kwa vijana kwa jumla.
- **Chaguzi mbadala.** Uko huru kuamua kutoshiriki kwa utafiti huu. Ukiamua kutoshiriki kwa utafiti huu, hakutakuwa na adhabu kwako. Hautapoteza faida zozote zako za kawaida, pia bado unaweza kupokea matibabu kutoka kwa kliniki yako jinsi umekuwa ukipokea.

**Taratibu na shughuli**

**Ikiwa umekubali kushiriki kwa utafiti huu, taratibu yafuatayo yatafanyika:**

**Siku ya Usajili**

Tutakuuliza maswali kuhusu jinsi unvayoishi, afya na ustawi wako wa kijamii, na unachojua kuhusu virusi vya ukimwi and kumeza dawa jinsi ulivyoagizwa. Utafiti wa kuchungua utachukua takriban dakika 45-60**.**

Utawekwa kwa moja kati ya mikakati miwili ya kuhusisha watu kwenye matibabu. Kuchaguliwa kwa mikakati ya kusaidia yenye utapokea itakuwa kwa nasibu, harakati ya kurusha shilingi na kuangalia inaanguka na upande gani. Mikakati za kuingilia kati ni:

- **“Matibabu ya kawaida”** yenye ni kwamba ukikosa kufika kwa tembezi la kliniki, taratibu za kliniki za kawaida za kukutafuta na kukumbusha uje kwa tembezi lako itafanyika. Au ukiwa na kiwango cha virusi cha juu kliniki itakushauri ili uweke viwango vya virusi view sufuri.
- **Kusaidiwa ka njia ya kielektroniki,** ambacho ni usaidizi wa wanarika kupitia simu za rununu na arafa fupi za simu zinazotumwa na kompyuta. Arafa fupi za simu yatumwa kwako mara kwa mara ikizingatia kukuza afya. Msaidizi wa kiafya atakusaidia, kupitia habari na ushauri kuendelea na matibabu. Mwingiliano na msaidizi wa kiafya itakuwa kupitia kutuma arafa za simu au kuzungumza na yeye. Haya mawasiliano yanaweza kuwa kwa aplikesheni unayopenda, kwa mfano kupitia arafa za simu za kawaida, What’s App, Snapchat, au Facebook Messenger. Mawasiliano yatakuwa takriban kila wiki kwa muda wa miezi miwili ya kwanza na baadaye kila mwezi, na uwezo wa kuongea zaidi au chini ikihitajika.

Njia ya Matibabu ya kawaida au kusaidiwa kwa njia ya kieletroniki yatakuwa kwa muda wa mwaka moja ikiwa hujakosa tembezi lako la kliniki na zaidi ya siku 14 AU uko na kiwango cha virusi kilicho juu.

Tutaendelea kukufuatilia katika utafiti huu kwa muda wa miaka miwili, kufuatilia kisajiliwa kwa utafiti.

*Ukikosa tembezi la kliniki kwa zaidia ya siku 14 AU uko na kiwango cha virusi ≥ 1000 kopi/ml:*

Ukikosa tembezi lako la kliniki na zaidi ya siku 14 AU uwe na kiwango cha juu cha virusi, bila kuangalia mikakati gani ulipokea kwanza, tutakutafuta unakoishi . Ikiwa tumefaulu kuwasiliana na wewe, tutakuuliza maswali ya kuchungua tena, maswali kuhusu kutosheleka kwako na yale uliyoyapitia na mikakati ya kuingilia kati ulizopokea, sababu za kutorudi, na ikiwa umehamia kituo kingine cha afya. Hii itachukua takriban dakika 45-60. Utafiti wa kuchugua huu utafanyika kwenye kituo cha afya au kwa jamii. Ikiwa hatutaweza kukupata, tutajaribu kuzungumza na yule ambaye ulipeana tuwasiliane naye kujua uliko.

Ikiwa hatutaweza kuwasiliana na wewe, tutakuweka tena kwa njia ya nasibu kwa moja kati tatu ya mikakati ya kuingilia kati kukusaidia kurusi kwenye matibabu:

- **“Matibabu ya kawaida”** hii ni kwamba utpokea habari kuhusu umuhimu wa kuendelea na matibabu na kukuhimiza kurudi kwa klinik. Kama hatuwezi kuwasiliana na wewe kibinafsi, kliniki itajaribu kuwasiliana na wewe kwa njia ya simu au tembezi la nyumbani jinsi inavyofanywa kwa kawaida. Au kama uko na kiwango cha juu cha virusi kliniki watakushauri ili urudishe kiwango cha virusi hadi sufuri.
- **Kupewa pesa ukitimiza masharti.** Ikiwa umewekwa kwa kupewa pesa ukitimiza masharti, mfanyikazi wa utafiti atakupea shilingi ya Kenya 500/= kwako kwa kuhudhuria tembezi lako la kliniki iliyopangwa katika muda chini ya siku 3 na 500/= ingine kwa kuwa na kiwango cha virusi hadi kutoonekana 2 kwa mwaka. Unaweza kuchagua kupokea pesa kwa njia ya mpesa au pesa taslimu.
- **Msaidizi mwanarika wa kibinafsi.** Ikiwa umewekwa kwa usaidizi wa kimatibabu ya ana kwa ana, utapewa mwanarika anayekusaidia ana kwa ana akilinganishwa na jinsia na kukaribiana kwa rika. Huyu akuwa mtu kutoka kwa kliniki ambaye atataka kukusaidia, kupitia habari, ushauri na kukusaidia kwa jamii, ili uendelee kuwa kwa matibabu na kuwa na kiwango cha virusi cha sufuri. Huyu mtu atawasiliana na wewe ana kwa ana na ikiwezekana kwa simu. Huyu mtu atakutana na wewe kwanza nyumbani mwako (au pahala pengine yenye umependelea) na baadaye kukutana na wewe takriban kila mwezi katika pahala ilipopendelea. Mikutano yanaweza mara mingi au chache kuliko kila mwezi, lakini si zaidi ya kila wiki. Ukiendelea kuwa na changamoto zinazoendelea kama kukosa matembezi au kiwach cha virusi cha juu, huu mtu ataendelea kufanya na wewe kukusaidia uyashinde haya changamoto.

Mikakati ya matibabu ya kawaida, kupokea pesa kwa kutimiza masharti, au usaidizi wa ana kwa ana yataendelea kwa muda wa mwaka moja baada ya kuwekwa kwenye mkakati mpya.

Tutaendelea kukufuatilia kwenye utafiti huu kwa muda wa miaka miwili, kufuatia kusajiliwa kwa utafiti.

*Mwaka moja na miaka miwili baada ya kusajiliwa kwa utafiti*

Tutakuuliza maswali ya utafiti wa kuchungua kuhusu jinsi unavyoishi, afya and ustawi wako wa kijamiii, na unayojua kuhusu virusi vya ikumwi na kumeza dawa jinsi umeagizwa, na kutosheleka na yale umekumbana nayo na mikakati za kuingilia kati zenye unaweza kuwa umepata kwa utafiti kwa mwaka moja na miaka miwili baada ya usajili kwa utafiti . Utafiti huu wa kuchungua utachukua muda wa dakika 45.

*Wakati wote wa utafiti*

Utaendelea kupokea matibabu yako kwenye kliniki kama kawaida. Deta kutoka kwa tembezi zako wa kliniki yatachukuliwa kutoka kwa rekodi zako za kimatibabu (k.m. tarehe ya usajili, hali ya afya ikijumuisha kiwango cha virusi, na tarehe ya kuazishwa ART).

Ikiwa kilinik yako haijapima kiwango cha virusi vya ukimwi kwenye mwili yako kulingana na miongozo ya kitaifa ya Kenya, tutauliza upeane damu kidogo ya kupima kiwango cha virusi vya ukimwi mwilini mwako, inayohitaji takriban mL5 (takriban kijiko kimoja cha chai) ya damu kwa karibu kila mwezi 6. Ukiwa kwenye kliniki wakati huo, damu itachukuliwa kwa njia ya kawaida kwenye kituo cha afya. Ikiwa huji kwenye kliniki, tutakutafuta na kuuliza upeane kipimo kwa mtu ambaye anaweza kutoa damu nje ya kituo cha afya. Matokeo ya kipimo cha kiwango cha virusi itapatikana kwako katika kliniki.

Tutaangalia rekodi zako za kliniki kuthibitisha habari kuhusu matibabu yako kama tembezi zako za kliniki, ili kujua ikiwa tunahitaji kukutafuta. Tutatumia habari zenye zimerekodiwa kwa rekodi zako za kliniki kuelewa jinsi haya mambo yanaathiri uwezo wako wa kuendelea kuja kwa matibabu.

Kama tumefanya mabadiliko yoyote kwenye utafiti, utaambiwa. Pia, ikiwa habari mpya imepatikana ikiwa uko kwa utafiti yenye unahitajika kujua, tutakuambia.

**Hatari** Tunapanga kuangalia kiwango cha virusi vya ukimwi kwenye mwili wako mara moja ikiwa kliniki haijafanya hivo. Kuna hatari ya uchungu au maumivu kutokana na kutoa damu. Kuna hatari kidogo zaidi ya kupata maambukizi kutokana na kutoa damu. Kwa habari zaidi kuhusu hatari na madhara, uliza mmoja wa madaktari kwenye kliniki.

Kuna uwezekano kuwa mtu anaweza kuona arafa fupi tunazokutumia. Arafa fupi hayatakuwa na kutaja kwa vyovyote virusi vya ukimwi na hata mtu kipata simu yako hawatajua chochote juu ya hali yako ya virusi vya ukimwi. Walakini, hii inaweza kuiabisha, na tungependa ujulishwe kuhusu hii hatari kabla ya kuamua kujiunga na utafiti.

Kuna pia uwezekano kuwa baadhi ya maswali tutakuuliza kuhusu hisia zako na yale ambayo umekumbana nayo kwa matibabu yanaweza kukufanya kuhisi kama hauko huru. Uko huru kukataa kujibu swali yoyote ambacho hungependa kujibu au kuacha kushiriki wakati wowote.

Hatari nyingine is kwamba wengine wanweza kugundua kuwa unashiriki kwa utafiti huu au kuona habari zako za kimatibabu au za kiafya za kibinafsi, kama hali yako ya virus ivy aukimwi. Tutafanya kila tuwezalo kuhakikisha kwamba haya hayafanyiki. Wafanyikazi wa utafiti na wanarika wanaosaidia na mambo ya matibabu wamefundishwa kwa njia ya kipekee kuweka habari zako binafsi na kulinda habari zako ili watu wengine wasijue kukuhusu.

**Faida**Kuna uwezekano kuwa hutapokea faida za kibinafsi kutokana na utafiti huu. Unaweza kupokea arafa fupi za simu na wanarika wanaokusiadia kwa mambo ya matibabu kupitia simu ya rununu, kupeana pesa, au kuwa na msaidizi kwa mambo ya matibabu mnaonana ana kwa ana. Haya mikakati ya kuingilia kati yanaweza kukusaidia kuendelea na matibabu na kwa sababu ya hiyo inaweza kusaidia afya yako. Katika mwisho wa utafiti, matokeo yatatusaidia kujua mikakati bora zaidi au mkusanyiko wa mikakati ya kutumia kusaidia watu waendelea kuwa kwa matibabu.

**Kurudisha pesa**

**Utarudishiwa pesa ya usafiri ya shlingi ya Kenya 500/= pindi tu unaposajiliwa kwa utafiti. Ikiwa umewekwa kwa mikakati ya kuingilia kati ya kupeana pesa ukitimiza masharti utapokea shilingi ya Kenya 500/= kwa kuja kwa matembezi ya kliniki iliyopangwa ndani ya siku tatu na kuwa na kiwango cha virusi iliyothibitiwa hadi mara 2 kwa mwaka.**

**Chaguo mbadala**Uko huru kuamua kutoshiriki kwa utafiti. Ukiamua kutoshiriki kwa utafiti huu, hakutakuwa na adhabu kwako. Hautapoteza faida zako zozote za kawaida, na badi pia unaweza kuokea matibabu yako jinsi umekua ukipokea kwenye kliniki.

**Usiri**Tutafanya kadri ya uwezo wetu kuhakikisha habari za kibinfasi yalikusanywa kwa minajili ya utafiti imewekwa siri. Walakini, hatuwezi kuhakikisha usiri kamilifu. Habari zako za kibinfasi yanaweza kupeanwa ikihitajika kisheria. Ikiwa habari kutoka kwa utafiti huu umechapishwa au kusomwa kwenye mikutano ya sayansi, jina lako au habari zako zingine za kibinafsi hazitatumika.

Wafanyikazi wa serikali na vyuo vikuu wakati mwingine hungalia utafiti kama huu kuhakikisha yanafanywa kwa usalama na kulingana na sheria . Ikiwa utafiti huu umeangaliwa, rekodi zako zitachunguzwa. Wachunguzi hao watalinda ubinafsi wako. Rekodi za utafiti hazitatumika kukuweka kwa hatari ya kisheria. Mashirika yanaweza kuangalia na/au kunakili rekodi zako za utafiti kwa ajili ya utafiti, utenda kazi, na uchambuzi wa deta yanajumuisha:

- The Washington University, St. Louis
- The Kenya Medical Research Institute
- University of California San Francisco
- University of Colorado, Denver
- University of California, Berkeley

Kushiriki kwa utafiti unaweza kuwa na kupoteza ubinafsi, walakini tutaweka mikakati ya usalama kulinda usiri wako. Rekodi ya kimatibabu itatengenezwa kwa sababu ya kushiriki kwako kwa utafiti huu. Fomu yako ya idhini na baadhi ya matokeo zako za utafiti zitajumuishwa kwa rekodi hii. Kwa hiyo, watao huduma wako wa kiafya wengine wanaweza kuona matokeo ya vipimo na kujua kuhusu kushiriki kwako kwa utafiti. Sheria za hospitali zinahitaji wahudumu wote wa afya kutunza habari zote kwenye rekodi zako za kimatibabu kwa usiri. Habari zozote za ziada kuhusu utafiti huu utapeanwa kwako ikijumuisha matokeo ya mwisho wa utafiti.

**Kushiriki**

Unaweza kuamua kujiondoa kwa utafiti wakati wowote bila adhabu au kupoteza faida ambazo ungepata. Ambia tu mtafiti au mfanyikazi wa utafiti papo hapo ukiwa ungependa kujiondoa kwa utafiti. Unaweza kuacha kuwa kwa utafiti kwa kutuma neno “STOP” kwa nambari ya utafiti tutakachokupa. Pia, mtafiti anaweza kukuondoa kwa utafiti wakati wowote ikiwa ameamini kuwa na kwa maslahi yako bora, ikiwa hutafuata sheria a utafiti, au ikiwa utafiti umekomeshwa.

**Ufadhili**

Utafiti huu umefadhiliwa na National Institutes of Health. Hakuna yeyote kwa watafiti waliyo na mgongano wa maslahi ya kifedha. Katika hali yenye utafiti umekomeshwa kutokana na maamuzi ya mtafiti au mfadhili wa utafiti, utaelezwa kuhusu hatua ya kuchukiliwa baada ya hiyo.

**Mawasiliano**

Unaweza kuzungumza na m(wa)tafiti kuhusu maswali, wasiwasi, au malalamishi yoyote uliyonayo kuhusu utafiti huu. Wasiliana na mfanyikazi wetu wa utafiti katika 0733-819 835. Ikiwa ungependa kuuliza maswali kuhusu uatfiti au haki zako kama mshiriki wa utafiti kwa mtu mwingine kando ya watafiti au ukiwa ungependa kuzungumzia shida au wasiwasi ambazo uanweza kuwa nazo kuhusu utafiti, unaweza kuwasiliana na Katibu wa Ethical Review Committee, Kenya Medical Research Institute kwa seru@kemri.org au P.O. BOX 54840-00200 Tel. 020-2722541, 0717719477. Pa unaweza kuwasiliana na Washington University Human Research Protection Office kwa +1-314-747-6800.Haya kamati yanshughulika na kulinda haki za wanaojitolea kwa miradi za utafiti

**IDHINI**

Umepewa nakala ya fomu hii ya idhini ili uweke.

**KUSHIRIKI KWA UTAFITI NI KWA HIARI.** Uko na haki ya kukataa kushiriki utafiti huu, au kujiondoa wakati wowote bila adhabu yoyote au kupoteza faida ambazo ungepata.

**Taarifa ya mshiriki**

Utafiti huu unaelezwa hapo juu nimeshaelezwa kuihusu. Najitolea kushiriki kwa utafiti huu. Nimekuwa na nafasi ya kuuliza maswali. Nikiwana maswali za siku zijazo kuhusu utafiti huu, ninaweza kuuliza watafiti waliondikwa hapo juu. Nikiwa na maswali kuhusu haki zangu kama mshiriki wa utafiti, ninaweza kuwasiliana na waliandikwa hapo juu.

Nimekubali habari zangu yatumike kwa minajili ya utafiti za siku zijazo na kupeanwa watafiti wengine bila ya kuongezea idhini na/bila vitambulishi.

Ikiwa ungependa kishiriki kwa utafiti huu, unafaa kuweka sahihi hapo chini.

Tarehe	Jina na Sahihi la Mshiriki

Tarehe	Mtu Anachukua Idhini (Jina na sahihi )

***Ikiwa hawezi kusoma na kuandika***

Nimeshuhudia kusomewa kwa njia sawa hii fomu ya idhini kwa mshiriki na mtu mwenyewe amekuwa na nafasi ya kuuliza maswali. Nathibitisha kuwa mtu mwenyewe amepeana idhini yaki akiwa huru**.**

Jina la shahidi _____________________ NA

Sahihi ya shahidi ***______________________***

Jina la mshiriki __________________ NA

Alama ya kidole gumba ya mshiriki

Tarehe ________________________

Nakili kwa:	1. Watafiti

2. Mshiriki wa utafiti

**KISWAHILI OVERALL PARENTAL CONSENT FORM**

**KENYA MEDICAL RESEARCH INSTITUTE**

**WASHINGTON UNIVERSITY, ST. LOUIS**

**PARENTS/GUARDIAN PERMISSION FORM**

**KWA WASHIRIKI WA UTAFITI WA UMRI WA MIAKA14 – 17**

**Study Title:** *Adaptive Strategies to Prevent and Treat Failures of Retention in Adolescent HIV Care (A4A Study)*

WATAFITI NA TAASISI SHIRIKISHI:

| Mtafiti | Taasisi | Mawasiliano |
| --- | --- | --- |
| Dkt. Elizabeth Bukusi | Kenya Medical Research Institute | +254 733-617 503 |
| Dkt. Elvin Geng | Washington University, Missouri | +1-415-430-5589 (USA) |
| Dkt. Lisa Abuogi | University of Colorado, Denver | +1-303-724-7430 (USA) |
| Dkt. Zachary Kwena | Kenya Medical Research Institute | +254 722-386 714 |
| Starley Shade | University of California, San Francisco | +1-415 794-4354 (USA) |
| Lina Montoya | University of Notrh Carolina at Chapel Hill | +1-4105857470 (USA) |
| Eliud Akama | Kenya Medical Research Institute | +254 728-590 376 |

**Eneo la utafiti :** Kisumu County

Tunaomba mtoto wako ashiriki kwa utafiti . Sanduku iliyoko hapo chini unakueleza vitu muhimu ambazo unafaa kufikiria kuhusu kabla ya kupeana mtoto wako ruhusa ya kujiunga na utafiti . Tutapeana habari zaidi chini ya sanduku hilo. Tafadhali uliza maswali kuhusu habari yoyote kabla ya kupeana rushusa. Pia ungependa kuzungumza na wengine (kwa mfano, familia yako, marafiki, au daktari) kuhusu utafiti huu, kabla ya kupeana idhini.

- **Idhini kwa hiari**. Unaombwa kuruhusu mtoto wako kushiriki kwa utafiti. Mtoto wako hataadhibiwa kwa kutoshiriki kwa utafiti au kujiondoa kwa utafiti wakati wowote.
- **Lengo**. Tunafanya utafiti huu kujua mchanganyiko ipi ya mikakati inaleta matokeo bora zaidi ya kuhusisha vijana na wanarika kwa matibabu ya virusi vya ukimwi. Kusalia kwa matibabu na kuweka viwango vya virusi hadi sufuri ni muhimu zaidi kwa kuhakikisha kwamba vijana wanaoishi na virusi vya ukimwi waendelee kuwa wenye afya. Lakini, vijana na wanarika wanakumbana na vizuizi vingi dhidi ya kumeza dawa kulingana na maagizo na kusalia kwenye matibabu. Katika utafiti huu tunajaribu kujua jinsi tunavyoweza kuwapa vijana na wanarika mkakati inayowasaidia kusalia kwenye matibabu na kuwa na viwangi vya virusi vya sufuri.
- **Washiriki.** Takriban vijana na vijana waliyobaleghe 880 wa umri kati ya miaka 14-24 wanaoishi na virusi vya ukimwi kwenye Kaunti ya Kisumu watakuwa kwa utafiti huu.
- **Muda.** Kushiriki kwako kwa utafiti utakuwa kwa muda wa miaka 2.
- **Taratibu na shughuli za utafiti.**

Katika utafiti huu , mtoto wako atakaguliwa na kuwekwa kwa njia ya nasibu kwa moja kati ya mikakati ya kuingilia kati mbili za kwanza , njia ya matibabu ya kawaida au usaidizi kutoka kwa wanarika kwenye mambo ya kimatibabu kwa njia ya kielektroniki (arafa fupi zinazotumwa na kompyuta na usaidizi wa wanarika kupitia simu ya rununu). Ikiwa mtoto wako atakosa tembezi lake la kliniki iliyopangwa na zaidi ya siku 14 AU ako na kiwango cha virusi iliyo juu ,atawekwa tena kwa njia ya nasibu kwa njia ya matibabu ya kawaida, kutumiwa fedha ukitimiza masharti fulani (kupokea pesa), au mwanarika anayekusaidia kwa mambo ya matiababu anayekushughulikia mwenyewe .

Mtoto wako ataombwa maswali ya utafiti wa kuchungua kuhusu jinsi anavyoishi , afya na ustawi wa kijamii , na kile anajua kuhusu virusi vya ukimwi na kumeza dawa jinsi mtu ameagizwa. Utafiti wa kuchungua utafanyika wakati wa usajili , na ikiwa amewekwa kwingine (ikiwa amekosa tembezi la kliniki na zaidi ya siku 14 AU ako na kiwango cha virusi cha juu) na baada ya kuwa kwa utafiti kwa muda wa mwaka 1 na tena baada ya miaka 2. Tutauliza mtoto wako maswali kuhusu kutosheleka kwake na yale amekumbana nayo kwa mikakati ya kuingilia kati yenye anaweza kuwa amepata , akiwa alipewa upya , baada ya kuwa kwa utafiti kwa mwaka 1 na tena kwa miaka 2 . Utafiti wa kuchungua ytafanywa kwenye kituo cha afya lakini inweza kufanywa kwa eneo la kijamii ikiwa inahitajika. Utafiti wa kuchungua utachukua takriban dakika 45-60.

Pia tutakusanya deta ya kliniki ya kawaida kutoka kwa rekodi zake za matibabu . Ikiwa kiwango cha virusi haijachukuliwa kwa kliniki jinsi inavykuwa ikifanywa wakati wa kufanywa ikiwa imefika , utafiti unaweza kuwasiliana naye na kuchukua damu ya kupima kiwango cha virusi kila miezi 6 .

- **Hatari.**
- Uchungu au maumivu kutoka pahali walipochukulia damu.
- Kufichulika kiajali hali ya virusi vya ukimwi. Walakini, tutaweka mikakati ya usalama kuhakikisha kwamba hali yako ya virusi imelindwa.
- **Faida**. Mtoto wako kuna uwezekano hatapokea faida kibinafsi kwa kushiriki kwa utafiti huu. Mtoto wako anaweza kupokea arafa fupi za simu Mtoto wako anaweza kupokea arafa fupi za simu na kusaidiwa na mwanarika kwa njia ya simu ya rununu, kupewa pesa, au usaidizi wa mwanarika kwa mambo ya matibabu . Haya mikakati ya kuingilia kati yanaweza kusaidia mtoto wako kusalia kwa matibabu nakumeza madawa vizuri na kwa hiyo inaweza kusaidia afya yake . Zaidi ya hayo , yale tutakayo jifunza kutoka kwa utafiti huu itatusaidia kuboresha matibabu ya virusi vya ukimwi kwa vijana kwa jumla.
- **Chaguzi mbadala.** Uko huru kuamua kutoruhusu mtoto wako kushiriki kwa utafiti . Ikiwa umeamua kutoruhusu mtoto wako, hakutakuwa na adhabu yoyote kwa mtoto wako. Mtoto wako hatapoteza faida zozote za kawaida , na anaweza bado kupokea matibabu yako kutoka kwa klinik yao jinsi wamekuwa wakifanya .

**Taratibu na shughuli**

**Ukikubali mtoto wako ashiriki kwa utafiti huu, na pia yeye amekubali , taratibu zifuatazo zitafanyika:**

**Siku ya Usajili**

Tutauliza mtoto wako maswali kuhusu jinsi anavyoishi , afya na ustawi wake wa kijamii , na anayojua kuhusu virusi vya ukimwi na kumweza dawa jinsi inavyotakikana. Utafiti wa kuchungua utachukua takriban dakika 45-60**.**

Atawekwa kwa moja kati ya mikakati miwili ya kuhusisha watu kwenye matibabu. Kuchaguliwa kwa mikakati ya kusaidia yenye utapokea itakuwa kwa nasibu, harakati ya kurusha shilingi na kuangalia inaanguka na upande gani. Mikakati za kuingilia kati ni:

- **“Matibabu ya kawaida”** yenye ni kwamba akikosa kufika kwa tembezi la kliniki, taratibu za kliniki za kawaida za kumtafuta na kumkumbusha aje kwa tembezi lake itafanyika. Au akiwa na kiwango cha virusi cha juu kliniki watamshauri ili aweke viwango vya virusi viwe sufuri.
- **Kusaidiwa ka njia ya kielektroniki,** ambacho ni usaidizi wa wanarika kupitia simu za rununu na arafa fupi za simu zinazotumwa na kompyuta. Arafa fupi za simu yatatumwa kwa mtoto wako mara kwa mara ikizingatia kukuza afya. Msaidizi wa kiafya atamsaidia, kupitia habari na ushauri kuendelea na matibabu. Mwingiliano na msaidizi wa kiafya itakuwa kupitia kutuma arafa za simu au kuzungumza na yeye. Haya mawasiliano yanaweza kuwa kwa aplikesheni anayopenda, kwa mfano kupitia arafa za simu za kawaida, What’s App, Snapchat, au Facebook Messenger. Mawasiliano yatakuwa takriban kila wiki kwa muda wa miezi miwili ya kwanza na baadaye kila mwezi, na uwezo wa kuongea zaidi ikihitajika.

Njia ya matibabu ya kawaida au kusaidiwa kwa njia ya kieletroniki yatakuwa kwa muda wa mwaka moja ikiwa hajakosa tembezi lake la kliniki na zaidi ya siku 14 AU ako na kiwango cha virusi kilicho juu.

Tutaendelea kumfuatilia mtoto wako katika utafiti huu kwa muda wa miaka miwili, kufuatilia kisajiliwa kwa utafiti.

*Ikiwa mtoto wako amekosa tembezi la kliniki na zaidi ya muda wa siku 14 OR ama na kiwango cha virusi ≥ nakala 1000 /ml:*

Ikiwa mtoto wako amekosa kuja kwa tembezi lake la kliniki kwa siku zaidi ya 14 AU ako na kiwango cha virusi cha juu , haijalishi ako kwenye mikakati gani ya kuingilia kati aliyepokea mara ya kwanza , tutamtafuta kwa eneo la kijamii . Ikiwa tutaweza kuwasiliana na mtoto wako tutamuuliza maswali ya kuchangua tena, maswali kuhusu kuridhika kwake na yale ambayo amekumbana nayo kuhusiana na mikakati ya kuingilia kati yenye alipokea ,sababu zulizomfanya hakurudi , na ikiwa amehamia kituo kingine cha afya . Utafiti huu wa kuchungua utachukua takriban dakika 45-60. Ikiwa hatuwezi kupata mtoto wako , Tutajaribu kuzungumza na alipeana kuungumza naye kujua aliko .

Ikiwa tutaweza kuwasiliana na mtoto wako, tutamweka tena kwa njia ya nasibu kwa moja kati tatu ya mikakati ya kuingilia kati kumsaidia kurudi kwenye matibabu	:

- **“Matibabu ya kawaida”** hii ni kwamba mtoto wako atapokea habari kuhusu umuhimu wa kuendelea na matibabu na kumhimiza kurudi kwa klinik. Kama hatuwezi kuwasiliana na mtoto wako kibinafsi, kliniki itajaribu kuwasiliana na yeye kwa njia ya simu au tembezi la nyumbani jinsi inavyofanywa kwa kawaida. Au mtoto wako ako na kiwango cha juu cha virusi kliniki watamshauri ili arudishe kiwango chake cha virusi hadi sufuri.
- **Kupewa pesa ukitimiza masharti.** Ikiwa mtoto wako amewekwa kwa kupeanwa pesa akitimiza masharti , mfanyikazi wa utafiti atapeana shilingi ya Kenya 500 kwa mtoto wako kwa kuhudhuria tembezi lake la kliniki lililopangwa ndani ya siku 3 na shilingi 500/= ingine kwa kuwa na virusi vilivyothibitiwa hadi mara 2 kwa mwaka. Mtoto wako anaweza kuamua kupokea pesa kwa njia ya Mpesa au pesa taslimu.
- **Msaidizi mwanarika wa kibinafsi.** Ikiwa mtoto wako amewekwa kwa usaidizi wa kimatibabu ya ana kwa ana, atapewa mwanarika anayemsaidia ana kwa ana akilinganishwa na jinsia na kukaribiana kwa rika. Huyu atakuwa mtu kutoka kwa kliniki ambaye atataka kumsaidia, kupitia habari, ushauri na kumsaidia kwa jamii, ili aendelee kuwa kwa matibabu na kuwa na kiwango cha virusi cha sufuri. Huyu mtu atawasiliana na mtoto wako ana kwa ana na ikiwezekana kwa simu. Huyu mtu atakutana na mtoto wako kwanza nyumbani mwako (au pahala pengine yenye amependelea) na baadaye kukutana na mtoto wako takriban kila mwezi katika pahala alipopendelea. Mikutano yanaweza kuwa mara mingi au chache kuliko kila mwezi, lakini si zaidi ya kila wiki. Ikiwa mtoto wakio ameendelea kuwa na changamoto zinazoendelea kama kukosa matembezi au kiwango cha virusi cha juu, huu mtu ataendelea kufanya na yeye kumsaidia ayashinde haya changamoto.

Mikakati ya matibabu ya kawaida, kupokea pesa kwa kutimiza masharti, au usaidizi wa ana kwa ana yataendelea kwa muda wa mwaka moja baada ya kuwekwa kwenye mkakati mpya.

Tutaendelea kumfuatilia kwenye utafiti huu kwa muda wa miaka miwili, kufuatia kusajiliwa kwa utafiti.

*Mwaka moja na miaka miwili baada ya kusajiliwa kwa utafiti*

Tutauliza mtoto wako maswali ya utafiti wa kuchungua kuhusu jinsi anavyoishi, afya and ustawi wake wa kijamiii, na anayojua kuhusu virusi vya ikumwi na kumeza dawa jinsi ameagizwa, na kutosheleka na yale umekumbana nayo na mikakati za kuingilia kati zenye unaweza kuwa umepata kwa utafiti kwa mwaka moja na miaka miwili baada ya usajili kwa utafiti . Utafiti huu wa kuchungua utachukua muda wa dakika 45.

*Wakati wote wa utafiti*

Mtoto wako ataendelea kupokea matibabu yake kwenye kliniki kama kawaida. Deta kutoka kwa tembezi zake wa kliniki yatachukuliwa kutoka kwa rekodi zake za kimatibabu (k.m. tarehe ya usajili, hali ya afya ikijumuisha kiwango cha virusi, na tarehe ya kuazishwa ART).

Ikiwa kiliniki haijapima kiwango cha virusi vya ukimwi kwenye mwili ya mtoto wako kulingana na miongozo ya kitaifa ya Kenya, tutauliza apeane damu kidogo ya kupima kiwango cha virusi vya ukimwi mwilini mwake, inayohitaji takriban mL5 (takriban kijiko kimoja cha chai) ya damu kwa karibu kila mwezi 6. Akiwa kwenye kliniki wakati huo, damu itachukuliwa kwa njia ya kawaida kwenye kituo cha afya. Ikiwa haji kwenye kliniki, tutamtafuta na kuuliza aeeane kipimo kwa mtu ambaye anaweza kutoa damu nje ya kituo cha afya. Matokeo ya kipimo cha kiwango cha virusi itapatikana kwako katika kliniki.

Pia tutaangalia rekodi za mtoto wako za kliniki kuthibitisha habari kuhusu matibabu yako kama tembezi zako za kliniki, ili kujua ikiwa tunahitaji kumtafuta. Tutatumia habari zenye zimerekodiwa kwa rekodi za mtoto wako za kliniki kuelewa jinsi haya mambo yanaathiri uwezo wake wa kuendelea kuja kwa matibabu.

Kama tumefanya mabadiliko yoyote kwenye utafiti, wewe na mtoto wako mtaambiwa. Au ikiwa mtoto wako ako na kiwango cha virusi cha juu kliniki watamshauri kiwango cha virusi hadi sufuri.

**Hatari**

Tunapanga kuangalia kiwango cha virusi vya ukimwi kwenye mwili wa mtoto wako mara moja ikiwa kliniki haijafanya hivo. Kuna hatari ya uchungu au maumivu kutokana na kutoa damu. Kuna hatari kidogo zaidi ya kupata maambukizi kutokana na kutoa damu. Kwa habari zaidi kuhusu hatari na madhara, uliza mmoja wa madaktari kwenye kliniki.

Kuna uwezekano kuwa mtu mwingine anaweza kuona arafa fupi yenye tumetumia mtoto wako Arafa fupi hayatakuwa na kutajwa yoyote kwa virusi vya ukimwi na hata ikiwa mtu mwingine angeziona hizo arafa fupi hawatajua chochote kuhusu hali yako ya virusi vya ukimwi . Walakini, hii inaweza kuwa cha kuaibisha kwenu wawili, na tungependa ujue kuhusu hatari hiyo kabla ya kuamua kuruhusu mtoto wako kushiriki kwa utafiti .

Kuna pia uwezekano kuwa baadhi ya maswali tutauliza mtoto wako kuhusu hisia zake na yale ambayo amekumbana nayo kwa matibabu yanaweza kumfanya kuhisi kama hako huru. Mtoto wako ako huru kukataa kujibu swali yoyote ambacho hangependa kujibu au kuacha kushiriki wakati wowote.

Hatari nyingine is kwamba wengine wanaweza kugundua kuwa mtoto wako anashiriki kwa utafiti huu au kuona habari zake za kimatibabu au za kiafya za kibinafsi, kama hali yako ya virusi vya ukimwi. Tutafanya kila tuwezalo kuhakikisha kwamba haya hayafanyiki. Wafanyikazi wa utafiti na wanarika wanaosaidia na mambo ya matibabu wamefundishwa kwa njia ya kipekee kuweka habari za mtoto wako binafsi na kulinda habari zako ili watu wengine wasijue kukuhusu.

**Faida**

Kuna uwezekano kuwa mtoto wako hatapokea faida za kibinafsi kutokana na utafiti huu. Mtoto wako anaweza kupokea arafa fupi za simu na wanarika wanaomsiadia kwa mambo ya matibabu kupitia simu ya rununu, kupeana pesa, au kuwa na msaidizi kwa mambo ya matibabu mnaonana ana kwa ana. Haya mikakati ya kuingilia kati yanaweza kukusaidia kuendelea na matibabu na kwa sababu ya hiyo inaweza kusaidia afya yako. Katika mwisho wa utafiti, matokeo yatatusaidia kujua mikakati bora zaidi au mkusanyiko wa mikakati ya kutumia kusaidia watu waendelea kuwa kwa matibabu.

**Kurudishiwa pesa**

**Mtoto wako atapewa pesa usafiri ya shilingi ya Kenya 500/= pindi tu anaposajiliwa.**

**Ikiwa mtoto wako amewekwa kwa mikakati ya kuingilia kati ya kupeana pesa ukitimiza masharti atapokea shilingi ya Kenya 500/= kwa kuja kwa matembezi ya kliniki iliyopangwa ndani ya siku tatu na kuwa na kiwango cha virusi iliyothibitiwa hadi mara 2 kwa mwaka.**

**Chaguo mbadala**

Mtoto wako ako huru kuamua kutoshiriki kwa utafiti. Akiamua kutoshiriki kwa utafiti huu, hakutakuwa na adhabu kwake. Hatapoteza faida zake zozote za kawaida, na bado pia anaweza kupokea matibabu yake jinsi amekua akipokea kwenye kliniki

**Usiri**

Tutafanya kadri ya uwezo wetu kuhakikisha habari za kibinfasi yalikusanywa kwa minajili ya utafiti imewekwa siri. Walakini, hatuwezi kuhakikisha usiri kamilifu. Habari za mtoto wako za kibinfasi yanaweza kupeanwa ikihitajika kisheria. Ikiwa habari kutoka kwa utafiti huu umechapishwa au kusomwa kwenye mikutano ya sayansi, jina la mtoto wako au habari zake zingine za kibinafsi hazitatumika.

Wafanyikazi wa serikali na vyuo vikuu wakati mwingine hungalia utafiti kama huu kuhakikisha yanafanywa kwa usalama na kulingana na sheria . Ikiwa utafiti huu umeangaliwa, rekodi za mtoto wako zitachunguzwa. Wachunguzi hao watalinda ubinafsi wa mtoto wako. Rekodi za utafiti hazitatumika kuweka mtoto wako kwa hatari ya kisheria. Mashirika yanaweza kuangalia na/au kunakili rekodi zako za utafiti kwa ajili ya utafiti, utenda kazi, na uchambuzi wa deta yanajumuisha:

- The Washington University, St. Louis
- The Kenya Medical Research Institute
- University of California San Francisco
- University of Colorado, Denver
- University of California, Berkeley

Kushiriki kwa utafiti unaweza kuwa na kupoteza ubinafsi, walakini tutaweka mikakati ya usalama kulinda usiri wako. Rekodi ya kimatibabu itatengenezwa kwa sababu ya kushiriki kwako kwa utafiti huu. Fomu ya mtoto wako ya idhini na baadhi ya matokeo zake za utafiti zitajumuishwa kwa rekodi hii. Kwa hiyo, watao huduma wa mtoto wako wa kiafya wengine wanaweza kuona matokeo ya vipimo na kujua kuhusu kushiriki kwako kwa utafiti. Sheria za hospitali zinahitaji wahudumu wote wa afya kutunza habari zote kwenye rekodi zako za kimatibabu kwa usiri. Habari zozote za ziada kuhusu utafiti huu utapeanwa kwa mtoto wako ikijumuisha matokeo ya mwisho wa utafiti..

**Kushiriki**

Mtoto wako anaweza kuamua kujiondoa kwa utafiti wakati wowote bila adhabu au kupoteza faida ambazo angepata. Ambia tu mtafiti au mfanyikazi wa utafiti papo hapo ukiwa mtoto wako angependa kujiondoa kwa utafiti. Mtoto wako anaweza kuacha kuwa kwa utafiti kwa kutuma neno “STOP” kwa nambari ya utafiti tutakachokupa. Pia, mtafiti anaweza kuondoa mtoto wako kwa utafiti wakati wowote ikiwa ameamini kuwa na kwa maslahi yake bora, ikiwa hatafuata sheria a utafiti, au ikiwa utafiti umekomeshwa.

**Ufadhili**

Utafiti huu umefadhiliwa na National Institutes of Health. Hakuna yeyote kwa watafiti waliyo na mgongano wa maslahi ya kifedha. Katika hali yenye utafiti umekomeshwa kutokana na maamuzi ya mtafiti au mfadhili wa utafiti, utaelezwa kuhusu hatua ya kuchukiliwa baada ya hiyo.

**Mawasiliano**

Unaweza kuzungumza na m(wa)tafiti kuhusu maswali, wasiwasi, au malalamishi yoyote uliyonayo kuhusu utafiti huu. Wasiliana na mfanyikazi wetu wa utafiti katika 0733-819 835. Ikiwa ungependa kuuliza maswali kuhusu uatfiti au haki zako kama mshiriki wa utafiti kwa mtu mwingine kando ya watafiti au ukiwa ungependa kuzungumzia shida au wasiwasi ambazo uanweza kuwa nazo kuhusu utafiti, unaweza kuwasiliana na Katibu wa Ethical Review Committee, Kenya Medical Research Institute kwa seru@kemri.org au P.O. BOX 54840-00200 Tel. 020-2722541, 0717719477. Pa unaweza kuwasiliana na Washington University Human Research Protection Office kwa +1-314-747-6800.Haya kamati yanshughulika na kulinda haki za wanaojitolea kwa miradi za utafiti.

**IDHINI**Umepewa nakala ya fomu hii ya idhini ili uweke..

**KUSHIRIKI KWA UTAFITI NI KWA HIARI.** Mtoto ako na haki ya kukataa kuwa kwa uatfiti huu, au kujiondoa wakati wowote bila adhabu au kupoteza faida ambazo angepata .

**Taarifa ya mshiriki**

Utafiti huu unaelezwa hapo juu nimeshaelezwa kuihusu. Najitolea kuruhusu mtoto wangu kushiriki kwa utafiti huu. Nimekuwa na nafasi ya kuuliza maswali. Nikiwana maswali za siku zijazo kuhusu utafiti huu, ninaweza kuuliza watafiti waliondikwa hapo juu. Nikiwa na maswali kuhusu haki za mtoto wangu kama mshiriki wa utafiti, ninaweza kuwasiliana na waliandikwa hapo juu.

Nimekubali utafiti utumie habari za mtoto wangu yenye haina jina au kitambulishi ya kliniki kwa utafiti za siku zijazo na kupeanwa na watafiti wengine bila idhini ingine kutoka kwangu .

Ukiruhusu mtoto wako kushirki kwa utafiti huu, unafaa kuweka sahihi hapo chini.

Tarehe	Jina na Sahihi la Mshiriki

Tarehe	Jina na Sahihi ya Mzazi/Mtunzi ya Kutoa Idhini

Tarehe	Mtu Anachukua Idhini (Jina na sahihi )

***Ikiwa hawezi kusoma na kuandika***

Nimeshuhudia kusomewa kwa njia sawa hii fomu ya idhini kwa mshiriki na mtu mwenyewe amekuwa na nafasi ya kuuliza maswali. Nathibitisha kuwa mtu mwenyewe amepeana idhini yaki akiwa huru**.**

Jina la shahidi _____________________ NA

Sahihi la shahidi __________________NA

Jina la mshiriki ___________________AND

Alama ya kidole gumba ya mshiriki

Tarehe ________________________

Nakala kwa:	1. Watafiti

2. Mshiriki wa utafiti

**Study Title:** *Adaptive Strategies to Prevent and Treat Failures of Retention in Adolescent HIV Care (A4A Study)*

**KENYA MEDICAL RESEARCH INSTITUTE**

**WASHINGTON UNIVERSITY, ST LOUIS**

**DHOLUO A4A ASSENT TO PARTICIPATE IN AN INDEPTH INTERVIEW (IDI)*(14 - 17 YEARS OLD)***

JOTIM NONRO KOD MIGEPE MA GITUDORE GODO:

| Jatim Nonro | Migao | Yor Tudruok |
| --- | --- | --- |
| Laktar Elizabeth Bukusi | Kenya Medical Research Institute | +254 733-617 503 |
| Laktar Elvin Geng | Washington University, St. Louis | +1-415-430-5589 (USA) |
| Laktar Lisa Abuogi | University of Colorado, Denver | +1-303-724-7430 (USA) |
| Laktar Zachary Kwena | Kenya Medical Research Institute | +254 722-386 714 |
| Starley Shade | University of California, San Francisco | +1-415-794-4354 (USA) |
| Lina Montoya | University of Notrh Carolina at Chapel Hill | +1-4105857470 (USA) |
| Eliud Akama | Kenya Medical Research Institute | +254 728-590 376 |

**Kama itimo e nonro:** Kisumu County

Wadwaro nyisi kuom gimoro ma watimo ma iluongo ni nonro . Nonro en ka lakteche choko weche mathoth mondo ging’e mathoth kuom gimoro . Wakwayi mondo ichiwri mar bedo e twak mar kanyakla ma en bath nonro ma watimo. Boksi man piny kanyo no nyisi gik ber ma onego ipar kapok iyiero mar bedo a twak mar kanyakla ni . Wabiro chiwo wach momedore e boksi man piny kanyo. Yie ipenj penjo kuom wach moro amora kapok iyiero ka idonjo kata ok idonj. Bende inyalo hero mar wuoyo kod jomamoko (kuom ranyisi, jo anyuola ni, osiepe, kata jotim nonro ) kuom nonro ni, kapok iyie mar donjo..

- **Yie kuom chiwruok**. Ikwayi mondo ichiwri mar bedo e twak mar kanyakla . En kuomi mar yiero ka idwaro bedo kata ok idwar . Onge kum kendo ok ibi wito gimoro amora ka iyiero ni ok idonj kata bang’e ka isedonjo, inyalo yiero mar wuok.
- **Gima omiyo itime**. Watimo twak mondo wang’i gigo ma rowere kod jomadongo matindo neno gi chenro mag nonro ni. Dwaher mar ng’eyo pachu, mor u, kod gik ma useneno gi chenro mag kony ma uyudo. Pachi kod weche ma ichiwo biro konyowa e ng’eyo kaka chenro mar konyo no tiyo.
- **Jochiwre:** Madirom rowere kod jomadongo matindo 60, ma jo higni 14 – 24, ma orwak e nonro mar A4A e Kisumu county, biro bedo e twak.
- **Chenro kod gik ma itimo.** Ka iyie mar bedo, wabiro keto saa mar twak kodi . Twak ni ibiro tim kod ng’at ma tayo twak ma otiegi e kama opondo mondo orit maling’ling’mari. Ibiro penji penjo kuom mor mari kod gik ma iseneno kuom chenro mar kony mane iyudo, kony ma ichiwo gi mbese e yor kompyuta/sime (ote machwok mag sime kod hocho ma itimo gi mbese e yor simb ong’we yamo ), hocho mar mbese ma wang’ gi wang’, kata chiwo pesa ka itimo gima idwaro. Wabiro penji kuom achiel kende kuom yore mag konyo go. E wi mano, wabiro kwayi thuolo mar mako twak, ma waburo ndiko piny kendo ng’iyo bang’e. Wach moro amora ma nyalo fwenyi ok bi bedo e weche ma ing’iyo. Wageno ni twak ni biro bedo kuom madirom dakika 45-60.
- **Rach.** Nitiere thuolo matin ma fwenyruok ma ok ochan mar chal mar kute mag ayaki . Kata kamano, wabiro keto chenro ma rito wach kuom chal mari mar kute mag ayaki.
- **Ber**. Nyalore ni ok ibi yudo ber moro amora mochimoe kodi tir kuom bedo e nonro ni. Kuom bedo e nonro ni inyalo konyo e chenro mag keto maber ngima rowere ma odak gi kute mag ayaki.
- **Yiero mamoko.** In thuolo mar yiero mondo kik ibed e nonro . Ka iyiero ni ok ibed e nonro ni , onge kum ma ibiro miyi . Ok ibi wito ber moro amora ma iyudo ga , kendo podi inyalo yudo thieth kowuok e klinik mari kaka iyudo ga .

**Chenro kod gik ma itimo**

Ka dipo ni janyuol okobiro kodi e klinik, wabiro tudre kode e yor sime mondo wakwa thuolo mondo ibed e nonro

Ka iyie, gik moluwo gi biro timore:

1. Wabiro kwayi mondo iket seyi kata alama e andike ni kaka ranyisi ni iyie kuom chiwruok mar bedo e nonro ni.
2. Wabiro keto saa ma wabiro twak kodi.
3. Wabiro penjo penjo kuom pachi, mor kod gik ma iseneno kuom chenro ma ne iyudo**, ma biro bedo** konyo e yor kompyuta (ote machwok ma ioro e yor kompyuta kod kony ma ichiwo gi mbese e yor sime), kony ma ichiwo gi mbese ka gibiro iri giwegi, kata pes ama ichiwo ka itimo gima idwaro.
4. **Pachi kod weche ma ichiwo biro konyowa ngéyo kaka chenro mar konyo no tiyo.**
5. Twak no ibiro ta kod ng’at ma tayo twak ma otiegi e kama opondo mondo orit maling’ling’ mari.
6. Wabiro kwayi mondo iyienwa wamak twak kod dwoko ma imiyowa mondo wandik gi piny kendo wangí gi bang’e.
7. Wach moro amora ma nyalo fwenyi ok bi bedo e weche ma ing’iyo.
8. Twak biro bedo kuom madirom dakika 45-60.

**Rach**

Nitie thuolo matin mar ji ngéyo chal ni mar kute mag ayaki ka ok ochan. Kata kamano, wabiro keto chenro ma biro rito weche kuom chal mari mar kute mag ayaki.

**Ber**

Nyalore ni ok ibi yudo ber moro amora mochimore kodi tir kuom bedo e nonro ni. Kata kamano, bedo mari nyalo konyo keto maber bedo mar rowere e thieth.

**Dwoko pesa**

**Ibiro dwokni pesa wuoth mar silings ma Kenya 500/=.**

**Yiero mamoko**

In thuolo mar yiero mondo kik ibed e nonro. Ka iyiero mondo kik ibed e nonro ni, onge kum ma ibiro miyi. Ok ibi wito moro amora kuom ber ma iyudo ga pile, kendo podi inyalo yudo thieth mari kowuok e klinik mari kaka ijayudo ga.

**Maling’ling’**

Wabiro timo kar nyalowa mondo wane ni weche gi manyalo fwenyi ma ochok ne nonro ni oket mopondo. Kata kamano, ok wanyal singo maling’ling’ ma chutho. Weche gi manyalo fwenyi inyalo chiw ka idwaro e chik. Ka weche ma owuok e nonro ni ogo chapa kata osom e buche mag sayans, nyingi kod weche gi mamoko ma nyalo fwenyi ok bi ti godo.

Jotij sirkal kata mbalariany seche moko ng’iyo ga nonro kaka ma e mondo gine ni itimo gi e yo makare kendo kaluwore gi chik. Ka ng’iyo nonro ni otimore , andike gi ibiro ng’i. Jogo ma ng’iyo nonro gi biro rito maling’ling’mari. Andike mag nonro ok bi ti godo e kelo ni rach e yor chik. Migepe ma nyalo ng’iyo kendo/kata kopo andike gi mag nonro ne nonro, ngíyo kaka itimo tich, kod ng’inyo weche mochok oriwo:

- The Washington University, St Louis
- The Kenya Medical Research Institute
- University of California San Francisco
- University of Colorado, Denver
- University of California, Berkeley

Bedo e nonro nyalo bedo gi wito maling’ling’, kata kamano wabiro keto yore mag rito maling’ling’mari . Wach moro amora momedore kuom nonro ibiro miyi koriwo nyaka dwok mogik mag nonro.

**Bedo e nonro**

Inyalo yiero mondo iwuog e twak e saa asaya, nyis anyisa jatij nonro kendo gibiro goli. E wi mano, jotij nonro nyalo goli, ka gineno ni in kuom ber mari mogik. Ok bi miyi kum e yo moro amora ka di po ni iyiero mar wuok.

**Joma ochiwo pesa**

Nonro ni ochung’ne gi National Institutes of Health. Onge moro amora kom jotim nonro man malo kanyo man kod chachni moro amora motudore gi yore pesa ma miyo dwaro mar gi nyalo chachni gi nonro. E thuolo ma nonro nyalo bedo ni ochung kowuok kuom yiero mar jatim nonro kata jachiw pesa, ibiro nyisi gima biro timore tok mano.

**Yor tudruok**

Inyalo wuoyo kod jotim nonro kuom penjo, parruok, kata ywagruok moro amora ma in go kuom nonro ni. Tudri kod jatij wa mar nonro e 0733-819 835. Ka diher mar penjo penjo kuom nonro kata ratiro magi kaka jachiwre ne ng’ato machielo kopogore gi jotim nonro kata diher mar wacho chandruok kata parruok moro amora ma in go kuom nonro , inyalo tudri gi Jagoro mar Ethical Review Committee, Kenya Medical Research Institute e seru@kemri.org kata P.O. BOX 54840-00200 Sime. 020-2722541, 0717719477. Bende inyalo tudri kod Washington University Human Research Protection Office e +1-314-747-6800.Komiti gi odich gi rito jochiwre mag nonro.

**YIE**

Osemiyi kopi mar andike mar yie ni mondo ikan.

**BEDO E NONRO EN KUOM CHIWRUOK.** In gi ratiro mar weyo bedo e nonro ni, kata wuok e saa asaya ma onge kum moro amora kata wito ber ma dine iyudo.

**Wach ma oa kuom Jachiwre**

Nonro ni ma owuo kuome malo kanyo oselerna. Achiwora mar bedo e nonro ni. Aseyudo thuolo mar penjo penjo. Ka an gi penjo e kinde mabiro kuom nonro ni, anyalo penjo jotim nonro ma ondik malo kanyo. Ka an gi penjo kuom ratiro maga kaka jachiwre nonro, anyalo tudra kod jok ma ondik malo kanyo.

Ayie mondo weche maga ma ochok oti godo e nonro ma kinde mabiro kendo ochiw ne jotim nonro mamoko maonge yie machielo kod/maonge gigo manyalo fwenya . Ka diher mar bedo e nonro ni, onego iket seyi piny kanyo

Tarik	Nying kod Seyi Jachiwre ne Yie

Tarik	Ng’at ma Kawo Yie (Nying kod Seyi)

***Ka ok onyal somo kata ndiko***

Aseneno somo mar andike mar yie ni e yo ma kare ne jachiwre kendo ng’atno osebedo gi thuolo mar penjo penjo. Asiro ni ng’atno ochiwo yie mare ka en thuolo**.**

Nying janeno _____________________ KOD

Seyi janeno *__________________*KOD

Nying janeno __________________KOD

**Alama mar lith lwedo mathuon**

**Tarik ________________________**

**Thuolo mar mako twak**

Achiwo ne twak ma kanyakla ma abiro bedo e mondo omak **: Ee ☐	Ooyo ☐**

Kopi ne :	1. Jotim nonro

2. Jachiwre nonro

**Study Title:** *Adaptive Strategies to Prevent and Treat Failures of Retention in Adolescent HIV Care (A4A Study)*

**KENYA MEDICAL RESEARCH INSTITUTE**

**WASHINGTON UNIVERSITY, ST LOUIS**

**DHOLUO CONSENT TO PARTICIPATE IN AN INDEPTH INTERVEIW (IDI)*(18 - 24 YEARS OLD)***

JOTIM NONRO KOD MIGEPE MA GITUDORE GODO:

| Jatim Nonro | Migao | Yor Tudruok |
| --- | --- | --- |
| Laktar Elizabeth Bukusi | Kenya Medical Research Institute | +254 733-617 503 |
| Laktar Elvin Geng | Washington University, St. Louis | +1-415-430-5589 (USA) |
| Laktar Lisa Abuogi | University of Colorado, Denver | +1-303-724-7430 (USA) |
| Laktar Zachary Kwena | Kenya Medical Research Institute | +254 722-386 714 |
| Starley Shade | University of California, San Francisco | +1-415-794-4354 (USA) |
| Lina Montoya | University of Notrh Carolina at Chapel Hill | +1-4105857470 (USA) |
| Eliud Akama | Kenya Medical Research Institute | +254 728-590 376 |

**Kama itimo e nonro :** Kisumu County

Ikwayi mondo ichiwri mar bedo e twak . Boksi man piny kanyo nyisi gik ma onego ipar ahinya kapok iyiero mar bedo e twak ni . Yie ipenj penjo moro amora kuom weche ma ochiw kapok iyiero mar bedo kata weyo bedo e nonro. Bende inyalo dwaro wuoyo kod jomamoko (kuom ranyisi , jo anyuola ni , osiepe, kata jotim nonro) kuom nonro ni , kapok iyie mar donjo.

- **Yie kuom chiwruok**. Ikwayi mondo ichiwri mar bedo e twak mar kanyakla . En kuomi mar yiero ka idwaro bedo kata ok idwar . Onge kum kendo ok ibi wito gimoro amora ka iyiero ni ok idonj kata bang’e ka isedonjo, inyalo yiero mar wuok.
- **Gima omiyo itime**. Watimo twak mondo wang’i gigo ma rowere kod jomadongo matindo neno gi chenro mag nonro ni. Dwaher mar ng’eyo pachu, mor u, kod gik ma useneno gi chenro mag kony ma uyudo. Pachi kod weche ma ichiwo biro konyowa e ng’eyo kaka chenro mar konyo no tiyo.
- **Jochiwre:** Madirom rowere kod jomadongo matindo 60, ma jo higni 14 – 24, ma orwak e nonro mar A4A e Kisumu county, biro bedo e twak.
- **Chenro kod gik ma itimo.** Ka iyie mar bedo, wabiro keto saa mar twak kodi . Twak ni ibiro tim kod ng’at ma tayo twak ma otiegi e kama opondo mondo orit maling’ling’mari. Ibiro penji penjo kuom mor mari kod gik ma iseneno kuom chenro mar kony mane iyudo, kony ma ichiwo gi mbese e yor kompyuta/sime (ote machwok mag sime kod hocho ma itimo gi mbese e yor simb ong’we yamo ), hocho mar mbese ma wang’ gi wang’, kata chiwo pesa ka itimo gima idwaro. Wabiro penji kuom achiel kende kuom yore mag konyo go. E wi mano, wabiro kwayi thuolo mar mako twak, ma waburo ndiko piny kendo ng’iyo bang’e. Wach moro amora ma nyalo fwenyi ok bi bedo e weche ma ing’iyo. Wageno ni twak ni biro bedo kuom madirom dakika 45-60.
- **Rach.** Nitiere thuolo matin ma fwenyruok ma ok ochan mar chal mar kute mag ayaki . Kata kamano, wabiro keto chenro ma rito wach kuom chal mari mar kute mag ayaki.
- **Ber**. Nyalore ni ok ibi yudo ber moro amora mochimoe kodi tir kuom bedo e nonro ni. Kuom bedo e nonro ni inyalo konyo e chenro mag keto maber ngima rowere ma odak gi kute mag ayaki.
- **Yiero mamoko.** In thuolo mar yiero mondo kik ibed e nonro . Ka iyiero ni ok ibed e nonro ni , onge kum ma ibiro miyi . Ok ibi wito ber moro amora ma iyudo ga , kendo podi inyalo yudo thieth kowuok e klinik mari kaka iyudo ga.

**Chenro kod gik ma itimo**

Ka iyie, gik moluwo gi biro timore:

1. Wabiro kwayi mondo iket seyi kata alama e andike ni kaka ranyisi ni iyie kuom chiwruok mar bedo e nonro ni.
2. Wabiro keto saa ma wabiro twak kodi.
3. Wabiro penjo penjo kuom pachi, mor kod gik ma iseneno kuom chenro ma ne iyudo**, ma biro bedo** konyo e yor kompyuta (ote machwok ma ioro e yor kompyuta kod kony ma ichiwo gi mbese e yor sime), kony ma ichiwo gi mbese ka gibiro iri giwegi, kata pes ama ichiwo ka itimo gima idwaro.
4. **Pachi kod weche ma ichiwo biro konyowa ngéyo kaka chenro mar konyo no tiyo..**
5. Twak no ibiro ta kod ng’at ma tayo twak ma otiegi e kama opondo mondo orit maling’ling’ mari.
6. Wabiro kwayi mondo iyienwa wamak twak kod dwoko ma imiyowa mondo wandik gi piny kendo wangí gi bang’e.
7. Wach moro amora ma nyalo fwenyi ok bi bedo e weche ma ing’iyo.
8. Twak biro bedo kuom madirom dakika 45-60.

**Rach**

Nitie thuolo matin mar ji ngéyo chal ni mar kute mag ayaki ka ok ochan. Kata kamano, wabiro keto chenro ma biro rito weche kuom chal mari mar kute mag ayaki.

**Ber**

Nyalore ni ok ibi yudo ber moro amora mochimore kodi tir kuom bedo e nonro ni. Kata kamano, bedo mari nyalo konyo keto maber bedo mar rowere e thieth.

**Dwoko pesa**

**Ibiro dwokni pesa wuoth mar silings ma Kenya 500/=.**

**Yiero mamoko**

In thuolo mar yiero mondo kik ibed e nonro. Ka iyiero mondo kik ibed e nonro ni, onge kum ma ibiro miyi. Ok ibi wito moro amora kuom ber ma iyudo ga pile, kendo podi inyalo yudo thieth mari kowuok e klinik mari kaka ijayudo ga.

**Maling’ling’**

Wabiro timo kar nyalowa mondo wane ni weche gi manyalo fwenyi ma ochok ne nonro ni oket mopondo. Kata kamano, ok wanyal singo maling’ling’ ma chutho. Weche gi manyalo fwenyi inyalo chiw ka idwaro e chik. Ka weche ma owuok e nonro ni ogo chapa kata osom e buche mag sayans, nyingi kod weche gi mamoko ma nyalo fwenyi ok bi ti godo.

Jotij sirkal kata mbalariany seche moko ng’iyo ga nonro kaka ma e mondo gine ni itimo gi e yo makare kendo kaluwore gi chik. Ka ng’iyo nonro ni otimore , andike gi ibiro ng’i. Jogo ma ng’iyo nonro gi biro rito maling’ling’mari. Andike mag nonro ok bi ti godo e kelo ni rach e yor chik. Migepe ma nyalo ng’iyo kendo/kata kopo andike gi mag nonro ne nonro, ngíyo kaka itimo tich, kod ng’inyo weche mochok oriwo:

- The Washington University, St Louis
- The Kenya Medical Research Institute
- University of California San Francisco
- University of Colorado, Denver
- University of California, Berkeley

Bedo e nonro nyalo bedo gi wito maling’ling’, kata kamano wabiro keto yore mag rito maling’ling’mari . Wach moro amora momedore kuom nonro ibiro miyi koriwo nyaka dwok mogik mag nonro.

**Bedo e nonro**

Inyalo yiero mondo iwuog e twak e saa asaya, nyis anyisa jatij nonro kendo gibiro goli. E wi mano, jotij nonro nyalo goli, ka gineno ni in kuom ber mari mogik. Ok bi miyi kum e yo moro amora ka di po ni iyiero mar wuok.

**Joma ochiwo pesa**

Nonro ni ochung’ne gi National Institutes of Health. Onge moro amora kom jotim nonro man malo kanyo man kod chachni moro amora motudore gi yore pesa ma miyo dwaro mar gi nyalo chachni gi nonro. E thuolo ma nonro nyalo bedo ni ochung kowuok kuom yiero mar jatim nonro kata jachiw pesa, ibiro nyisi gima biro timore tok mano.

**Yor tudruok**

Inyalo wuoyo kod jotim nonro kuom penjo, parruok, kata ywagruok moro amora ma in go kuom nonro ni. Tudri kod jatij wa mar nonro e 0733-819 835. Ka diher mar penjo penjo kuom nonro kata ratiro magi kaka jachiwre ne ng’ato machielo kopogore gi jotim nonro kata diher mar wacho chandruok kata parruok moro amora ma in go kuom nonro , inyalo tudri gi Jagoro mar Ethical Review Committee, Kenya Medical Research Institute e seru@kemri.org kata P.O. BOX 54840-00200 Sime. 020-2722541, 0717719477. Bende inyalo tudri kod Washington University Human Research Protection Office e +1-314-747-6800.Komiti gi odich gi rito jochiwre mag nonro.

**YIE**

Osemiyi kopi mar andike mar yie ni mondo ikan.

**BEDO E NONRO EN KUOM CHIWRUOK.** In gi ratiro mar weyo bedo e nonro ni, kata wuok e saa asaya ma onge kum moro amora kata wito ber ma dine iyudo.

**Wach ma oa kuom Jachiwre**

Nonro ni ma owuo kuome malo kanyo oselerna. Achiwora mar bedo e nonro ni. Aseyudo thuolo mar penjo penjo. Ka an gi penjo e kinde mabiro kuom nonro ni, anyalo penjo jotim nonro ma ondik malo kanyo. Ka an gi penjo kuom ratiro maga kaka jachiwre nonro, anyalo tudra kod jok ma ondik malo kanyo.

Ayie mondo weche maga ma ochok oti godo e nonro ma kinde mabiro kendo ochiw ne jotim nonro mamoko maonge yie machielo kod/maonge gigo manyalo fwenya .

Ka diher mar bedo e nonro ni, onego iket seyi piny kanyo.

Tarik	Nying kod Seyi Jachiwre ne Yie

Tarik	Ng’at ma Kawo Yie (Nying kod seyi)

***Ka ok onyal somo kata ndiko***

Aseneno somo mar andike mar yie ni e yo ma kare ne jachiwre kendo ng’atno osebedo gi thuolo mar penjo penjo. Asiro ni ng’atno ochiwo yie mare ka en thuolo**.**

Nying janeno _____________________ KOD

Seyi janeno *__________________*KOD

Nying jachiwre __________________A

**Alama mar lith lwedo mathuon mar jachiwre**

**Tarik ________________________**

**Thuolo mar mako twak**

Achiwo thuolo ne twak mar kanyakla ma abiro bedo e mondo omak**: Ee ☐	Ooyo ☐**

Kopi ne:	1. Jotim nonro

2. Jachiwre nonro

**Study Title:** *Adaptive Strategies to Prevent and Treat Failures of Retention in Adolescent HIV Care (A4A Study)*

**KENYA MEDICAL RESEARCH INSTITUTE**

**WASHINGTON UNIVERSITY, ST. LOUIS**

**DHOLUO PARENTAL CONSENT FOR IN-DEPTH INTERVIEWS**

JOTIM NONRO KOD MIGEPE MA GITUDORE GODO:

| Jatim Nonro | Migao | Yor Tudruok |
| --- | --- | --- |
| Laktar Elizabeth Bukusi | Kenya Medical Research Institute | +254 733-617 503 |
| Laktar Elvin Geng | Washington University, St. Louis | +1-415-430-5589 (USA) |
| Laktar Lisa Abuogi | University of Colorado, Denver | +1-303-724-7430 (USA) |
| Laktar Zachary Kwena | Kenya Medical Research Institute | +254 722-386 714 |
| Starley Shade | University of California, San Francisco | +1-415-794-4354 (USA) |
| Lina Montoya | University of Notrh Carolina at Chapel Hill | +1-4105857470 (USA) |
| Eliud Akama | Kenya Medical Research Institute | +254 728-590 376 |

**Kama itimo e Nonro:** Kisumu County

Ikwayi mondo iyie ne nyathini mondo obed e twak . Boksi man piny no nyisi gik ma onego ipar kapok iyiero mar yie ne nyathini mondo odonj e twak. Wabiro chiwo wach matut e bwo boksi no. Yie ipenj penjo kuom wach moro amora kapok iyiero mar yie ne nyathini mondo obed kata kik obed twak. Bende inyalo hero mar wuoyo kod jomamoko (kuom ranyisi , anyuola ni , osiepe, kata jotim nonro) kuom nonro ni , kapok iyie mondo idonj.

- **Yie kuom chiwruok**. Ikwayi mondo iyie ne nyathini obed e twak. En kuomi mondo ka iyiero mar yie ne nyathini mondo obed kata kik obed e twak. Onge kum ne in kendo ok ibi wito gimoro amora ka iyiero mar ni ok idwar ni nyathini obedi e twak.

**Gima omiyo itime**. Watimo twak mondo wane gik ma rowere kod jomadongo matindo oseneno kod chenro mag konyo man e nonro ni . Dwaher mar ng’eyo paro, mor, kod gigo ma nyathini oseneno gi chenro mar konyo mane oyudo. Pach nyathini kod weche mochiwo biro konyowa ngéyo kaka chenro mar konyo ni tiyo **.**

- **Jochiwre:** Madirom rowere kod jomadongo matindo 60, jo higni 14 – 24 , ma orwaki e nonro mar A4A e Kisumu county, biro bedo e twak.
- **Chenro kod gik ma itimo.** Ka iyie mar chiwo thuolo ne nyathini mondo obed e twak , bende wabiro kwayo thuolo kuom nyathini bende . Ka uyie uduto mar donjo , wabiro keto saa ma nyathini biro bedo gi twak. Twak ibiro tim kod ngát ma tayo twak ma otiegi e kama opondo mondo orit maling’ling’mar nyathini . Nyathini ibiro penj penjo kuom mor mare kod gik ma oseneno gi chenro mar kony mane oyudo , mbese ma konyo e yor kompyuta/sime (ote machwok mag sime ma ioro gi simb ong’we yamo), mbese ma hocho wang’gi wang’, kata chiwo pesa ka itimo gima idwaro. Wabiro penjo nyathini kuom achiel kende kuom chenro mag konyo adek. E wi mano , wakwayo thuolo mari mar mako weche mag twak, ma bangé to wabiro ndiko piny kendo wang’iyo . Wach moro amora ma nyalo fwenyo nyathini ok bi ket e weche ma ingíyo. Wageno ni twak ni biro bedo kuom madirom dakika 45-60.
- **Rach.** Nitie thuolo matin mar fwenyruok ma chal mar kute mag ayaki. Kata kamano, wabiro keto chenro mag rito wach kuom chal mar nyathini mar kute mag ayaki.
- **Ber**. Nyalore ni nyathini ok bi yudo ber mochimore kode tir kuom bedo e nonro ni. Kuom bedo e nonro ni okonyo e jiwo yore ma gero ngima mag rowere ma odak kod kute mag ayaki.
- **Yiero mamoko.** In thuolo mar yiero mondo kik iyie ni nyathini mondo obed e twak. Ka iyiero ni nyathini kik bed e nonro ni , onge kum ma ibiro miye. Ok obi wito ber ma ojayudo, kendo podi onyalo yudo thieth mare e klinik kaka oyudo ga..

**Procedures and Activities**

Ka iyie mar chiwo thuolo ne nyathini mondo odonj, kendo nyathini bende oyie, gik moluwo gi biro timore:

1. Wabiro kwayi mondo iket seyi kata alama e andike ni kaka ranyisi ni kuom chiwruok ni iyie mar bedo e nonro kendo mondo wakwa nyathini thuolo.
2. Wabiro keto saa mar twak kod nyathini .
3. Wabiro penjo nyathini penjo kuom pache, mor kod gik ma oseneno kuom chenro mar konyo mane okete mondo oyud **, ma biro bedo** konyo ji e yor sime/kompyuta (ote machwok ma ioro gi kompyuta kod siro ma itimo gi mbese e yor sime), kony ma ichiwo gi mbese ka gibiro iri giwegi, kata pesa ma ichiwo ka itimo gima idwaro.
4. **Paro kod weche ma nyathini ochiwo biro konyowa ng’eyo kaka chenro mar konyo no tiyo .**
5. Twak ibiro ta kod ja ta twak ma otiegi e kama opondo mondo orit maling’ling’mari .
6. Wabiro kwaye mondo oyienwa mondo wamak twak kod dwoko ge ma ochiwo mondo wandik piny kendo ng’iyo gi bang’e .
7. Wach moro amora ma nyalo fwenyo nyathini ok bi bedo e weche ma ibiro ng’i .
8. Twak biro bedo kuom dakika 45-60.

**Rach**

Nitie thuolo matin mar fwenyruok ma chal mar kute mag ayaki . Kata kamano, wabiro keto chenro mag neno ni maling’ling’orit weche kuom chal mar nyathini mar kute mag ayaki.

**Ber**

Nyalore ni nyathini ok nyalo yudo ber mochimore kode tir kuom bedo e nonro ni. Kata kamano, bedo mar nyathini nyalo keto maber medo bedo mar rowere e thieth.

**Dwoko pesa**

**Nyathini ibiro dwokni pesa mar wuoth mar silings ma Kenya 500/=.**

**Yiero mamoko**

In thuolo mar yiero mondo kik iyie ne nyathini odonj e nonro. Ka iyiero ni ok iyie mondo nyathini obed e nonro ni, onge kum ma ibiro miye . Ok obi wito moro amora kuom bero ma oyudo ga, kendo podi onyalo yudo thieth mare ka owok e klinik kaka oyudo ga.

**Maling’ling’**

Wabiro timo kar nyalo wa mondo wane ni weche duto ma ochok ne nonro ni oket mopondo. Kata kamano, ok wanyal singo maling’ling’ma chutho. Weche nyathini ma nyalo fwenye inyalo chiw ka idwaro e yor chik. Ka weche ma owuok e nonro ni ogo chapa kata osom e buche mag sayans , nyingi kata nying nyathini kod weche mamoko ma nyalo fwenyo u ok bi ti godo.

Jotij sirkal kata mbalariany seche moko ng’iyo ga nonro kaka ma e mondo gine ni itimo gi e yo makare kendo kaluwore gi chik. Ka ng’iyo nonro ni otimore , andike gi ibiro ng’i. Jogo ma ng’iyo nonro gi biro rito maling’ling’mari. Andike mag nonro ok bi ti godo e kelo ni rach e yor chik. Migepe ma nyalo ng’iyo kendo/kata kopo andike gi mag nonro ne nonro, ngíyo kaka itimo tich, kod ng’inyo weche mochok oriwo:

- The Washington University, St. Louis
- The Kenya Medical Research Institute
- University of California San Francisco
- University of Colorado, Denver
- University of California, Berkeley

Bedo e nonro nyalo bedo gi wito maling’ling’, kata kamano wabiro keto yore mag rito maling’ling’mari. Wach moro amora momedore kuom nonro ibiro miyi koriwo nyaka dwoko mogik mag nonro.

**Bedo e nonro**

Nyathini nyalo yiero mar weyo twak e saa asaya , nyisi anyisa jatij nonro kendo gibiro gole. E wi mano, jatij nonro nyalo gole e nonro, ka gineno in en kuom ber mare. Ok be miye kum e yo moro amora ka oyiero mondo owe bedo e nonro.

**Joma ochiwo pesa**

Nonro ni ochung’ne gi National Institutes of Health. Onge moro amora kom jotim nonro man malo kanyo man kod chachni moro amora motudore gi yore pesa ma miyo dwaro mar gi nyalo chachni gi nonro. E thuolo ma nonro nyalo bedo ni ochung kowuok kuom yiero mar jatim nonro kata jachiw pesa, ibiro nyisi gima biro timore tok mano..

**Yor tudruok**

Inyalo wuoyo kod jotim nonro kuom penjo, parruok, kata ywagruok moro amora ma in go kuom nonro ni. Tudri kod jatij wa mar nonro e 0733-819 835. Ka diher mar penjo penjo kuom nonro kata ratiro magi kaka jachiwre ne ng’ato machielo kopogore gi jotim nonro kata diher mar wacho chandruok kata parruok moro amora ma in go kuom nonro , inyalo tudri gi Jagoro mar Ethical Review Committee, Kenya Medical Research Institute e seru@kemri.org kata P.O. BOX 54840-00200 Sime. 020-2722541, 0717719477. Bende inyalo tudri kod Washington University Human Research Protection Office e +1-314-747-6800.Komiti gi odich gi rito jochiwre mag nonro.

**YIE**

Osemiyi kopi mar andike mar yie ni mondo ikan.

**BEDO E NONRO EN KUOM CHIWRUOK.** In gi ratiro mar weyo bedo e nonro ni, kata wuok e saa asaya maonge kum moro amora kata wito ber ma dine iyudo. **Wach ma oa kuom jachiwre**

Oselerna wach kuom nonro ma ondik malo kanyo. Ayie mar chiwo thuolo mondo nyathina obed e nonro ni. Asebedo gi thuolo mar penjo penjo . Ka an gi penjo e kinde mabiro kuom nonro , anyalo penjo achiel kuom jotim nonro mondik malo kanyo. Ka an gi penjo kuom ratiro maga kaka jachiwre nonro , anyalo tudra gi jogo ma ondik malo kanyo.

Ayie ni weche ma ochok kuom nyathina mondo oti godo ne nonro ma kinde mabiro kendo ochiw ne jotim nonro mamoko maonge yie machielo mara ka en gi weche ma fwenye kata ma ok nyal fwenye.

Ka diher mar yie ne nyathini mondo obed e nonro ni , onego iket seyi piny kanyo.

Tarik	Nying kod Seyi Jachiwre ne Yie

Tarik	Ng’at ma Kawo Yie (Nying kod seyi )

***Ka ok onyal somo kata ndiko***

Aseneno somo mar andike mar yie ni e yo ma kare ne jachiwre kendo ng’atno osebedo gi thuolo mar penjo penjo. Asiro ni ng’atno ochiwo yie mare ka en thuolo**.**

Nying janeno _____________________ KOD

Seyi janeno *__________________*KOD

Nying jachiwre __________________KOD

**Alama lith lwedo mathuon**

**Tarik ________________________**

**Thuolo mar mako twak**

Achiwo thuolo ne twak mar kanyakla ma nyathina biro bedo mondo omak**: Ee ☐	Ooyo ☐**

Kopi ne:	1. Jotim nonro

2. Jachiwre nonro
