# Supplementary figures and images for: Examining and classifying reasons for missing viral loads among adults living with HIV: An extended outcome investigation and ascertainment approach in Western Kenya
Source: PLOS Glob Public Health. 2025 May 12;5(5):e0004038. doi: 10.1371/journal.pgph.0004038 (PMC12068717; doi:10.1371/journal.pgph.0004038)

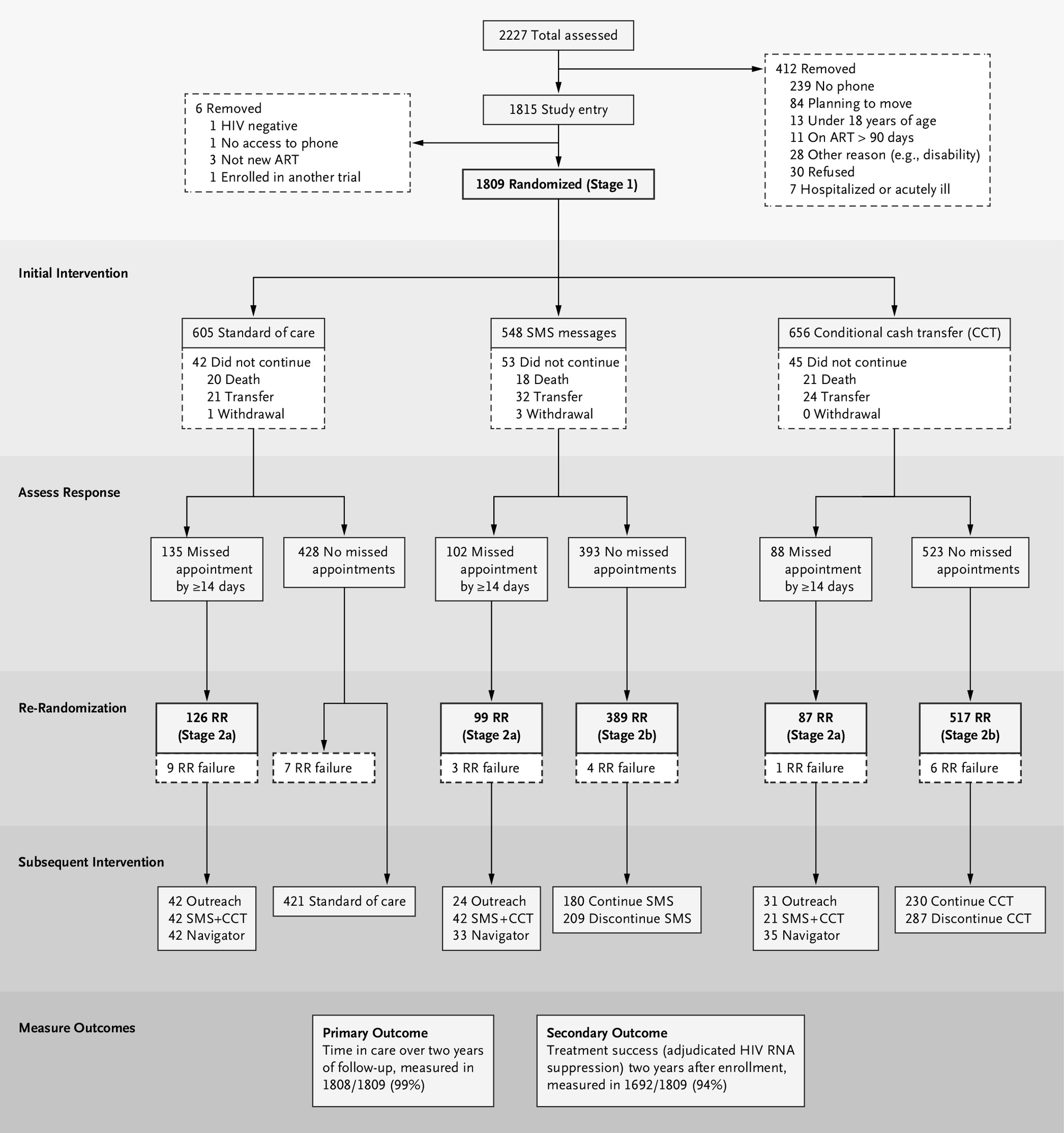

Supplement: S1 Fig — (JPG) [file pgph.0004038.s003.jpg]

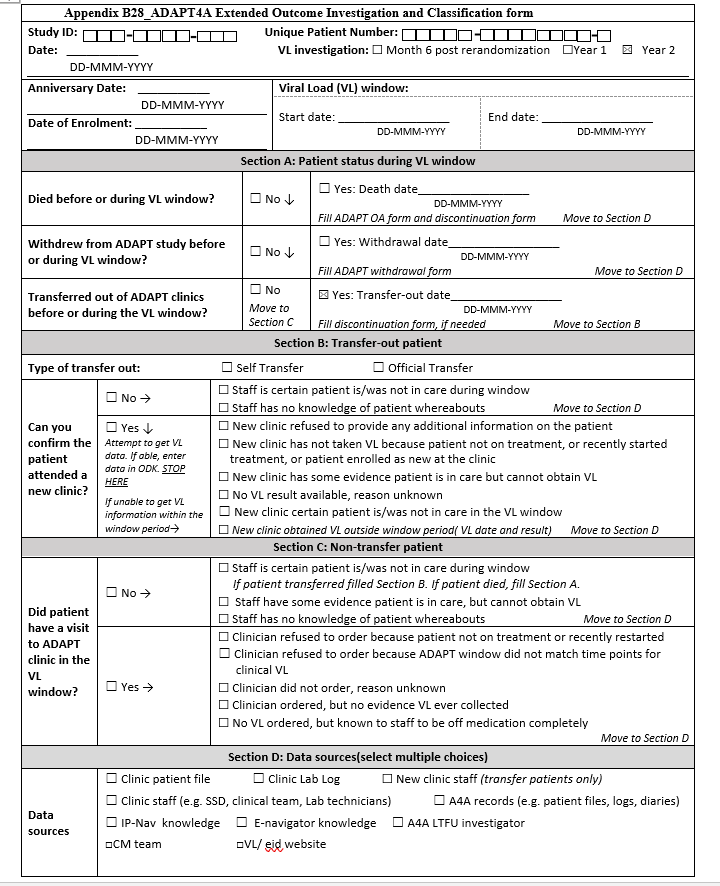

Supplement: S1 File — (DOCX) [file pgph.0004038.s004.docx]
